# Supplementary material for: Data-driven recombination detection in viral genomes
Source: Nat Commun. 2024 Apr 17;15:3313. doi: 10.1038/s41467-024-47464-5 (PMC11024102; doi:10.1038/s41467-024-47464-5)
Supplement: Supplementary file 1 — Supplementary Information [file 41467_2024_47464_MOESM1_ESM.pdf]

**Supplementary Information (document structure)**


- **Supplementary Notes 1 (page 2):**
  Guide for the interpretation of Supplementary Notes 2, 3, and 4.
- **Supplementary Notes 2 (page 4):**
  RecombinHunt output when run on the lineage consensus-genomes created using GISAID SARS-CoV-2 genome sequences. One result is provided for each retained Pango lineage (that passed quality controls and for which an unambiguous ground truth is available). Results correspond to those shown in Table 2.
- **Supplementary Notes 3 (page 62):**
  RecombinHunt output when run on the lineage consensus-genomes created using Nextstrain SARS-CoV-2 genome sequences. One result is provided for each retained Pango lineage (that passed quality controls and for which an unambiguous ground truth is available). Results correspond to those shown in Table 4.
- **Supplementary Notes 4 (page 126):**
  RecombinHunt output when run on the Nextstrain mpox genome sequences. One result is provided for each single genome sequence (as listed in Table 5c).
- **Supplementary Notes 5 (page 135):**
  Detailed report on GISAID cases on which RH disagrees with Pango lineage GT. This document extends the discussion presented in the 'Lineage analysis using consensus-genomes' section.
- **Supplementary Notes 6 (page 158):**
  Detailed report on Nextstrain cases on which RH disagrees with Pango lineage GT. This document extends the discussion presented in the 'Comparison with the RIPPLES method' section.

## Supplementary Notes 1

Guide for the interpretation of Supplementary Notes 2, 3, and 4.

Information known from ground truth (in order left to right):

- candidates,
- breakpoint on target (edge excluded)
- breakpoint genomic coordinates
- Rank (see definition below)

Information computed by Recombinhunt (in order left to right):

- candidates,
- breakpoint on target (edge excluded)
- breakpoint genomic coordinates

Detailed Information on the runtime analysis of Recombinhunt

Top (in order left to right):

- search direction of the first preferred candidate (L1)
- range of region candidates before gap resolution
- gap history (reported only if initial gap is not 0)

Bottom:

- additional notes on algorithm decisions

**Case 1 (1BP mid): XA** **test: OK**

```
Target: (75%) 33 samples      Number of changes: 36
GT: B.1.177 + B.1.1.7         GT BR: 12-14              GT BR coord: 21254 - 21765   Rank L1 L2: 2 1
BC: B.1.177.18 + B.1.1.7      BC BR: 13-14              BC BR coord: 21300 - 21301
Direction L1: <<              Initial region span: 1-13,14-36 Gap history (edge excluded):
Alt. candidates: [], []
Model 1BP/2BP comparison: -
Rec. model vs L1: 1.24e-202 Rec. model vs L2: 6.86e-101
Flags: Model_2BP_Bad_L1_opp
```

list of valid alternative candidates

If the target sequence is suitable for both 1BP and 2BP models, recombinhunt compares their AIC scores using this P-value.

P-values comparing the ricombinant model (either 1 BP or 2 BP) against the non-rercombinant model computed using only the candidate L1 or only the candidate L2.

**B.1.177.18 >>**

| | num_seq | t_ch_MAX | max_CL | CL@BC_t_ch_MAX | aic | PV | PV_OK | t_ch_MAX_OK | phyl_OK |
|---|---|---|---|---|---|---|---|---|---|
| B.1.177.18 | 395 | 13 | 31.403269 | NaN | NaN | NaN | * | * | * |
| B.1.177 | 34152 | 8 | 8.891676 | -8.623179 | 47.246358 | 4.122796e-18 | | | * |
| B.1.177.44 | 304 | 4 | 3.510834 | -45.289495 | 120.578991 | 4.917957e-34 | | | |

**B.1.1.7 <<**

| | num_seq | t_ch_MAX | max_CL | CL@BC_t_ch_MAX | aic | PV | PV_OK | t_ch_MAX_OK | phyl_OK |
|---|---|---|---|---|---|---|---|---|---|
| B.1.1.7 | 405547 | 14 | 34.611559 | NaN | NaN | NaN | * | * | * |
| Q.1 | 511 | 25 | 18.876925 | 12.310140 | 27.379720 | 2.066489e-10 | | | * |
| Q.3 | 2418 | 18 | 17.365214 | 11.027517 | 29.944966 | 5.745610e-11 | | | * |
| Q.7 | 4477 | 25 | 16.792133 | 10.116308 | 31.767384 | 2.301212e-11 | | | * |
| Q.4 | 518 | 28 | 13.787988 | -9.792570 | 71.585141 | 5.189835e-20 | | | * |

Columns:

1. caniddate name
2. number of sequences labeled with the candidate lineage name
3. position of maximum likelihood ratio in target changes
4. value of maximum likelihood ratio
5. value of likelihood ratio evaluated at t_ch_MAX of the first candidate
6. AIC score
7. P-value comparing each candidate with the first candidate

Columns 8,9,10 are described aside

Symbol * highlights candidates with acceptable p-value when compared to the first candidate, or having t_ch_MAX within distance 1 to the t_ch_MAX of the first candidate, or the candidate being on the same phylogenetic branch of first candidate.

The first candidate has always three asterisks.

## Definitions:

- Rank: When the GT candidate is chosen as first candidate also by our method, the rank of the region is evaluated 1.
  More in general, the rank of a region tells the position of the GT candidate in the list of alternative candidates for that region.
  As the list of candidates cannot exceed the size 10 (except when all the candidates have same max_CL), when the GT is not reported in the list, then the rank is symbolically assigned the value 11.
  Note: in the 2BP model, the rank of L1-opposite is meaningless (please refer to the rank of L1 instead).

## Supplementary Notes 2

RecombinHunt output when run on the lineage consensus-genomes created using GISAID SARS-CoV-2 genome sequences. One result is provided for each retained Pango lineage (that passed quality controls and for which an unambiguous ground truth is available). Results correspond to those shown in Table 2.

## Case 1 (1BP mid): XA test: OK

Target: (75%) 7 samples
Number of changes: 32
GT: B.1.177 + B.1.1.7
GT BR: 12-14
GT BR coord: 21254 - 21765
Rank L1 L2: 11 1
BC: B.1.177.18 + B.1.1.7
BC BR: 13-14
BC BR coord: 21257 - 21258
Direction L1: <<
Initial region span: 1-13,14-32
Gap history (edge excluded):
Alt. candidates: [], []
Model 1BP/2BP comparison: -
Rec. model vs L1: 1.02e-160
Rec. model vs L2: 3.12e-94
Flags: Model_2BP_Bad_L1_opp

### B.1.177.18 >>

| | num_seq | t_ch_MAX | max_CL | CL@BC_t_ch_MAX | aic | PV | PV_OK | t_ch_MAX_OK | phyl_OK |
|---|---|---|---|---|---|---|---|---|---|
| **B.1.177.18** | 214 | 13 | 24.658973 | None | None | None | * | * | * |

### B.1.1.7 <<

| | num_seq | t_ch_MAX | max_CL | CL@BC_t_ch_MAX | aic | PV | PV_OK | t_ch_MAX_OK | phyl_OK |
|---|---|---|---|---|---|---|---|---|---|
| **B.1.1.7** | 421412 | 14 | 31.788154 | NaN | NaN | NaN | * | * | * |
| **Q.1** | 3458 | 18 | 23.127376 | 17.594831 | 28.810338 | 6.842106e-07 | | | * |
| **Q.7** | 441 | 18 | 11.421698 | 5.926496 | 52.147007 | 5.847540e-12 | | | * |
| **Q.8** | 186 | 25 | 10.775085 | -1.100236 | 66.200473 | 5.200612e-15 | | | * |
| **Q.4** | 1080 | 21 | 8.931125 | -1.670187 | 67.340375 | 2.941079e-15 | | | * |
| **Q.3** | 2495 | 28 | 8.684782 | 0.805467 | 62.389066 | 3.494503e-14 | | | * |
| **Q.2** | 1179 | 29 | 6.484080 | -14.923551 | 93.847103 | 5.151495e-21 | | | * |
| **Q.6** | 44 | 27 | 3.624840 | -15.093599 | 94.187198 | 4.346135e-21 | | | * |

Cumulative Likelihood per-region

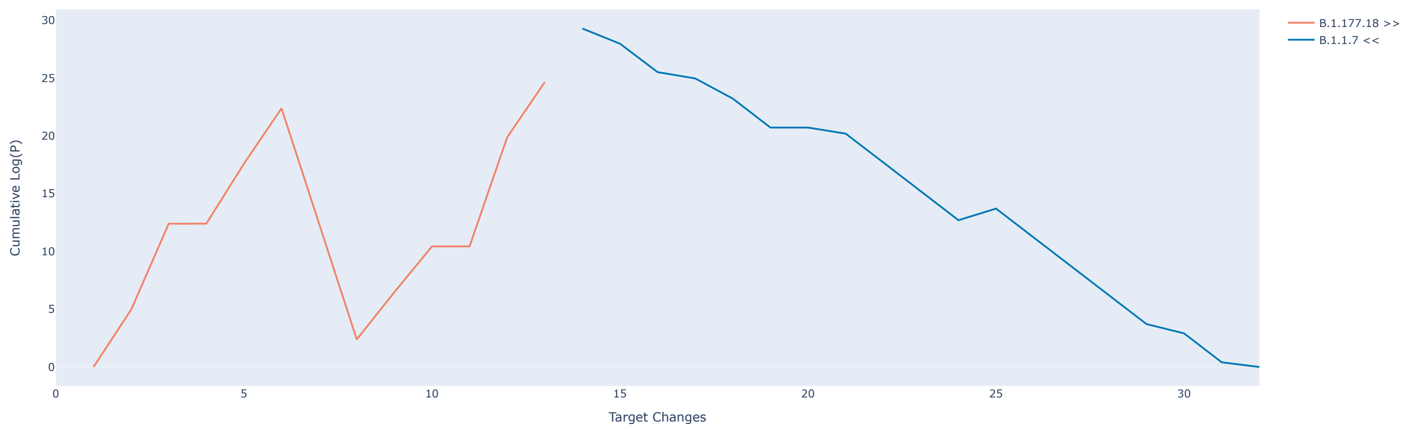


Cumulative Likelihood whole genome

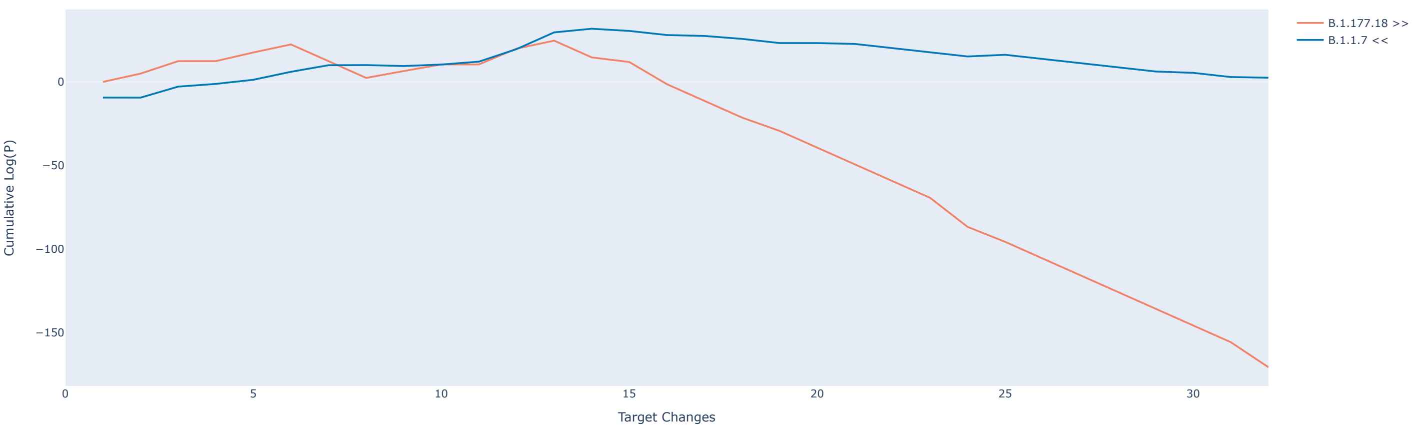


**Target sequence**

, 241_C|T, 445_T|C, 2019_T|C, 3037_C|T, 4999_C|T, 6286_C|T, 8090_C|T, 9430_C|T, 10323_A|G, 13945_C|T, 14408_C|T, 20410_G|A, 21255_G|C, 21765_TACATG|......, 21993_ATT|..., 23063_A|T, 23208_C|T, 23271_C|A, 23403_A|G, 23604_C|A, 23709_C|T, 24506_T|G, 24914_G|C, 25855_G|T, 27972_C|T, 28048_G|T, 28095_A|T, 28111_A|G, 28273_A|., 28280_GAT|CTA, 28881_GGG|AAC, 28977_C|T

## Case 2 (1BP mid): XAD test: OK

Target: (75%) 70 samples
Number of changes: 65
GT: BA.2* + BA.1*
GT BR: 54-56
GT BR coord: 26062 - 26529
Rank L1 L2: 1 11
BC: BA.2 + BA.1.14.1
BC BR: 55-56
BC BR coord: 26274 - 26275
Direction L1: >>
Initial region span: 1-55,56-65
Gap history (edge excluded):
Alt. candidates: [BA.2.34], [BA.1.1, BA.1.14]
Model 1BP/2BP comparison: 1BP vs 2BP: 8.46e-04
Rec. model vs L1: 7.19e-24
Rec. model vs L2: 2.84e-257
Flags: Model_1BP_Best

## BA.2 >>

| | num_seq | t_ch_MAX | max_CL | CL@BC_t_ch_MAX | aic | PV | PV_OK | t_ch_MAX_OK | phyl_OK |
|---|---|---|---|---|---|---|---|---|---|
| **BA.2** | 378689 | 55 | 44.173826 | NaN | NaN | NaN | * | * | * |
| **BA.2.9** | 108830 | 53 | 41.963775 | 39.979259 | 48.041481 | 1.507074e-02 | * | | * |
| **BA.2.34** | 325 | 55 | 34.962948 | 34.962948 | 58.074104 | 1.000340e-04 | * | * | * |
| **BA.2.3** | 36275 | 53 | 31.668248 | 28.895465 | 70.209069 | 2.311960e-07 | | | * |
| **BA.2.6** | 1333 | 52 | 31.313484 | 26.423707 | 75.152587 | 1.955568e-08 | | | * |
| **BA.2.65** | 1625 | 55 | 31.016450 | 31.016450 | 65.967101 | 1.926126e-06 | | * | * |
| **BA.2.51** | 288 | 47 | 28.869675 | 27.394430 | 73.211140 | 5.158680e-08 | | | * |
| **BA.2.36** | 5393 | 55 | 28.094961 | 28.094961 | 71.810078 | 1.038831e-07 | | * | * |
| **BA.2.49** | 769 | 55 | 27.696994 | 27.696994 | 72.606012 | 6.963490e-08 | | * | * |
| **BA.2.14** | 992 | 55 | 27.111998 | 27.111998 | 73.776005 | 3.879401e-08 | | * | * |

## BA.1.14.1 <<

| | num_seq | t_ch_MAX | max_CL | CL@BC_t_ch_MAX | aic | PV | PV_OK | t_ch_MAX_OK | phyl_OK |
|---|---|---|---|---|---|---|---|---|---|
| **BA.1.14.1** | 2183 | 56 | 7.913833 | NaN | NaN | NaN | * | * | * |
| **BA.1.1.14** | 8250 | 56 | 6.459875 | 6.459875 | 7.080249 | 0.233400 | * | * | |
| **BA.1.1** | 349352 | 56 | 6.344954 | 6.344954 | 7.310091 | 0.208045 | * | * | * |
| **BA.1.17.2** | 77688 | 56 | 6.254972 | 6.254972 | 7.490056 | 0.190139 | * | * | |
| **BA.1.13.1** | 1484 | 56 | 6.233160 | 6.233160 | 7.533680 | 0.186374 | * | * | |
| **BA.1.17.1** | 201 | 56 | 6.137022 | 6.137022 | 7.725955 | 0.168638 | * | * | |
| **BA.1.14** | 3706 | 56 | 6.120886 | 6.120886 | 7.758227 | 0.166127 | * | * | * |
| **BA.1.14.2** | 357 | 56 | 6.036716 | 6.036716 | 7.926568 | 0.152590 | * | * | |
| **BD.1** | 2033 | 56 | 5.744300 | 5.744300 | 8.511401 | 0.114178 | * | * | |
| **BA.1.13** | 893 | 56 | 5.675918 | 5.675918 | 8.648164 | 0.106459 | * | * | |

Cumulative Likelihood per-region

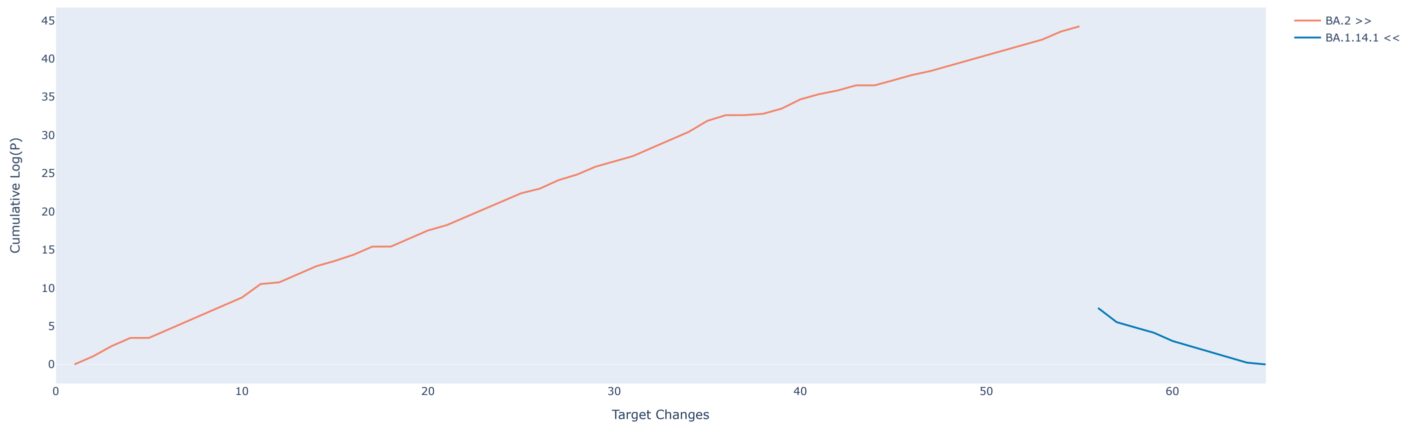


Cumulative Likelihood whole genome

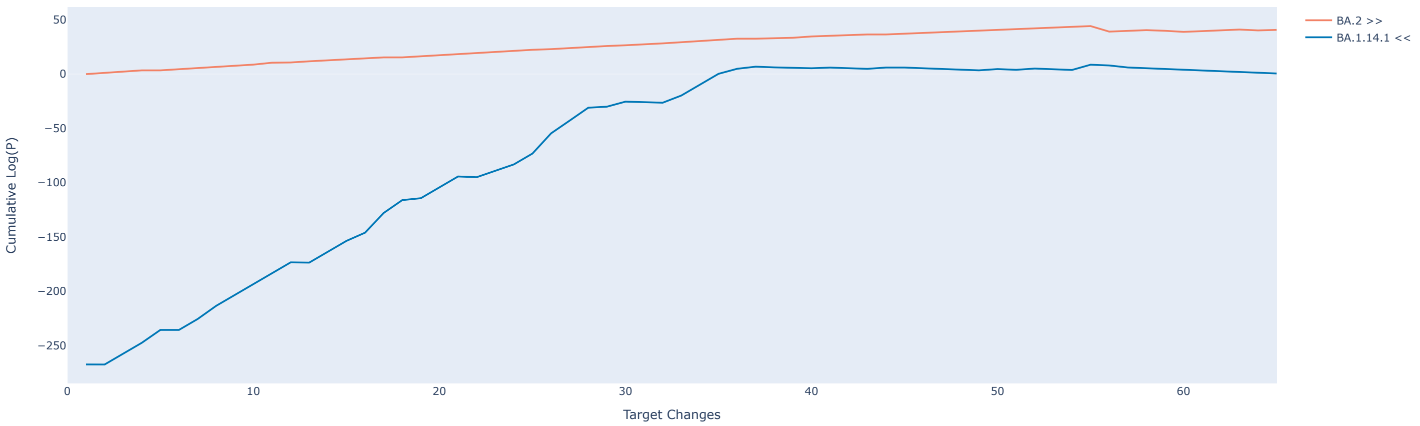


**Target sequence**

, 241_C|T, 670_T|G, 1250_A|G, 2790_C|T, 3037_C|T, 4184_G|A, 4321_C|T, 9344_C|T, 9424_A|G, 9534_C|T, 9866_C|T, 10029_C|T, 10198_C|T, 10447_G|A, 10449_C|A, 11288_TCTGGTTTT|........., 12880_C|T, 14408_C|T, 15714_C|T, 17410_C|T, 18163_A|G, 19955_C|T, 20055_A|G, 21618_C|T, 21633_TACCCCCTG|........., 21987_G|A, 22200_T|G, 22578_G|A, 22674_C|T, 22679_T|C, 22686_C|T, 22688_A|G, 22775_G|A, 22786_A|C, 22792_C|T, 22813_G|T, 22992_G|A, 22995_C|A, 23013_A|C, 23040_A|G, 23055_A|G, 23063_A|T, 23075_T|C, 23403_A|G, 23525_C|T, 23599_T|G, 23604_C|A, 23854_C|A, 23948_G|T, 24424_A|T, 24469_T|A, 25000_C|T, 25584_C|T, 26060_C|T, 26270_C|T, 26530_A|G, 26577_C|G, 26709_G|A, 27259_A|C, 27807_C|T, 28271_A|T, 28311_C|T, 28362_GAGAACGCA|........., 28877_AG|TC, 28881_GGG|AAC

## Case 3 (1BP mid): XAE          test: OK

Target: (75%) 50 samples | Number of changes: 67

GT: BA.2* + BA.1* | GT BR: 53–56 | GT BR coord: 24505 – 26049 | Rank L1 L2: 1 5

BC: BA.2 + BA.1.14 | BC BR: 55–56 | BC BR coord: 25584 – 25585

Direction L1: >> | Initial region span: 1–55,56–67 | Gap history (edge excluded):

Alt. candidates: [], [BA.1.1, BA.1]

Model 1BP/2BP comparison: –

Rec. model vs L1: 3.06e–26 | Rec. model vs L2: 1.53e–206

Flags: Model_2BP_Bad_L1_opp

## BA.2 >>

| | num_seq | t_ch_MAX | max_CL | CL@BC_t_ch_MAX | aic | PV | PV_OK | t_ch_MAX_OK | phyl_OK |
|---|---|---|---|---|---|---|---|---|---|
| **BA.2** | 378689 | 55 | 42.329452 | NaN | NaN | NaN | * | * | * |
| **BA.2.9** | 108830 | 55 | 28.555297 | 28.555297 | 78.889406 | 1.041342e-06 | | * | * |
| **BA.2.56** | 3106 | 55 | 25.831213 | 25.831213 | 84.337574 | 6.825603e-08 | | * | * |
| **BA.2.12.1** | 84020 | 49 | 15.782250 | 13.026069 | 109.947862 | 1.874995e-13 | | | * |
| **BA.2.12** | 2844 | 49 | 14.875869 | 12.091727 | 111.816546 | 7.360967e-14 | | | * |
| **BA.2.3** | 36275 | 55 | 14.590258 | 14.590258 | 106.819483 | 8.967494e-13 | | * | * |
| **BA.2.20** | 634 | 55 | 14.575944 | 14.575944 | 106.848111 | 8.833985e-13 | | * | * |
| **BA.2.31** | 1389 | 55 | 14.211050 | 14.211050 | 107.577901 | 6.132523e-13 | | * | * |
| **BA.2.10** | 28641 | 55 | 14.066062 | 14.066062 | 107.867876 | 5.304769e-13 | | * | * |
| **BA.2.62** | 249 | 55 | 13.126373 | 13.126373 | 109.747254 | 2.072190e-13 | | * | * |

## BA.1.14 <<

| | num_seq | t_ch_MAX | max_CL | CL@BC_t_ch_MAX | aic | PV | PV_OK | t_ch_MAX_OK | phyl_OK |
|---|---|---|---|---|---|---|---|---|---|
| **BA.1.14** | 3706 | 56 | 6.955366 | NaN | NaN | NaN | * | * | * |
| **P.1.7** | 2219 | 65 | 6.475888 | -89.667163 | 215.334326 | 1.092615e-42 | | | |
| **BA.1.1** | 349352 | 56 | 6.341983 | 6.341983 | 23.316033 | 5.406409e-01 | * | * | * |
| **BA.1.17.2** | 77688 | 56 | 4.733679 | 4.733679 | 26.532642 | 1.086091e-01 | * | * | |
| **BA.1** | 127335 | 56 | 4.170416 | 4.170416 | 27.659168 | 6.172909e-02 | * | * | * |
| **P.1** | 33918 | 65 | 3.665291 | -95.756110 | 227.512220 | 2.475220e-45 | | | |
| **BA.1.15** | 80931 | 56 | 0.711334 | -3.092500 | 42.185001 | 4.318575e-05 | * | * | |

Cumulative Likelihood per-region

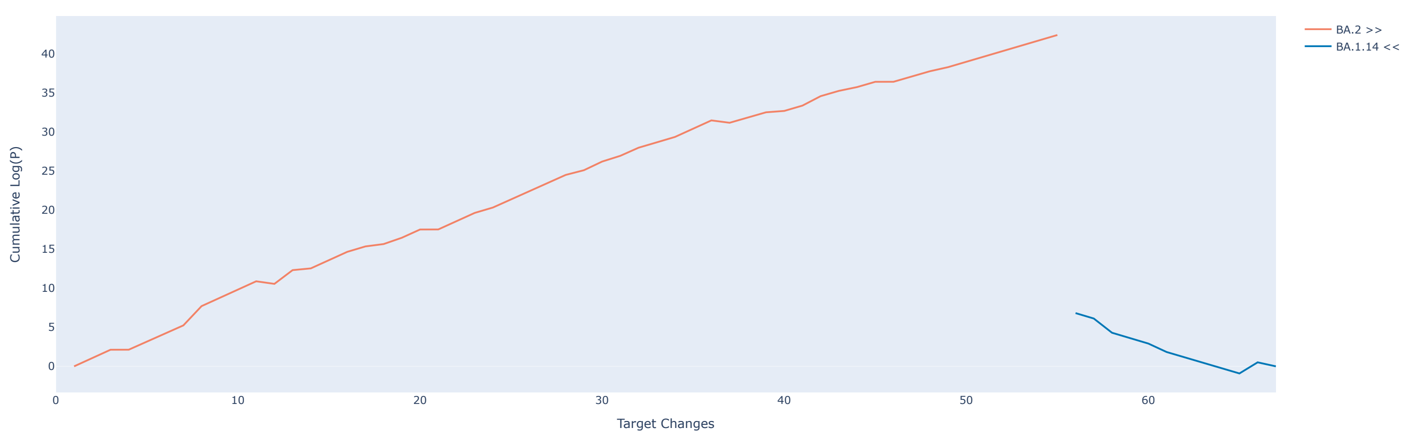


Cumulative Likelihood whole genome

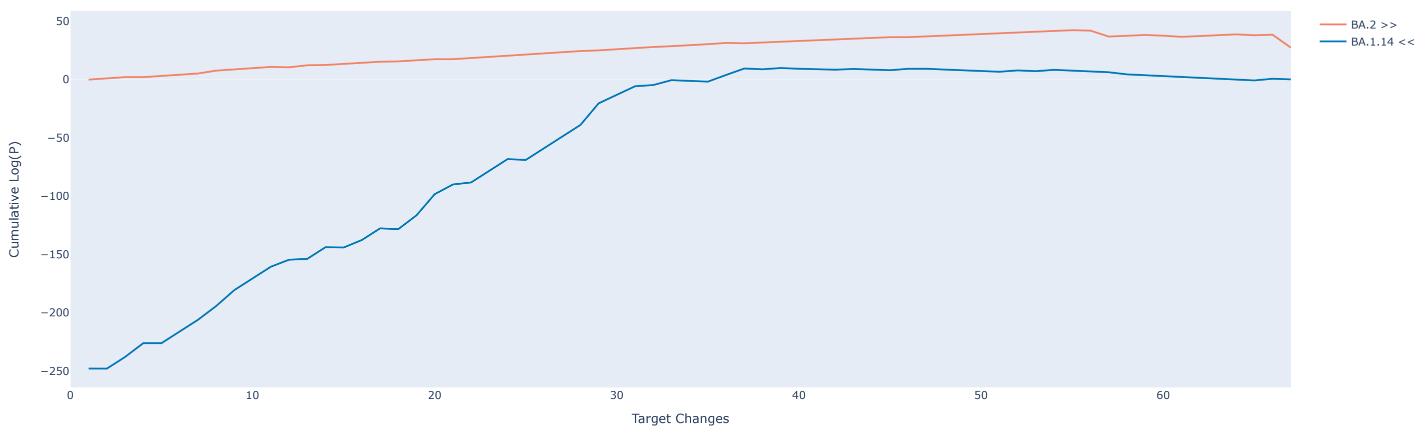


**Target sequence**

, 241_C|T, 670_T|G, 2790_C|T, 3037_C|T, 4184_G|A, 4321_C|T, 4975_G|A, 6390_A|C, 9344_C|T, 9424_A|G, 9534_C|T, 9803_C|T, 9866_C|T, 10029_C|T, 10198_C|T, 10447_G|A, 10449_C|A, 11249_C|T, 11288_TCTGGTTTT|........., 12880_C|T, 14408_C|T, 15714_C|T, 17410_C|T, 18163_A|G, 19955_C|T, 20055_A|G, 21618_C|T, 21633_TACCCCCTG|........., 21987_G|A, 22200_T|G, 22578_G|A, 22674_C|T, 22679_T|C, 22686_C|T, 22688_A|G, 22775_G|A, 22813_G|T, 22882_T|G, 22992_G|A, 22995_C|A, 23013_A|C, 23040_A|G, 23055_A|G, 23063_A|T, 23075_T|C, 23403_A|G, 23525_C|T, 23599_T|G, 23604_C|A, 23854_C|A, 23948_G|T, 24424_A|T, 24469_T|A, 25000_C|T, 25584_C|T, 26270_C|T, 26530_A|G, 26577_C|G, 26709_G|A, 27259_A|C, 27807_C|T, 28271_A|T, 28311_C|T, 28362_GAGAACGCA|........., 28877_AG|TC, 28881_GGG|AAC, 29748_T|C

## Case 4 (1BP mid): XAL    test: OK

Target: (75%) 72 samples
GT: BA.1* + BA.2*
BC: BA.1.1 + BA.2
Direction L1: <<
Alt. candidates: [BA.1, BA.1.1.16, BA.1.1.13, BA.1.15, B.1.1.529, BA.1.1.18], [BA.2.10, BA.2.3]
Model 1BP/2BP comparison:
Rec. model vs L1: 3.87e-96
Flags: Model_2BP_Bad_L1_opp

Number of changes: 61
GT BR: 15-17
BC BR: 15-16
Initial region span: 1-15,16-61
-
Rec. model vs L2: 6.09e-76

GT BR coord: 17412 - 19954
BC BR coord: 17420 - 17421
Gap history (edge excluded):

Rank_L1_L2: 2 1

## BA.1.1 >>

| | num_seq | t_ch_MAX | max_CL | CL@BC_t_ch_MAX | aic | PV | PV_OK | t_ch_MAX_OK | phyl_OK |
|---|---|---|---|---|---|---|---|---|---|
| **BA.1.1** | 349352 | 15 | 15.430761 | NaN | NaN | NaN | * | * | * |
| **BA.1** | 127335 | 15 | 10.823141 | 10.823141 | 20.353719 | 1.000170e-02 | * | * | * |
| **BA.1.1.16** | 1583 | 15 | 8.309145 | 8.309145 | 25.381710 | 8.087668e-04 | * | * | * |
| **BA.1.1.13** | 3131 | 15 | 8.186278 | 8.186278 | 25.627445 | 7.137342e-04 | * | * | * |
| **BA.1.15** | 80931 | 15 | 7.661159 | 7.661159 | 26.677682 | 4.222133e-04 | * | * | * |
| **BA.1.1.14** | 8250 | 13 | 6.401275 | 1.720061 | 38.559878 | 1.111278e-06 | | | * |
| **BA.1.1.15** | 6410 | 13 | 6.390062 | -4.902166 | 51.804331 | 1.481812e-09 | | | * |
| **B.1.1.529** | 1110 | 15 | 5.383698 | 5.383698 | 31.232603 | 4.340222e-05 | * | * | * |
| **BA.1.1.18** | 27122 | 15 | 4.687521 | 4.687521 | 32.624959 | 2.166094e-05 | * | * | * |
| **BA.1.1.10** | 1083 | 12 | 4.583548 | 2.322203 | 37.355594 | 2.024880e-06 | | | * |

## BA.2 <<

| | num_seq | t_ch_MAX | max_CL | CL@BC_t_ch_MAX | aic | PV | PV_OK | t_ch_MAX_OK | phyl_OK |
|---|---|---|---|---|---|---|---|---|---|
| **BA.2** | 378689 | 16 | 35.256640 | NaN | NaN | NaN | * | * | * |
| **BA.2.33** | 389 | 20 | 33.399925 | 26.280540 | 47.438920 | 1.265339e-04 | * | | * |
| **BA.2.63** | 120 | 20 | 33.320721 | 17.128729 | 65.742543 | 1.344041e-08 | | | * |
| **BA.2.40** | 17 | 20 | 33.232799 | 19.548834 | 60.902332 | 1.511490e-07 | | | * |
| **BA.2.23** | 3557 | 20 | 33.200450 | 25.713545 | 48.572910 | 7.191685e-05 | * | | * |
| **BA.2.10** | 28641 | 16 | 33.117026 | 33.117026 | 33.765948 | 1.176548e-01 | * | * | * |
| **BA.2.26** | 490 | 20 | 33.023107 | 25.884287 | 48.231426 | 8.524339e-05 | * | | * |
| **BA.2.19** | 365 | 20 | 32.920566 | 25.789071 | 48.421858 | 7.751803e-05 | * | | * |
| **BA.2.5** | 1908 | 20 | 32.487516 | 25.338643 | 49.322713 | 4.942768e-05 | * | | * |
| **BA.2.3** | 36275 | 16 | 32.475147 | 32.475147 | 35.049706 | 6.203851e-02 | * | * | * |

Cumulative Likelihood per-region

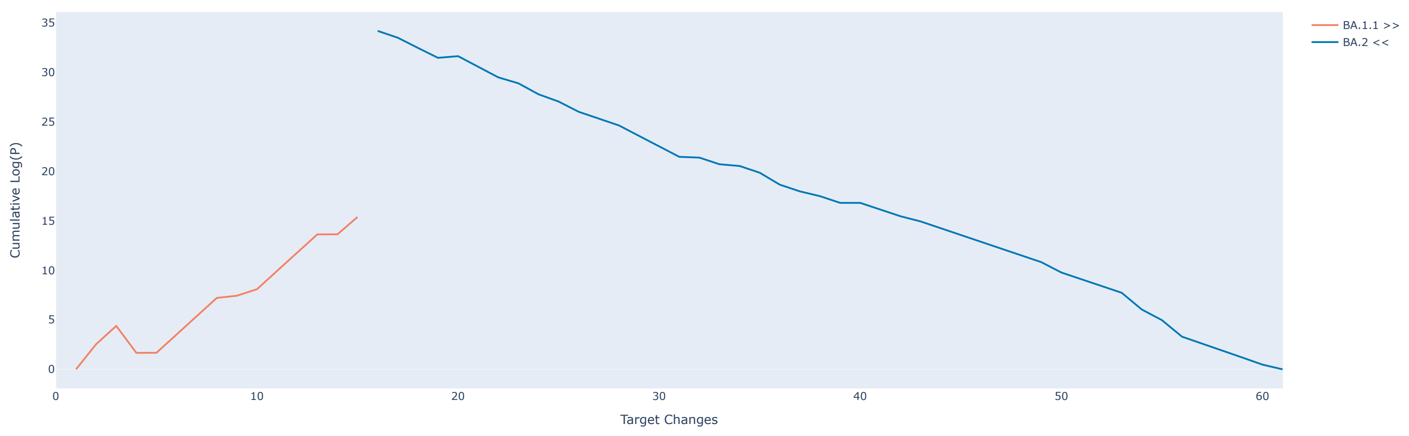


Cumulative Likelihood whole genome

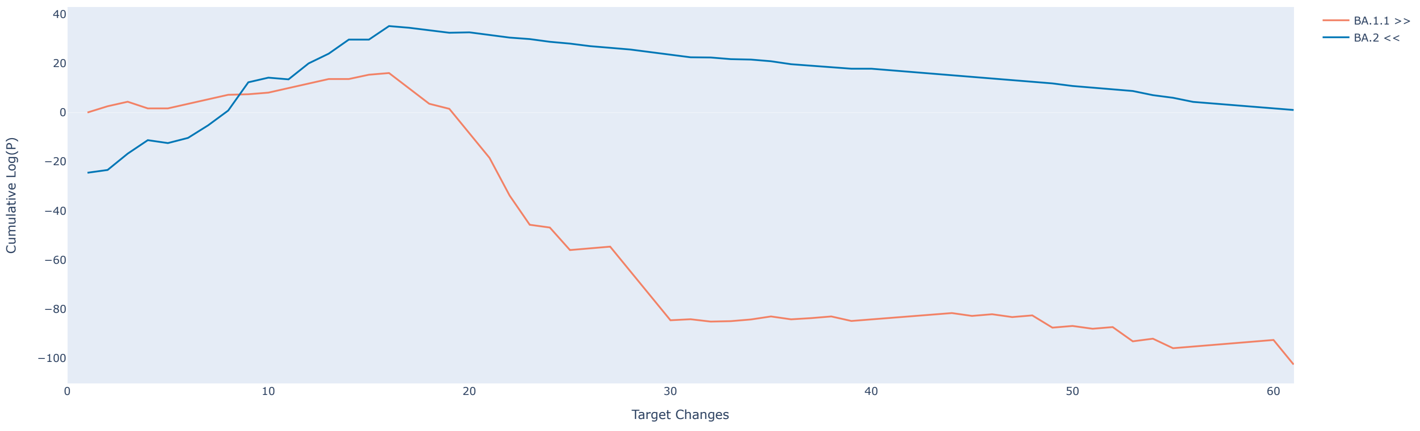


**Target sequence**

, 241_C|T, 2470_C|T, 2832_A|G, 2865_A|G, 3037_C|T, 5386_T|G, 6513_GTT|..., 8393_G|A, 10029_C|T, 10449_C|A, 11286_TGTCTGGTT|........., 11537_A|G, 13195_T|C, 14408_C|T, 15240_C|T, 18163_A|G, 19955_C|T, 20055_A|G, 21586_G|T, 21618_C|T, 21633_TACCCCCTG|........., 21987_G|A, 22200_T|G, 22578_G|A, 22674_C|T, 22679_T|C, 22686_C|T, 22688_A|G, 22775_G|A, 22786_A|C, 22813_G|T, 22992_G|A, 22995_C|A, 23013_A|C, 23040_A|G, 23055_A|G, 23063_A|T, 23075_T|C, 23403_A|G, 23525_C|T, 23599_T|G, 23604_C|A, 23854_C|A, 23948_G|T, 24424_A|T, 24469_T|A, 25000_C|T, 25584_C|T, 26060_C|T, 26270_C|T, 26577_C|G, 26709_G|A, 26858_C|T, 27259_A|C, 27382_GAT|CTC, 27807_C|T, 28271_A|T, 28311_C|T, 28362_GAGAACGCA|........., 28881_GGG|AAC, 29510_A|C

## Case 5 (1BP mid): XAN test: OK

Target: (75%) 123 samples  Number of changes: 71
GT: BA.2* + BA.5.1  GT BR: 21–28  GT BR coord: 17822 – 21766  Rank L1 L2: 2 2
BC: BJ.1 + BA.5.1.23  BC BR: 11–12  BC BR coord: 9866 – 9867
Direction L1: <<  Initial region span: 1–11,12–71  Gap history (edge excluded):
Alt. candidates: [BA.2], []
Model 1BP/2BP comparison: –
Rec. model vs L1: 5.39e–228  Rec. model vs L2: 3.15e–16
Flags: Model_2BP_Bad_L1_opp

## BJ.1 >>

| | num_seq | t_ch_MAX | max_CL | CL@BC_t_ch_MAX | aic | PV | PV_OK | t_ch_MAX_OK | phyl_OK |
|---|---|---|---|---|---|---|---|---|---|
| **BJ.1** | 104 | 11 | 11.563215 | NaN | NaN | NaN | * | * | * |
| **BA.2** | 378689 | 11 | 10.571641 | 10.571641 | 14.856718 | 3.697234e-01 | * | * | * |
| **BA.2.12.1** | 84020 | 11 | 9.474990 | 9.474990 | 17.050019 | 1.236871e-01 | * | * | |
| **BA.2.9** | 108830 | 11 | 8.523852 | 8.523852 | 18.952296 | 4.783489e-02 | * | * | |
| **BA.5.1** | 112751 | 10 | 8.481981 | 2.760063 | 30.479874 | 1.499813e-04 | * | * | |
| **BA.5.2.20** | 8940 | 10 | 7.548899 | -2.451101 | 40.902201 | 8.191489e-07 | | * | |
| **BA.4.1.4** | 381 | 10 | 7.317506 | -2.682494 | 41.364988 | 6.508413e-07 | | * | |
| **BE.1** | 14022 | 10 | 4.439221 | -1.517257 | 39.034514 | 2.086547e-06 | | * | |
| **BA.2.76.2** | 17 | 6 | 4.328819 | -5.094821 | 46.189642 | 5.816396e-08 | | | |
| **BA.2.2.1** | 394 | 6 | 4.318634 | -12.222865 | 60.445730 | 4.657301e-11 | | | |

## BA.5.1.23 <<

| | num_seq | t_ch_MAX | max_CL | CL@BC_t_ch_MAX | aic | PV | PV_OK | t_ch_MAX_OK | phyl_OK |
|---|---|---|---|---|---|---|---|---|---|
| **BA.5.1.23** | 5847 | 12 | 58.206044 | NaN | NaN | NaN | * | * | * |
| **BA.5.1** | 112751 | 2 | 54.346444 | 51.515203 | 30.969593 | 1.243283e-03 | * | | * |
| **BA.5.1.6** | 1479 | 12 | 51.946721 | 51.946721 | 30.106558 | 1.911246e-03 | * | * | |
| **BA.5.1.24** | 3729 | 22 | 44.467295 | 33.534616 | 66.930767 | 1.931769e-11 | | | |
| **DE.1** | 216 | 22 | 44.313322 | 39.643890 | 54.712221 | 8.699502e-09 | | | * |
| **BA.5.1.14** | 26 | 12 | 43.568392 | 43.568392 | 46.863217 | 4.406566e-07 | | * | |
| **BA.5** | 16505 | 12 | 41.756180 | 41.756180 | 50.487640 | 7.175560e-08 | | * | * |
| **BA.5.1.12** | 1348 | 12 | 37.494984 | 37.494984 | 59.010032 | 1.013354e-09 | | * | |
| **BA.5.1.17** | 738 | 12 | 35.894091 | 35.894091 | 62.211817 | 2.045927e-10 | | * | |
| **BA.5.1.22** | 9211 | 22 | 35.443542 | 32.711211 | 68.577578 | 8.465687e-12 | | | |

Cumulative Likelihood per-region

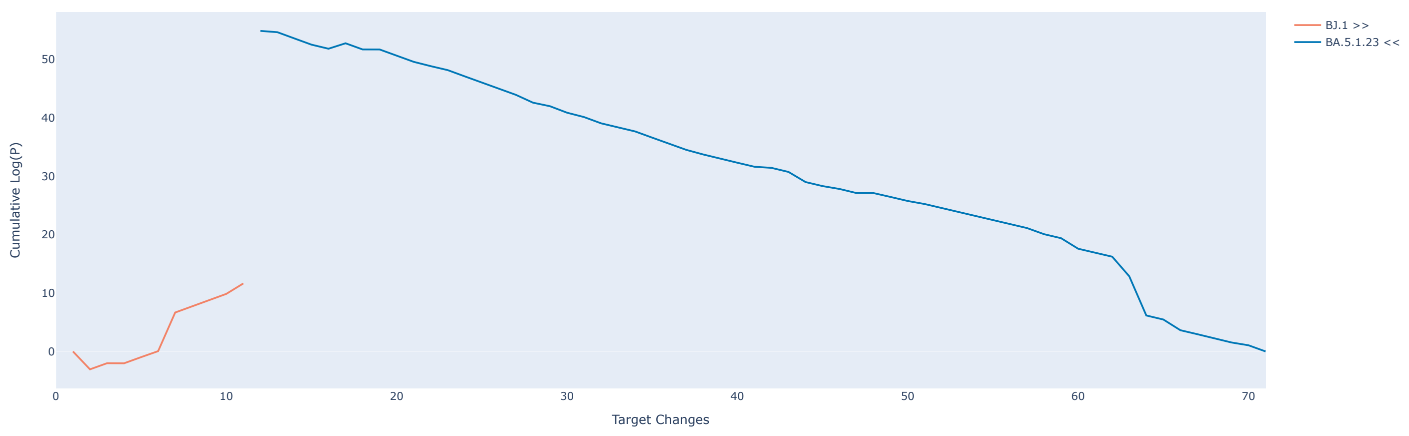


Cumulative Likelihood whole genome

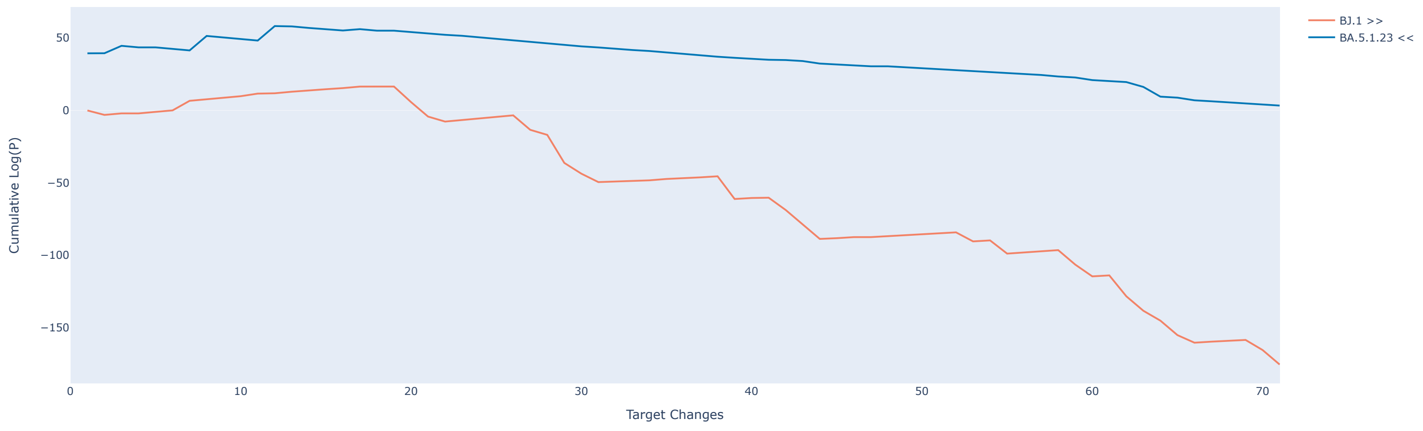


**Target sequence**

, 241_C|T, 670_T|G, 2790_C|T, 3037_C|T, 4184_G|A, 4321_C|T, 7973_C|A, 9344_C|T, 9424_A|G, 9534_C|T, 9866_C|T, 10029_C|T, 10198_C|T, 10447_G|A, 10449_C|A, 11288_TCTGGTTTT|........., 12880_C|T, 14408_C|T, 15714_C|T, 17410_C|T, 17822_C|T, 18163_A|G, 19955_C|T, 20055_A|G, 21618_C|T, 21633_TACCCCCTG|........., 21765_TACATG|......, 21987_G|A, 22200_T|G, 22578_G|A, 22674_C|T, 22679_T|C, 22686_C|T, 22688_A|G, 22775_G|A, 22786_A|C, 22813_G|T, 22882_T|G, 22917_T|G, 22992_G|A, 22995_C|A, 23013_A|C, 23018_T|G, 23055_A|G, 23063_A|T, 23075_T|C, 23403_A|G, 23525_C|T, 23599_T|G, 23604_C|A, 23854_C|A, 23948_G|T, 24424_A|T, 24469_T|A, 25000_C|T, 25584_C|T, 26060_C|T, 26270_C|T, 26529_G|A, 26577_C|G, 26709_G|A, 27438_T|C, 27696_T|C, 27807_C|T, 27889_C|T, 28271_A|T, 28311_C|T, 28362_GAGAACGCA|........., 28881_GGG|AAC, 29510_A|C, 29666_C|T

## Case 6 (1BP mid): XAQ          test: OK

Target: (75%) 5 samples

GT: BA.1* + BA.2*

BC: BA.1 + BA.2

Direction L1: <<

Alt. candidates: [BA.1.1.1, BA.1.1, BA.1.1.13, BA.1.1.16, BA.1.18, BA.1.15.1], [BA.2.9.5, BA.2.3, BA.2.9, BA.2.29, BA.2.1, BA.2.23, BA.2.27, BA.2.31, BA.2.26]

Number of changes: 64

GT BR: 17–18

BC BR: 16–17

Initial region span: 1–16,17–64

GT BR coord: 18165 – 19954

BC BR coord: 17420 – 17421

Gap history (edge excluded):

Rank L1 L2: 1 1

Model 1BP/2BP comparison:
Rec. model vs L1: 1.57e−91
Flags: Model_2BP_Bad_L1_opp

–
Rec. model vs L2: 3.26e−72

## BA.1 >>

| | num_seq | t_ch_MAX | max_CL | CL@BC_t_ch_MAX | aic | PV | PV_OK | t_ch_MAX_OK | phyl_OK |
|---|---|---|---|---|---|---|---|---|---|
| **BA.1** | 127335 | 16 | 13.887874 | NaN | NaN | NaN | * | * | * |
| **BA.1.1.1** | 29279 | 16 | 10.887419 | 5.749934 | 34.500133 | 2.916372e-04 | * | * | * |
| **BA.1.1** | 349352 | 16 | 8.828758 | 8.828758 | 28.342483 | 6.345560e-03 | * | * | * |
| **BA.1.1.13** | 3131 | 16 | 8.014651 | 8.014651 | 29.970698 | 2.808794e-03 | * | * | * |
| **BA.1.1.16** | 1583 | 16 | 7.879093 | 7.879093 | 30.241815 | 2.454088e-03 | * | * | * |
| **BA.1.20** | 11897 | 12 | 6.617948 | 2.400678 | 41.198644 | 1.023190e-05 | * | | * |
| **BA.1.12** | 726 | 7 | 5.966050 | -6.014614 | 58.029227 | 2.266566e-09 | | | * |
| **BA.1.18** | 16128 | 16 | 5.455903 | 5.455903 | 35.088195 | 2.171331e-04 | * | * | * |
| **BA.1.15.1** | 21857 | 16 | 5.369408 | 5.369408 | 35.261183 | 1.994394e-04 | * | * | * |
| **BA.1.1.15** | 6410 | 12 | 5.260483 | -6.191579 | 58.383157 | 1.902685e-09 | | | * |

## BA.2 <<

| | num_seq | t_ch_MAX | max_CL | CL@BC_t_ch_MAX | aic | PV | PV_OK | t_ch_MAX_OK | phyl_OK |
|---|---|---|---|---|---|---|---|---|---|
| **BA.2** | 378689 | 17 | 37.430700 | NaN | NaN | NaN | * | * | * |
| **BA.2.9.5** | 1119 | 17 | 36.473808 | 36.473808 | 29.052384 | 0.384812 | * | * | * |
| **BA.2.3** | 36275 | 17 | 33.269460 | 33.269460 | 35.461080 | 0.015608 | * | * | * |
| **BA.2.9** | 108830 | 17 | 30.260594 | 30.260594 | 41.478813 | 0.000769 | * | * | * |
| **BA.2.29** | 1418 | 17 | 29.474325 | 29.474325 | 43.051350 | 0.000351 | * | * | * |
| **BA.2.1** | 3218 | 17 | 28.577648 | 28.577648 | 44.844703 | 0.000143 | * | * | * |
| **BA.2.23** | 3557 | 17 | 28.561411 | 28.561411 | 44.877179 | 0.000141 | * | * | * |
| **BA.2.27** | 215 | 17 | 28.490718 | 28.490718 | 45.018565 | 0.000131 | * | * | * |
| **BA.2.31** | 1389 | 17 | 28.385206 | 28.385206 | 45.229587 | 0.000118 | * | * | * |
| **BA.2.26** | 490 | 17 | 28.145516 | 28.145516 | 45.708968 | 0.000093 | * | * | * |

Cumulative Likelihood per-region

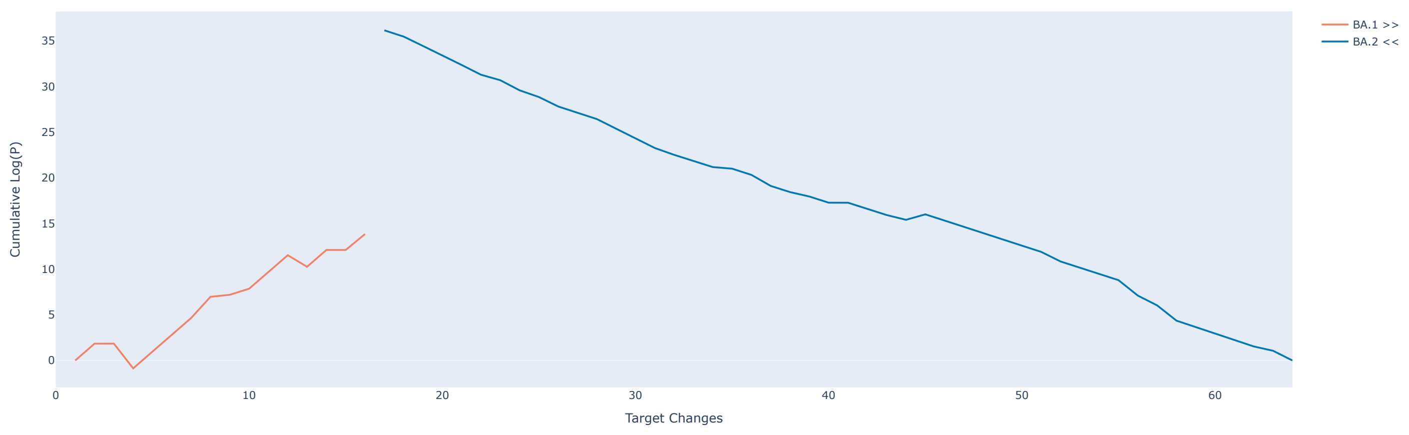


Cumulative Likelihood whole genome

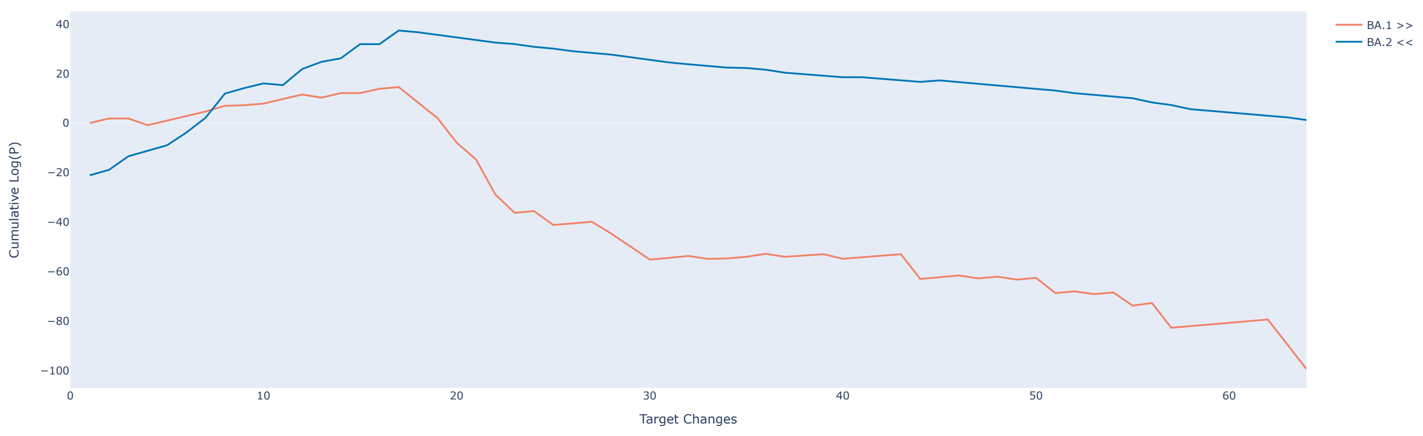


**Target sequence**

, 241_C|T, 2832_A|G, 3037_C|T, 4586_C|T, 5386_T|G, 6513_GTT|..., 8393_G|A, 9857_C|T, 10029_C|T, 10449_C|A, 11286_TGTCTGGTT|........., 11537_A|G, 12085_C|T, 13195_T|C, 14408_C|T, 15240_C|T, 18163_A|G, 19955_C|T, 20055_A|G, 21618_C|T, 21633_TACCCCCTG|........., 21987_G|A, 22200_T|G, 22578_G|A, 22674_C|T, 22679_T|C, 22686_C|T, 22688_A|G, 22775_G|A, 22786_A|C, 22813_G|T, 22882_T|G, 22992_G|A, 22995_C|A, 23013_A|C, 23040_A|G, 23055_A|G, 23063_A|T, 23075_T|C, 23403_A|G, 23525_C|T, 23599_T|G, 23604_C|A, 23779_A|T, 23854_C|A, 23948_G|T, 24424_A|T, 24469_T|A, 25000_C|T, 25584_C|T, 26060_C|T, 26270_C|T, 26577_C|G, 26709_G|A, 26858_C|T, 27259_A|C, 27382_GAT|CTC, 27807_C|T, 28271_A|T, 28311_C|T, 28362_GAGAACGCA|........., 28881_GGG|AAC, 29510_A|C, 29734_GAGGCCACGCGGAGTACGATCGAGTG|..........................

## Case 7 (1BP mid): XAV test: KO

Target: (75%) 42 samples
GT: BA.2* + BA.5*
BC: BA.5.1.24
Direction L1: >>

Number of changes: 70
GT BR: 19–22
BC BR:
Initial region span: 1–69

GT BR coord: 15959 − 17279
BC BR coord:
Gap history (edge excluded):

Rank L1 L2: 11 −

Alt. candidates: [BA.5]
Model 1BP/2BP comparison: –
Rec. model vs L1: – Rec. model vs L2: –
Flags: NotEnoughSpaceAfterL1, SingleCandidateGenome

## BA.5.1.24 >>

| | num_seq | t_ch_MAX | max_CL | CL@BC_t_ch_MAX | aic | PV | PV_OK | t_ch_MAX_OK | phyl_OK |
|---|---|---|---|---|---|---|---|---|---|
| **BA.5.1.24** | 3729 | 69 | 55.366186 | NaN | NaN | NaN | * | * | * |
| **BA.5** | 16505 | 70 | 46.645542 | 46.645542 | 78.708916 | 1.773146e-03 | * | * | * |
| **BA.5.3.1** | 4372 | 62 | 37.468326 | 27.227311 | 117.545378 | 6.527481e-12 | | | |
| **BA.5.2.1** | 93917 | 62 | 35.771453 | 28.713219 | 114.573561 | 2.896306e-11 | | | |
| **BA.5.1** | 112751 | 69 | 34.754075 | 32.302796 | 107.394408 | 1.049450e-09 | | * | * |
| **BA.5.5** | 17354 | 62 | 33.231756 | 32.063089 | 107.873821 | 8.255263e-10 | | | |
| **BA.5.1.3** | 5405 | 69 | 32.957703 | 30.515174 | 110.969651 | 1.752163e-10 | | * | |
| **BA.5.1.10** | 6066 | 67 | 31.894366 | 24.687676 | 122.624647 | 5.173794e-13 | | | |
| **BE.1** | 14022 | 62 | 30.047253 | 25.732811 | 120.534379 | 1.471116e-12 | | | |
| **BA.5.1.23** | 5847 | 63 | 29.248469 | 22.599424 | 126.801151 | 6.399317e-14 | | | |

Cumulative Likelihood per-region

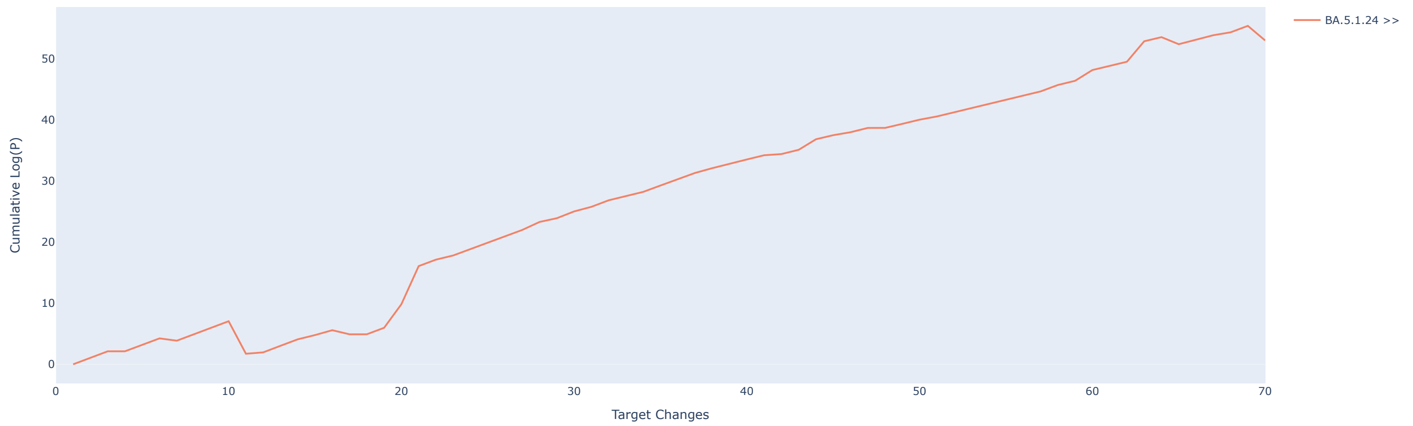


Cumulative Likelihood whole genome

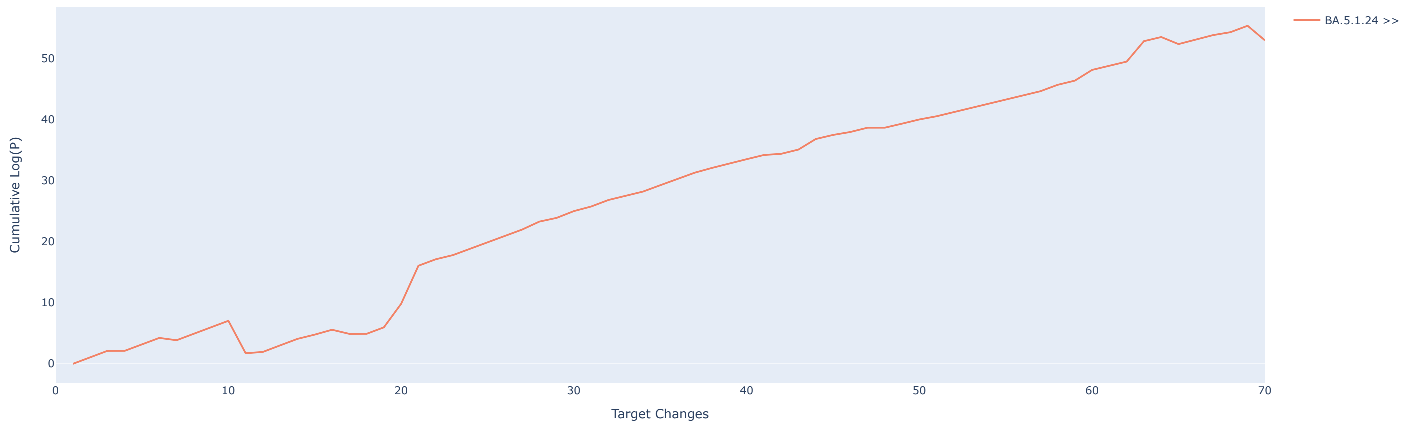


**Target sequence**

, 241_C|T, 670_T|G, 2790_C|T, 3037_C|T, 4184_G|A, 4321_C|T, 6606_C|T, 9344_C|T, 9424_A|G, 9534_C|T, 9866_C|T, 10029_C|T, 10198_C|T, 10447_G|A, 10449_C|A, 11288_TCTGGTTTT|........., 12880_C|T, 14408_C|T, 15714_C|T, 15960_C|T, 17278_G|T, 17410_C|T, 18163_A|G, 19955_C|T, 20055_A|G, 21618_C|T, 21633_TACCCCCTG|........., 21765_TACATG|......, 21987_G|A, 22200_T|G, 22578_G|A, 22674_C|T, 22679_T|C, 22686_C|T, 22688_A|G, 22775_G|A, 22786_A|C, 22813_G|T, 22882_T|G, 22917_T|G, 22992_G|A, 22995_C|A, 23013_A|C, 23018_T|G, 23055_A|G, 23063_A|T, 23075_T|C, 23403_A|G, 23525_C|T, 23599_T|G, 23604_C|A, 23854_C|A, 23948_G|T, 24424_A|T, 24469_T|A, 25000_C|T, 25584_C|T, 26060_C|T, 26270_C|T, 26529_G|A, 26577_C|G, 26709_G|A, 27438_T|C, 27807_C|T, 28271_A|T, 28311_C|T, 28362_GAGAACGCA|........., 28881_GGG|AAC, 29510_A|C, 29734_GAGGCCACGCGGAGTACGATCGAGTG|..........................

## Case 8 (1BP mid): XB test: KO

Target: (75%) 1244 samples Number of changes: 33
GT: B.1.634 + B.1.631 GT BR: 13–18 GT BR coord: 20999 – 22501 Rank L1 L2: 1 –
BC: B.1.634 BC BR: BC BR coord:
Direction L1: >> Initial region span: 1–17 Gap history (edge excluded):
Alt. candidates: []
Model 1BP/2BP comparison: –
Rec. model vs L1: – Rec. model vs L2: –
Flags: Model_1BP_NoL2, SingleCandidateGenome

# B.1.634 >>

| | num_seq | t_ch_MAX | max_CL | CL@BC_t_ch_MAX | aic | PV | PV_OK | t_ch_MAX_OK | phyl_OK |
|---|---|---|---|---|---|---|---|---|---|
| **B.1.634** | 99 | 17 | 30.942021 | NaN | NaN | NaN | * | * | * |
| **B.1.1.222** | 2333 | 5 | 6.112474 | -164.563536 | 691.127073 | 1.263362e-15 | | | |
| **B.1.189** | 62 | 3 | 3.123010 | -286.796737 | 935.593474 | 1.041630e-68 | | | |
| **B.1.157** | 99 | 3 | 2.684212 | -291.352232 | 944.704465 | 1.095221e-70 | | | |
| **AY.29** | 7365 | 3 | 2.015649 | -211.597572 | 785.195144 | 4.725925e-36 | | | |
| **B.1.142** | 215 | 3 | 1.924441 | -286.532388 | 935.064775 | 1.357692e-68 | | | |
| **B.1.36** | 2785 | 3 | 1.745751 | -161.785279 | 685.570559 | 2.036416e-14 | | | |
| **BL.1.1** | 36 | 3 | 1.495102 | -397.768085 | 1157.536170 | 6.635645e-117 | | | |
| **R.1** | 2286 | 4 | 1.131266 | -265.630327 | 893.260653 | 1.620153e-59 | | | |
| **AY.25.3** | 2371 | 3 | 1.010690 | -258.457611 | 878.915222 | 2.105947e-56 | | | |

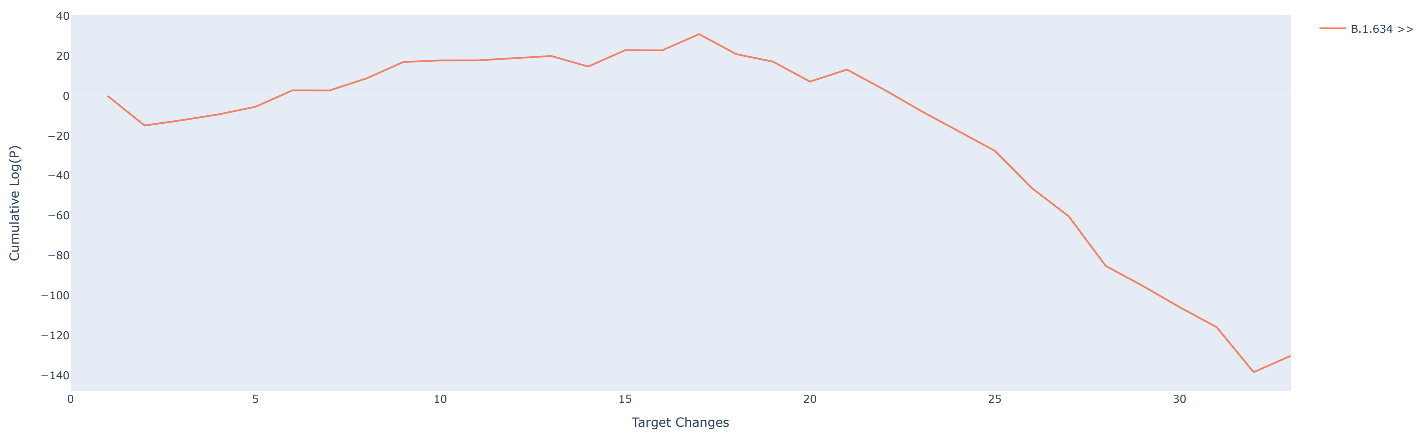


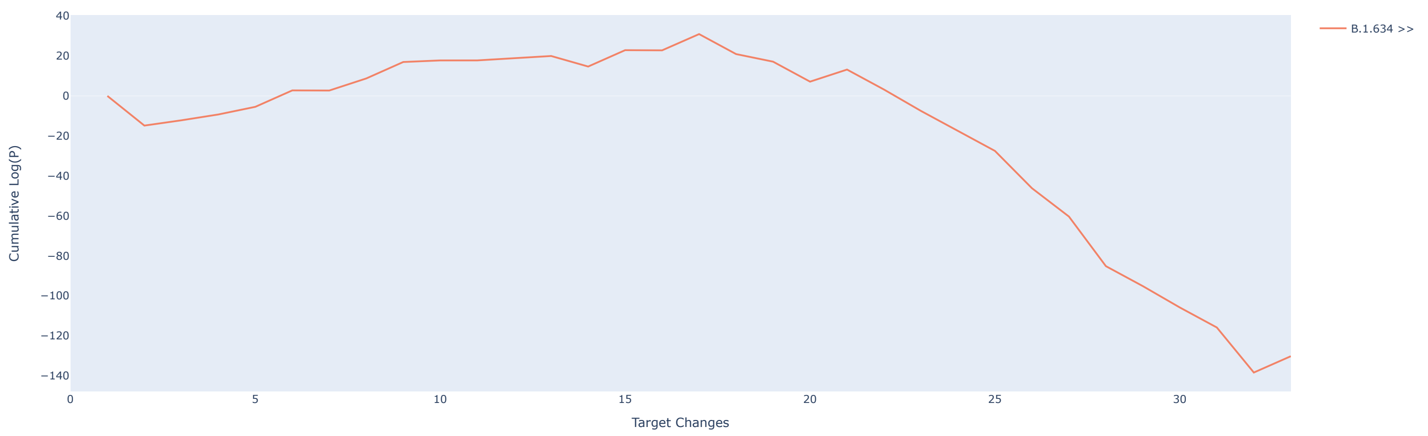


**Target sequence**

, 241_C|T, 3037_C|T, 3688_C|T, 3884_C|T, 6633_C|T, 7142_A|G, 9614_A|G, 9693_C|T, 9754_A|C, 11288_TCTGGTTTT|........., 14408_C|T, 15451_G|A, 16466_C|T, 21057_C|T, 21615_T|G, 21846_C|T, 22036_A|C, 22882_T|A, 23403_A|G, 23604_C|A, 23625_C|T, 23765_T|G, 24442_C|T, 24642_C|T, 26158_GTTA|...., 26222_C|T, 27389_C|T, 27769_C|T, 28048_G|A, 28273_A|., 28330_A|G, 28854_C|T, 28910_A|T

## Case 9 (1BP mid): XBB test: OK

Target: (75%) 1752 samples Number of changes: 85
GT: BJ.1 + BM.1.1.1 GT BR: 49–52 GT BR coord: 22891 – 22935 Rank L1 L2: 1 1
BC: BJ.1 + BM.1.1.1 BC BR: 51–52 BC BR coord: 22897 – 22898
Direction L1: >> Initial region span: 1–51,52–85 Gap history (edge excluded):
Alt. candidates: [], []
Model 1BP/2BP comparison: –
Rec. model vs L1: 7.31e−108 Rec. model vs L2: 2.99e−87
Flags: Model_2BP_Bad_L1_opp

## BJ.1 >>

| | num_seq | t_ch_MAX | max_CL | CL@BC_t_ch_MAX | aic | PV | PV_OK | t_ch_MAX_OK | phyl_OK |
|---|---|---|---|---|---|---|---|---|---|
| **BJ.1** | 104 | 51 | 63.297954 | NaN | NaN | NaN | * | * | * |
| **BA.2.10.1** | 4835 | 19 | 9.020675 | -77.326697 | 304.653394 | 8.459379e-62 | | | * |
| **BA.2** | 378689 | 19 | 7.838547 | -68.849347 | 287.698693 | 4.054938e-58 | | | * |
| **BL.1.4** | 25 | 6 | 7.251440 | -48.957846 | 247.915691 | 1.762388e-49 | | | |
| **BA.2.9.4** | 419 | 19 | 6.048983 | -131.990934 | 413.981869 | 1.536847e-85 | | | |
| **BA.2.56** | 3106 | 19 | 5.686764 | -102.486341 | 354.972681 | 1.001127e-72 | | | |
| **BA.2.11** | 598 | 19 | 5.154259 | -136.044464 | 422.088929 | 2.664198e-87 | | | |
| **BA.2.44** | 567 | 19 | 5.069362 | -162.789513 | 475.579027 | 6.461905e-99 | | | |
| **BA.2.14** | 992 | 19 | 4.692635 | -149.000568 | 448.001136 | 6.299143e-93 | | | |
| **BA.5** | 16505 | 11 | 4.667817 | -50.691672 | 251.383344 | 3.124439e-50 | | | |

## BM.1.1.1 <<

| | num_seq | t_ch_MAX | max_CL | CL@BC_t_ch_MAX | aic | PV | PV_OK | t_ch_MAX_OK | phyl_OK |
|---|---|---|---|---|---|---|---|---|---|
| **BM.1.1.1** | 41 | 52 | 38.676273 | NaN | NaN | NaN | * | * | * |
| **DS.1** | 115 | 52 | 34.710282 | 34.710282 | 2.579436 | 1.896804e-02 | * | * | |
| **BY.1** | 683 | 52 | 34.575429 | 34.575429 | 2.849142 | 1.657268e-02 | * | * | |
| **BA.2.75.7** | 38 | 52 | 34.425073 | 34.425073 | 3.149854 | 1.426423e-02 | * | * | |
| **BM.4.1.1** | 570 | 52 | 33.628381 | 33.628381 | 4.743237 | 6.441460e-03 | * | * | |
| **BN.1.5** | 1645 | 52 | 33.343818 | 33.343818 | 5.312363 | 4.844070e-03 | * | * | |
| **BN.4** | 22 | 57 | 30.200919 | 25.315431 | 21.369137 | 1.576978e-06 | | | |
| **BN.1** | 2068 | 57 | 30.149077 | 25.284837 | 21.430327 | 1.530372e-06 | | | |
| **CJ.1** | 538 | 57 | 30.132383 | 20.859605 | 30.280789 | 1.832499e-08 | | | * |
| **BN.1.8** | 260 | 57 | 30.111528 | 25.264853 | 21.470294 | 1.500068e-06 | | | |

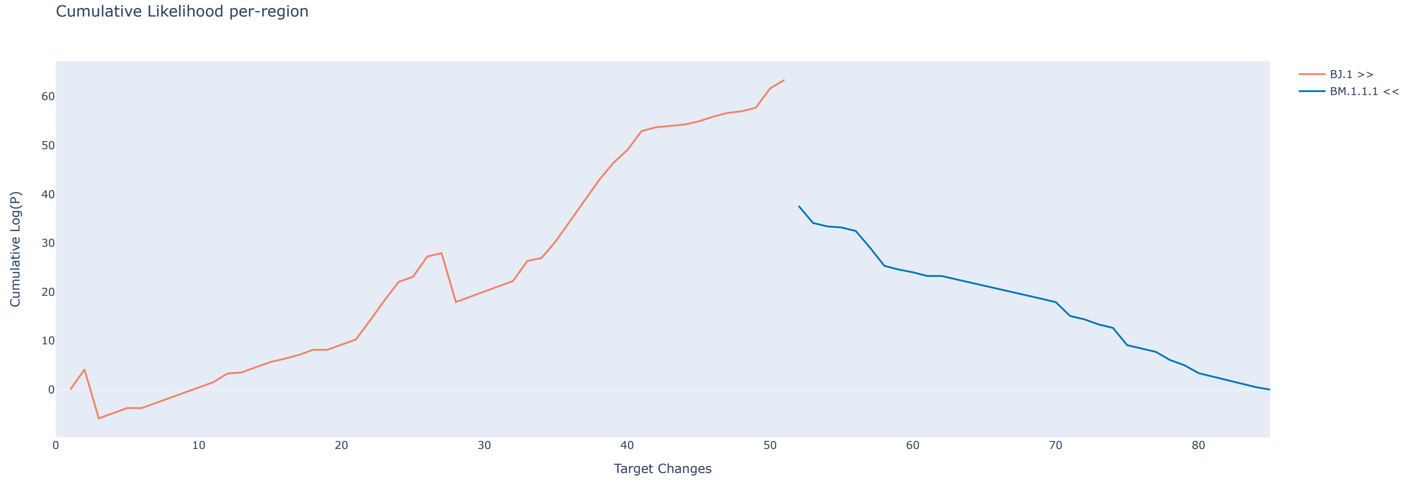


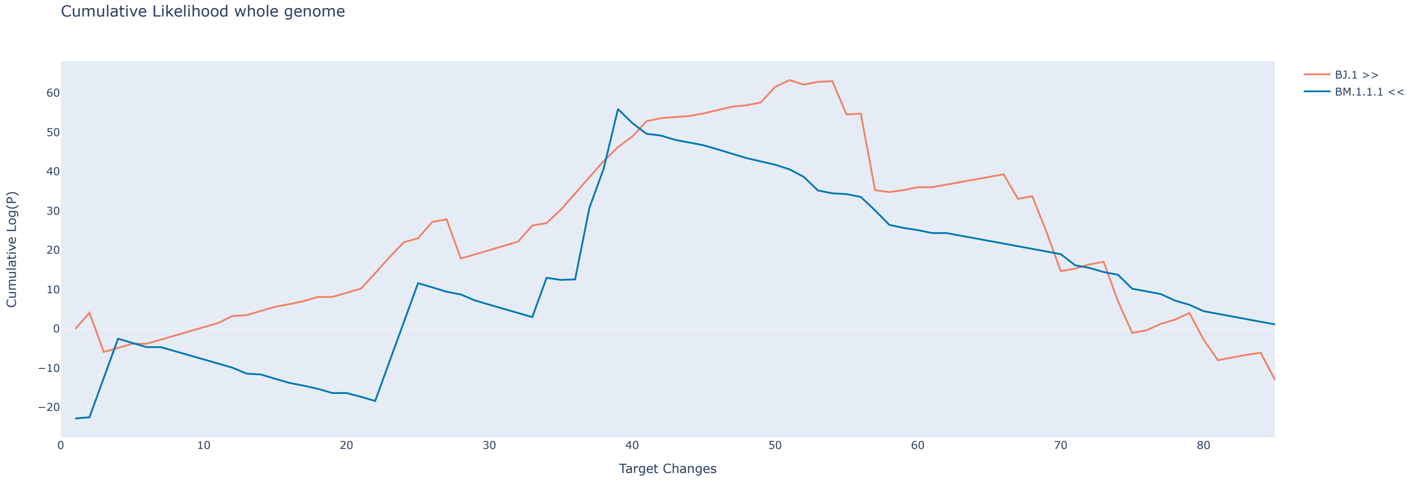


**Target sequence**

, 241_C|T, 405_A|G, 510_G|A, 670_T|G, 2790_C|T, 3037_C|T, 4184_G|A, 4321_C|T, 9344_C|T, 9424_A|G, 9534_C|T, 9866_C|T, 10029_C|T, 10198_C|T, 10447_G|A, 10449_C|A, 11288_TCTGGTTTT|........., 12880_C|T, 14408_C|T, 15451_G|A, 15714_C|T, 15738_C|T, 15939_T|C, 16342_T|C, 17410_C|T, 17859_T|C, 18163_A|G, 19326_A|G, 19955_C|T, 20055_A|G, 21618_C|T, 21633_TACCCCCTG|........., 21810_T|C, 21987_G|A, 21991_TTA|..., 22000_C|A, 22109_C|G, 22200_T|A, 22577_GG|CA, 22599_G|C, 22664_C|A, 22674_C|T, 22679_T|C, 22686_C|T, 22688_A|G, 22775_G|A, 22786_A|C, 22813_G|T, 22882_T|G, 22895_GT|CC, 22898_G|A, 22942_T|G, 22992_G|A, 22995_C|A, 23013_A|C, 23019_T|C, 23031_T|C, 23055_A|G, 23063_A|T, 23075_T|C, 23403_A|G, 23525_C|T, 23599_T|G, 23604_C|A, 23854_C|A, 23948_G|T, 24424_A|T, 24469_T|A, 25000_C|T, 25416_C|T, 25584_C|T, 26060_C|T, 26270_C|T, 26275_A|G, 26577_C|G, 26709_G|A, 26858_C|T, 27259_A|C, 27382_GAT|CTC, 27807_C|T, 28271_A|T, 28311_C|T, 28362_GAGAACGCA|........., 28881_GGG|AAC, 29510_A|C

## Case 10 (1BP mid): XBD test: OK

Target: (75%) 141 samples    Number of changes: 82
GT: BA.2.75.2 + BA.5.2.1    GT BR: 52–66    GT BR coord: 23012 – 24616    Rank_L1_L2: 1 11
BC: BA.2.75.2 + BF.3    BC BR: 65–66    BC BR coord: 24494 – 24495
Direction L1: >>    Initial region span: 1–65,66–82    Gap history (edge excluded):
Alt. candidates: [CA.3, CA.6, CA.2], []
Model 1BP/2BP comparison:    1BP vs 2BP: 7.99e–05
Rec. model vs L1: 4.78e–64    Rec. model vs L2: 9.53e–170

Flags: Model_1BP_Best

## BA.2.75.2 >>

| | num_seq | t_ch_MAX | max_CL | CL@BC_t_ch_MAX | aic | PV | PV_OK | t_ch_MAX_OK | phyl_OK |
|---|---|---|---|---|---|---|---|---|---|
| **BA.2.75.2** | 1428 | 65 | 105.987435 | NaN | NaN | NaN | * | * | * |
| **CA.3** | 300 | 65 | 101.645497 | 101.645497 | -55.290994 | 1.303653e-02 | * | * | * |
| **BY.1** | 683 | 65 | 99.897642 | 99.897642 | -51.795285 | 2.276764e-03 | * | * | |
| **CA.6** | 22 | 65 | 99.381524 | 99.381524 | -50.763048 | 1.353583e-03 | * | * | * |
| **CA.2** | 43 | 65 | 98.769967 | 98.769967 | -49.539934 | 7.354706e-04 | * | * | * |
| **BA.2.75.6** | 24 | 50 | 88.288689 | 65.001697 | 17.996605 | 1.586502e-18 | | | |
| **CA.3.1** | 124 | 65 | 87.807027 | 87.807027 | -27.614054 | 1.272115e-08 | | * | * |
| **BM.4.1.1** | 570 | 65 | 87.727029 | 87.727029 | -27.454057 | 1.174310e-08 | | * | |
| **CA.4** | 46 | 53 | 85.952039 | 78.260060 | -8.520121 | 9.103020e-13 | | | * |
| **BM.2** | 118 | 53 | 85.303531 | 81.823332 | -15.646664 | 3.216957e-11 | | | |

## BF.3 <<

| | num_seq | t_ch_MAX | max_CL | CL@BC_t_ch_MAX | aic | PV | PV_OK | t_ch_MAX_OK | phyl_OK |
|---|---|---|---|---|---|---|---|---|---|
| **BF.3** | 929 | 66 | 22.775840 | NaN | NaN | NaN | * | * | * |
| **BF.23** | 175 | 66 | 22.629653 | 22.629653 | -11.259307 | 8.650223e-01 | * | * | |
| **BF.25** | 355 | 67 | 18.475533 | 8.475533 | 17.048934 | 6.160116e-07 | | * | |
| **BF.17** | 357 | 67 | 18.459636 | 8.459636 | 17.080729 | 6.068404e-07 | | * | |
| **BF.1.1** | 147 | 67 | 18.405257 | 8.405257 | 17.189486 | 5.743654e-07 | | * | |
| **BF.16** | 246 | 67 | 18.249306 | 8.249306 | 17.501388 | 4.918953e-07 | | * | |
| **BF.2** | 1209 | 66 | 18.216945 | 18.216945 | -2.433891 | 1.046206e-02 | * | * | |
| **BF.1** | 5940 | 67 | 18.215021 | 17.328097 | -0.656193 | 4.317840e-03 | * | * | |
| **BF.14** | 3321 | 67 | 18.214516 | 14.964595 | 4.070809 | 4.056580e-04 | * | * | |
| **BF.28** | 4033 | 67 | 18.151409 | 16.786686 | 0.426629 | 2.503664e-03 | * | * | |

Cumulative Likelihood per-region

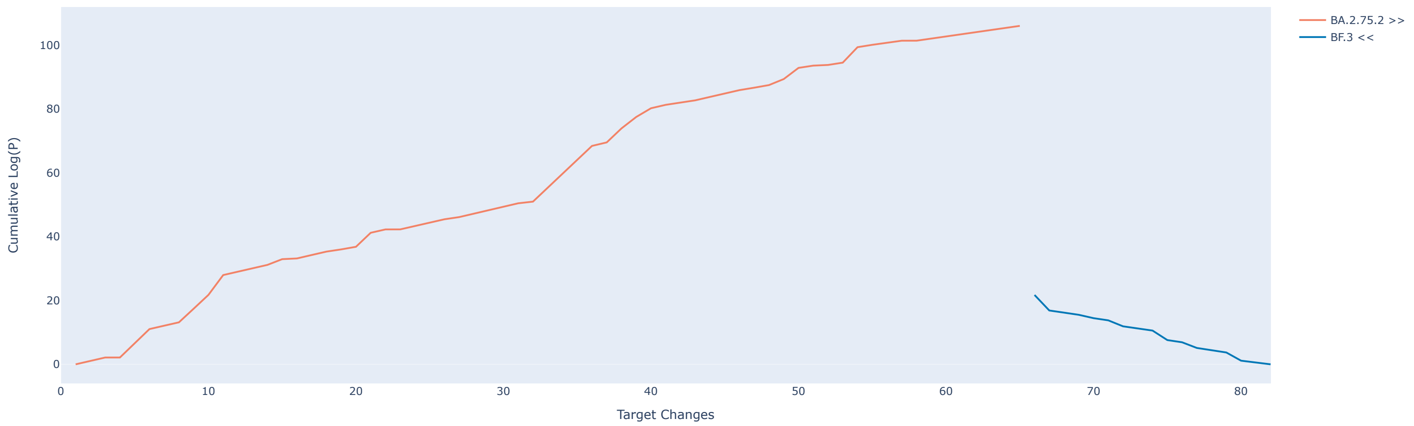


Cumulative Likelihood whole genome

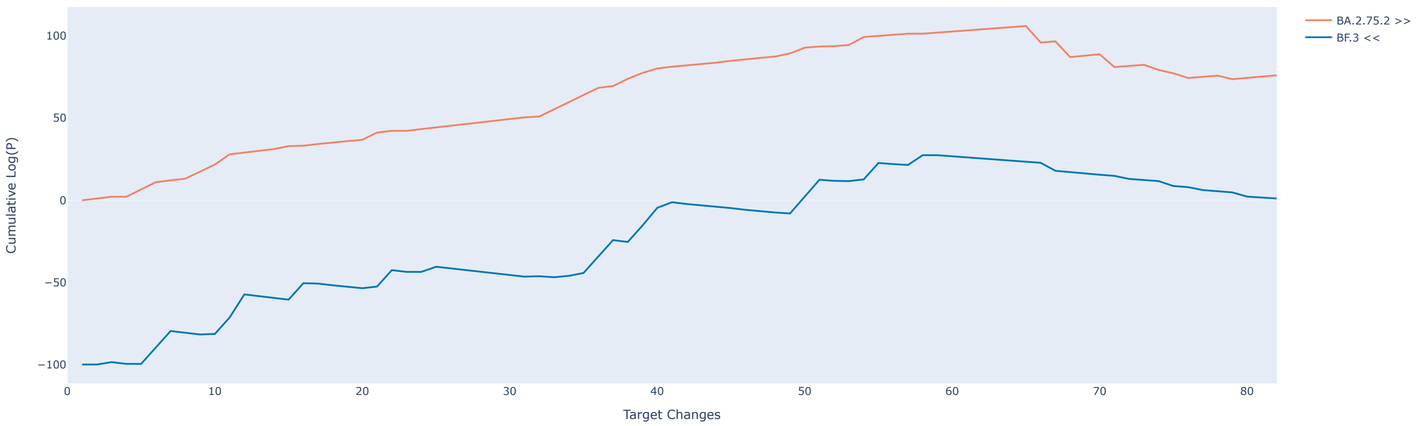


**Target sequence**

, 241_C|T, 670_T|G, 2790_C|T, 3037_C|T, 3796_C|T, 3927_C|T, 4184_G|A, 4321_C|T, 4586_C|T, 5183_C|T, 5192_C|T, 9344_C|T, 9424_A|G, 9534_C|T, 9866_C|T, 10029_C|T, 10198_C|T, 10447_G|A, 10449_C|A, 11288_TCTGGTTTT|........., 12444_A|G, 12880_C|T, 14408_C|T, 15451_G|A, 15714_C|T, 17410_C|T, 18163_A|G, 19955_C|T, 20055_A|G, 21618_C|T, 21633_TACCCCCTG|........., 21987_G|A, 22001_A|G, 22016_T|C, 22033_C|A, 22190_A|G, 22200_T|G, 22331_G|A, 22577_GG|CA, 22599_G|C, 22674_C|T, 22679_T|C, 22686_C|T, 22688_A|G, 22775_G|A, 22786_A|C, 22813_G|T, 22882_T|G, 22898_G|A, 22942_T|G, 22992_G|A, 22995_C|A, 23013_A|C, 23019_T|C, 23055_A|G, 23063_A|T, 23075_T|C, 23403_A|G, 23525_C|T, 23599_T|G, 23604_C|A, 23854_C|A, 23948_G|T, 24424_A|T, 24469_T|A, 24620_G|T, 25000_C|T, 25584_C|T, 26060_C|T, 26270_C|T, 26529_G|A, 26577_C|G, 26709_G|A, 27038_A|G, 27807_C|T, 27889_C|T, 28271_A|T, 28311_C|T, 28330_A|G, 28362_GAGAACGCA|........., 28881_GGG|AAC, 29510_A|C

## Case 11 (1BP mid): XBE          test: OK

Target: (75%) 63 samples
GT: BA.5.2* + BE.4.1
BC: BA.5.2.6 + BE.4
Direction L1: >>
Alt. candidates: [CP.3], [CQ.2, BE.4.1.1, BE.4.1, CQ.1.1, CQ.1]

Number of changes: 72
GT BR: 31–53
BC BR: 63–64
Initial region span: 1–63,64–72

GT BR coord: 22591 – 23609   Rank L1 L2: 11 4
BC BR coord: 26719 – 26720
Gap history (edge excluded):

Model 1BP/2BP comparison: –
Rec. model vs L1: 5.22e-26 Rec. model vs L2: 3.98e-39
Flags: Model_2BP_Bad_L1_opp

## BA.5.2.6 >>

| | num_seq | t_ch_MAX | max_CL | CL@BC_t_ch_MAX | aic | PV | PV_OK | t_ch_MAX_OK | phyl_OK |
|---|---|---|---|---|---|---|---|---|---|
| **BA.5.2.6** | 6167 | 63 | 61.024915 | NaN | NaN | NaN | * | * | * |
| **BA.5.2.12** | 1681 | 63 | 58.124022 | 58.124022 | 27.751955 | 0.055023 | * | * | |
| **CP.3** | 123 | 63 | 57.362413 | 57.362413 | 29.275174 | 0.025604 | * | * | * |
| **CP.1** | 412 | 60 | 54.514867 | 49.346273 | 45.307454 | 0.000008 | | | * |
| **BA.5.2.28** | 1617 | 63 | 50.958454 | 50.958454 | 42.083093 | 0.000043 | * | * | |
| **BA.5.2.20** | 8940 | 63 | 50.716992 | 50.716992 | 42.566017 | 0.000033 | * | * | |
| **BA.5.2.26** | 2393 | 58 | 50.364909 | 47.739468 | 48.521065 | 0.000002 | | | |
| **BA.5.2.2** | 2056 | 63 | 48.743511 | 48.743511 | 46.512978 | 0.000005 | | * | |
| **BA.5.2.16** | 828 | 63 | 48.627584 | 48.627584 | 46.744833 | 0.000004 | | * | |
| **BA.5.2.22** | 2146 | 63 | 48.577336 | 48.577336 | 46.845328 | 0.000004 | | * | |

## BE.4 <<

| | num_seq | t_ch_MAX | max_CL | CL@BC_t_ch_MAX | aic | PV | PV_OK | t_ch_MAX_OK | phyl_OK |
|---|---|---|---|---|---|---|---|---|---|
| **BE.4** | 1310 | 64 | 16.493377 | NaN | NaN | NaN | * | * | * |
| **CQ.2** | 790 | 64 | 16.489531 | 16.489531 | -12.979063 | 9.950125e-01 | * | * | * |
| **BE.4.1.1** | 122 | 64 | 16.464802 | 16.464802 | -12.929603 | 9.704455e-01 | * | * | * |
| **BE.4.1** | 354 | 64 | 16.345907 | 16.345907 | -12.691814 | 8.607080e-01 | * | * | * |
| **CQ.1.1** | 39 | 64 | 10.783524 | 10.783524 | -1.567049 | 3.312673e-03 | * | * | * |
| **CQ.1** | 284 | 64 | 10.662170 | 10.662170 | -1.324340 | 2.923423e-03 | * | * | * |
| **BE.1.4** | 780 | 64 | 10.038598 | 10.038598 | -0.077196 | 1.572639e-03 | * | * | |
| **BE.5** | 336 | 65 | 9.566480 | -0.433520 | 20.867041 | 4.440117e-08 | | * | |
| **BE.1.4.1** | 137 | 65 | 9.505979 | -0.494021 | 20.988042 | 4.181545e-08 | | * | |
| **BE.1.2** | 455 | 65 | 9.504659 | -0.495341 | 20.990681 | 4.181545e-08 | | * | |

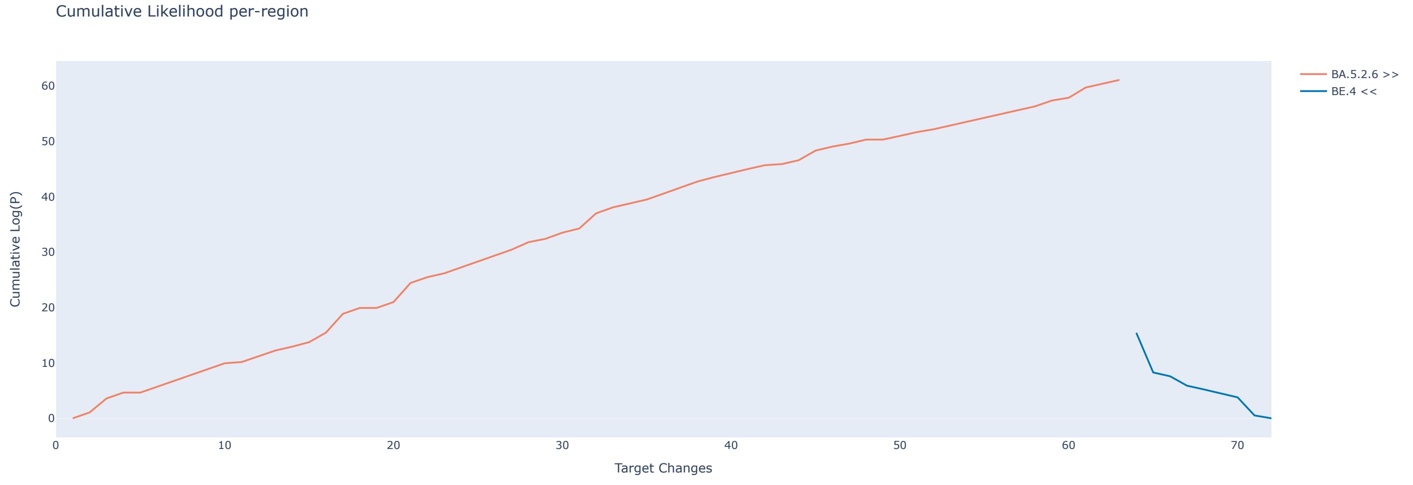


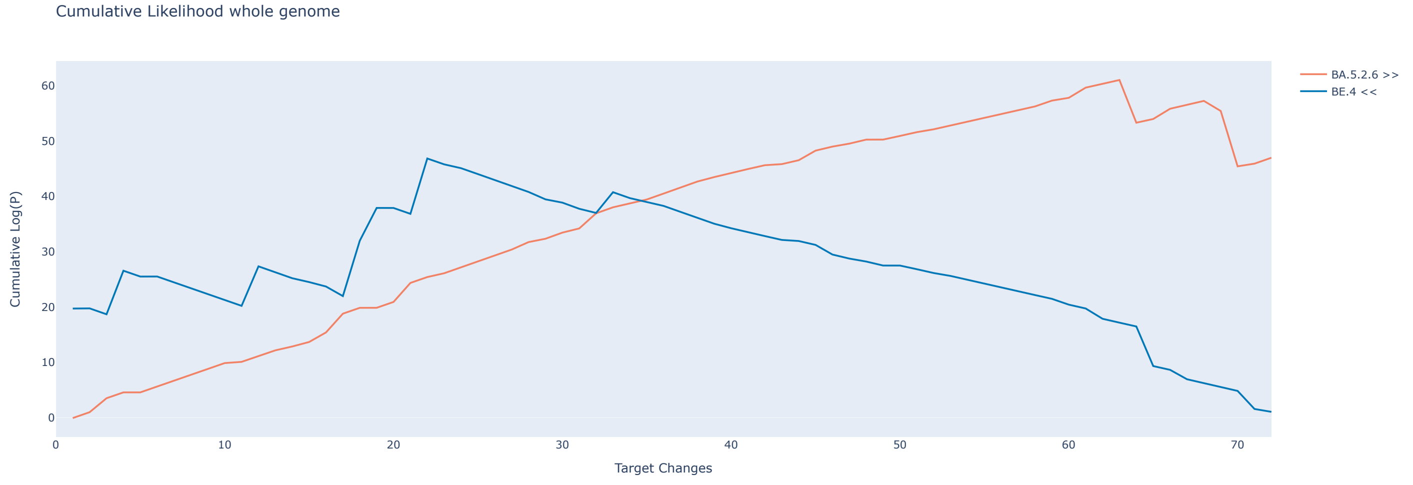


**Target sequence**

, 241_C|T, 670_T|G, 1627_C|T, 2790_C|T, 3037_C|T, 4184_G|A, 4321_C|T, 9344_C|T, 9424_A|G, 9534_C|T, 10029_C|T, 10198_C|T, 10447_G|A, 10449_C|A, 11288_TCTGGTTTT|........., 12160_G|A, 12310_G|A, 12880_C|T, 14408_C|T, 15714_C|T, 16616_C|A, 17410_C|T, 18163_A|G, 19955_C|T, 20055_A|G, 21618_C|T, 21633_TACCCCCTG|........., 21765_TACATG|......, 21987_G|A, 22200_T|G, 22578_G|A, 22599_G|C, 22674_C|T, 22679_T|C, 22686_C|T, 22688_A|G, 22775_G|A, 22786_A|C, 22813_G|T, 22882_T|G, 22917_T|G, 22992_G|A, 22995_C|A, 23013_A|C, 23018_T|G, 23055_A|G, 23063_A|T, 23075_T|C, 23403_A|G, 23525_C|T, 23599_T|G, 23604_C|A, 23854_C|A, 23948_G|T, 24424_A|T, 24469_T|A, 25000_C|T, 25584_C|T, 26060_C|T, 26270_C|T, 26529_G|A, 26577_C|G, 26709_G|A, 27800_C|T, 27807_C|T, 27889_C|T, 28271_A|T, 28311_C|T, 28362_GAGAACGCA|........., 28681_G|T, 28881_GGG|AAC, 29510_A|C

## Case 12 (1BP mid): XBF test: KO

Target: (75%) 4944 samples
Number of changes: 84
GT: BA.5.2 + CJ.1
GT BR: 14-16
GT BR coord: 9864 - 9867
Rank L1 L2: 11 -
BC: BM.1.1.1
BC BR:
BC BR coord:
Direction L1: <<
Initial region span: 2-84
Gap history (edge excluded):

Alt. candidates: []
Model 1BP/2BP comparison: –
Rec. model vs L1: – Rec. model vs L2: –
Flags: NotEnoughSpaceAfterL1, SingleCandidateGenome

## BM.1.1.1 <<

| | num_seq | t_ch_MAX | max_CL | CL@BC_t_ch_MAX | aic | PV | PV_OK | t_ch_MAX_OK | phyl_OK |
|---|---|---|---|---|---|---|---|---|---|
| **BM.1.1.1** | 41 | 2 | 123.443348 | NaN | NaN | NaN | * | * | * |
| **CJ.1** | 538 | 12 | 106.286982 | 48.983244 | 106.033513 | 6.361779e-33 | | | * |
| **CJ.1.1** | 72 | 12 | 98.780518 | 16.022225 | 171.955550 | 3.069453e-47 | | | * |
| **BM.2** | 118 | 12 | 86.021334 | 32.248314 | 139.503372 | 3.432894e-40 | | | |
| **BM.4.1.1** | 570 | 12 | 80.304864 | 18.302356 | 167.395289 | 3.000906e-46 | | | |
| **CV.2** | 64 | 12 | 79.931899 | 3.576559 | 196.846882 | 1.208554e-52 | | | |
| **BA.2.75** | 1935 | 12 | 75.930787 | 18.319386 | 167.361229 | 3.061528e-46 | | | * |
| **BN.5** | 231 | 12 | 75.224925 | 2.253227 | 199.493547 | 3.228475e-53 | | | |
| **CB.1** | 240 | 12 | 73.942745 | 10.566390 | 182.867221 | 1.312153e-49 | | | |
| **BA.2.75.3** | 384 | 12 | 72.608293 | 9.330394 | 185.339213 | 3.816198e-50 | | | * |

Cumulative Likelihood per-region

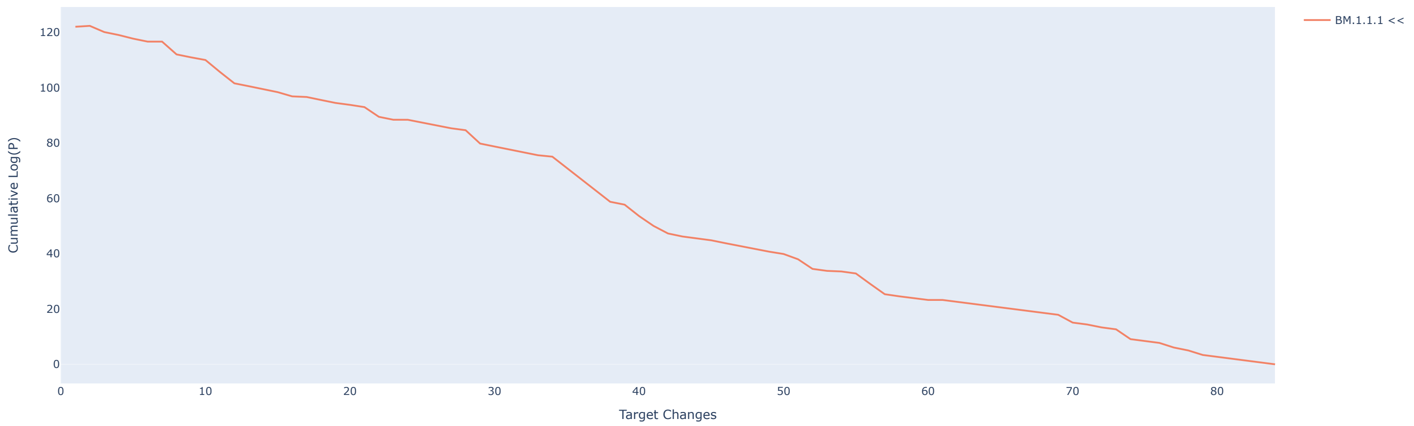


Cumulative Likelihood whole genome

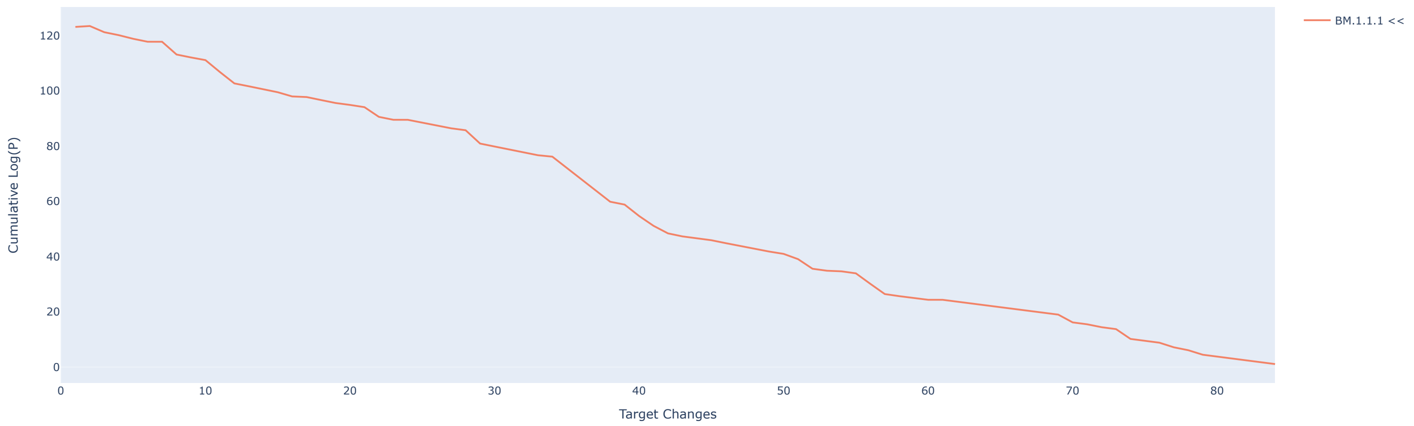


**Target sequence**

, 241_C|T, 625_G|T, 670_T|G, 1627_C|T, 2790_C|T, 3037_C|T, 3339_T|C, 4184_G|A, 4321_C|T, 6070_C|T, 8692_C|T, 9344_C|T, 9424_A|G, 9534_C|T, 9866_C|T, 10029_C|T, 10198_C|T, 10447_G|A, 10449_C|A, 11288_TCTGGTTTT|........., 12444_A|G, 12880_C|T, 14408_C|T, 15451_G|A, 15714_C|T, 17410_C|T, 18163_A|G, 18583_G|A, 19955_C|T, 20055_A|G, 21618_C|T, 21633_TACCCCCTG|........., 21987_G|A, 22001_A|G, 22016_T|C, 22033_C|A, 22190_A|G, 22200_T|G, 22331_G|A, 22577_GG|CA, 22599_G|C, 22674_C|T, 22679_T|C, 22686_C|T, 22688_A|G, 22775_G|A, 22786_A|C, 22813_G|T, 22882_T|G, 22898_G|A, 22942_T|G, 22992_G|A, 22995_C|A, 23013_A|C, 23018_TT|CC, 23031_T|C, 23055_A|G, 23063_A|T, 23075_T|C, 23403_A|G, 23525_C|T, 23599_T|G, 23604_C|A, 23854_C|A, 23948_G|T, 24424_A|T, 24469_T|A, 25000_C|T, 25416_C|T, 25584_C|T, 26060_C|T, 26270_C|T, 26275_A|G, 26577_C|G, 26709_G|A, 26858_C|T, 27259_A|C, 27382_GAT|CTC, 27807_C|T, 28271_A|T, 28311_C|T, 28362_GAGAACGCA|........., 28881_GGG|AAC, 29510_A|C

## Case 13 (1BP mid): XBG test: OK

Target: (75%) 86 samples Number of changes: 75
GT: BA.2.76 + BA.5.2 GT BR: 32–41 GT BR coord: 22600 – 22916 Rank L1 L2: 1 1
BC: BA.2.76 + BA.5.2 BC BR: 40–41 BC BR coord: 22891 – 22892
Direction L1: >> Initial region span: 1–40,41–75 Gap history (edge excluded):
Alt. candidates: [], [BA.5.2.21]
Model 1BP/2BP comparison: 1BP vs 2BP: 1.75e-03
Rec. model vs L1: 2.27e-64 Rec. model vs L2: 3.95e-50
Flags: Model_1BP_Best

## BA.2.76 >>

| | num_seq | t_ch_MAX | max_CL | CL@BC_t_ch_MAX | aic | PV | PV_OK | t_ch_MAX_OK | phyl_OK |
|---|---|---|---|---|---|---|---|---|---|
| **BA.2.76** | 3279 | 40 | 51.003467 | NaN | NaN | NaN | * | * | * |
| **BA.5.2** | 108274 | 75 | 28.642429 | -6.495798 | 114.991596 | 1.066761e-25 | | | |
| **BA.5.1.10** | 6066 | 56 | 27.425381 | 17.047073 | 67.905853 | 1.783854e-15 | | | |
| **BA.5.2.6** | 6167 | 75 | 24.645228 | 1.164380 | 99.671239 | 2.263409e-22 | | | |
| **BA.2** | 378689 | 40 | 22.800947 | 22.800947 | 56.398106 | 5.632797e-13 | | * | * |
| **BA.5.2.1** | 93917 | 56 | 19.125981 | 8.616527 | 84.766946 | 3.892754e-19 | | | |
| **BA.2.9** | 108830 | 38 | 19.002080 | 17.420117 | 67.159766 | 2.595493e-15 | | | |
| **BA.2.5** | 1908 | 29 | 18.516257 | 11.444808 | 79.110383 | 6.596450e-18 | | | |
| **BA.2.12.2** | 360 | 16 | 16.992829 | -24.657803 | 151.315606 | 1.377552e-33 | | | |
| **BA.2.12** | 2844 | 22 | 16.912483 | 6.017221 | 89.965558 | 2.891287e-20 | | | |

## BA.5.2 <<

| | num_seq | t_ch_MAX | max_CL | CL@BC_t_ch_MAX | aic | PV | PV_OK | t_ch_MAX_OK | phyl_OK |
|---|---|---|---|---|---|---|---|---|---|
| **BA.5.2** | 108274 | 41 | 35.138227 | NaN | NaN | NaN | * | * | * |
| **BA.5.2.21** | 3715 | 41 | 24.071980 | 24.071980 | 33.856040 | 1.557256e-05 | * | * | * |
| **BA.5.2.6** | 6167 | 41 | 23.480847 | 23.480847 | 35.038305 | 8.632296e-06 | | * | * |
| **CP.2** | 30 | 62 | 19.762803 | 7.052649 | 67.894702 | 6.350961e-13 | | | * |
| **BA.5.2.43** | 575 | 62 | 19.746922 | 1.646506 | 78.706989 | 2.839921e-15 | | | * |
| **BA.5.2.49** | 341 | 62 | 19.733501 | 6.182132 | 69.635736 | 2.647475e-13 | | | * |
| **BA.5.2.48** | 2644 | 62 | 19.725105 | 12.920591 | 56.158819 | 2.238601e-10 | | | * |
| **CR.1.1** | 335 | 62 | 19.715468 | 6.403740 | 69.192521 | 3.315493e-13 | | | * |
| **CN.2** | 184 | 62 | 19.710146 | 12.214389 | 57.571223 | 1.106112e-10 | | | * |
| **BU.1** | 393 | 62 | 19.707767 | 2.114274 | 77.771451 | 4.543858e-15 | | | * |

Cumulative Likelihood per-region

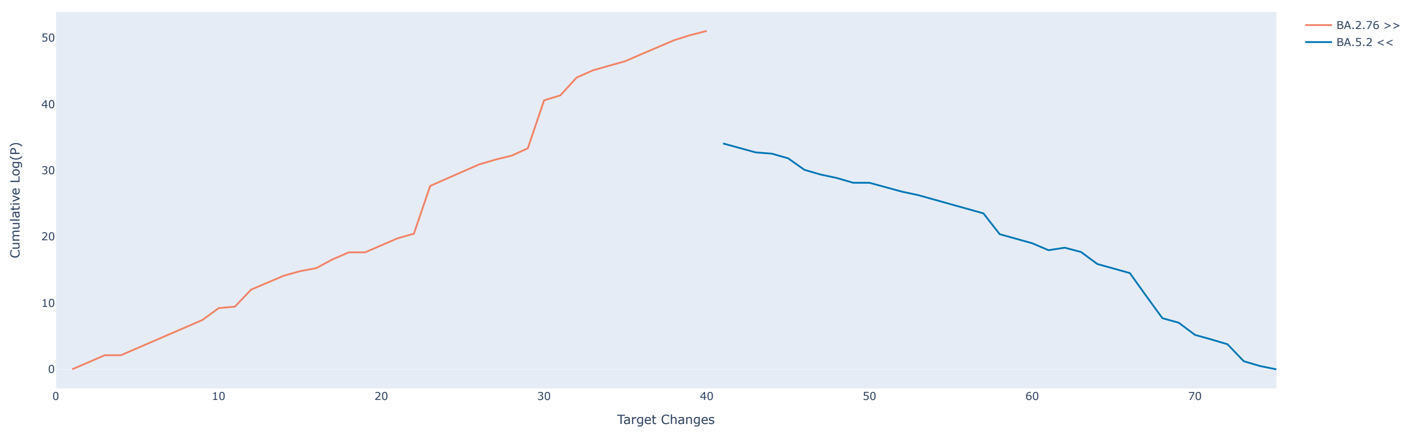


Cumulative Likelihood whole genome

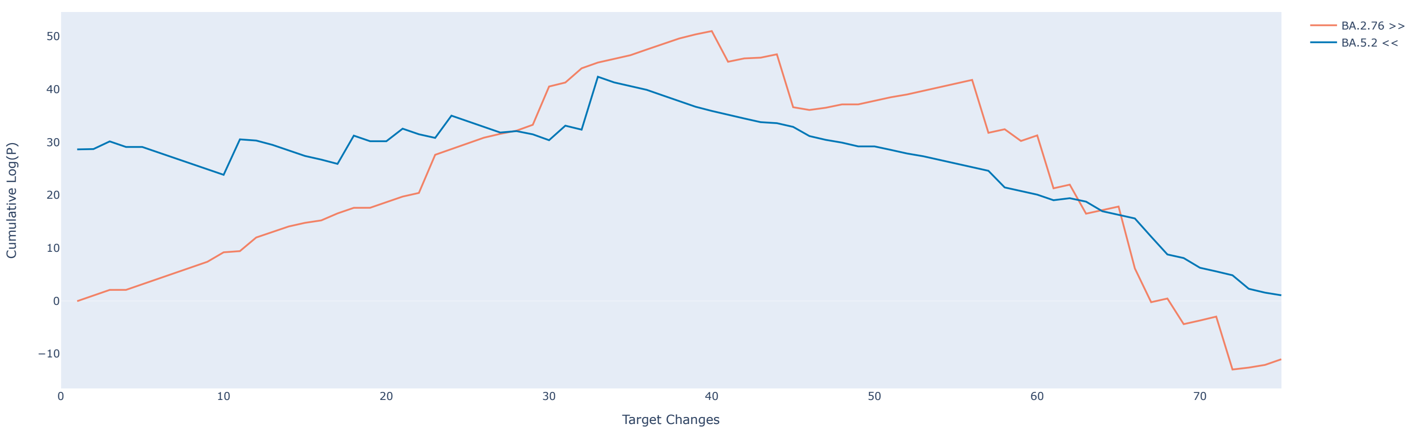


**Target sequence**

, 241_C|T, 670_T|G, 2790_C|T, 3037_C|T, 4184_G|A, 4321_C|T, 9344_C|T, 9424_A|G, 9534_C|T, 9866_C|T, 10029_C|T, 10116_C|T, 10198_C|T, 10447_G|A, 10449_C|A, 11288_TCTGGTTTT|........., 11665_C|T, 12880_C|T, 14408_C|T, 15714_C|T, 17410_C|T, 18163_A|G, 19812_A|G, 19955_C|T, 20055_A|G, 21618_C|T, 21633_TACCCCCTG|........., 21987_G|A, 22200_T|G, 22304_T|A, 22578_G|A, 22599_G|C, 22674_C|T, 22679_T|C, 22686_C|T, 22688_A|G, 22775_G|A, 22786_A|C, 22813_G|T, 22882_T|G, 22917_T|G, 22992_G|A, 22995_C|A, 23013_A|C, 23018_T|G, 23055_A|G, 23063_A|T, 23075_T|C, 23403_A|G, 23525_C|T, 23599_T|G, 23604_C|A, 23854_C|A, 23948_G|T, 24424_A|T, 24469_T|A, 24676_A|G, 25000_C|T, 25584_C|T, 26060_C|T, 26180_T|C, 26270_C|T, 26529_G|A, 26577_C|G, 26709_G|A, 27012_C|T, 27513_C|T, 27807_C|T, 27889_C|T, 28271_A|T, 28311_C|T, 28330_A|G, 28362_GAGAACGCA|........., 28881_GGG|AAC, 29510_A|C

## Case 14 (1BP mid): XBH    test: KO

Target: (75%) 71 samples
Number of changes: 78
GT: BA.2.3.17 + BA.2.75.2
GT BR: 19–28
GT BR coord: 15450 – 22001
Rank L1 L2: 11 11 –
BC: BA.2.75.2 + BA.2.3.17 + BA.2.75.2
BC BR: 4–5, 19–20
BC BR coord: 3039 – 3040, 15713 – 15714
Direction L1: <<
Initial region span: 1–4,8–19,20–78
Gap history (edge excluded): 4–20 -> 4–8
Alt. candidates: [], [CA.6, CA.2]
Model 1BP/2BP comparison: 2BP vs 1BP: 3.65e–02
Rec. model vs L1: 4.34e–55
Rec. model vs L2: 3.88e–142
Flags: Model_2BP_Best

## BA.2.75.2 >>

| | num_seq | t_ch_MAX | max_CL | CL@BC_t_ch_MAX | aic | PV | PV_OK | t_ch_MAX_OK | phyl_OK |
|---|---|---|---|---|---|---|---|---|---|
| **BA.2.75.2** | 1428 | 4 | 2.113939 | None | None | None | * | * | * |

## BA.2.3.17 <<

| | num_seq | t_ch_MAX | max_CL | CL@BC_t_ch_MAX | aic | PV | PV_OK | t_ch_MAX_OK | phyl_OK |
|---|---|---|---|---|---|---|---|---|---|
| **BA.2.3.17** | 845 | 8 | 16.685755 | NaN | NaN | NaN | * | * | * |
| **BA.2.3.18** | 48 | 8 | 12.833009 | 0.446017 | 41.107967 | 0.021174 | * | * | |
| **BA.2.25** | 331 | 8 | 12.427574 | 4.550567 | 32.898865 | 1.284025 | * | * | |
| **BA.2.9.5** | 1119 | 8 | 11.067047 | 4.609301 | 32.781398 | 1.363425 | * | * | |
| **BA.2.21** | 872 | 8 | 10.115892 | -5.894690 | 53.789380 | 0.000037 | * | * | |
| **BA.2.40.1** | 1414 | 8 | 10.083599 | 10.060878 | 21.878244 | 317.348329 | * | * | |
| **BA.2.13** | 2436 | 8 | 9.902437 | 2.021175 | 37.957649 | 0.102284 | * | * | |
| **BA.2.36** | 5393 | 8 | 9.672561 | 9.565883 | 22.868233 | 193.446309 | * | * | |
| **BA.2.24** | 1290 | 5 | 9.606670 | 9.606670 | 22.786659 | 201.341002 | * | | |
| **XBB.3** | 323 | 8 | 9.597366 | 1.710877 | 38.578246 | 0.075020 | * | * | |

## BA.2.75.2 <<

| | num_seq | t_ch_MAX | max_CL | CL@BC_t_ch_MAX | aic | PV | PV_OK | t_ch_MAX_OK | phyl_OK |
|---|---|---|---|---|---|---|---|---|---|
| **BA.2.75.2** | 1428 | 20 | 90.038404 | NaN | NaN | NaN | * | * | * |
| **CA.6** | 22 | 20 | 89.494911 | 91.436847 | -38.873693 | 4.891921e-01 | * | * | * |
| **CA.7** | 390 | 22 | 86.489176 | 81.958129 | -19.916257 | 3.754389e-05 | * | | * |
| **CA.2** | 43 | 20 | 82.344732 | 84.420882 | -24.841765 | 4.394441e-04 | * | * | * |
| **CA.3** | 300 | 20 | 76.656992 | 78.771002 | -13.542005 | 1.545752e-06 | | * | * |
| **BM.1.1.3** | 1433 | 23 | 73.023581 | 72.451912 | -0.903824 | 2.782266e-09 | | | |
| **CA.1** | 120 | 20 | 72.697457 | 74.845929 | -5.691857 | 3.051645e-08 | | * | * |
| **BY.1** | 683 | 20 | 71.668123 | 73.746863 | -3.493727 | 1.015804e-08 | | * | |
| **BM.1.1.1** | 41 | 2 | 69.657790 | 67.184318 | 9.631364 | 1.438263e-11 | | | |
| **BM.4.1.1** | 570 | 23 | 68.918262 | 68.341447 | 7.317107 | 4.565080e-11 | | | |

Cumulative Likelihood per-region

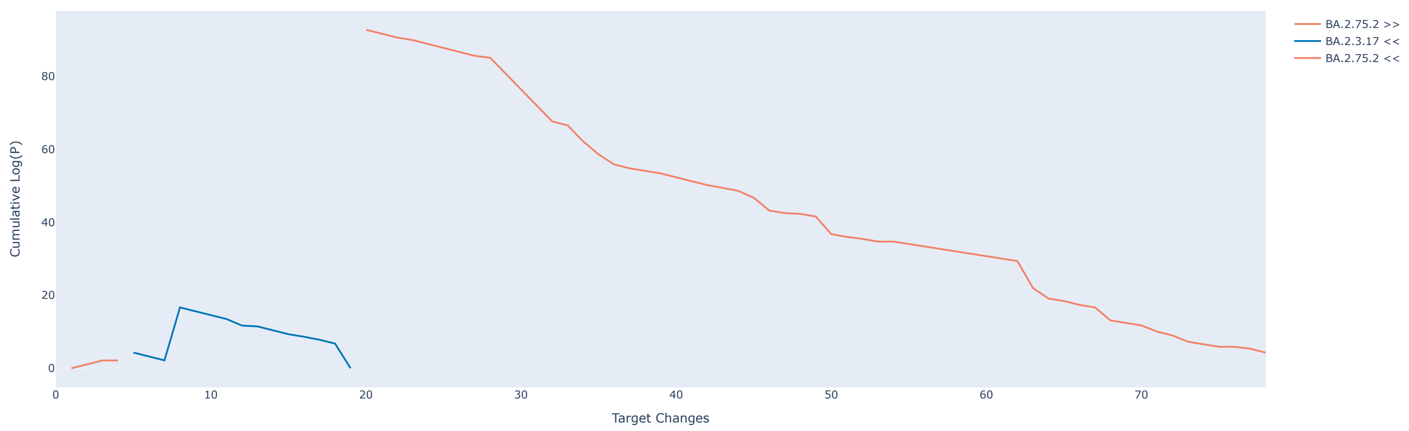


Cumulative Likelihood whole genome

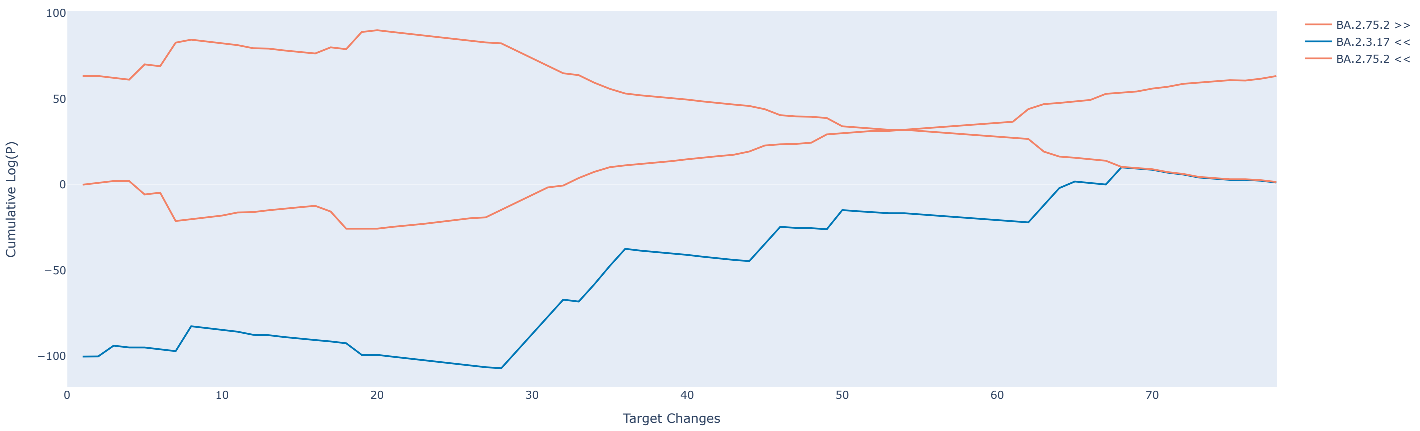


**Target sequence**

, 241_C|T, 670_T|G, 2790_C|T, 3037_C|T, 4184_G|A, 4321_C|T, 7528_C|T, 9344_C|T, 9424_A|G, 9534_C|T, 9866_C|T, 10029_C|T, 10198_C|T, 10447_G|A, 10449_C|A, 11288_TCTGGTTTT|........., 12880_C|T, 13730_C|T, 14408_C|T, 15714_C|T, 17410_C|T, 18163_A|G, 19955_C|T, 20055_A|G, 21618_C|T, 21633_TACCCCCTG|........., 21987_G|A, 22001_A|G, 22016_T|C, 22033_C|A, 22190_A|G, 22200_T|G, 22331_G|A, 22577_GG|CA, 22599_G|C, 22674_C|T, 22679_T|C, 22686_C|T, 22688_A|G, 22775_G|A, 22786_A|C, 22813_G|T, 22882_T|G, 22898_G|A, 22942_T|G, 22992_G|A, 22995_C|A, 23013_A|C, 23019_T|C, 23055_A|G, 23063_A|T, 23075_T|C, 23403_A|G, 23525_C|T, 23599_T|G, 23604_C|A, 23854_C|A, 23948_G|T, 24424_A|T, 24469_T|A, 25000_C|T, 25157_G|A, 25416_C|T, 25584_C|T, 26060_C|T, 26270_C|T, 26275_A|G, 26577_C|G, 26709_G|A, 26858_C|T, 27259_A|C, 27382_GAT|CTC, 27807_C|T, 28271_A|T, 28311_C|T, 28881_GGG|AAC, 29510_A|C, 29734_GAGGCCACGCGGAGTACGATCGAGTG|.........................

## Case 15 (1BP mid): XBJ        test: OK

Target: (75%) 120 samples        Number of changes: 88

GT: BA.2.3.20 + BA.5.2* GT BR: 59–73 GT BR coord: 23014 – 25810 Rank_L1_L2: 1 4
BC: BA.2.3.20 + BA.5.2.36 BC BR: 72–73 BC BR coord: 25584 – 25585
Direction L1: >> Initial region span: 1–72,73–88 Gap history (edge excluded):
Alt. candidates: [CM.2, CM.5, CM.11], [BA.5.2]
Model 1BP/2BP comparison: –
Rec. model vs L1: 8.54e–89 Rec. model vs L2: 1.20e–254
Flags: Model_2BP_Bad_L1_opp

## BA.2.3.20 >>

| | num_seq | t_ch_MAX | max_CL | CL@BC_t_ch_MAX | aic | PV | PV_OK | t_ch_MAX_OK | phyl_OK |
|---|---|---|---|---|---|---|---|---|---|
| **BA.2.3.20** | 941 | 72 | 154.015753 | NaN | NaN | NaN | * | * | * |
| **CM.2** | 1505 | 71 | 153.585959 | 146.813183 | -131.626367 | 7.465858e-04 | * | * | * |
| **CM.5** | 141 | 72 | 147.707065 | 147.707065 | -133.414129 | 1.818033e-03 | * | * | * |
| **CM.11** | 76 | 72 | 144.419689 | 144.419689 | -126.839378 | 6.806823e-05 | * | * | * |
| **CM.7** | 37 | 59 | 135.642362 | 125.008751 | -88.017501 | 2.530979e-13 | | | * |
| **CM.4** | 270 | 72 | 135.260336 | 135.260336 | -108.520672 | 7.158252e-09 | | * | * |
| **CM.6** | 15 | 72 | 133.344612 | 133.344612 | -104.689223 | 1.054710e-09 | | * | * |
| **CM.8** | 34 | 72 | 133.250496 | 133.250496 | -104.500993 | 9.591247e-10 | | * | * |
| **CM.10** | 104 | 72 | 131.714401 | 131.714401 | -101.428801 | 2.066489e-10 | | * | * |
| **CM.9** | 24 | 71 | 129.027019 | 122.256239 | -82.512477 | 1.609931e-14 | | * | * |

## BA.5.2.36 <<

| | num_seq | t_ch_MAX | max_CL | CL@BC_t_ch_MAX | aic | PV | PV_OK | t_ch_MAX_OK | phyl_OK |
|---|---|---|---|---|---|---|---|---|---|
| **BA.5.2.36** | 306 | 73 | 25.611731 | NaN | NaN | NaN | * | * | * |
| **BA.5.2.24** | 347 | 73 | 24.961687 | 24.961687 | -17.923374 | 5.220458e-01 | * | * | |
| **BA.5.2.38** | 49 | 73 | 24.831427 | 24.831427 | -17.662853 | 4.584060e-01 | * | * | |
| **BA.5.2** | 108274 | 73 | 24.193566 | 24.193566 | -16.387131 | 2.429256e-01 | * | * | * |
| **BA.5.2.6** | 6167 | 73 | 23.183246 | 23.183246 | -14.366491 | 8.847812e-02 | * | * | |
| **BA.5.2.18** | 1359 | 73 | 23.107844 | 23.107844 | -14.215687 | 8.208500e-02 | * | * | |
| **BA.5.2.31** | 513 | 73 | 22.182515 | 22.182515 | -12.365030 | 3.254928e-02 | * | * | |
| **BA.5.2.16** | 828 | 73 | 21.984959 | 21.984959 | -11.969919 | 2.664910e-02 | * | * | |
| **CP.2** | 30 | 74 | 20.814695 | 10.814695 | 10.370610 | 3.755028e-07 | | * | |
| **BA.5.2.43** | 575 | 74 | 20.798813 | 10.798813 | 10.402373 | 3.699123e-07 | | * | |

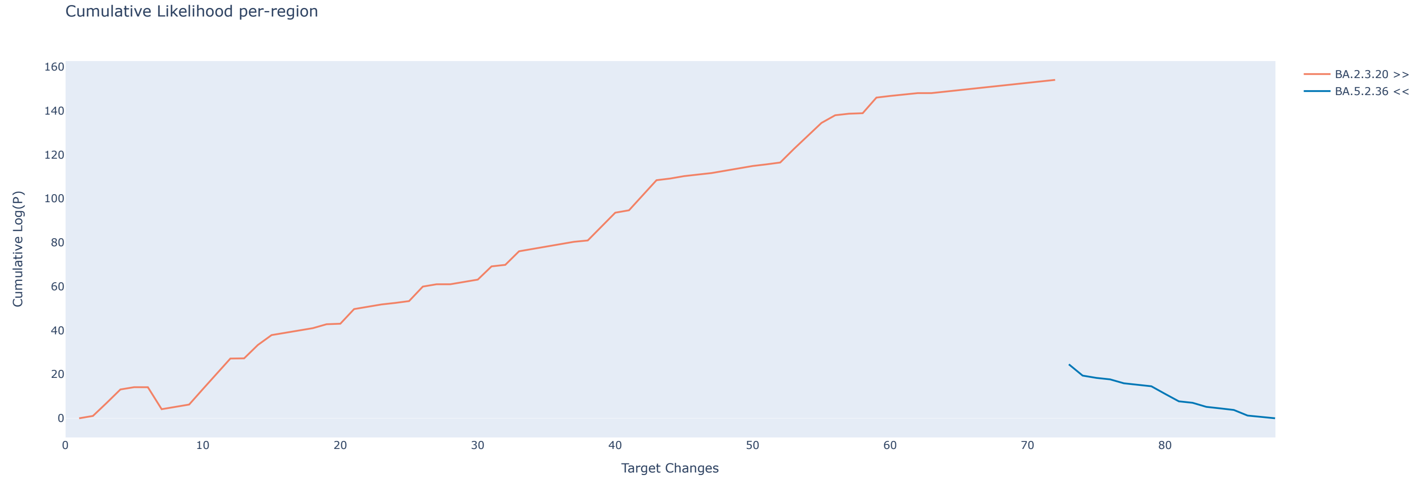


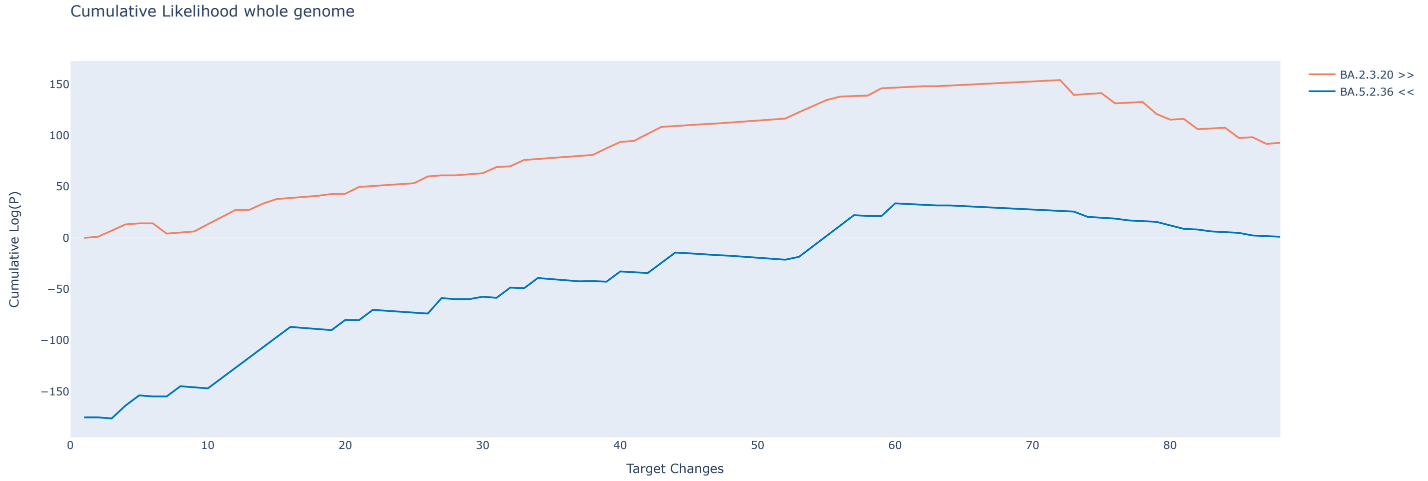


**Target sequence**

, 241_C|T, 670_T|G, 1471_C|T, 2445_C|T, 2790_C|T, 3037_C|T, 3072_G|A, 4184_G|A, 4321_C|T, 5406_T|C, 6770_A|G, 6786_C|T, 8016_C|T, 8208_C|T, 8991_C|T, 9344_C|T, 9424_A|G, 9534_C|T, 9866_C|T, 10029_C|T, 10189_C|T, 10198_C|T, 10447_G|A, 10449_C|A, 11288_TCTGGTTTT|........., 12030_A|G, 12880_C|T, 14408_C|T, 15714_C|T, 17410_C|T, 17678_C|T, 18163_A|G, 18252_C|T, 19955_C|T, 20055_A|G, 21618_C|T, 21633_TACCCCCTG|........., 21987_G|A, 22020_T|C, 22054_T|G, 22200_T|G, 22295_C|A, 22332_G|A, 22578_G|A, 22674_C|T, 22679_T|C, 22686_C|T, 22688_A|G, 22775_G|A, 22786_A|C, 22813_G|T, 22882_T|G, 22893_A|G, 22910_A|G, 22916_C|A, 22942_T|G, 22992_G|A, 22995_C|A, 23012_GA|AG, 23055_A|G, 23063_A|T, 23075_T|C, 23403_A|G, 23525_C|T, 23599_T|G, 23604_C|A, 23854_C|A, 23948_G|T, 24424_A|T, 24469_T|A, 25000_C|T, 25584_C|T, 25896_C|T, 26060_C|T, 26270_C|T, 26529_G|A, 26577_C|G, 26709_G|A, 27012_C|T, 27513_C|T, 27807_C|T, 27889_C|T, 28271_A|T, 28311_C|T, 28330_A|G, 28362_GAGAACGCA|........., 28881_GGG|AAC, 29510_A|C

**Case 16 (1BP mid): XBM** **test: KO**

Target: (75%) 205 samples Number of changes: 77
GT: BA.2.76 + BF.3 GT BR: 33–42 GT BR coord: 22600 – 22916 Rank L1 L2: 11 11 –
BC: BF.3 + BA.2.76 + BF.3 BC BR: 6–7, 33–34 BC BR coord: 4330 – 4331, 22673 – 22674
Direction L1: << Initial region span: 1–6,25–33,34–77 Gap history (edge excluded): 6–34 -> 6–25
Alt. candidates: [], [BF.3.1]
Model 1BP/2BP comparison: 2BP vs 1BP: 2.47e−11
Rec. model vs L1: 9.42e−38 Rec. model vs L2: 8.83e−96
Flags: Model_2BP_Best

## BF.3 >>

| | num_seq | t_ch_MAX | max_CL | CL@BC_t_ch_MAX | aic | PV | PV_OK | t_ch_MAX_OK | phyl_OK |
|---|---|---|---|---|---|---|---|---|---|
| **BF.3** | 929 | 6 | 1.737356 | None | None | None | * | * | * |

## BA.2.76 <<

| | num_seq | t_ch_MAX | max_CL | CL@BC_t_ch_MAX | aic | PV | PV_OK | t_ch_MAX_OK | phyl_OK |
|---|---|---|---|---|---|---|---|---|---|
| **BA.2.76** | 3279 | 25 | 16.346946 | NaN | NaN | NaN | * | * | * |
| **BA.5.1.20** | 205 | 25 | 13.211996 | -27.554379 | 143.108758 | 3.721463e-19 | | * | |
| **BA.2.76.2** | 17 | 30 | 11.852659 | -44.048532 | 176.097064 | 2.552855e-26 | | | * |
| **BA.5.2.6** | 6167 | 25 | 9.414638 | -20.557341 | 129.114682 | 4.081079e-16 | | * | |
| **BA.2.76.1** | 42 | 25 | 8.594817 | -45.065766 | 178.131533 | 9.251609e-27 | | * | * |
| **BA.2** | 378689 | 7 | 7.791043 | 7.791043 | 72.417913 | 8.333974e-04 | * | | * |
| **BQ.1.1.4** | 4564 | 25 | 7.013627 | -36.753906 | 161.507812 | 3.760144e-23 | | * | |
| **BA.5.1.10** | 6066 | 25 | 6.282065 | -13.464115 | 114.928231 | 4.896919e-13 | | * | |
| **BA.2.9** | 108830 | 7 | 4.559194 | 4.559194 | 78.881613 | 3.296712e-05 | * | | |
| **BA.5.2.19** | 671 | 25 | 4.466805 | -52.135655 | 192.271311 | 7.866025e-30 | | * | |

## BF.3 <<

| | num_seq | t_ch_MAX | max_CL | CL@BC_t_ch_MAX | aic | PV | PV_OK | t_ch_MAX_OK | phyl_OK |
|---|---|---|---|---|---|---|---|---|---|
| **BF.3** | 929 | 34 | 53.787254 | NaN | NaN | NaN | * | * | * |
| **BF.3.1** | 99 | 34 | 52.391756 | 54.080944 | 3.838112 | 2.357461e-01 | * | * | * |
| **BA.5.2.1** | 93917 | 3 | 37.606263 | 36.850404 | 38.299193 | 7.754442e-09 | | | * |
| **BF.28** | 4033 | 34 | 36.203548 | 37.866232 | 36.267536 | 2.139732e-08 | | * | |
| **BF.5** | 32753 | 34 | 32.388255 | 34.018168 | 43.963663 | 4.576117e-10 | | * | |
| **BA.5** | 16505 | 24 | 26.064011 | 27.293693 | 57.412614 | 5.493723e-13 | | | * |
| **BF.7** | 34088 | 32 | 22.095734 | 20.266816 | 71.466369 | 4.861570e-16 | | | |
| **BF.31** | 266 | 61 | 20.811625 | 3.334492 | 105.331016 | 2.158594e-23 | | | * |
| **BF.23** | 175 | 34 | 20.713057 | 22.370523 | 67.258954 | 3.989941e-15 | | * | |
| **BF.26** | 4833 | 32 | 20.615808 | 20.791003 | 70.417993 | 8.218284e-16 | | | |

Cumulative Likelihood per-region

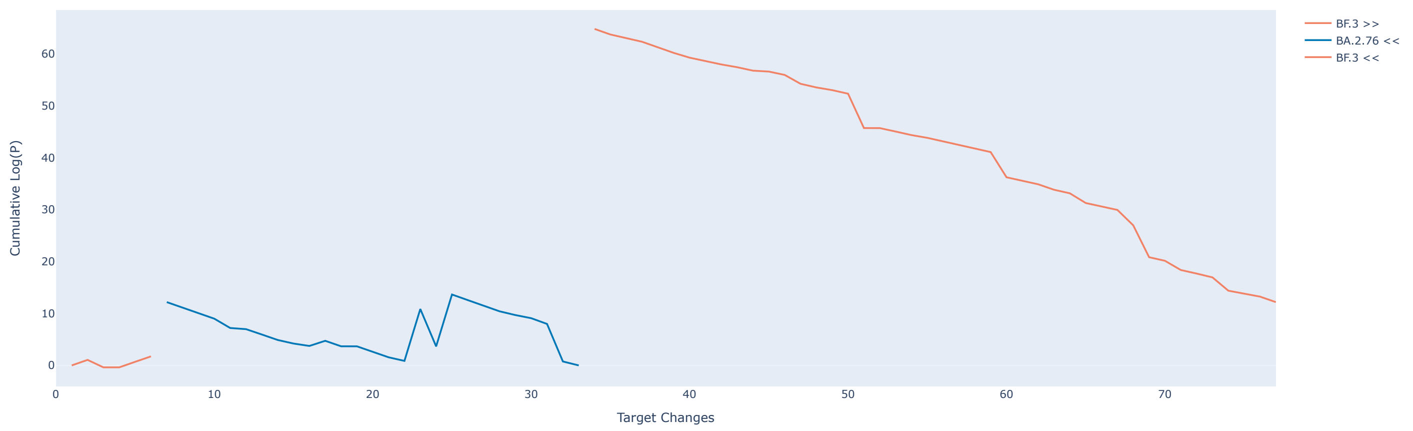


Cumulative Likelihood whole genome

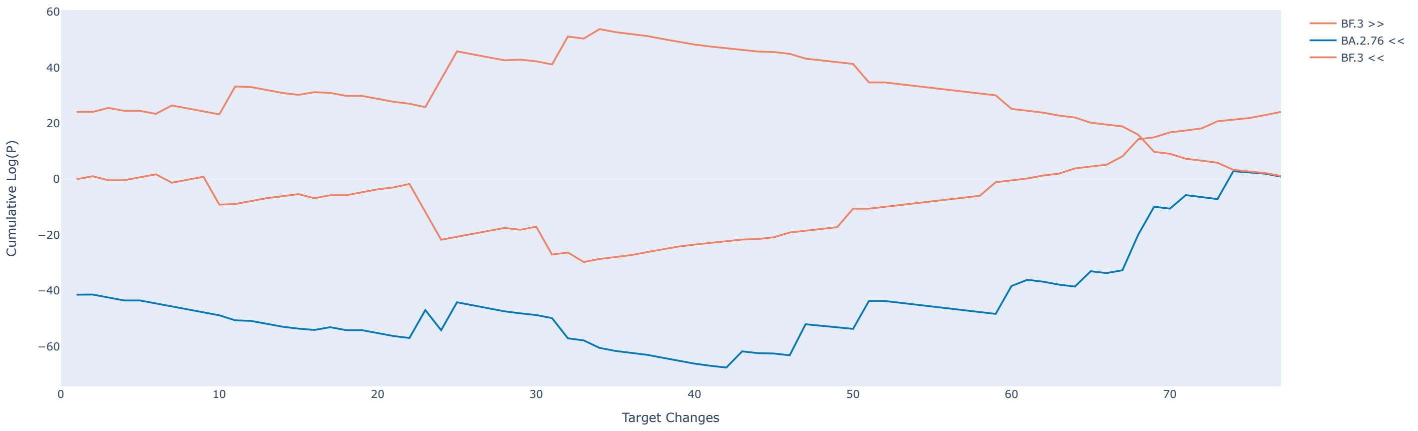


**Target sequence**

, 241_C|T, 670_T|G, 2790_C|T, 3037_C|T, 4184_G|A, 4321_C|T, 9344_C|T, 9424_A|G, 9534_C|T, 9866_C|T, 10029_C|T, 10198_C|T, 10447_G|A, 10449_C|A, 11288_TCTGGTTTT|........., 12534_C|T, 12880_C|T, 14408_C|T, 15714_C|T, 17410_C|T, 18163_A|G, 18429_T|C, 19812_A|G, 19891_G|A, 19955_C|T, 20055_A|G, 21618_C|T, 21633_TACCCCCTG|........., 21987_G|A, 22200_T|G, 22304_T|A, 22578_G|A, 22599_G|C, 22674_C|T, 22679_T|C, 22686_C|T, 22688_A|G, 22775_G|A, 22786_A|C, 22813_G|T, 22882_T|G, 22917_T|G, 22992_G|A, 22995_C|A, 23013_A|C, 23018_T|G, 23055_A|G, 23063_A|T, 23075_T|C,

23202_C|T, 23403_A|G, 23525_C|T, 23599_T|G, 23604_C|A, 23854_C|A, 23948_G|T, 24424_A|T, 24469_T|A, 24620_G|T, 25000_C|T, 25584_C|T, 26060_C|T, 26270_C|T, 26529_G|A, 26577_C|G, 26709_G|A, 27038_A|G, 27549_C|T, 27807_C|T, 27889_C|T, 28271_A|T, 28311_C|T, 28330_A|G, 28362_GAGAACGCA|........., 28881_GGG|AAC, 29510_A|C, 29734_GAGGCCACGCGGAGTACGATCGAGTG|..........................

## Case 17 (1BP mid): XJ                    test: OK

Target: (75%) 47 samples
Number of changes: 60
GT: BA.1* + BA.2*
GT BR: 13–16
GT BR coord: 13199 – 17401
Rank L1 L2: 11 11
BC: BA.1.17.2 + BA.2.65
BC BR: 13–14
BC BR coord: 13196 – 13197
Direction L1: <<
Initial region span: 1–13,14–60
Gap history (edge excluded):
Alt. candidates: [BD.1, BA.1.17, B.1.1.529], []
Model 1BP/2BP comparison: –
Rec. model vs L1: 2.52e−159
Rec. model vs L2: 2.59e−123
Flags: Model_2BP_Bad_L1_opp

### BA.1.17.2 >>

| | num_seq | t_ch_MAX | max_CL | CL@BC_t_ch_MAX | aic | PV | PV_OK | t_ch_MAX_OK | phyl_OK |
|---|---|---|---|---|---|---|---|---|---|
| **BA.1.17.2** | 77688 | 13 | 21.938143 | NaN | NaN | NaN | * | * | * |
| **BD.1** | 2033 | 13 | 21.788590 | 21.788590 | -11.577180 | 8.607080e-01 | * | * | * |
| **BA.1.17** | 27172 | 13 | 17.792887 | 17.792887 | -3.585775 | 1.584344e-02 | * | * | * |
| **B.1.1.529** | 1110 | 13 | 14.660121 | 14.660121 | 2.679758 | 6.891856e-04 | * | * | * |
| **BA.1.12** | 726 | 13 | 8.652264 | 8.652264 | 14.695473 | 1.691322e-06 | | * | |
| **BA.1.1.1** | 29279 | 13 | 6.096697 | 6.096697 | 19.806606 | 1.314026e-07 | | * | |
| **BA.1.1.4** | 865 | 13 | 2.298781 | 2.298781 | 27.402437 | 2.954312e-09 | | * | |
| **BA.1.20** | 11897 | 12 | 1.977700 | -2.054381 | 36.108762 | 3.794057e-11 | | * | |
| **BA.1.4** | 266 | 3 | 1.952310 | -6.100147 | 44.200294 | 6.643283e-13 | | | |
| **BA.1.3** | 17 | 3 | 1.952310 | -22.690541 | 77.381082 | 4.144167e-20 | | | |

### BA.2.65 <<

| | num_seq | t_ch_MAX | max_CL | CL@BC_t_ch_MAX | aic | PV | PV_OK | t_ch_MAX_OK | phyl_OK |
|---|---|---|---|---|---|---|---|---|---|
| **BA.2.65** | 1625 | 14 | 39.265934 | NaN | NaN | NaN | * | * | * |
| **BA.2.1** | 3218 | 14 | 33.715590 | 33.715590 | 48.568819 | 3.887457e-03 | * | * | |
| **BA.2.23** | 3557 | 14 | 31.855366 | 31.855366 | 52.289268 | 6.051707e-04 | * | * | |
| **BA.2.5** | 1908 | 14 | 31.264033 | 31.264033 | 53.471934 | 3.354626e-04 | * | * | |
| **BA.2.10** | 28641 | 16 | 30.685569 | 27.950964 | 60.098071 | 1.218872e-05 | * | | |
| **BA.2.3.14** | 529 | 14 | 28.961137 | 28.961137 | 58.077726 | 3.346535e-05 | * | * | |
| **BA.2.3** | 36275 | 14 | 27.794008 | 27.794008 | 60.411983 | 1.043860e-05 | * | * | |
| **BA.2.9.5** | 1119 | 14 | 27.611911 | 27.611911 | 60.776179 | 8.675566e-06 | | * | |
| **BA.2.56** | 3106 | 14 | 25.756141 | 25.756141 | 64.487718 | 1.357318e-06 | | * | |
| **BA.2.12** | 2844 | 14 | 25.275876 | 25.275876 | 65.448247 | 8.398857e-07 | | * | |

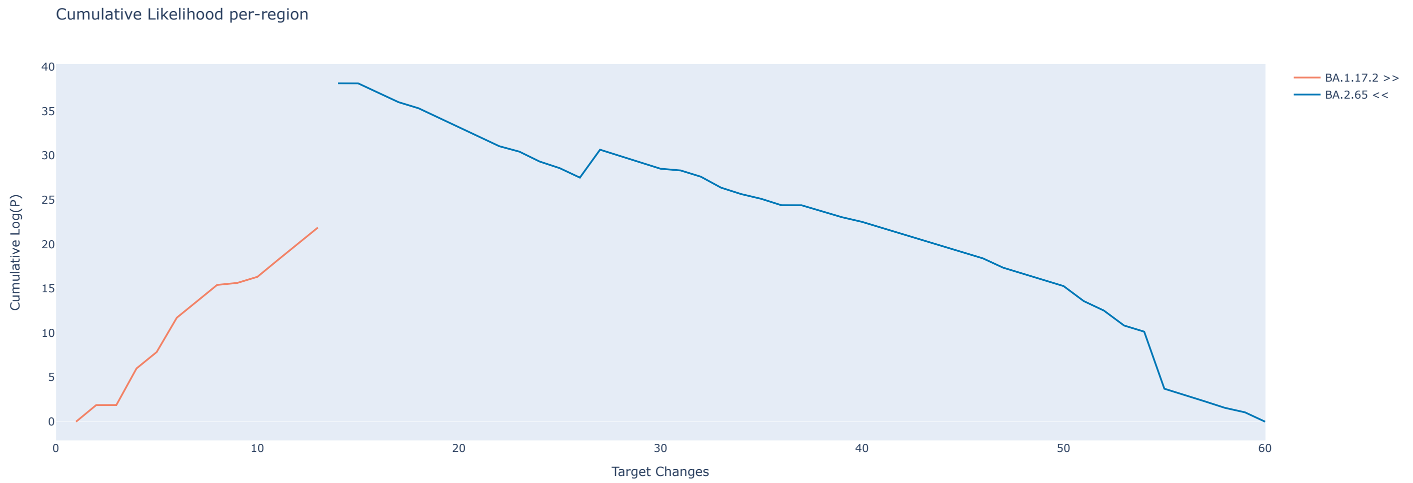


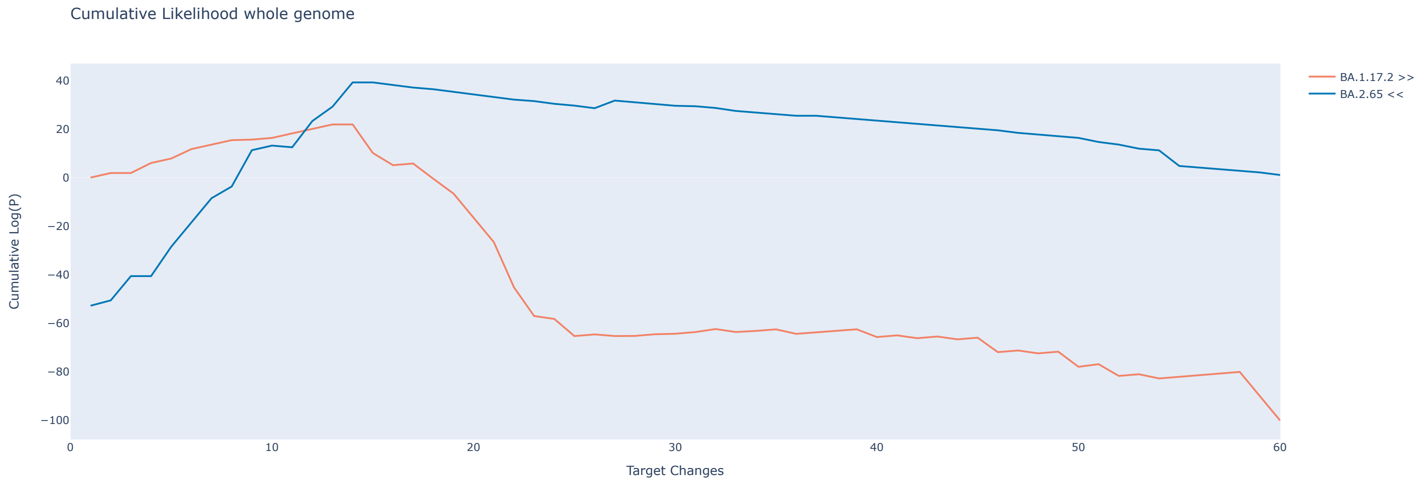


**Target sequence**

, 241_C|T, 2832_A|G, 3037_C|T, 3241_C|T, 5386_T|G, 5924_G|A, 6513_GTT|..., 8393_G|A, 10029_C|T, 10449_C|A, 11286_TGTCTGGTT|........., 11537_A|G, 13195_T|C, 14408_C|T, 15714_C|T, 17410_C|T, 18163_A|G, 19955_C|T, 20055_A|G, 21618_C|T, 21633_TACCCCCTG|........., 21987_G|A, 22200_T|G, 22578_G|A, 22674_C|T, 22679_T|C, 22813_G|T, 22882_T|G, 22992_G|A, 22995_C|A, 23013_A|C, 23040_A|G,

23055_A|G, 23063_A|T, 23075_T|C, 23403_A|G, 23525_C|T, 23599_T|G, 23604_C|A, 23854_C|A, 23948_G|T, 24424_A|T, 24469_T|A, 25000_C|T, 25584_C|T, 26060_C|T, 26270_C|T, 26577_C|G, 26709_G|A, 26858_C|T, 27259_A|C, 27382_GAT|CTC, 27807_C|T, 27945_C|T, 28271_A|T, 28311_C|T, 28362_GAGAACGCA|........., 28881_GGG|AAC, 29510_A|C, 29734_GAGGCCACGCGGAGTACGATCGAGTG|..........................

## Case 18 (1BP mid): XK                    test: OK

Target: (75%) 25 samples    Number of changes: 55
GT: BA.1* + BA.2*    GT BR: 15–17    GT BR coord: 18162 – 18165    Rank L1 L2: 5 3
BC: BA.1.7 + BA.2.13    BC BR: 15–16    BC BR coord: 17420 – 17421
Direction L1: <<    Initial region span: 1–14,16–55    Gap history (edge excluded): 14–16
Alt. candidates: [BA.1], [BA.2]
Model 1BP/2BP comparison:    –
Rec. model vs L1: 7.10e−205    Rec. model vs L2: 1.99e−185
Flags: Model_2BP_Bad_L1_opp

### BA.1.7 >>

| | num_seq | t_ch_MAX | max_CL | CL@BC_t_ch_MAX | aic | PV | PV_OK | t_ch_MAX_OK | phyl_OK |
|---|---|---|---|---|---|---|---|---|---|
| **BA.1.7** | 220 | 14 | 18.437216 | NaN | NaN | NaN | * | * | * |
| **BA.1.1.1** | 29279 | 15 | 18.300450 | 18.300450 | 1.399100 | 19244.873643 | * | * | |
| **BA.1.21** | 2247 | 14 | 18.275399 | 8.275399 | 21.449201 | 0.852144 | * | * | |
| **BA.1.12** | 726 | 14 | 16.881986 | 6.881986 | 24.236028 | 0.211189 | * | * | |
| **BA.1** | 127335 | 14 | 14.853274 | 12.512849 | 12.974303 | 59.145470 | * | * | * |
| **BA.1.1** | 349352 | 14 | 12.953356 | 8.371529 | 21.256942 | 0.937067 | * | * | |
| **BA.1.14** | 3706 | 11 | 11.802406 | -0.891634 | 39.783268 | 0.000089 | * | | |
| **BA.1.15** | 80931 | 14 | 10.990827 | 0.990827 | 36.018347 | 0.000584 | * | * | |
| **BA.1.17** | 27172 | 14 | 10.804897 | 0.804897 | 36.390206 | 0.000486 | * | * | |
| **BA.1.15.1** | 21857 | 14 | 10.486074 | 0.486074 | 37.027851 | 0.000353 | * | * | |

### BA.2.13 <<

| | num_seq | t_ch_MAX | max_CL | CL@BC_t_ch_MAX | aic | PV | PV_OK | t_ch_MAX_OK | phyl_OK |
|---|---|---|---|---|---|---|---|---|---|
| **BA.2.13** | 2436 | 16 | 30.426658 | NaN | NaN | NaN | * | * | * |
| **BA.2.44** | 567 | 24 | 28.845608 | 8.842664 | 92.314673 | 4.245463e-10 | | | |
| **BA.2** | 378689 | 16 | 27.659589 | 27.659589 | 54.680822 | 6.297610e-02 | * | * | * |
| **BA.2.71** | 409 | 22 | 24.560168 | 21.219167 | 67.561667 | 1.005355e-04 | * | | |
| **BA.2.36** | 5393 | 22 | 24.406331 | 22.466161 | 65.067678 | 3.491531e-04 | * | | |
| **BA.2.7** | 2003 | 16 | 24.210582 | 24.210582 | 61.578836 | 1.999216e-03 | * | * | |
| **BA.2.9** | 108830 | 16 | 23.305977 | 23.305977 | 63.388046 | 8.087668e-04 | * | * | |
| **BA.2.9.2** | 376 | 16 | 20.741136 | 20.741136 | 68.517727 | 6.220968e-05 | * | * | |
| **BA.2.25** | 331 | 16 | 18.415541 | 18.415541 | 73.168918 | 6.083076e-06 | | * | |
| **BA.2.45** | 433 | 36 | 17.800502 | 4.551433 | 100.897135 | 5.789356e-12 | | | |

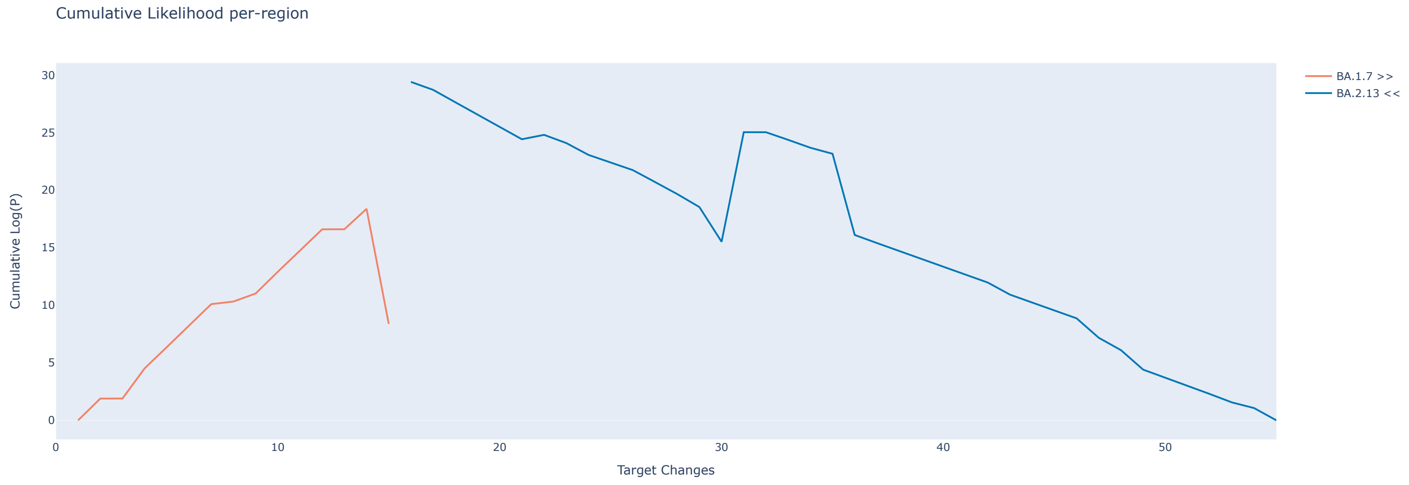


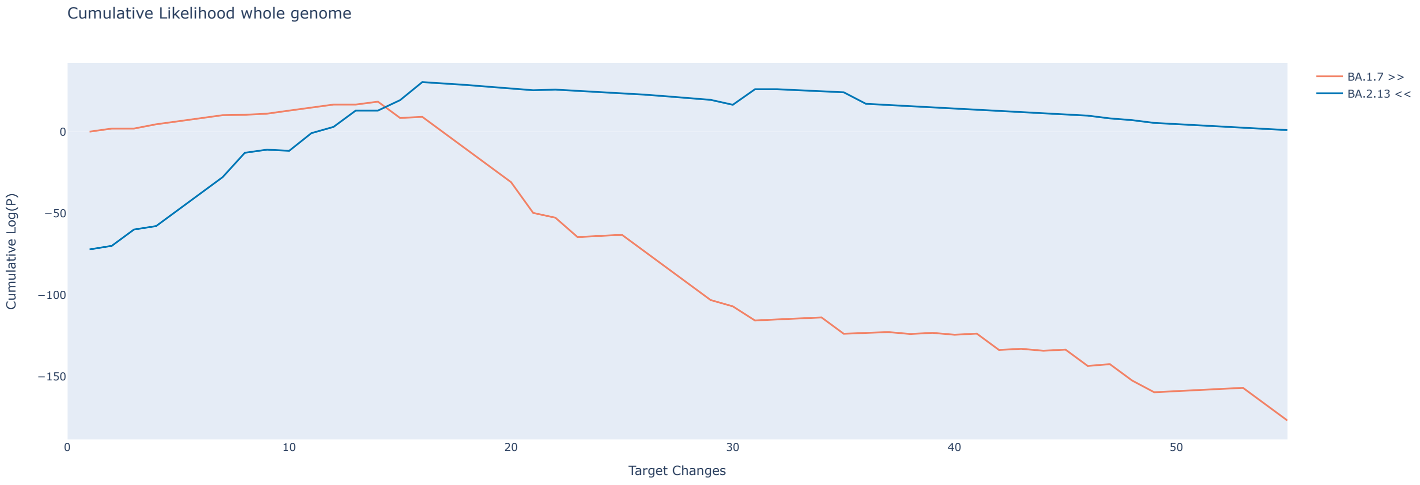


**Target sequence**

, 241_C|T, 2832_A|G, 3037_C|T, 5147_C|T, 5386_T|G, 6513_GTT|..., 8393_G|A, 10029_C|T, 10449_C|A, 11286_TGTCTGGTT|........., 11537_A|G, 13195_T|C, 14408_C|T, 15240_C|T, 16064_A|G, 18163_A|G,

19955_C|T, 20055_A|G, 21618_C|T, 21633_TACCCCCTG|........., 21987_G|A, 22578_G|A, 22674_C|T, 22679_T|C, 22686_C|T, 22688_A|G, 22775_G|A, 22786_A|C, 22792_C|T, 22813_G|T, 23403_A|G, 23525_C|T, 23599_T|G, 23604_C|A, 23767_A|G, 23854_C|A, 23948_G|T, 24424_A|T, 24469_T|A, 25000_C|T, 25584_C|T, 26060_C|T, 26270_C|T, 26577_C|G, 26709_G|A, 26858_C|T, 27259_A|C, 27382_GAT|CTC, 27807_C|T, 28271_A|T, 28311_C|T, 28362_GAGAACGCA|........., 28881_GGG|AAC, 29510_A|C, 29734_GAGGCCACGCGGAGTACGATCGAGTG|.........................

## Case 19 (1BP mid): XM                    test: OK

Target: (75%) 301 samples
GT: BA.1.1* + BA.2*
BC: BA.1.1.16 + BA.2.33
Direction L1: <<
Alt. candidates: [BA.1.1, B.1.1.529], [BA.2]
Model 1BP/2BP comparison:
Rec. model vs L1: 6.61e-224
Flags: Model_2BP_Bad_L1_opp

Number of changes: 59
GT BR: 14-17
BC BR: 14-15
Initial region span: 1-14,15-59
–
Rec. model vs L2: 2.17e-217

GT BR coord: 17409 - 19996
BC BR coord: 17420 - 17421
Gap history (edge excluded):

Rank L1 L2: 3 7

### BA.1.1.16 >>

| | num_seq | t_ch_MAX | max_CL | CL@BC_t_ch_MAX | aic | PV | PV_OK | t_ch_MAX_OK | phyl_OK |
|---|---|---|---|---|---|---|---|---|---|
| **BA.1.1.16** | 1583 | 14 | 18.309145 | NaN | NaN | NaN | * | * | * |
| **BA.1.1.13** | 3131 | 14 | 18.186278 | 18.186278 | 3.627445 | 0.882497 | * | * | |
| **BA.1.1** | 349352 | 14 | 18.149208 | 18.149208 | 3.701584 | 0.852144 | * | * | * |
| **BA.1.1.14** | 8250 | 12 | 16.401275 | 11.720061 | 16.559878 | 0.001374 | * | | |
| **BA.1.1.15** | 6410 | 12 | 16.390062 | 5.097834 | 29.804331 | 0.000002 | | | |
| **B.1.1.529** | 1110 | 14 | 15.383698 | 15.383698 | 9.232603 | 0.053665 | * | * | * |
| **BA.1.1.18** | 27122 | 14 | 14.687521 | 14.687521 | 10.624959 | 0.026783 | * | * | |
| **BA.1.1.10** | 1083 | 11 | 14.583548 | 12.322203 | 15.355594 | 0.002504 | * | | |
| **BA.1.21** | 2247 | 14 | 13.769899 | 13.769899 | 12.460202 | 0.010673 | * | * | |
| **BA.1.1.6** | 86 | 14 | 12.587793 | 12.587793 | 14.824413 | 0.003280 | * | * | |

### BA.2.33 <<

| | num_seq | t_ch_MAX | max_CL | CL@BC_t_ch_MAX | aic | PV | PV_OK | t_ch_MAX_OK | phyl_OK |
|---|---|---|---|---|---|---|---|---|---|
| **BA.2.33** | 389 | 15 | 36.280540 | NaN | NaN | NaN | * | * | * |
| **BA.2.26** | 490 | 15 | 35.884287 | 35.884287 | 26.231426 | 0.673680 | * | * | |
| **BA.2.19** | 365 | 15 | 35.789071 | 35.789071 | 26.421858 | 0.612626 | * | * | |
| **BA.2.23** | 3557 | 15 | 35.713545 | 35.713545 | 26.572910 | 0.568360 | * | * | |
| **BA.2.63** | 120 | 16 | 35.470144 | 27.128729 | 43.742543 | 0.000106 | * | * | |
| **BA.2.40** | 17 | 16 | 35.432931 | 29.548834 | 38.902332 | 0.001195 | * | * | |
| **BA.2** | 378689 | 15 | 35.423967 | 35.423967 | 27.152066 | 0.425283 | * | * | * |
| **BA.2.5** | 1908 | 15 | 35.338643 | 35.338643 | 27.322713 | 0.390628 | * | * | |
| **BA.2.31** | 1389 | 15 | 35.250412 | 35.250412 | 27.499176 | 0.357007 | * | * | |
| **BA.2.29** | 1418 | 15 | 35.218281 | 35.218281 | 27.563438 | 0.346456 | * | * | |

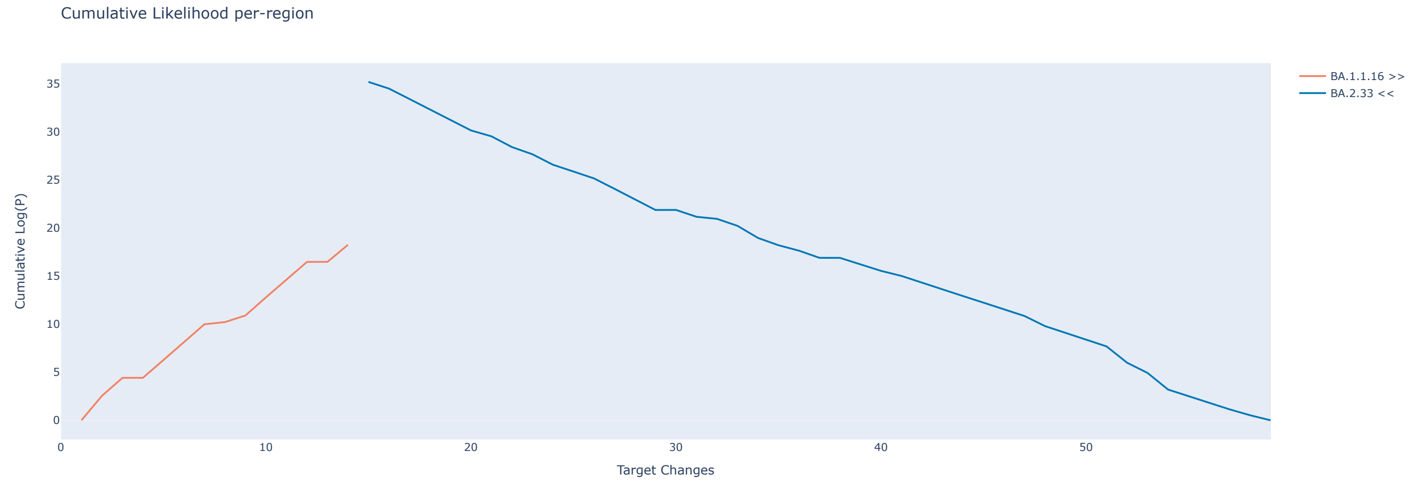


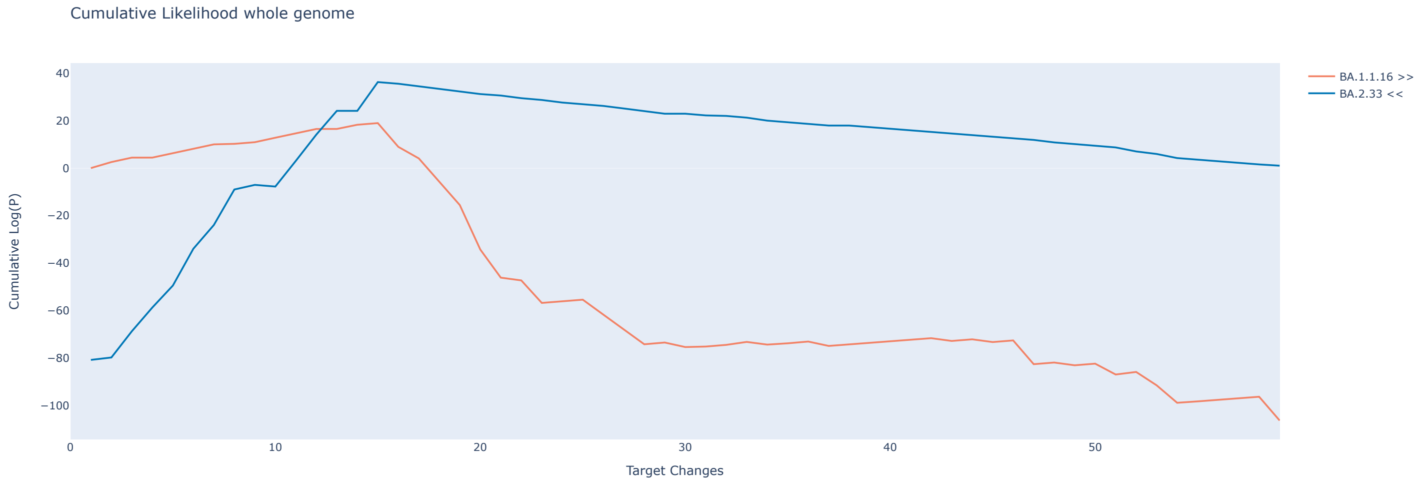


**Target sequence**

, 241_C|T, 2470_C|T, 2832_A|G, 3037_C|T, 5386_T|G, 6513_GTT|..., 8393_G|A, 10029_C|T, 10449_C|A, 11286_TGTCTGGTT|........., 11537_A|G, 13195_T|C, 14408_C|T, 15240_C|T, 18163_A|G, 19955_C|T,

20055_A|G, 21618_C|T, 21633_TACCCCCTG|........, 21987_G|A, 22200_T|G, 22578_G|A, 22674_C|T, 22679_T|C, 22686_C|T, 22688_A|G, 22775_G|A, 22786_A|C, 22813_G|T, 22992_G|A, 22995_C|A, 23013_A|C, 23040_A|G, 23055_A|G, 23063_A|T, 23075_T|C, 23403_A|G, 23525_C|T, 23599_T|G, 23604_C|A, 23854_C|A, 23948_G|T, 24424_A|T, 24469_T|A, 25000_C|T, 25584_C|T, 26060_C|T, 26270_C|T, 26577_C|G, 26709_G|A, 26858_C|T, 27259_A|C, 27382_GAT|CTC, 27807_C|T, 28271_A|T, 28311_C|T, 28362_GAGAACGCA|........, 28881_GGG|AAC, 29510_A|C

## Case 20 (1BP mid): XV test: OK

Target: (75%) 27 samples
GT: BA.1* + BA.2*
BC: BA.1.6 + BA.2.25
Direction L1: <<
Alt. candidates: [BA.1], [BA.2]
Model 1BP/2BP comparison: -
Rec. model vs L1: 1.37e-256
Flags: Model_2BP_Bad_L1_opp

Number of changes: 61
GT BR: 12-14
BC BR: 12-13
Initial region span: 1-12,13-61
Rec. model vs L2: 1.73e-167

GT BR coord: 13195 - 15714
BC BR coord: 13196 - 13197
Gap history (edge excluded):

Rank L1 L2: 4 3

### BA.1.6 >>

| | num_seq | t_ch_MAX | max_CL | CL@BC_t_ch_MAX | aic | PV | PV_OK | t_ch_MAX_OK | phyl_OK |
|---|---|---|---|---|---|---|---|---|---|
| **BA.1.6** | 208 | 12 | 16.421408 | NaN | NaN | NaN | * | * | * |
| **BA.1.21** | 2247 | 12 | 13.916832 | 13.916832 | 4.166337 | 0.081676 | * | * | |
| **BA.1.1.1** | 29279 | 12 | 13.415145 | 13.415145 | 5.169710 | 0.049539 | * | * | |
| **BA.1** | 127335 | 12 | 12.563030 | 12.563030 | 6.873941 | 0.021174 | * | * | * |
| **BA.1.14** | 3706 | 11 | 11.593434 | 7.133244 | 17.733512 | 0.000093 | * | * | |
| **BA.1.1** | 349352 | 12 | 11.211145 | 11.211145 | 9.577709 | 0.005462 | * | * | |
| **BA.1.20** | 11897 | 11 | 10.475005 | 6.442924 | 19.114152 | 0.000047 | * | * | |
| **BA.1.1.15** | 6410 | 12 | 10.354041 | 10.354041 | 11.291918 | 0.002323 | * | * | |
| **BA.1.1.14** | 8250 | 12 | 10.116092 | 10.116092 | 11.767817 | 0.001827 | * | * | |
| **BA.1.15** | 80931 | 12 | 8.868317 | 8.868317 | 14.263366 | 0.000526 | * | * | |

### BA.2.25 <<

| | num_seq | t_ch_MAX | max_CL | CL@BC_t_ch_MAX | aic | PV | PV_OK | t_ch_MAX_OK | phyl_OK |
|---|---|---|---|---|---|---|---|---|---|
| **BA.2.25** | 331 | 13 | 39.990749 | NaN | NaN | NaN | * | * | * |
| **BA.2.69** | 96 | 14 | 38.260158 | 27.529588 | 58.940824 | 0.000004 | | * | |
| **BA.2** | 378689 | 13 | 38.190137 | 38.190137 | 37.619726 | 0.165299 | * | * | * |
| **BA.2.7** | 2003 | 13 | 37.533138 | 37.533138 | 38.933723 | 0.085863 | * | * | |
| **BA.2.9** | 108830 | 13 | 36.238040 | 36.238040 | 41.523920 | 0.023518 | * | * | |
| **BA.2.14** | 992 | 20 | 35.411132 | 32.844490 | 48.311019 | 0.000789 | * | | |
| **BA.2.71** | 409 | 20 | 35.402450 | 32.504354 | 48.991293 | 0.000561 | * | | |
| **BA.2.9.5** | 1119 | 13 | 35.364624 | 35.364624 | 43.270752 | 0.009804 | * | * | |
| **BA.2.36** | 5393 | 20 | 34.874664 | 33.409476 | 47.181048 | 0.001388 | * | | |
| **BA.2.9.6** | 294 | 13 | 34.394612 | 34.394612 | 45.210775 | 0.003716 | * | * | |

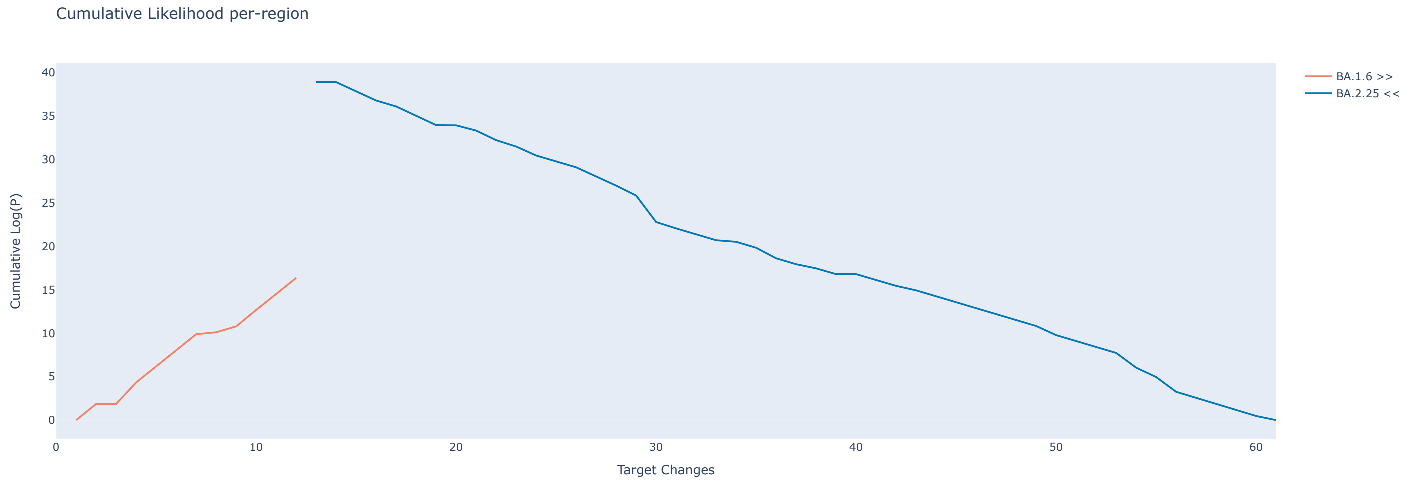


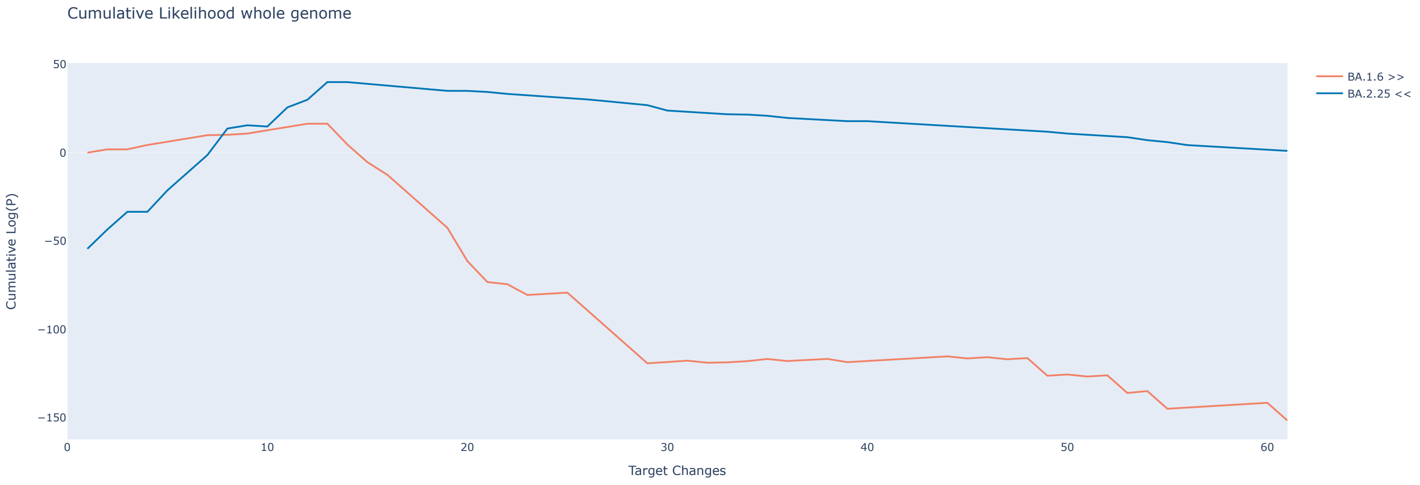


**Target sequence**

, 241_C|T, 2832_A|G, 3037_C|T, 3583_C|T, 5386_T|G, 6513_GTT|..., 8393_G|A, 10029_C|T, 10449_C|A, 11286_TGTCTGGTT|........, 11537_A|G, 13195_T|C, 14408_C|T, 15714_C|T, 17410_C|T, 18163_A|G,

19955_C|T, 20055_A|G, 21618_C|T, 21987_G|A, 22200_T|G, 22578_G|A, 22674_C|T, 22679_T|C, 22686_C|T, 22688_A|G, 22775_G|A, 22786_A|C, 22792_C|T, 22813_G|T, 22882_T|G, 22992_G|A, 22995_C|A, 23013_A|C, 23040_A|G, 23055_A|G, 23063_A|T, 23075_T|C, 23403_A|G, 23525_C|T, 23599_T|G, 23604_C|A, 23854_C|A, 23948_G|T, 24424_A|T, 24469_T|A, 25000_C|T, 25584_C|T, 26060_C|T, 26270_C|T, 26577_C|G, 26709_G|A, 26858_C|T, 27259_A|C, 27382_GAT|CTC, 27807_C|T, 28271_A|T, 28311_C|T, 28362_GAGAACGCA|........., 28881_GGG|AAC, 29510_A|C

# Case 21 (1BP mid): XY test: OK

Target: (75%) 44 samples
Number of changes: 68
GT: BA.1* + BA.2*
GT BR: 14–16
GT BR coord: 11539 – 12880
Rank L1 L2: 11 1
BC: BA.1.1 + BA.2
BC BR: 14–15
BC BR coord: 11547 – 11548
Direction L1: <<
Initial region span: 1–14,16–68
Gap history (edge excluded): 14–16
Alt. candidates: [], []
Model 1BP/2BP comparison: –
Rec. model vs L1: 2.08e–113
Rec. model vs L2: 3.43e–67
Flags: Model_2BP_Bad_L1_opp

## BA.1.1 >>

| | num_seq | t_ch_MAX | max_CL | CL@BC_t_ch_MAX | aic | PV | PV_OK | t_ch_MAX_OK | phyl_OK |
|---|---|---|---|---|---|---|---|---|---|
| **BA.1.1** | 349352 | 14 | 16.772306 | NaN | NaN | NaN | * | * | * |
| **B.1.1.529** | 1110 | 14 | 3.200904 | 3.200904 | 23.598192 | 1.278274e-06 | | * | * |
| **BA.1.1.13** | 3131 | 14 | 3.037686 | 3.037686 | 23.924629 | 1.089273e-06 | | * | * |
| **BA.1.15.1** | 21857 | 8 | 1.403558 | -9.141060 | 48.282121 | 5.590234e-12 | | | * |
| **BA.1.1.14** | 8250 | 11 | 0.285990 | -6.009592 | 42.019184 | 1.278709e-10 | | | * |
| **BA.1.1.16** | 1583 | 11 | 0.260584 | -6.019209 | 42.038417 | 1.265986e-10 | | | * |
| **BA.1.1.15** | 6410 | 11 | 0.130326 | -6.174678 | 42.349356 | 1.084209e-10 | | | * |

## BA.2 <<

| | num_seq | t_ch_MAX | max_CL | CL@BC_t_ch_MAX | aic | PV | PV_OK | t_ch_MAX_OK | phyl_OK |
|---|---|---|---|---|---|---|---|---|---|
| **BA.2** | 378689 | 16 | 40.243825 | NaN | NaN | NaN | * | * | * |
| **BA.2.52** | 627 | 29 | 31.653414 | 2.606746 | 122.786507 | 6.449139e-17 | | | * |
| **BA.2.36** | 5393 | 29 | 30.325054 | 12.631287 | 102.737425 | 1.456478e-12 | | | * |
| **BA.2.7** | 2003 | 29 | 29.251008 | 9.225758 | 109.548484 | 4.836500e-14 | | | * |
| **BA.2.24** | 1290 | 30 | 26.770377 | -1.902442 | 131.804883 | 7.128614e-19 | | | * |
| **BA.2.9.5** | 1119 | 29 | 26.457990 | 6.431426 | 115.137148 | 2.955821e-15 | | | * |
| **BA.2.9** | 108830 | 29 | 26.330497 | 25.852163 | 76.295675 | 8.029286e-07 | | | * |
| **BA.2.1** | 3218 | 29 | 25.394932 | 11.194360 | 105.611280 | 3.468098e-13 | | | * |
| **BA.2.3.8** | 143 | 29 | 24.591117 | -3.761810 | 135.523621 | 1.109730e-19 | | | * |
| **BA.2.56** | 3106 | 38 | 23.365226 | 4.981569 | 118.036861 | 6.933477e-16 | | | * |

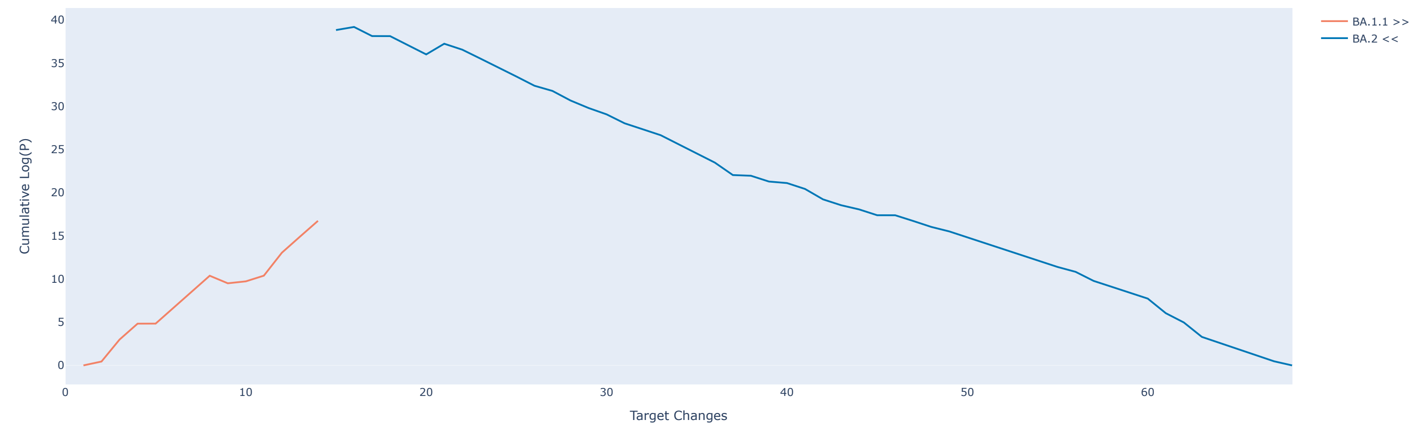


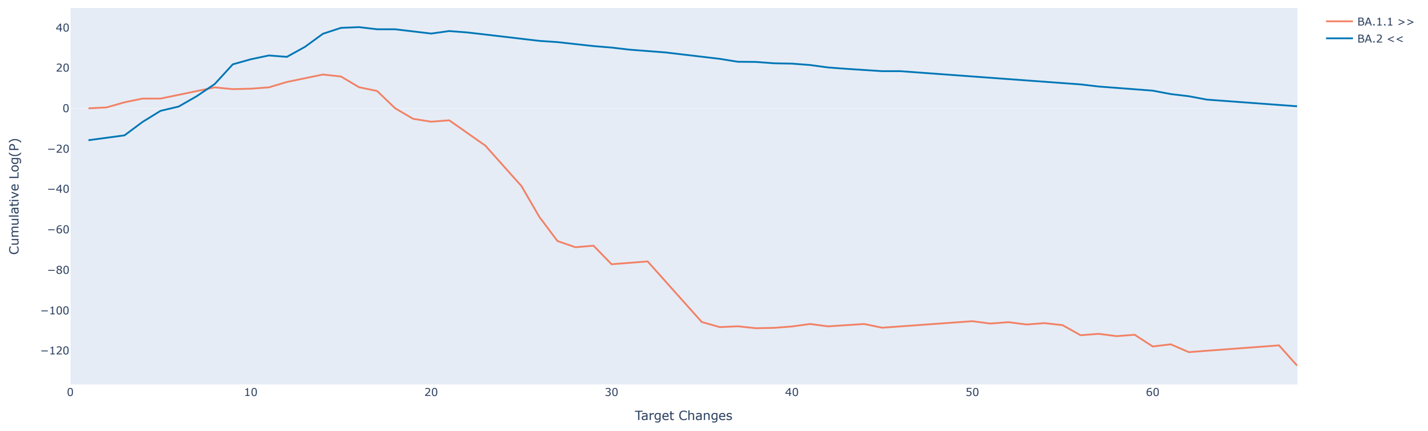


**Target sequence**

, 241_C|T, 1585_A|G, 2470_C|T, 2832_A|G, 3037_C|T, 5386_T|G, 6513_GTT|..., 8393_G|A, 9611_C|T, 10029_C|T, 10449_C|A, 11049_T|C, 11286_TGTCTGGTT|........., 11537_A|G, 12085_C|T, 12880_C|T, 14408_C|T, 15714_C|T, 17410_C|T, 17443_G|T, 18163_A|G, 19955_C|T, 20055_A|G, 21618_C|T, 21633_TACCCCCTG|........., 21987_G|A, 22200_T|G, 22351_A|G, 22578_G|A, 22674_C|T, 22679_T|C, 22686_C|T, 22688_A|G, 22775_G|A, 22786_A|C, 22792_C|T, 22813_G|T, 22992_G|A, 22995_C|A, 23013_A|C, 23040_A|G, 23055_A|G, 23063_A|T, 23075_T|C, 23403_A|G, 23525_C|T, 23599_T|G, 23604_C|A, 23854_C|A, 23948_G|T, 24424_A|T, 24469_T|A, 25000_C|T, 25584_C|T, 26058_C|T, 26060_C|T, 26270_C|T, 26577_C|G, 26709_G|A, 26858_C|T, 27259_A|C, 27382_GAT|CTC, 27807_C|T, 28271_A|T, 28311_C|T, 28362_GAGAACGCA|........., 28881_GGG|AAC, 29510_A|C

## Case 22 (1BP mid): XZ test: OK

Target: (75%) 49 samples
Number of changes: 65
GT: BA.2* + BA.1*
GT BR: 55-56
GT BR coord: 26061 - 26251
Rank L1 L2: 1 11
BC: BA.2 + BA.1.1.12
BC BR: 56-57
BC BR coord: 26274 - 26275
Direction L1: >>
Initial region span: 1-56,57-65
Gap history (edge excluded):
Alt. candidates: [BA.2.34, BA.2.65], []
Model 1BP/2BP comparison: 1BP vs 2BP: 2.13e-03
Rec. model vs L1: 2.84e-23
Rec. model vs L2: 1.62e-296
Flags: Model_1BP_Best

### BA.2 >>

| | num_seq | t_ch_MAX | max_CL | CL@BC_t_ch_MAX | aic | PV | PV_OK | t_ch_MAX_OK | phyl_OK |
|---|---|---|---|---|---|---|---|---|---|
| **BA.2** | 378689 | 56 | 42.686241 | NaN | NaN | NaN | * | * | * |
| **BA.2.9** | 108830 | 54 | 41.981863 | 39.997347 | 50.005306 | 6.788094e-02 | * | | * |
| **BA.2.34** | 325 | 56 | 36.572625 | 36.572625 | 56.854751 | 2.220551e-03 | * | * | * |
| **BA.2.6** | 1333 | 53 | 32.628654 | 27.738877 | 74.522247 | 3.231982e-07 | | | * |
| **BA.2.65** | 1625 | 56 | 32.445704 | 32.445704 | 65.108592 | 3.571285e-05 | * | * | * |
| **BA.2.3** | 36275 | 54 | 32.328021 | 29.555239 | 70.889522 | 1.984785e-06 | | | * |
| **BA.2.56** | 3106 | 34 | 30.702250 | 28.908183 | 72.183635 | 1.041342e-06 | | | * |
| **BA.2.51** | 288 | 48 | 30.271597 | 28.796352 | 72.407296 | 9.282173e-07 | | | * |
| **BA.2.30** | 73 | 55 | 29.728241 | 23.419247 | 83.161506 | 4.298499e-09 | | * | * |
| **BA.2.36** | 5393 | 56 | 29.416499 | 29.416499 | 71.167001 | 1.725489e-06 | | * | * |

### BA.1.1.12 <<

| | num_seq | t_ch_MAX | max_CL | CL@BC_t_ch_MAX | aic | PV | PV_OK | t_ch_MAX_OK | phyl_OK |
|---|---|---|---|---|---|---|---|---|---|
| **BA.1.1.12** | 1392 | 57 | 7.786122 | NaN | NaN | NaN | * | * | * |
| **BA.1.3** | 17 | 57 | 7.769603 | 7.769603 | 2.460795 | 0.985112 | * | * | |
| **BA.1.5** | 122 | 57 | 7.763763 | 7.763763 | 2.472474 | 0.980199 | * | * | |
| **BA.1.9** | 345 | 57 | 7.759709 | 7.759709 | 2.480582 | 0.975310 | * | * | |
| **BA.1.1.13** | 3131 | 57 | 7.755146 | 7.755146 | 2.489709 | 0.970446 | * | * | |
| **BA.1.1.18** | 27122 | 57 | 7.750197 | 7.750197 | 2.499607 | 0.965605 | * | * | |
| **BD.1** | 2033 | 57 | 7.744761 | 7.744761 | 2.510477 | 0.960789 | * | * | |
| **BA.1.1.17** | 229 | 57 | 7.737327 | 7.737327 | 2.525347 | 0.951229 | * | * | |
| **BA.1.17.2** | 77688 | 57 | 7.731957 | 7.731957 | 2.536087 | 0.946485 | * | * | |
| **BA.1.1.10** | 1083 | 57 | 7.728517 | 7.728517 | 2.542966 | 0.946485 | * | * | |

Cumulative Likelihood per-region

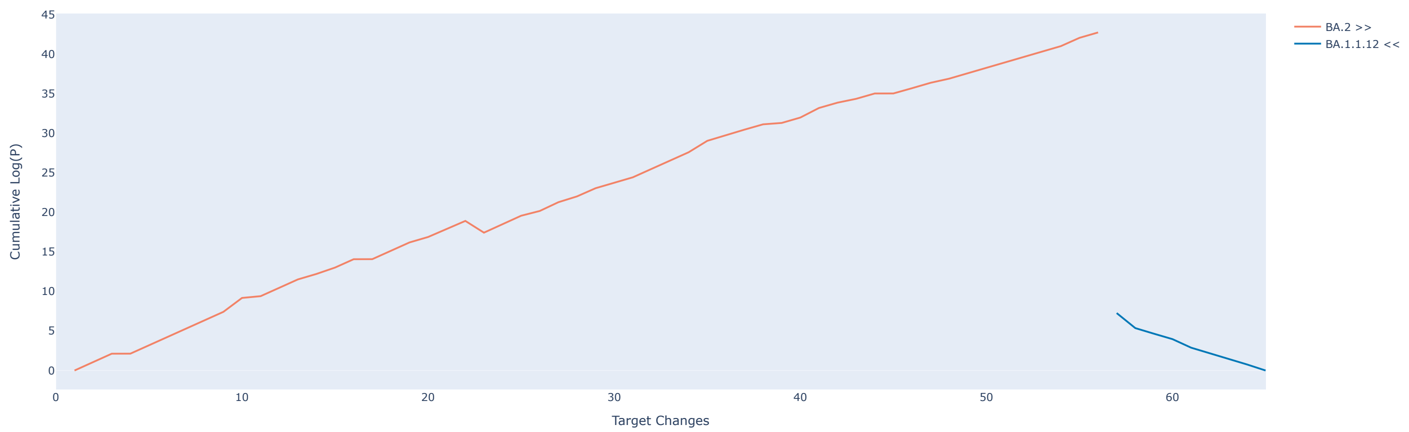


Cumulative Likelihood whole genome

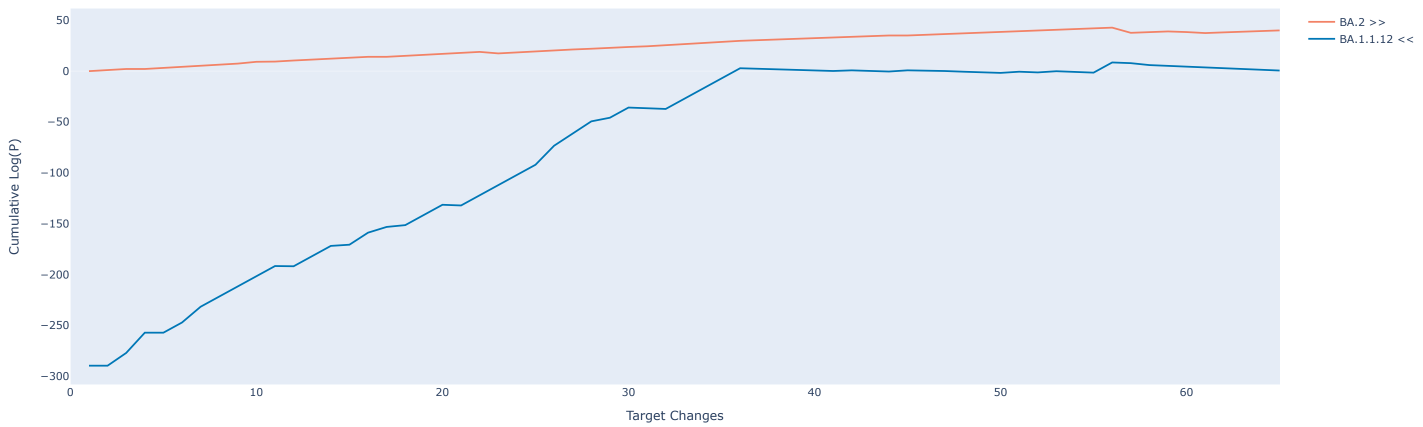


**Target sequence**

, 241_C|T, 670_T|G, 2790_C|T, 3037_C|T, 4184_G|A, 4321_C|T, 9344_C|T, 9424_A|G, 9534_C|T, 9866_C|T, 10029_C|T, 10198_C|T, 10447_G|A, 10449_C|A, 11288_TCTGGTTTT|........., 12880_C|T, 14408_C|T, 15714_C|T, 17410_C|T, 18163_A|G, 19955_C|T, 20055_A|G, 20398_G|A, 21618_C|T, 21633_TACCCCCTG|........., 21987_G|A, 22200_T|G, 22578_G|A, 22674_C|T, 22679_T|C, 22686_C|T, 22688_A|G, 22775_G|A, 22786_A|C, 22792_C|T, 22813_G|T, 22882_T|G, 22992_G|A, 22995_C|A, 23013_A|C, 23040_A|G, 23055_A|G, 23063_A|T, 23075_T|C, 23403_A|G, 23525_C|T, 23599_T|G, 23604_C|A, 23854_C|A, 23948_G|T, 24424_A|T, 24469_T|A, 25000_C|T, 25584_C|T, 26060_C|T, 26270_C|T, 26530_A|G, 26577_C|G, 26709_G|A, 27259_A|C, 27807_C|T, 28271_A|T, 28311_C|T, 28362_GAGAACGCA|........., 28881_GGG|AAC

## Case 23 (1BP 5'): XAA test: OK

Target: (75%) 33 samples
Number of changes: 67
GT: BA.1* + BA.2*
GT BR: 7-8
GT BR coord: 8938 - 9344
Rank L1 L2: 2 1
BC: BA.1.20 + BA.2
BC BR: 7-8
BC BR coord: 8250 - 8251
Direction L1: <<
Initial region span: 1-7,8-67
Gap history (edge excluded):
Alt. candidates: [BA.1], [BA.2.5]
Model 1BP/2BP comparison: -
Rec. model vs L1: 8.78e-177
Rec. model vs L2: 3.56e-37
Flags: Model_2BP_Bad_L1_opp

### BA.1.20 >>

| | num_seq | t_ch_MAX | max_CL | CL@BC_t_ch_MAX | aic | PV | PV_OK | t_ch_MAX_OK | phyl_OK |
|---|---|---|---|---|---|---|---|---|---|
| **BA.1.20** | 11897 | 7 | 7.894962 | NaN | NaN | NaN | * | * | * |
| **BA.1** | 127335 | 6 | 3.755976 | 2.335085 | 13.329830 | 3.848776e-03 | * | * | * |
| **BA.1.15** | 80931 | 6 | 3.556360 | 3.140763 | 11.718475 | 8.608545e-03 | * | * | |
| **BA.1.1.15** | 6410 | 6 | 3.506372 | -6.493628 | 30.987256 | 5.629922e-07 | | * | |
| **BA.1.1.18** | 27122 | 6 | 2.149546 | -1.963360 | 21.926719 | 5.222235e-05 | * | * | |
| **BA.1.1** | 349352 | 6 | 1.147572 | 0.227331 | 17.545337 | 4.666178e-04 | * | * | |

### BA.2 <<

| | num_seq | t_ch_MAX | max_CL | CL@BC_t_ch_MAX | aic | PV | PV_OK | t_ch_MAX_OK | phyl_OK |
|---|---|---|---|---|---|---|---|---|---|
| **BA.2** | 378689 | 8 | 47.755622 | NaN | NaN | NaN | * | * | * |
| **BA.2.5** | 1908 | 8 | 38.584412 | 38.584412 | 58.831177 | 1.041165e-04 | * | * | * |
| **BA.2.33** | 389 | 24 | 35.584573 | 28.311235 | 79.377530 | 3.590408e-09 | | | * |
| **BA.2.63** | 120 | 24 | 35.470144 | 18.922576 | 98.154849 | 3.015019e-13 | | | * |
| **BA.2.40** | 17 | 24 | 35.432931 | 21.600651 | 92.798699 | 4.375501e-12 | | | * |
| **BA.2.37** | 2257 | 8 | 35.351397 | 35.351397 | 65.297206 | 4.098047e-06 | | * | * |
| **BA.2.26** | 490 | 24 | 35.194461 | 20.503571 | 94.992858 | 1.463779e-12 | | | * |
| **BA.2.19** | 365 | 24 | 35.109679 | 9.480197 | 117.039606 | 2.384398e-17 | | | * |
| **BA.2.23** | 3557 | 24 | 35.020393 | 27.714043 | 80.571914 | 1.980335e-09 | | | * |
| **BA.2.31** | 1389 | 24 | 34.577016 | 20.424706 | 95.150589 | 1.351238e-12 | | | * |

Cumulative Likelihood per-region

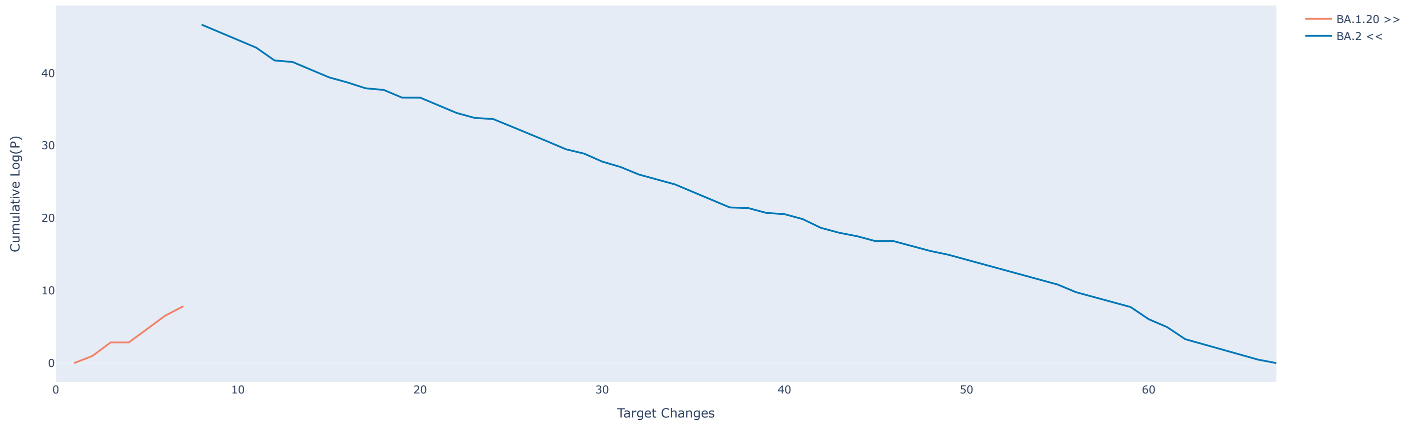


Cumulative Likelihood whole genome

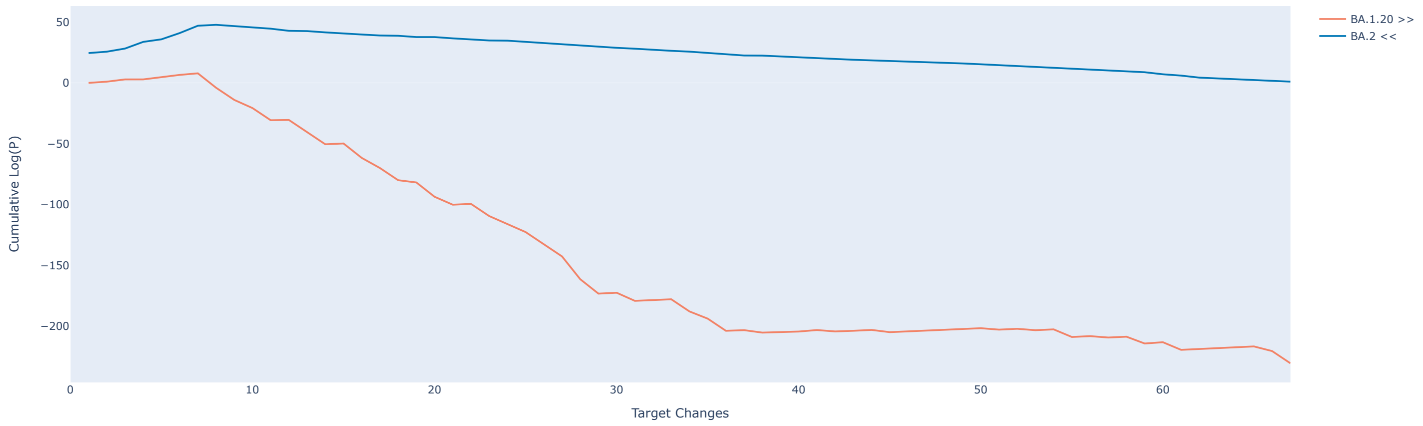


**Target sequence**

, 241_C|T, 1610_A|G, 2832_A|G, 3037_C|T, 5386_T|G, 6513_GTT|..., 8247_C|T, 9344_C|T, 9424_A|G, 9534_C|T, 9866_C|T, 10029_C|T, 10198_C|T, 10447_G|A, 10449_C|A, 11288_TCTGGTTTT|........., 12445_C|T, 12880_C|T, 14408_C|T, 15714_C|T, 17410_C|T, 18163_A|G, 19356_A|G, 19955_C|T, 20055_A|G, 21618_C|T, 21633_TACCCCCTG|........., 21987_G|A, 22200_T|G, 22578_G|A, 22674_C|T, 22679_T|C, 22686_C|T, 22688_A|G, 22775_G|A, 22786_A|C, 22813_G|T, 22992_G|A, 22995_C|A, 23013_A|C, 23040_A|G, 23055_A|G, 23063_A|T, 23075_T|C, 23403_A|G, 23525_C|T, 23599_T|G, 23604_C|A, 23854_C|A, 23948_G|T, 24424_A|T, 24469_T|A, 25000_C|T, 25584_C|T, 26060_C|T, 26270_C|T, 26577_C|G, 26709_G|A, 26858_C|T, 27259_A|C, 27382_GAT|CTC, 27807_C|T, 28271_A|T, 28311_C|T, 28362_GAGAACGCA|........., 28881_GGG|AAC, 29510_A|C

## Case 24 (1BP 5'): XAB test: OK

Target: (75%) 73 samples
GT: BA.1* + BA.2*
BC: BA.1.1.16 + BA.2
Direction L1: <<
Alt. candidates: [BA.1.1, B.1.1.529], [BA.2.9, BA.2.51, BA.2.34, BA.2.36, BA.2.14, BA.2.25, BA.2.27]
Model 1BP/2BP comparison:
Rec. model vs L1: 1.93e-240
Flags: Model_2BP_Bad_L1_opp

Number of changes: 67
GT BR: 6-7 GT BR coord: 6515 - 8393 Rank L1 L2: 11 1
BC BR: 6-7 BC BR coord: 6512 - 6513
Initial region span: 1-6,7-67 Gap history (edge excluded):
-
Rec. model vs L2: 2.86e-48

## BA.1.1.16 >>

| | num_seq | t_ch_MAX | max_CL | CL@BC_t_ch_MAX | aic | PV | PV_OK | t_ch_MAX_OK | phyl_OK |
|---|---|---|---|---|---|---|---|---|---|
| **BA.1.1.16** | 1583 | 6 | 8.195140 | NaN | NaN | NaN | * | * | * |
| **BA.1.1.2** | 7493 | 6 | 8.185254 | 8.185254 | -2.370508 | 0.990050 | * | * | |
| **BA.1.1.18** | 27122 | 6 | 8.174906 | 8.174906 | -2.349812 | 0.980199 | * | * | |
| **BA.1.1.10** | 1083 | 6 | 8.144769 | 8.144769 | -2.289537 | 0.951229 | * | * | |
| **BA.1.1** | 349352 | 6 | 8.125137 | 8.125137 | -2.250274 | 0.932394 | * | * | * |
| **BA.1.1.14** | 8250 | 6 | 8.102711 | 8.102711 | -2.205422 | 0.913931 | * | * | |
| **BA.1.1.13** | 3131 | 6 | 8.091567 | 8.091567 | -2.183133 | 0.900325 | * | * | |
| **BA.1.1.15** | 6410 | 6 | 8.089843 | 8.089843 | -2.179686 | 0.900325 | * | * | |
| **B.1.1.529** | 1110 | 6 | 6.113152 | 6.113152 | 1.773696 | 0.124930 | * | * | * |
| **BA.1.1.6** | 86 | 4 | 4.458730 | 2.449708 | 9.100583 | 0.003199 | * | | |

## BA.2 <<

| | num_seq | t_ch_MAX | max_CL | CL@BC_t_ch_MAX | aic | PV | PV_OK | t_ch_MAX_OK | phyl_OK |
|---|---|---|---|---|---|---|---|---|---|
| **BA.2** | 378689 | 7 | 50.537672 | NaN | NaN | NaN | * | * | * |
| **BA.2.9** | 108830 | 7 | 47.537617 | 47.537617 | 44.924766 | 4.978707e-02 | * | * | * |
| **BA.2.51** | 288 | 7 | 46.308487 | 46.308487 | 47.383025 | 1.455239e-02 | * | * | * |
| **BA.2.34** | 325 | 7 | 44.896839 | 44.896839 | 50.206322 | 3.535148e-03 | * | * | * |
| **BA.2.36** | 5393 | 7 | 44.034130 | 44.034130 | 51.931740 | 1.495941e-03 | * | * | * |
| **BA.2.14** | 992 | 7 | 44.025502 | 44.025502 | 51.948995 | 1.481056e-03 | * | * | * |
| **BA.2.25** | 331 | 7 | 41.973860 | 41.973860 | 56.052280 | 1.906636e-04 | * | * | * |
| **BA.2.27** | 215 | 7 | 41.549129 | 41.549129 | 56.901743 | 1.246501e-04 | * | * | * |
| **BA.2.3** | 36275 | 7 | 40.028956 | 35.512910 | 68.974180 | 2.983496e-07 | | * | * |
| **BA.2.7** | 2003 | 7 | 39.678930 | 33.508209 | 72.983583 | 4.017584e-08 | | * | * |

Cumulative Likelihood per-region

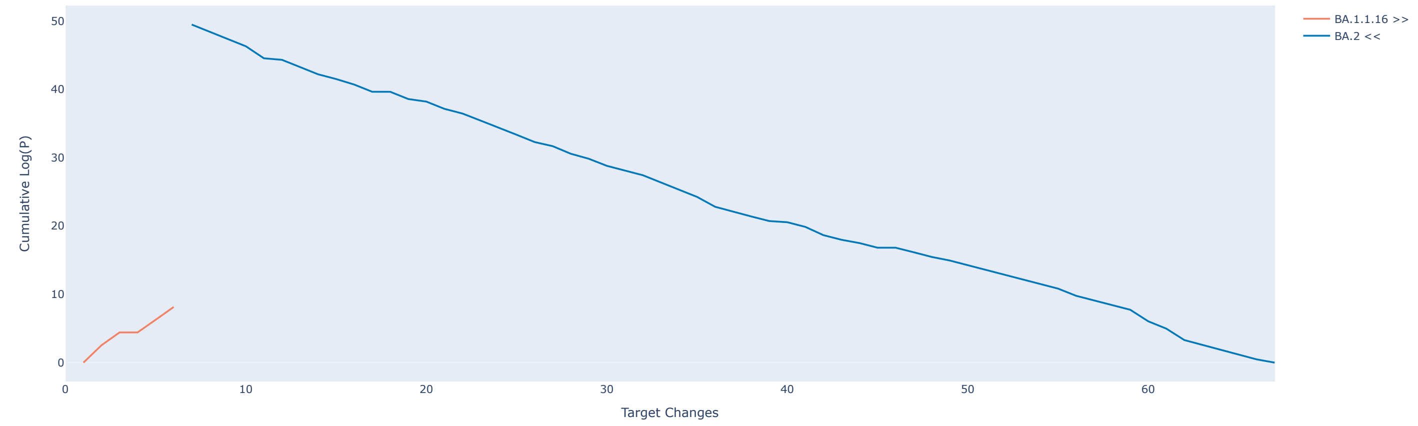


Cumulative Likelihood whole genome

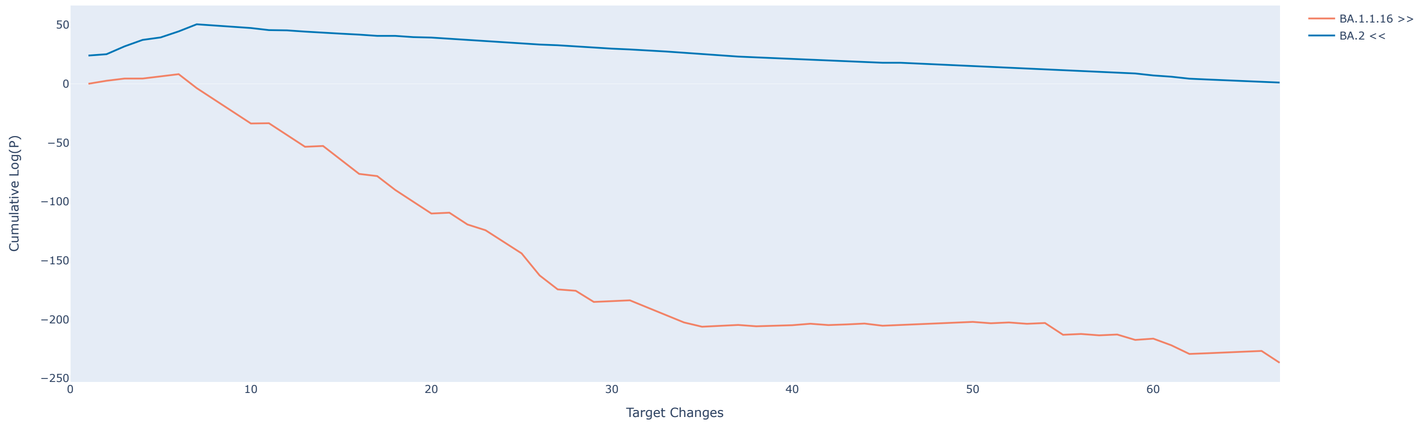


**Target sequence**

, 241_C|T, 2470_C|T, 2832_A|G, 3037_C|T, 5386_T|G, 6513_GTT|..., 9344_C|T, 9424_A|G, 9534_C|T, 9866_C|T, 10029_C|T, 10198_C|T, 10447_G|A, 10449_C|A, 11288_TCTGGTTTT|........., 12880_C|T, 14408_C|T, 15714_C|T, 17010_C|T, 17410_C|T, 18163_A|G, 19955_C|T, 20055_A|G, 21618_C|T, 21633_TACCCCCTG|........., 21987_G|A, 22200_T|G, 22578_G|A, 22674_C|T, 22679_T|C, 22686_C|T, 22688_A|G, 22775_G|A, 22786_A|C, 22792_C|T, 22813_G|T, 22882_T|G, 22992_G|A, 22995_C|A, 23013_A|C, 23040_A|G, 23055_A|G, 23063_A|T, 23075_T|C, 23403_A|G, 23525_C|T, 23599_T|G, 23604_C|A, 23854_C|A, 23948_G|T, 24424_A|T, 24469_T|A, 25000_C|T, 25584_C|T, 26060_C|T, 26270_C|T, 26577_C|G, 26709_G|A, 26858_C|T, 27259_A|C, 27382_GAT|CTC, 27807_C|T, 28271_A|T, 28311_C|T, 28362_GAGAACGCA|........., 28881_GGG|AAC, 29510_A|C

**Case 25 (1BP 5'): XAF** **test: OK**

Target: (75%) 64 samples
Number of changes: 65
GT: BA.1* + BA.2*
GT BR: 8-10
GT BR coord: 10447 - 11288
Rank L1 L2: 11 3
BC: BA.1.1.16 + BA.2.9
BC BR: 9-10
BC BR coord: 10448 - 10449
Direction L1: <<
Initial region span: 1-9,12-65
Gap history (edge excluded): 9-12
Alt. candidates: [BA.1.1, B.1.1.529], []
Model 1BP/2BP comparison: -
Rec. model vs L1: 2.43e-214
Rec. model vs L2: 8.17e-116
Flags: Model_2BP_Bad_L1_opp

## BA.1.1.16 >>

| | num_seq | t_ch_MAX | max_CL | CL@BC_t_ch_MAX | aic | PV | PV_OK | t_ch_MAX_OK | phyl_OK |
|---|---|---|---|---|---|---|---|---|---|
| **BA.1.1.16** | 1583 | 9 | 10.958152 | NaN | NaN | NaN | * | * | * |
| **BA.1.1.2** | 7493 | 9 | 10.949780 | 10.949780 | -1.899560 | 0.990050 | * | * | |
| **BA.1.1.10** | 1083 | 9 | 10.878141 | 10.878141 | -1.756281 | 0.923116 | * | * | |
| **BA.1.1** | 349352 | 9 | 10.852205 | 10.852205 | -1.704411 | 0.895834 | * | * | * |
| **BA.1.1.13** | 3131 | 9 | 10.850903 | 10.850903 | -1.701805 | 0.895834 | * | * | |
| **BA.1.1.14** | 8250 | 9 | 10.842690 | 10.842690 | -1.685380 | 0.891366 | * | * | |
| **BA.1.1.15** | 6410 | 9 | 10.840137 | 10.840137 | -1.680274 | 0.886920 | * | * | |
| **B.1.1.529** | 1110 | 9 | 8.570706 | 8.570706 | 2.858587 | 0.091630 | * | * | * |
| **BA.1.1.18** | 27122 | 6 | 8.174906 | 7.340406 | 5.319188 | 0.026783 | * | | |
| **BA.1.21** | 2247 | 9 | 6.483408 | 6.483408 | 7.033184 | 0.011390 | * | * | |

## BA.2.9 <<

| | num_seq | t_ch_MAX | max_CL | CL@BC_t_ch_MAX | aic | PV | PV_OK | t_ch_MAX_OK | phyl_OK |
|---|---|---|---|---|---|---|---|---|---|
| **BA.2.9** | 108830 | 12 | 52.425502 | NaN | NaN | NaN | * | * | * |
| **BA.2.9.3** | 1075 | 13 | 38.400995 | 27.307878 | 75.384244 | 2.838955e-11 | | * | * |
| **BA.2** | 378689 | 13 | 36.237087 | 31.439498 | 67.121004 | 1.765203e-09 | | * | * |
| **BA.2.9.5** | 1119 | 13 | 36.096265 | 23.579572 | 82.840855 | 6.811458e-13 | | * | * |
| **BA.2.36** | 5393 | 13 | 33.234915 | 21.621106 | 86.757789 | 9.594512e-14 | | * | |
| **BA.2.9.2** | 376 | 13 | 32.000762 | 12.829689 | 104.340622 | 1.460745e-17 | | * | * |
| **BA.2.9.6** | 294 | 13 | 29.930488 | 10.740650 | 108.518700 | 1.806754e-18 | | * | * |
| **BA.2.9.1** | 361 | 20 | 29.544028 | 3.130751 | 123.738499 | 8.951956e-22 | | | * |
| **BA.2.3** | 36275 | 13 | 26.858210 | 14.832007 | 100.335986 | 1.079353e-16 | | * | |
| **BA.2.29** | 1418 | 13 | 23.952732 | 12.295908 | 105.408183 | 8.555135e-18 | | * | |

Cumulative Likelihood per-region

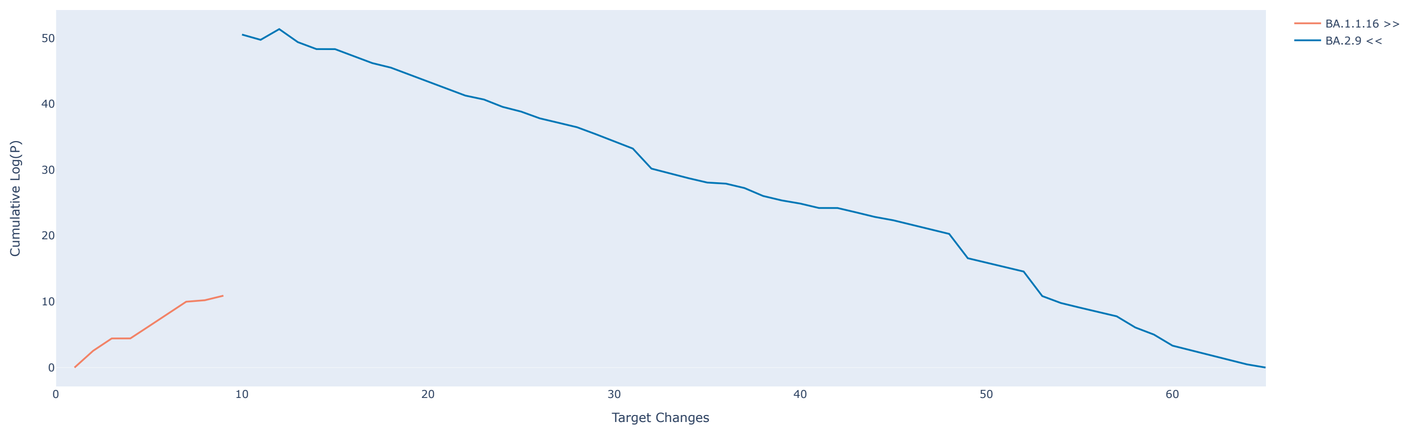


Cumulative Likelihood whole genome

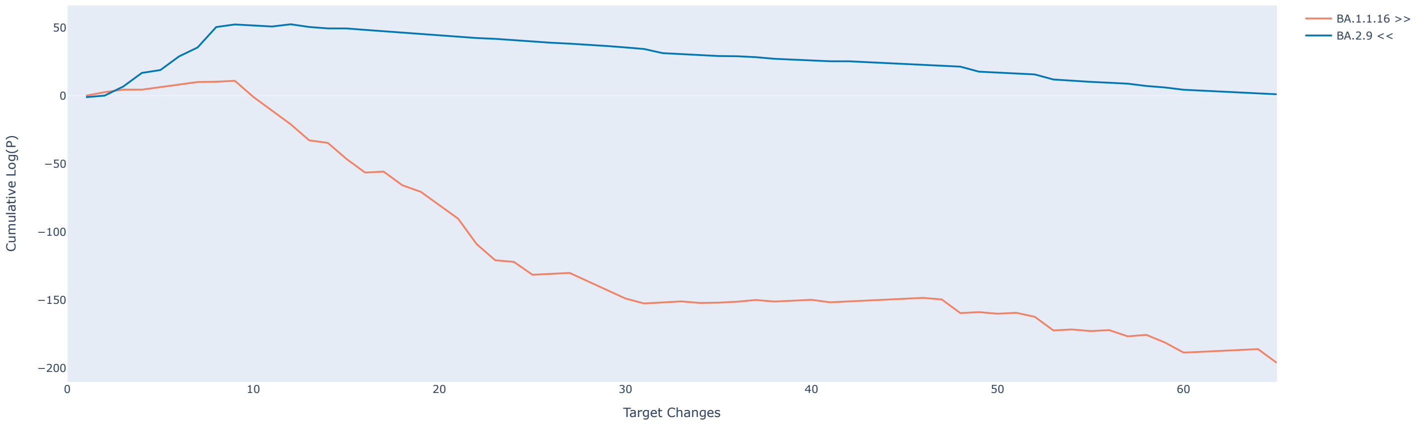


**Target sequence**

, 241_C|T, 2470_C|T, 2832_A|G, 3037_C|T, 5386_T|G, 6513_GTT|..., 8393_G|A, 10029_C|T, 10449_C|A, 11288_TCTGGTTTT|........., 11514_C|T, 11516_G|T, 12880_C|T, 14408_C|T, 15714_C|T, 17410_C|T, 18163_A|G, 19955_C|T, 20055_A|G, 21618_C|T, 21633_TACCCCCTG|........., 21987_G|A, 22200_T|G, 22578_G|A, 22674_C|T, 22679_T|C, 22686_C|T, 22688_A|G, 22775_G|A, 22786_A|C, 22792_C|T, 22813_G|T, 22882_T|G, 22992_G|A, 22995_C|A, 23013_A|C, 23040_A|G, 23055_A|G, 23063_A|T, 23075_T|C, 23403_A|G, 23525_C|T, 23599_T|G, 23604_C|A, 23854_C|A, 23948_G|T, 24424_A|T, 24433_A|G, 24469_T|A, 25000_C|T, 25584_C|T, 25624_C|T, 26060_C|T, 26270_C|T, 26577_C|G, 26709_G|A, 26858_C|T, 27259_A|C, 27382_GAT|CTC, 27807_C|T, 28271_A|T, 28311_C|T, 28362_GAGAACGCA|........., 28881_GGG|AAC, 29510_A|C

**Case 26 (1BP 5'): XAG** **test: OK**

Target: (75%) 227 samples
Number of changes: 70
GT: BA.1* + BA.2*
GT BR: 8–9
GT BR coord: 6515 – 8393
Rank L1 L2: 11 1
BC: BA.1.1.14 + BA.2
BC BR: 6–7
BC BR coord: 5387 – 5388
Direction L1: <<
Initial region span: 1–6,9–70
Gap history (edge excluded): 6–9
Alt. candidates: [BA.1.1], [BA.2.9]
Model 1BP/2BP comparison: –
Rec. model vs L1: 3.13e−192
Rec. model vs L2: 1.33e−36
Flags: Model_2BP_Bad_L1_opp

## BA.1.1.14 >>

| | num_seq | t_ch_MAX | max_CL | CL@BC_t_ch_MAX | aic | PV | PV_OK | t_ch_MAX_OK | phyl_OK |
|---|---|---|---|---|---|---|---|---|---|
| **BA.1.1.14** | 8250 | 6 | 10.679082 | NaN | NaN | NaN | * | * | * |
| **BA.1.1.10** | 1083 | 6 | 7.459769 | 7.459769 | 1.080462 | 3.995506e-02 | * | * | |
| **BA.1.1** | 349352 | 6 | 5.664660 | 5.664660 | 4.670680 | 6.637632e-03 | * | * | * |
| **BA.1.1.2** | 7493 | 3 | 4.471676 | -3.677852 | 23.355704 | 5.801379e-07 | | | |
| **BA.1.1.16** | 1583 | 3 | 4.471013 | -3.676136 | 23.352272 | 5.830459e-07 | | | |
| **BA.1.1.18** | 27122 | 3 | 4.459549 | -3.687623 | 23.375245 | 5.743654e-07 | | | |
| **BA.1.1.6** | 86 | 3 | 4.452918 | -9.429814 | 34.859628 | 1.846452e-09 | | | |
| **BA.1.1.12** | 1392 | 3 | 4.430911 | -11.740712 | 39.481424 | 1.832811e-10 | | | |
| **BA.1.1.15** | 6410 | 3 | 4.428521 | -3.721578 | 23.443155 | 5.573904e-07 | | | |
| **BA.1.1.13** | 3131 | 3 | 4.428414 | -3.717159 | 23.434318 | 5.601843e-07 | | | |

## BA.2 <<

| | num_seq | t_ch_MAX | max_CL | CL@BC_t_ch_MAX | aic | PV | PV_OK | t_ch_MAX_OK | phyl_OK |
|---|---|---|---|---|---|---|---|---|---|
| **BA.2** | 378689 | 9 | 48.297172 | NaN | NaN | NaN | * | * | * |
| **BA.2.9** | 108830 | 9 | 44.544281 | 35.723054 | 76.553891 | 9.419248e-03 | * | * | * |
| **BA.2.9.5** | 1119 | 19 | 42.534699 | 21.416748 | 105.166505 | 5.744632e-09 | | | * |
| **BA.2.81** | 436 | 20 | 42.427978 | 5.896300 | 136.207401 | 1.044749e-15 | | | * |
| **BA.2.7** | 2003 | 19 | 40.582365 | 17.575488 | 112.849024 | 1.234728e-10 | | | * |
| **BA.2.69** | 96 | 24 | 39.510309 | 0.777695 | 146.444610 | 6.274738e-18 | | | * |
| **BA.2.25** | 331 | 24 | 39.090732 | 11.076658 | 125.846684 | 1.856339e-13 | | | * |
| **BA.2.37** | 2257 | 9 | 35.978871 | 15.978871 | 116.042258 | 2.505369e-11 | | * | * |
| **BA.2.1** | 3218 | 19 | 35.929166 | 9.618846 | 128.762307 | 4.332702e-14 | | | * |
| **BA.2.71** | 409 | 29 | 34.072260 | 3.292597 | 141.414805 | 7.759723e-17 | | | * |

Cumulative Likelihood per-region

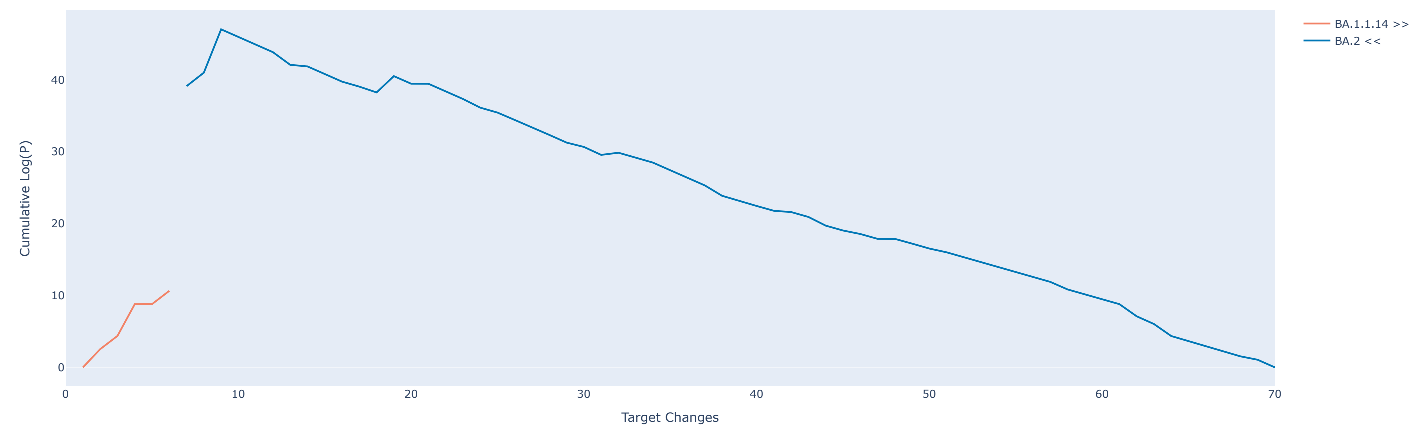


Cumulative Likelihood whole genome

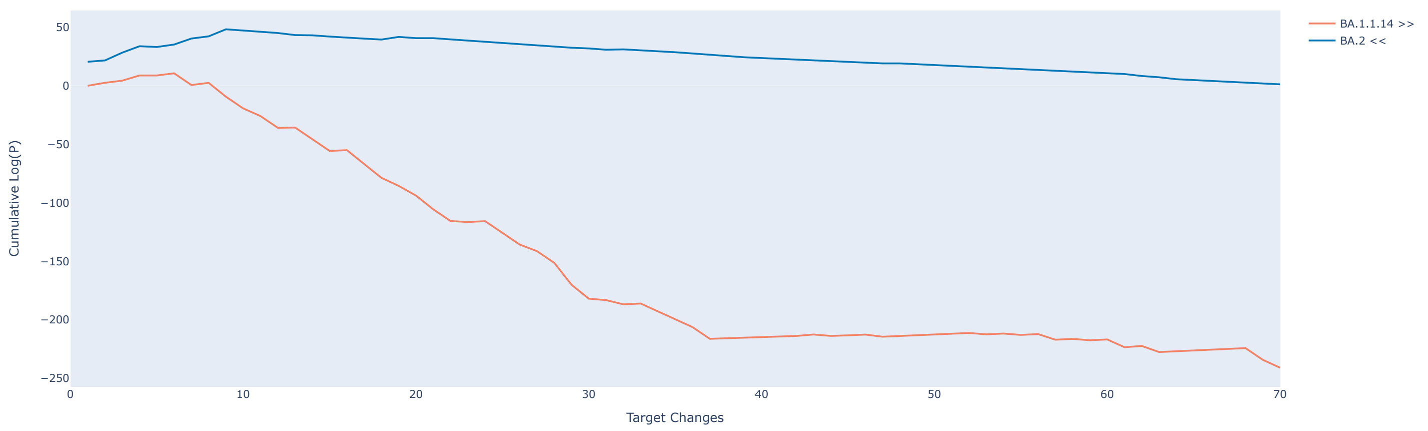


**Target sequence**

, 241_C|T, 2470_C|T, 2832_A|G, 2857_C|T, 3037_C|T, 5386_T|G, 5585_C|A, 6513_GTT|..., 9344_C|T, 9424_A|G, 9534_C|T, 9866_C|T, 10029_C|T, 10198_C|T, 10447_G|A, 10449_C|A, 11288_TCTGGTTTT|........., 12334_A|G, 12880_C|T, 14408_C|T, 15714_C|T, 17410_C|T, 17502_C|T, 18163_A|G, 19955_C|T, 20055_A|G, 21618_C|T, 21633_TACCCCCTG|........., 21987_G|A, 22200_T|G, 22578_G|A, 22679_T|C, 22686_C|T, 22688_A|G, 22775_G|A, 22786_A|C, 22792_C|T, 22813_G|T, 22882_T|G, 22992_G|A, 22995_C|A, 23013_A|C, 23040_A|G, 23055_A|G, 23063_A|T, 23075_T|C, 23403_A|G, 23525_C|T, 23599_T|G, 23604_C|A, 23854_C|A, 23948_G|T, 24424_A|T, 24469_T|A, 25000_C|T, 25584_C|T, 26060_C|T, 26270_C|T, 26577_C|G, 26709_G|A, 26858_C|T, 27259_A|C, 27382_GAT|CTC, 27807_C|T, 28271_A|T, 28311_C|T, 28362_GAGAACGCA|........., 28881_GGG|AAC, 29510_A|C, 29734_GAGGCCACGCGGAGTACGATCGAGTG|..........................

**Case 27 (1BP 5'): XAM**    **test: OK**

Target: (75%) 147 samples
GT: BA.1.1 + BA.2.9
BC: BA.1.1.16 + BA.2.9
Direction L1: <<
Alt. candidates: [BA.1.1, B.1.1.529], []
Model 1BP/2BP comparison:
Rec. model vs L1: 2.55e-269
Flags: Model_2BP_Bad_L1_opp

Number of changes: 69
GT BR: 6-7
BC BR: 6-7
Initial region span: 1-6,7-69
-
Rec. model vs L2: 4.72e-55

GT BR coord: 8087 - 9192
BC BR coord: 6512 - 6513
Gap history (edge excluded):

Rank L1 L2: 5 1

## BA.1.1.16 >>

| | num_seq | t_ch_MAX | max_CL | CL@BC_t_ch_MAX | aic | PV | PV_OK | t_ch_MAX_OK | phyl_OK |
|---|---|---|---|---|---|---|---|---|---|
| **BA.1.1.16** | 1583 | 6 | 8.195140 | NaN | NaN | NaN | * | * | * |
| **BA.1.1.2** | 7493 | 6 | 8.185254 | 8.185254 | -2.370508 | 0.990050 | * | * | |
| **BA.1.1.18** | 27122 | 6 | 8.174906 | 8.174906 | -2.349812 | 0.980199 | * | * | |
| **BA.1.1.10** | 1083 | 6 | 8.144769 | 8.144769 | -2.289537 | 0.951229 | * | * | |
| **BA.1.1** | 349352 | 6 | 8.125137 | 8.125137 | -2.250274 | 0.932394 | * | * | * |
| **BA.1.1.14** | 8250 | 6 | 8.102711 | 8.102711 | -2.205422 | 0.913931 | * | * | |
| **BA.1.1.13** | 3131 | 6 | 8.091567 | 8.091567 | -2.183133 | 0.900325 | * | * | |
| **BA.1.1.15** | 6410 | 6 | 8.089843 | 8.089843 | -2.179686 | 0.900325 | * | * | |
| **B.1.1.529** | 1110 | 6 | 6.113152 | 6.113152 | 1.773696 | 0.124930 | * | * | * |
| **BA.1.1.6** | 86 | 4 | 4.458730 | 2.449708 | 9.100583 | 0.003199 | * | | |

## BA.2.9 <<

| | num_seq | t_ch_MAX | max_CL | CL@BC_t_ch_MAX | aic | PV | PV_OK | t_ch_MAX_OK | phyl_OK |
|---|---|---|---|---|---|---|---|---|---|
| **BA.2.9** | 108830 | 7 | 57.628233 | NaN | NaN | NaN | * | * | * |
| **BA.2.9.3** | 1075 | 7 | 45.365051 | 45.365051 | 55.269899 | 4.713876e-06 | | * | * |
| **BA.2** | 378689 | 7 | 42.221966 | 42.221966 | 61.556068 | 2.030122e-07 | | * | * |
| **BA.2.9.5** | 1119 | 7 | 41.628256 | 41.628256 | 62.743489 | 1.125352e-07 | | * | * |
| **BA.2.36** | 5393 | 7 | 39.636409 | 39.636409 | 66.727183 | 1.530632e-08 | | * | |
| **BA.2.3** | 36275 | 7 | 32.814936 | 28.298890 | 89.402221 | 1.828702e-13 | | * | |
| **BA.2.9.2** | 376 | 17 | 32.000762 | 30.904641 | 84.190717 | 2.474457e-12 | | | * |
| **BA.2.29** | 1418 | 7 | 30.340762 | 30.340762 | 85.318477 | 1.406383e-12 | | * | |
| **BA.2.9.6** | 294 | 17 | 29.930488 | 28.687914 | 88.624172 | 2.700957e-13 | | | * |
| **BA.2.9.1** | 361 | 24 | 29.544028 | 21.196557 | 103.606886 | 1.501345e-16 | | | * |

Cumulative Likelihood per-region

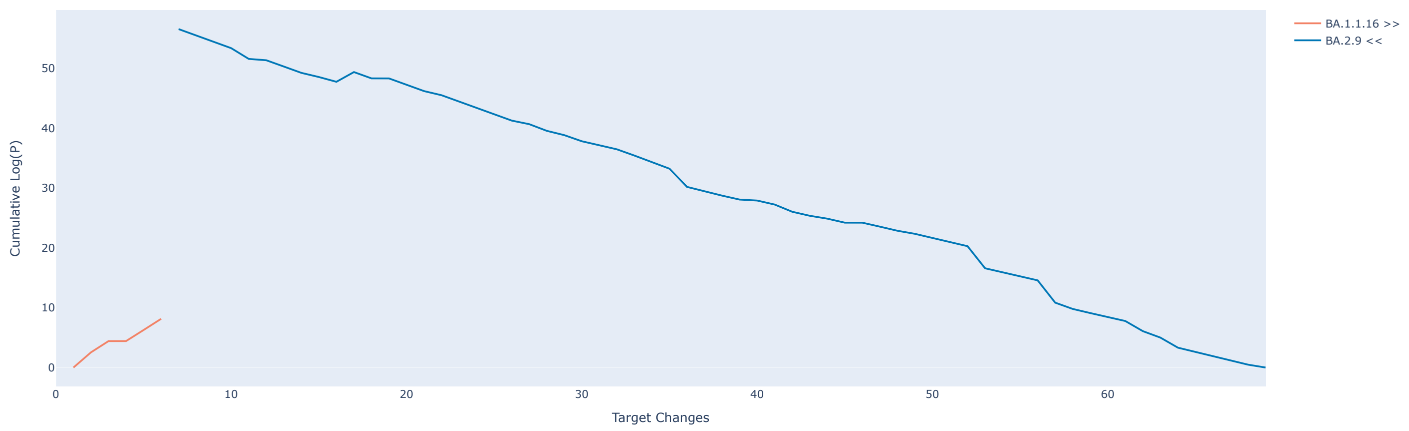


Cumulative Likelihood whole genome

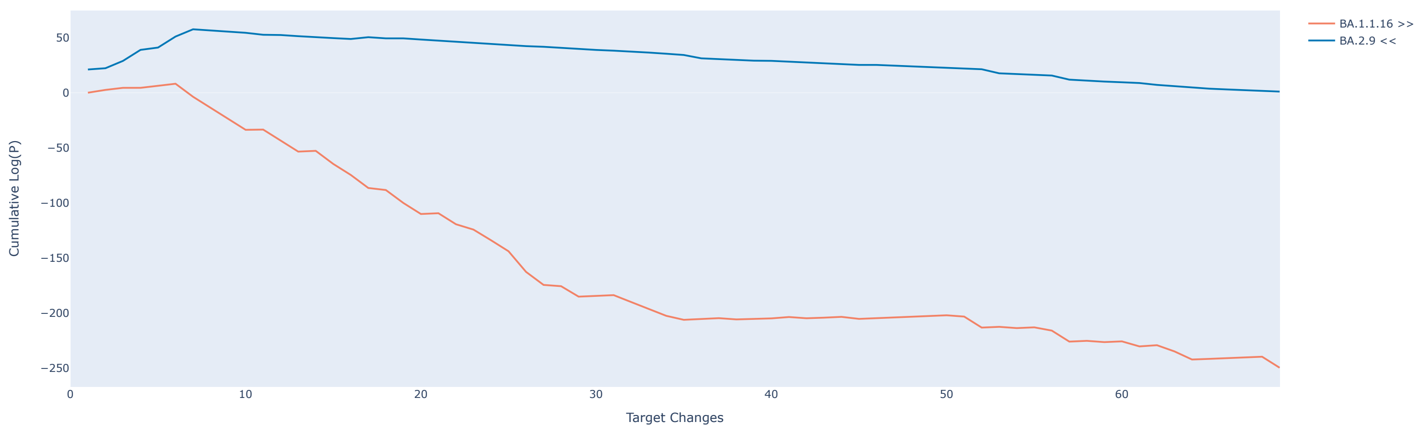


**Target sequence**

, 241_C|T, 2470_C|T, 2832_A|G, 3037_C|T, 5386_T|G, 6513_GTT|..., 9344_C|T, 9424_A|G, 9534_C|T, 9866_C|T, 10029_C|T, 10198_C|T, 10447_G|A, 10449_C|A, 11288_TCTGGTTTT|........., 11514_C|T, 12880_C|T, 14408_C|T, 15714_C|T, 17410_C|T, 18163_A|G, 19955_C|T, 20055_A|G, 21618_C|T, 21633_TACCCCCTG|........., 21987_G|A, 22200_T|G, 22578_G|A, 22674_C|T, 22679_T|C, 22686_C|T, 22688_A|G, 22775_G|A, 22786_A|C, 22792_C|T, 22813_G|T, 22882_T|G, 22992_G|A, 22995_C|A, 23013_A|C, 23040_A|G, 23055_A|G, 23063_A|T, 23075_T|C, 23403_A|G, 23525_C|T, 23599_T|G, 23604_C|A, 23854_C|A, 23948_G|T, 24424_A|T, 24433_A|G, 24469_T|A, 25000_C|T, 25584_C|T, 25624_C|T, 26060_C|T, 26270_C|T, 26577_C|G, 26709_G|A, 26858_C|T, 27259_A|C, 27382_GAT|CTC, 27807_C|T, 28271_A|T, 28311_C|T, 28362_GAGAACGCA|........., 28881_GGG|AAC, 29510_A|C

**Case 28 (1BP 5'): XAR** **test: KO**

Target: (75%) 12 samples
GT: BA.1* + BA.2*
BC: BA.2
Direction L1: <<
Alt. candidates: [BA.2.10]
Model 1BP/2BP comparison: –
Rec. model vs L1: –
Flags: NotEnoughSpaceAfterL1, SingleCandidateGenome

Number of changes: 64
GT BR: 1–3
BC BR:
Initial region span: 2–64
Rec. model vs L2: –

GT BR coord: 2833 – 4184
BC BR coord:
Gap history (edge excluded):

Rank L1 L2: 11 –

## BA.2 <<

| | num_seq | t_ch_MAX | max_CL | CL@BC_t_ch_MAX | aic | PV | PV_OK | t_ch_MAX_OK | phyl_OK |
|---|---|---|---|---|---|---|---|---|---|
| **BA.2** | 378689 | 2 | 50.565165 | NaN | NaN | NaN | * | * | * |
| **BA.2.37** | 2257 | 5 | 46.914125 | 29.282294 | 103.435411 | 1.078435e-06 | | | * |
| **BA.2.10** | 28641 | 2 | 45.750688 | 36.898300 | 88.203400 | 2.198456e-03 | * | * | * |
| **BA.2.3** | 36275 | 5 | 43.763269 | 33.970321 | 94.059359 | 1.173910e-04 | * | | * |
| **BA.2.9** | 108830 | 2 | 42.440214 | 30.395057 | 101.209886 | 3.288760e-06 | | * | * |
| **BA.2.23** | 3557 | 2 | 41.440215 | 23.311004 | 115.377993 | 2.754582e-09 | | * | * |
| **BA.2.29** | 1418 | 2 | 41.083370 | 20.819526 | 120.360948 | 2.283823e-10 | | * | * |
| **BA.2.5** | 1908 | 3 | 40.620877 | 21.495835 | 119.008330 | 4.485504e-10 | | * | * |
| **BA.2.27** | 215 | 3 | 40.001516 | 18.702153 | 124.595694 | 2.741310e-11 | | * | * |
| **BA.2.33** | 389 | 5 | 39.996156 | 17.327281 | 127.345438 | 6.931118e-12 | | | * |

Cumulative Likelihood per-region

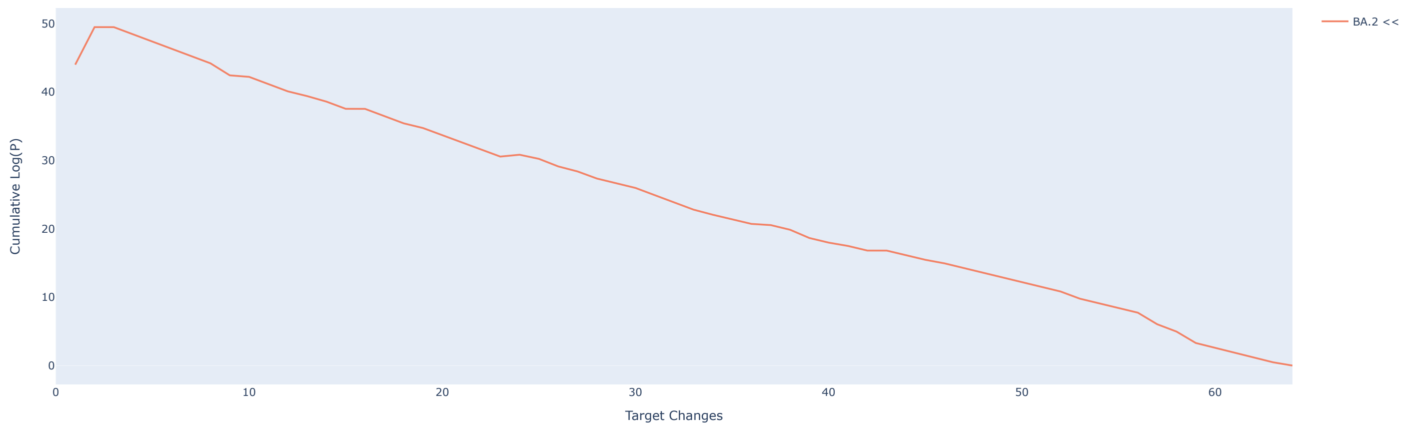


Cumulative Likelihood whole genome

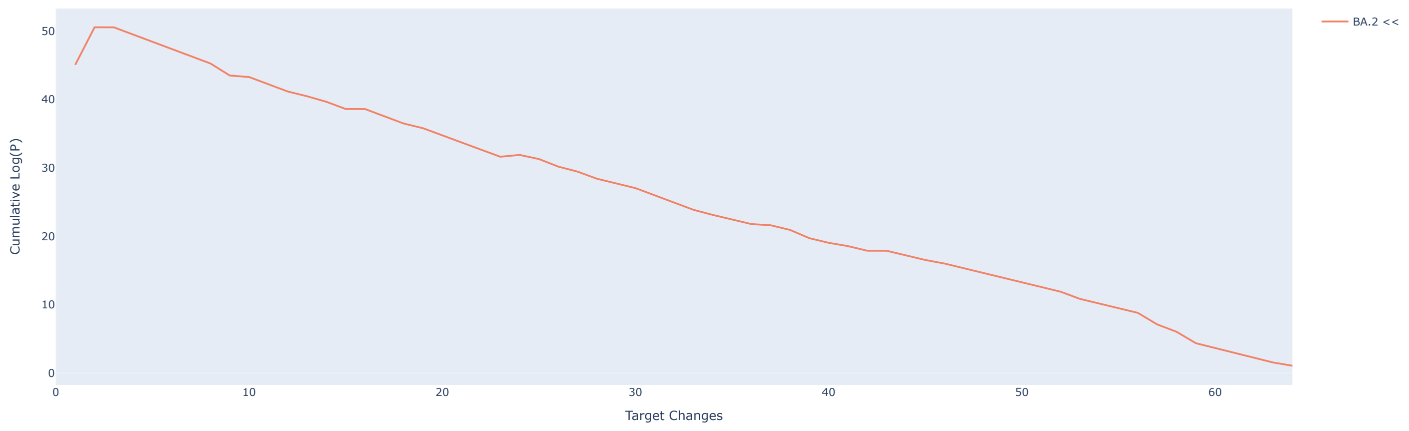


**Target sequence**

, 2832_A|G, 3037_C|T, 4184_G|A, 4321_C|T, 9344_C|T, 9424_A|G, 9534_C|T, 9866_C|T, 10029_C|T, 10198_C|T, 10447_G|A, 10449_C|A, 11288_TCTGGTTTT|........., 12880_C|T, 14408_C|T, 15714_C|T, 17410_C|T, 18163_A|G, 19955_C|T, 20055_A|G, 21618_C|T, 21633_TACCCCCTG|........., 21796_G|T, 21987_G|A, 22200_T|G, 22578_G|A, 22674_C|T, 22679_T|C, 22686_C|T, 22688_A|G, 22775_G|A, 22786_A|C, 22813_G|T, 22882_T|G, 22992_G|A, 22995_C|A, 23013_A|C, 23040_A|G, 23055_A|G, 23063_A|T, 23075_T|C, 23403_A|G, 23525_C|T, 23599_T|G, 23604_C|A, 23854_C|A, 23948_G|T, 24424_A|T, 24469_T|A, 25000_C|T, 25584_C|T, 26060_C|T, 26270_C|T, 26577_C|G, 26709_G|A, 26858_C|T, 27259_A|C, 27382_GAT|CTC, 27807_C|T, 28271_A|T, 28311_C|T, 28362_GAGAACGCA|........., 28881_GGG|AAC, 29510_A|C

## Case 29 (1BP 5'): XAU        test: OK

Target: (75%) 72 samples
GT: BA.1.1* + BA.2.9*
BC: BA.1.1.2 + BA.2.9
Direction L1: <<
Alt. candidates: [BA.1.1], []
Model 1BP/2BP comparison: –
Rec. model vs L1: 3.37e-242
Flags: Model_2BP_Bad_L1_opp

Number of changes: 69
GT BR: 3–5
BC BR: 3–4
Initial region span: 1–3,4–69
Rec. model vs L2: 2.51e-25

GT BR coord: 2834 – 4184
BC BR coord: 2835 – 2836
Gap history (edge excluded):

Rank L1 L2: 9 1

## BA.1.1.2 >>

| | num_seq | t_ch_MAX | max_CL | CL@BC_t_ch_MAX | aic | PV | PV_OK | t_ch_MAX_OK | phyl_OK |
|---|---|---|---|---|---|---|---|---|---|
| **BA.1.1.2** | 7493 | 3 | 4.471676 | NaN | NaN | NaN | * | * | * |
| **BA.1.1.16** | 1583 | 3 | 4.471013 | 4.471013 | -2.942027 | 1.000000 | * | * | |
| **BA.1.1.18** | 27122 | 3 | 4.459549 | 4.459549 | -2.919098 | 0.990050 | * | * | |
| **BA.1.1.6** | 86 | 3 | 4.452918 | 4.452918 | -2.905835 | 0.985112 | * | * | |
| **BA.1.1.12** | 1392 | 3 | 4.430911 | 4.430911 | -2.861823 | 0.960789 | * | * | |
| **BA.1.1.15** | 6410 | 3 | 4.428521 | 4.428521 | -2.857042 | 0.960789 | * | * | |
| **BA.1.1.13** | 3131 | 3 | 4.428414 | 4.428414 | -2.856828 | 0.960789 | * | * | |
| **BA.1.1.10** | 1083 | 3 | 4.427245 | 4.427245 | -2.854491 | 0.955997 | * | * | |
| **BA.1.1** | 349352 | 3 | 4.422482 | 4.422482 | -2.844964 | 0.951229 | * | * | * |
| **BA.1.1.14** | 8250 | 3 | 4.409831 | 4.409831 | -2.819662 | 0.941765 | * | * | |

## BA.2.9 <<

| | num_seq | t_ch_MAX | max_CL | CL@BC_t_ch_MAX | aic | PV | PV_OK | t_ch_MAX_OK | phyl_OK |
|---|---|---|---|---|---|---|---|---|---|
| **BA.2.9** | 108830 | 4 | 60.888095 | NaN | NaN | NaN | * | * | * |
| **BA.2** | 378689 | 4 | 44.601801 | 44.601801 | 72.796399 | 8.420597e-08 | | * | * |
| **BA.2.9.3** | 1075 | 4 | 39.417976 | 39.417976 | 83.164048 | 4.739117e-10 | | * | * |
| **BA.2.9.5** | 1119 | 7 | 34.978314 | 28.705499 | 104.589002 | 1.052524e-14 | | | * |
| **BA.2.36** | 5393 | 4 | 34.176356 | 34.176356 | 93.647289 | 2.499326e-12 | | * | |
| **BA.2.3** | 36275 | 7 | 33.965507 | 31.568338 | 98.863324 | 1.847080e-13 | | | |
| **BA.2.9.2** | 376 | 7 | 30.904641 | 27.081919 | 107.836163 | 2.072543e-15 | | | * |
| **BA.2.9.1** | 361 | 24 | 29.544028 | 6.711722 | 148.576555 | 2.950699e-24 | | | * |
| **BA.2.9.6** | 294 | 7 | 28.687914 | 26.497559 | 109.004881 | 1.160413e-15 | | | * |
| **BA.2.29** | 1418 | 4 | 24.958232 | 24.958232 | 112.083537 | 2.487707e-16 | | * | |

Cumulative Likelihood per-region

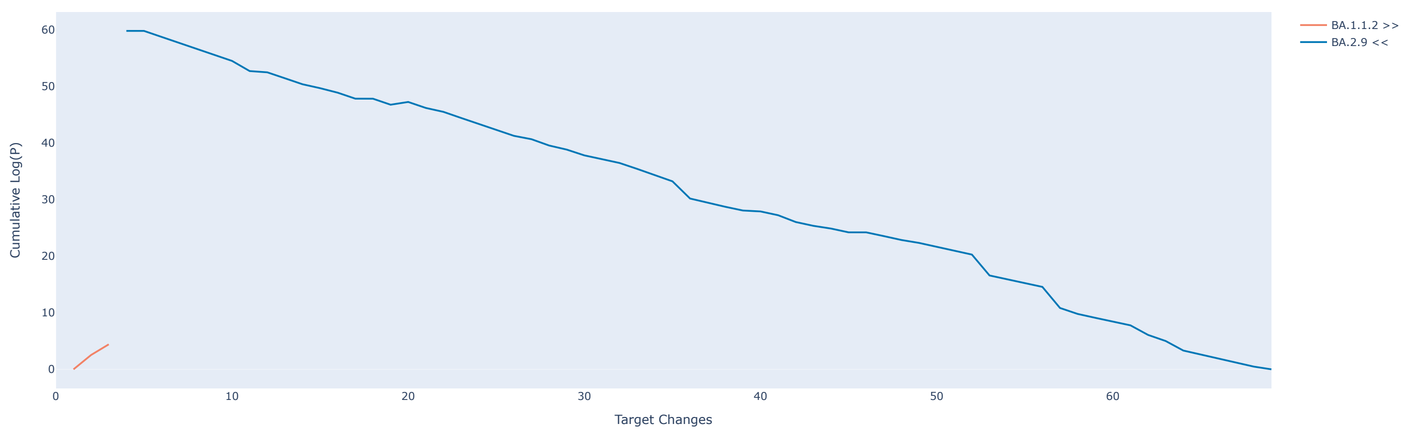


Cumulative Likelihood whole genome

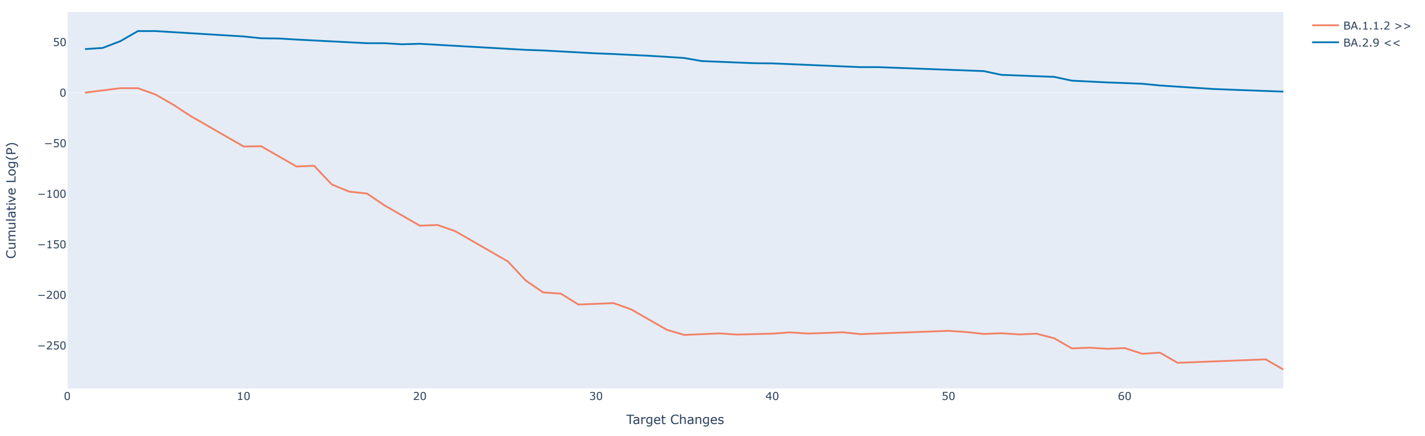


**Target sequence**

, 241_C|T, 2470_C|T, 2832_A|G, 3037_C|T, 4184_G|A, 4321_C|T, 9344_C|T, 9424_A|G, 9534_C|T, 9866_C|T, 10029_C|T, 10198_C|T, 10447_G|A, 10449_C|A, 11288_TCTGGTTTT|........., 12880_C|T, 14408_C|T, 15714_C|T, 17407_T|C, 17410_C|T, 18163_A|G, 19955_C|T, 20055_A|G, 21618_C|T, 21633_TACCCCCTG|........., 21987_G|A, 22200_T|G, 22578_G|A, 22674_C|T, 22679_T|C, 22686_C|T, 22688_A|G, 22775_G|A, 22786_A|C, 22792_C|T, 22813_G|T, 22882_T|G, 22992_G|A, 22995_C|A, 23013_A|C, 23040_A|G, 23055_A|G, 23063_A|T, 23075_T|C, 23403_A|G, 23525_C|T, 23599_T|G, 23604_C|A, 23854_C|A, 23948_G|T, 24424_A|T, 24433_A|G, 24469_T|A, 25000_C|T, 25584_C|T, 25624_C|T, 26060_C|T, 26270_C|T, 26577_C|G, 26709_G|A, 26858_C|T, 27259_A|C, 27382_GAT|CTC, 27807_C|T, 28271_A|T, 28311_C|T, 28362_GAGAACGCA|........., 28881_GGG|AAC, 29510_A|C

## Case 30 (1BP 5'): XE    test: OK

Target: (75%) 1009 samples
GT: BA.1* + BA.2*
BC: BA.1.17.2 + BA.2.29
Direction L1: <<
Alt. candidates: [BD.1, BA.1.17, B.1.1.529], [BA.2]
Model 1BP/2BP comparison:
Rec. model vs L1: 5.48e−227
Flags: Model_2BP_Bad_L1_opp

Number of changes: 63
GT BR: 9−11
BC BR: 9−10
Initial region span: 1−9,10−63
−
Rec. model vs L2: 2.70e−106

GT BR coord: 10447 − 11288
BC BR coord: 10447 − 10448
Gap history (edge excluded):

Rank L1 L2: 11 2

## BA.1.17.2 >>

| | num_seq | t_ch_MAX | max_CL | CL@BC_t_ch_MAX | aic | PV | PV_OK | t_ch_MAX_OK | phyl_OK |
|---|---|---|---|---|---|---|---|---|---|
| **BA.1.17.2** | 77688 | 9 | 15.693638 | NaN | NaN | NaN | * | * | * |
| **BD.1** | 2033 | 9 | 15.589280 | 15.589280 | -9.178560 | 9.003245e-01 | * | * | * |
| **BA.1.17** | 27172 | 9 | 11.738266 | 11.738266 | -1.476532 | 1.915867e-02 | * | * | * |
| **B.1.1.529** | 1110 | 9 | 8.963077 | 8.963077 | 4.073846 | 1.194533e-03 | * | * | * |
| **BA.1.12** | 726 | 9 | 2.484307 | 2.484307 | 17.031386 | 1.832187e-06 | | * | |
| **BA.1.1.4** | 865 | 5 | 2.205344 | -3.895162 | 29.790325 | 3.105783e-09 | | | |
| **BC.2** | 99 | 3 | 1.952310 | -12.242171 | 46.484343 | 7.378764e-13 | | | |
| **BA.1.7** | 220 | 3 | 1.952310 | -12.242171 | 46.484343 | 7.378764e-13 | | | |
| **BA.1.4** | 266 | 3 | 1.952310 | -12.257280 | 46.514560 | 7.268909e-13 | | | |
| **BA.1.3** | 17 | 3 | 1.952310 | -28.971585 | 79.943169 | 4.001630e-20 | | | |

## BA.2.29 <<

| | num_seq | t_ch_MAX | max_CL | CL@BC_t_ch_MAX | aic | PV | PV_OK | t_ch_MAX_OK | phyl_OK |
|---|---|---|---|---|---|---|---|---|---|
| **BA.2.29** | 1418 | 10 | 41.760456 | NaN | NaN | NaN | * | * | * |
| **BA.2** | 378689 | 10 | 41.609934 | 41.609934 | 32.780133 | 8.607080e-01 | * | * | * |
| **BA.2.31** | 1389 | 12 | 41.102602 | 35.754033 | 44.491935 | 2.466389e-03 | * | | |
| **BA.2.1** | 3218 | 15 | 40.697119 | 28.076568 | 59.846865 | 1.139410e-06 | | | |
| **BA.2.23** | 3557 | 15 | 40.680630 | 39.876896 | 36.246209 | 1.518291e-01 | * | | |
| **BA.2.27** | 215 | 15 | 40.591808 | 33.155113 | 49.689773 | 1.831876e-04 | * | | |
| **BA.2.26** | 490 | 15 | 40.265298 | 32.837058 | 50.325884 | 1.330215e-04 | * | | |
| **BA.2.5** | 1908 | 15 | 39.516592 | 39.375443 | 37.249113 | 9.208898e-02 | * | | |
| **BA.2.11** | 598 | 15 | 39.039884 | 31.621167 | 52.757666 | 3.946880e-05 | * | | |
| **BA.2.19** | 365 | 16 | 38.782715 | 25.871105 | 64.257789 | 1.256205e-07 | | | |

Cumulative Likelihood per-region

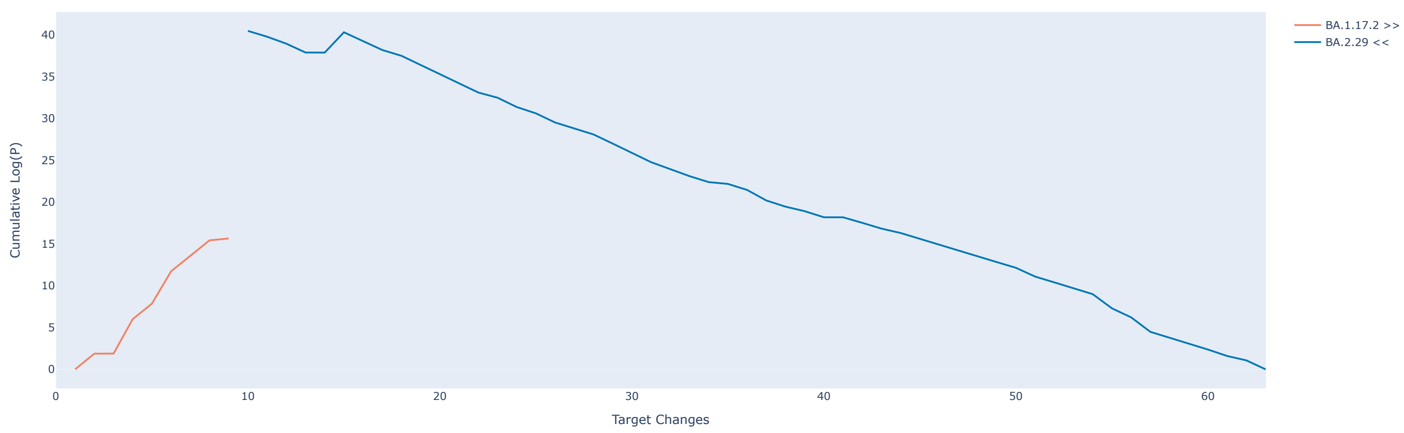


Cumulative Likelihood whole genome

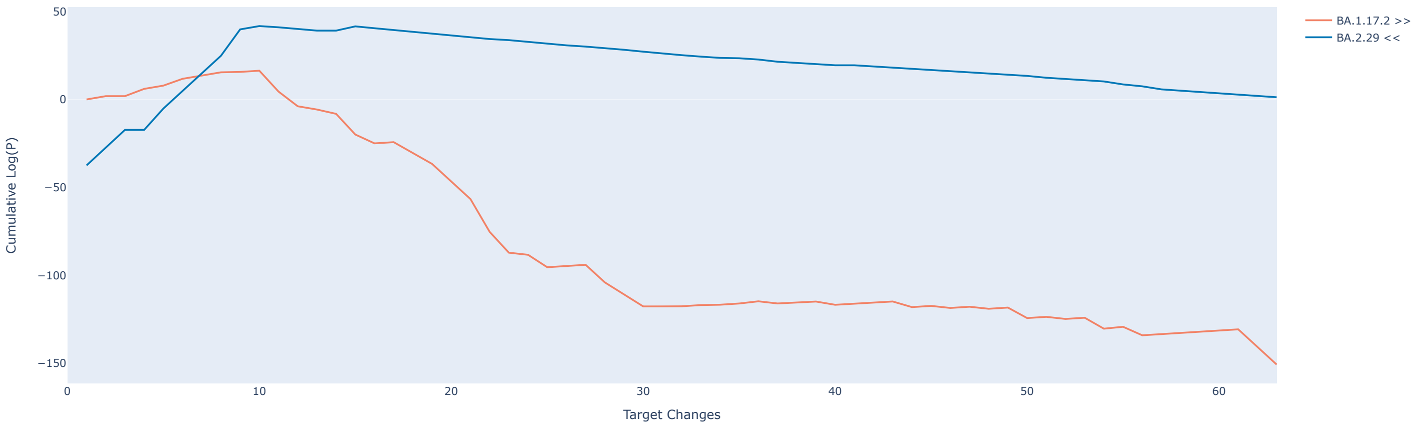


**Target sequence**

, 241_C|T, 2832_A|G, 3037_C|T, 3241_C|T, 5386_T|G, 5924_G|A, 6513_GTT|..., 8393_G|A, 10029_C|T, 10449_C|A, 11288_TCTGGTTTT|........., 12880_C|T, 14408_C|T, 14599_C|T, 15714_C|T, 17410_C|T, 18163_A|G, 19955_C|T, 20055_A|G, 21618_C|T, 21633_TACCCCCTG|........., 21987_G|A, 22200_T|G, 22578_G|A, 22674_C|T, 22679_T|C, 22686_C|T, 22688_A|G, 22775_G|A, 22786_A|C, 22813_G|T, 22882_T|G, 22992_G|A, 22995_C|A, 23013_A|C, 23040_A|G, 23055_A|G, 23063_A|T, 23075_T|C, 23403_A|G, 23525_C|T, 23599_T|G, 23604_C|A, 23854_C|A, 23948_G|T, 24424_A|T, 24469_T|A, 25000_C|T, 25584_C|T, 26060_C|T, 26270_C|T, 26577_C|G, 26709_G|A, 26858_C|T, 27259_A|C, 27382_GAT|CTC, 27807_C|T, 28271_A|T, 28311_C|T, 28362_GAGAACGCA|........., 28881_GGG|AAC, 29510_A|C, 29734_GAGGCCACGCGGAGTACGATCGAGTG|..........................

## Case 31 (1BP 5'): XF                    test: OK

Target: (75%) 12 samples
GT: B.1.617.2* + BA.1*
BC: AY.37 + BA.1.16
Direction L1: <<
Alt. candidates: [AY.3], [BA.1, BA.1.1]
Model 1BP/2BP comparison: -
Rec. model vs L1: 0.00e+00
Flags: Model_2BP_Bad_L1_opp

Number of changes: 62
GT BR: 6-7
BC BR: 6-7
Initial region span: 1-3,7-62
Rec. model vs L2: 2.96e-21

GT BR coord: 5385 - 6512
BC BR coord: 4183 - 4184
Gap history (edge excluded): 3-7

Rank L1 L2: 11 2

## AY.37 >>

| | num_seq | t_ch_MAX | max_CL | CL@BC_t_ch_MAX | aic | PV | PV_OK | t_ch_MAX_OK | phyl_OK |
|---|---|---|---|---|---|---|---|---|---|
| **AY.37** | 992 | 3 | 5.007771 | NaN | NaN | NaN | * | * | * |
| **AY.4.2** | 29310 | 3 | 3.984537 | -4.773105 | 29.546210 | 1.528550e+08 | * | * | |
| **AY.32** | 1205 | 3 | 3.448173 | -5.318218 | 30.636435 | 8.863169e+07 | * | * | |
| **AY.3** | 78123 | 3 | 3.343999 | -5.424712 | 30.849423 | 7.979728e+07 | * | * | * |
| **AY.4.2.1** | 7291 | 3 | 3.021518 | -5.743818 | 31.487635 | 5.794472e+07 | * | * | |
| **AY.23** | 7434 | 3 | 2.994539 | -11.806022 | 43.612044 | 1.352662e+05 | * | * | |
| **AY.117** | 7391 | 3 | 2.711689 | -12.514964 | 45.029928 | 6.650284e+04 | * | * | |
| **AY.102** | 5717 | 3 | 2.580798 | -6.177387 | 32.354774 | 3.769357e+07 | * | * | |
| **AY.46.4** | 2996 | 3 | 2.517013 | -6.248599 | 32.497198 | 3.496996e+07 | * | * | |
| **AY.36** | 5985 | 3 | 1.820773 | -6.943830 | 33.887659 | 1.745261e+07 | * | * | |

## BA.1.16 <<

| | num_seq | t_ch_MAX | max_CL | CL@BC_t_ch_MAX | aic | PV | PV_OK | t_ch_MAX_OK | phyl_OK |
|---|---|---|---|---|---|---|---|---|---|
| **BA.1.16** | 3049 | 7 | 62.542765 | NaN | NaN | NaN | * | * | * |
| **BA.1** | 127335 | 7 | 61.218080 | 59.374808 | 13.250383 | 2.671353e-01 | * | * | * |
| **BA.1.17** | 27172 | 7 | 55.026018 | 49.315841 | 33.368319 | 1.142165e-05 | * | * | |
| **BA.1.1** | 349352 | 7 | 53.261114 | 51.414811 | 29.170378 | 9.327112e-05 | * | * | * |
| **BA.1.17.2** | 77688 | 7 | 50.196404 | 44.473652 | 43.052697 | 9.031159e-08 | | * | |
| **BA.1.1.18** | 27122 | 8 | 49.805337 | 46.223585 | 39.552830 | 5.197073e-07 | | * | |
| **BA.1.22** | 57 | 7 | 49.611228 | 42.541810 | 46.916381 | 1.304319e-08 | | * | |
| **BA.1.19** | 1453 | 7 | 49.517286 | 47.669728 | 36.660543 | 2.204522e-06 | | * | |
| **BA.1.18** | 16128 | 7 | 49.292937 | 42.153952 | 47.692095 | 8.875244e-09 | | * | |
| **BA.1.1.2** | 7493 | 11 | 48.870721 | 45.103606 | 41.792789 | 1.695700e-07 | | | |

Cumulative Likelihood per-region

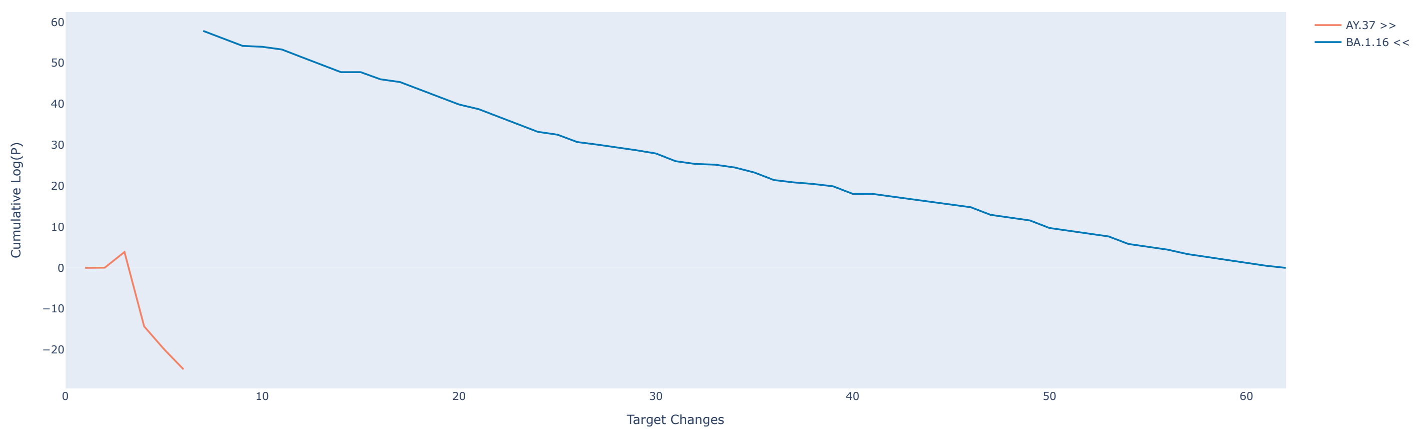


Cumulative Likelihood whole genome

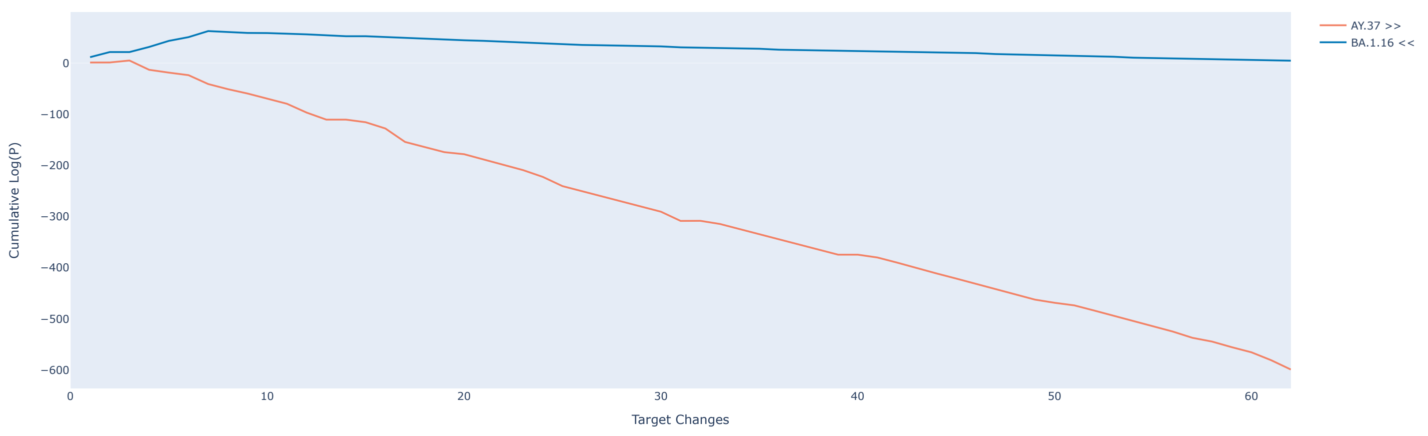


**Target sequence**

, 210_G|T, 241_C|T, 1390_T|C, 2255_A|G, 3037_C|T, 4181_G|T, 6513_GTT|..., 8393_G|A, 10029_C|T, 10449_C|A, 11286_TGTCTGGTT|........., 11537_A|G, 13195_T|C, 14408_C|T, 15240_C|T, 18163_A|G, 21762_C|., 21764_A|., 21767_CATG|...., 21846_C|T, 21987_GTGTTTATT|........., 22194_ATT|..., 22205_.........|AGCCAGAAG, 22578_G|A, 22673_TC|CT, 22679_T|C, 22686_C|T, 22813_G|T, 22882_T|G, 22898_G|A, 22992_G|A, 22995_C|A, 23013_A|C, 23040_A|G, 23048_G|A, 23055_A|G, 23063_A|T, 23075_T|C, 23202_C|A, 23403_A|G, 23525_C|T, 23599_T|G, 23604_C|A, 23854_C|A, 23948_G|T, 24130_C|A, 24424_A|T, 24469_T|A, 24503_C|T, 25000_C|T, 25584_C|T, 26270_C|T, 26530_A|G, 26577_C|G, 26709_G|A, 27259_A|C, 27807_C|T, 28271_A|T, 28311_C|T, 28362_GAGAACGCA|........., 28881_GGG|AAC, 29632_C|T

## Case 32 (1BP 5'): XG test: OK

Target: (75%) 272 samples
GT: BA.1* + BA.2*
BC: BA.1.17 + BA.2
Direction L1: <<
Alt. candidates: [B.1.1.529], [BA.2.7, BA.2.6, BA.2.9, BA.2.25]
Model 1BP/2BP comparison: -
Rec. model vs L1: 2.19e-140
Flags: Model_2BP_Bad_L1_opp

Number of changes: 66
GT BR: 6-7
BC BR: 6-7
Initial region span: 1-6,7-66
Rec. model vs L2: 2.82e-29

GT BR coord: 5926 - 6512
BC BR coord: 5943 - 5944
Gap history (edge excluded):

Rank L1 L2: 11 1

## BA.1.17 >>

| | num_seq | t_ch_MAX | max_CL | CL@BC_t_ch_MAX | aic | PV | PV_OK | t_ch_MAX_OK | phyl_OK |
|---|---|---|---|---|---|---|---|---|---|
| **BA.1.17** | 27172 | 6 | 12.307493 | NaN | NaN | NaN | * | * | * |
| **B.1.1.529** | 1110 | 6 | 6.343611 | 6.343611 | 1.312777 | 2.579912e-03 | * | * | * |
| **BA.1.7** | 220 | 4 | 3.801244 | -16.198756 | 46.397512 | 4.172879e-13 | | | |
| **BC.2** | 99 | 4 | 3.801244 | -16.198756 | 46.397512 | 4.172879e-13 | | | |
| **BA.1.21.1** | 585 | 4 | 3.799533 | -16.200467 | 46.400933 | 4.172879e-13 | | | |
| **BA.1.4** | 266 | 4 | 3.797478 | -16.202522 | 46.405045 | 4.152067e-13 | | | |
| **BA.1.9** | 345 | 4 | 3.795439 | -16.204561 | 46.409123 | 4.152067e-13 | | | |
| **BA.1.14.1** | 2183 | 4 | 3.791607 | -8.028072 | 30.056144 | 1.474422e-09 | | | |
| **BC.1** | 1102 | 4 | 3.791244 | -23.878831 | 61.757662 | 1.927765e-16 | | | |
| **BA.1.15.2** | 896 | 4 | 3.773191 | -16.226809 | 46.453618 | 4.069850e-13 | | | |

## BA.2 <<

| | num_seq | t_ch_MAX | max_CL | CL@BC_t_ch_MAX | aic | PV | PV_OK | t_ch_MAX_OK | phyl_OK |
|---|---|---|---|---|---|---|---|---|---|
| **BA.2** | 378689 | 7 | 49.364133 | NaN | NaN | NaN | * | * | * |
| **BA.2.7** | 2003 | 7 | 48.910262 | 42.739541 | 54.520918 | 1.326780e-03 | * | * | * |
| **BA.2.6** | 1333 | 7 | 44.229040 | 44.229040 | 51.541920 | 5.887052e-03 | * | * | * |
| **BA.2.9** | 108830 | 7 | 43.539377 | 43.539377 | 52.921247 | 2.952804e-03 | * | * | * |
| **BA.2.25** | 331 | 7 | 39.851953 | 39.851953 | 60.296093 | 7.373743e-05 | * | * | * |
| **BA.2.3** | 36275 | 7 | 37.499071 | 32.983025 | 74.033950 | 7.695846e-08 | | * | * |
| **BA.2.69** | 96 | 22 | 35.444409 | 27.407763 | 85.184475 | 2.917861e-10 | | | * |
| **BA.2.14** | 992 | 25 | 35.411132 | 32.796837 | 74.406327 | 6.364150e-08 | | | * |
| **BA.2.71** | 409 | 25 | 35.402450 | 32.439931 | 75.120138 | 4.462373e-08 | | | * |
| **BA.2.9.5** | 1119 | 7 | 35.303492 | 35.303492 | 69.393016 | 7.831043e-07 | | * | * |

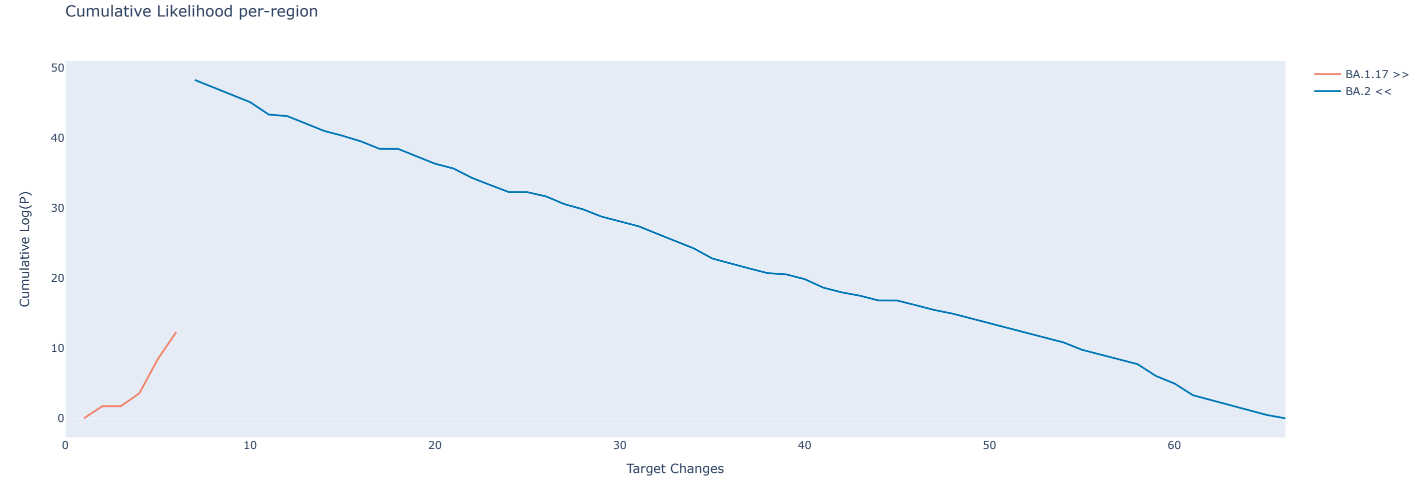


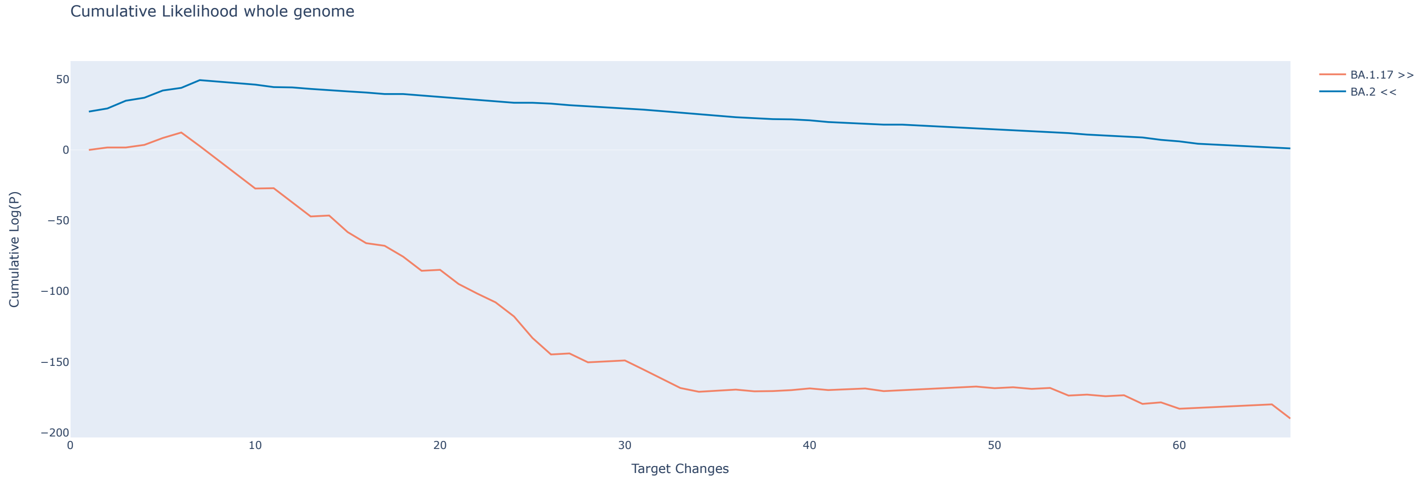


**Target sequence**

, 241_C|T, 2832_A|G, 3037_C|T, 5386_T|G, 5672_C|T, 5924_G|A, 9344_C|T, 9424_A|G, 9534_C|T, 9866_C|T, 10029_C|T, 10198_C|T, 10447_G|A, 10449_C|A, 11288_TCTGGTTTT|........, 12880_C|T, 14408_C|T, 15714_C|T, 17410_C|T, 18163_A|G, 19855_A|G, 19955_C|T, 20055_A|G, 21618_C|T, 21987_G|A, 22200_T|G, 22578_G|A, 22674_C|T, 22679_T|C, 22686_C|T, 22688_A|G, 22775_G|A, 22786_A|C, 22792_C|T, 22813_G|T, 22882_T|G, 22992_G|A, 22995_C|A, 23013_A|C, 23040_A|G, 23055_A|G, 23063_A|T, 23075_T|C, 23403_A|G, 23525_C|T, 23599_T|G, 23604_C|A, 23854_C|A, 23948_G|T, 24424_A|T, 24469_T|A, 25000_C|T, 25584_C|T, 26060_C|T, 26270_C|T, 26577_C|G, 26709_G|A, 26858_C|T, 27259_A|C, 27382_GAT|CTC, 27807_C|T, 28271_A|T, 28311_C|T, 28362_GAGAACGCA|........, 28881_GGG|AAC, 29510_A|C

## Case 33 (1BP 5'): XH    test: OK

Target: (75%) 93 samples    Number of changes: 65
GT: BA.1* + BA.2*    GT BR: 10-12    GT BR coord: 10447 - 11288    Rank L1 L2: 1 3
BC: BA.1 + BA.2.9    BC BR: 10-11    BC BR coord: 10447 - 10448
Direction L1: <<    Initial region span: 1-10,11-65    Gap history (edge excluded):
Alt. candidates: [], [BA.2.9.5]
Model 1BP/2BP comparison:    -
Rec. model vs L1: 7.30e-123    Rec. model vs L2: 5.96e-64
Flags: Model_2BP_Bad_L1_opp

## BA.1 >>

| | num_seq | t_ch_MAX | max_CL | CL@BC_t_ch_MAX | aic | PV | PV_OK | t_ch_MAX_OK | phyl_OK |
|---|---|---|---|---|---|---|---|---|---|
| **BA.1** | 127335 | 10 | 8.186856 | None | None | None | * | * | * |

## BA.2.9 <<

| | num_seq | t_ch_MAX | max_CL | CL@BC_t_ch_MAX | aic | PV | PV_OK | t_ch_MAX_OK | phyl_OK |
|---|---|---|---|---|---|---|---|---|---|
| **BA.2.9** | 108830 | 11 | 51.600395 | NaN | NaN | NaN | * | * | * |
| **BA.2.9.5** | 1119 | 11 | 47.869239 | 47.869239 | 26.261523 | 2.399284e-02 | * | * | * |
| **BA.2** | 378689 | 11 | 40.017890 | 40.017890 | 41.964221 | 9.351255e-06 | | * | * |
| **BA.2.9.3** | 1075 | 11 | 37.278341 | 37.278341 | 47.443318 | 6.038138e-07 | | * | * |
| **BA.2.29** | 1418 | 11 | 36.829992 | 36.829992 | 48.340017 | 3.850087e-07 | | * | |
| **BA.2.36** | 5393 | 11 | 34.995368 | 34.995368 | 52.009265 | 6.145258e-08 | | * | |
| **BA.2.9.2** | 376 | 11 | 33.527050 | 33.527050 | 54.945899 | 1.412951e-08 | | * | * |
| **BA.2.9.6** | 294 | 11 | 31.434604 | 31.434604 | 59.130791 | 1.747639e-09 | | * | * |
| **BA.2.65** | 1625 | 11 | 31.119088 | 31.119088 | 59.761823 | 1.275408e-09 | | * | |
| **BA.2.1** | 3218 | 13 | 30.824980 | 27.144939 | 67.710122 | 2.395126e-11 | | | |

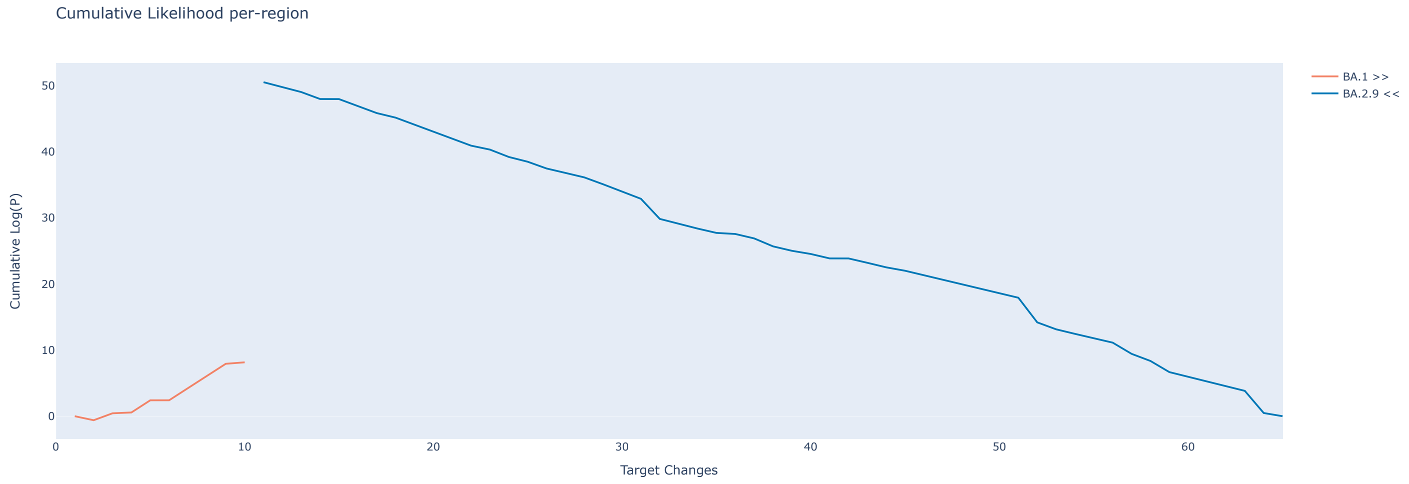


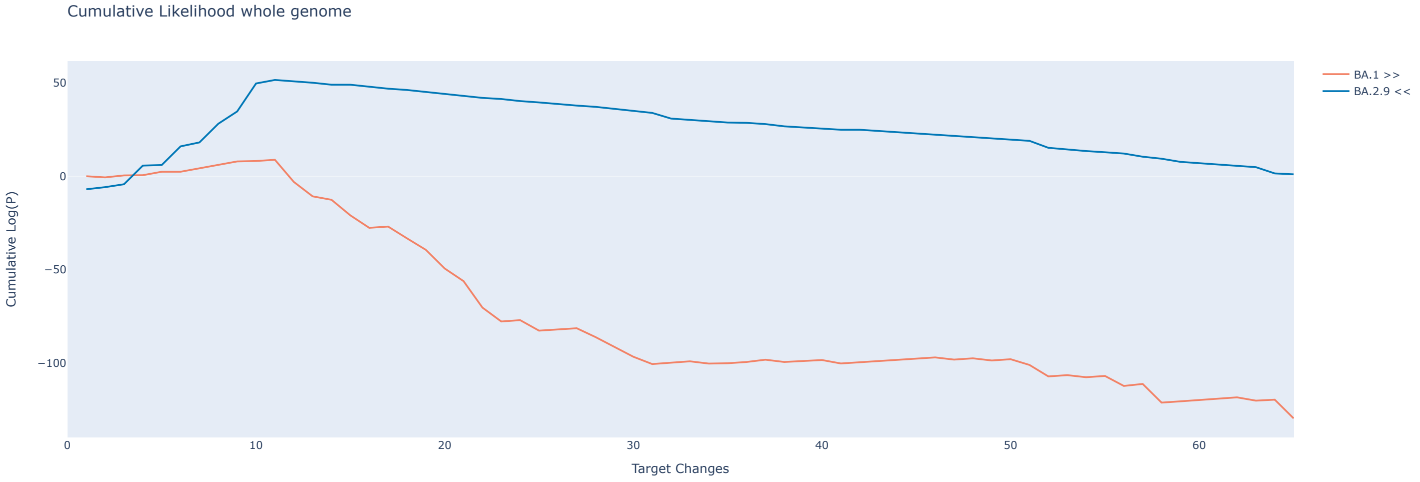


**Target sequence**

, 241_C|T, 902_T|C, 904_C|A, 1244_G|A, 2832_A|G, 3037_C|T, 5386_T|G, 6513_GTT|..., 8393_G|A, 10029_C|T, 10449_C|A, 11288_TCTGGTTTT|........., 12880_C|T, 14408_C|T, 15714_C|T, 17410_C|T, 18163_A|G, 19955_C|T, 20055_A|G, 21618_C|T, 21633_TACCCCCTG|........., 21987_G|A, 22200_T|G, 22578_G|A, 22674_C|T, 22679_T|C, 22686_C|T, 22688_A|G, 22775_G|A, 22786_A|C, 22792_C|T, 22813_G|T, 22882_T|G, 22992_G|A, 22995_C|A, 23013_A|C, 23040_A|G, 23055_A|G, 23063_A|T, 23075_T|C, 23403_A|G, 23525_C|T, 23599_T|G, 23604_C|A, 23854_C|A, 23948_G|T, 24424_A|T, 24469_T|A, 25000_C|T, 25584_C|T, 25624_C|T, 26060_C|T, 26270_C|T, 26577_C|G, 26709_G|A, 26858_C|T, 27259_A|C, 27382_GAT|CTC, 27807_C|T, 28271_A|T, 28311_C|T, 28362_GAGAACGCA|........., 28435_C|T, 28881_GGG|AAC, 29510_A|C

## Case 34 (1BP 5'): XL test: OK

Target: (75%) 28 samples
Number of changes: 69
GT: BA.1* + BA.2*
GT BR: 8–9
GT BR coord: 6517 – 8393
Rank L1 L2: 9 1
BC: BA.1.17.2 + BA.2
BC BR: 8–9
BC BR coord: 6512 – 6513
Direction L1: <<
Initial region span: 1–8,10–69
Gap history (edge excluded): 8–10
Alt. candidates: [BA.1.17, BD.1], [BA.2.3, BA.2.9]
Model 1BP/2BP comparison: –
Rec. model vs L1: 2.63e–152
Rec. model vs L2: 1.36e–42
Flags: Model_2BP_Bad_L1_opp

## BA.1.17.2 >>

| | num_seq | t_ch_MAX | max_CL | CL@BC_t_ch_MAX | aic | PV | PV_OK | t_ch_MAX_OK | phyl_OK |
|---|---|---|---|---|---|---|---|---|---|
| **BA.1.17.2** | 77688 | 8 | 12.447828 | NaN | NaN | NaN | * | * | * |
| **BA.1.17** | 27172 | 8 | 8.458423 | 8.458423 | 1.083154 | 1.849971e-02 | * | * | * |
| **BD.1** | 2033 | 8 | 3.513202 | 3.513202 | 10.973556 | 1.316979e-04 | * | * | * |
| **BA.1.20** | 11897 | 4 | 3.359384 | -3.015527 | 24.031054 | 1.921480e-07 | | | |
| **BA.1.21** | 2247 | 4 | 2.475668 | -7.415792 | 32.831584 | 2.359066e-09 | | | |
| **BA.1.15.1** | 21857 | 4 | 2.238006 | -3.415304 | 24.830607 | 1.288006e-07 | | | |
| **BA.1.1.1** | 29279 | 4 | 1.716965 | -2.183745 | 22.367490 | 4.406566e-07 | | | |
| **BA.1.16** | 3049 | 4 | 1.448955 | -16.361416 | 50.722832 | 3.075927e-13 | | | |
| **BA.1** | 127335 | 4 | 1.344747 | -8.348326 | 34.696653 | 9.261360e-10 | | | * |
| **BA.1.14** | 3706 | 3 | 1.285142 | -9.136773 | 36.273545 | 4.224289e-10 | | | |

## BA.2 <<

| | num_seq | t_ch_MAX | max_CL | CL@BC_t_ch_MAX | aic | PV | PV_OK | t_ch_MAX_OK | phyl_OK |
|---|---|---|---|---|---|---|---|---|---|
| **BA.2** | 378689 | 10 | 47.759799 | NaN | NaN | NaN | * | * | * |
| **BA.2.3** | 36275 | 10 | 43.955245 | 38.850528 | 62.298944 | 2.117721e-04 | * | * | * |
| **BA.2.10** | 28641 | 10 | 42.479636 | 32.479636 | 75.040728 | 3.625875e-07 | | * | * |
| **BA.2.9** | 108830 | 10 | 41.162423 | 39.475095 | 61.049811 | 3.956423e-04 | * | * | * |
| **BA.2.1** | 3218 | 21 | 39.913001 | 24.654515 | 90.690969 | 1.448967e-10 | | | * |
| **BA.2.29** | 1418 | 21 | 39.390935 | 29.324042 | 81.351916 | 1.546015e-08 | | | * |
| **BA.2.23** | 3557 | 21 | 38.936750 | 28.810907 | 82.378186 | 9.237450e-09 | | | * |
| **BA.2.27** | 215 | 21 | 38.409675 | 28.295427 | 83.409147 | 5.519382e-09 | | | * |
| **BA.2.31** | 1389 | 21 | 38.312163 | 34.039801 | 71.920398 | 1.725489e-06 | | | * |
| **BA.2.26** | 490 | 22 | 38.075873 | 20.575375 | 98.849249 | 2.449836e-12 | | | * |

Cumulative Likelihood per-region

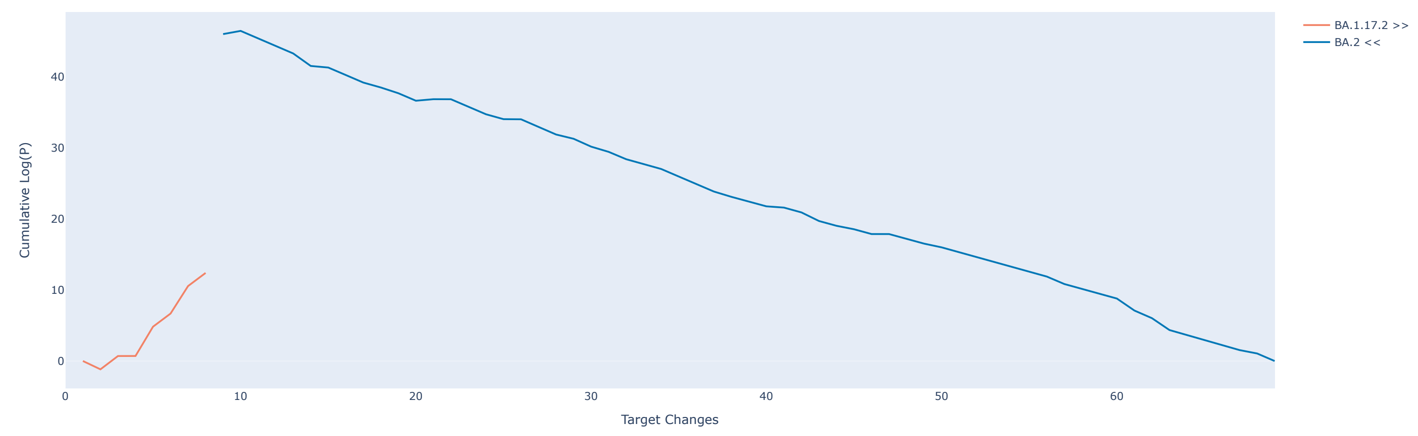


Cumulative Likelihood whole genome

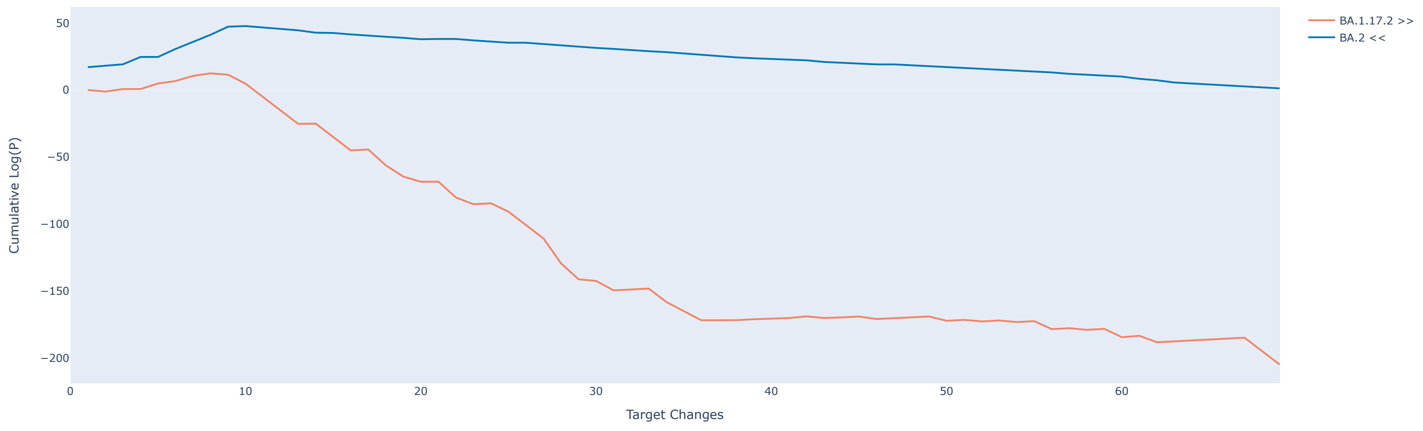


**Target sequence**

, 241_C|T, 875_C|T, 2832_A|G, 3037_C|T, 3241_C|T, 5386_T|G, 5924_G|A, 6513_GTT|..., 9208_T|C, 9344_C|T, 9424_A|G, 9534_C|T, 9866_C|T, 10029_C|T, 10198_C|T, 10447_G|A, 10449_C|A, 11288_TCTGGTTTT|........., 12880_C|T, 14229_G|A, 14408_C|T, 15714_C|T, 17410_C|T, 18163_A|G, 19955_C|T, 21618_C|T, 21633_TACCCCCTG|........., 21987_G|A, 22200_T|G, 22578_G|A, 22674_C|T, 22679_T|C, 22686_C|T, 22688_A|G, 22775_G|A, 22786_A|C, 22813_G|T, 22882_T|G, 22992_G|A, 22995_C|A, 23013_A|C, 23040_A|G, 23055_A|G, 23063_A|T, 23075_T|C, 23403_A|G, 23525_C|T, 23599_T|G, 23604_C|A, 23854_C|A, 23948_G|T, 24424_A|T, 24469_T|A, 25000_C|T, 25584_C|T, 26060_C|T, 26270_C|T, 26577_C|G, 26709_G|A, 26858_C|T, 27259_A|C, 27382_GAT|CTC, 27807_C|T, 28271_A|T, 28311_C|T, 28362_GAGAACGCA|........., 28881_GGG|AAC, 29510_A|C, 29734_GAGGCCACGCGGAGTACGATCGAGTG|..........................

## Case 35 (1BP 5'): XN    test: KO

Target: (75%) 82 samples
GT: BA.1* + BA.2*
BC: BA.2
Direction L1: <<
Alt. candidates: [BA.2.9]
Model 1BP/2BP comparison: −
Rec. model vs L1: −
Flags: NotEnoughSpaceAfterL1, SingleCandidateGenome

Number of changes: 66
GT BR: 2–4    GT BR coord: 2833 − 4184    Rank L1 L2: 11 −
BC BR:    BC BR coord:
Initial region span: 3–66    Gap history (edge excluded):
Rec. model vs L2: −

# BA.2 <<

| | num_seq | t_ch_MAX | max_CL | CL@BC_t_ch_MAX | aic | PV | PV_OK | t_ch_MAX_OK | phyl_OK |
|---|---|---|---|---|---|---|---|---|---|
| **BA.2** | 378689 | 3 | 52.350025 | NaN | NaN | NaN | * | * | * |
| **BA.2.9** | 108830 | 3 | 47.530232 | 35.325223 | 91.349554 | 8.783944e-05 | * | * | * |
| **BA.2.25** | 331 | 3 | 43.101101 | 22.149113 | 117.701774 | 1.666709e-10 | | * | * |
| **BA.2.69** | 96 | 18 | 41.705367 | 20.816807 | 120.366387 | 4.386081e-11 | | | * |
| **BA.2.7** | 2003 | 15 | 40.673883 | 22.228497 | 117.543007 | 1.805524e-10 | | | * |
| **BA.2.9.5** | 1119 | 15 | 40.376735 | 20.021232 | 121.957536 | 1.980672e-11 | | | * |
| **BA.2.1** | 3218 | 16 | 38.984958 | 17.563386 | 126.873228 | 1.700668e-12 | | | * |
| **BA.2.29** | 1418 | 3 | 37.248366 | 17.062883 | 127.874234 | 1.031507e-12 | | * | * |
| **BA.2.9.6** | 294 | 15 | 36.547468 | 12.724920 | 136.550160 | 1.344727e-14 | | | * |
| **BA.2.9.2** | 376 | 15 | 36.364860 | 18.114494 | 125.771013 | 2.947688e-12 | | | * |

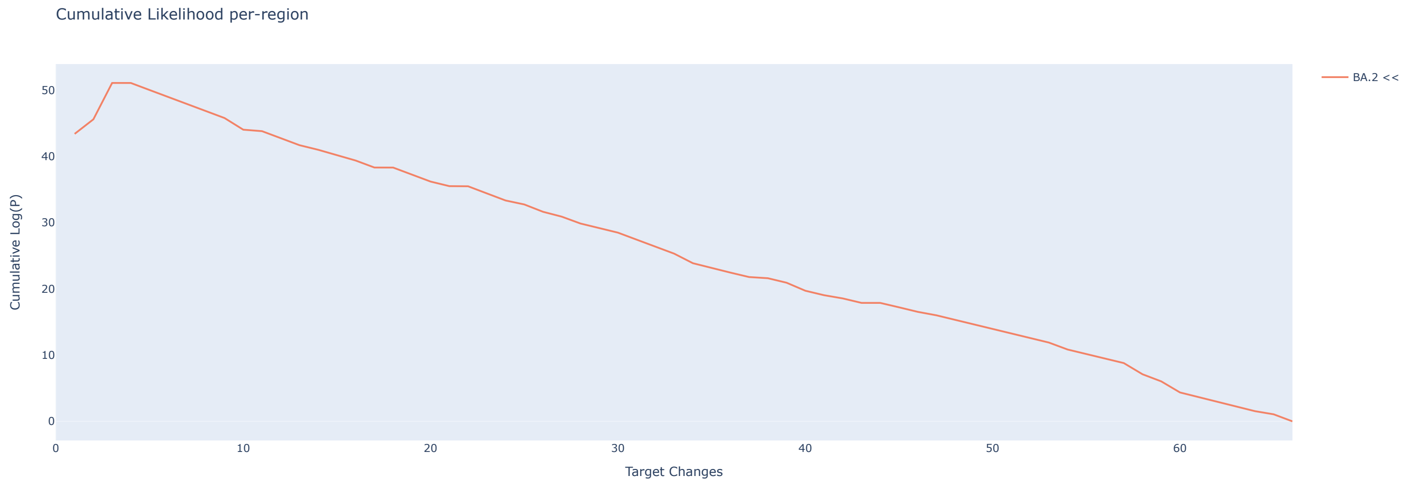


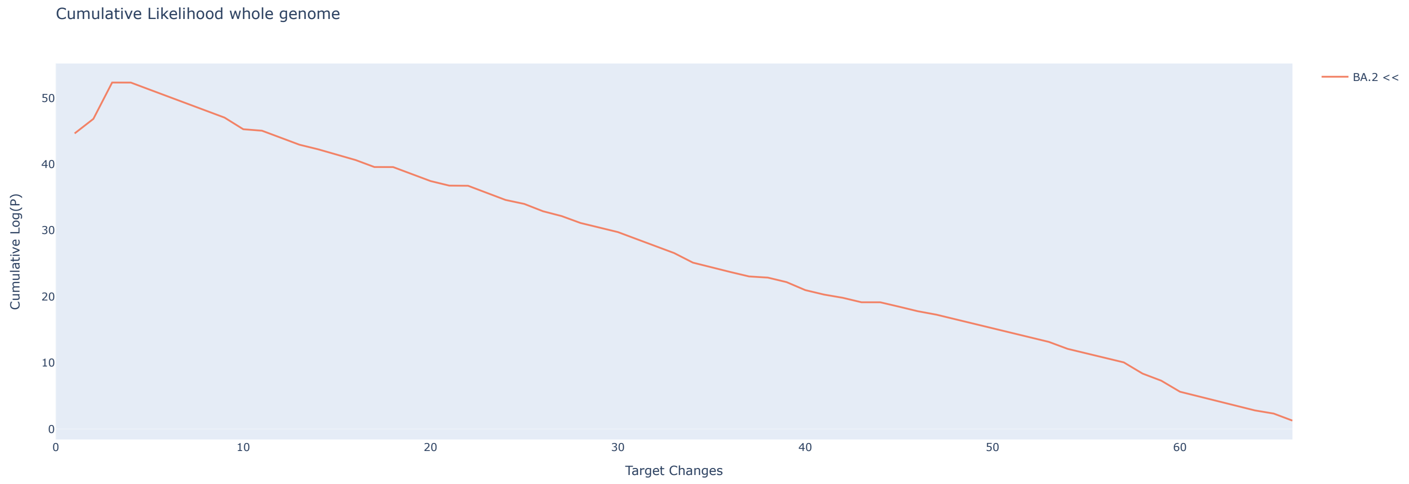


**Target sequence**

, 241_C|T, 2832_A|G, 3037_C|T, 4184_G|A, 4321_C|T, 9344_C|T, 9424_A|G, 9534_C|T, 9866_C|T, 10029_C|T, 10198_C|T, 10447_G|A, 10449_C|A, 10986_G|A, 11288_TCTGGTTTT|........., 12880_C|T, 14408_C|T, 15714_C|T, 17410_C|T, 18163_A|G, 19955_C|T, 21618_C|T, 21633_TACCCCCTG|........., 21987_G|A, 22200_T|G, 22578_G|A, 22674_C|T, 22679_T|C, 22686_C|T, 22688_A|G, 22775_G|A, 22786_A|C, 22792_C|T, 22813_G|T, 22882_T|G, 22992_G|A, 22995_C|A, 23013_A|C, 23040_A|G, 23055_A|G, 23063_A|T, 23075_T|C, 23403_A|G, 23525_C|T, 23599_T|G, 23604_C|A, 23854_C|A, 23948_G|T, 24424_A|T, 24469_T|A, 25000_C|T, 25584_C|T, 26060_C|T, 26270_C|T, 26577_C|G, 26709_G|A, 26858_C|T, 27259_A|C, 27382_GAT|CTC, 27807_C|T, 28271_A|T, 28311_C|T, 28362_GAGAACGCA|........., 28881_GGG|AAC, 29510_A|C, 29734_GAGGCCACGCGGAGTACGATCGAGTG|..........................

## Case 36 (1BP 5'): XQ test: OK

Target: (75%) 43 samples
Number of changes: 64
GT: BA.1.1* + BA.2*
GT BR: 4–5
GT BR coord: 4321 – 5386
Rank L1 L2: 9 2
BC: BA.1.1.16 + BA.2.5
BC BR: 4–5
BC BR coord: 4330 – 4331
Direction L1: <<
Initial region span: 1–4,5–64
Gap history (edge excluded):
Alt. candidates: [BA.1.1], [BA.2]
Model 1BP/2BP comparison: –
Rec. model vs L1: 1.23e–310
Rec. model vs L2: 3.77e–61
Flags: Model_2BP_Bad_L1_opp

## BA.1.1.16 >>

| | num_seq | t_ch_MAX | max_CL | CL@BC_t_ch_MAX | aic | PV | PV_OK | t_ch_MAX_OK | phyl_OK |
|---|---|---|---|---|---|---|---|---|---|
| **BA.1.1.16** | 1583 | 4 | 4.475561 | NaN | NaN | NaN | * | * | * |
| **BA.1.1.2** | 7493 | 4 | 4.475351 | 4.475351 | 1.049298 | 1.000000 | * | * | |
| **BA.1.1.18** | 27122 | 4 | 4.464734 | 4.464734 | 1.070532 | 0.990050 | * | * | |
| **BA.1.1.6** | 86 | 4 | 4.458730 | 4.458730 | 1.082541 | 0.985112 | * | * | |
| **BA.1.1.13** | 3131 | 4 | 4.434226 | 4.434226 | 1.131548 | 0.960789 | * | * | |
| **BA.1.1.15** | 6410 | 4 | 4.433709 | 4.433709 | 1.132582 | 0.960789 | * | * | |
| **BA.1.1.10** | 1083 | 4 | 4.433057 | 4.433057 | 1.133885 | 0.960789 | * | * | |
| **BA.1.1.12** | 1392 | 3 | 4.430911 | -3.586769 | 17.173538 | 0.000316 | * | * | |
| **BA.1.1** | 349352 | 4 | 4.427017 | 4.427017 | 1.145967 | 0.951229 | * | * | * |
| **BA.1.1.14** | 8250 | 4 | 4.414915 | 4.414915 | 1.170169 | 0.941765 | * | * | |

## BA.2.5 <<

| | num_seq | t_ch_MAX | max_CL | CL@BC_t_ch_MAX | aic | PV | PV_OK | t_ch_MAX_OK | phyl_OK |
|---|---|---|---|---|---|---|---|---|---|
| **BA.2.5** | 1908 | 5 | 45.503751 | NaN | NaN | NaN | * | * | * |
| **BA.2** | 378689 | 5 | 44.690863 | 44.690863 | 44.618275 | 4.426393e-01 | * | * | * |
| **BA.2.23** | 3557 | 5 | 44.641762 | 44.641762 | 44.716476 | 4.210516e-01 | * | * | |
| **BA.2.33** | 389 | 5 | 41.431985 | 35.916966 | 62.166068 | 6.840942e-05 | * | * | |
| **BA.2.3** | 36275 | 5 | 41.142992 | 36.626946 | 60.746109 | 1.391442e-04 | * | * | |
| **BA.2.10** | 28641 | 5 | 39.864093 | 39.864093 | 54.271814 | 3.552868e-03 | * | * | |
| **BA.2.29** | 1418 | 5 | 39.324042 | 39.324042 | 55.351916 | 2.070428e-03 | * | * | |
| **BA.2.27** | 215 | 5 | 38.295427 | 38.295427 | 57.409147 | 7.391572e-04 | * | * | |
| **BA.2.31** | 1389 | 5 | 38.137115 | 38.137115 | 57.725770 | 6.298682e-04 | * | * | |
| **BA.2.26** | 490 | 5 | 37.994317 | 30.575375 | 72.849249 | 3.280827e-07 | | * | |

Cumulative Likelihood per-region

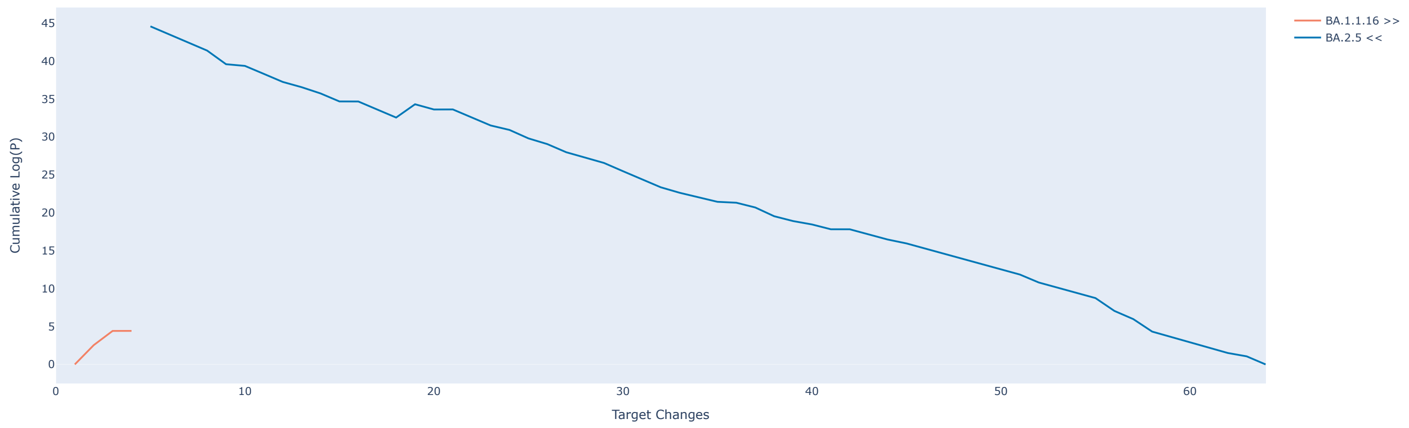


Cumulative Likelihood whole genome

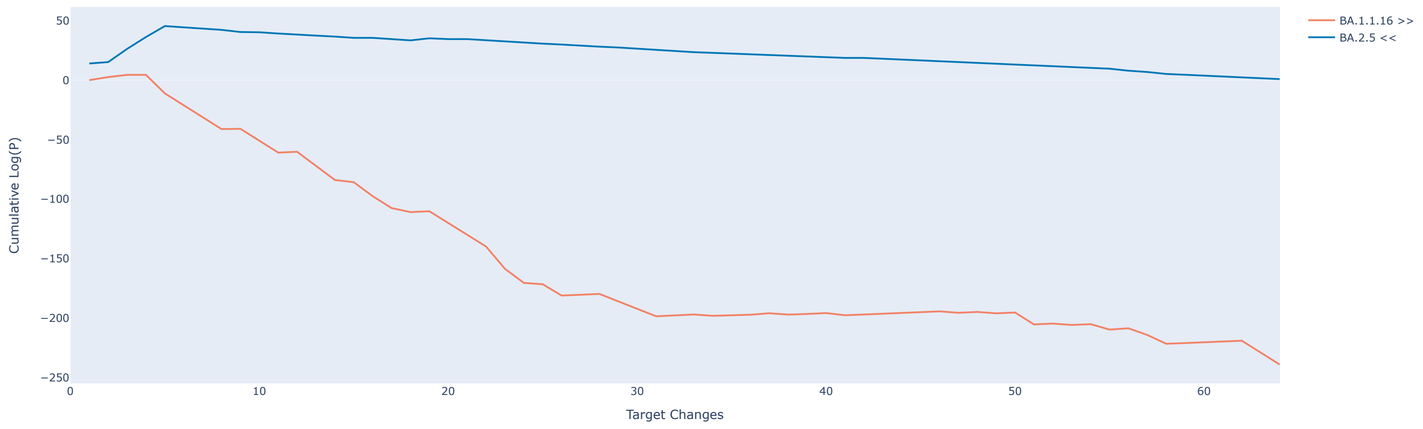


**Target sequence**

, 241_C|T, 2470_C|T, 2832_A|G, 3037_C|T, 9344_C|T, 9424_A|G, 9534_C|T, 9866_C|T, 10029_C|T, 10198_C|T, 10447_G|A, 10449_C|A, 11288_TCTGGTTTT|........., 12880_C|T, 14408_C|T, 15714_C|T, 17410_C|T, 17615_A|G, 18163_A|G, 19955_C|T, 21618_C|T, 21633_TACCCCCTG|........., 21987_G|A, 22200_T|G, 22578_G|A, 22674_C|T, 22679_T|C, 22686_C|T, 22688_A|G, 22775_G|A, 22786_A|C, 22813_G|T, 22882_T|G, 22992_G|A, 22995_C|A, 23013_A|C, 23040_A|G, 23055_A|G, 23063_A|T, 23075_T|C, 23403_A|G, 23525_C|T, 23599_T|G, 23604_C|A, 23854_C|A, 23948_G|T, 24424_A|T, 24469_T|A, 25000_C|T, 25584_C|T, 26060_C|T, 26270_C|T, 26577_C|G, 26709_G|A, 26858_C|T, 27259_A|C, 27382_GAT|CTC, 27807_C|T, 28271_A|T, 28311_C|T, 28362_GAGAACGCA|........., 28881_GGG|AAC, 29510_A|C, 29734_GAGGCCACGCGGAGTACGATCGAGTG|..........................

## Case 37 (1BP 5'): XR          test: OK

Target: (75%) 91 samples
Number of changes: 66
GT: BA.1.1* + BA.2*
GT BR: 4-5
GT BR coord: 4321 - 4892
Rank L1 L2: 9 1
BC: BA.1.1.16 + BA.2
BC BR: 4-5
BC BR coord: 4330 - 4331
Direction L1: <<
Initial region span: 1-4,5-66
Gap history (edge excluded):
Alt. candidates: [BA.1.1], []
Model 1BP/2BP comparison: -
Rec. model vs L1: 3.44e-263
Rec. model vs L2: 8.24e-42
Flags: Model_2BP_Bad_L1_opp

## BA.1.1.16 >>

| | num_seq | t_ch_MAX | max_CL | CL@BC_t_ch_MAX | aic | PV | PV_OK | t_ch_MAX_OK | phyl_OK |
|---|---|---|---|---|---|---|---|---|---|
| **BA.1.1.16** | 1583 | 4 | 4.475561 | NaN | NaN | NaN | * | * | * |
| **BA.1.1.2** | 7493 | 4 | 4.475351 | 4.475351 | 1.049298 | 1.000000 | * | * | |
| **BA.1.1.18** | 27122 | 4 | 4.464734 | 4.464734 | 1.070532 | 0.990050 | * | * | |
| **BA.1.1.6** | 86 | 4 | 4.458730 | 4.458730 | 1.082541 | 0.985112 | * | * | |
| **BA.1.1.13** | 3131 | 4 | 4.434226 | 4.434226 | 1.131548 | 0.960789 | * | * | |
| **BA.1.1.15** | 6410 | 4 | 4.433709 | 4.433709 | 1.132582 | 0.960789 | * | * | |
| **BA.1.1.10** | 1083 | 4 | 4.433057 | 4.433057 | 1.133885 | 0.960789 | * | * | |
| **BA.1.1.12** | 1392 | 3 | 4.430911 | -3.586769 | 17.173538 | 0.000316 | * | * | |
| **BA.1.1** | 349352 | 4 | 4.427017 | 4.427017 | 1.145967 | 0.951229 | * | * | * |
| **BA.1.1.14** | 8250 | 4 | 4.414915 | 4.414915 | 1.170169 | 0.941765 | * | * | |

## BA.2 <<

| | num_seq | t_ch_MAX | max_CL | CL@BC_t_ch_MAX | aic | PV | PV_OK | t_ch_MAX_OK | phyl_OK |
|---|---|---|---|---|---|---|---|---|---|
| **BA.2** | 378689 | 5 | 50.046163 | NaN | NaN | NaN | * | * | * |
| **BA.2.9** | 108830 | 6 | 40.051020 | 37.947127 | 66.105747 | 5.559513e-06 | | * | * |
| **BA.2.10** | 28641 | 19 | 36.906353 | 32.660443 | 76.679115 | 2.817023e-08 | | | * |
| **BA.2.56** | 3106 | 17 | 34.147320 | 32.681188 | 76.637625 | 2.873931e-08 | | | * |
| **BA.2.3** | 36275 | 17 | 33.886207 | 28.651366 | 84.697268 | 5.108220e-10 | | | * |
| **BA.2.29** | 1418 | 17 | 30.059381 | 19.992488 | 102.015024 | 8.856851e-14 | | | * |
| **BA.2.1** | 3218 | 17 | 29.240554 | 21.570561 | 98.858879 | 4.299962e-13 | | | * |
| **BA.2.23** | 3557 | 17 | 29.237260 | 27.292899 | 87.414202 | 1.317651e-10 | | | * |
| **BA.2.27** | 215 | 17 | 29.154949 | 19.040701 | 103.918599 | 3.425308e-14 | | | * |
| **BA.2.31** | 1389 | 17 | 29.092382 | 12.146145 | 117.707710 | 3.469278e-17 | | | * |

Cumulative Likelihood per-region

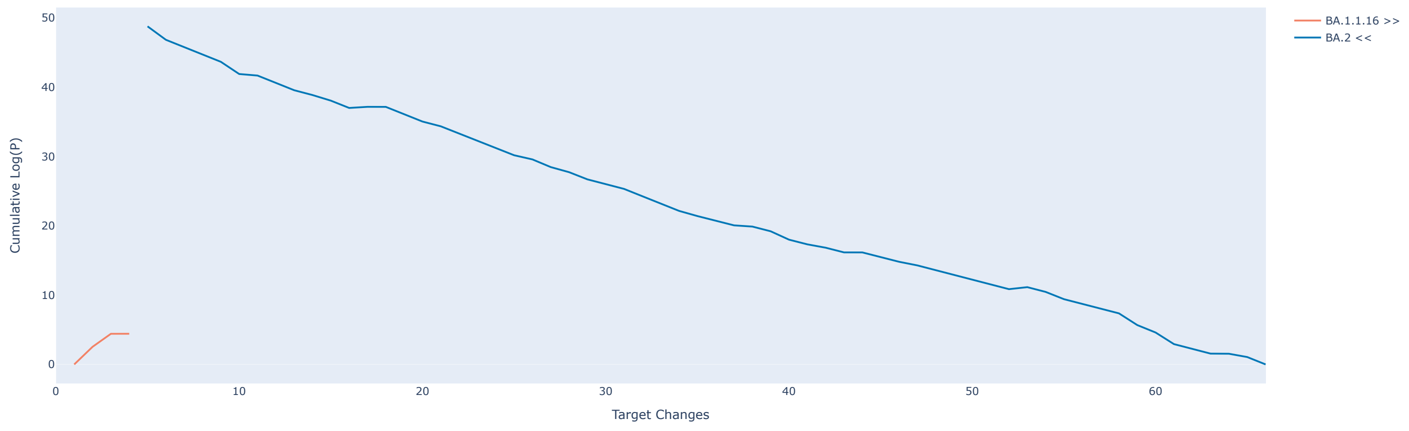


Cumulative Likelihood whole genome

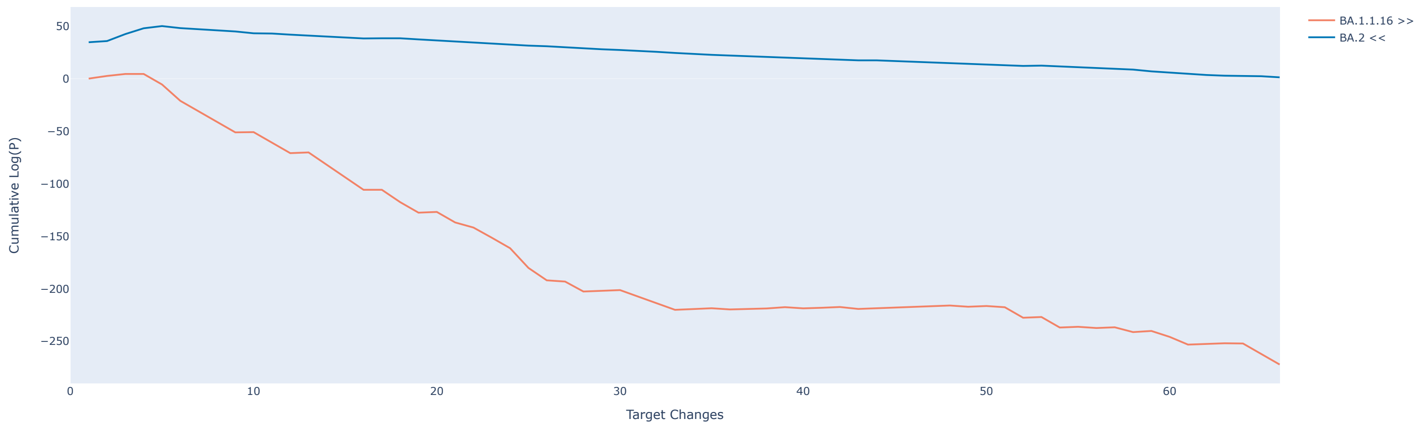


**Target sequence**

, 241_C|T, 2470_C|T, 2832_A|G, 3037_C|T, 4893_C|T, 9344_C|T, 9424_A|G, 9534_C|T, 9866_C|T, 10029_C|T, 10198_C|T, 10447_G|A, 10449_C|A, 11288_TCTGGTTTT|........., 12880_C|T, 13295_G|A, 14408_C|T, 15714_C|T, 17410_C|T, 18163_A|G, 19955_C|T, 20055_A|G, 21618_C|T, 21633_TACCCCCTG|........., 21987_G|A, 22200_T|G, 22578_G|A, 22674_C|T, 22679_T|C, 22686_C|T, 22688_A|G, 22775_G|A, 22786_A|C, 22813_G|T, 22882_T|G, 22992_G|A, 22995_C|A, 23013_A|C, 23040_A|G, 23055_A|G, 23063_A|T, 23075_T|C, 23403_A|G, 23525_C|T, 23599_T|G, 23604_C|A, 23854_C|A, 23948_G|T, 24424_A|T, 24469_T|A, 25000_C|T, 25524_A|G, 25584_C|T, 26060_C|T, 26270_C|T, 26577_C|G, 26709_G|A, 26858_C|T, 27259_A|C, 27382_GAT|CTC, 27807_C|T, 28271_A|T, 28311_C|T, 28881_GGG|AAC, 29510_A|C, 29734_GAGGCCACGCGGAGTACGATCGAGTG|..........................

## Case 38 (1BP 5'): XS                test: OK

Target: (75%) 15 samples
GT: B.1.617.2* + BA.1.1*
BC: AY.126 + BA.1.1
Direction L1: <<
Alt. candidates: [], [BA.1.1.16, BA.1.1.18, BA.1, BA.1.17]
Model 1BP/2BP comparison: -
Rec. model vs L1: 0.00e+00
Flags: Model_2BP_Bad_L1_opp

Number of changes: 64
GT BR: 10-12
BC BR: 10-11
Initial region span: 1-10,11-64
Rec. model vs L2: 8.23e-63

GT BR coord: 9054 - 10448
BC BR coord: 9069 - 9070
Gap history (edge excluded):

Rank L1 L2: 11 1

## AY.126 >>

| | num_seq | t_ch_MAX | max_CL | CL@BC_t_ch_MAX | aic | PV | PV_OK | t_ch_MAX_OK | phyl_OK |
|---|---|---|---|---|---|---|---|---|---|
| **AY.126** | 28427 | 10 | 9.079379 | NaN | NaN | NaN | * | * | * |
| **AY.3** | 78123 | 10 | 8.146715 | 8.146715 | 3.706571 | 0.392586 | * | * | |
| **AY.43** | 140119 | 10 | 6.968258 | 6.968258 | 6.063485 | 0.121238 | * | * | |
| **AY.36** | 5985 | 10 | 6.533682 | 6.533682 | 6.932637 | 0.078473 | * | * | |
| **AY.25.2** | 421 | 10 | 6.516813 | 6.516813 | 6.966375 | 0.076919 | * | * | |
| **AY.116** | 1253 | 10 | 6.268967 | 6.268967 | 7.462067 | 0.060205 | * | * | |
| **AY.112** | 7084 | 10 | 6.042436 | 6.042436 | 7.915129 | 0.047835 | * | * | |
| **AY.34.1** | 3230 | 10 | 5.820653 | 5.820653 | 8.358694 | 0.038388 | * | * | |
| **AY.129** | 6725 | 10 | 5.374521 | 5.374521 | 9.250958 | 0.024600 | * | * | |
| **AY.103** | 145033 | 10 | 4.794493 | 4.794493 | 10.411015 | 0.013774 | * | * | |

## BA.1.1 <<

| | num_seq | t_ch_MAX | max_CL | CL@BC_t_ch_MAX | aic | PV | PV_OK | t_ch_MAX_OK | phyl_OK |
|---|---|---|---|---|---|---|---|---|---|
| **BA.1.1** | 349352 | 11 | 55.637057 | NaN | NaN | NaN | * | * | * |
| **BA.1.1.16** | 1583 | 11 | 49.345182 | 49.345182 | 21.309636 | 1.854760e-03 | * | * | * |
| **BA.1.1.18** | 27122 | 11 | 47.248904 | 47.248904 | 25.502192 | 2.282658e-04 | * | * | * |
| **BA.1** | 127335 | 11 | 46.797053 | 46.797053 | 26.405894 | 1.448227e-04 | * | * | * |
| **BA.1.1.1** | 29279 | 20 | 45.065055 | 44.701912 | 30.596176 | 1.782337e-05 | * | | * |
| **BA.1.1.2** | 7493 | 20 | 44.991666 | 37.361643 | 45.276715 | 1.156827e-08 | | | * |
| **BA.1.17** | 27172 | 11 | 44.883430 | 44.883430 | 30.233139 | 2.144541e-05 | * | * | * |
| **BA.1.1.10** | 1083 | 15 | 44.719094 | 43.437907 | 33.124187 | 5.055671e-06 | | | * |
| **BA.1.1.12** | 1392 | 20 | 44.240548 | 43.188174 | 33.623652 | 3.937360e-06 | | | * |
| **BA.1.1.14** | 8250 | 20 | 43.825319 | 43.466650 | 33.066699 | 5.183656e-06 | | | * |

Cumulative Likelihood per-region

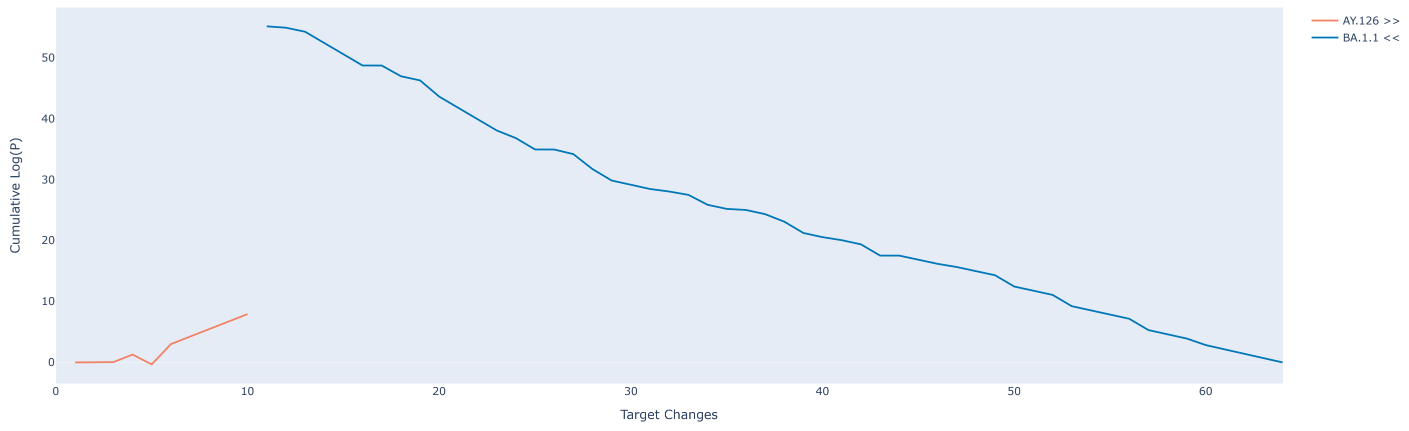


Cumulative Likelihood whole genome

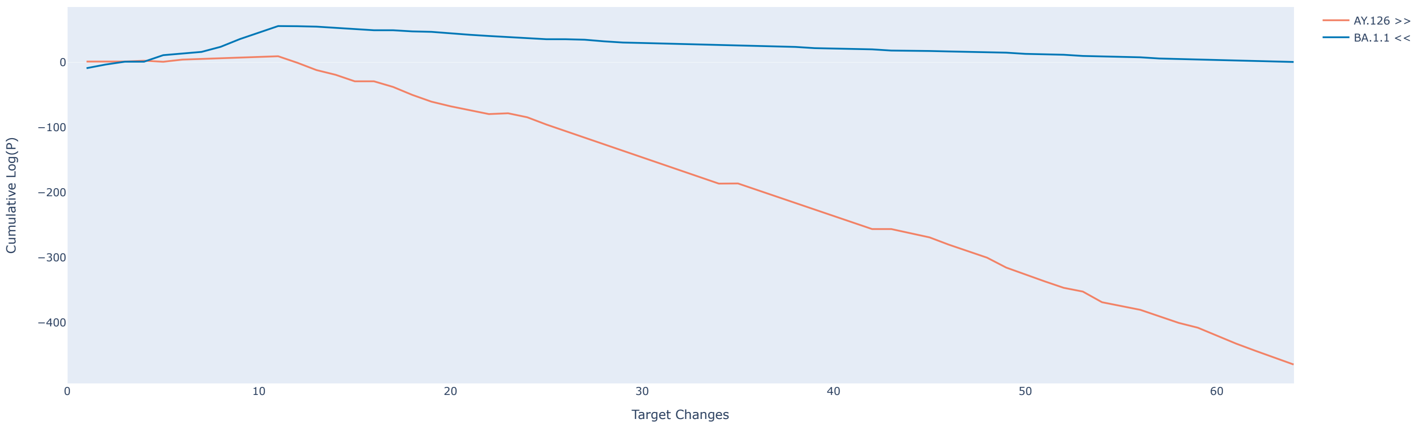


**Target sequence**

, 210_G|T, 241_C|T, 3037_C|T, 4181_G|T, 5365_C|T, 6196_C|T, 6402_C|T, 7124_C|T, 8986_C|T, 9053_G|T, 10029_C|T, 10449_C|A, 11286_TGTCTGGTT|........., 11537_A|G, 13195_T|C, 14408_C|T, 15240_C|T, 18163_A|G, 21595_C|T, 21762_C|., 21764_A|., 21767_CATG|...., 21846_C|T, 21987_GTGTTTATT|........., 22194_ATT|..., 22578_G|A, 22599_G|A, 22673_TC|CT, 22679_T|C, 22686_C|T, 22813_G|T, 22882_T|G, 22898_G|A, 22992_G|A, 22995_C|A, 23013_A|C, 23040_A|G, 23048_G|A, 23055_A|G, 23063_A|T, 23075_T|C, 23202_C|A, 23403_A|G, 23525_C|T, 23599_T|G, 23604_C|A, 23854_C|A, 23948_G|T, 24130_C|A, 24424_A|T, 24469_T|A, 24503_C|T, 25000_C|T, 25584_C|T, 26270_C|T, 26530_A|G, 26577_C|G, 26709_G|A, 27259_A|C, 27807_C|T, 28271_A|T, 28311_C|T, 28362_GAGAACGCA|........., 28881_GGG|AAC

## Case 39 (1BP 5'): XU          test: OK

Target: (75%) 4 samples — Number of changes: 63
GT: BA.1* + BA.2* — GT BR: 5–6 — GT BR coord: 6517 – 9344 — Rank L1 L2: 11 2
BC: BA.1.17 + BA.2.37 — BC BR: 5–6 — BC BR coord: 5943 – 5944
Direction L1: << — Initial region span: 1–5,6–63 — Gap history (edge excluded):
Alt. candidates: [B.1.1.529], [BA.2]
Model 1BP/2BP comparison: –
Rec. model vs L1: 2.49e–224 — Rec. model vs L2: 8.39e–51
Flags: Model_2BP_Bad_L1_opp

## BA.1.17 >>

| | num_seq | t_ch_MAX | max_CL | CL@BC_t_ch_MAX | aic | PV | PV_OK | t_ch_MAX_OK | phyl_OK |
|---|---|---|---|---|---|---|---|---|---|
| **BA.1.17** | 27172 | 5 | 7.424514 | NaN | NaN | NaN | * | * | * |
| **B.1.1.529** | 1110 | 5 | 4.442427 | 4.442427 | 3.115145 | 5.053950e-02 | * | * | * |
| **BC.2** | 99 | 4 | 3.801244 | -6.198756 | 24.397512 | 1.209867e-06 | | * | |
| **BA.1.7** | 220 | 4 | 3.801244 | -6.198756 | 24.397512 | 1.209867e-06 | | * | |
| **BA.1.21.1** | 585 | 4 | 3.799533 | -6.200467 | 24.400933 | 1.209867e-06 | | * | |
| **BA.1.4** | 266 | 4 | 3.797478 | -6.202522 | 24.405045 | 1.203833e-06 | | * | |
| **BA.1.9** | 345 | 4 | 3.795439 | -6.204561 | 24.409123 | 1.203833e-06 | | * | |
| **BA.1.14.1** | 2183 | 4 | 3.791607 | -6.208393 | 24.416787 | 1.197829e-06 | | * | |
| **BC.1** | 1102 | 4 | 3.791244 | -13.878831 | 39.757662 | 5.589283e-10 | | * | |
| **BA.1.15.2** | 896 | 4 | 3.773191 | -6.226809 | 24.453618 | 1.179995e-06 | | * | |

## BA.2.37 <<

| | num_seq | t_ch_MAX | max_CL | CL@BC_t_ch_MAX | aic | PV | PV_OK | t_ch_MAX_OK | phyl_OK |
|---|---|---|---|---|---|---|---|---|---|
| **BA.2.37** | 2257 | 6 | 45.627539 | NaN | NaN | NaN | * | * | * |
| **BA.2** | 378689 | 6 | 44.173013 | 44.173013 | 55.653974 | 2.334004e-01 | * | * | * |
| **BA.2.12** | 2844 | 6 | 42.823672 | 42.823672 | 58.352655 | 6.050677e-02 | * | * | |
| **BA.2.73** | 273 | 6 | 41.726775 | 40.517191 | 62.965618 | 6.005978e-03 | * | * | |
| **BA.2.67** | 258 | 15 | 40.587890 | 30.311856 | 83.376289 | 2.221307e-07 | | | |
| **BA.2.23** | 3557 | 6 | 38.738467 | 38.738467 | 66.523066 | 1.017914e-03 | * | * | |
| **BA.2.68** | 711 | 16 | 38.112645 | 37.602218 | 68.795564 | 3.255482e-04 | * | | |
| **BA.2.5** | 1908 | 6 | 37.932899 | 37.932899 | 68.134202 | 4.550970e-04 | * | * | |
| **BA.2.57** | 241 | 20 | 36.468629 | 21.331097 | 101.337806 | 2.796688e-11 | | | |
| **BA.2.3** | 36275 | 6 | 36.127116 | 31.611070 | 80.777860 | 8.150633e-07 | | * | * |

Cumulative Likelihood per-region

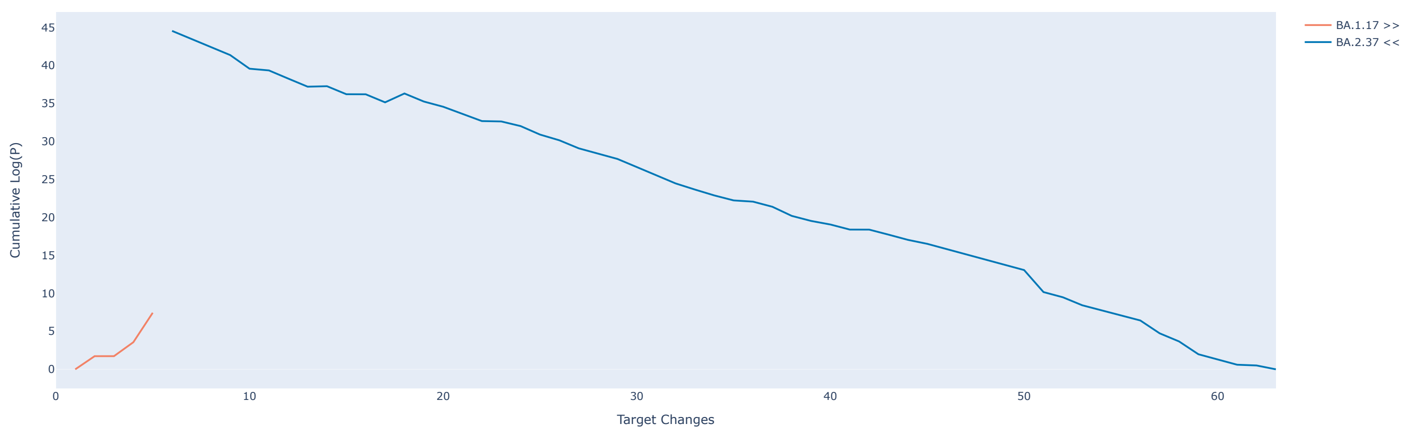


Cumulative Likelihood whole genome

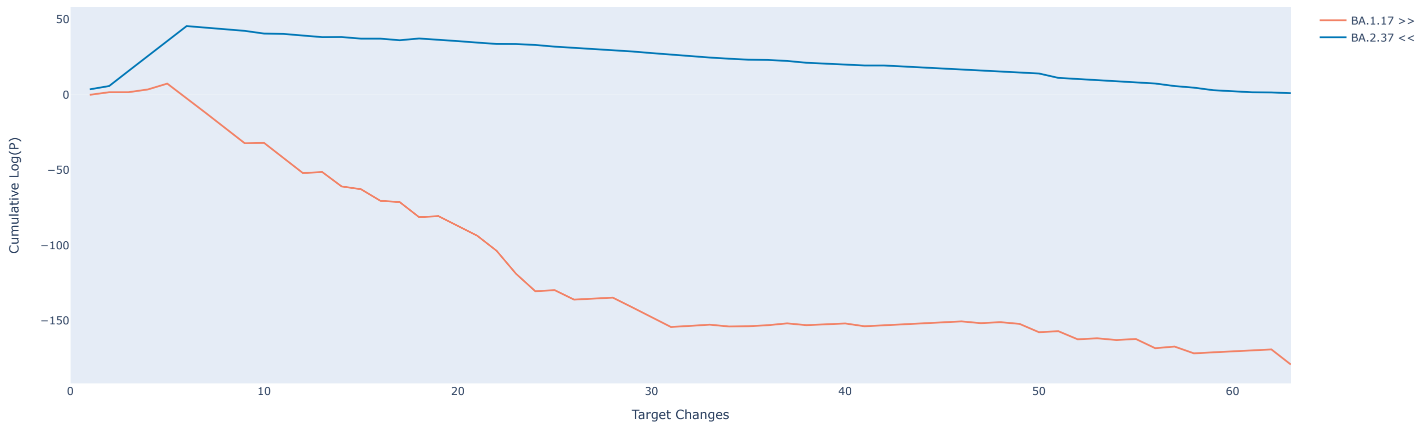


**Target sequence**

, 241_C|T, 2832_A|G, 3037_C|T, 5386_T|G, 5924_G|A, 9344_C|T, 9424_A|G, 9534_C|T, 9866_C|T, 10029_C|T, 10198_C|T, 10447_G|A, 10449_C|A, 12880_C|T, 14408_C|T, 15714_C|T, 16887_C|T, 17410_C|T, 18163_A|G, 19955_C|T, 20055_A|G, 21618_C|T, 21987_G|A, 22200_T|G, 22578_G|A, 22674_C|T, 22679_T|C, 22686_C|T, 22688_A|G, 22775_G|A, 22786_A|C, 22813_G|T, 22882_T|G, 22992_G|A, 22995_C|A, 23013_A|C, 23040_A|G, 23055_A|G, 23063_A|T, 23075_T|C, 23403_A|G, 23525_C|T, 23599_T|G, 23604_C|A, 23854_C|A, 23948_G|T, 24424_A|T, 24469_T|A, 25000_C|T, 25416_C|T, 25584_C|T, 26060_C|T, 26270_C|T, 26577_C|G, 26709_G|A, 26858_C|T, 27259_A|C, 27382_GAT|CTC, 27807_C|T, 28271_A|T, 28311_C|T, 28881_GGG|AAC, 29510_A|C

## Case 40 (1BP 5'): XW test: OK

Target: (75%) 88 samples
Number of changes: 67
GT: BA.1* + BA.2*
GT BR: 3-5
GT BR coord: 2833 - 4184
Rank L1 L2: 11 2
BC: BA.1.1.2 + BA.2.23
BC BR: 3-4
BC BR coord: 2835 - 2836
Direction L1: <<
Initial region span: 1-3,4-67
Gap history (edge excluded):
Alt. candidates: [BA.1.1], [BA.2]
Model 1BP/2BP comparison: -
Rec. model vs L1: 3.71e-298
Rec. model vs L2: 4.44e-35
Flags: Model_2BP_Bad_L1_opp

## BA.1.1.2 >>

| | num_seq | t_ch_MAX | max_CL | CL@BC_t_ch_MAX | aic | PV | PV_OK | t_ch_MAX_OK | phyl_OK |
|---|---|---|---|---|---|---|---|---|---|
| **BA.1.1.2** | 7493 | 3 | 4.471676 | NaN | NaN | NaN | * | * | * |
| **BA.1.1.16** | 1583 | 3 | 4.471013 | 4.471013 | -2.942027 | 1.000000 | * | * | |
| **BA.1.1.18** | 27122 | 3 | 4.459549 | 4.459549 | -2.919098 | 0.990050 | * | * | |
| **BA.1.1.6** | 86 | 3 | 4.452918 | 4.452918 | -2.905835 | 0.985112 | * | * | |
| **BA.1.1.12** | 1392 | 3 | 4.430911 | 4.430911 | -2.861823 | 0.960789 | * | * | |
| **BA.1.1.15** | 6410 | 3 | 4.428521 | 4.428521 | -2.857042 | 0.960789 | * | * | |
| **BA.1.1.13** | 3131 | 3 | 4.428414 | 4.428414 | -2.856828 | 0.960789 | * | * | |
| **BA.1.1.10** | 1083 | 3 | 4.427245 | 4.427245 | -2.854491 | 0.955997 | * | * | |
| **BA.1.1** | 349352 | 3 | 4.422482 | 4.422482 | -2.844964 | 0.951229 | * | * | * |
| **BA.1.1.14** | 8250 | 3 | 4.409831 | 4.409831 | -2.819662 | 0.941765 | * | * | |

## BA.2.23 <<

| | num_seq | t_ch_MAX | max_CL | CL@BC_t_ch_MAX | aic | PV | PV_OK | t_ch_MAX_OK | phyl_OK |
|---|---|---|---|---|---|---|---|---|---|
| **BA.2.23** | 3557 | 4 | 52.015892 | NaN | NaN | NaN | * | * | * |
| **BA.2** | 378689 | 4 | 48.453276 | 48.453276 | 53.093449 | 2.843882e-02 | * | * | * |
| **BA.2.33** | 389 | 21 | 39.024527 | 15.489030 | 119.021940 | 1.372126e-16 | | | |
| **BA.2.26** | 490 | 21 | 38.343641 | 10.430254 | 129.139491 | 8.706908e-19 | | | |
| **BA.2.19** | 365 | 21 | 38.140828 | 12.885593 | 124.228815 | 1.014044e-17 | | | |
| **BA.2.29** | 1418 | 21 | 37.952370 | 17.487315 | 115.025370 | 1.008815e-15 | | | |
| **BA.2.5** | 1908 | 21 | 37.543278 | 19.700802 | 110.598396 | 9.242167e-15 | | | |
| **BA.2.10** | 28641 | 21 | 37.457108 | 29.401568 | 91.196864 | 1.508101e-10 | | | |
| **BA.2.40** | 17 | 23 | 37.117851 | 11.694983 | 126.610033 | 3.084938e-18 | | | |
| **BA.2.36** | 5393 | 4 | 37.080071 | 37.080071 | 75.839858 | 3.264464e-07 | | * | |

Cumulative Likelihood per-region

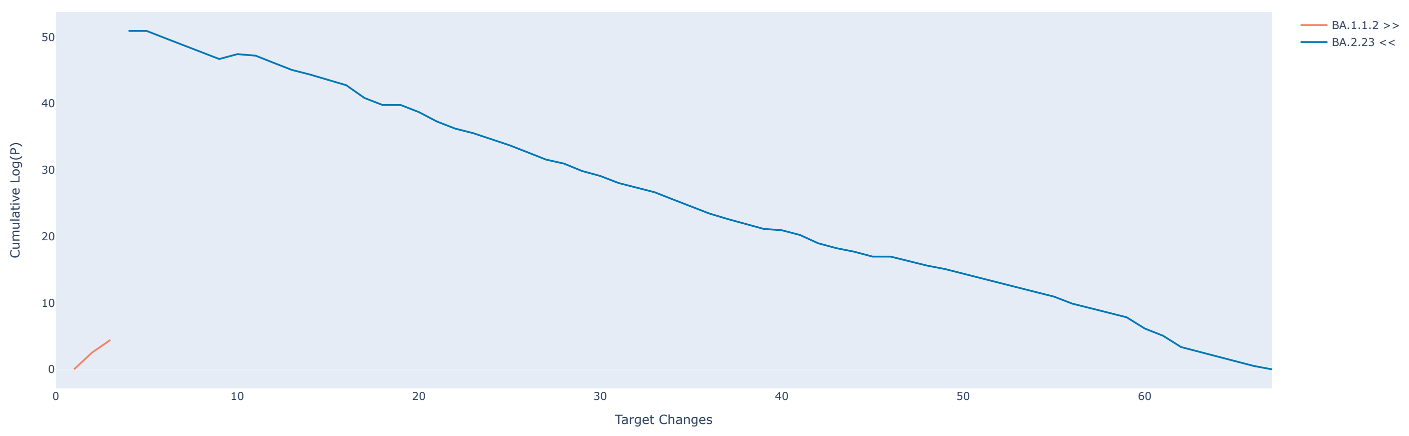


Cumulative Likelihood whole genome

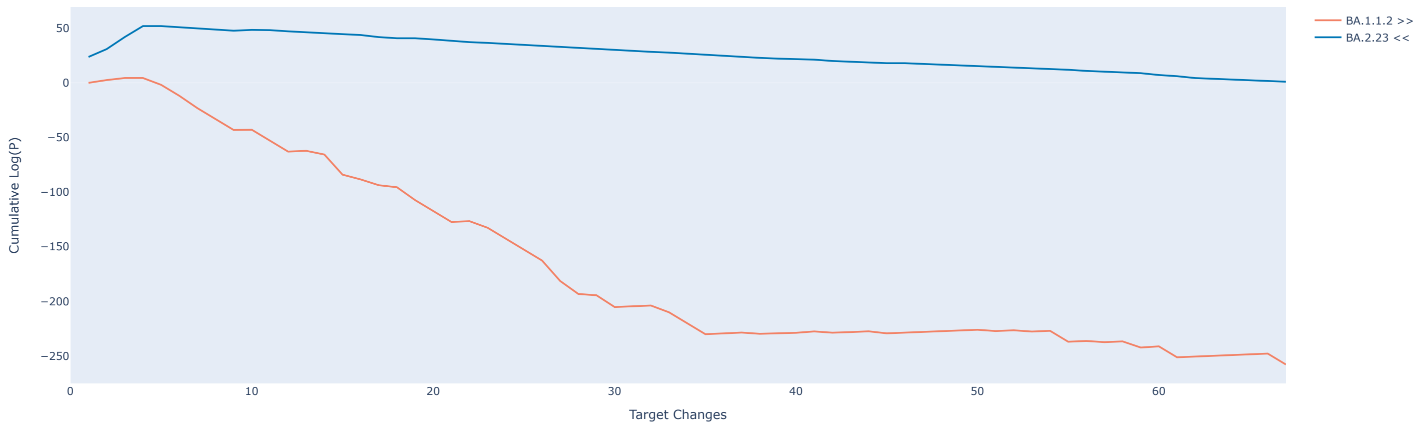


**Target sequence**

, 241_C|T, 2470_C|T, 2832_A|G, 3037_C|T, 4184_G|A, 4321_C|T, 9344_C|T, 9424_A|G, 9534_C|T, 10029_C|T, 10198_C|T, 10447_G|A, 10449_C|A, 10507_C|T, 11288_TCTGGTTTT|........., 12756_C|T, 12880_C|T, 14408_C|T, 15714_C|T, 16020_G|T, 17410_C|T, 18163_A|G, 19955_C|T, 20055_A|G, 21618_C|T, 21633_TACCCCCTG|........., 21987_G|A, 22200_T|G, 22578_G|A, 22674_C|T, 22679_T|C, 22686_C|T, 22688_A|G, 22775_G|A, 22786_A|C, 22813_G|T, 22882_T|G, 22992_G|A, 22995_C|A, 23013_A|C, 23040_A|G, 23055_A|G, 23063_A|T, 23075_T|C, 23403_A|G, 23525_C|T, 23599_T|G, 23604_C|A, 23854_C|A, 23948_G|T, 24424_A|T, 24469_T|A, 25000_C|T, 25584_C|T, 26060_C|T, 26270_C|T, 26577_C|G, 26709_G|A, 26858_C|T, 27259_A|C, 27382_GAT|CTC, 27807_C|T, 28271_A|T, 28311_C|T, 28362_GAGAACGCA|........., 28881_GGG|AAC, 29510_A|C

## Case 41 (1BP 3'): XAH    test: OK

Target: (75%) 141 samples
Number of changes: 64
GT: BA.2* + BA.1*
GT BR: 59-61
GT BR coord: 26858 - 27382
Rank L1 L2: 1 11
BC: BA.2 + BA.1.17
BC BR: 56-57
BC BR coord: 26274 - 26275
Direction L1: >>
Initial region span: 1-56,57-64
Gap history (edge excluded):
Alt. candidates: [BA.2.10], [BA.1.17.1]
Model 1BP/2BP comparison: 1BP vs 2BP: 4.46e-04
Rec. model vs L1: 2.49e-13
Rec. model vs L2: 5.04e-165
Flags: Model_1BP_Best

## BA.2 >>

| | num_seq | t_ch_MAX | max_CL | CL@BC_t_ch_MAX | aic | PV | PV_OK | t_ch_MAX_OK | phyl_OK |
|---|---|---|---|---|---|---|---|---|---|
| **BA.2** | 378689 | 56 | 44.384021 | NaN | NaN | NaN | * | * | * |
| **BA.2.10** | 28641 | 56 | 36.779101 | 36.779101 | 62.441799 | 4.979554e-04 | * | * | * |
| **BA.2.3** | 36275 | 54 | 32.742267 | 29.969484 | 76.061031 | 5.490919e-07 | | | * |
| **BA.2.56** | 3106 | 56 | 31.492262 | 31.492262 | 73.015477 | 2.510572e-06 | | * | * |
| **BA.2.20** | 634 | 56 | 28.435258 | 28.435258 | 79.129485 | 1.183050e-07 | | * | * |
| **BA.2.38** | 3718 | 35 | 27.909653 | 20.270644 | 95.458711 | 3.365027e-11 | | | * |
| **BA.2.9** | 108830 | 54 | 27.464942 | 25.480427 | 85.039147 | 6.161165e-09 | | | * |
| **BA.2.5** | 1908 | 56 | 26.289556 | 26.289556 | 83.420889 | 1.384973e-08 | | * | * |
| **BA.2.14** | 992 | 56 | 25.972809 | 25.972809 | 84.054381 | 1.010738e-08 | | * | * |
| **BA.2.37** | 2257 | 53 | 25.024332 | 24.515594 | 86.968812 | 2.347300e-09 | | | * |

## BA.1.17 <<

| | num_seq | t_ch_MAX | max_CL | CL@BC_t_ch_MAX | aic | PV | PV_OK | t_ch_MAX_OK | phyl_OK |
|---|---|---|---|---|---|---|---|---|---|
| **BA.1.17** | 27172 | 57 | 6.922200 | NaN | NaN | NaN | * | * | * |
| **BA.1.17.1** | 201 | 57 | 6.912994 | 6.912994 | 4.174011 | 0.995012 | * | * | * |
| **BA.1.1.7** | 324 | 57 | 6.751781 | 6.751781 | 4.496438 | 0.843665 | * | * | |
| **BA.1.1.11** | 1121 | 57 | 6.347258 | 6.347258 | 5.305483 | 0.562705 | * | * | |
| **BA.1.10** | 840 | 57 | 6.281969 | 6.281969 | 5.436061 | 0.527292 | * | * | |
| **BA.1.1.12** | 1392 | 57 | 6.253920 | 6.253920 | 5.492161 | 0.514274 | * | * | |
| **BA.1.1.17** | 229 | 57 | 6.239727 | 6.239727 | 5.520546 | 0.506617 | * | * | |
| **BA.1.1.18** | 27122 | 57 | 6.238343 | 6.238343 | 5.523313 | 0.506617 | * | * | |
| **BA.1.9** | 345 | 57 | 6.235109 | 6.235109 | 5.529783 | 0.504090 | * | * | |
| **BA.1.1.13** | 3131 | 57 | 6.230902 | 6.230902 | 5.538197 | 0.501576 | * | * | |

Cumulative Likelihood per-region

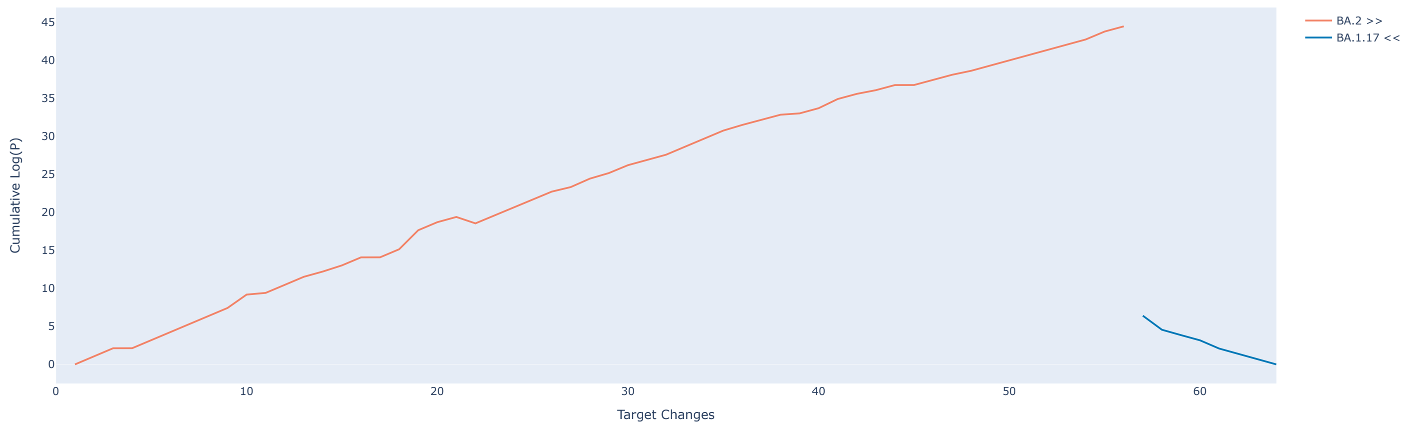


Cumulative Likelihood whole genome

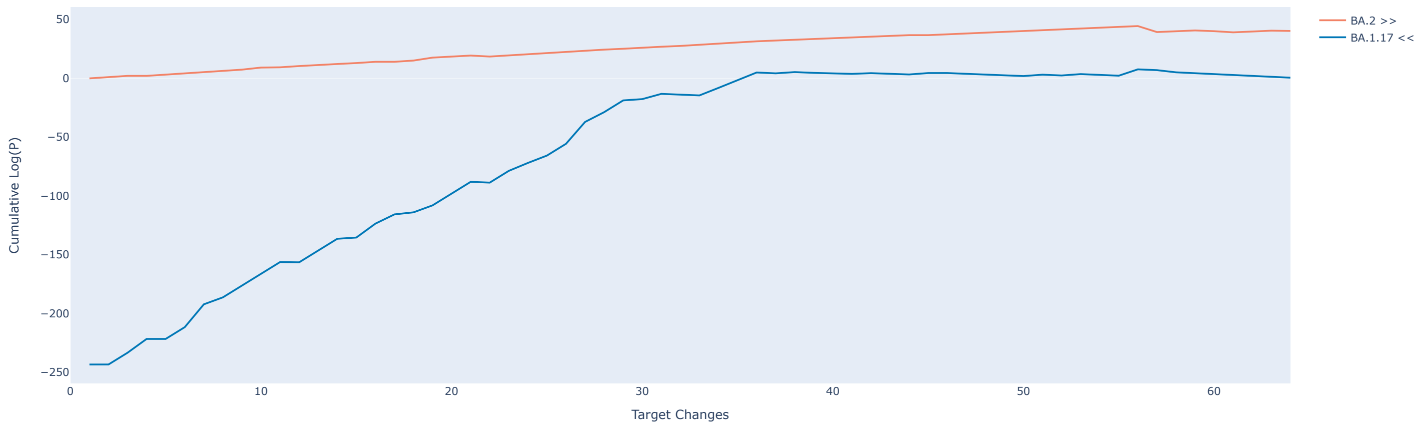


**Target sequence**

, 241_C|T, 670_T|G, 2790_C|T, 3037_C|T, 4184_G|A, 4321_C|T, 9344_C|T, 9424_A|G, 9534_C|T, 9866_C|T, 10029_C|T, 10198_C|T, 10447_G|A, 10449_C|A, 11288_TCTGGTTTT|........., 12880_C|T, 14408_C|T, 15714_C|T, 17242_G|T, 17410_C|T, 18163_A|G, 18246_C|T, 19955_C|T, 20055_A|G, 21618_C|T, 21633_TACCCCCTG|........., 21987_G|A, 22200_T|G, 22578_G|A, 22674_C|T, 22679_T|C, 22686_C|T, 22688_A|G, 22775_G|A, 22786_A|C, 22813_G|T, 22882_T|G, 22992_G|A, 22995_C|A, 23013_A|C, 23040_A|G, 23055_A|G, 23063_A|T, 23075_T|C, 23403_A|G, 23525_C|T, 23599_T|G, 23604_C|A, 23854_C|A, 23948_G|T, 24424_A|T, 24469_T|A, 25000_C|T, 25584_C|T, 26060_C|T, 26270_C|T, 26530_A|G, 26577_C|G, 26709_G|A, 27259_A|C, 27807_C|T, 28271_A|T, 28311_C|T, 28881_GGG|AAC

## Case 42 (1BP 3'): XAP    test: OK

Target: (75%) 26 samples
Number of changes: 64
GT: BA.2* + BA.1*
GT BR: 54-56
GT BR coord: 26062 - 26529
Rank L1 L2: 1 11
BC: BA.2 + BA.1.1.12
BC BR: 55-56
BC BR coord: 26274 - 26275
Direction L1: >>
Initial region span: 1-55,56-64
Gap history (edge excluded):
Alt. candidates: [BA.2.65, BA.2.34], []
Model 1BP/2BP comparison:
1BP vs 2BP: 2.13e-03
Rec. model vs L1: 2.84e-23
Rec. model vs L2: 2.04e-287
Flags: Model_1BP_Best

## BA.2 >>

| | num_seq | t_ch_MAX | max_CL | CL@BC_t_ch_MAX | aic | PV | PV_OK | t_ch_MAX_OK | phyl_OK |
|---|---|---|---|---|---|---|---|---|---|
| **BA.2** | 378689 | 55 | 41.572925 | NaN | NaN | NaN | * | * | * |
| **BA.2.65** | 1625 | 55 | 33.231503 | 33.231503 | 65.536993 | 2.375815e-04 | * | * | * |
| **BA.2.62** | 249 | 53 | 32.213901 | 26.866176 | 78.267649 | 4.088163e-07 | | | * |
| **BA.2.59** | 132 | 52 | 31.016142 | 18.769009 | 94.461983 | 1.247138e-10 | | | * |
| **BA.2.34** | 325 | 55 | 30.550038 | 30.550038 | 70.899925 | 1.628933e-05 | * | * | * |
| **BA.2.58** | 74 | 52 | 30.407347 | 26.436206 | 79.127588 | 2.659387e-07 | | | * |
| **BA.2.17** | 100 | 52 | 29.024907 | 19.110756 | 93.778489 | 1.752163e-10 | | | * |
| **BA.2.10** | 28641 | 55 | 28.486016 | 28.486016 | 75.027968 | 2.065786e-06 | | * | * |
| **BA.2.9** | 108830 | 53 | 28.007451 | 26.022936 | 79.954128 | 1.764903e-07 | | | * |
| **BA.2.3.16** | 67 | 53 | 27.913551 | 25.149591 | 81.700819 | 7.357210e-08 | | | * |

## BA.1.1.12 <<

| | num_seq | t_ch_MAX | max_CL | CL@BC_t_ch_MAX | aic | PV | PV_OK | t_ch_MAX_OK | phyl_OK |
|---|---|---|---|---|---|---|---|---|---|
| **BA.1.1.12** | 1392 | 56 | 7.786122 | NaN | NaN | NaN | * | * | * |
| **BA.1.3** | 17 | 56 | 7.769603 | 7.769603 | 2.460795 | 0.985112 | * | * | |
| **BA.1.5** | 122 | 56 | 7.763763 | 7.763763 | 2.472474 | 0.980199 | * | * | |
| **BA.1.9** | 345 | 56 | 7.759709 | 7.759709 | 2.480582 | 0.975310 | * | * | |
| **BA.1.1.13** | 3131 | 56 | 7.755146 | 7.755146 | 2.489709 | 0.970446 | * | * | |
| **BA.1.1.18** | 27122 | 56 | 7.750197 | 7.750197 | 2.499607 | 0.965605 | * | * | |
| **BD.1** | 2033 | 56 | 7.744761 | 7.744761 | 2.510477 | 0.960789 | * | * | |
| **BA.1.1.17** | 229 | 56 | 7.737327 | 7.737327 | 2.525347 | 0.951229 | * | * | |
| **BA.1.17.2** | 77688 | 56 | 7.731957 | 7.731957 | 2.536087 | 0.946485 | * | * | |
| **BA.1.1.10** | 1083 | 56 | 7.728517 | 7.728517 | 2.542966 | 0.946485 | * | * | |

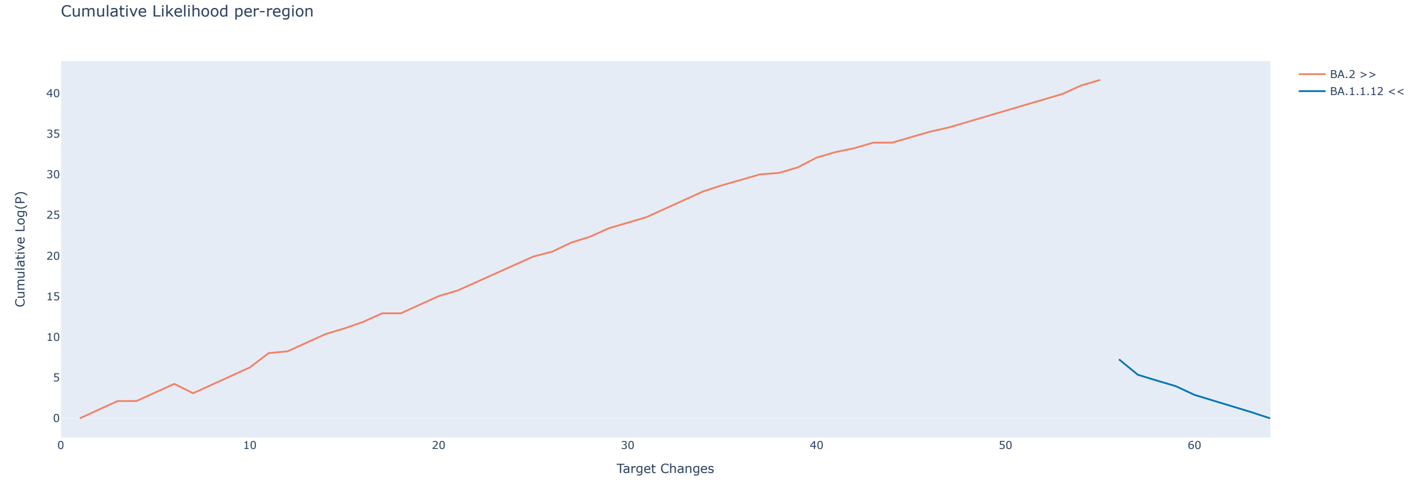


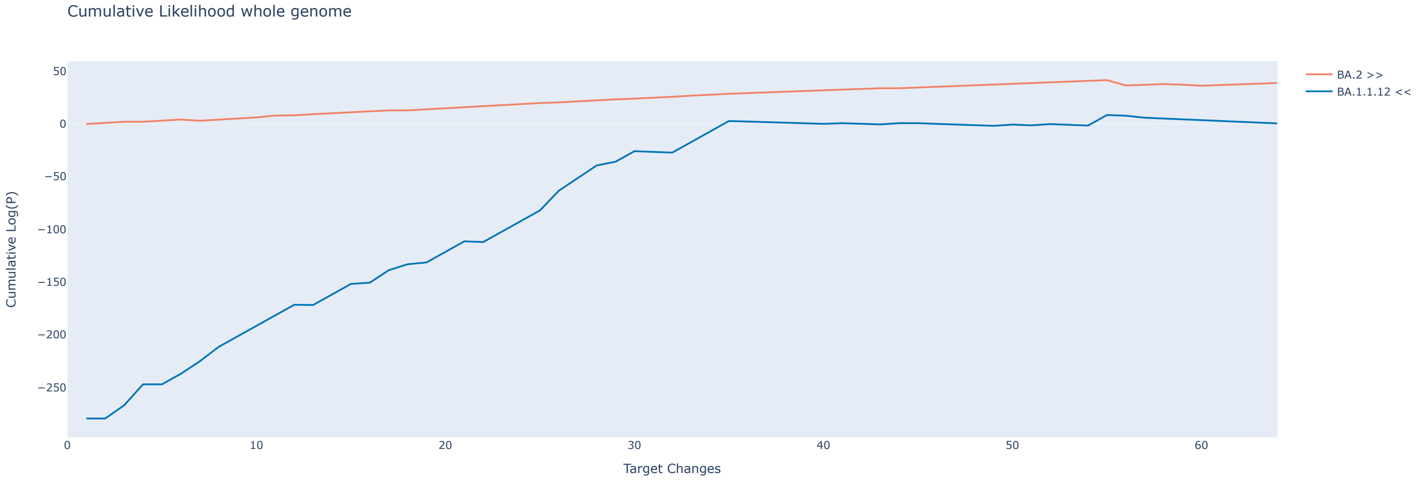


**Target sequence**

, 241_C|T, 670_T|G, 2790_C|T, 3037_C|T, 4184_G|A, 4321_C|T, 5407_C|A, 9344_C|T, 9424_A|G, 9534_C|T, 9866_C|T, 10029_C|T, 10198_C|T, 10447_G|A, 10449_C|A, 11288_TCTGGTTTT|........., 12880_C|T, 14408_C|T, 15714_C|T, 17410_C|T, 18163_A|G, 19955_C|T, 20055_A|G, 21618_C|T, 21633_TACCCCCTG|........., 21987_G|A, 22200_T|G, 22578_G|A, 22674_C|T, 22679_T|C, 22686_C|T, 22688_A|G, 22775_G|A, 22786_A|C, 22813_G|T, 22882_T|G, 22992_G|A, 22995_C|A, 23013_A|C, 23040_A|G, 23055_A|G, 23063_A|T, 23075_T|C, 23403_A|G, 23525_C|T, 23599_T|G, 23604_C|A, 23854_C|A, 23948_G|T, 24424_A|T, 24469_T|A, 25000_C|T, 25584_C|T, 26060_C|T, 26270_C|T, 26530_A|G, 26577_C|G, 26709_G|A, 27259_A|C, 27807_C|T, 28271_A|T, 28311_C|T, 28362_GAGAACGCA|........., 28881_GGG|AAC

## Case 43 (1BP 3'): XAS    test: KO

Target: (75%) 65 samples    Number of changes: 67
GT: BA.5* + BA.2*    GT BR: 40-61    GT BR coord: 23039 - 27788    Rank L1 L2: 5 11
BC: BA.4.8 + BA.2.65    BC BR: 61-62    BC BR coord: 27811 - 27812
Direction L1: >>    Initial region span: 1-61,62-67    Gap history (edge excluded):
Alt. candidates: [BA.4], []
Model 1BP/2BP comparison:   -
Rec. model vs L1: 7.66e-12    Rec. model vs L2: 2.56e-33
Flags: Model_2BP_Bad_L1_opp

## BA.4.8 >>

| | num_seq | t_ch_MAX | max_CL | CL@BC_t_ch_MAX | aic | PV | PV_OK | t_ch_MAX_OK | phyl_OK |
|---|---|---|---|---|---|---|---|---|---|
| **BA.4.8** | 75 | 61 | 50.317014 | NaN | NaN | NaN | * | * | * |
| **BA.4** | 16319 | 60 | 46.108972 | 42.582860 | 58.834281 | 4.394441e-04 | * | * | * |
| **BA.5.1.2** | 3816 | 55 | 44.215686 | 8.209350 | 127.581300 | 5.176436e-19 | | | |
| **BA.5.1** | 112751 | 55 | 44.038738 | 19.727190 | 104.545620 | 5.187186e-14 | | | |
| **BA.5** | 16505 | 55 | 43.983002 | 33.753377 | 76.493247 | 6.428111e-08 | | | |
| **BA.5.1.1** | 4748 | 53 | 42.412650 | 17.041593 | 109.916814 | 3.538760e-15 | | | |
| **BF.8** | 1864 | 55 | 41.828394 | 21.926275 | 100.147451 | 4.681442e-13 | | | |
| **BA.5.1.20** | 205 | 55 | 41.741341 | 8.618538 | 126.762924 | 7.799946e-19 | | | |
| **BA.5.2.1** | 93917 | 55 | 41.664609 | 18.061640 | 107.876720 | 9.813671e-15 | | | |
| **BA.4.1** | 26267 | 60 | 41.074274 | 37.546708 | 68.906583 | 2.844851e-06 | | * | |

## BA.2.65 <<

| | num_seq | t_ch_MAX | max_CL | CL@BC_t_ch_MAX | aic | PV | PV_OK | t_ch_MAX_OK | phyl_OK |
|---|---|---|---|---|---|---|---|---|---|
| **BA.2.65** | 1625 | 62 | 10.153498 | NaN | NaN | NaN | * | * | * |
| **BA.2.55** | 113 | 62 | 6.197908 | 6.197908 | 3.604183 | 0.019159 | * | * | |
| **BN.1.3.1** | 3278 | 62 | 5.402499 | 5.402499 | 5.195002 | 0.008609 | * | * | |
| **BA.2.3.14** | 529 | 62 | 5.333563 | 5.333563 | 5.332875 | 0.008067 | * | * | |
| **BA.2.51** | 288 | 62 | 5.185314 | 5.185314 | 5.629372 | 0.006943 | * | * | |
| **BN.1.5** | 1645 | 62 | 5.090472 | 5.090472 | 5.819057 | 0.006314 | * | * | |
| **BA.5** | 16505 | 62 | 4.738187 | 4.738187 | 6.523626 | 0.004449 | * | * | |
| **BA.2.38** | 3718 | 62 | 4.565608 | 4.565608 | 6.868784 | 0.003735 | * | * | |
| **BF.13** | 958 | 62 | 4.430240 | 2.581050 | 10.837901 | 0.000513 | * | * | |
| **BA.2.12.1** | 84020 | 62 | 4.419125 | 4.419125 | 7.161750 | 0.003231 | * | * | |

Cumulative Likelihood per-region

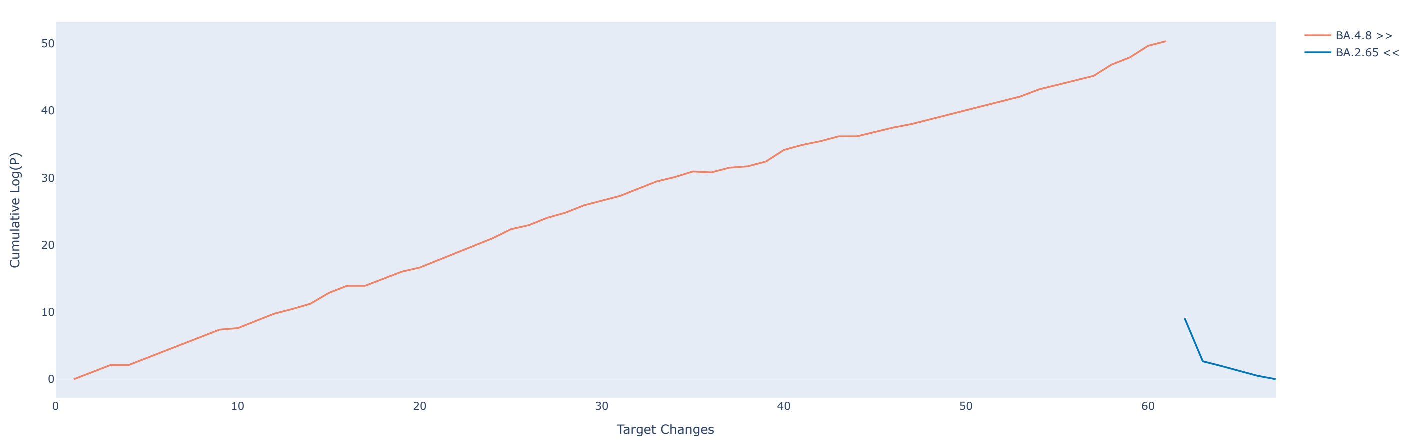


Cumulative Likelihood whole genome

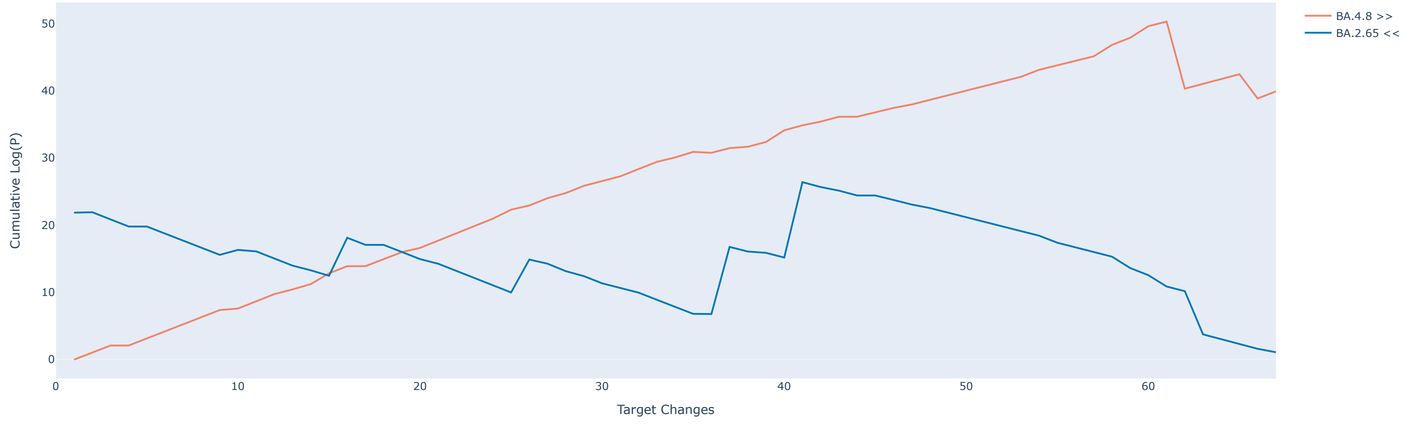


**Target sequence**

, 241_C|T, 670_T|G, 2790_C|T, 3037_C|T, 4184_G|A, 4321_C|T, 9344_C|T, 9424_A|G, 9534_C|T, 10029_C|T, 10198_C|T, 10447_G|A, 10449_C|A, 11288_TCTGGTTTT|........., 12160_G|A, 12880_C|T, 14408_C|T, 15714_C|T, 17410_C|T, 18163_A|G, 19955_C|T, 20055_A|G, 21618_C|T, 21633_TACCCCCTG|........., 21765_TACATG|......, 21987_G|A, 22200_T|G, 22578_G|A, 22674_C|T, 22679_T|C, 22686_C|T, 22688_A|G, 22775_G|A, 22786_A|C, 22813_G|T, 22917_T|G, 22992_G|A, 22995_C|A, 23013_A|C, 23018_T|G, 23055_A|G, 23063_A|T, 23075_T|C, 23403_A|G, 23525_C|T, 23599_T|G, 23604_C|A, 23854_C|A, 23948_G|T, 24424_A|T, 24469_T|A, 25000_C|T, 25584_C|T, 26060_C|T, 26270_C|T, 26577_C|G, 26709_G|A, 26858_C|T, 27259_A|C, 27382_GAT|CTC, 27807_C|T, 27945_C|T, 28271_A|T, 28311_C|T, 28362_GAGAACGCA|........., 28881_GGG|AAC, 29510_A|C

## Case 44 (1BP 3'): XAT test: KO

Target: (75%) 28 samples
Number of changes: 66
GT: BA.2.3.13 + BA.1*
GT BR: 57–59
GT BR coord: 26061 – 26529
Rank L1 L2: 1 –
BC: BA.2.3.13
BC BR:
BC BR coord:
Direction L1: >>
Initial region span: 1–66
Gap history (edge excluded):
Alt. candidates: []
Model 1BP/2BP comparison: –
Rec. model vs L1: –
Rec. model vs L2: –
Flags: NotEnoughSpaceAfterL1, SingleCandidateGenome

# BA.2.3.13 >>

| | num_seq | t_ch_MAX | max_CL | CL@BC_t_ch_MAX | aic | PV | PV_OK | t_ch_MAX_OK | phyl_OK |
|---|---|---|---|---|---|---|---|---|---|
| **BA.2.3.13** | 1294 | 66 | 70.463000 | NaN | NaN | NaN | * | * | * |
| **BA.2.3** | 36275 | 66 | 51.378356 | 50.316710 | 57.366579 | 5.197958e-09 | | * | * |
| **BA.2** | 378689 | 66 | 37.704677 | 36.642962 | 84.714075 | 6.012114e-15 | | * | * |
| **BA.2.3.16** | 67 | 60 | 34.588661 | 29.098403 | 99.803194 | 3.178889e-18 | | | |
| **BA.2.3.9** | 191 | 66 | 31.149703 | 21.612549 | 114.774901 | 1.784765e-21 | | * | |
| **BA.2.5** | 1908 | 66 | 28.320178 | 27.255640 | 103.488720 | 5.023449e-19 | | * | |
| **BA.2.12** | 2844 | 49 | 28.308752 | 25.111778 | 107.776444 | 5.880853e-20 | | | |
| **BA.2.3.10** | 230 | 66 | 28.125275 | 27.059992 | 103.880017 | 4.133468e-19 | | * | |
| **BA.2.3.1** | 1543 | 66 | 27.517876 | 26.445806 | 105.108389 | 2.234722e-19 | | * | * |
| **BA.2.9** | 108830 | 55 | 23.550675 | 20.014462 | 117.971075 | 3.603379e-22 | | | |

Cumulative Likelihood per-region

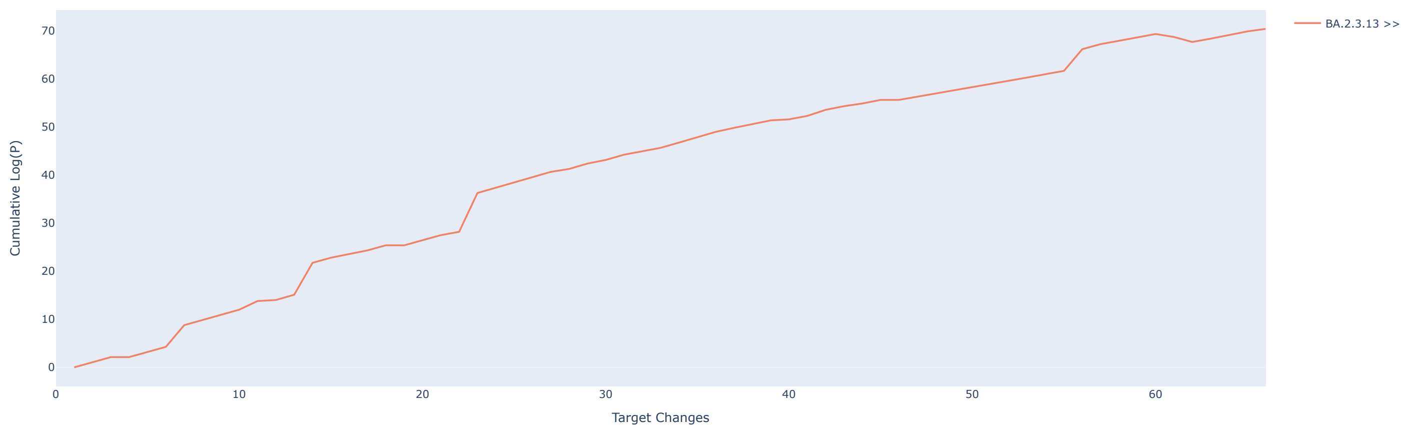


Cumulative Likelihood whole genome

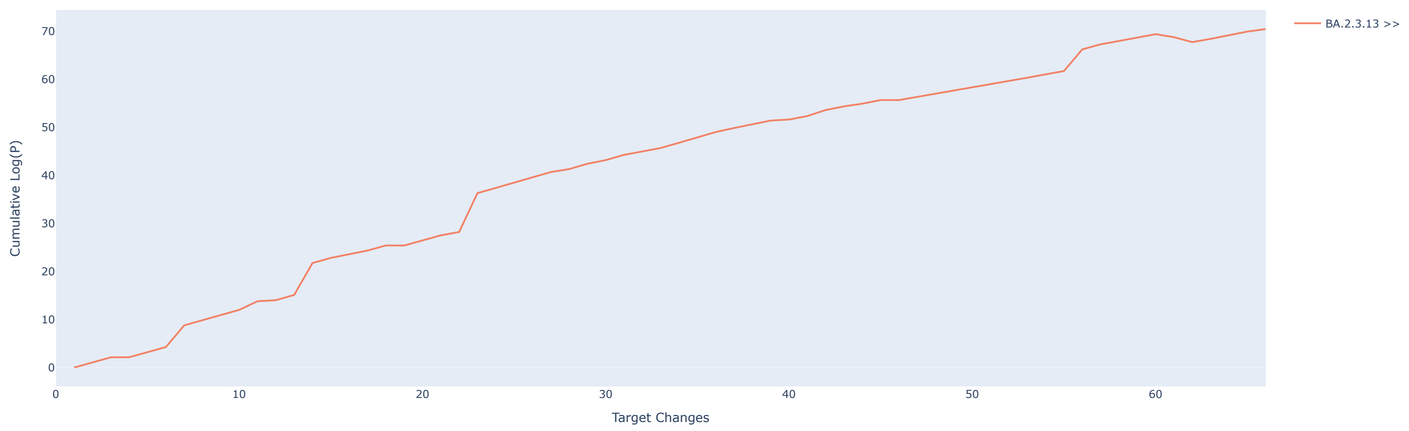


**Target sequence**

, 241_C|T, 670_T|G, 2790_C|T, 3037_C|T, 4184_G|A, 4321_C|T, 8991_C|T, 9344_C|T, 9424_A|G, 9534_C|T, 9866_C|T, 10029_C|T, 10198_C|T, 10277_C|T, 10447_G|A, 10449_C|A, 11288_TCTGGTTTT|........., 12880_C|T, 14408_C|T, 15714_C|T, 17410_C|T, 18163_A|G, 19317_C|T, 19955_C|T, 20055_A|G, 21618_C|T, 21633_TACCCCCTG|........., 21987_G|A, 22200_T|G, 22578_G|A, 22674_C|T, 22679_T|C, 22686_C|T, 22688_A|G, 22775_G|A, 22786_A|C, 22813_G|T, 22882_T|G, 22992_G|A, 22995_C|A, 23013_A|C, 23040_A|G, 23055_A|G, 23063_A|T, 23075_T|C, 23403_A|G, 23525_C|T, 23599_T|G, 23604_C|A, 23854_C|A, 23948_G|T, 24424_A|T, 24469_T|A, 25000_C|T, 25584_C|T, 25810_C|T, 26060_C|T, 26270_C|T, 26577_C|G, 26709_G|A, 27259_A|C, 27807_C|T, 28271_A|T, 28311_C|T, 28362_GAGAACGCA|........., 28881_GGG|AAC

## Case 45 (1BP 3'): XC    test: OK

Target: (75%) 4 samples    Number of changes: 36
GT: AY.29 + B.1.1.7    GT BR: 27–28    GT BR coord: 25999 – 26002    Rank L1 L2: 2 2
BC: AY.29.1 + Q.1    BC BR: 28–29    BC BR coord: 26766 – 26767
Direction L1: >>    Initial region span: 1–28,29–36    Gap history (edge excluded):
Alt. candidates: [], [B.1.1.7]
Model 1BP/2BP comparison:    –
Rec. model vs L1: 3.45e–98    Rec. model vs L2: 9.71e–268
Flags: Model_2BP_Bad_L1_opp

# AY.29.1 >>

| | num_seq | t_ch_MAX | max_CL | CL@BC_t_ch_MAX | aic | PV | PV_OK | t_ch_MAX_OK | phyl_OK |
|---|---|---|---|---|---|---|---|---|---|
| **AY.29.1** | 971 | 28 | 41.490853 | NaN | NaN | NaN | * | * | * |
| **AY.29** | 7365 | 20 | 23.460489 | 18.866568 | 46.266864 | 1.493095e-10 | | | * |
| **AY.4** | 245319 | 28 | 14.479069 | 14.479069 | 55.041863 | 1.860827e-12 | | * | |
| **B.1.617.2** | 53135 | 29 | 12.558525 | 9.298622 | 65.402755 | 1.047275e-14 | | * | * |
| **AY.118** | 9304 | 28 | 12.402287 | 12.402287 | 59.195426 | 2.324735e-13 | | * | |
| **AY.29.2** | 370 | 13 | 11.096885 | -19.051024 | 122.102047 | 5.102841e-27 | | | |
| **AY.43** | 140119 | 29 | 8.295117 | 8.016033 | 67.967934 | 2.897292e-15 | | * | |
| **AY.122** | 80528 | 28 | 7.311977 | 7.311977 | 69.376047 | 1.431577e-15 | | * | |
| **AY.45** | 4701 | 13 | 7.231363 | -7.653536 | 99.307072 | 4.535213e-22 | | | |
| **AY.112** | 7084 | 29 | 7.190383 | 5.663917 | 72.672166 | 2.763123e-16 | | * | |

# Q.1 <<

| | num_seq | t_ch_MAX | max_CL | CL@BC_t_ch_MAX | aic | PV | PV_OK | t_ch_MAX_OK | phyl_OK |
|---|---|---|---|---|---|---|---|---|---|
| **Q.1** | 3458 | 29 | 14.246280 | NaN | NaN | NaN | * | * | * |
| **B.1.1.7** | 421412 | 29 | 13.828935 | 13.828935 | 2.342130 | 6.603403e-01 | * | * | * |
| **Q.3** | 2495 | 30 | 13.681620 | 3.681620 | 22.636760 | 2.580351e-05 | * | * | |
| **Q.4** | 1080 | 30 | 13.555732 | 3.555732 | 22.888535 | 2.277152e-05 | * | * | |
| **Q.2** | 1179 | 33 | 6.484080 | -5.062444 | 40.124888 | 4.129952e-09 | | | |
| **Q.8** | 186 | 30 | 3.710475 | -6.289525 | 42.579049 | 1.207154e-09 | | * | |
| **Q.6** | 44 | 32 | 2.452070 | -14.925662 | 59.851323 | 2.146001e-13 | | | |

Cumulative Likelihood per-region

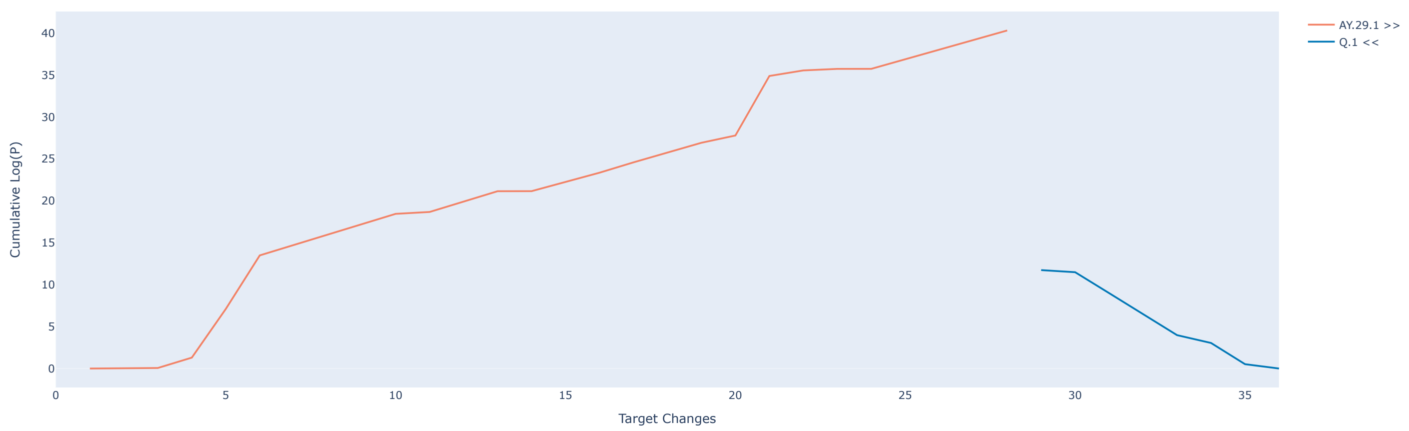


Cumulative Likelihood whole genome

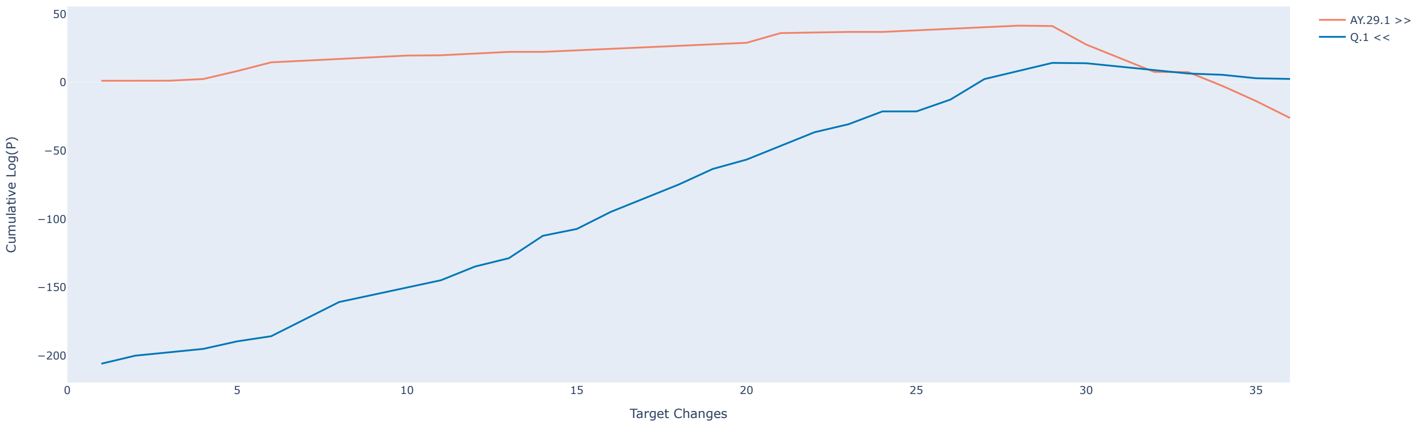


**Target sequence**

, 210_G|T, 241_C|T, 3037_C|T, 4181_G|T, 5239_C|T, 5514_T|C, 6402_C|T, 7124_C|T, 8986_C|T, 9053_G|T, 10029_C|T, 11201_A|G, 11332_A|G, 14408_C|T, 15451_G|A, 16466_C|T, 19220_C|T, 21618_C|G, 21846_C|T, 22029_AGTTCA|......, 22081_G|T, 22917_T|G, 22995_C|A, 23403_A|G, 23604_C|G, 24410_G|A, 25469_C|T, 26767_T|C, 27390_G|T, 27972_C|T, 28048_G|T, 28111_A|G, 28273_A|., 28280_GAT|CTA, 28881_GGG|AAC, 28977_C|T

## Case 46 (1BP 3'): XP        test: KO

Target: (75%) 12 samples
GT: BA.1.1* + BA.2*
BC: BA.1.1
Direction L1: >>
Alt. candidates: []
Model 1BP/2BP comparison: -
Rec. model vs L1: -
Flags: NotEnoughSpaceAfterL1, SingleCandidateGenome

Number of changes: 66
GT BR: 58-65
BC BR:
Initial region span: 1-64
GT BR coord: 27384 - 29510
BC BR coord:
Gap history (edge excluded):
Rank L1 L2: 1 -
Rec. model vs L2: -

## BA.1.1 >>

| | num_seq | t_ch_MAX | max_CL | CL@BC_t_ch_MAX | aic | PV | PV_OK | t_ch_MAX_OK | phyl_OK |
|---|---|---|---|---|---|---|---|---|---|
| **BA.1.1** | 349352 | 64 | 55.864614 | NaN | NaN | NaN | * | * | * |
| **BA.1.1.13** | 3131 | 46 | 53.895287 | 20.819875 | 112.360250 | 2.924418e-07 | | | * |
| **BA.1.1.16** | 1583 | 46 | 51.587060 | 14.118913 | 125.762175 | 3.599702e-10 | | | * |
| **BA.1.1.18** | 27122 | 46 | 49.937710 | 22.235119 | 109.529761 | 1.203833e-06 | | | * |
| **BA.1.1.6** | 86 | 46 | 49.350156 | 3.913662 | 146.172676 | 1.331347e-14 | | | * |
| **BA.1.1.2** | 7493 | 46 | 48.337401 | 22.295545 | 109.408911 | 1.278274e-06 | | | * |
| **BA.1.1.10** | 1083 | 46 | 47.975980 | 20.837157 | 112.325686 | 2.968615e-07 | | | * |
| **BA.1.1.14** | 8250 | 46 | 47.134429 | 22.759660 | 108.480681 | 2.035030e-06 | | | * |
| **BA.1.1.12** | 1392 | 46 | 46.099131 | 16.982429 | 120.035142 | 6.285628e-09 | | | * |
| **BA.1.1.8** | 571 | 46 | 44.492006 | 16.204311 | 121.591378 | 2.895813e-09 | | | * |

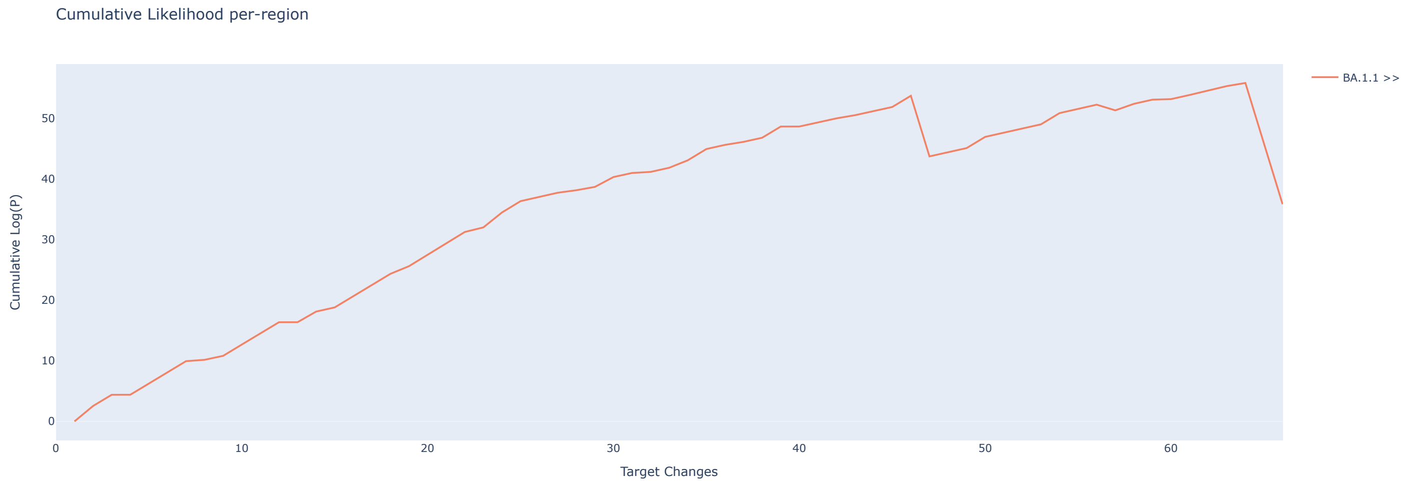


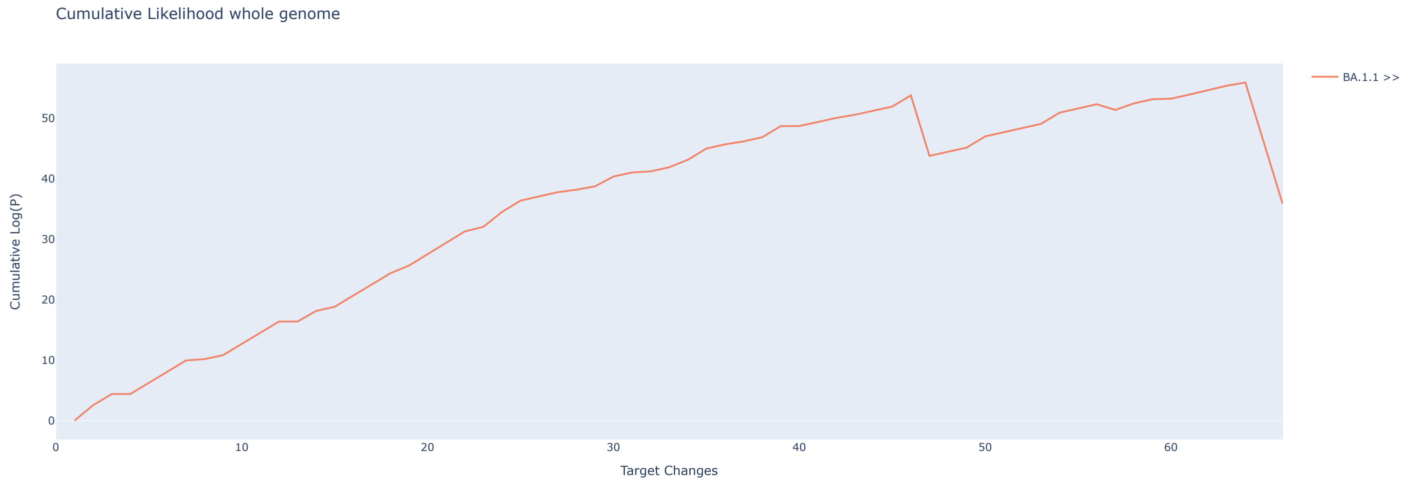


**Target sequence**

, 241_C|T, 2470_C|T, 2832_A|G, 3037_C|T, 5386_T|G, 6513_GTT|..., 8393_G|A, 10029_C|T, 10449_C|A, 11286_TGTCTGGTT|........., 11537_A|G, 13195_T|C, 14408_C|T, 15240_C|T, 18163_A|G, 21762_C|., 21764_A|., 21767_CATG|...., 21846_C|T, 21987_GTGTTTATT|........., 22194_ATT|..., 22205_.........|AGCCAGAAG, 22578_G|A, 22599_G|A, 22673_TC|CT, 22679_T|C, 22686_C|T, 22813_G|T, 22882_T|G, 22898_G|A, 22992_G|A, 22995_C|A, 23013_A|C, 23040_A|G, 23048_G|A, 23055_A|G, 23063_A|T, 23075_T|C, 23202_C|A, 23403_A|G, 23525_C|T, 23599_T|G, 23604_C|A, 23854_C|A, 23948_G|T, 24130_C|A, 24190_A|C, 24424_A|T, 24469_T|A, 24503_C|T, 25000_C|T, 25584_C|T, 26270_C|T, 26530_A|G, 26577_C|G, 26709_G|A, 26880_C|A, 27259_A|C, 27807_C|T, 28254_A|., 28271_A|T, 28311_C|T, 28362_GAGAACGCA|........., 28881_GGG|AAC, 29510_A|C, 29734_GAGGCCACGCGGAGTACGATCGAGTG|..........................

## Case 47 (1BP 3'): XT    test: OK

Target: (75%) 11 samples
GT: BA.2* + BA.1*
BC: BA.2 + BA.1.22
Direction L1: >>
Alt. candidates: [BA.2.10, BA.2.65, BA.2.36], []
Model 1BP/2BP comparison:
Rec. model vs L1: 1.67e-29
Flags: Model_1BP_Best

Number of changes: 61
GT BR: 52-54
BC BR: 53-54
Initial region span: 1-53,54-61
1BP vs 2BP: 7.65e-04
Rec. model vs L2: 1.96e-260

GT BR coord: 26061 - 26529
BC BR coord: 26274 - 26275
Gap history (edge excluded):

Rank L1 L2: 1 11

## BA.2 >>

| | num_seq | t_ch_MAX | max_CL | CL@BC_t_ch_MAX | aic | PV | PV_OK | t_ch_MAX_OK | phyl_OK |
|---|---|---|---|---|---|---|---|---|---|
| **BA.2** | 378689 | 53 | 34.251793 | NaN | NaN | NaN | * | * | * |
| **BA.2.6** | 1333 | 50 | 29.842479 | 24.952702 | 86.094596 | 9.188250e-05 | * | | * |
| **BA.2.10** | 28641 | 53 | 29.196695 | 29.196695 | 77.606610 | 6.377367e-03 | * | * | * |
| **BA.2.9** | 108830 | 51 | 27.274634 | 25.290118 | 85.419763 | 1.284463e-04 | * | | * |
| **BA.2.65** | 1625 | 53 | 24.881644 | 24.881644 | 86.236713 | 8.524339e-05 | * | * | * |
| **BA.2.36** | 5393 | 53 | 24.541255 | 24.541255 | 86.917489 | 6.067371e-05 | * | * | * |
| **BA.5** | 16505 | 53 | 22.570052 | 22.570052 | 90.859897 | 8.461365e-06 | | * | |
| **BA.2.12** | 2844 | 45 | 22.508404 | 21.458108 | 93.083783 | 2.788519e-06 | | | * |
| **BA.5.1** | 112751 | 33 | 21.211785 | 19.010990 | 97.978020 | 2.406313e-07 | | | |
| **BA.2.38** | 3718 | 33 | 20.611766 | 11.483329 | 113.033342 | 1.298034e-10 | | | * |

## BA.1.22 <<

| | num_seq | t_ch_MAX | max_CL | CL@BC_t_ch_MAX | aic | PV | PV_OK | t_ch_MAX_OK | phyl_OK |
|---|---|---|---|---|---|---|---|---|---|
| **BA.1.22** | 57 | 54 | 7.049052 | NaN | NaN | NaN | * | * | * |
| **BA.1.5** | 122 | 54 | 6.376220 | 6.376220 | 5.247560 | 0.509156 | * | * | |
| **BA.1.1.12** | 1392 | 54 | 6.368152 | 6.368152 | 5.263696 | 0.506617 | * | * | |
| **BA.1.1.13** | 3131 | 54 | 6.352696 | 6.352696 | 5.294608 | 0.499074 | * | * | |
| **BD.1** | 2033 | 54 | 6.343377 | 6.343377 | 5.313246 | 0.494109 | * | * | |
| **BA.1.17.2** | 77688 | 54 | 6.339704 | 6.339704 | 5.320591 | 0.491644 | * | * | |
| **BA.1.9** | 345 | 54 | 6.328817 | 6.328817 | 5.342366 | 0.486752 | * | * | |
| **BA.1.1.18** | 27122 | 54 | 6.319569 | 6.319569 | 5.360861 | 0.481909 | * | * | |
| **BA.1.1.10** | 1083 | 54 | 6.319121 | 6.319121 | 5.361758 | 0.481909 | * | * | |
| **BA.1.3** | 17 | 54 | 6.315387 | 6.315387 | 5.369226 | 0.479505 | * | * | |

Cumulative Likelihood per-region

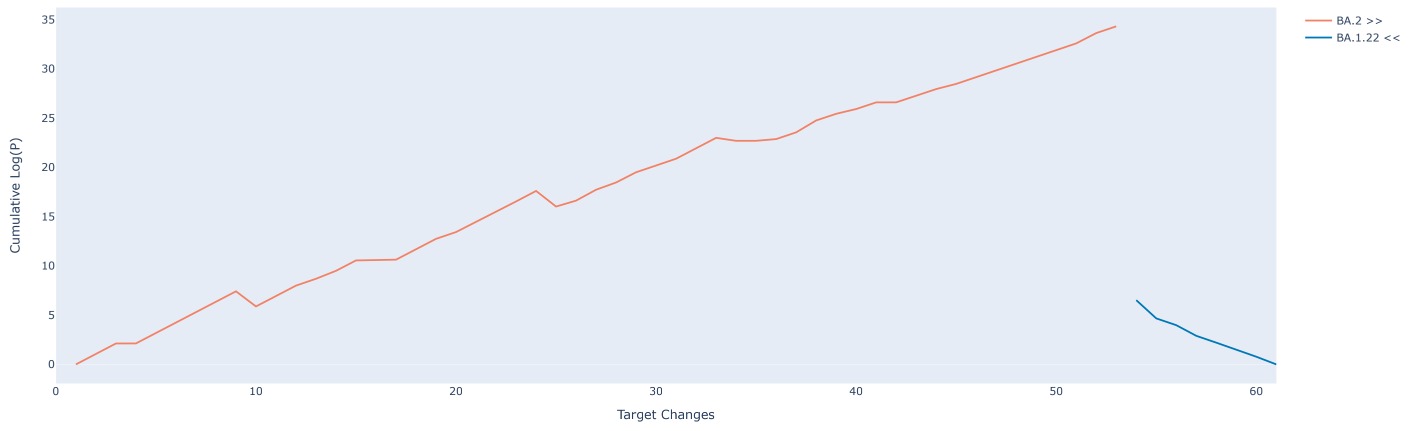


Cumulative Likelihood whole genome

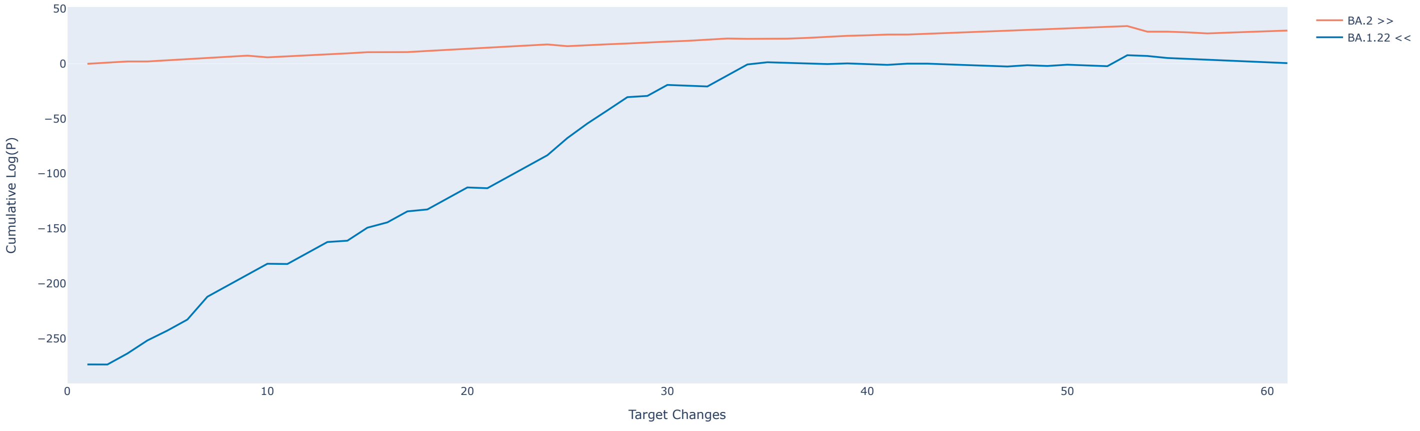


**Target sequence**

, 241_C|T, 670_T|G, 2790_C|T, 3037_C|T, 4184_G|A, 4321_C|T, 9344_C|T, 9424_A|G, 9534_C|T, 10029_C|T, 10198_C|T, 10447_G|A, 10449_C|A, 11288_TCTGGTTTT|........., 12880_C|T, 13994_C|T, 14408_C|T, 15714_C|T, 17410_C|T, 18163_A|G, 19955_C|T, 20055_A|G, 21618_C|T, 21633_TACCCCCTG|........., 21786_G|T, 21987_G|A, 22200_T|G, 22578_G|A, 22674_C|T, 22679_T|C, 22686_C|T, 22688_A|G, 22775_G|A, 22813_G|T, 22992_G|A, 22995_C|A, 23013_A|C, 23040_A|G, 23055_A|G, 23063_A|T, 23075_T|C, 23403_A|G, 23525_C|T, 23599_T|G, 23604_C|A, 23854_C|A, 23948_G|T, 24424_A|T, 24469_T|A, 25000_C|T, 25584_C|T, 26060_C|T, 26270_C|T, 26530_A|G, 26709_G|A, 27259_A|C, 27807_C|T, 28271_A|T, 28311_C|T, 28362_GAGAACGCA|........., 28881_GGG|AAC

## Case 48 (2BP): XAC        test: OK

Target: (75%) 33 samples    Number of changes: 68
GT: BA.2* + BA.1* + BA.2*    GT BR: 56–58, 62–68    GT BR coord: 25812 – 26060, 27384 – 29510    Rank L1 L2: 3 11 11
BC: BA.2.3 + BA.1.1.16 + BA.2.3    BC BR: 56–57, 62–63    BC BR coord: 25818 – 25819, 27381 – 27382
Direction L1: >>    Initial region span: 1–56,57–62,63–68    Gap history (edge excluded): 56–63 ->
Alt. candidates: [BA.2.3.1], []
Model 1BP/2BP comparison:    2BP vs 1BP: 1.45e–06
Rec. model vs L1: 1.38e–19    Rec. model vs L2: 7.61e–288
Flags: Model_2BP_Best

## BA.2.3 >>

| | num_seq | t_ch_MAX | max_CL | CL@BC_t_ch_MAX | aic | PV | PV_OK | t_ch_MAX_OK | phyl_OK |
|---|---|---|---|---|---|---|---|---|---|
| **BA.2.3** | 36275 | 56 | 47.870494 | NaN | NaN | NaN | * | * | * |
| **BA.2.3.1** | 1543 | 56 | 42.290363 | 46.773308 | 44.453384 | 4.148535e-03 | * | * | * |
| **BA.2** | 378689 | 54 | 37.302460 | 39.694164 | 58.611671 | 3.492125e-06 | | | * |
| **BA.2.12** | 2844 | 48 | 37.111011 | 25.186906 | 87.626188 | 1.743721e-12 | | | |
| **BA.2.3.16** | 67 | 54 | 36.915568 | 35.882092 | 66.235816 | 7.695846e-08 | | | * |
| **BA.2.3.9** | 191 | 53 | 34.667408 | 31.376876 | 75.246247 | 8.506673e-10 | | | * |
| **BA.2.3.2** | 444 | 54 | 34.467282 | 33.419830 | 71.160340 | 6.574943e-09 | | | * |
| **BA.2.56** | 3106 | 55 | 34.165303 | 34.979709 | 68.040582 | 3.128898e-08 | | * | |
| **BA.2.9** | 108830 | 54 | 32.897865 | 30.128282 | 77.743435 | 2.449419e-10 | | | |
| **BA.2.65** | 1625 | 54 | 30.459820 | 31.721055 | 74.557890 | 1.201134e-09 | | | |

## BA.1.1.16 >>

| | num_seq | t_ch_MAX | max_CL | CL@BC_t_ch_MAX | aic | PV | PV_OK | t_ch_MAX_OK | phyl_OK |
|---|---|---|---|---|---|---|---|---|---|
| **BA.1.1.16** | 1583 | 62 | 6.401810 | NaN | NaN | NaN | * | * | * |
| **BA.1.1.12** | 1392 | 62 | 5.968640 | 5.968640 | 14.062719 | 6.505091e-01 | * | * | |
| **A.24** | 10 | 57 | 5.840761 | -44.159239 | 114.318478 | 1.101719e-22 | | | |
| **BA.1.19** | 1453 | 62 | 5.384230 | 5.384230 | 15.231541 | 3.624024e-01 | * | * | |
| **B.1.565** | 719 | 57 | 5.229047 | -44.770953 | 115.541907 | 5.986202e-23 | | | |
| **B.1.1.374** | 158 | 57 | 5.061753 | -54.843490 | 135.686980 | 2.521358e-27 | | | |
| **P.1.15** | 2234 | 57 | 4.953586 | -41.520524 | 109.041047 | 1.543862e-21 | | | |
| **BF.16** | 246 | 57 | 4.547557 | -19.416069 | 64.832139 | 6.147350e-12 | | | |
| **CM.6.1** | 25 | 57 | 4.231323 | -6.974416 | 39.948833 | 1.553500e-06 | | | |
| **BA.1.14.1** | 2183 | 62 | 4.082354 | 4.082354 | 17.835291 | 9.827359e-02 | * | * | |

## BA.2.3 <<

| | num_seq | t_ch_MAX | max_CL | CL@BC_t_ch_MAX | aic | PV | PV_OK | t_ch_MAX_OK | phyl_OK |
|---|---|---|---|---|---|---|---|---|---|
| **BA.2.3** | 36275 | 63 | 4.387944 | None | None | None | * | * | * |

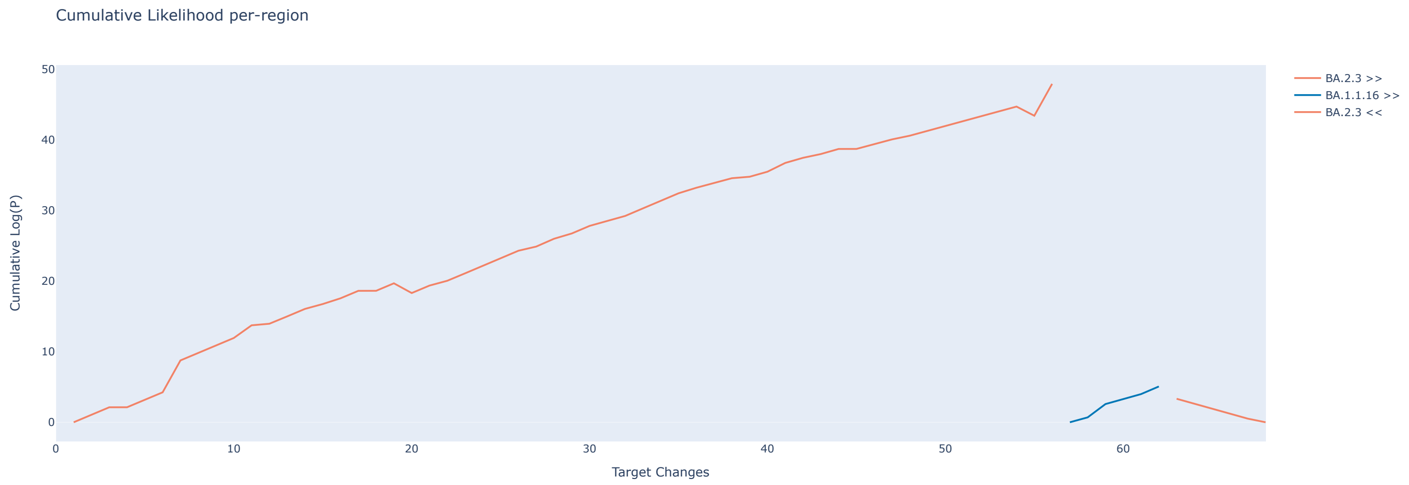


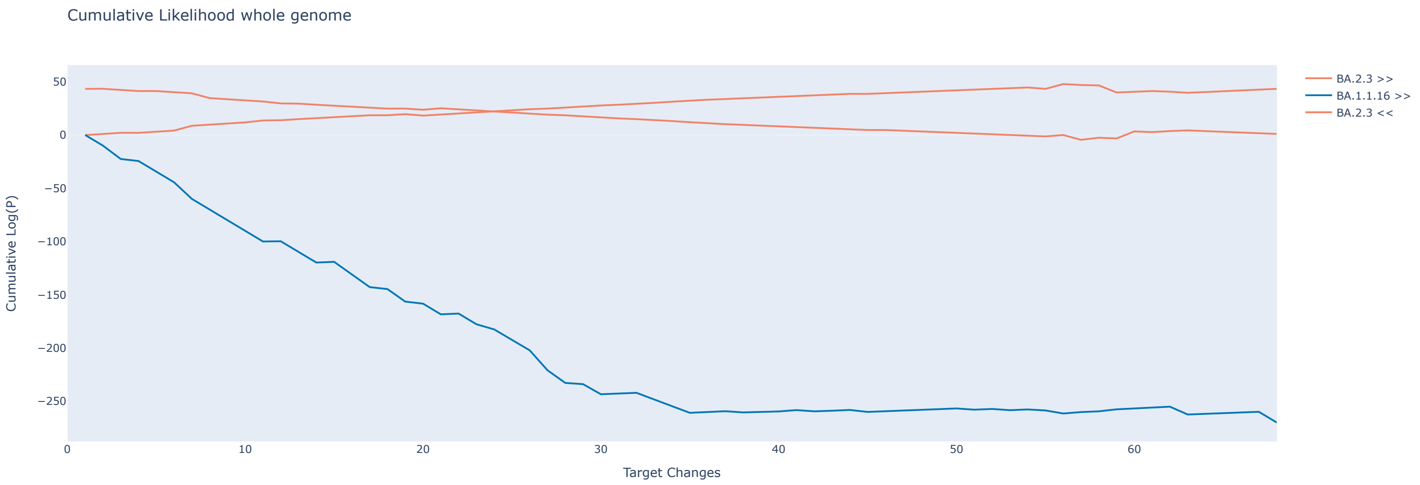


**Target sequence**

, 241_C|T, 670_T|G, 2790_C|T, 3037_C|T, 4184_G|A, 4321_C|T, 8991_C|T, 9344_C|T, 9424_A|G, 9534_C|T, 9866_C|T, 10029_C|T, 10198_C|T, 10447_G|A, 10449_C|A, 11288_TCTGGTTTT|........., 12880_C|T, 14408_C|T, 15714_C|T, 15720_C|T, 17410_C|T, 18163_A|G, 19955_C|T, 20055_A|G, 21618_C|T, 21633_TACCCCCTG|........., 21987_G|A, 22200_T|G, 22578_G|A, 22674_C|T, 22679_T|C, 22686_C|T, 22688_A|G, 22775_G|A, 22786_A|C, 22813_G|T, 22882_T|G, 22992_G|A, 22995_C|A, 23013_A|C, 23040_A|G, 23055_A|G, 23063_A|T, 23075_T|C, 23403_A|G, 23525_C|T, 23599_T|G, 23604_C|A, 23854_C|A, 23948_G|T, 24424_A|T, 24469_T|A, 25000_C|T, 25584_C|T, 25731_C|T, 25810_C|T, 26028_C|T, 26270_C|T, 26530_A|G, 26577_C|G, 26709_G|A, 27259_A|C, 27807_C|T, 28271_A|T, 28311_C|T, 28362_GAGAACGCA|........., 28881_GGG|AAC, 29510_A|C

## Case 49 (2BP): XAK

## test: KO

Target: (75%) 110 samples

Number of changes: 69

GT: BA.2* + BA.1* + BA.2*
BC: BA.2
Direction L1: <<
Alt. candidates: []
Model 1BP/2BP comparison:
Rec. model vs L1: -
Flags: Model_1BP_L1eqL2, Model_2BP_Bad_L2, SingleCandidateGenome

GT BR: 20-23, 23-24
BC BR:
Initial region span: 30-69
-
Rec. model vs L2: -

GT BR coord: 13194 - 15241, 21617 - 21763
BC BR coord:
Gap history (edge excluded):

Rank L1 L2: 1 - -

## BA.2 <<

| | num_seq | t_ch_MAX | max_CL | CL@BC_t_ch_MAX | aic | PV | PV_OK | t_ch_MAX_OK | phyl_OK |
|---|---|---|---|---|---|---|---|---|---|
| **BA.2** | 378689 | 30 | 19.474959 | NaN | NaN | NaN | * | * | * |
| **BA.2.9** | 108830 | 39 | 14.843899 | -10.098510 | 256.197019 | 2.523586e-08 | | | * |
| **BA.2.3** | 36275 | 30 | 13.962685 | -10.873294 | 257.746587 | 1.162625e-08 | | * | * |
| **BY.1** | 683 | 58 | 12.697358 | -74.856436 | 385.712872 | 1.902299e-36 | | | * |
| **BA.2.75** | 1935 | 30 | 11.937729 | -54.499890 | 344.999780 | 1.316264e-27 | | * | * |
| **BA.2.12.1** | 84020 | 55 | 11.764788 | -28.540269 | 293.080537 | 2.475300e-16 | | | * |
| **BN.1.7** | 411 | 38 | 11.541585 | -116.453569 | 468.907138 | 1.631655e-54 | | | * |
| **BN.1.5** | 1645 | 30 | 10.512018 | -86.278049 | 408.556099 | 2.077133e-41 | | * | * |
| **BA.2.52** | 627 | 39 | 10.112739 | -65.691869 | 367.383737 | 1.817975e-32 | | | * |
| **BA.4.1** | 26267 | 42 | 9.782419 | -22.785379 | 281.570759 | 7.816144e-14 | | | |

Cumulative Likelihood per-region

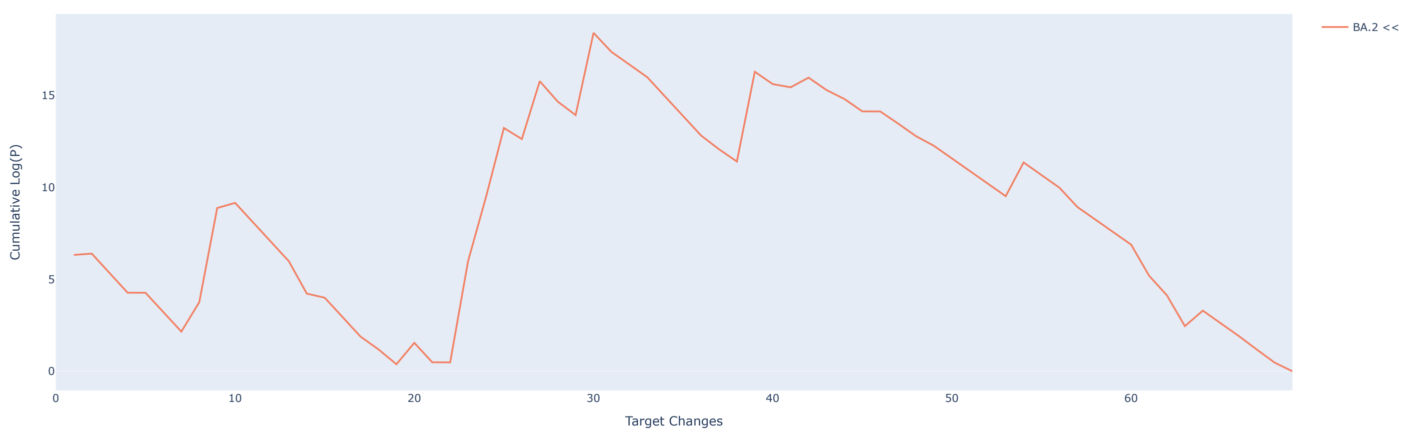


Cumulative Likelihood whole genome

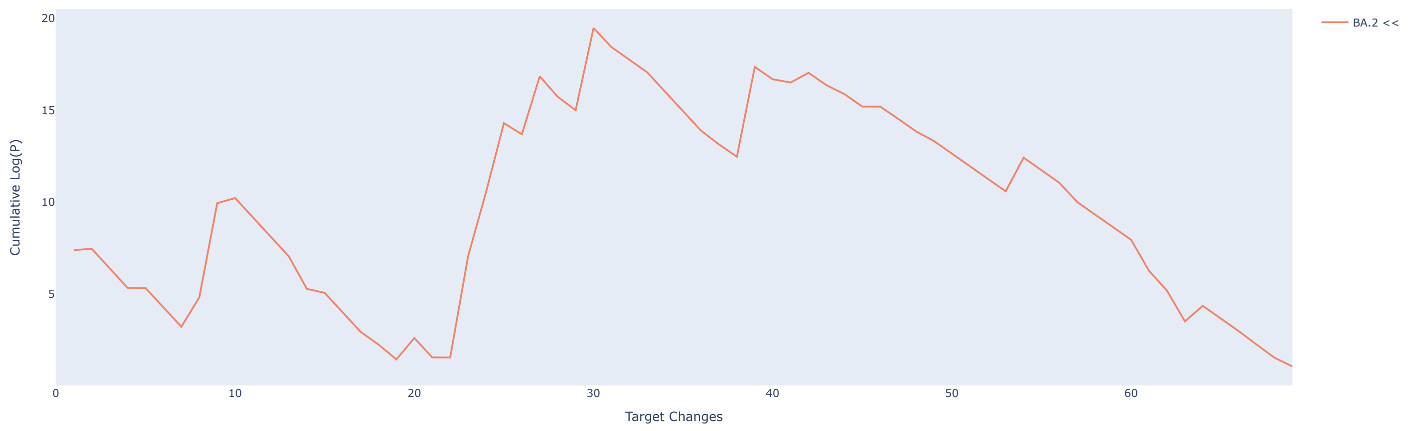


**Target sequence**

, 241_C|T, 670_T|G, 2790_C|T, 3037_C|T, 4184_G|A, 4321_C|T, 4927_C|T, 5386_T|G, 7834_C|T, 9344_C|T, 9424_A|G, 9534_C|T, 9866_C|T, 10029_C|T, 10198_C|T, 10447_G|A, 10449_C|A, 11288_TCTGGTTTT|........., 12049_C|T, 12880_C|T, 14408_C|T, 15240_C|T, 18163_A|G, 21765_TACATG|......, 21987_G|A, 22001_A|G, 22200_T|G, 22578_G|A, 22599_G|A, 22674_C|T, 22679_T|C, 22686_C|T, 22688_A|G, 22775_G|A, 22786_A|C, 22813_G|T, 22882_T|G, 22942_T|G, 22992_G|A, 22995_C|A, 23013_A|C, 23055_A|G, 23063_A|T, 23075_T|C, 23403_A|G, 23525_C|T, 23599_T|G, 23604_C|A, 23854_C|A, 23948_G|T, 24424_A|T, 24469_T|A, 24863_C|T, 25000_C|T, 25584_C|T, 26060_C|T, 26270_C|T, 26577_C|G, 26709_G|A, 26858_C|T, 27259_A|C, 27382_GAT|CTC, 27507_A|G, 27807_C|T, 28271_A|T, 28311_C|T, 28362_GAGAACGCA|........., 28881_GGG|AAC, 29510_A|C

## Case 50 (2BP): XAW

## test: KO

Target: (75%) 28 samples
GT: AY.122 + BA.2* + AY.122
BC: AY.122 + BQ.1.12 + AY.122
Direction L1: >>
Alt. candidates: [], []
Model 1BP/2BP comparison:
Rec. model vs L1: 8.83e-102
Flags: Model_2BP_Best

Number of changes: 103
GT BR: 48-53, 91-93
BC BR: 51-52, 97-98
Initial region span: 1-48,96-97,98-103
2BP vs 1BP: 9.82e-83
Rec. model vs L2: 0.00e+00

GT BR coord: 22035 - 22588, 28270 - 28312
BC BR coord: 22291 - 22292, 28880 - 28881
Gap history (edge excluded): 48-98 -> 48-96

Rank L1 L2: 1 11 1

## AY.122 >>

| | num_seq | t_ch_MAX | max_CL | CL@BC_t_ch_MAX | aic | PV | PV_OK | t_ch_MAX_OK | phyl_OK |
|---|---|---|---|---|---|---|---|---|---|
| **AY.122** | 80528 | 48 | 19.968974 | NaN | NaN | NaN | * | * | * |
| **AY.28** | 280 | 3 | 6.864636 | -378.477802 | 942.955603 | 7.921939e-172 | | | |
| **AY.117** | 7391 | 3 | 4.769573 | -243.151582 | 672.303163 | 4.697900e-113 | | | |
| **AY.124.1** | 562 | 3 | 3.836279 | -309.733224 | 805.466448 | 5.680750e-142 | | | |
| **AY.99.2** | 14345 | 5 | 2.768780 | -148.744584 | 483.489167 | 4.693257e-72 | | | |
| **AY.43.1** | 697 | 3 | 2.732541 | -303.742955 | 793.485910 | 2.268974e-139 | | | |
| **AY.42** | 16868 | 3 | 2.717974 | -182.997481 | 551.994963 | 6.264527e-87 | | | |
| **AY.34.1** | 3230 | 3 | 2.699284 | -211.808617 | 609.617234 | 1.917312e-99 | | | |
| **AY.33** | 10326 | 3 | 2.686574 | -136.373318 | 458.746637 | 1.105852e-66 | | | |
| **AY.25** | 47119 | 37 | 2.585180 | -31.645088 | 249.290176 | 3.367892e-21 | | | |

## BQ.1.12 <<

| | num_seq | t_ch_MAX | max_CL | CL@BC_t_ch_MAX | aic | PV | PV_OK | t_ch_MAX_OK | phyl_OK |
|---|---|---|---|---|---|---|---|---|---|
| **BQ.1.12** | 2239 | 96 | 7.499143 | NaN | NaN | NaN | * | * | * |
| **C.37** | 5858 | 96 | 4.944372 | -360.244348 | 920.488695 | 1.405005e-118 | | * | |
| **B.1.1.525** | 39 | 97 | 4.432309 | -407.727328 | 1015.454657 | 3.368030e-139 | | * | |
| **AY.103** | 145033 | 91 | 3.843814 | -292.321496 | 784.642991 | 4.437421e-89 | | | |
| **AZ.2** | 794 | 97 | 3.199799 | -433.501875 | 1067.003749 | 2.154942e-150 | | * | |
| **BF.11.5** | 292 | 97 | 3.093161 | -164.094019 | 528.188038 | 2.160432e-33 | | * | |
| **AY.90** | 568 | 96 | 3.034895 | -420.848098 | 1041.696197 | 6.718322e-145 | | * | |
| **AY.43** | 140119 | 97 | 2.909475 | -329.896904 | 859.793809 | 2.130666e-105 | | * | |
| **BE.1.1.1** | 1136 | 96 | 2.882548 | -121.648006 | 443.296012 | 5.863674e-15 | | * | * |
| **BN.1.7** | 411 | 97 | 2.754229 | -149.234017 | 498.468033 | 6.139833e-27 | | * | |

## AY.122 <<

| | num_seq | t_ch_MAX | max_CL | CL@BC_t_ch_MAX | aic | PV | PV_OK | t_ch_MAX_OK | phyl_OK |
|---|---|---|---|---|---|---|---|---|---|
| **AY.122** | 80528 | 98 | 4.982981 | None | None | None | * | * | * |

Cumulative Likelihood per-region

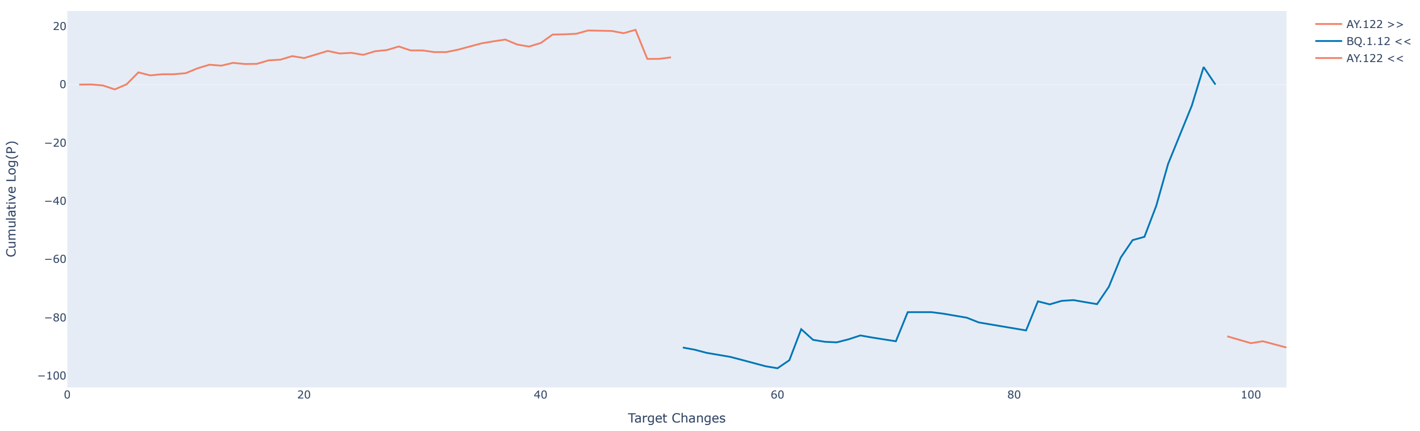


Cumulative Likelihood whole genome

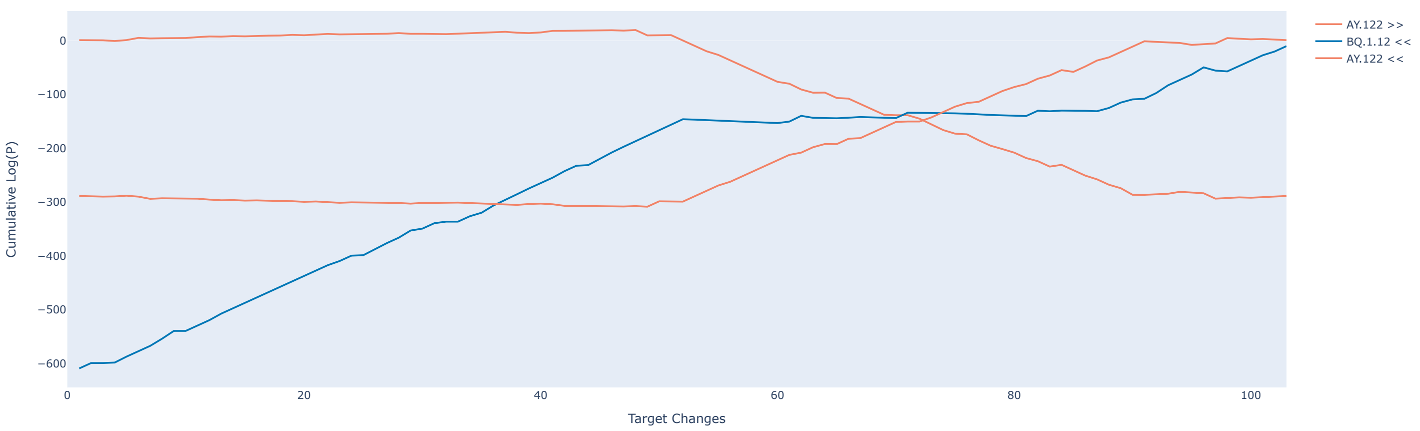


**Target sequence**

, 210_G|T, 241_C|T, 335_C|T, 569_G|A, 689_T|C, 1048_G|T, 1125_G|A, 1973_C|T, 3037_C|T, 3246_G|A, 3692_G|T, 4181_G|T, 4561_G|A, 5052_C|T, 5167_C|T, 6149_G|A, 6402_C|T, 6640_T|C, 7124_C|T, 7427_G|A, 8986_C|T, 9053_G|T, 9676_T|C, 10029_C|T, 10323_A|G, 11201_A|G, 11330_G|A, 11332_A|G, 11866_C|T, 13806_C|T, 13994_C|T, 14408_C|T, 14585_C|T, 15451_G|A, 16466_C|T, 17304_C|T, 17550_C|T, 17973_A|G, 18525_G|A, 19220_C|T, 19290_C|T, 20402_C|T, 21575_C|T, 21618_C|G, 21636_C|T, 21987_GTGTTT|......, 21995_T|G, 22029_AGTTCA|......, 22194_ATT|..., 22283_T|C, 22289_GCTTTA|......, 22578_G|A, 22674_C|T, 22679_T|C, 22686_C|T, 22688_A|G, 22775_G|A, 22786_A|C, 22813_G|T, 22882_T|G, 22899_G|A, 22942_T|A, 22992_G|A, 22995_C|A, 23013_A|C, 23042_T|C, 23055_A|G, 23063_A|T, 23075_T|C, 23114_C|T, 23403_A|G, 23525_C|T, 23604_C|A, 23854_C|A, 23948_G|T, 24145_G|A, 24424_A|T, 24469_T|A, 25000_C|T, 25584_C|T, 25592_A|G, 26060_C|T, 26270_C|T, 26537_C|T, 26577_C|G, 26709_G|A, 26858_C|T, 27259_A|C, 27382_GAT|CTC, 27807_C|T, 28248_GATTTC|......, 28273_A|., 28461_A|G, 28562_G|A, 28646_G|A, 28849_C|T, 28873_C|T, 28881_G|T, 28916_G|T, 29149_C|T, 29402_G|T, 29635_C|T, 29742_G|T

**Case 51 (2BP): XAZ** **test: KO**

Target: (75%) 1390 samples
GT: BA.2.5 + BA.5 + BA.2.5
BC: BQ.1.9 + BA.5
Direction L1: <<
Alt. candidates: [], [BA.5.5, BA.5.2.1, BE.1, BA.5.1]
Model 1BP/2BP comparison: -
Rec. model vs L1: 1.63e-48
Flags: Model_1BP_L1eqL2, Model_2BP_Bad_L1_opp

Number of changes: 69
GT BR: 8-14, 63-64
BC BR: 3-4
Initial region span: 1-3,9-69
Rec. model vs L2: 3.94e-02

GT BR coord: 3358 - 9866, 27384 - 27387
BC BR coord: 1912 - 1913
Gap history (edge excluded): 3-9
Rank L1 L2: 11 1 -

## BQ.1.9 >>

| | num_seq | t_ch_MAX | max_CL | CL@BC_t_ch_MAX | aic | PV | PV_OK | t_ch_MAX_OK | phyl_OK |
|---|---|---|---|---|---|---|---|---|---|
| **BQ.1.9** | 261 | 3 | 4.016068 | NaN | NaN | NaN | * | * | * |
| **BA.2.10.3** | 101 | 3 | 3.292852 | 3.292852 | -0.585703 | 0.486752 | * | * | |
| **BA.5.1.26** | 554 | 7 | 3.157240 | 1.008949 | 3.982101 | 0.049539 | * | | |
| **BA.2.3.14** | 529 | 5 | 3.085477 | 1.724770 | 2.550459 | 0.101266 | * | | |
| **BA.2.32** | 598 | 6 | 3.076592 | 1.782277 | 2.435446 | 0.106992 | * | | |
| **BA.2.65** | 1625 | 6 | 3.025377 | 1.637668 | 2.724663 | 0.093014 | * | | |
| **BA.2.63** | 120 | 3 | 2.892846 | 2.892846 | 0.214307 | 0.326280 | * | * | |
| **BA.2.67** | 258 | 3 | 2.822638 | 2.822638 | 0.354724 | 0.304221 | * | * | |
| **BL.6** | 138 | 3 | 2.780377 | 2.780377 | 0.439247 | 0.290835 | * | * | |
| **BA.5.1.12** | 1348 | 6 | 2.741270 | -0.910132 | 7.820265 | 0.007263 | * | | |

## BA.5 <<

| | num_seq | t_ch_MAX | max_CL | CL@BC_t_ch_MAX | aic | PV | PV_OK | t_ch_MAX_OK | phyl_OK |
|---|---|---|---|---|---|---|---|---|---|
| **BA.5** | 16505 | 9 | 50.999342 | NaN | NaN | NaN | * | * | * |
| **BA.5.3.3** | 1282 | 11 | 47.514322 | 20.304322 | 117.391355 | 4.822372e-09 | | | * |
| **BA.5.5** | 17354 | 9 | 44.210813 | 31.771002 | 94.457995 | 4.596708e-04 | * | * | * |
| **BA.5.2.1** | 93917 | 9 | 43.237413 | 40.457053 | 77.085894 | 2.718282e+00 | * | * | * |
| **BA.5.3** | 1582 | 9 | 42.804823 | 21.261839 | 115.476321 | 1.253176e-08 | | * | * |
| **BE.1** | 14022 | 9 | 42.490736 | 28.329889 | 101.340221 | 1.473920e-05 | * | * | * |
| **BA.5.1** | 112751 | 9 | 42.465047 | 38.056481 | 81.887037 | 2.465970e-01 | * | * | * |
| **BF.17** | 357 | 11 | 42.264684 | 17.202073 | 123.595854 | 2.161605e-10 | | | * |
| **BA.5.3.2** | 912 | 9 | 41.888687 | 9.715789 | 138.568423 | 1.213618e-13 | | * | * |
| **BF.28** | 4033 | 11 | 41.675675 | 21.192800 | 115.614399 | 1.174310e-08 | | | * |

Cumulative Likelihood per-region

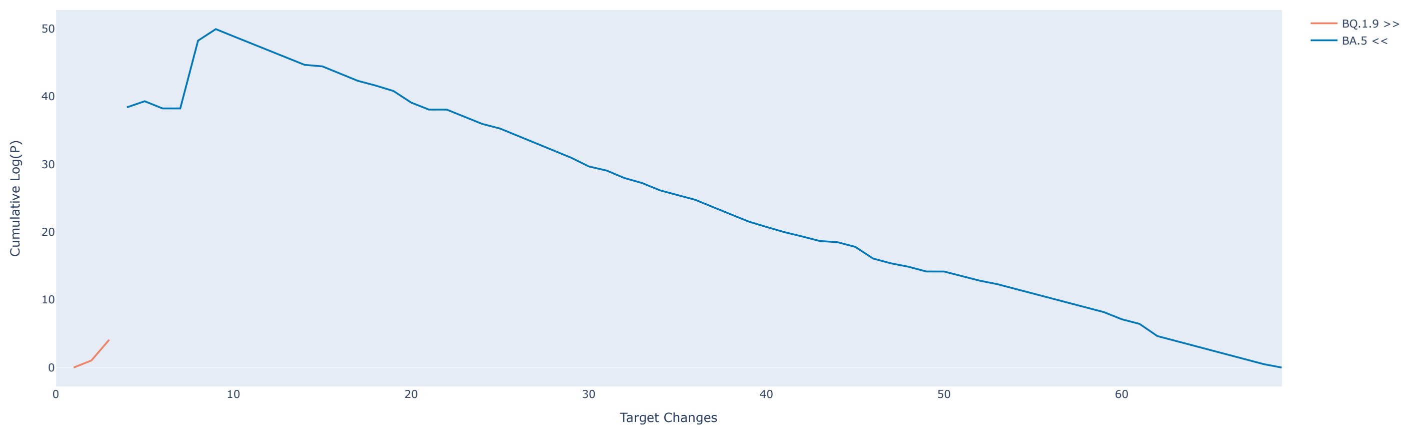


Cumulative Likelihood whole genome

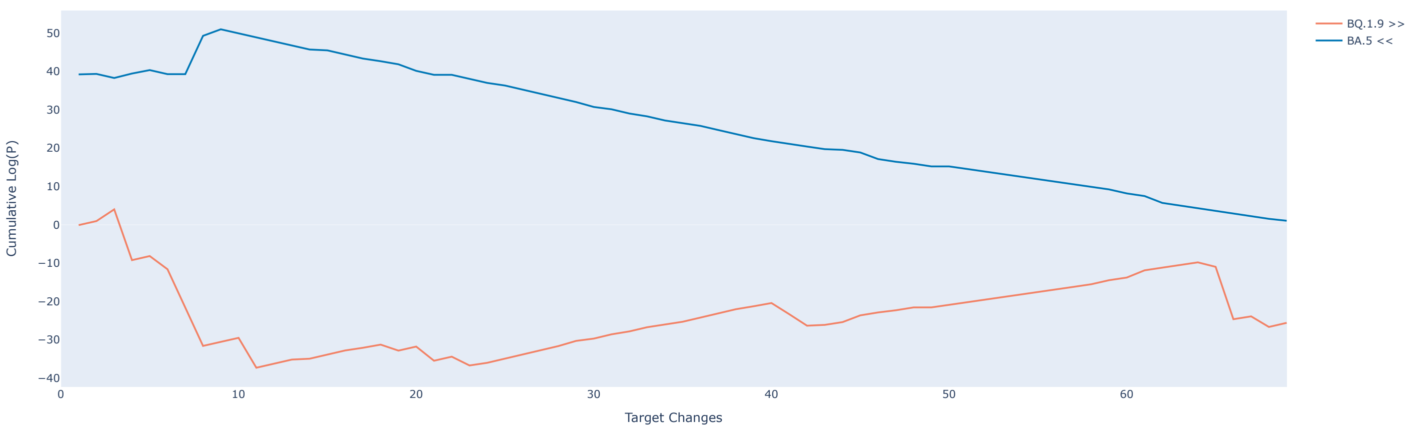


**Target sequence**

, 241_C|T, 670_T|G, 1912_C|T, 2232_C|T, 2790_C|T, 3037_C|T, 3317_C|T, 3358_T|C, 4184_G|A, 4321_C|T, 9344_C|T, 9424_A|G, 9534_C|T, 10029_C|T, 10198_C|T, 10447_G|A, 10449_C|A, 11288_TCTGGTTTT|........., 12160_G|A, 12880_C|T, 14408_C|T, 15714_C|T, 17410_C|T, 18163_A|G, 19955_C|T, 20055_A|G, 21618_C|T, 21633_TACCCCCTG|........., 21765_TACATG|......, 21987_G|A, 22200_T|G, 22578_G|A, 22674_C|T, 22679_T|C, 22686_C|T, 22688_A|G, 22775_G|A, 22786_A|C, 22813_G|T, 22882_T|G, 22917_T|G, 22992_G|A, 22995_C|A, 23013_A|C, 23018_T|G, 23055_A|G, 23063_A|T, 23075_T|C, 23403_A|G, 23525_C|T, 23599_T|G, 23604_C|A, 23854_C|A, 23948_G|T, 24424_A|T, 24469_T|A, 25000_C|T, 25584_C|T, 26060_C|T, 26270_C|T, 26529_G|A, 26577_C|G, 26709_G|A, 27807_C|T, 28271_A|T, 28311_C|T, 28362_GAGAACGCA|........., 28881_GGG|AAC, 29510_A|C

**Case 52 (2BP): XBL** **test: OK**

Target: (75%) 153 samples | Number of changes: 92
GT: XBB.1 + BA.2.75 + XBB.1 | GT BR: 2–7, 12–22 | GT BR coord: 405 – 3796, 5183 – 12444 | Rank L1 L2: 11 11 3
BC: XBB.1.5 + BN.1.3 + XBB.1.5 | BC BR: 5–6, 12–13 | BC BR coord: 3039 – 3040, 5183 – 5184
Direction L1: << | Initial region span: 1–5,6–12,13–92 | Gap history (edge excluded): 5–13 ->
Alt. candidates: [BA.2.75.5], []
Model 1BP/2BP comparison: 2BP vs 1BP: 5.67e–06
Rec. model vs L1: 8.60e–16 | Rec. model vs L2: 1.76e–130
Flags: Model_2BP_Best

## XBB.1.5 >>

| | num_seq | t_ch_MAX | max_CL | CL@BC_t_ch_MAX | aic | PV | PV_OK | t_ch_MAX_OK | phyl_OK |
|---|---|---|---|---|---|---|---|---|---|
| **XBB.1.5** | 52808 | 5 | 6.196606 | None | None | None | * | * | * |

## BN.1.3 >>

| | num_seq | t_ch_MAX | max_CL | CL@BC_t_ch_MAX | aic | PV | PV_OK | t_ch_MAX_OK | phyl_OK |
|---|---|---|---|---|---|---|---|---|---|
| **BN.1.3** | 10795 | 12 | 22.663661 | NaN | NaN | NaN | * | * | * |
| **BA.2.75.2** | 1428 | 12 | 22.189107 | 22.189107 | -24.378213 | 0.621885 | * | * | |
| **BA.2.75.5** | 645 | 12 | 21.945301 | 21.945301 | -23.890601 | 0.486752 | * | * | * |
| **CH.1.1.3** | 3169 | 12 | 16.528199 | 16.528199 | -13.056399 | 0.002166 | * | * | |
| **BR.1.2** | 75 | 12 | 14.440790 | 14.440790 | -8.881580 | 0.000268 | * | * | |
| **BM.1.1.3** | 1433 | 12 | 13.630498 | 13.630498 | -7.260995 | 0.000119 | * | * | |
| **BL.1.2** | 12 | 12 | 9.789820 | 9.789820 | 0.420361 | 0.000003 | | * | |
| **BR.1.1** | 25 | 12 | 9.789820 | 9.789820 | 0.420361 | 0.000003 | | * | |
| **BL.1.3** | 18 | 12 | 9.789820 | 9.789820 | 0.420361 | 0.000003 | | * | |
| **BY.1.2.1** | 11 | 12 | 9.789820 | 9.789820 | 0.420361 | 0.000003 | | * | |

## XBB.1.5 <<

| | num_seq | t_ch_MAX | max_CL | CL@BC_t_ch_MAX | aic | PV | PV_OK | t_ch_MAX_OK | phyl_OK |
|---|---|---|---|---|---|---|---|---|---|
| **XBB.1.5** | 52808 | 13 | 123.330708 | NaN | NaN | NaN | * | * | * |
| **XBB.1.9.1** | 3817 | 22 | 110.766927 | 108.013057 | -12.026114 | 4.553294e-10 | | | |
| **XBB.1** | 12130 | 13 | 107.654484 | 113.845684 | -23.691368 | 1.549753e-07 | | * | * |
| **XBB.1.9** | 1693 | 13 | 103.203085 | 102.579468 | -1.158935 | 1.985799e-12 | | * | |
| **XBB.1.1** | 740 | 13 | 101.810003 | 108.040958 | -12.081916 | 4.668561e-10 | | * | |
| **XBB.1.2** | 2772 | 13 | 100.671522 | 106.876935 | -9.753870 | 1.456230e-10 | | * | |
| **XBB.1.4** | 204 | 13 | 95.642727 | 101.895661 | 0.208679 | 1.001022e-12 | | * | |
| **XBB.1.7** | 100 | 13 | 91.025479 | 97.355367 | 9.289265 | 1.068431e-14 | | * | |
| **XBB.4** | 279 | 13 | 86.418935 | 92.708028 | 18.583944 | 1.026712e-16 | | * | |
| **XBB.1.6** | 69 | 13 | 86.080631 | 81.296844 | 41.406311 | 1.132341e-21 | | * | |

Cumulative Likelihood per-region

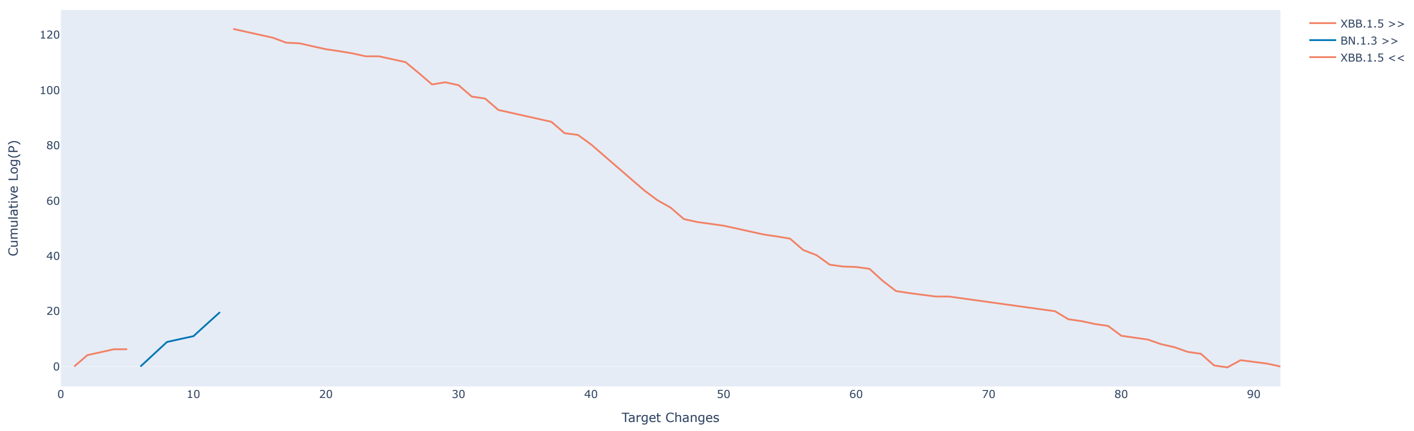


Cumulative Likelihood whole genome

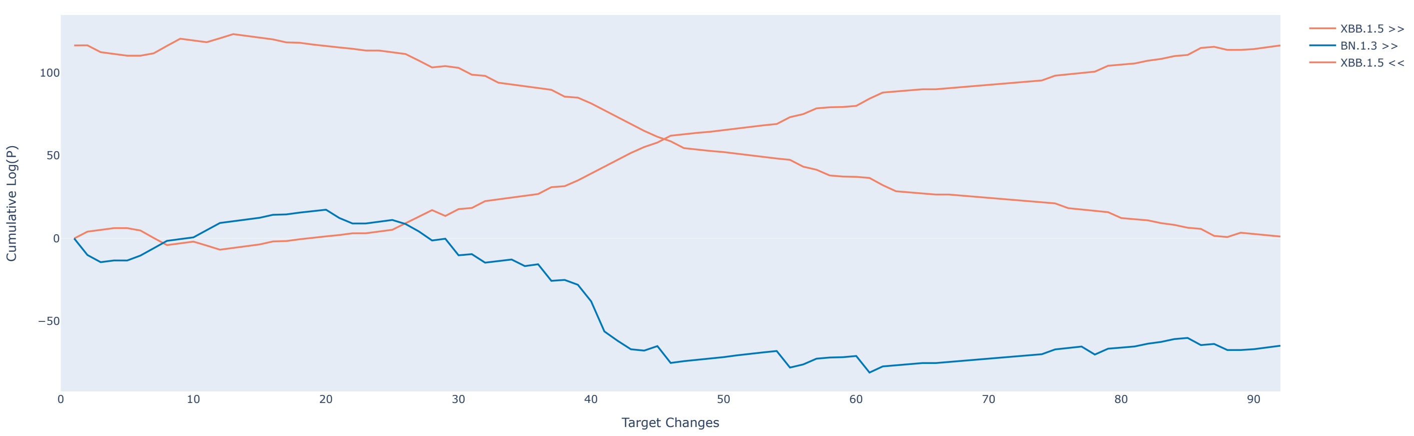


**Target sequence**

, 241_C|T, 405_A|G, 670_T|G, 2790_C|T, 3037_C|T, 3446_G|A, 3796_C|T, 3927_C|T, 4184_G|A, 4321_C|T, 4586_C|T, 5183_C|T, 9344_C|T, 9424_A|G, 9534_C|T, 9866_C|T, 10029_C|T, 10198_C|T, 10447_G|A, 10449_C|A, 11288_TCTGGTTTT|........., 12880_C|T, 14408_C|T, 15451_G|A, 15714_C|T, 15738_C|T, 15939_T|C, 16342_T|C, 17410_C|T, 17859_T|C, 18163_A|G, 19326_A|G, 19955_C|T, 20055_A|G, 21618_C|T, 21633_TACCCCCTG|........., 21810_T|C, 21987_G|A, 21991_TTA|..., 22000_C|A, 22109_C|G, 22200_T|A, 22317_G|T, 22577_GG|CA, 22599_G|C, 22664_C|A, 22674_C|T, 22679_T|C, 22686_C|T, 22688_A|G,

22775_G|A, 22786_A|C, 22813_G|T, 22882_T|G, 22895_GT|CC, 22898_G|A, 22942_T|G, 22992_G|A, 22995_C|A, 23013_A|C, 23018_TT|CC, 23031_T|C, 23055_A|G, 23063_A|T, 23075_T|C, 23403_A|G, 23525_C|T, 23599_T|G, 23604_C|A, 23854_C|A, 23948_G|T, 24424_A|T, 24469_T|A, 25000_C|T, 25416_C|T, 25584_C|T, 26060_C|T, 26270_C|T, 26275_A|G, 26577_C|G, 26709_G|A, 26858_C|T, 27259_A|C, 27382_GAT|CTC, 27807_C|T, 27915_G|T, 28271_A|T, 28311_CC|TT, 28362_GAGAACGCA|........., 28881_GGG|AAC, 29510_A|C, 29734_GAGGCCACGCGGAGTACGATCGAGTG|..........................

## Case 53 (2BP): XD test: OK

Target: (75%) 14 samples
Number of changes: 67
GT: B.1.617.2* + BA.1* + B.1.617.2*
GT BR: 23-26, 51-53
GT BR coord: 22075 - 22236, 24999 - 25481
Rank_L1_L2: 2 11 11
BC: AY.4 + BA.1.22 + AY.4
BC BR: 23-24, 52-53
BC BR coord: 22031 - 22032, 25468 - 25469
Direction L1: >>
Initial region span: 1-23,24-52,53-67
Gap history (edge excluded): 23-53 ->
Alt. candidates: [], []
Model 1BP/2BP comparison: 2BP vs 1BP: 5.74e-121
Rec. model vs L1: 2.21e-170
Rec. model vs L2: 0.00e+00
Flags: Model_1BP_L1eqL2, Model_2BP_Best

### AY.4 >>

| | num_seq | t_ch_MAX | max_CL | CL@BC_t_ch_MAX | aic | PV | PV_OK | t_ch_MAX_OK | phyl_OK |
|---|---|---|---|---|---|---|---|---|---|
| **AY.4** | 245319 | 23 | 25.550447 | NaN | NaN | NaN | * | * | * |
| **B.1.617.2** | 53135 | 23 | 7.827505 | 18.583689 | 54.832623 | 2.030467e-09 | | * | * |
| **AY.122** | 80528 | 20 | 5.172517 | 4.759244 | 82.481511 | 2.011290e-15 | | | |
| **AY.126** | 28427 | 6 | 3.623978 | -8.137706 | 108.275412 | 5.024304e-21 | | | |
| **AY.39** | 18915 | 6 | 3.339922 | -1.710123 | 95.420246 | 3.115942e-18 | | | |
| **AY.43** | 140119 | 6 | 1.301792 | -2.278201 | 96.556402 | 1.762145e-18 | | | * |
| **AY.103** | 145033 | 6 | 1.300674 | -12.701899 | 117.403797 | 5.256457e-23 | | | |

### BA.1.22 >>

| | num_seq | t_ch_MAX | max_CL | CL@BC_t_ch_MAX | aic | PV | PV_OK | t_ch_MAX_OK | phyl_OK |
|---|---|---|---|---|---|---|---|---|---|
| **BA.1.22** | 57 | 52 | 29.202296 | NaN | NaN | NaN | * | * | * |
| **BA.1.3** | 17 | 52 | 29.085646 | 29.085646 | 1.828708 | 0.891366 | * | * | |
| **BA.1.17.1** | 201 | 52 | 28.862672 | 28.862672 | 2.274656 | 0.715338 | * | * | |
| **BA.1.13.1** | 1484 | 52 | 28.628065 | 28.628065 | 2.743870 | 0.565525 | * | * | |
| **BA.1.6** | 208 | 52 | 28.539225 | 28.539225 | 2.921551 | 0.516851 | * | * | |
| **BA.1.10** | 840 | 52 | 28.113315 | 28.113315 | 3.773370 | 0.337902 | * | * | |
| **BA.1.20** | 11897 | 52 | 28.078238 | 28.078238 | 3.843524 | 0.326280 | * | * | |
| **BA.1.16.1** | 90 | 52 | 28.026107 | 18.924693 | 22.150614 | 0.000034 | * | * | |
| **BA.1.16** | 3049 | 52 | 27.906353 | 27.906353 | 4.187293 | 0.273898 | * | * | |
| **BA.1.21** | 2247 | 52 | 27.901395 | 27.901395 | 4.197209 | 0.272532 | * | * | |

### AY.4 <<

| | num_seq | t_ch_MAX | max_CL | CL@BC_t_ch_MAX | aic | PV | PV_OK | t_ch_MAX_OK | phyl_OK |
|---|---|---|---|---|---|---|---|---|---|
| **AY.4** | 245319 | 53 | 13.051347 | None | None | None | * | * | * |

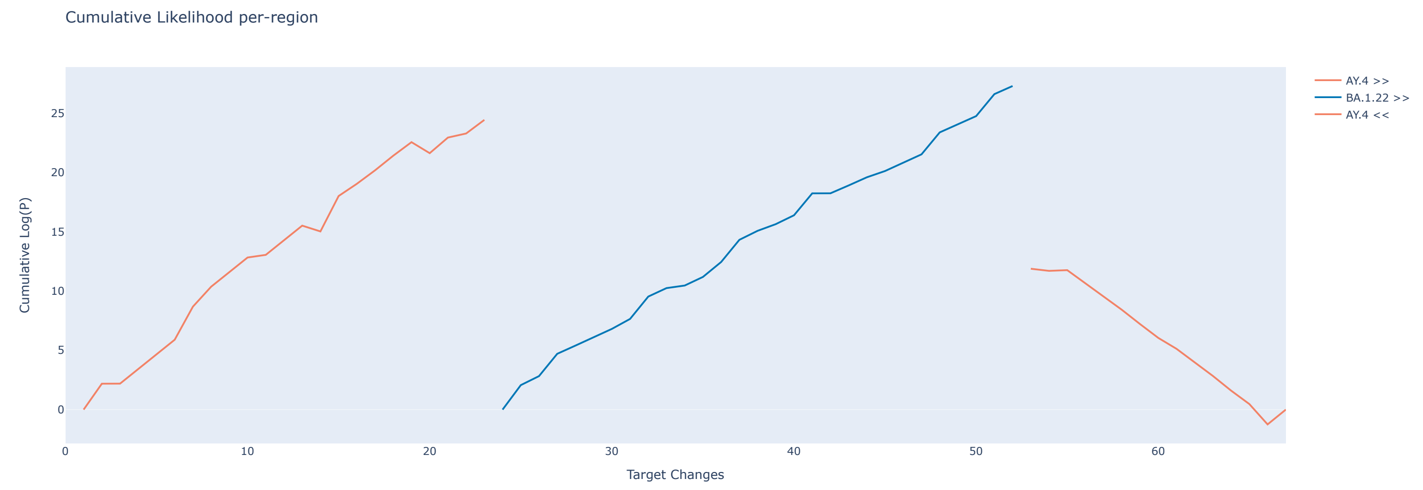


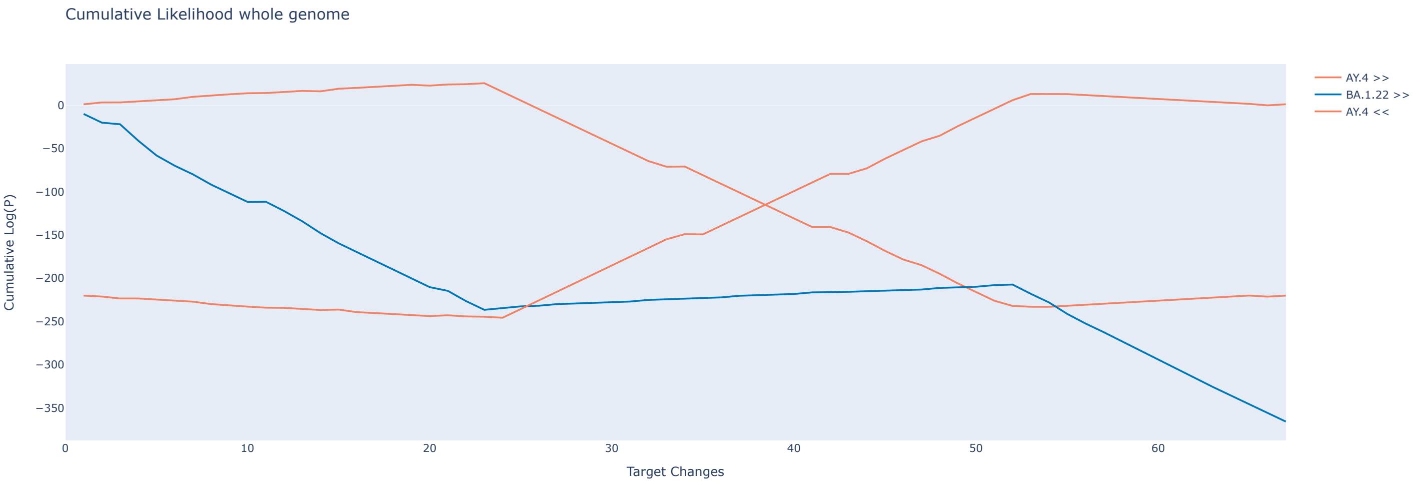


**Target sequence**

, 210_G|T, 1321_A|C, 3037_C|T, 4181_G|T, 6402_C|T, 7124_C|T, 7851_C|T, 8723_A|G, 8986_C|T, 9053_G|T, 10029_C|T, 11201_A|G, 11332_A|G, 14407_CC|TT, 15264_T|C, 15451_G|A, 16466_C|T, 19220_C|T, 21618_C|G, 21641_G|T, 21846_C|T, 21987_G|A, 22029_AGTTCA|......, 22194_ATT|..., 22205_.........|AGCCAGAAG, 22578_G|A, 22673_TC|CT, 22679_T|C, 22686_C|T, 22813_G|T, 22882_T|G, 22898_G|A, 22992_G|A, 22995_C|A, 23013_A|C, 23040_A|G, 23048_G|A, 23055_A|G, 23063_A|T, 23075_T|C, 23202_C|A, 23403_A|G, 23525_C|T, 23599_T|G, 23604_C|A, 23854_C|A, 23948_G|T, 24130_C|A, 24424_A|T, 24469_T|A, 24503_C|T, 25000_C|T, 25667_C|T, 25855_G|T, 26767_T|C, 27638_T|C, 27752_C|T, 27874_C|T, 28248_GATTTC|......, 28273_A|., 28461_A|G, 28881_G|T, 28916_G|T, 29402_G|T, 29540_G|A, 29645_G|T, 29742_G|T

## Case 54 (undefined): XAJ test: KO

Target: (75%) 38 samples
GT: BA.2.12.1* + BA.4*
BC: BA.2.12
Direction L1: <<
Alt. candidates: [BA.2.12.1]
Model 1BP/2BP comparison: –
Rec. model vs L1: –
Flags: NotEnoughSpaceAfterL1, SingleCandidateGenome

Number of changes: 72
GT BR: –
GT BR coord: –
Rank L1 L2: 8 –
BC BR:
BC BR coord:
Initial region span: 2–72
Gap history (edge excluded):
Rec. model vs L2: –

### BA.2.12 <<

| | num_seq | t_ch_MAX | max_CL | CL@BC_t_ch_MAX | aic | PV | PV_OK | t_ch_MAX_OK | phyl_OK |
|---|---|---|---|---|---|---|---|---|---|
| **BA.2.12** | 2844 | 2 | 33.809519 | NaN | NaN | NaN | * | * | * |
| **BA.4** | 16319 | 20 | 29.258065 | 21.506413 | 142.987175 | 4.955562e-06 | | | |
| **BA.4.4** | 2910 | 20 | 28.348997 | 18.011320 | 149.977361 | 1.503951e-07 | | | |
| **BA.4.8** | 75 | 2 | 27.840903 | 27.802766 | 130.394467 | 2.698660e-03 | * | * | |
| **BA.4.6** | 20299 | 20 | 27.624500 | 5.492162 | 175.015676 | 5.493723e-13 | | | |
| **BG.1** | 247 | 49 | 25.675909 | -7.530675 | 201.061349 | 1.217174e-18 | | | * |
| **BG.3** | 152 | 49 | 25.644309 | -6.753840 | 199.507681 | 2.641988e-18 | | | * |
| **BA.2.12.1** | 84020 | 2 | 25.551601 | 25.467831 | 135.064338 | 2.612586e-04 | * | * | * |
| **BA.4.6.5** | 1581 | 20 | 22.468256 | -2.444253 | 190.888506 | 1.966709e-16 | | | |
| **BA.4.6.4** | 870 | 20 | 21.120350 | -11.717934 | 209.435868 | 1.843566e-20 | | | |

Cumulative Likelihood per-region

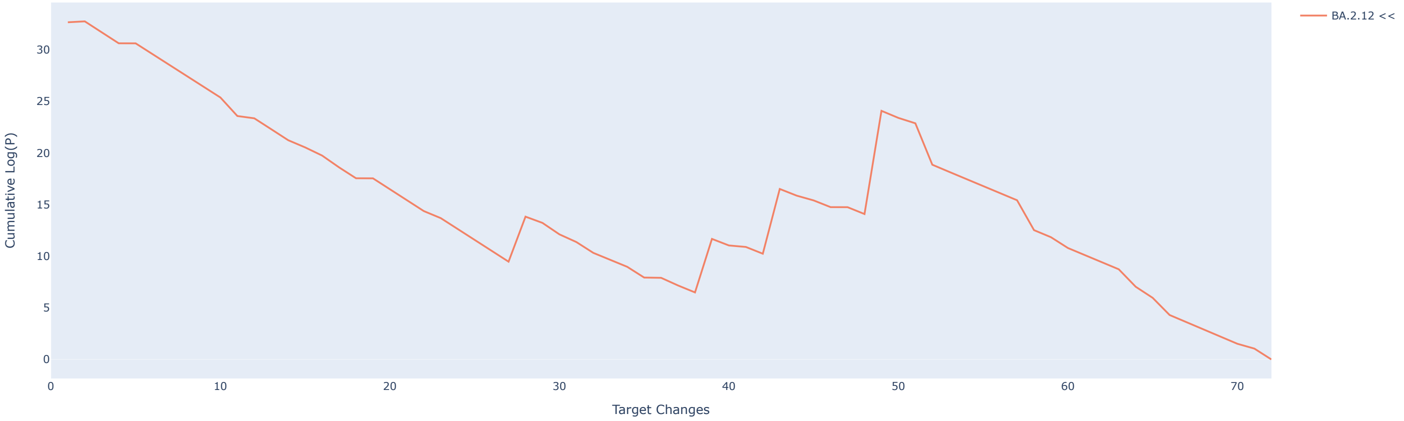


Cumulative Likelihood whole genome

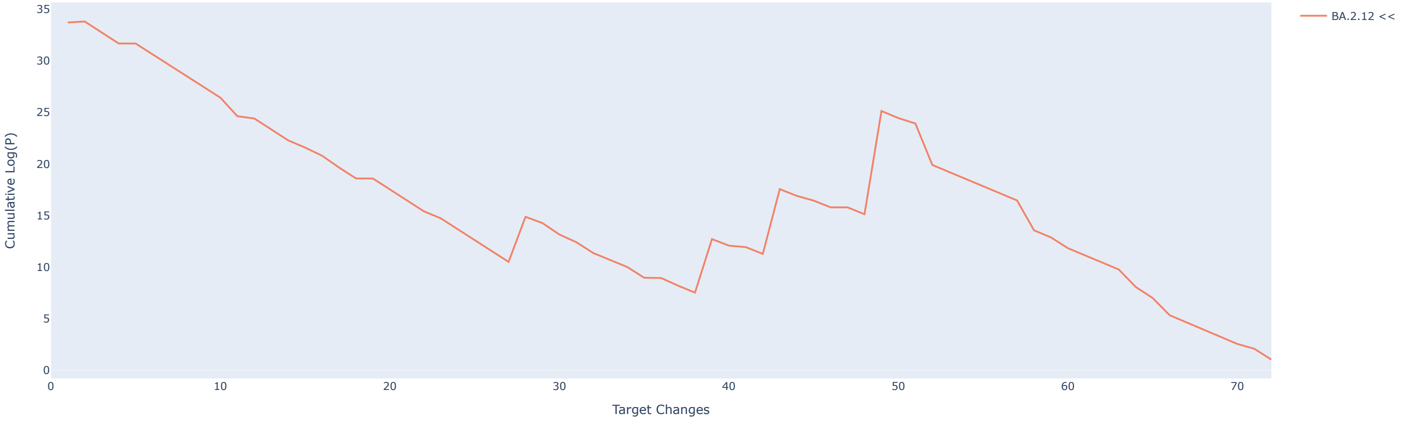


**Target sequence**

, 241_C|T, 670_T|G, 2790_C|T, 3037_C|T, 4184_G|A, 4321_C|T, 9344_C|T, 9424_A|G, 9534_C|T, 9866_C|T, 10029_C|T, 10198_C|T, 10447_G|A, 10449_C|A, 11288_TCTGGTTTT|........., 11674_C|T, 12880_C|T, 14408_C|T, 15009_T|C, 15714_C|T, 17410_C|T, 18163_A|G, 19955_C|T, 20055_A|G, 21618_C|T, 21633_TACCCCCTG|........., 21765_TACATG|......, 21987_G|A, 22200_T|G, 22578_G|A, 22674_C|T, 22679_T|C, 22686_C|T, 22688_A|G, 22775_G|A, 22813_G|T, 22882_T|G, 22917_T|G, 22992_G|A, 22995_C|A, 23013_A|C, 23018_T|G, 23055_A|G, 23063_A|T, 23075_T|C, 23403_A|G, 23525_C|T, 23535_A|G, 23599_T|G, 23604_C|A, 23673_C|T, 23854_C|A, 23948_G|T, 24424_A|T, 24469_T|A, 25000_C|T, 25416_C|T, 25584_C|T, 26060_C|T, 26270_C|T, 26577_C|G, 26709_G|A, 26858_C|T, 27259_A|C, 27382_GAT|CTC, 27807_C|T, 28271_A|T, 28311_C|T, 28362_GAGAACGCA|........., 28881_GGG|AAC, 29510_A|C, 29734_GAGGCCACGCGGAGTACGATCGAGTG|..........................

## Case 55 (undefined): XAY test: KO

Target: (75%) 21 samples
GT: BA.2* + AY.45 + BA.2* + AY.45 + BA.2*
BC: BA.2.38.1
Direction L1: <<
Alt. candidates: []
Model 1BP/2BP comparison: –
Rec. model vs L1: –
Flags: Model_2BP_Bad_L2, SingleCandidateGenome

Number of changes: 75
GT BR: –
GT BR coord: –
Rank L1 L2: 11 – – – –
BC BR:
BC BR coord:
Initial region span: 68–75
Gap history (edge excluded):
Rec. model vs L2: –

# BA.2.38.1 <<

| | num_seq | t_ch_MAX | max_CL | CL@BC_t_ch_MAX | aic | PV | PV_OK | t_ch_MAX_OK | phyl_OK |
|---|---|---|---|---|---|---|---|---|---|
| **BA.2.38.1** | 579 | 68 | 10.315466 | NaN | NaN | NaN | * | * | * |
| **CH.1.1.4** | 198 | 68 | 7.842123 | -359.083061 | 1028.166122 | 7.890413e-37 | | * | |
| **BA.2.4** | 261 | 68 | 7.256485 | -298.562695 | 907.125391 | 1.515660e-10 | | * | |
| **BR.1.2** | 75 | 68 | 6.813785 | -402.405634 | 1114.811269 | 1.211889e-55 | | * | |
| **BN.1.8** | 260 | 68 | 6.783333 | -354.255400 | 1018.510799 | 9.879662e-35 | | * | |
| **BA.2.32** | 598 | 68 | 6.652532 | -281.508816 | 873.017633 | 3.868068e-03 | * | * | |
| **BN.5** | 231 | 68 | 6.567069 | -357.602286 | 1025.204571 | 3.483590e-36 | | * | |
| **BA.5.3** | 1582 | 69 | 6.482184 | -237.769335 | 785.538670 | 3.832964e+16 | * | * | |
| **BA.2.75.2** | 1428 | 68 | 6.243422 | -292.705059 | 895.410119 | 5.315785e-08 | | * | |
| **BA.2.3.5** | 125 | 68 | 6.066999 | -303.681390 | 917.362780 | 9.103020e-13 | | * | |

Cumulative Likelihood per-region

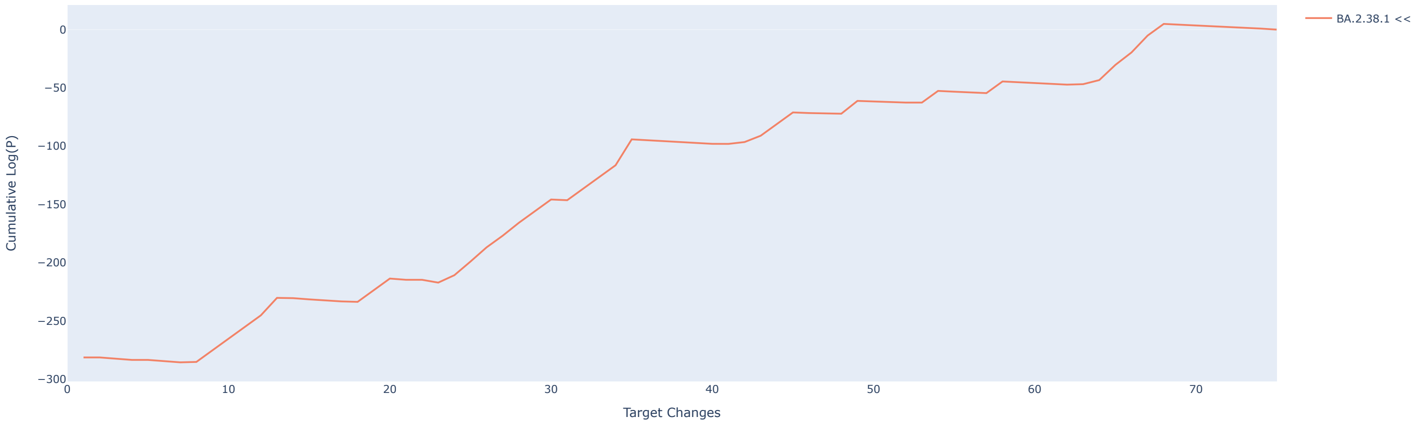


Cumulative Likelihood whole genome

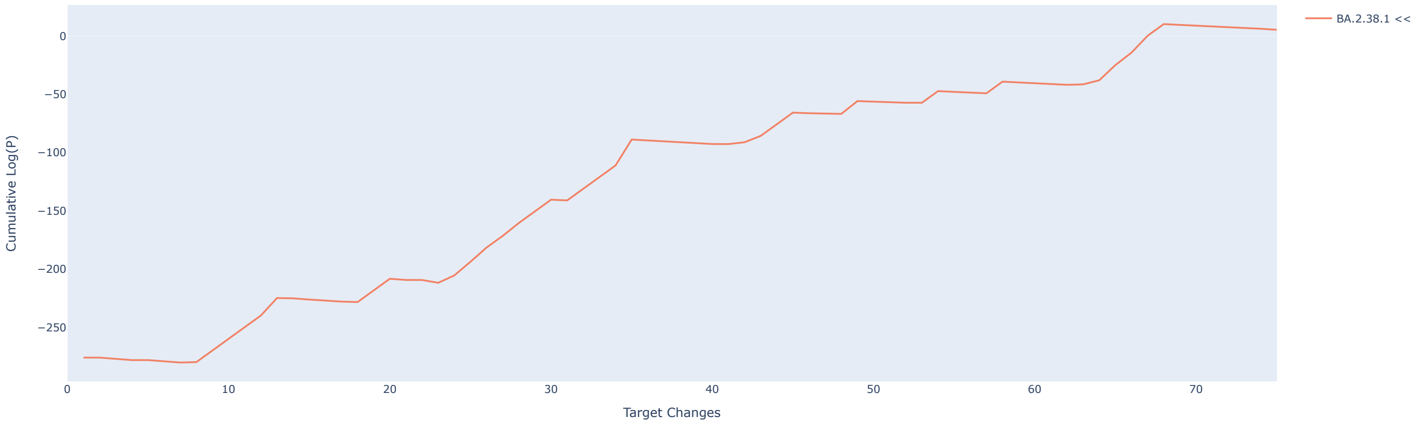


**Target sequence**

, 241_C|T, 670_T|G, 2790_C|T, 3037_C|T, 4184_G|A, 4321_C|T, 4456_C|T, 5869_C|T, 7124_C|T, 8595_C|T, 8986_C|T, 9053_G|T, 10029_C|T, 10198_C|T, 10447_G|A, 10449_C|A, 11288_TCTGGTTTT|........., 12163_G|A, 12747_C|T, 12880_C|T, 14408_C|T, 15026_C|T, 15451_G|A, 16466_C|T, 19220_C|T, 21614_C|T, 21618_C|G, 21623_A|G, 21846_C|T, 21987_G|A, 22017_G|T, 22029_AGTTCA|......, 22118_T|C, 22200_T|G, 22578_G|A, 22674_C|T, 22679_T|C, 22686_C|T, 22688_A|G, 22775_G|A, 22813_G|T, 22882_T|G, 22899_G|A, 22916_CT|AG, 22992_G|A, 22995_C|A, 23013_A|C, 23018_TT|CC, 23055_A|G, 23063_A|T, 23075_T|C, 23403_A|G, 23423_C|T, 23525_C|T, 23599_T|G, 23604_C|A, 23679_C|T, 23854_C|A, 23948_G|T, 24424_A|T, 24469_T|A, 24912_C|T, 25413_C|T, 25469_C|T, 26681_C|T, 26767_T|C, 27575_C|G, 27807_C|T, 28271_A|T, 28311_C|T, 28362_GAGAACGCA|........., 28881_GGG|AAC, 29510_A|C, 29734_GAGGCCACGCGGAGTACGATCGAGTG|.........................., 29779_G|T

## Case 56 (undefined): XBC          test: KO

Target: (75%) 4 samples
GT: BA.2* + B.1.617.2* + BA.2* + B.1.617.2*
BC: BA.2.2.1 + AY.26.1
Direction L1: <<
Alt. candidates: [BA.2.2], []
Model 1BP/2BP comparison: –
Rec. model vs L1: 6.79e−122
Flags: Model_2BP_Bad_L1_opp

Number of changes: 73
GT BR: 2–5, 34–36, 60–62
BC BR: 63–64
Initial region span: 1–5,68–73
Rec. model vs L2: 2.09e+27

GT BR coord: 2789 – 4185, 22577 – 22675, 24999 – 25585
BC BR coord: 27705 – 27706
Gap history (edge excluded): 5–68

Rank L1 L2: 11 11 – –

## BA.2.2.1 >>

| | num_seq | t_ch_MAX | max_CL | CL@BC_t_ch_MAX | aic | PV | PV_OK | t_ch_MAX_OK | phyl_OK |
|---|---|---|---|---|---|---|---|---|---|
| **BA.2.2.1** | 394 | 5 | 5.730617 | NaN | NaN | NaN | * | * | * |
| **BA.2.2** | 1696 | 5 | 5.608882 | -280.062839 | 850.125678 | 2.656759e+34 | * | * | * |
| **BA.2.38.2** | 359 | 5 | 5.196574 | -355.989775 | 1001.979550 | 2.821913e+01 | * | * | |
| **BA.2.75.9** | 12 | 4 | 2.199765 | -382.255469 | 1054.510938 | 1.106112e-10 | | * | |
| **BM.6** | 10 | 4 | 2.199765 | -402.223106 | 1094.446211 | 2.349298e-19 | | * | |
| **BA.2.40** | 17 | 4 | 2.199765 | -345.600476 | 981.200953 | 9.180435e+05 | * | * | |
| **BA.2.76.2** | 17 | 4 | 2.199765 | -383.500678 | 1057.001356 | 3.184948e-11 | | * | |
| **BR.1.1** | 25 | 4 | 2.199765 | -397.605883 | 1085.211765 | 2.384398e-17 | | * | |
| **BA.2.33** | 389 | 4 | 2.192023 | -312.718899 | 915.437797 | 1.747696e+20 | * | * | |
| **BG.3** | 152 | 4 | 2.186520 | -390.267265 | 1070.534531 | 3.673671e-14 | | * | |

## AY.26.1 <<

| | num_seq | t_ch_MAX | max_CL | CL@BC_t_ch_MAX | aic | PV | PV_OK | t_ch_MAX_OK | phyl_OK |
|---|---|---|---|---|---|---|---|---|---|
| **AY.26.1** | 134 | 68 | 10.832268 | NaN | NaN | NaN | * | * | * |
| **AY.28** | 280 | 68 | 7.300342 | -21.554180 | 81.108360 | 2.989691e-02 | * | * | |
| **AY.50** | 1016 | 70 | 6.980219 | -32.926906 | 103.853812 | 3.449039e-07 | | | |
| **AY.25.1.2** | 57 | 68 | 6.281430 | -23.813228 | 85.626457 | 3.119758e-03 | * | * | |
| **AY.55** | 178 | 68 | 6.130783 | -22.722204 | 83.444408 | 9.325525e-03 | * | * | |
| **AY.43.1** | 697 | 69 | 5.907891 | -27.564889 | 93.129778 | 7.336966e-05 | * | * | |
| **AY.48** | 758 | 69 | 5.866995 | -41.760531 | 121.521062 | 5.020031e-11 | | * | |
| **AY.19** | 171 | 68 | 5.771971 | -28.082467 | 94.164934 | 4.383842e-05 | * | * | |
| **AY.4.14** | 451 | 68 | 5.589538 | -32.448663 | 102.897326 | 5.546104e-07 | | * | |
| **AY.102** | 5717 | 68 | 5.427889 | -24.666244 | 87.332488 | 1.333431e-03 | * | * | |

Cumulative Likelihood per-region

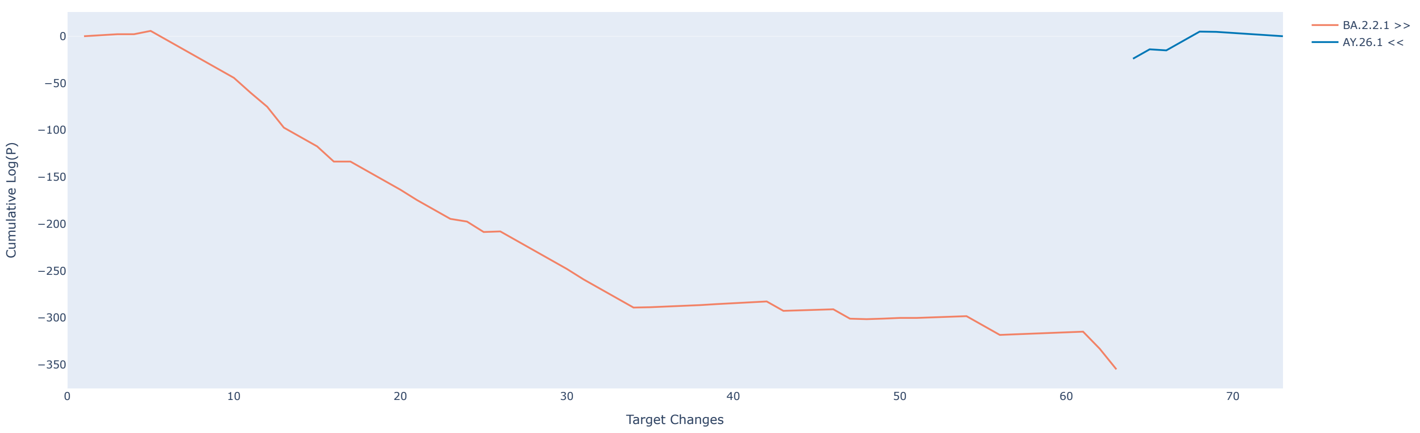


Cumulative Likelihood whole genome

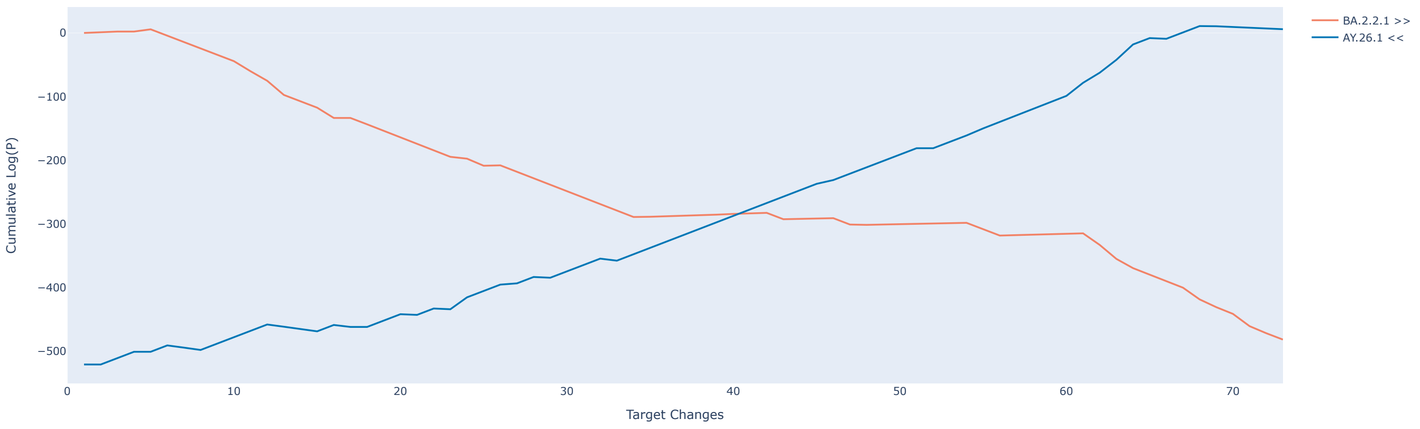


**Target sequence**

, 241_C|T, 670_T|G, 2790_C|T, 3037_C|T, 4893_C|T, 5184_C|T, 5584_A|G, 6196_C|T, 6576_G|T, 7091_G|A, 9073_C|T, 9891_C|T, 11418_T|C, 11514_C|T, 11956_C|T, 13019_C|T, 14408_C|T, 15030_T|C, 15237_C|T, 15451_G|A, 15914_...|TGA, 16466_C|T, 16616_C|T, 21618_C|T, 21635_C|T, 21987_G|A, 21991_TTA|..., 22029_AGTTCA|......, 22188_C|T, 22197_T|C, 22205_G|C, 22227_C|T, 22289_GCTTTA|......, 22329_C|T, 22674_C|T, 22679_T|C, 22686_C|T, 22688_A|G, 22775_G|A, 22786_A|C, 22813_G|T, 22882_T|G, 22898_G|A, 22992_G|A, 22995_C|A, 23013_A|C, 23018_TT|CC, 23055_A|G, 23063_A|T, 23075_T|C, 23403_A|G, 23525_C|T, 23599_T|G, 23604_C|A, 23614_A|G, 23670_A|T, 23854_C|A, 23948_G|T, 24424_A|T, 24469_T|A, 25000_C|T, 25854_C|T, 26819_T|C, 27718_T|C, 27752_C|T, 27890_G|T, 27898_A|C, 28248_GATTTC|......, 28461_A|G, 28881_G|T, 29402_G|T, 29742_G|T, 29769_C|T

## Case 57 (undefined): XBK    test: OK

Target: (75%) 667 samples  Number of changes: 85
GT: BA.5.2 + CJ.1  GT BR: –  GT BR coord: –  Rank L1 L2: 11 1
BC: BF.5.2 + CJ.1  BC BR: 3–4  BC BR coord: 1629 – 1630
Direction L1: <<  Initial region span: 1–3,4–85  Gap history (edge excluded):
Alt. candidates: [], []
Model 1BP/2BP comparison: –
Rec. model vs L1: 0.00e+00  Rec. model vs L2: 4.38e–03
Flags: Model_2BP_Bad_L1_opp

# BF.5.2 >>

| | num_seq | t_ch_MAX | max_CL | CL@BC_t_ch_MAX | aic | PV | PV_OK | t_ch_MAX_OK | phyl_OK |
|---|---|---|---|---|---|---|---|---|---|
| **BF.5.2** | 61 | 3 | 3.656337 | NaN | NaN | NaN | * | * | * |
| **BF.22** | 295 | 3 | 3.655819 | 3.655819 | -1.311638 | 1.000000 | * | * | |
| **BA.5.2.12** | 1681 | 3 | 3.653729 | 3.653729 | -1.307459 | 1.000000 | * | * | |
| **BF.7.15** | 689 | 3 | 3.647974 | 3.647974 | -1.295949 | 0.995012 | * | * | |
| **BF.5.1** | 270 | 3 | 3.646683 | 3.646683 | -1.293366 | 0.990050 | * | * | |
| **BF.9** | 380 | 3 | 3.624340 | 3.624340 | -1.248679 | 0.970446 | * | * | |
| **CK.1.1** | 76 | 3 | 3.618799 | 3.618799 | -1.237599 | 0.965605 | * | * | |
| **BA.5.2.43** | 575 | 3 | 3.611016 | 3.611016 | -1.222032 | 0.955997 | * | * | |
| **BF.32** | 302 | 3 | 3.608588 | 3.608588 | -1.217177 | 0.955997 | * | * | |
| **CP.2** | 30 | 3 | 3.605064 | 3.605064 | -1.210127 | 0.951229 | * | * | |

# CJ.1 <<

| | num_seq | t_ch_MAX | max_CL | CL@BC_t_ch_MAX | aic | PV | PV_OK | t_ch_MAX_OK | phyl_OK |
|---|---|---|---|---|---|---|---|---|---|
| **CJ.1** | 538 | 4 | 129.292122 | NaN | NaN | NaN | * | * | * |
| **BM.2** | 118 | 1 | 113.149675 | 112.499030 | -36.998060 | 5.107350e-08 | | | |
| **CJ.1.1** | 72 | 4 | 100.126282 | 100.126282 | -12.252564 | 2.156758e-13 | | * | * |
| **BM.1.1.1** | 41 | 11 | 93.280558 | 77.128381 | 33.743237 | 2.224333e-23 | | | * |
| **CV.2** | 64 | 6 | 89.391477 | 73.030135 | 41.939730 | 3.686315e-25 | | | |
| **BA.2.75** | 1935 | 4 | 86.539022 | 86.539022 | 14.921956 | 2.715882e-19 | | * | * |
| **BM.4.1.1** | 570 | 7 | 85.563232 | 85.169082 | 17.661836 | 6.901245e-20 | | | |
| **CB.1** | 240 | 4 | 84.720477 | 84.720477 | 18.559046 | 4.400428e-20 | | * | |
| **BA.2.75.5** | 645 | 4 | 83.331414 | 83.331414 | 21.337172 | 1.096038e-20 | | * | |
| **BA.2.75.3** | 384 | 4 | 83.316021 | 83.316021 | 21.367958 | 1.079720e-20 | | * | * |

Cumulative Likelihood per-region

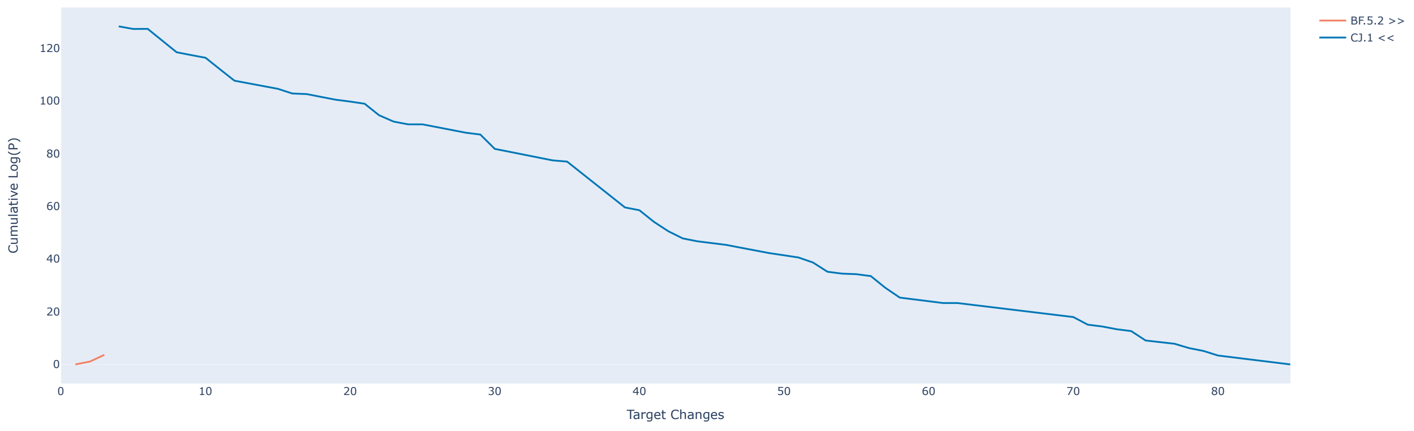


Cumulative Likelihood whole genome

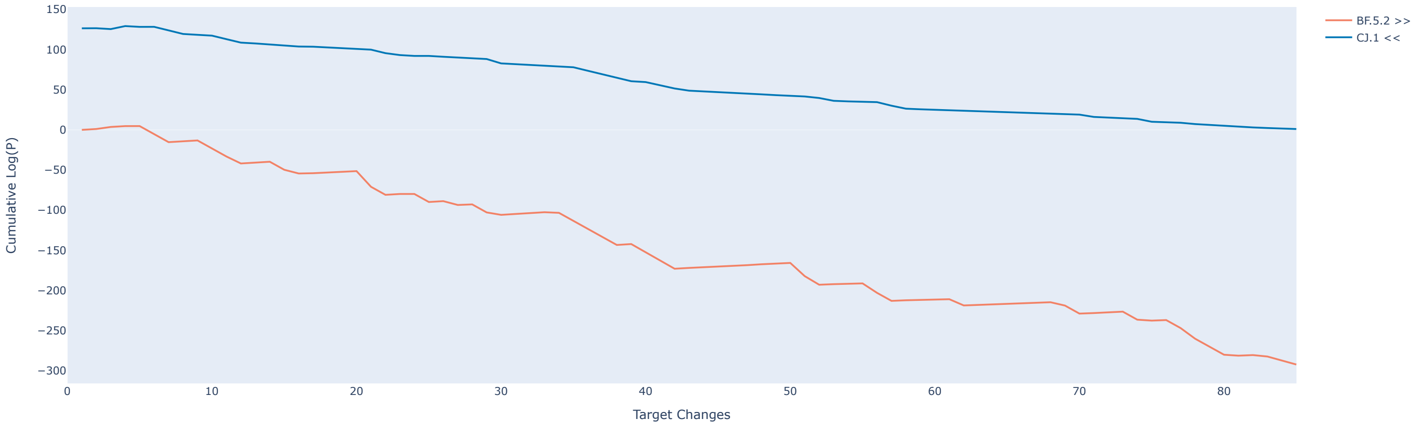


**Target sequence**

, 241_C|T, 670_T|G, 1627_C|T, 2790_C|T, 3037_C|T, 3796_C|T, 3927_C|T, 4184_G|A, 4321_C|T, 4586_C|T, 5183_C|T, 9344_C|T, 9424_A|G, 9534_C|T, 9866_C|T, 10029_C|T, 10198_C|T, 10447_G|A, 10449_C|A, 11288_TCTGGTTTT|........., 12444_A|G, 12574_T|C, 12880_C|T, 14408_C|T, 15451_G|A, 15714_C|T, 17410_C|T, 18163_A|G, 18583_G|A, 19955_C|T, 20055_A|G, 21618_C|T, 21633_TACCCCCTG|........., 21987_G|A, 22001_A|G, 22016_T|C, 22033_C|A, 22190_A|G, 22200_T|G, 22331_G|A, 22577_GG|CA, 22599_G|C, 22674_C|T, 22679_T|C, 22686_C|T, 22688_A|G, 22775_G|A, 22786_A|C, 22813_G|T, 22882_T|G, 22898_G|A, 22942_T|G, 22992_G|A, 22995_C|A, 23013_A|C, 23018_TT|CC, 23031_T|C, 23055_A|G, 23063_A|T, 23075_T|C, 23403_A|G, 23525_C|T, 23599_T|G, 23604_C|A, 23854_C|A, 23948_G|T, 24424_A|T, 24469_T|A, 25000_C|T, 25416_C|T, 25584_C|T, 26060_C|T, 26270_C|T, 26275_A|G, 26577_C|G, 26709_G|A, 26858_C|T, 27259_A|C, 27382_GAT|CTC, 27807_C|T, 28271_A|T, 28311_C|T, 28362_GAGAACGCA|........., 28881_GGG|AAC, 29510_A|C

**Supplementary Notes 3**

RecombinHunt output when run on the lineage consensus-genomes created using Nextstrain SARS-CoV-2 genome sequences. One result is provided for each retained Pango lineage (that passed quality controls and for which an unambiguous ground truth is available). Results correspond to those shown in Table 4.

## Case 1 (1BP mid): XA                                test: OK

Target: (75%) 33 samples
Number of changes: 36
GT: B.1.177 + B.1.1.7
GT BR: 12–14
GT BR coord: 21254 – 21765
Rank L1 L2: 2 1
BC: B.1.177.18 + B.1.1.7
BC BR: 13–14
BC BR coord: 21300 – 21301
Direction L1: <<
Initial region span: 1–13,14–36
Gap history (edge excluded):
Alt. candidates: [], []
Model 1BP/2BP comparison: –
Rec. model vs L1: 1.24e-202
Rec. model vs L2: 6.86e-101
Flags: Model_2BP_Bad_L1_opp

### B.1.177.18 >>

| | num_seq | t_ch_MAX | max_CL | CL@BC_t_ch_MAX | aic | PV | PV_OK | t_ch_MAX_OK | phyl_OK |
|---|---|---|---|---|---|---|---|---|---|
| **B.1.177.18** | 395 | 13 | 31.403269 | NaN | NaN | NaN | * | * | * |
| **B.1.177** | 34152 | 8 | 8.891676 | -8.623179 | 47.246358 | 4.122796e-18 | | | * |
| **B.1.177.44** | 304 | 4 | 3.510834 | -45.289495 | 120.578991 | 4.917957e-34 | | | |

### B.1.1.7 <<

| | num_seq | t_ch_MAX | max_CL | CL@BC_t_ch_MAX | aic | PV | PV_OK | t_ch_MAX_OK | phyl_OK |
|---|---|---|---|---|---|---|---|---|---|
| **B.1.1.7** | 405547 | 14 | 34.611559 | NaN | NaN | NaN | * | * | * |
| **Q.1** | 511 | 25 | 18.876925 | 12.310140 | 27.379720 | 2.066489e-10 | | | * |
| **Q.3** | 2418 | 18 | 17.365214 | 11.027517 | 29.944966 | 5.745610e-11 | | | * |
| **Q.7** | 4477 | 25 | 16.792133 | 10.116308 | 31.767384 | 2.301212e-11 | | | * |
| **Q.4** | 518 | 28 | 13.787988 | -9.792570 | 71.585141 | 5.189835e-20 | | | * |

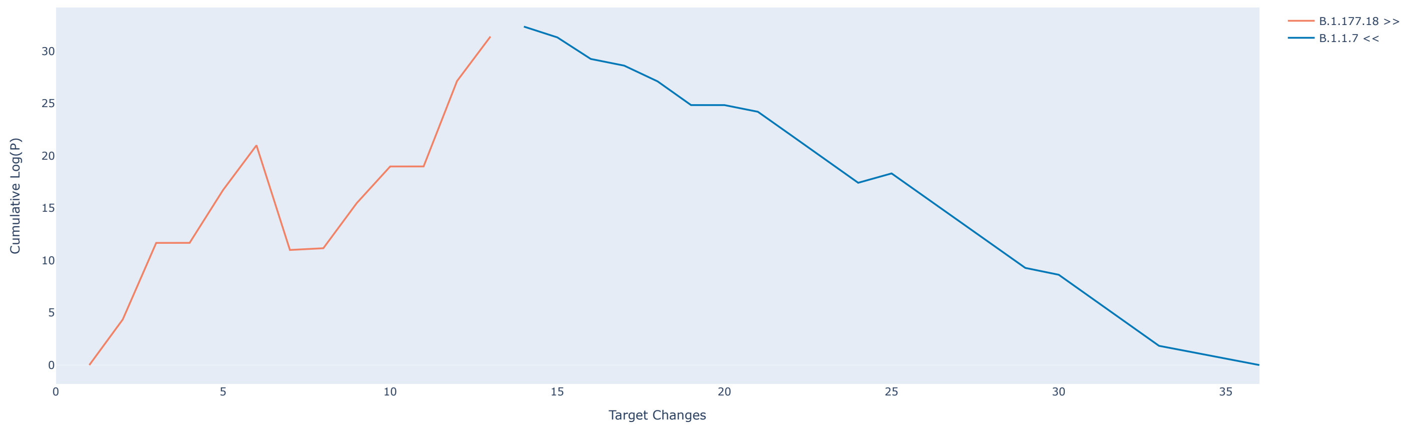


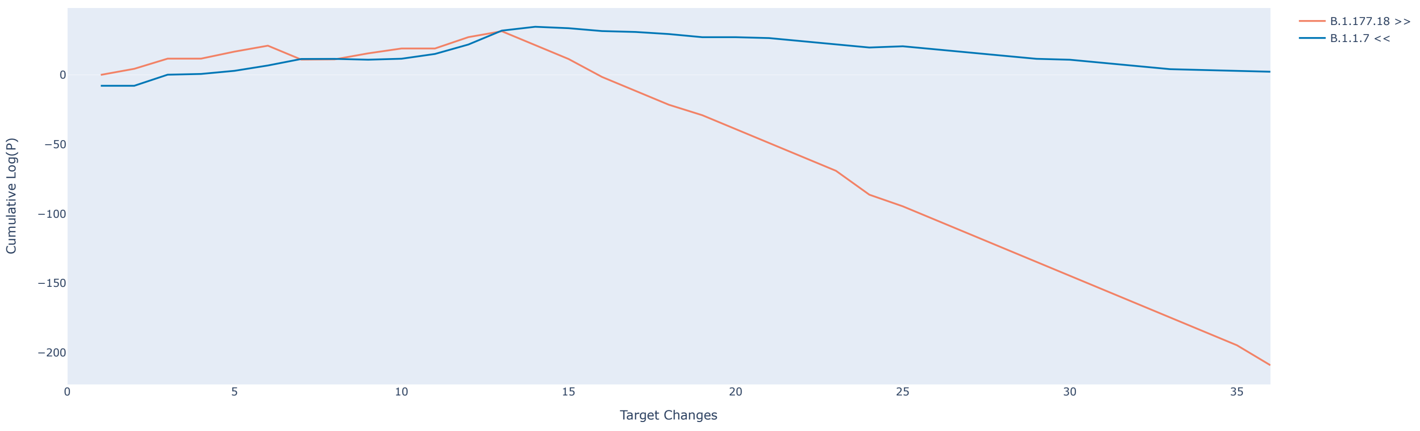


**Target sequence**

, 241_C|T, 445_T|C, 2019_T|C, 3037_C|T, 4999_C|T, 6286_C|T, 8090_C|T, 9430_C|T, 10323_A|G, 13945_C|T, 14408_C|T, 20410_G|A, 21255_G|C, 21765_21770, 21992_21994, 23063_A|T, 23208_C|T, 23271_C|A, 23403_A|G, 23604_C|A, 23709_C|T, 24506_T|G, 24914_G|C, 25855_G|T, 27972_C|T, 28048_G|T, 28095_A|T, 28111_A|G, 28271_28271, 28280_G|C, 28281_A|T, 28282_T|A, 28881_G|A, 28882_G|A, 28883_G|C, 28977_C|T

## Case 2 (1BP mid): XAD                                test: OK

Target: (75%) 11 samples
GT: BA.2* + BA.1*
BC: BA.2 + BA.1
Direction L1: >>
Alt. candidates: [BA.2.34], [BA.1.14.1, BA.1.14, BA.1.1.2, BA.1.1.1, BA.1.1.12, BA.1.9, BC.1, BA.1.17]
Model 1BP/2BP comparison:
Rec. model vs L1: 4.11e-21
Flags: Model_1BP_Best

Number of changes: 69
GT BR: 55–57
GT BR coord: 26062 – 26529
Rank L1 L2: 1 1
BC BR: 56–57
BC BR coord: 26274 – 26275
Initial region span: 1–56,57–69
Gap history (edge excluded):
1BP vs 2BP: 4.57e-06
Rec. model vs L2: 4.03e-170

## BA.2 >>

| | num_seq | t_ch_MAX | max_CL | CL@BC_t_ch_MAX | aic | PV | PV_OK | t_ch_MAX_OK | phyl_OK |
|---|---|---|---|---|---|---|---|---|---|
| **BA.2** | 359165 | 56 | 53.967442 | NaN | NaN | NaN | * | * | * |
| **BA.2.9** | 61803 | 54 | 52.203501 | 50.345599 | 27.308803 | 2.678268e-02 | * | | * |
| **BA.2.34** | 44 | 56 | 44.092869 | 44.092869 | 39.814262 | 5.170273e-05 | * | * | * |
| **BA.2.6** | 327 | 53 | 40.842282 | 35.930833 | 56.138334 | 1.470615e-08 | | | * |
| **BA.2.10.2** | 42 | 56 | 38.085272 | 38.085272 | 51.829457 | 1.268831e-07 | | * | * |
| **BA.2.7** | 1628 | 56 | 37.675167 | 37.675167 | 52.649665 | 8.420597e-08 | | * | * |
| **BA.2.49** | 212 | 56 | 37.524977 | 37.524977 | 52.950046 | 7.247675e-08 | | * | * |
| **BA.2.36** | 1320 | 56 | 36.968571 | 36.968571 | 54.062857 | 4.160689e-08 | | * | * |
| **BA.2.51** | 234 | 48 | 36.524675 | 36.507550 | 54.984900 | 2.626575e-08 | | | * |
| **BA.2.14** | 912 | 56 | 35.761882 | 35.761882 | 56.476236 | 1.240706e-08 | | * | * |

## BA.1 <<

| | num_seq | t_ch_MAX | max_CL | CL@BC_t_ch_MAX | aic | PV | PV_OK | t_ch_MAX_OK | phyl_OK |
|---|---|---|---|---|---|---|---|---|---|
| **BA.1** | 100089 | 57 | 13.319708 | NaN | NaN | NaN | * | * | * |
| **BA.1.14.1** | 181 | 57 | 11.854724 | 11.854724 | 16.290553 | 2.310780e-01 | * | * | * |
| **XBB.3.1** | 11 | 61 | 11.254619 | -6.650348 | 53.300697 | 2.123925e-09 | | | |
| **BA.1.14** | 1016 | 57 | 9.989341 | 9.989341 | 20.021318 | 3.579311e-02 | * | * | * |
| **BA.1.1.2** | 2970 | 57 | 9.967335 | 9.967335 | 20.065330 | 3.490937e-02 | * | * | * |
| **BA.1.1.1** | 8304 | 57 | 9.429079 | 9.429079 | 21.141842 | 2.044535e-02 | * | * | * |
| **BA.1.1.12** | 1943 | 57 | 9.333571 | 9.333571 | 21.332859 | 1.859244e-02 | * | * | * |
| **BA.1.9** | 186 | 57 | 9.050095 | 9.050095 | 21.899810 | 1.398178e-02 | * | * | * |
| **BC.1** | 209 | 57 | 8.822905 | 8.822905 | 22.354189 | 1.116468e-02 | * | * | * |
| **BA.1.17** | 6954 | 57 | 8.574815 | 8.574815 | 22.850369 | 8.695062e-03 | * | * | * |

Cumulative Likelihood per-region

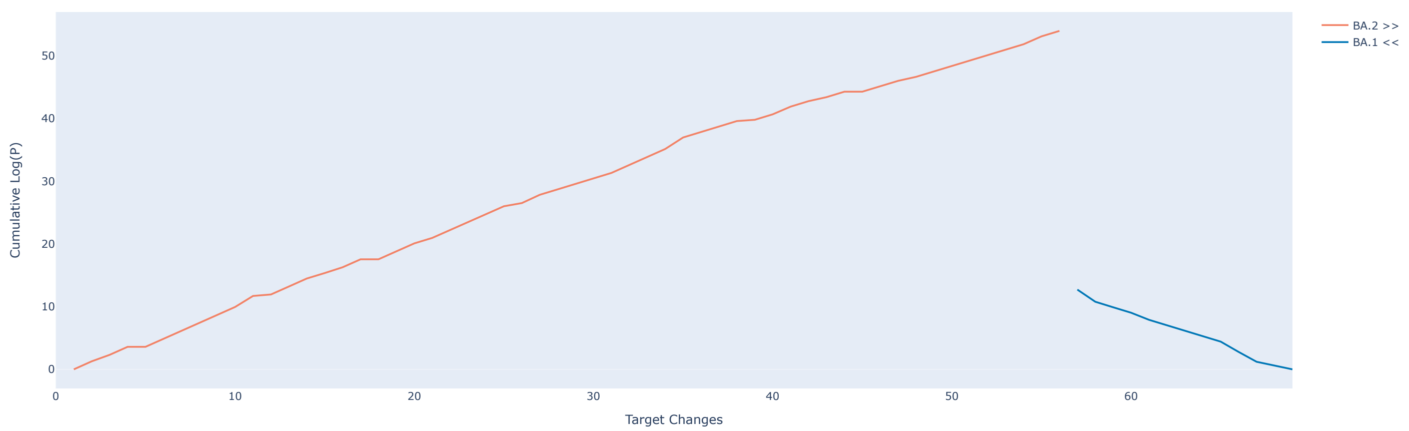


Cumulative Likelihood whole genome

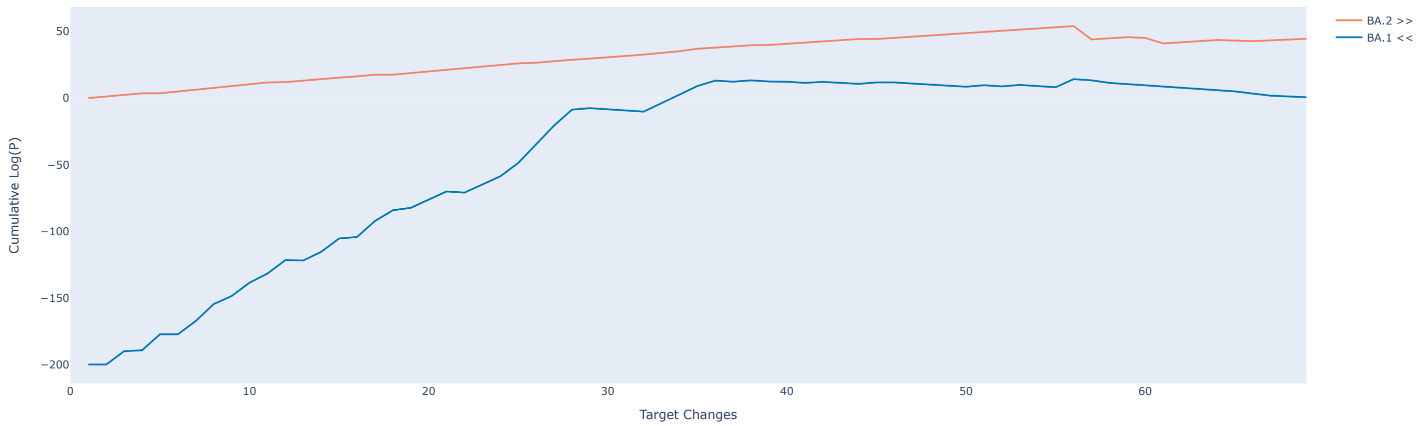


**Target sequence**

, 241_C|T, 670_T|G, 1250_A|G, 2790_C|T, 3037_C|T, 4184_G|A, 4321_C|T, 9344_C|T, 9424_A|G, 9534_C|T, 9866_C|T, 10029_C|T, 10198_C|T, 10447_G|A, 10449_C|A, 11288_11296, 12880_C|T, 14408_C|T, 15714_C|T, 17410_C|T, 18163_A|G, 19955_C|T, 20055_A|G, 21618_C|T, 21633_21641, 21987_G|A, 22200_T|G, 22578_G|A, 22674_C|T, 22679_T|C, 22686_C|T, 22688_A|G, 22775_G|A, 22786_A|C, 22792_C|T, 22813_G|T, 22882_T|G, 22992_G|A, 22995_C|A, 23013_A|C, 23040_A|G, 23055_A|G, 23063_A|T, 23075_T|C, 23403_A|G, 23525_C|T, 23599_T|G, 23604_C|A, 23854_C|A, 23948_G|T, 24424_A|T, 24469_T|A, 25000_C|T, 25584_C|T, 26060_C|T, 26270_C|T, 26530_A|G, 26577_C|G, 26709_G|A, 27259_A|C, 27807_C|T, 28271_A|T, 28311_C|T, 28362_28370, 28877_A|T, 28878_G|C, 28881_G|A, 28882_G|A, 28883_G|C

## Case 3 (1BP mid): XAE test: OK

Target: (75%) 18 samples
Number of changes: 72
GT: BA.2* + BA.1*
GT BR: 55-58
GT BR coord: 24505 - 26049
Rank L1 L2: 1 1
BC: BA.2 + BA.1
BC BR: 57-58
BC BR coord: 25593 - 25594
Direction L1: >>
Initial region span: 1-57,58-72
Gap history (edge excluded):
Alt. candidates: [], [BA.1.1, BA.1.14.1, BA.1.14]
Model 1BP/2BP comparison: -
Rec. model vs L1: 1.11e-28
Rec. model vs L2: 1.85e-168
Flags: Model_2BP_Bad_L1_opp

## BA.2 >>

| | num_seq | t_ch_MAX | max_CL | CL@BC_t_ch_MAX | aic | PV | PV_OK | t_ch_MAX_OK | phyl_OK |
|---|---|---|---|---|---|---|---|---|---|
| **BA.2** | 359165 | 57 | 51.259609 | NaN | NaN | NaN | * | * | * |
| **BA.2.9** | 61803 | 57 | 36.899966 | 36.899966 | 76.200068 | 5.801379e-07 | | * | * |
| **BA.2.10** | 12936 | 57 | 30.574327 | 30.574327 | 88.851346 | 1.039007e-09 | | * | * |
| **BA.2.12.1** | 94944 | 56 | 29.065180 | 27.021734 | 95.956531 | 2.969626e-11 | | * | * |
| **BA.2.3** | 22517 | 57 | 23.762261 | 23.762261 | 102.475479 | 1.139992e-12 | | * | * |
| **BA.2.18** | 3723 | 57 | 21.694697 | 21.694697 | 106.610607 | 1.445718e-13 | | * | * |
| **BA.2.23** | 8100 | 57 | 16.569198 | 16.569198 | 116.861603 | 8.596555e-16 | | * | * |
| **BA.2.20** | 357 | 57 | 15.793425 | 15.793425 | 118.413151 | 3.960465e-16 | | * | * |
| **BA.5.1** | 33374 | 43 | 11.700752 | 9.439929 | 131.120142 | 6.883428e-19 | | | |
| **BA.2.35** | 21 | 7 | 9.903515 | 7.374104 | 135.251792 | 8.729446e-20 | | | * |

## BA.1 <<

| | num_seq | t_ch_MAX | max_CL | CL@BC_t_ch_MAX | aic | PV | PV_OK | t_ch_MAX_OK | phyl_OK |
|---|---|---|---|---|---|---|---|---|---|
| **BA.1** | 100089 | 58 | 12.359106 | NaN | NaN | NaN | * | * | * |
| **BA.1.1** | 262925 | 58 | 8.403370 | 8.403370 | 39.193259 | 1.915867e-02 | * | * | * |
| **P.1.7** | 359 | 67 | 8.311404 | -95.836738 | 247.673476 | 1.026854e-47 | | | |
| **BA.1.14.1** | 181 | 58 | 2.725271 | 2.725271 | 50.549457 | 6.539923e-05 | * | * | * |
| **XBB.3.1** | 11 | 63 | 1.254619 | -17.035436 | 90.070873 | 1.713617e-13 | | | |
| **BA.1.14** | 1016 | 58 | 0.859889 | 0.859889 | 54.280222 | 1.013009e-05 | * | * | * |
| **BA.1.1.2** | 2970 | 58 | 0.836535 | 0.836535 | 54.326930 | 9.879981e-06 | | * | * |
| **BA.1.1.1** | 8304 | 58 | 0.296974 | 0.296974 | 55.406053 | 5.757541e-06 | | * | * |
| **B.1.1** | 16129 | 69 | 0.215146 | -69.045555 | 194.091111 | 4.428511e-36 | | | * |
| **BA.1.1.12** | 1943 | 58 | 0.204118 | 0.204118 | 55.591763 | 5.261997e-06 | | * | * |

Cumulative Likelihood per-region

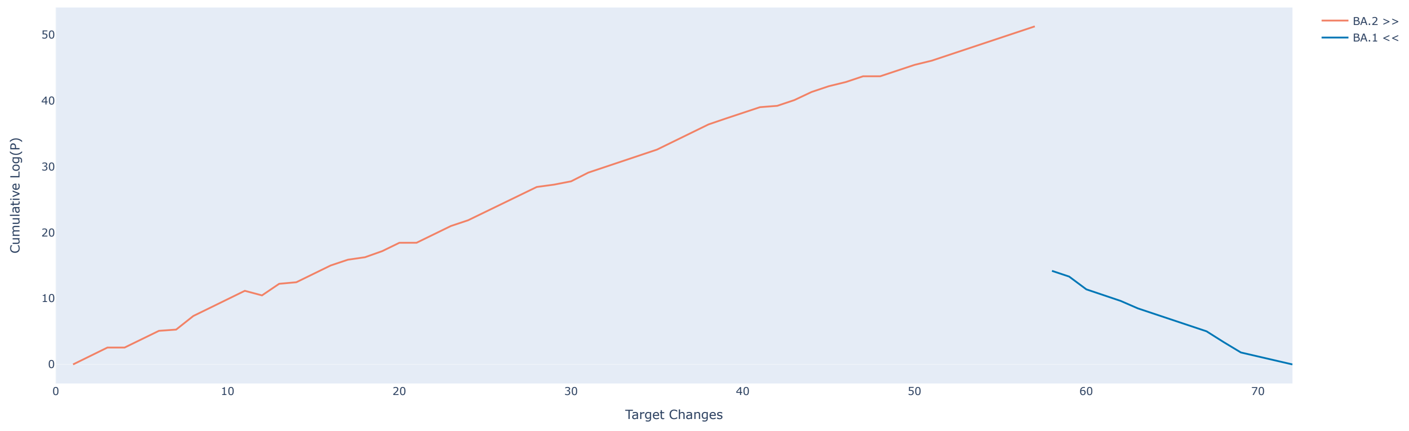


Cumulative Likelihood whole genome

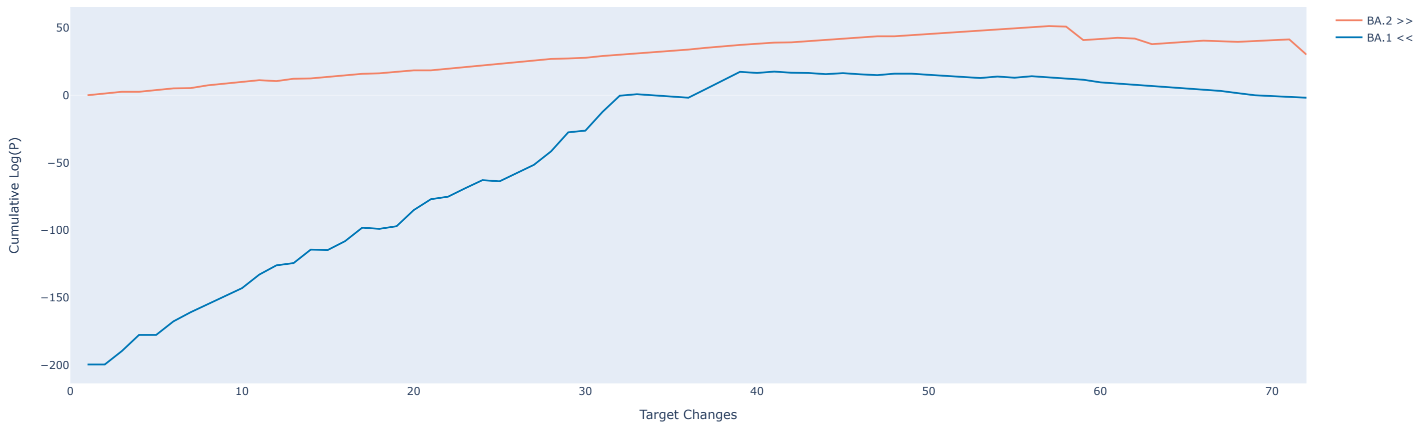


**Target sequence**

, 241_C|T, 670_T|G, 2790_C|T, 3037_C|T, 4184_G|A, 4321_C|T, 4975_G|A, 6390_A|C, 9344_C|T, 9424_A|G, 9534_C|T, 9803_C|T, 9866_C|T, 10029_C|T, 10198_C|T, 10447_G|A, 10449_C|A, 11249_C|T, 11288_11296, 12880_C|T, 14408_C|T, 15714_C|T, 17410_C|T, 18163_A|G, 19955_C|T, 20055_A|G, 21618_C|T, 21633_21641, 21859_C|T, 21987_G|A, 22200_T|G, 22578_G|A, 22674_C|T, 22679_T|C, 22686_C|T, 22688_A|G, 22775_G|A, 22786_A|C, 22813_G|T, 22882_T|G, 22992_G|A, 22995_C|A, 23013_A|C, 23040_A|G, 23055_A|G, 23063_A|T, 23075_T|C, 23403_A|G, 23525_C|T, 23599_T|G, 23604_C|A, 23854_C|A, 23948_G|T, 24424_A|T, 24469_T|A, 25000_C|T, 25584_C|T, 26270_C|T, 26530_A|G, 26577_C|G, 26709_G|A, 27259_A|C, 27807_C|T, 28271_A|T, 28311_C|T, 28362_28370, 28877_A|T, 28878_G|C, 28881_G|A, 28882_G|A, 28883_G|C, 29748_T|C

## Case 4 (1BP mid): XAL    test: OK

Target: (75%) 13 samples    Number of changes: 67
GT: BA.1* + BA.2*    GT BR: 17–19    GT BR coord: 17412 – 19954    Rank L1 L2: 11 1
BC: BA.1.1 + BA.2    BC BR: 17–18    BC BR coord: 17426 – 17427
Direction L1: <<    Initial region span: 1–17,18–67    Gap history (edge excluded):
Alt. candidates: [], []
Model 1BP/2BP comparison: –
Rec. model vs L1: 2.40e−104    Rec. model vs L2: 1.96e−86
Flags: Model_2BP_Bad_L1_opp

## BA.1.1 >>

| | num_seq | t_ch_MAX | max_CL | CL@BC_t_ch_MAX | aic | PV | PV_OK | t_ch_MAX_OK | phyl_OK |
|---|---|---|---|---|---|---|---|---|---|
| **BA.1.1** | 262925 | 17 | 15.957903 | NaN | NaN | NaN | * | * | * |
| **BA.1.1.9** | 172 | 3 | 4.528654 | -19.230206 | 88.460412 | 5.214074e-16 | | | * |
| **BA.1.1.6** | 22 | 3 | 4.528654 | -16.537171 | 83.074342 | 7.719707e-15 | | | * |
| **BA.1.1.12** | 1943 | 3 | 4.528654 | -18.169963 | 86.339926 | 1.504975e-15 | | | * |
| **BA.1.1.15** | 5038 | 3 | 4.528058 | -23.656666 | 97.313332 | 6.243442e-18 | | | * |
| **BA.1.1.13** | 3393 | 3 | 4.527769 | -10.663911 | 71.327823 | 2.734698e-12 | | | * |
| **BA.1.1.14** | 5810 | 3 | 4.524864 | -17.188576 | 84.377152 | 4.009940e-15 | | | * |
| **BA.1.1.2** | 2970 | 3 | 4.521573 | -17.878802 | 85.757604 | 2.011290e-15 | | | * |
| **BA.1.1.16** | 594 | 3 | 4.518527 | -10.677243 | 71.354485 | 2.707488e-12 | | | * |
| **BA.1.1.18** | 22283 | 3 | 4.515195 | -14.120767 | 78.241535 | 8.638175e-14 | | | * |

## BA.2 <<

| | num_seq | t_ch_MAX | max_CL | CL@BC_t_ch_MAX | aic | PV | PV_OK | t_ch_MAX_OK | phyl_OK |
|---|---|---|---|---|---|---|---|---|---|
| **BA.2** | 359165 | 18 | 44.674959 | NaN | NaN | NaN | * | * | * |
| **BA.2.40** | 27 | 22 | 43.952678 | 30.628348 | 48.743305 | 7.949394e-07 | | | * |
| **BA.2.27** | 19 | 22 | 43.141748 | 36.565680 | 36.868640 | 3.005189e-04 | * | | * |
| **BA.2.11** | 22 | 22 | 42.701511 | 36.125443 | 37.749114 | 1.935451e-04 | * | | * |
| **BA.2.63** | 46 | 22 | 41.984653 | 26.542213 | 56.915574 | 1.330668e-08 | | | * |
| **BA.2.23** | 8100 | 22 | 41.808991 | 35.200501 | 39.598998 | 7.674671e-05 | * | | * |
| **BA.2.1** | 7140 | 22 | 41.776744 | 35.162927 | 39.674147 | 7.410704e-05 | * | | * |
| **BA.2.10** | 12936 | 22 | 41.730847 | 35.117110 | 39.765780 | 7.049280e-05 | * | | * |
| **BA.2.31** | 453 | 22 | 41.701241 | 35.118529 | 39.762943 | 7.084615e-05 | * | | * |
| **BA.2.19** | 114 | 22 | 41.692648 | 35.107769 | 39.784461 | 7.014121e-05 | * | | * |

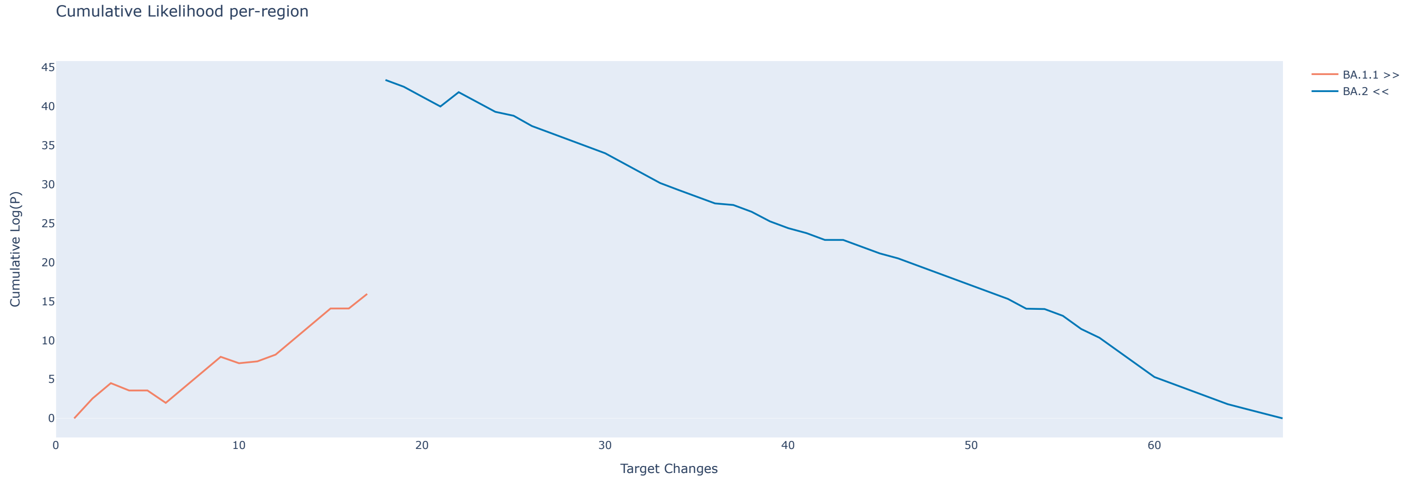


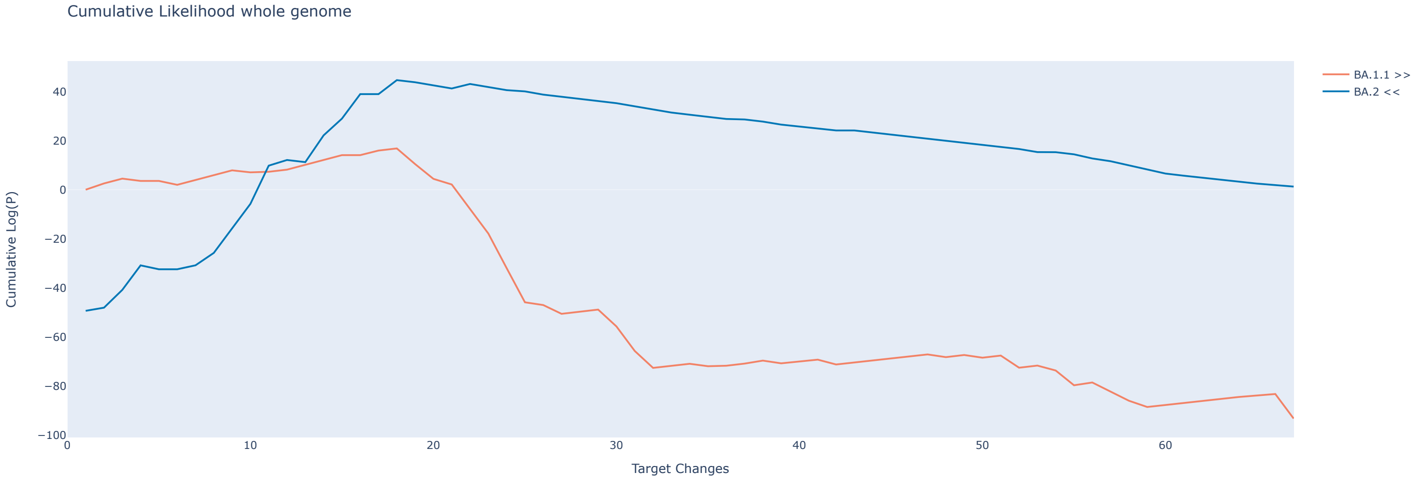


**Target sequence**

, 241_C|T, 2470_C|T, 2832_A|G, 2865_A|G, 3037_C|T, 3880_G|A, 5386_T|G, 6513_6515, 8393_G|A, 9136_G|T, 10029_C|T, 10449_C|A, 11285_11293, 11537_A|G, 13195_T|C, 14408_C|T, 15240_C|T, 18163_A|G, 19955_C|T, 20055_A|G, 21586_G|T, 21618_C|T, 21633_21641, 21987_G|A, 22200_T|G, 22578_G|A, 22674_C|T, 22679_T|C, 22686_C|T, 22688_A|G, 22775_G|A, 22786_A|C, 22813_G|T, 22882_T|G, 22992_G|A, 22995_C|A, 23013_A|C, 23040_A|G, 23055_A|G, 23063_A|T, 23075_T|C, 23403_A|G, 23525_C|T, 23599_T|G, 23604_C|A, 23854_C|A, 23948_G|T, 24424_A|T, 24469_T|A, 25000_C|T, 25584_C|T, 26060_C|T, 26270_C|T, 26709_G|A, 26858_C|T, 27259_A|C, 27382_G|C, 27383_A|T, 27384_T|C, 27807_C|T, 28271_A|T, 28311_C|T, 28362_28370, 28881_G|A, 28882_G|A, 28883_G|C, 29510_A|C

## Case 5 (1BP mid): XAN test: OK

Target: (75%) 36 samples
GT: BA.2* + BA.5.1
BC: BA.2 + BA.5.1.23
Direction L1: <<
Alt. candidates: [BA.2.9, BA.2.12.1], [BA.5.1]
Model 1BP/2BP comparison:
Rec. model vs L1: 2.19e-69
Flags: Model_2BP_Bad_L1_opp

Number of changes: 72
GT BR: 21-28
BC BR: 11-12
Initial region span: 1-11,12-72
-
Rec. model vs L2: 4.03e-17

GT BR coord: 17822 - 21766
BC BR coord: 9866 - 9867
Gap history (edge excluded):

Rank L1 L2: 1 2

## BA.2 >>

| | num_seq | t_ch_MAX | max_CL | CL@BC_t_ch_MAX | aic | PV | PV_OK | t_ch_MAX_OK | phyl_OK |
|---|---|---|---|---|---|---|---|---|---|
| BA.2 | 359165 | 11 | 12.597424 | NaN | NaN | NaN | * | * | * |
| BA.2.9 | 61803 | 11 | 10.328386 | 10.328386 | 9.343229 | 1.038300e-01 | * | * | * |
| BA.2.12.1 | 94944 | 11 | 10.310540 | 10.310540 | 9.378919 | 1.017741e-01 | * | * | * |
| BA.5.2.20 | 3367 | 10 | 8.377130 | -1.622870 | 33.245740 | 6.673174e-07 | | * | |
| BE.1 | 5712 | 10 | 6.648242 | -3.351758 | 36.703515 | 1.188980e-07 | | * | |
| XBB.1.5 | 35885 | 11 | 6.555955 | 6.555955 | 16.888090 | 2.381559e-03 | * | * | |
| BA.5.1.17 | 115 | 6 | 5.104312 | -11.089421 | 52.178843 | 5.172913e-11 | | | |
| BA.2.35 | 21 | 6 | 5.104312 | -5.055609 | 40.111219 | 2.161237e-08 | | | * |
| BA.2.8 | 4251 | 6 | 5.104312 | 0.697170 | 28.605659 | 6.790405e-06 | | | * |
| BT.1 | 55 | 6 | 5.104312 | -11.071877 | 52.143754 | 5.277413e-11 | | | |

## BA.5.1.23 <<

| | num_seq | t_ch_MAX | max_CL | CL@BC_t_ch_MAX | aic | PV | PV_OK | t_ch_MAX_OK | phyl_OK |
|---|---|---|---|---|---|---|---|---|---|
| BA.5.1.23 | 2767 | 12 | 64.508225 | NaN | NaN | NaN | * | * | * |
| BA.5.1 | 33374 | 12 | 58.031158 | 58.031158 | 29.937683 | 1.533811e-03 | * | * | * |
| DE.1 | 74 | 22 | 49.569307 | 45.730940 | 54.538120 | 6.981514e-09 | | | * |
| BA.5.1.22 | 3125 | 22 | 49.543797 | 39.420375 | 67.159251 | 1.269262e-11 | | | |
| BA.5.1.6 | 980 | 12 | 46.677980 | 46.677980 | 52.644040 | 1.805217e-08 | | * | |
| BA.5.1.1 | 4192 | 12 | 43.890951 | 43.890951 | 58.218098 | 1.108786e-09 | | * | |
| BA.5.1.4 | 313 | 23 | 42.747702 | 22.165342 | 101.669316 | 4.071929e-19 | | | |
| BA.5.1.12 | 412 | 12 | 42.641714 | 42.641714 | 60.716571 | 3.176725e-10 | | * | |
| BA.5.1.3 | 1626 | 12 | 42.602836 | 42.602836 | 60.794328 | 3.067463e-10 | | * | |
| BA.5.1.24 | 1230 | 22 | 40.272407 | 30.184810 | 85.630379 | 1.238346e-15 | | | |

Cumulative Likelihood per-region

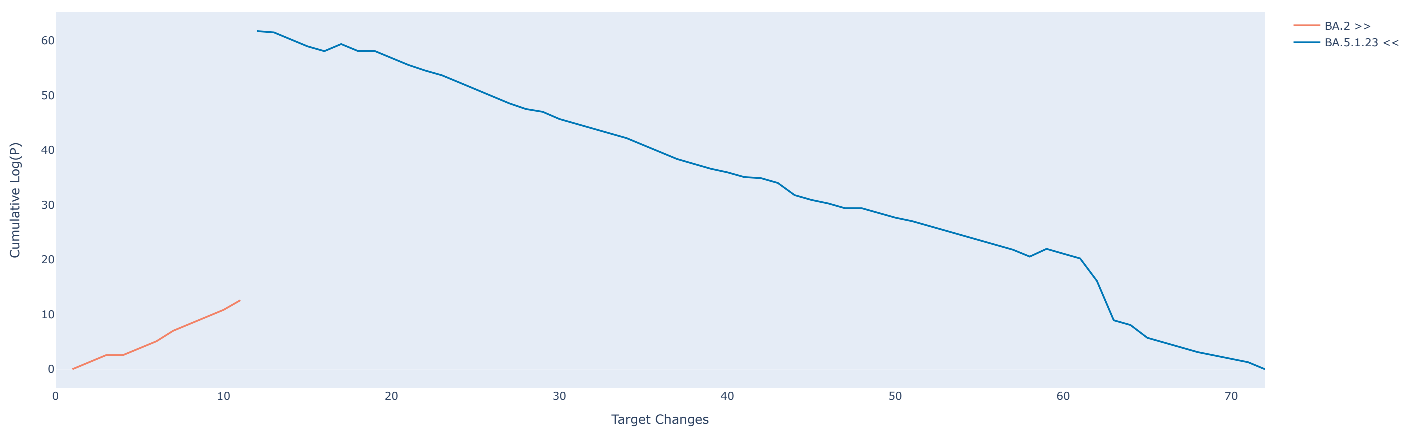


Cumulative Likelihood whole genome

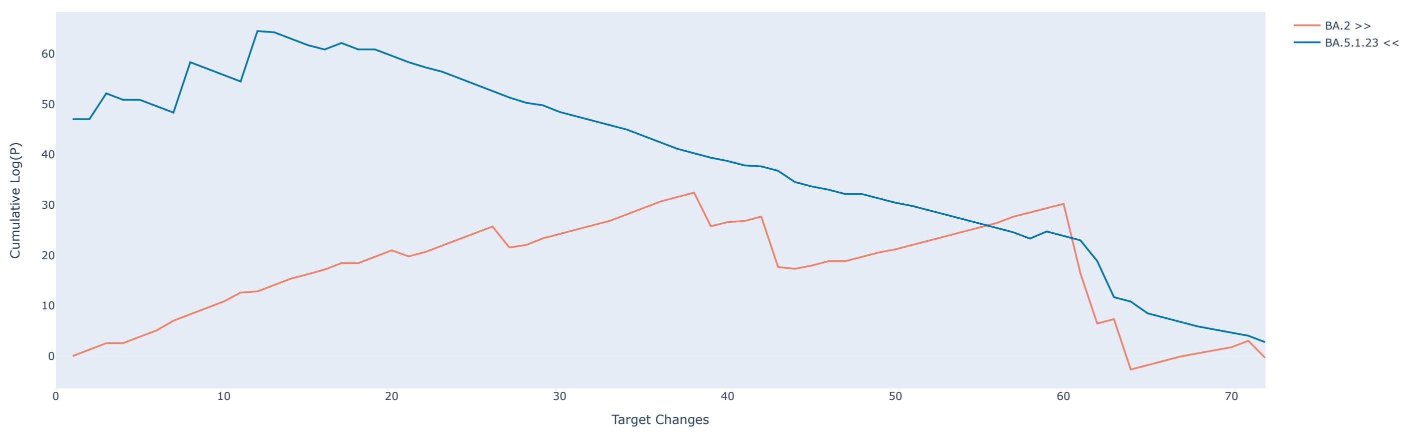


**Target sequence**

, 241_C|T, 670_T|G, 2790_C|T, 3037_C|T, 4184_G|A, 4321_C|T, 7973_C|A, 9344_C|T, 9424_A|G, 9534_C|T, 9866_C|T, 10029_C|T, 10198_C|T, 10447_G|A, 10449_C|A, 11288_11296, 12880_C|T, 14408_C|T, 15714_C|T, 17410_C|T, 17822_C|T, 18163_A|G, 19955_C|T, 20055_A|G, 21618_C|T, 21633_21641, 21765_21770, 21987_G|A, 22200_T|G, 22578_G|A, 22674_C|T, 22679_T|C, 22686_C|T, 22688_A|G, 22775_G|A, 22786_A|C, 22813_G|T, 22882_T|G, 22917_T|G, 22992_G|A, 22995_C|A, 23013_A|C, 23018_T|G, 23055_A|G, 23063_A|T, 23075_T|C, 23403_A|G, 23525_C|T, 23599_T|G, 23604_C|A, 23854_C|A, 23948_G|T, 24424_A|T, 24469_T|A, 25000_C|T, 25584_C|T, 26060_C|T, 26270_C|T, 26577_C|G, 26709_G|A, 27438_T|C, 27696_T|C, 27807_C|T, 27889_C|T, 28271_A|T, 28311_C|T, 28362_28370, 28881_G|A, 28882_G|A, 28883_G|C, 29510_A|C, 29666_C|T

## Case 6 (1BP mid): XAP          test: OK

Target: (75%) 261 samples   Number of changes: 65
GT: BA.2* + BA.1*   GT BR: 53–55   GT BR coord: 26062 – 26529   Rank L1 L2: 2 11
BC: BA.2.65 + BA.1.23   BC BR: 54–55   BC BR coord: 26274 – 26275
Direction L1: >>   Initial region span: 1–54,55–65   Gap history (edge excluded):
Alt. candidates: [BA.2], []
Model 1BP/2BP comparison:   1BP vs 2BP: 2.42e–05
Rec. model vs L1: 9.79e–70   Rec. model vs L2: 0.00e+00
Flags: Model_1BP_Best

## BA.2.65 >>

| | num_seq | t_ch_MAX | max_CL | CL@BC_t_ch_MAX | aic | PV | PV_OK | t_ch_MAX_OK | phyl_OK |
|---|---|---|---|---|---|---|---|---|---|
| **BA.2.65** | 815 | 54 | 51.185310 | NaN | NaN | NaN | * | * | * |
| **BA.2** | 359165 | 54 | 51.098737 | 51.098737 | 23.802526 | 9.185123e-01 | * | * | * |
| **BA.2.62** | 65 | 52 | 48.900643 | 44.034171 | 37.931658 | 7.848641e-04 | * | | |
| **BA.2.34** | 44 | 54 | 48.400996 | 48.400996 | 29.198008 | 6.172909e-02 | * | * | |
| **BA.2.59** | 62 | 51 | 48.273031 | 36.287362 | 53.425277 | 3.380743e-07 | | | |
| **BA.2.17** | 37 | 51 | 48.267015 | 38.220172 | 49.559655 | 2.340842e-06 | | | |
| **BA.2.10.2** | 42 | 54 | 47.185245 | 47.185245 | 31.629510 | 1.831564e-02 | * | * | |
| **BA.2.10** | 12936 | 54 | 47.090195 | 47.090195 | 31.819611 | 1.665575e-02 | * | * | |
| **BA.2.9** | 61803 | 52 | 46.024061 | 44.166158 | 37.667684 | 8.938255e-04 | * | | |
| **BA.2.32** | 346 | 54 | 45.459904 | 45.459904 | 35.080193 | 3.263353e-03 | * | * | |

## BA.1.23 <<

| | num_seq | t_ch_MAX | max_CL | CL@BC_t_ch_MAX | aic | PV | PV_OK | t_ch_MAX_OK | phyl_OK |
|---|---|---|---|---|---|---|---|---|---|
| **BA.1.23** | 12 | 55 | 10.223988 | NaN | NaN | NaN | * | * | * |
| **BA.1.5** | 274 | 55 | 10.223988 | 10.223988 | 1.552024 | 1.000000 | * | * | |
| **BA.1.1.12** | 1943 | 55 | 10.219352 | 10.219352 | 1.561295 | 0.995012 | * | * | |
| **BC.1** | 209 | 55 | 10.219192 | 10.219192 | 1.561616 | 0.995012 | * | * | |
| **BA.1.9** | 186 | 55 | 10.213207 | 10.213207 | 1.573587 | 0.990050 | * | * | |
| **BA.1.1.13** | 3393 | 55 | 10.212778 | 10.212778 | 1.574444 | 0.990050 | * | * | |
| **BA.1.1.17** | 191 | 55 | 10.208157 | 10.208157 | 1.583687 | 0.985112 | * | * | |
| **BA.1.10** | 459 | 55 | 10.202140 | 10.202140 | 1.595720 | 0.975310 | * | * | |
| **BD.1** | 2101 | 55 | 10.197169 | 10.197169 | 1.605662 | 0.970446 | * | * | |
| **BA.1.1.9** | 172 | 55 | 10.194627 | 10.194627 | 1.610746 | 0.970446 | * | * | |

Cumulative Likelihood per-region

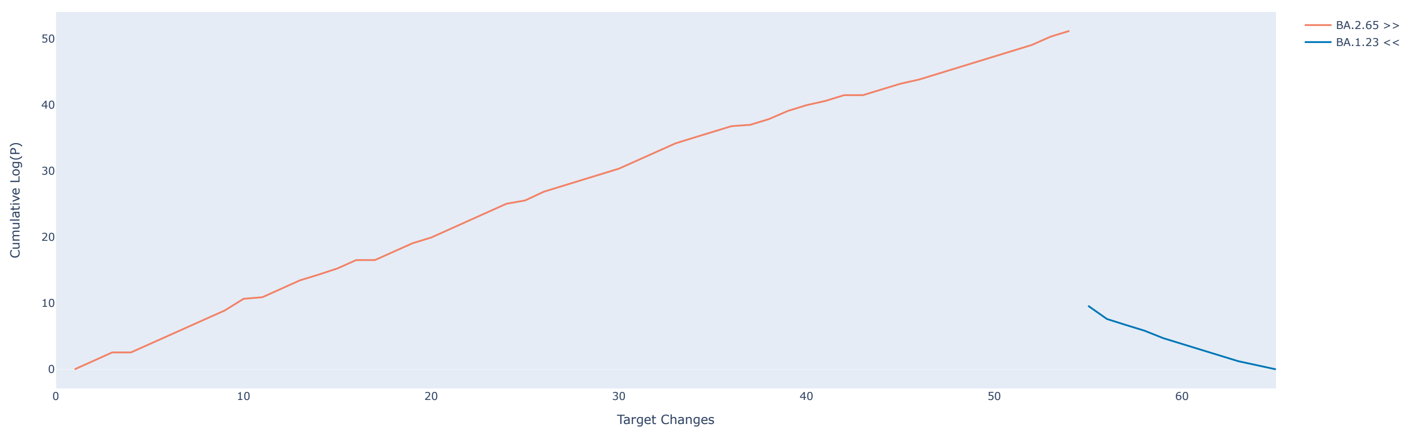


Cumulative Likelihood whole genome

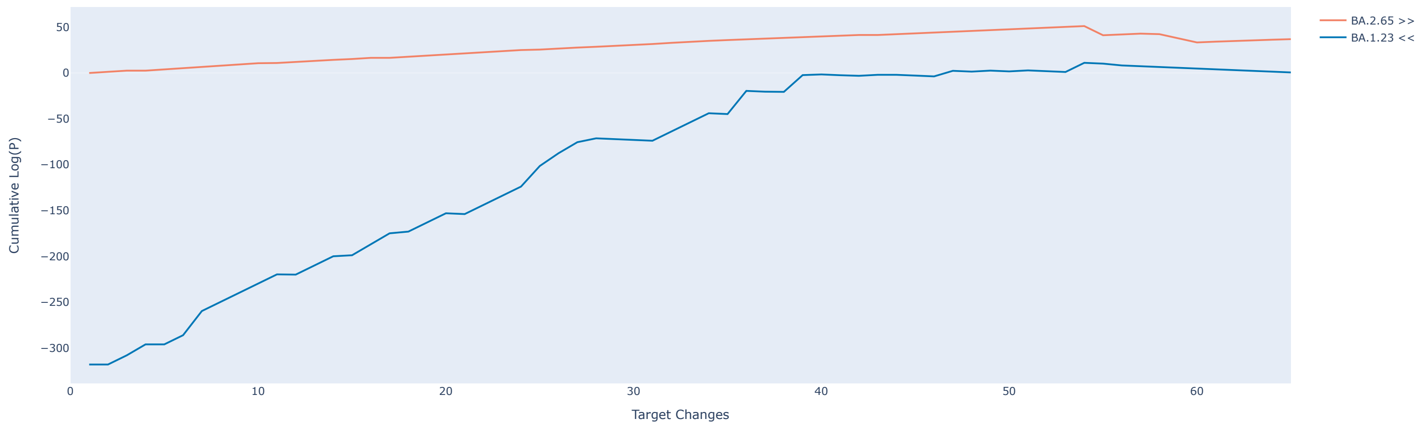


**Target sequence**

, 241_C|T, 670_T|G, 2790_C|T, 3037_C|T, 4184_G|A, 4321_C|T, 9344_C|T, 9424_A|G, 9534_C|T, 9866_C|T, 10029_C|T, 10198_C|T, 10447_G|A, 10449_C|A, 11288_11296, 12880_C|T, 14408_C|T, 15714_C|T, 17410_C|T, 18163_A|G, 19955_C|T, 20055_A|G, 21618_C|T, 21633_21641, 21987_G|A, 22200_T|G, 22578_G|A, 22674_C|T, 22679_T|C, 22686_C|T, 22688_A|G, 22775_G|A, 22786_A|C, 22813_G|T, 22882_T|G, 22992_G|A, 22995_C|A, 23013_A|C, 23040_A|G, 23055_A|G, 23063_A|T, 23075_T|C, 23403_A|G, 23525_C|T, 23599_T|G, 23604_C|A, 23854_C|A, 23948_G|T, 24424_A|T, 24469_T|A, 25000_C|T, 25584_C|T, 26060_C|T, 26270_C|T, 26530_A|G, 26577_C|G, 26709_G|A, 27259_A|C, 27807_C|T, 28271_A|T, 28311_C|T, 28362_28370, 28881_G|A, 28882_G|A, 28883_G|C

## Case 7 (1BP mid): XAS        test: KO

Target: (75%) 161 samples
GT: BA.5* + BA.2*
BC: B.1.1.529
Direction L1: >>
Alt. candidates: [BA.4, BA.4.1]
Model 1BP/2BP comparison:
Rec. model vs L1: –
Flags: Model_1BP_L1eqL2, Model_2BP_NotEnoughSpace_ForL2, Model_2BP_Bad_L2, SingleCandidateGenome

Number of changes: 73
GT BR: 41–64        GT BR coord: 23039 – 27788        Rank L1 L2: 6 –
BC BR:        BC BR coord:
Initial region span: 1–64        Gap history (edge excluded):
–
Rec. model vs L2: –

## B.1.1.529 >>

| | num_seq | t_ch_MAX | max_CL | CL@BC_t_ch_MAX | aic | PV | PV_OK | t_ch_MAX_OK | phyl_OK |
|---|---|---|---|---|---|---|---|---|---|
| **B.1.1.529** | 248 | 64 | 64.425845 | NaN | NaN | NaN | * | * | * |
| **BA.4** | 6396 | 63 | 60.103804 | 56.267842 | 67.464316 | 5.489050e-03 | * | * | * |
| **BA.4.1** | 15800 | 63 | 54.742197 | 52.765910 | 74.468181 | 1.649283e-04 | * | * | * |
| **BA.5.1** | 33374 | 56 | 54.368027 | 17.453439 | 145.093122 | 7.627055e-20 | | | * |
| **BA.5.1.2** | 1602 | 56 | 54.359283 | -1.707270 | 183.414539 | 3.641452e-28 | | | * |
| **BA.5** | 3423 | 56 | 54.352774 | 37.075212 | 105.849576 | 2.530548e-11 | | | * |
| **BA.5.1.1** | 4192 | 54 | 52.236372 | 2.555708 | 174.888584 | 2.578512e-26 | | | * |
| **BA.4.1.9** | 175 | 63 | 51.635233 | 40.928585 | 98.142830 | 1.195143e-09 | | * | * |
| **BA.5.1.17** | 115 | 53 | 51.302564 | -13.736648 | 207.473296 | 2.171261e-33 | | | * |
| **BF.8** | 1870 | 56 | 51.280449 | -6.826645 | 193.653289 | 2.176140e-30 | | | * |

Cumulative Likelihood per-region

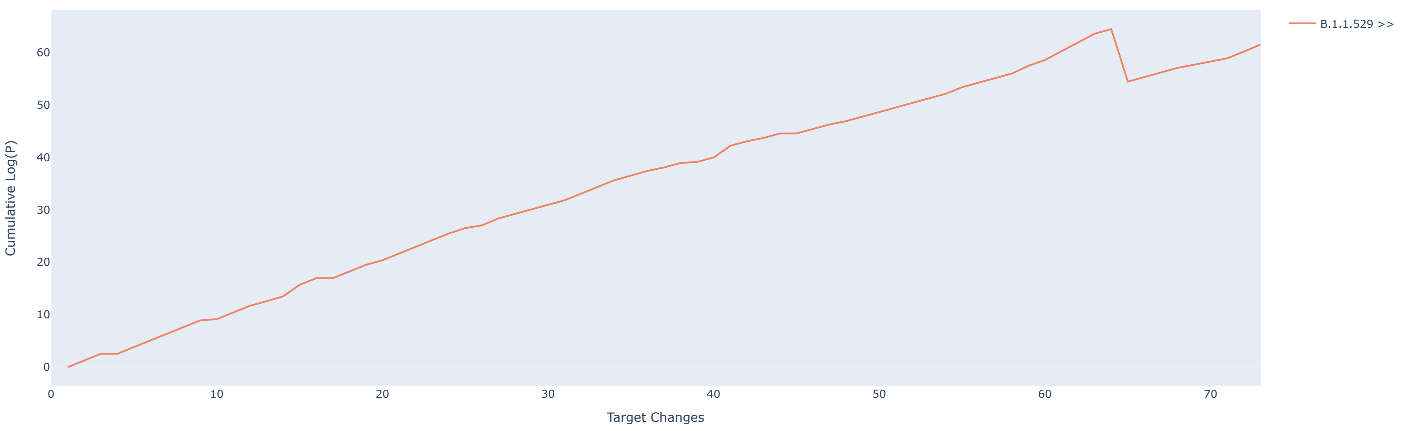


Cumulative Likelihood whole genome

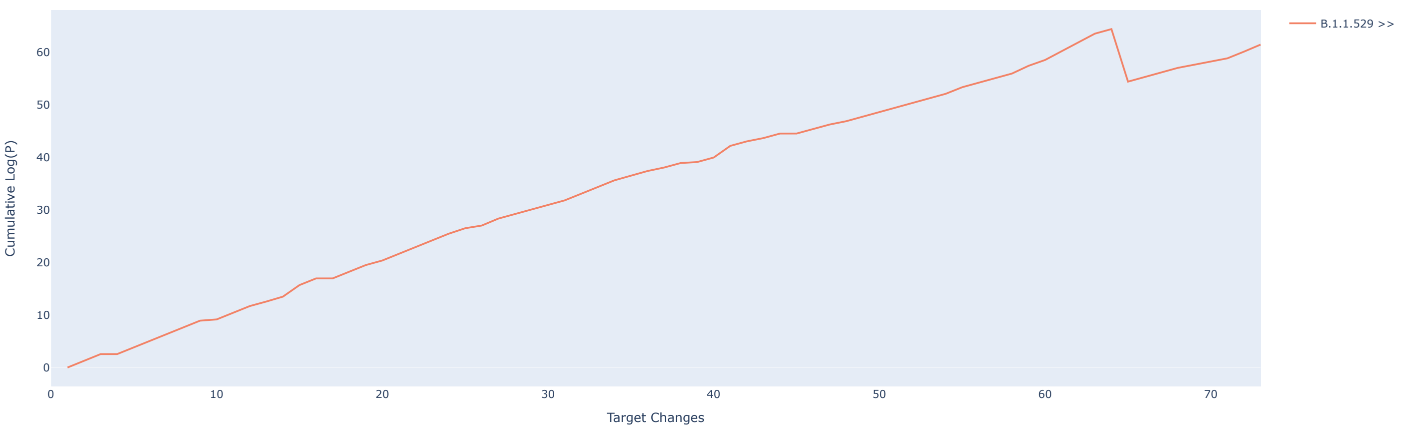


**Target sequence**

, 241_C|T, 670_T|G, 2790_C|T, 3037_C|T, 4184_G|A, 4321_C|T, 9344_C|T, 9424_A|G, 9534_C|T, 10029_C|T, 10198_C|T, 10447_G|A, 10449_C|A, 11288_11296, 12160_G|A, 12880_C|T, 14408_C|T, 15714_C|T, 17410_C|T, 18163_A|G, 19955_C|T, 20055_A|G, 21618_C|T, 21633_21641, 21765_21770, 21987_G|A, 22200_T|G, 22578_G|A, 22674_C|T, 22679_T|C, 22686_C|T, 22688_A|G, 22775_G|A, 22786_A|C, 22813_G|T, 22882_T|G, 22917_T|G, 22992_G|A, 22995_C|A, 23013_A|C, 23018_T|G, 23055_A|G, 23063_A|T, 23075_T|C, 23403_A|G, 23525_C|T, 23599_T|G, 23604_C|A, 23854_C|A, 23948_G|T, 24424_A|T, 24469_T|A, 25000_C|T, 25584_C|T, 26060_C|T, 26270_C|T, 26577_C|G, 26709_G|A, 26858_C|T, 27259_A|C, 27382_G|C, 27383_A|T, 27384_T|C, 27807_C|T, 27945_C|T, 28271_A|T, 28311_C|T, 28362_28370, 28881_G|A, 28882_G|A, 28883_G|C, 29510_A|C, 29734_29759

## Case 8 (1BP mid): XAT        test: KO

Target: (75%) 3 samples
GT: BA.2.3.13 + BA.1*
BC: BA.2.3
Direction L1: >>
Alt. candidates: [BA.2.3.9, BA.2.3.16, BA.2.3.5, BA.2.3.10, BA.2]
Model 1BP/2BP comparison:
Rec. model vs L1: -
Flags: Model_2BP_NotEnoughSpace_ForL2, Model_2BP_Bad_L2, SingleCandidateGenome

Number of changes: 66
GT BR: 55-57
GT BR coord: 26061 - 26529
Rank L1 L2: 11 -
BC BR:
BC BR coord:
Initial region span: 1-58
Gap history (edge excluded):
-
Rec. model vs L2: -

## BA.2.3 >>

| | num_seq | t_ch_MAX | max_CL | CL@BC_t_ch_MAX | aic | PV | PV_OK | t_ch_MAX_OK | phyl_OK |
|---|---|---|---|---|---|---|---|---|---|
| **BA.2.3** | 22517 | 58 | 62.540692 | NaN | NaN | NaN | * | * | * |
| **BA.2.3.9** | 127 | 58 | 62.421074 | 52.311910 | 59.376180 | 6.961120e-04 | * | * | * |
| **BA.2.3.16** | 36 | 58 | 61.975493 | 53.543667 | 56.912665 | 2.393497e-03 | * | * | * |
| **BA.2.3.2** | 339 | 56 | 60.815256 | 52.964521 | 58.070958 | 1.340115e-03 | * | | * |
| **BA.2.3.5** | 139 | 58 | 56.427314 | 53.475714 | 57.048572 | 2.231681e-03 | * | * | * |
| **BA.2.3.7** | 88 | 58 | 55.305404 | 43.616585 | 76.766830 | 1.165436e-07 | | * | * |
| **BA.2.3.10** | 121 | 58 | 54.244671 | 51.278003 | 61.443994 | 2.485168e-04 | * | * | * |
| **BA.2.3.1** | 111 | 66 | 53.667663 | 50.949025 | 62.101950 | 1.786646e-04 | * | | * |
| **BA.2** | 359165 | 58 | 50.698306 | 49.135497 | 65.729006 | 2.909338e-05 | * | * | * |
| **BA.2.3.12** | 62 | 58 | 50.513351 | 47.702307 | 68.595387 | 6.927580e-06 | | * | * |

Cumulative Likelihood per-region

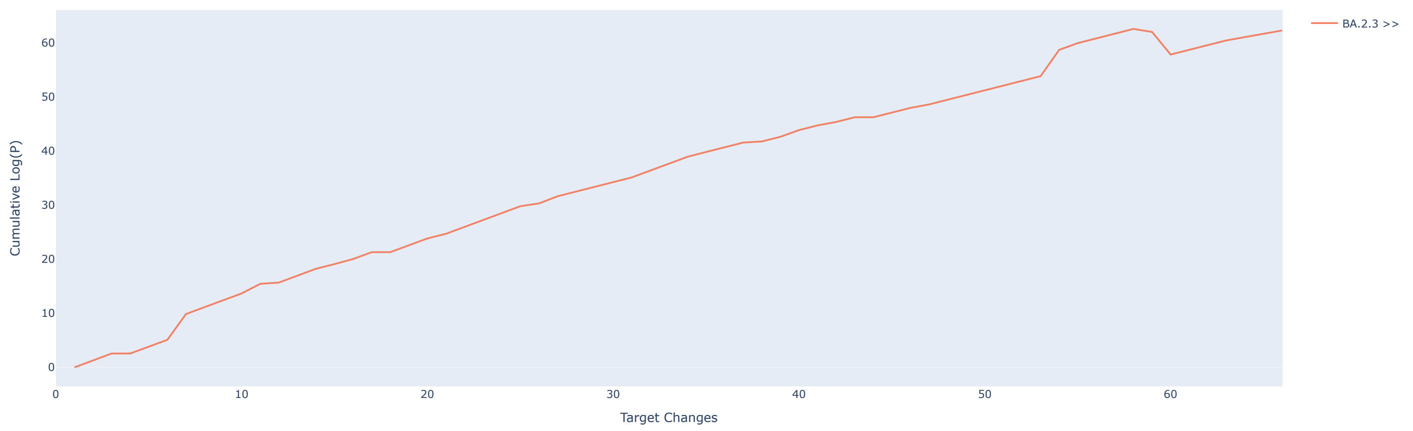


Cumulative Likelihood whole genome

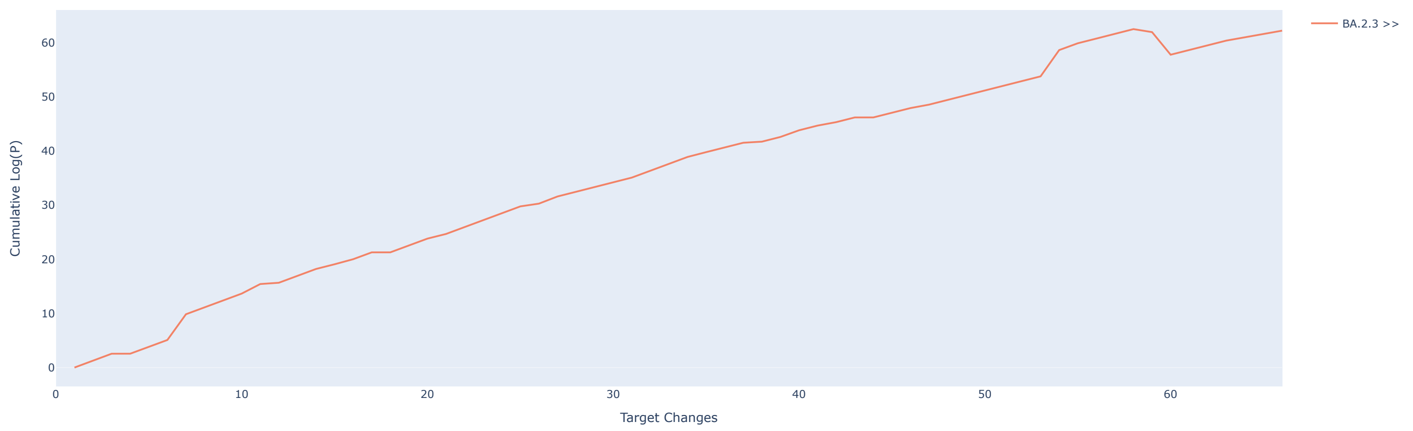


**Target sequence**

, 241_C|T, 670_T|G, 2790_C|T, 3037_C|T, 4184_G|A, 4321_C|T, 8991_C|T, 9344_C|T, 9424_A|G, 9534_C|T, 9866_C|T, 10029_C|T, 10198_C|T, 10447_G|A, 10449_C|A, 11288_11296, 12880_C|T, 14408_C|T, 15714_C|T, 17410_C|T, 18163_A|G, 19955_C|T, 20055_A|G, 21618_C|T, 21633_21641, 21987_G|A, 22200_T|G, 22578_G|A, 22674_C|T, 22679_T|C, 22686_C|T, 22688_A|G, 22775_G|A, 22786_A|C, 22813_G|T, 22882_T|G, 22992_G|A, 22995_C|A, 23013_A|C, 23040_A|G, 23055_A|G, 23063_A|T, 23075_T|C, 23403_A|G, 23525_C|T, 23599_T|G, 23604_C|A, 23854_C|A, 23948_G|T, 24424_A|T, 24469_T|A, 25000_C|T, 25584_C|T, 25810_C|T, 26060_C|T, 26270_C|T, 26577_C|G, 26709_G|A, 27259_A|C, 27807_C|T, 28271_A|T, 28311_C|T, 28362_28370, 28881_G|A, 28882_G|A, 28883_G|C

## Case 9 (1BP mid): XAV          test: KO

Target: (75%) 19 samples
Number of changes: 72
GT: BA.2* + BA.5*
GT BR: 19–22
GT BR coord: 15959 – 17279
Rank L1 L2: 11 –
BC: BA.5.1.24
BC BR:
BC BR coord:
Direction L1: >>
Initial region span: 1–71
Gap history (edge excluded):
Alt. candidates: [BA.5.1]
Model 1BP/2BP comparison: –
Rec. model vs L1: –
Rec. model vs L2: –
Flags: NotEnoughSpaceAfterL1, SingleCandidateGenome

## BA.5.1.24 >>

| | num_seq | t_ch_MAX | max_CL | CL@BC_t_ch_MAX | aic | PV | PV_OK | t_ch_MAX_OK | phyl_OK |
|---|---|---|---|---|---|---|---|---|---|
| **BA.5.1.24** | 1230 | 71 | 64.444202 | NaN | NaN | NaN | * | * | * |
| **BA.5.1** | 33374 | 71 | 55.398262 | 52.586658 | 70.826685 | 1.209661e-04 | * | * | * |
| **BA.5.1.3** | 1626 | 71 | 47.010083 | 44.148893 | 87.702214 | 2.626575e-08 | | * | |
| **BA.5** | 3423 | 72 | 46.309183 | 46.309183 | 83.381635 | 2.277539e-07 | | * | * |
| **BA.5.2.1** | 58578 | 62 | 43.349192 | 38.557348 | 98.885305 | 9.761402e-11 | | | |
| **BA.5.5** | 22209 | 62 | 41.693047 | 41.449527 | 93.100946 | 1.765203e-09 | | | |
| **BA.5.3** | 440 | 62 | 36.496006 | 31.945456 | 112.109089 | 1.314697e-13 | | | |
| **BA.5.1.22** | 3125 | 71 | 35.632468 | 32.837387 | 110.325227 | 3.201458e-13 | | * | |
| **BA.5.1.1** | 4192 | 71 | 35.358405 | 27.665992 | 120.668016 | 1.819891e-15 | | * | |
| **BF.7** | 4385 | 62 | 34.889980 | 14.117624 | 147.764753 | 2.385209e-21 | | | |

Cumulative Likelihood per-region

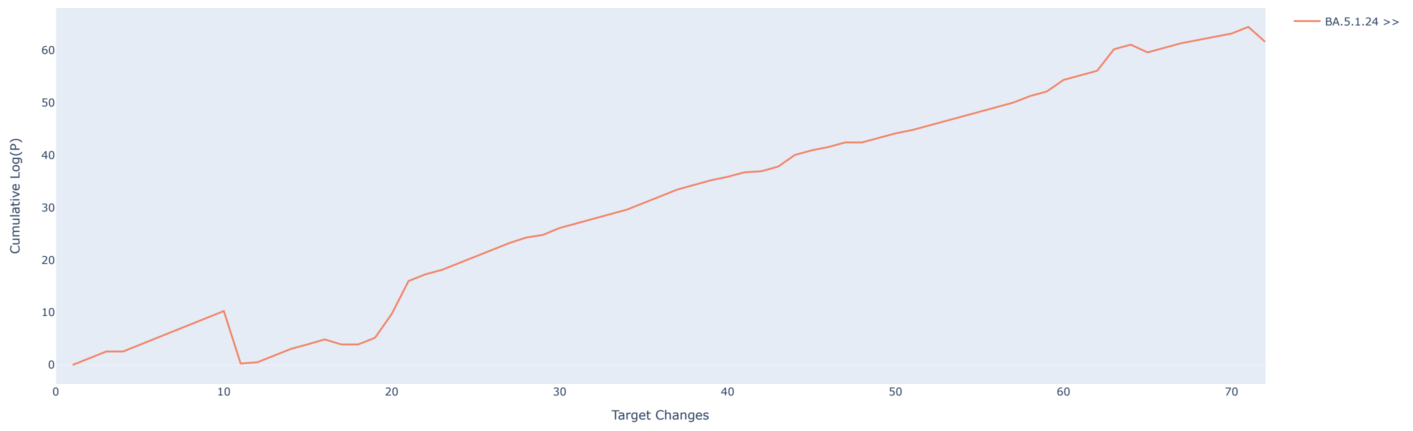


Cumulative Likelihood whole genome

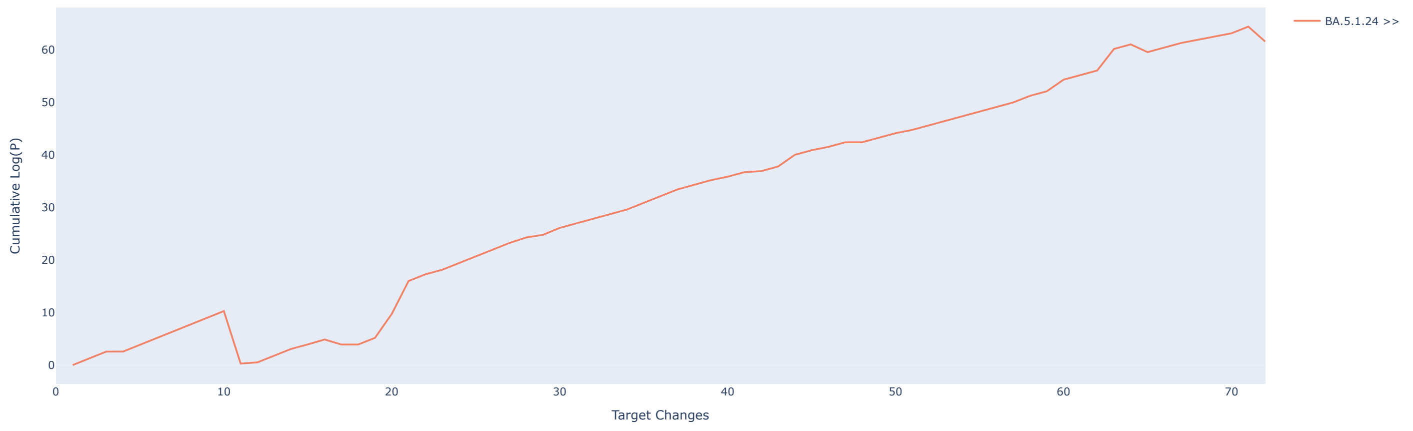


**Target sequence**

, 241_C|T, 670_T|G, 2790_C|T, 3037_C|T, 4184_G|A, 4321_C|T, 6606_C|T, 9344_C|T, 9424_A|G, 9534_C|T, 9866_C|T, 10029_C|T, 10198_C|T, 10447_G|A, 10449_C|A, 11288_11296, 12880_C|T, 14408_C|T, 15714_C|T, 15960_C|T, 17278_G|T, 17410_C|T, 18163_A|G, 19955_C|T, 20055_A|G, 21618_C|T, 21633_21641, 21765_21770, 21987_G|A, 22200_T|G, 22578_G|A, 22674_C|T, 22679_T|C, 22686_C|T, 22688_A|G, 22775_G|A, 22786_A|C, 22813_G|T, 22882_T|G, 22917_T|G, 22992_G|A, 22995_C|A, 23013_A|C, 23018_T|G, 23055_A|G, 23063_A|T, 23075_T|C, 23403_A|G, 23525_C|T, 23599_T|G, 23604_C|A, 23854_C|A, 23948_G|T, 24424_A|T, 24469_T|A, 25000_C|T, 25584_C|T, 26060_C|T, 26270_C|T, 26529_G|A, 26577_C|G, 26709_G|A, 27438_T|C, 27807_C|T, 28271_A|T, 28311_C|T, 28362_28370, 28881_G|A, 28882_G|A, 28883_G|C, 29510_A|C, 29734_29759

## Case 10 (1BP mid): XB test: KO

Target: (75%) 1247 samples
Number of changes: 33
GT: B.1.634 + B.1.631
GT BR: 13–18
GT BR coord: 20999 – 22501
Rank L1 L2: 11 –
BC: BN.1.9
BC BR:
BC BR coord:
Direction L1: >>
Initial region span: 1–3
Gap history (edge excluded):
Alt. candidates: [B.1]
Model 1BP/2BP comparison: –
Rec. model vs L1: –
Rec. model vs L2: –
Flags: Model_1BP_NoL2, SingleCandidateGenome

## BN.1.9 >>

| | num_seq | t_ch_MAX | max_CL | CL@BC_t_ch_MAX | aic | PV | PV_OK | t_ch_MAX_OK | phyl_OK |
|---|---|---|---|---|---|---|---|---|---|
| **BN.1.9** | 70 | 3 | 1.710556 | NaN | NaN | NaN | * | * | * |
| **B.1.239** | 228 | 3 | 1.475507 | -236.321380 | 818.642759 | 1.327587e+78 | * | * | |
| **B.1.1** | 16129 | 5 | 1.191402 | -126.168585 | 598.337170 | 9.132718e+125 | * | | * |
| **R.1** | 1553 | 4 | 1.044120 | -282.046143 | 910.092286 | 1.840550e+58 | * | * | |
| **AY.3.2** | 798 | 3 | 0.898292 | -240.242480 | 826.484961 | 2.634077e+76 | * | * | |
| **AY.29** | 1010 | 3 | 0.860154 | -260.476837 | 866.953675 | 4.292197e+67 | * | * | |
| **B.1** | 29438 | 4 | 0.273979 | -91.740141 | 529.480283 | 8.191418e+140 | * | * | * |
| **BA.5.11** | 250 | 3 | 0.113330 | -286.390299 | 918.780599 | 2.387471e+56 | * | * | |
| **AY.129** | 5456 | 3 | 0.086744 | -216.053439 | 778.106879 | 8.395384e+86 | * | * | |

Cumulative Likelihood per-region

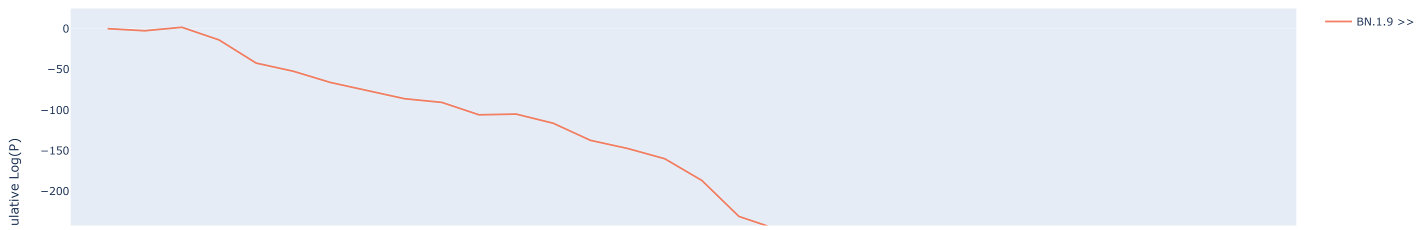

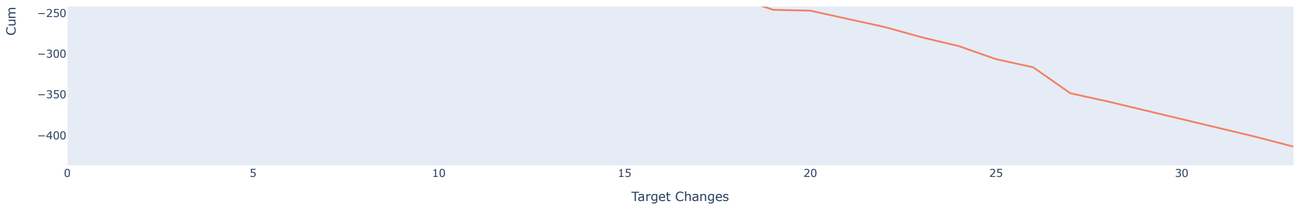


Cumulative Likelihood whole genome

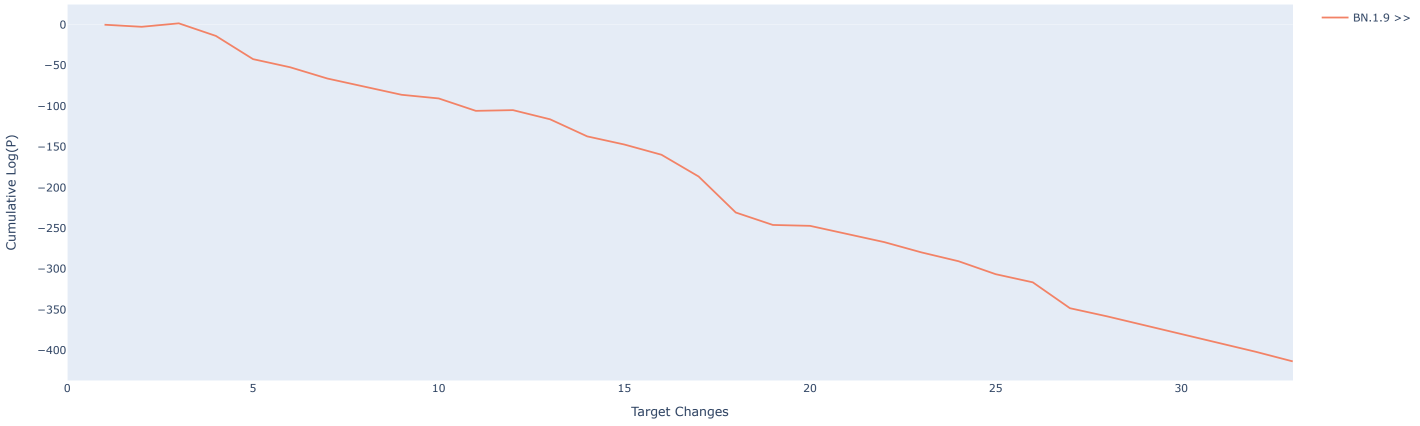


**Target sequence**

, 241_C|T, 3037_C|T, 3688_C|T, 3884_C|T, 6633_C|T, 7142_A|G, 9614_A|G, 9693_C|T, 9754_A|C, 11288_11296, 14408_C|T, 15451_G|A, 16466_C|T, 21057_C|T, 21615_T|G, 21846_C|T, 22036_A|C, 22882_T|A, 23403_A|G, 23604_C|A, 23625_C|T, 23765_T|G, 24442_C|T, 24642_C|T, 26158_26161, 26222_C|T, 27389_C|T, 27769_C|T, 28048_G|A, 28271_28271, 28330_A|G, 28854_C|T, 28910_A|T

## Case 11 (1BP mid): XBB test: KO

Target: (75%) 162 samples
Number of changes: 92
GT: BJ.1 + BM.1.1.1
GT BR: 50–54
GT BR coord: 22891 – 22935
Rank L1 L2: 11 1
BC: BA.2.9 + BM.1.1.1
BC BR: 38–39
BC BR coord: 22331 – 22332
Direction L1: <<
Initial region span: 1–19,53–92
Gap history (edge excluded): 19–53
Alt. candidates: [BA.2], [CJ.1.1, CJ.1.2]
Model 1BP/2BP comparison: –
Rec. model vs L1: 1.25e–66
Rec. model vs L2: 8.02e–109
Flags: Model_2BP_Bad_L1_opp

## BA.2.9 >>

| | num_seq | t_ch_MAX | max_CL | CL@BC_t_ch_MAX | aic | PV | PV_OK | t_ch_MAX_OK | phyl_OK |
|---|---|---|---|---|---|---|---|---|---|
| **BA.2.9** | 61803 | 19 | 9.036559 | NaN | NaN | NaN | * | * | * |
| **BA.2.56** | 585 | 19 | 8.644369 | -93.672104 | 295.344209 | 2.913797e-23 | | * | |
| **BA.2** | 359165 | 19 | 8.433608 | -46.693790 | 201.387580 | 7.335718e-03 | * | * | * |
| **BG.2** | 5326 | 17 | 8.007406 | -102.175876 | 312.351752 | 5.899087e-27 | | | |
| **BA.2.78** | 23 | 19 | 4.796647 | -97.524760 | 303.049520 | 6.169559e-25 | | * | |
| **BA.2.12.1** | 94944 | 17 | 4.686453 | -64.350850 | 236.701700 | 1.577515e-10 | | | |
| **BA.5.1** | 33374 | 11 | 4.349979 | -46.628807 | 201.257615 | 7.828378e-03 | * | | |
| **BA.5.1.27** | 1123 | 11 | 1.043770 | -109.212542 | 326.425083 | 5.168346e-30 | | | |
| **CD.1** | 31 | 11 | 0.834118 | -125.494522 | 358.989045 | 4.395795e-37 | | | |

## BM.1.1.1 <<

| | num_seq | t_ch_MAX | max_CL | CL@BC_t_ch_MAX | aic | PV | PV_OK | t_ch_MAX_OK | phyl_OK |
|---|---|---|---|---|---|---|---|---|---|
| **BM.1.1.1** | 15 | 53 | 52.536144 | NaN | NaN | NaN | * | * | * |
| **CJ.1.1** | 15 | 52 | 52.489942 | 48.842077 | 22.315846 | 2.247143e+04 | * | * | * |
| **CJ.1.2** | 43 | 53 | 48.524504 | 34.876639 | 50.246722 | 1.935122e-02 | * | * | * |
| **XBF.3** | 37 | 53 | 48.520635 | 34.872770 | 50.254459 | 1.935122e-02 | * | * | |
| **XBF.5** | 14 | 53 | 48.473926 | 34.826061 | 50.347877 | 1.840745e-02 | * | * | |
| **XBF.6** | 10 | 53 | 48.442674 | 34.794809 | 50.410382 | 1.786342e-02 | * | * | |
| **DS.1** | 11 | 53 | 46.656894 | 16.924364 | 86.151273 | 3.098292e-10 | | * | |
| **BN.1.5** | 730 | 53 | 46.286330 | 26.120907 | 67.758185 | 3.051126e-06 | | * | |
| **BM.1.1.3** | 259 | 53 | 43.840502 | 39.904005 | 40.191990 | 2.959440e+00 | * | * | |
| **XBF.7** | 10 | 53 | 42.132038 | 28.484173 | 63.031654 | 3.247630e-05 | * | * | |

Cumulative Likelihood per-region

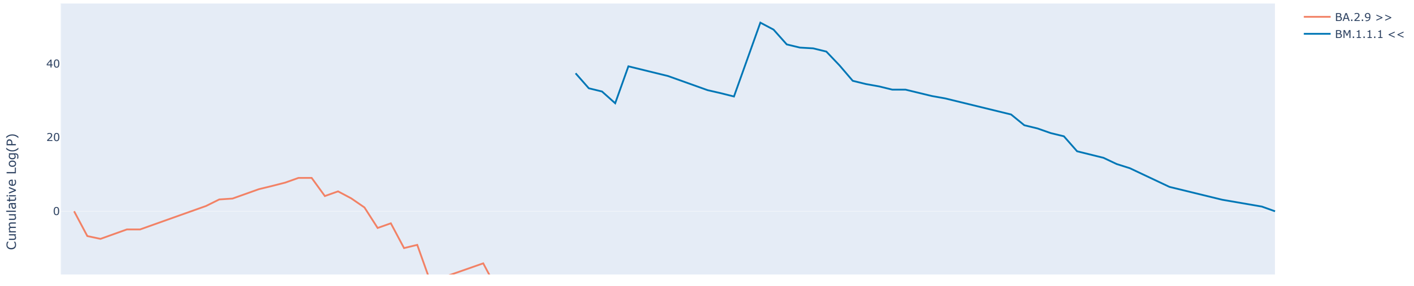

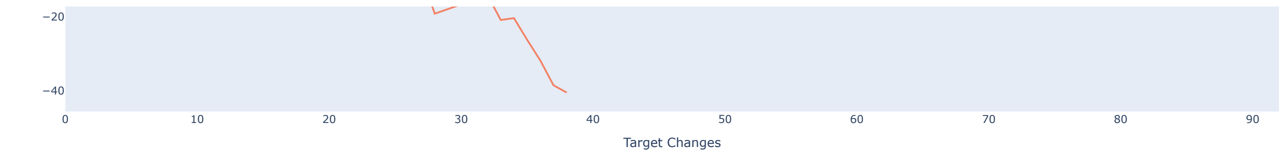


Cumulative Likelihood whole genome

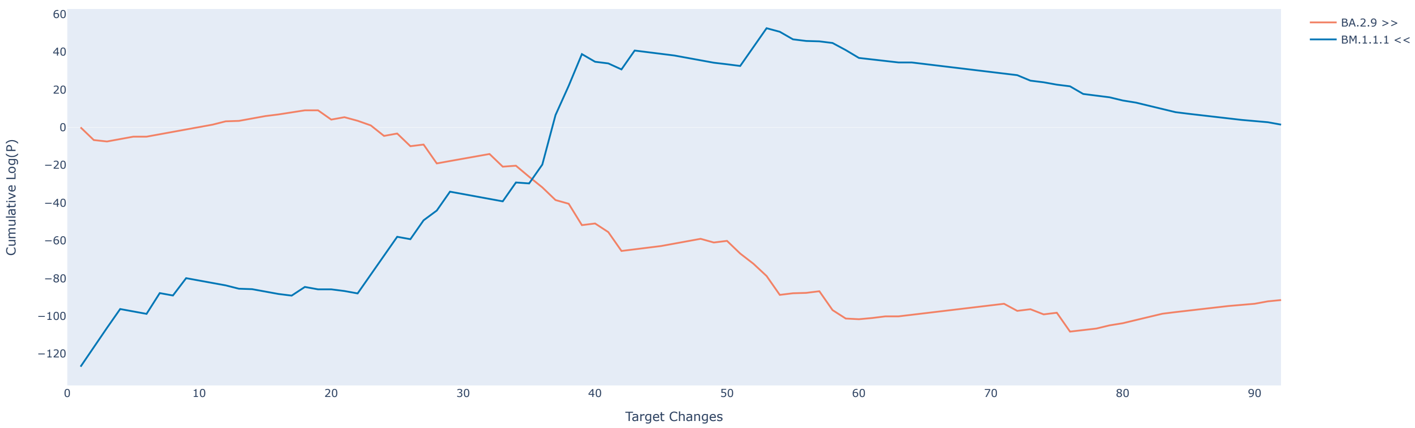


**Target sequence**

, 241_C|T, 405_A|G, 510_G|A, 670_T|G, 2790_C|T, 3037_C|T, 4184_G|A, 4321_C|T, 9344_C|T, 9424_A|G, 9534_C|T, 9866_C|T, 10029_C|T, 10198_C|T, 10447_G|A, 10449_C|A, 11288_11296, 12880_C|T, 14408_C|T, 15451_G|A, 15714_C|T, 15738_C|T, 15939_T|C, 16342_T|C, 17410_C|T, 17859_T|C, 18163_A|G, 19326_A|G, 19955_C|T, 20055_A|G, 21618_C|T, 21633_21641, 21810_T|C, 21987_G|A, 21992_21994, 22000_C|A, 22109_C|G, 22200_T|A, 22577_G|C, 22578_G|A, 22599_G|C, 22664_C|A, 22674_C|T, 22679_T|C, 22686_C|T, 22688_A|G, 22775_G|A, 22786_A|C, 22813_G|T, 22882_T|G, 22895_G|C, 22896_T|C, 22898_G|A, 22942_T|G, 22992_G|A, 22995_C|A, 23013_A|C, 23019_T|C, 23031_T|C, 23055_A|G, 23063_A|T, 23075_T|C, 23403_A|G, 23525_C|T, 23599_T|G, 23604_C|A, 23854_C|A, 23948_G|T, 24424_A|T, 24469_T|A, 25000_C|T, 25416_C|T, 25584_C|T, 26060_C|T, 26270_C|T, 26275_A|G, 26577_C|G, 26709_G|A, 26858_C|T, 27259_A|C, 27382_G|C, 27383_A|T, 27384_T|C, 27807_C|T, 28271_A|T, 28311_C|T, 28362_28370, 28881_G|A, 28882_G|A, 28883_G|C, 29510_A|C, 29734_29759

## Case 12 (1BP mid): XBD    test: OK

Target: (75%) 66 samples    Number of changes: 86
GT: BA.2.75.2 + BA.5.2.1    GT BR: 53-67    GT BR coord: 23012 - 24616    Rank L1 L2: 1 11
BC: BA.2.75.2 + BF.3    BC BR: 66-67    BC BR coord: 24494 - 24495
Direction L1: >>    Initial region span: 1-66,67-86    Gap history (edge excluded):
Alt. candidates: [CA.3], []
Model 1BP/2BP comparison:    1BP vs 2BP: 1.19e-04
Rec. model vs L1: 2.05e-85    Rec. model vs L2: 5.26e-203
Flags: Model_1BP_Best

### BA.2.75.2 >>

| | num_seq | t_ch_MAX | max_CL | CL@BC_t_ch_MAX | aic | PV | PV_OK | t_ch_MAX_OK | phyl_OK |
|---|---|---|---|---|---|---|---|---|---|
| **BA.2.75.2** | 582 | 66 | 124.732494 | NaN | NaN | NaN | * | * | * |
| **CA.3** | 34 | 66 | 124.659639 | 124.659639 | -83.319279 | 9.323938e-01 | * | * | * |
| **BY.1** | 153 | 66 | 108.393790 | 108.393790 | -50.787580 | 8.050070e-08 | | * | |
| **CA.3.1** | 148 | 66 | 106.608864 | 106.608864 | -47.217729 | 1.350778e-08 | | * | * |
| **CA.1** | 134 | 58 | 103.469312 | 101.224475 | -36.448950 | 6.193102e-11 | | | * |
| **BM.1.1** | 104 | 66 | 102.378550 | 102.378550 | -38.757099 | 1.965705e-10 | | * | |
| **CA.5** | 64 | 66 | 102.060259 | 102.060259 | -38.120518 | 1.427395e-10 | | * | * |
| **CA.7** | 115 | 66 | 96.718741 | 96.718741 | -27.437483 | 6.845601e-13 | | * | * |
| **CH.1.1** | 2870 | 71 | 96.139034 | 95.406956 | -24.813911 | 1.837868e-13 | | | |
| **BL.1** | 158 | 54 | 95.997680 | 85.299100 | -4.598200 | 7.512225e-18 | | | |

### BF.3 <<

| | num_seq | t_ch_MAX | max_CL | CL@BC_t_ch_MAX | aic | PV | PV_OK | t_ch_MAX_OK | phyl_OK |
|---|---|---|---|---|---|---|---|---|---|
| **BF.3** | 55 | 67 | 30.946163 | NaN | NaN | NaN | * | * | * |
| **BF.23** | 32 | 67 | 30.697081 | 30.697081 | -21.394161 | 7.788008e-01 | * | * | |
| **BF.24** | 52 | 67 | 29.905479 | 29.905479 | -19.810957 | 3.534547e-01 | * | * | |
| **BF.25** | 295 | 68 | 25.209185 | 15.209185 | 9.581631 | 1.466818e-07 | | * | |
| **BF.16** | 260 | 68 | 25.192166 | 15.192166 | 9.615669 | 1.437773e-07 | | * | |
| **BF.32** | 224 | 68 | 25.164066 | 15.164066 | 9.671868 | 1.402275e-07 | | * | * |
| **BF.1.1** | 158 | 68 | 25.153194 | 15.153194 | 9.693613 | 1.388322e-07 | | * | |
| **BF.4** | 881 | 68 | 25.135641 | 15.135641 | 9.728717 | 1.360831e-07 | | * | |
| **BF.14** | 602 | 68 | 25.127418 | 15.127418 | 9.745164 | 1.347291e-07 | | * | |
| **BF.40** | 51 | 68 | 25.110389 | 15.110389 | 9.779222 | 1.327232e-07 | | * | |

Cumulative Likelihood per-region

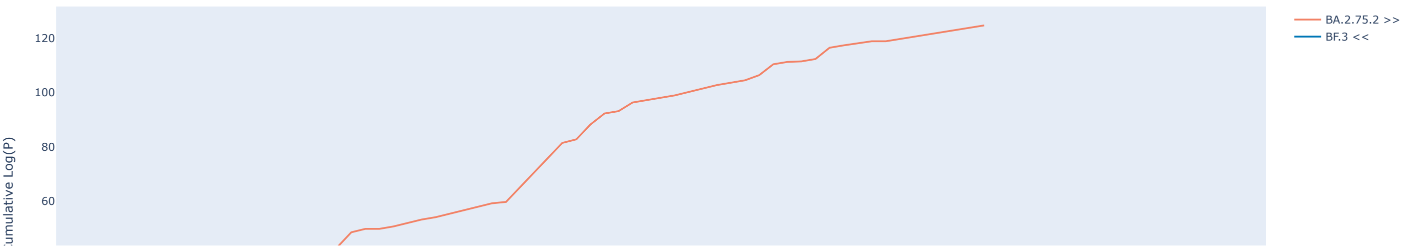

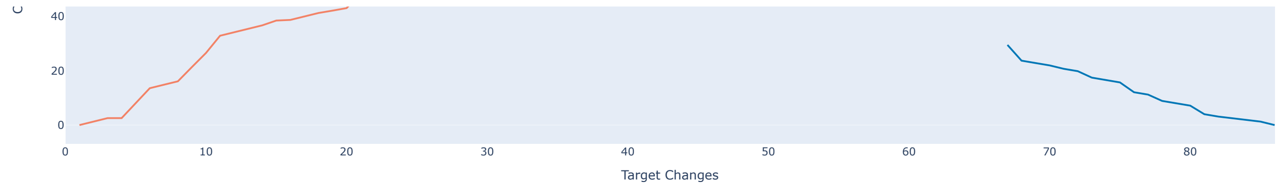


Cumulative Likelihood whole genome

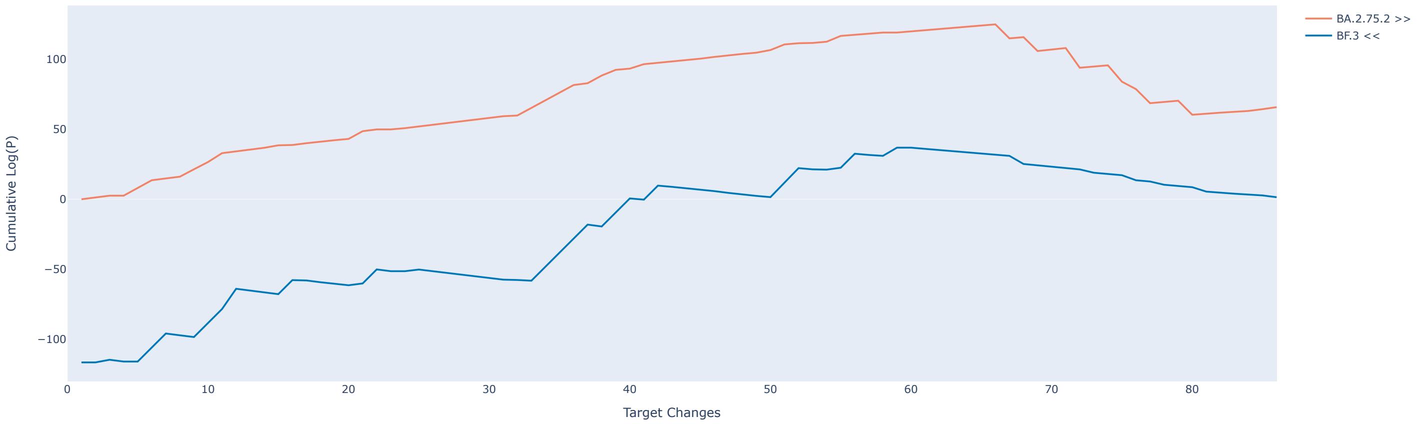


**Target sequence**

, 241_C|T, 670_T|G, 2790_C|T, 3037_C|T, 3796_C|T, 3927_C|T, 4184_G|A, 4321_C|T, 4586_C|T, 5183_C|T, 5192_C|T, 9344_C|T, 9424_A|G, 9534_C|T, 9866_C|T, 10029_C|T, 10198_C|T, 10447_G|A, 10449_C|A, 11288_11296, 12444_A|G, 12880_C|T, 14408_C|T, 15451_G|A, 15714_C|T, 17410_C|T, 18163_A|G, 19955_C|T, 20055_A|G, 21618_C|T, 21633_21641, 21987_G|A, 22001_A|G, 22016_T|C, 22033_C|A, 22190_A|G, 22200_T|G, 22331_G|A, 22577_G|C, 22578_G|A, 22599_G|C, 22674_C|T, 22679_T|C, 22686_C|T, 22688_A|G, 22775_G|A, 22786_A|C, 22813_G|T, 22882_T|G, 22898_G|A, 22942_T|G, 22992_G|A, 22995_C|A, 23013_A|C, 23019_T|C, 23055_A|G, 23063_A|T, 23075_T|C, 23403_A|G, 23525_C|T, 23599_T|G, 23604_C|A, 23854_C|A, 23948_G|T, 24424_A|T, 24469_T|A, 24620_G|T, 25000_C|T, 25584_C|T, 26060_C|T, 26270_C|T, 26529_G|A, 26577_C|G, 26709_G|A, 27038_A|G, 27807_C|T, 27889_C|T, 28271_A|T, 28311_C|T, 28330_A|G, 28362_28370, 28881_G|A, 28882_G|A, 28883_G|C, 29510_A|C, 29734_29759

## Case 13 (1BP mid): XBE test: OK

Target: (75%) 134 samples
GT: BA.5.2* + BE.4.1
BC: BA.5.2.6 + BE.4.1.1
Direction L1: >>
Alt. candidates: [BA.5.2], [BE.4, CQ.2, BE.4.1, CQ.1.1]
Model 1BP/2BP comparison:
Rec. model vs L1: 2.32e-38
Flags: Model_1BP_Best

Number of changes: 75
GT BR: 31–53
BC BR: 63–64
Initial region span: 1–63,64–75
1BP vs 2BP: 6.70e-03
Rec. model vs L2: 1.93e-81

GT BR coord: 22591 – 23609 Rank L1 L2: 4 4
BC BR coord: 26719 – 26720
Gap history (edge excluded):

### BA.5.2.6 >>

| | num_seq | t_ch_MAX | max_CL | CL@BC_t_ch_MAX | aic | PV | PV_OK | t_ch_MAX_OK | phyl_OK |
|---|---|---|---|---|---|---|---|---|---|
| **BA.5.2.6** | 2139 | 63 | 73.358317 | NaN | NaN | NaN | * | * | * |
| **CP.1** | 243 | 60 | 67.152654 | 62.829710 | 16.340581 | 2.672262e-05 | * | | * |
| **CR.1** | 77 | 63 | 66.491494 | 66.491494 | 9.017011 | 1.038477e-03 | * | * | |
| **BA.5.2** | 32711 | 63 | 65.676454 | 65.676454 | 10.647092 | 4.596708e-04 | * | * | * |
| **BA.5.2.20** | 3367 | 63 | 64.296521 | 64.296521 | 13.406958 | 1.156433e-04 | * | * | |
| **BA.5.2.28** | 617 | 63 | 62.216234 | 62.216234 | 17.567532 | 1.444734e-05 | * | * | |
| **BA.5.2.33** | 216 | 63 | 60.172621 | 60.172621 | 21.654759 | 1.878569e-06 | | * | |
| **BA.5.2.12** | 65 | 63 | 60.041795 | 60.041795 | 21.916410 | 1.641336e-06 | | * | |
| **BA.5.2.34** | 1147 | 63 | 59.618284 | 59.618284 | 22.763433 | 1.078435e-06 | | * | |
| **BA.5.2.2** | 368 | 63 | 59.216569 | 59.216569 | 23.566862 | 7.192909e-07 | | * | |

### BE.4.1.1 <<

| | num_seq | t_ch_MAX | max_CL | CL@BC_t_ch_MAX | aic | PV | PV_OK | t_ch_MAX_OK | phyl_OK |
|---|---|---|---|---|---|---|---|---|---|
| **BE.4.1.1** | 30 | 64 | 21.600967 | NaN | NaN | NaN | * | * | * |
| **BE.4** | 332 | 64 | 21.589362 | 21.589362 | -15.178724 | 9.900498e-01 | * | * | * |
| **CQ.2** | 380 | 64 | 21.572806 | 21.572806 | -15.145612 | 9.753099e-01 | * | * | * |
| **BE.4.1** | 83 | 64 | 21.480210 | 21.480210 | -14.960421 | 8.869204e-01 | * | * | * |
| **CQ.1.1** | 81 | 64 | 15.405719 | 15.405719 | -2.811438 | 2.039603e-03 | * | * | * |
| **BE.1.4** | 775 | 64 | 14.951205 | 14.951205 | -1.902411 | 1.294022e-03 | * | * | |
| **BE.1.4.4** | 25 | 65 | 14.024707 | 4.024707 | 19.950587 | 2.329563e-08 | | * | |
| **BE.10** | 131 | 65 | 14.001540 | -1.929708 | 31.859417 | 6.040193e-11 | | * | |
| **BE.1.2** | 393 | 65 | 13.959847 | 3.959847 | 20.080307 | 2.182958e-08 | | * | |
| **BE.2** | 670 | 65 | 13.926631 | 3.926631 | 20.146738 | 2.107876e-08 | | * | |

Cumulative Likelihood per-region

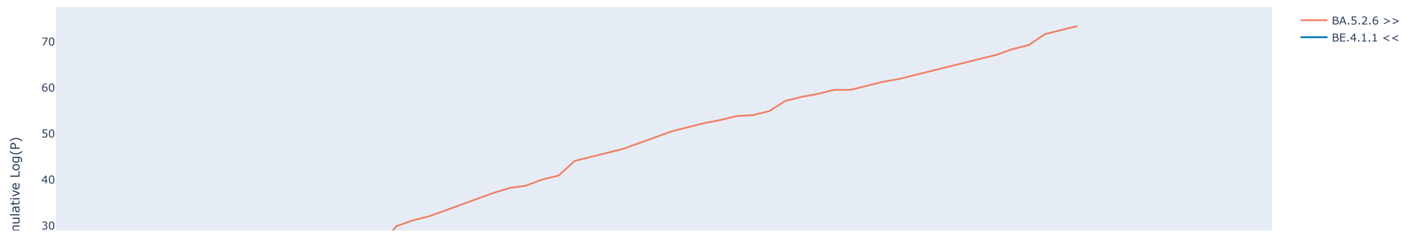

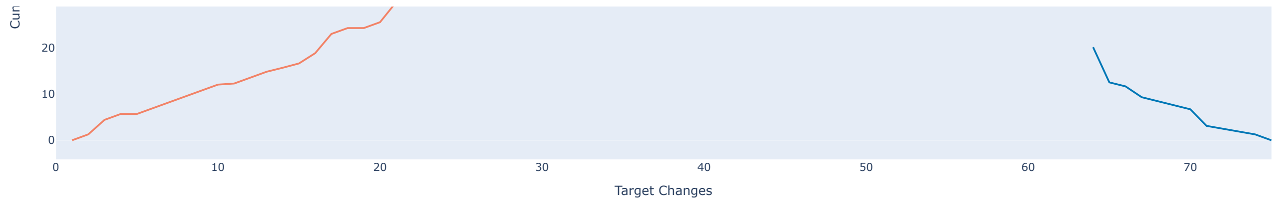


Cumulative Likelihood whole genome

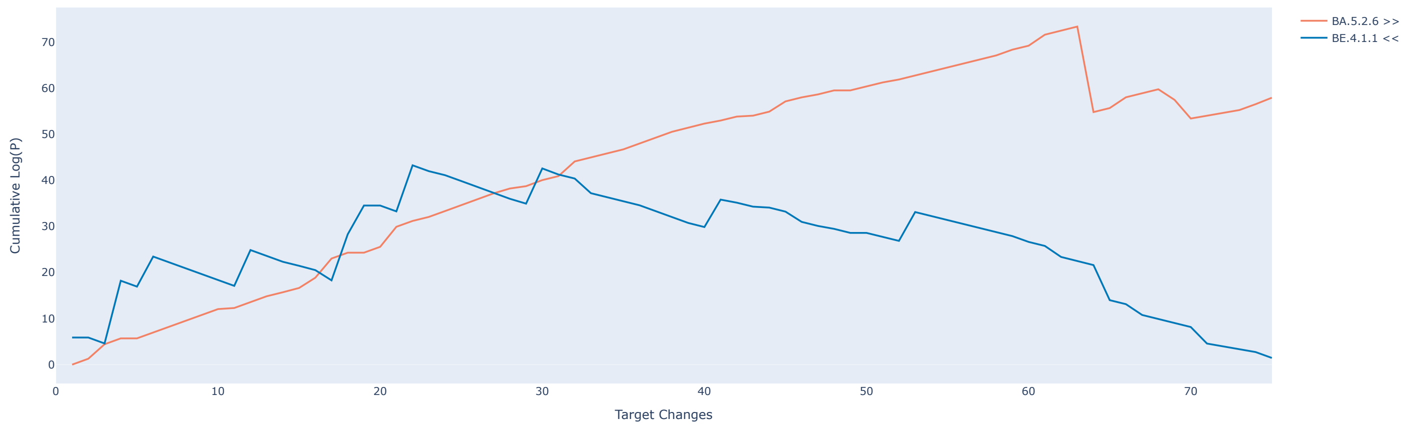


**Target sequence**

, 241_C|T, 670_T|G, 1627_C|T, 2790_C|T, 3037_C|T, 4184_G|A, 4321_C|T, 9344_C|T, 9424_A|G, 9534_C|T, 10029_C|T, 10198_C|T, 10447_G|A, 10449_C|A, 11288_11296, 12160_G|A, 12310_G|A, 12880_C|T, 14408_C|T, 15714_C|T, 16616_C|A, 17410_C|T, 18163_A|G, 19955_C|T, 20055_A|G, 21618_C|T, 21633_21641, 21765_21770, 21987_G|A, 22200_T|G, 22578_G|A, 22599_G|C, 22674_C|T, 22679_T|C, 22686_C|T, 22688_A|G, 22775_G|A, 22786_A|C, 22813_G|T, 22882_T|G, 22917_T|G, 22992_G|A, 22995_C|A, 23013_A|C, 23018_T|G, 23055_A|G, 23063_A|T, 23075_T|C, 23403_A|G, 23525_C|T, 23599_T|G, 23604_C|A, 23854_C|A, 23948_G|T, 24424_A|T, 24469_T|A, 25000_C|T, 25584_C|T, 26060_C|T, 26270_C|T, 26529_G|A, 26577_C|G, 26709_G|A, 27800_C|T, 27807_C|T, 27889_C|T, 28271_A|T, 28311_C|T, 28362_28370, 28681_G|T, 28881_G|A, 28882_G|A, 28883_G|C, 29510_A|C, 29734_29759

## Case 14 (1BP mid): XBF test: OK

Target: (75%) 298 samples Number of changes: 91
GT: BA.5.2 + CJ.1 GT BR: 14–16 GT BR coord: 9864 – 9867 Rank L1 L2: 10 11
BC: BA.5.2.3 + CJ.1.1 BC BR: 11–12 BC BR coord: 8703 – 8704
Direction L1: << Initial region span: 1–4,12–91 Gap history (edge excluded): 4–12
Alt. candidates: [], []
Model 1BP/2BP comparison: –
Rec. model vs L1: 1.15e–266 Rec. model vs L2: 1.27e–56
Flags: Model_2BP_Bad_L1_opp

## BA.5.2.3 >>

| | num_seq | t_ch_MAX | max_CL | CL@BC_t_ch_MAX | aic | PV | PV_OK | t_ch_MAX_OK | phyl_OK |
|---|---|---|---|---|---|---|---|---|---|
| **BA.5.2.3** | 1656 | 4 | 10.896722 | NaN | NaN | NaN | * | * | * |
| **BZ.2** | 95 | 4 | 10.886449 | -30.699861 | 93.399723 | 1.183251e-09 | | * | * |
| **BF.3.1** | 271 | 6 | 6.580749 | -11.703811 | 55.407622 | 2.101361e-01 | * | | |
| **BU.1** | 270 | 6 | 6.569534 | -20.893708 | 73.787417 | 2.144541e-05 | * | | |
| **BA.5.2.13** | 710 | 6 | 5.614248 | -8.990481 | 49.980962 | 3.174023e+00 | * | | |
| **BQ.1.1.61** | 44 | 3 | 5.572200 | -38.007698 | 108.015396 | 7.913785e-13 | | * | |
| **BA.5.2.6** | 2139 | 6 | 4.511918 | -2.476735 | 36.953470 | 2.143081e+03 | * | | |
| **BF.10** | 7646 | 6 | 4.335376 | -8.542218 | 49.084436 | 4.977860e+00 | * | | |
| **BA.5.2.20** | 3367 | 6 | 4.018239 | -12.563127 | 57.126255 | 8.892162e-02 | * | | |
| **BA.5.2** | 32711 | 6 | 3.974897 | -4.398732 | 40.797465 | 3.126236e+02 | * | | * |

## CJ.1.1 <<

| | num_seq | t_ch_MAX | max_CL | CL@BC_t_ch_MAX | aic | PV | PV_OK | t_ch_MAX_OK | phyl_OK |
|---|---|---|---|---|---|---|---|---|---|
| **CJ.1.1** | 15 | 12 | 135.149664 | NaN | NaN | NaN | * | * | * |
| **CJ.1.2** | 43 | 12 | 135.053813 | 135.053813 | -98.107625 | 9.093729e-01 | * | * | |
| **BM.1.1.1** | 15 | 12 | 120.281631 | 120.281631 | -68.563261 | 3.483702e-07 | | * | * |
| **BM.1.1** | 104 | 12 | 116.161775 | 116.161775 | -60.323550 | 5.659105e-09 | | * | * |
| **BM.4.1.1** | 124 | 12 | 106.327528 | 106.327528 | -40.655055 | 3.045321e-13 | | * | |
| **BM.1.1.3** | 259 | 12 | 102.650590 | 102.650590 | -33.301180 | 7.681205e-15 | | * | |
| **BY.1** | 153 | 12 | 99.804800 | 99.804800 | -27.609600 | 4.465412e-16 | | * | |
| **CH.1.1.7** | 26 | 12 | 95.776866 | 95.776866 | -19.553731 | 7.936971e-18 | | * | |
| **CH.1.1** | 2870 | 12 | 95.574400 | 95.574400 | -19.148801 | 6.498242e-18 | | * | |
| **BA.2.75.2** | 582 | 12 | 93.241277 | 93.241277 | -14.482554 | 6.290979e-19 | | * | |

Cumulative Likelihood per-region

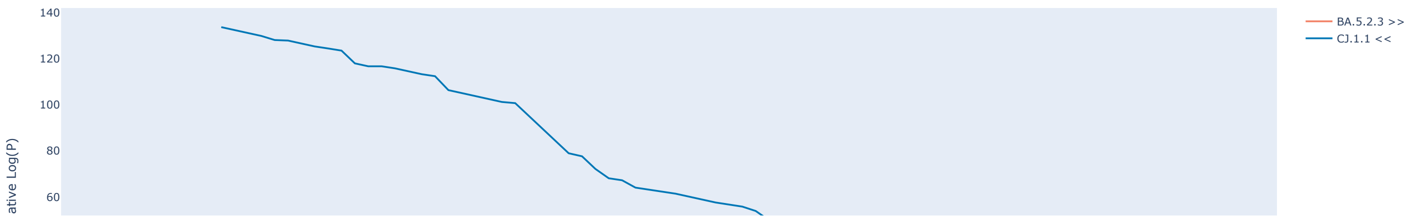

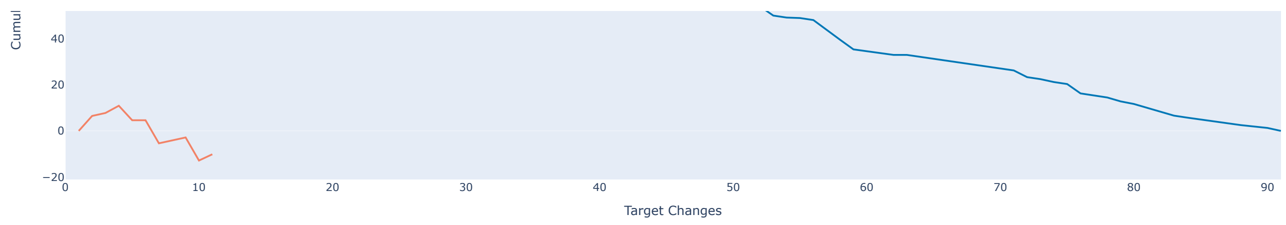


Cumulative Likelihood whole genome

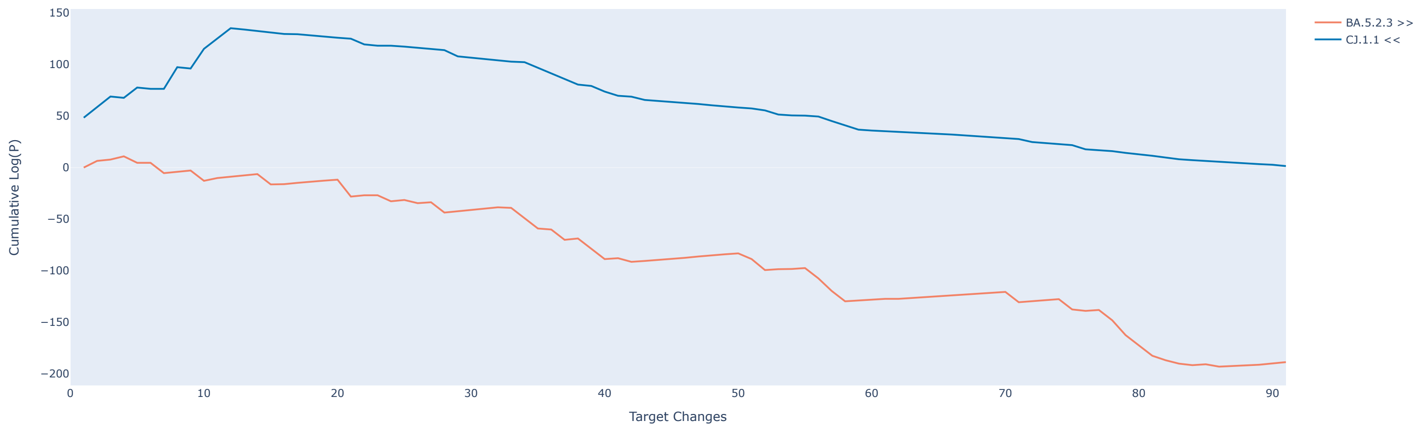


**Target sequence**

, 241_C|T, 625_G|T, 670_T|G, 1627_C|T, 2790_C|T, 3037_C|T, 3339_T|C, 4184_G|A, 4321_C|T, 6070_C|T, 8692_C|T, 9344_C|T, 9424_A|G, 9534_C|T, 9866_C|T, 10029_C|T, 10198_C|T, 10447_G|A, 10449_C|A, 11288_11296, 12444_A|G, 12880_C|T, 14408_C|T, 15451_G|A, 15714_C|T, 17410_C|T, 18163_A|G, 18583_G|A, 19955_C|T, 20055_A|G, 21618_C|T, 21633_21641, 21987_G|A, 22001_A|G, 22016_T|C, 22033_C|A, 22190_A|G, 22200_T|G, 22331_G|A, 22577_G|C, 22578_G|A, 22599_G|C, 22674_C|T, 22679_T|C, 22686_C|T, 22688_A|G, 22775_G|A, 22786_A|C, 22813_G|T, 22882_T|G, 22898_G|A, 22942_T|G, 22992_G|A, 22995_C|A, 23013_A|C, 23018_T|C, 23019_T|C, 23031_T|C, 23055_A|G, 23063_A|T, 23075_T|C, 23403_A|G, 23525_C|T, 23599_T|G, 23604_C|A, 23854_C|A, 23948_G|T, 24424_A|T, 24469_T|A, 25000_C|T, 25416_C|T, 25584_C|T, 26060_C|T, 26270_C|T, 26275_A|G, 26577_C|G, 26709_G|A, 26858_C|T, 27259_A|C, 27382_G|C, 27383_A|T, 27384_T|C, 27807_C|T, 28271_A|T, 28311_C|T, 28362_28370, 28881_G|A, 28882_G|A, 28883_G|C, 29510_A|C, 29734_29759

## Case 15 (1BP mid): XBG test: OK

Target: (75%) 36 samples
Number of changes: 78
GT: BA.2.76 + BA.5.2
GT BR: 32–41
GT BR coord: 22600 – 22916
Rank L1 L2: 1 1
BC: BA.2.76 + BA.5.2
BC BR: 23–24
BC BR coord: 19814 – 19815
Direction L1: <<
Initial region span: 1–23,24–78
Gap history (edge excluded):
Alt. candidates: [BA.2], [BA.5.2.6]
Model 1BP/2BP comparison: 1BP vs 2BP: 4.24e–02
Rec. model vs L1: 1.77e–82
Rec. model vs L2: 8.88e–44
Flags: Model_1BP_Best

### BA.2.76 >>

| | num_seq | t_ch_MAX | max_CL | CL@BC_t_ch_MAX | aic | PV | PV_OK | t_ch_MAX_OK | phyl_OK |
|---|---|---|---|---|---|---|---|---|---|
| **BA.2.76** | 356 | 23 | 22.469484 | NaN | NaN | NaN | * | * | * |
| **BA.2.36** | 1320 | 22 | 20.539123 | 10.539123 | 32.921755 | 6.589718e-06 | | * | |
| **BA.2** | 359165 | 22 | 19.876246 | 19.318600 | 15.362800 | 4.285213e-02 | * | * | * |
| **BA.2.9** | 61803 | 22 | 19.088473 | 9.088473 | 35.823053 | 1.545752e-06 | | * | |
| **BA.2.12.2** | 23 | 16 | 18.867651 | -7.912830 | 69.825660 | 6.367401e-14 | | | |
| **BA.2.9.3** | 351 | 16 | 17.748944 | 2.429170 | 49.141659 | 1.980335e-09 | | | |
| **BA.2.8** | 4251 | 16 | 17.334997 | 2.015223 | 49.969553 | 1.307695e-09 | | | |
| **BA.2.12.1** | 94944 | 16 | 15.674499 | 3.081399 | 47.837203 | 3.793412e-09 | | | |
| **BA.2.38** | 706 | 16 | 15.429346 | 0.109572 | 53.780856 | 1.946146e-10 | | | |
| **BA.2.3** | 22517 | 22 | 15.290053 | 5.290053 | 43.419893 | 3.457967e-08 | | * | |

### BA.5.2 <<

| | num_seq | t_ch_MAX | max_CL | CL@BC_t_ch_MAX | aic | PV | PV_OK | t_ch_MAX_OK | phyl_OK |
|---|---|---|---|---|---|---|---|---|---|
| **BA.5.2** | 32711 | 24 | 55.288042 | NaN | NaN | NaN | * | * | * |
| **BA.5.2.6** | 2139 | 24 | 45.673096 | 45.673096 | 32.653808 | 6.672039e-05 | * | * | * |
| **CN.2** | 183 | 31 | 35.480300 | 22.883952 | 78.232096 | 8.446705e-15 | | | * |
| **BA.5.2.13** | 710 | 31 | 35.440575 | 31.326649 | 61.346703 | 3.909604e-11 | | | * |
| **BA.5.2.28** | 617 | 31 | 32.471216 | 28.362657 | 67.274686 | 2.025914e-12 | | | * |
| **BA.5.2.39** | 70 | 31 | 31.952150 | 27.856642 | 68.286716 | 1.216553e-12 | | | * |
| **CF.1** | 42 | 33 | 31.484112 | 7.549491 | 108.901017 | 1.848338e-21 | | | * |
| **BA.5.2.31** | 603 | 33 | 31.413764 | 10.067269 | 103.865462 | 2.285768e-20 | | | * |
| **BA.5.2.58** | 79 | 33 | 31.407016 | 10.544527 | 102.910946 | 3.693970e-20 | | | * |
| **BA.5.2.37** | 68 | 33 | 31.393858 | 14.210430 | 95.579140 | 1.442722e-18 | | | * |

Cumulative Likelihood per-region

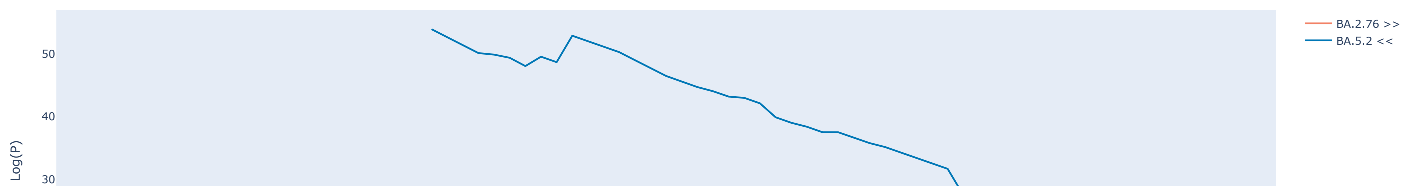

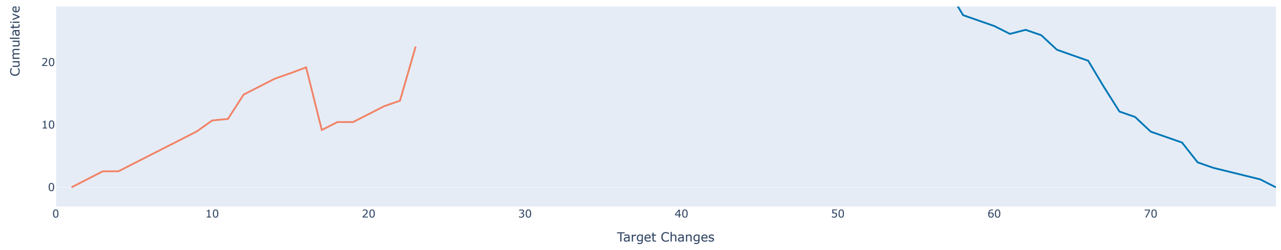


Cumulative Likelihood whole genome

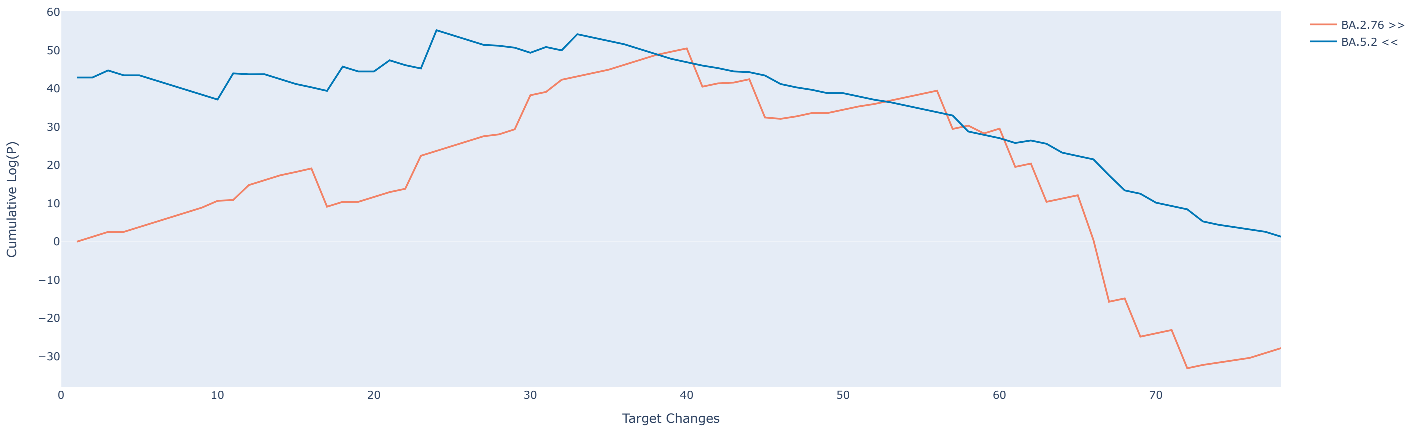


**Target sequence**

, 241_C|T, 670_T|G, 2790_C|T, 3037_C|T, 4184_G|A, 4321_C|T, 9344_C|T, 9424_A|G, 9534_C|T, 9866_C|T, 10029_C|T, 10116_C|T, 10198_C|T, 10447_G|A, 10449_C|A, 11288_11296, 11665_C|T, 12880_C|T, 14408_C|T, 15714_C|T, 17410_C|T, 18163_A|G, 19812_A|G, 19955_C|T, 20055_A|G, 21618_C|T, 21633_21641, 21987_G|A, 22200_T|G, 22304_T|A, 22578_G|A, 22599_G|C, 22674_C|T, 22679_T|C, 22686_C|T, 22688_A|G, 22775_G|A, 22786_A|C, 22813_G|T, 22882_T|G, 22917_T|G, 22992_G|A, 22995_C|A, 23013_A|C, 23018_T|G, 23055_A|G, 23063_A|T, 23075_T|C, 23403_A|G, 23525_C|T, 23599_T|G, 23604_C|A, 23854_C|A, 23948_G|T, 24424_A|T, 24469_T|A, 24676_A|G, 25000_C|T, 25584_C|T, 26060_C|T, 26180_T|C, 26270_C|T, 26529_G|A, 26577_C|G, 26709_G|A, 27012_C|T, 27513_C|T, 27807_C|T, 27889_C|T, 28271_A|T, 28311_C|T, 28330_A|G, 28362_28370, 28881_G|A, 28882_G|A, 28883_G|C, 29510_A|C, 29734_29759

## Case 16 (1BP mid): XBH test: KO

Target: (75%) 10 samples
GT: BA.2.3.17 + BA.2.75.2
BC: BA.2.75.2 + BA.2.3.17 + BA.2.75.2
Direction L1: <<
Alt. candidates: [], [CA.3]
Model 1BP/2BP comparison:
Rec. model vs L1: 2.52e−68
Flags: Model_2BP_Best

Number of changes: 84
GT BR: 19−28
BC BR: 4−5, 19−20
Initial region span: 1−4,8−19,20−84
2BP vs 1BP: 3.35e−03
Rec. model vs L2: 3.16e−139

GT BR coord: 15450 − 22001
BC BR coord: 3037 − 3038, 15713 − 15714
Gap history (edge excluded): 4−20 −> 4−8

Rank L1 L2: 11 11 −

### BA.2.75.2 >>

| | num_seq | t_ch_MAX | max_CL | CL@BC_t_ch_MAX | aic | PV | PV_OK | t_ch_MAX_OK | phyl_OK |
|---|---|---|---|---|---|---|---|---|---|
| **BA.2.75.2** | 582 | 4 | 2.56067 | None | None | None | * | * | * |

### BA.2.3.17 <<

| | num_seq | t_ch_MAX | max_CL | CL@BC_t_ch_MAX | aic | PV | PV_OK | t_ch_MAX_OK | phyl_OK |
|---|---|---|---|---|---|---|---|---|---|
| **BA.2.3.17** | 1067 | 8 | 18.058003 | NaN | NaN | NaN | * | * | * |
| **BA.2.24** | 82 | 8 | 15.255018 | -0.099392 | 42.198784 | 1.940791e-07 | | * | |
| **XBB.2** | 470 | 5 | 14.390962 | 14.390962 | 13.218075 | 3.809832e-01 | * | | |
| **BA.2.20** | 357 | 5 | 14.224026 | 14.224026 | 13.551948 | 3.230333e-01 | * | | |
| **XBB.1.17.1** | 49 | 8 | 13.980666 | 6.517423 | 28.965154 | 1.448227e-04 | * | * | |
| **BA.2.10** | 12936 | 5 | 12.214761 | 12.214761 | 17.570478 | 4.328280e-02 | * | | |
| **BA.2** | 359165 | 5 | 11.291286 | 11.291286 | 19.417428 | 1.716299e-02 | * | | * |
| **BA.2.21** | 527 | 8 | 10.249214 | -13.142938 | 68.285876 | 4.193796e-13 | | * | |
| **XBB.1.5** | 35885 | 5 | 10.234246 | 10.234246 | 21.531509 | 5.976023e-03 | * | | |
| **BA.2.23** | 8100 | 5 | 10.069112 | 10.069112 | 21.861777 | 5.067032e-03 | * | | |

### BA.2.75.2 <<

| | num_seq | t_ch_MAX | max_CL | CL@BC_t_ch_MAX | aic | PV | PV_OK | t_ch_MAX_OK | phyl_OK |
|---|---|---|---|---|---|---|---|---|---|
| **BA.2.75.2** | 582 | 20 | 110.337790 | NaN | NaN | NaN | * | * | * |
| **CA.7** | 115 | 22 | 107.202371 | 103.131179 | -46.262359 | 5.714035e-05 | * | | * |
| **CA.3** | 34 | 20 | 101.120966 | 103.688520 | -47.377041 | 1.000340e-04 | * | * | * |
| **XBF.5** | 14 | 23 | 95.847572 | 95.784296 | -31.568592 | 3.690200e-08 | | | |
| **CA.1** | 134 | 20 | 93.970328 | 96.522844 | -33.045689 | 7.734422e-08 | | * | * |
| **CH.1.1** | 2870 | 25 | 92.780448 | 88.784965 | -17.569929 | 3.365027e-11 | | | |
| **BY.1** | 153 | 20 | 92.584887 | 95.145884 | -30.291768 | 1.945815e-08 | | * | |
| **BM.1.1** | 104 | 23 | 89.098219 | 89.025281 | -18.050563 | 4.277788e-11 | | | |
| **BM.4.1.1** | 124 | 23 | 89.061871 | 89.006759 | -18.013517 | 4.193082e-11 | | | |
| **BY.1.2** | 23 | 20 | 86.066121 | 88.633675 | -17.267351 | 2.896306e-11 | | * | |

Cumulative Likelihood per-region

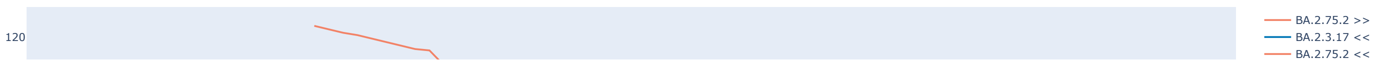

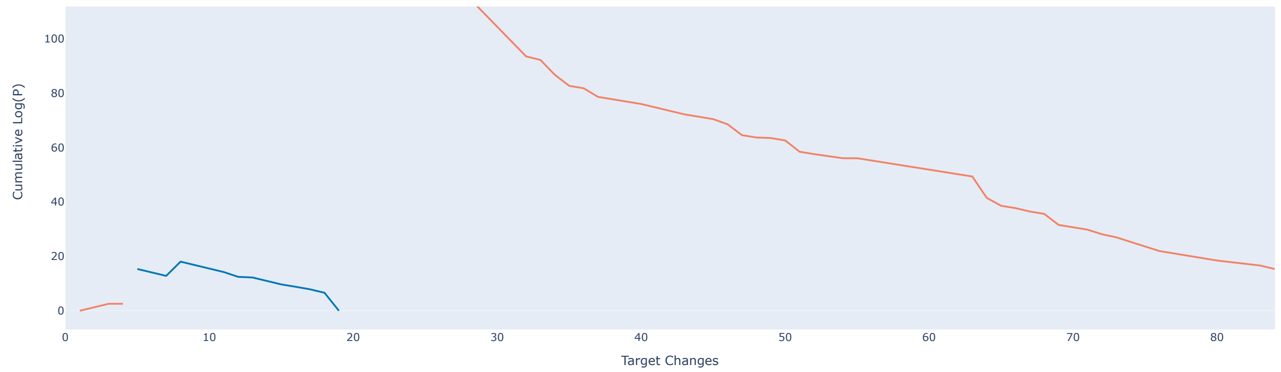


Cumulative Likelihood whole genome

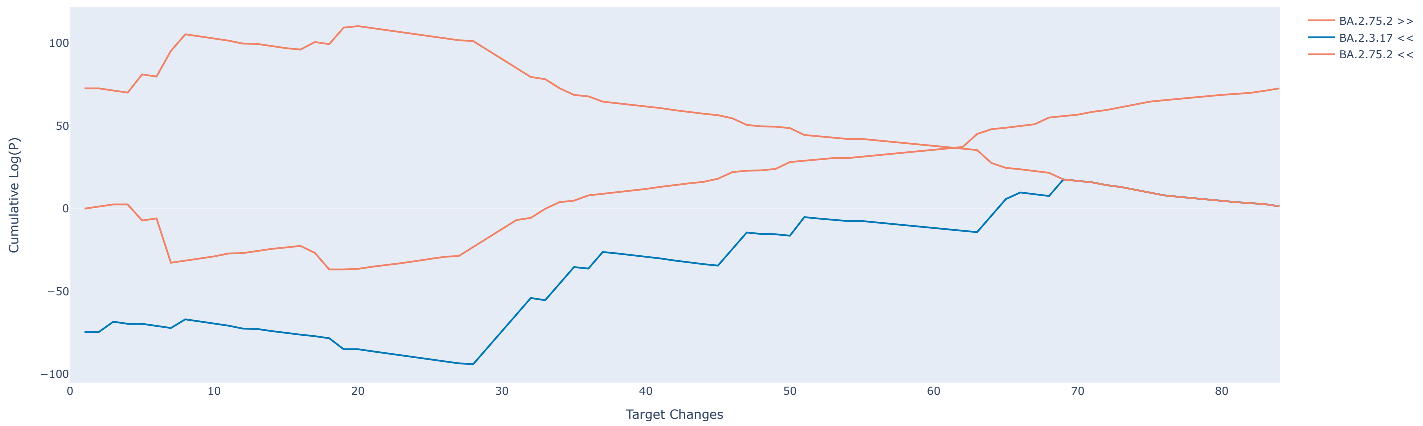


**Target sequence**

, 241_C|T, 670_T|G, 2790_C|T, 3037_C|T, 4184_G|A, 4321_C|T, 7528_C|T, 9344_C|T, 9424_A|G, 9534_C|T, 9866_C|T, 10029_C|T, 10198_C|T, 10447_G|A, 10449_C|A, 11288_11296, 12880_C|T, 13730_C|T, 14408_C|T, 15714_C|T, 17410_C|T, 18163_A|G, 19955_C|T, 20055_A|G, 21618_C|T, 21633_21641, 21987_G|A, 22001_A|G, 22016_T|C, 22033_C|A, 22190_A|G, 22200_T|G, 22331_G|A, 22577_G|C, 22578_G|A, 22599_G|C, 22674_C|T, 22679_T|C, 22686_C|T, 22688_A|G, 22775_G|A, 22786_A|C, 22813_G|T, 22882_T|G, 22898_G|A, 22942_T|G, 22992_G|A, 22995_C|A, 23013_A|C, 23019_T|C, 23055_A|G, 23063_A|T, 23075_T|C, 23403_A|G, 23525_C|T, 23599_T|G, 23604_C|A, 23854_C|A, 23948_G|T, 24424_A|T, 24469_T|A, 25000_C|T, 25157_G|A, 25416_C|T, 25584_C|T, 26060_C|T, 26270_C|T, 26275_A|G, 26577_C|G, 26709_G|A, 26858_C|T, 27259_A|C, 27382_G|C, 27383_A|T, 27384_T|C, 27807_C|T, 28271_A|T, 28311_C|T, 28362_28370, 28881_G|A, 28882_G|A, 28883_G|C, 29510_A|C, 29734_29759

## Case 17 (1BP mid): XBJ test: OK

Target: (75%) 9 samples
GT: BA.2.3.20 + BA.5.2*
BC: BA.2.3.20 + BA.5.2.62
Direction L1: >>
Alt. candidates: [], [BA.5.2.6, BA.5.2]
Model 1BP/2BP comparison:
Rec. model vs L1: 8.73e-113
Flags: Model_1BP_Best

Number of changes: 92
GT BR: 60-74
BC BR: 73-74
Initial region span: 1-73,74-92
1BP vs 2BP: 3.53e-04
Rec. model vs L2: 1.44e-254

GT BR coord: 23014 - 25810
BC BR coord: 25593 - 25594
Gap history (edge excluded):

Rank L1 L2: 1 7

### BA.2.3.20 >>

| | num_seq | t_ch_MAX | max_CL | CL@BC_t_ch_MAX | aic | PV | PV_OK | t_ch_MAX_OK | phyl_OK |
|---|---|---|---|---|---|---|---|---|---|
| **BA.2.3.20** | 409 | 73 | 178.739797 | NaN | NaN | NaN | * | * | * |
| **CM.2** | 237 | 72 | 172.509207 | 165.611205 | -159.222409 | 1.984785e-06 | | * | * |
| **CM.11** | 15 | 73 | 160.870034 | 160.870034 | -149.740067 | 1.734433e-08 | | * | * |
| **CM.5** | 22 | 73 | 160.053102 | 160.053102 | -148.106204 | 7.677284e-09 | | * | * |
| **CM.4** | 40 | 73 | 159.614533 | 159.614533 | -147.229067 | 4.944451e-09 | | * | * |
| **CM.3** | 13 | 60 | 158.147675 | 149.595583 | -127.191166 | 2.200327e-13 | | | * |
| **CM.9** | 18 | 72 | 155.370363 | 148.468132 | -124.936263 | 7.143417e-14 | | * | * |
| **CM.10** | 34 | 73 | 153.617186 | 153.617186 | -135.234373 | 1.225607e-11 | | * | * |
| **CM.5.2** | 39 | 60 | 150.036595 | 149.228663 | -126.457325 | 1.527460e-13 | | | * |
| **CM.8.1** | 136 | 73 | 145.810539 | 145.810539 | -119.621078 | 4.996694e-15 | | * | * |

### BA.5.2.62 <<

| | num_seq | t_ch_MAX | max_CL | CL@BC_t_ch_MAX | aic | PV | PV_OK | t_ch_MAX_OK | phyl_OK |
|---|---|---|---|---|---|---|---|---|---|
| **BA.5.2.62** | 81 | 74 | 35.633236 | NaN | NaN | NaN | * | * | * |
| **BA.5.2.38** | 13 | 74 | 35.024852 | 35.024852 | -32.049704 | 5.433509e-01 | * | * | |
| **BA.5.2.36** | 60 | 74 | 33.479053 | 33.479053 | -28.958107 | 1.159032e-01 | * | * | |
| **BA.5.2.6** | 2139 | 74 | 33.297406 | 33.297406 | -28.594811 | 9.632764e-02 | * | * | * |
| **BA.5.2.56** | 85 | 74 | 33.207049 | 33.207049 | -28.414098 | 8.803683e-02 | * | * | |
| **BA.5.2.24** | 84 | 74 | 33.013660 | 33.013660 | -28.027320 | 7.280286e-02 | * | * | |
| **BA.5.2** | 32711 | 74 | 32.108188 | 32.108188 | -26.216376 | 2.945181e-02 | * | * | * |
| **BA.5.2.57** | 288 | 74 | 31.854261 | 31.854261 | -25.708522 | 2.282269e-02 | * | * | |
| **CK.1** | 718 | 74 | 29.965227 | 29.965227 | -21.930455 | 3.447865e-03 | * | * | |
| **DQ.1** | 44 | 75 | 28.425831 | 18.425831 | 1.148339 | 3.355768e-08 | | * | |

Cumulative Likelihood per-region

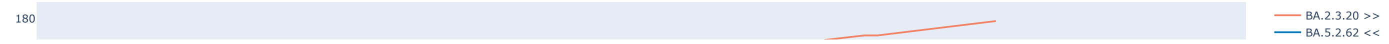

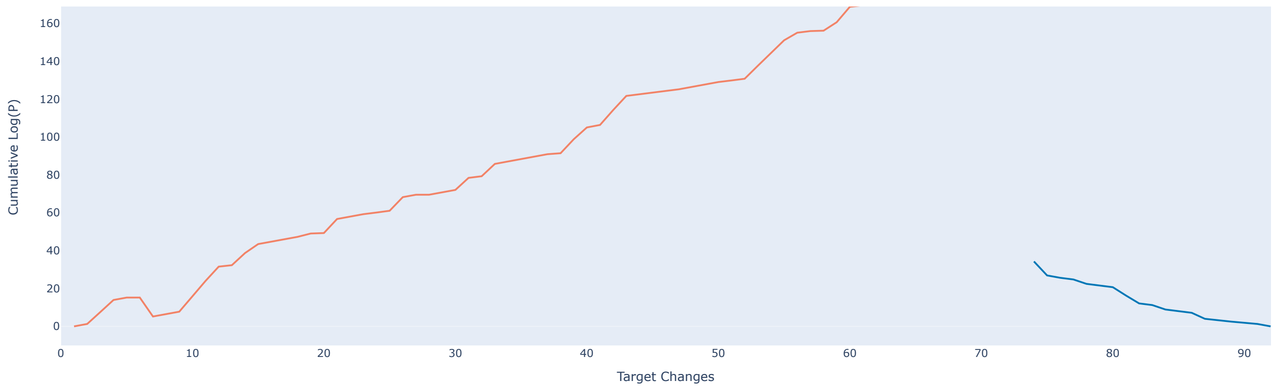


Cumulative Likelihood whole genome

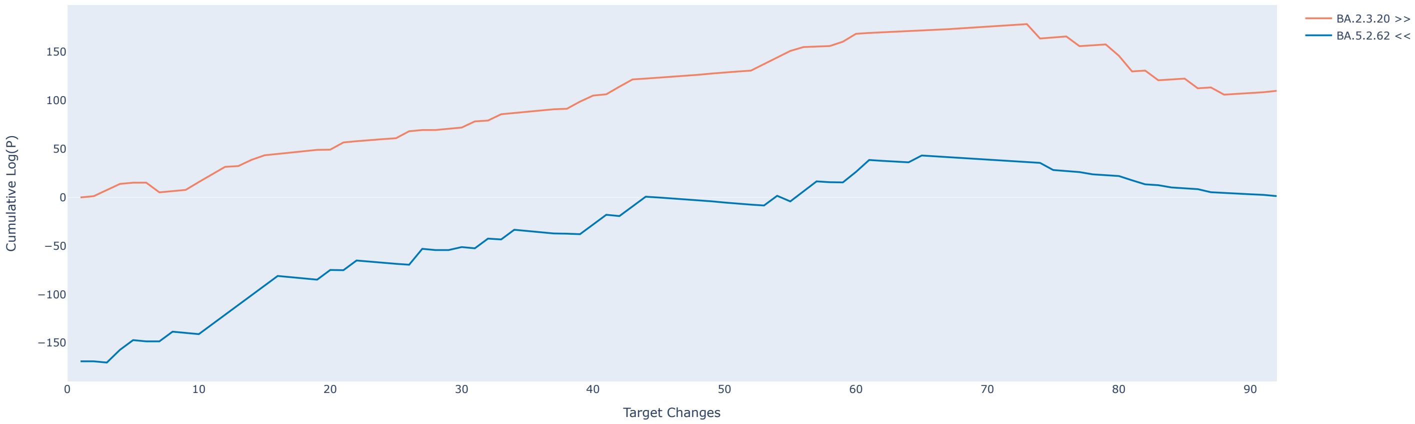


**Target sequence**

, 241_C|T, 670_T|G, 1471_C|T, 2445_C|T, 2790_C|T, 3037_C|T, 3072_G|A, 4184_G|A, 4321_C|T, 5406_T|C, 6770_A|G, 6786_C|T, 8016_C|T, 8208_C|T, 8991_C|T, 9344_C|T, 9424_A|G, 9534_C|T, 9866_C|T, 10029_C|T, 10189_C|T, 10198_C|T, 10447_G|A, 10449_C|A, 11288_11296, 12030_A|G, 12880_C|T, 14408_C|T, 15714_C|T, 17410_C|T, 17678_C|T, 18163_A|G, 18252_C|T, 19955_C|T, 20055_A|G, 21618_C|T, 21633_21641, 21987_G|A, 22020_T|C, 22054_T|G, 22200_T|G, 22295_C|A, 22332_G|A, 22578_G|A, 22674_C|T, 22679_T|C, 22686_C|T, 22688_A|G, 22775_G|A, 22786_A|C, 22813_G|T, 22882_T|G, 22893_A|G, 22910_A|G, 22916_C|A, 22942_T|G, 22992_G|A, 22995_C|A, 23012_G|A, 23013_A|G, 23055_A|G, 23063_A|T, 23075_T|C, 23403_A|G, 23525_C|T, 23599_T|G, 23604_C|A, 23854_C|A, 23948_G|T, 24424_A|T, 24469_T|A, 25000_C|T, 25584_C|T, 25896_C|T, 26060_C|T, 26270_C|T, 26529_G|A, 26577_C|G, 26709_G|A, 27012_C|T, 27513_C|T, 27807_C|T, 27889_C|T, 28271_A|T, 28311_C|T, 28330_A|G, 28362_28370, 28881_G|A, 28882_G|A, 28883_G|C, 29510_A|C, 29734_29759

## Case 18 (1BP mid): XBM test: OK

Target: (75%) 49 samples Number of changes: 77
GT: BA.2.76 + BF.3 GT BR: 31–40 GT BR coord: 22600 – 22916 Rank L1 L2: 1 3
BC: BA.2.76 + BF.3.1 BC BR: 31–32 BC BR coord: 22599 – 22600
Direction L1: << Initial region span: 1–31,32–77 Gap history (edge excluded):
Alt. candidates: [], []
Model 1BP/2BP comparison: 1BP vs 2BP: 4.42e–02
Rec. model vs L1: 3.87e–133 Rec. model vs L2: 1.40e–69
Flags: Model_1BP_Best

### BA.2.76 >>

| | num_seq | t_ch_MAX | max_CL | CL@BC_t_ch_MAX | aic | PV | PV_OK | t_ch_MAX_OK | phyl_OK |
|---|---|---|---|---|---|---|---|---|---|
| **BA.2.76** | 356 | 31 | 38.452194 | NaN | NaN | NaN | * | * | * |
| **BA.2** | 359165 | 28 | 24.237436 | 19.364848 | 41.270304 | 5.146237e-09 | | | * |
| **BA.2.38.3** | 12 | 20 | 19.967271 | -26.383347 | 132.766693 | 6.958526e-29 | | | |
| **BA.2.17** | 37 | 20 | 19.967271 | -2.634541 | 85.269081 | 1.435526e-18 | | | |
| **BA.2.40** | 27 | 20 | 19.967271 | -18.991347 | 117.982694 | 1.132726e-25 | | | |
| **BA.2.9.4** | 13 | 20 | 19.967271 | -15.321920 | 110.643840 | 4.446166e-24 | | | |
| **BA.2.8** | 4251 | 20 | 19.965153 | -18.975666 | 117.951331 | 1.149845e-25 | | | |
| **BA.2.9.3** | 351 | 20 | 19.964418 | -20.768083 | 121.536165 | 1.910209e-26 | | | |
| **BA.2.72** | 268 | 20 | 19.959794 | -19.551625 | 119.103249 | 6.470235e-26 | | | |
| **BA.2.18** | 3723 | 20 | 19.956519 | -6.591635 | 93.183270 | 2.750278e-20 | | | |

### BF.3.1 <<

| | num_seq | t_ch_MAX | max_CL | CL@BC_t_ch_MAX | aic | PV | PV_OK | t_ch_MAX_OK | phyl_OK |
|---|---|---|---|---|---|---|---|---|---|
| **BF.3.1** | 271 | 32 | 65.589660 | NaN | NaN | NaN | * | * | * |
| **BF.26** | 3508 | 30 | 50.048620 | 47.904110 | 12.191779 | 2.086902e-08 | | | |
| **BF.3** | 55 | 32 | 48.934759 | 48.934759 | 10.130482 | 5.845550e-08 | | * | * |
| **BF.28** | 2148 | 32 | 46.833157 | 46.833157 | 14.333686 | 7.158252e-09 | | * | |
| **BA.5.2.1** | 58578 | 32 | 45.914577 | 45.914577 | 16.170846 | 2.852700e-09 | | * | * |
| **BA.5.2.6** | 2139 | 23 | 42.415182 | 28.063082 | 51.873835 | 5.047770e-17 | | | |
| **BF.10** | 7646 | 30 | 37.802517 | 36.251508 | 35.496984 | 1.810506e-13 | | | |
| **BF.5** | 6397 | 32 | 37.497138 | 37.497138 | 33.005725 | 6.287768e-13 | | * | |
| **BF.7** | 4385 | 30 | 37.435365 | 33.384203 | 41.231594 | 1.031683e-14 | | | |
| **BF.7.4.1** | 1026 | 30 | 34.663876 | 30.612029 | 46.775942 | 6.432489e-16 | | | |

Cumulative Likelihood per-region

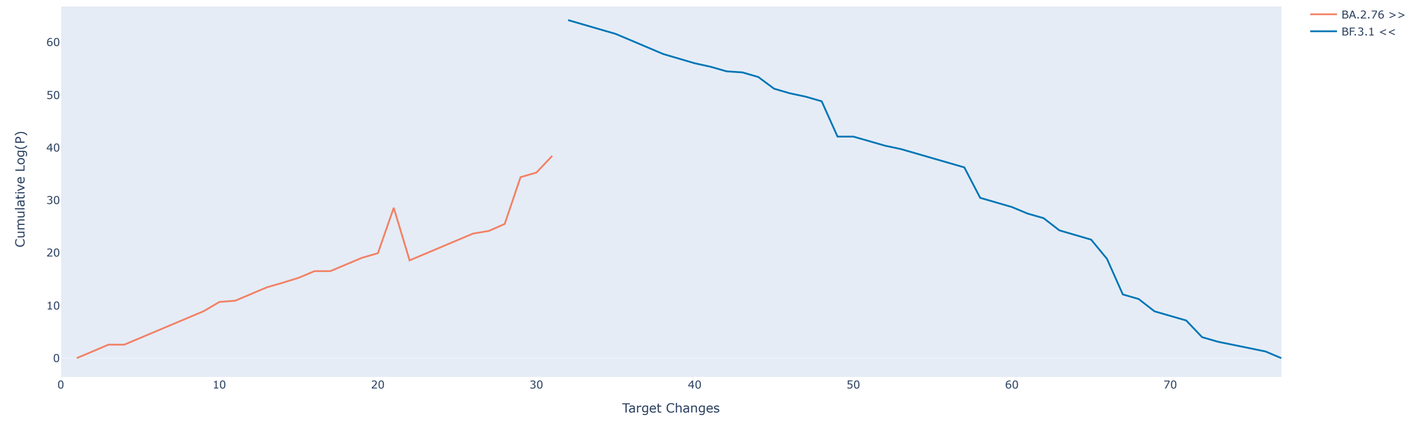


Cumulative Likelihood whole genome

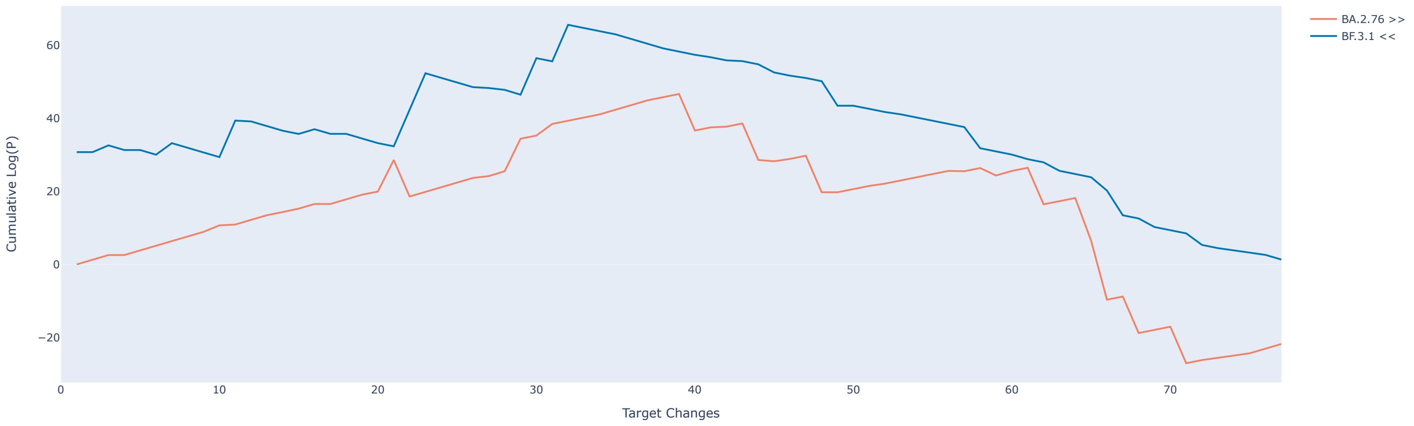


**Target sequence**

, 241_C|T, 670_T|G, 2790_C|T, 3037_C|T, 4184_G|A, 4321_C|T, 9344_C|T, 9424_A|G, 9534_C|T, 9866_C|T, 10029_C|T, 10198_C|T, 10447_G|A, 10449_C|A, 11288_11296, 12880_C|T, 14408_C|T, 15714_C|T, 17410_C|T, 18163_A|G, 19812_A|G, 19891_G|A, 19955_C|T, 20055_A|G, 21618_C|T, 21633_21641, 21987_G|A, 22200_T|G, 22304_T|A, 22578_G|A, 22599_G|C, 22674_C|T, 22679_T|C, 22686_C|T, 22688_A|G, 22775_G|A, 22786_A|C, 22813_G|T, 22882_T|G, 22917_T|G, 22992_G|A, 22995_C|A, 23013_A|C, 23018_T|G, 23055_A|G, 23063_A|T, 23075_T|C, 23202_C|T, 23403_A|G, 23525_C|T, 23599_T|G, 23604_C|A, 23854_C|A, 23948_G|T, 24424_A|T, 24469_T|A, 24620_G|T, 25000_C|T, 25584_C|T, 26060_C|T, 26270_C|T, 26529_G|A, 26577_C|G, 26709_G|A, 27038_A|G, 27549_C|T, 27807_C|T, 27889_C|T, 28271_A|T, 28311_C|T, 28330_A|G, 28362_28370, 28881_G|A, 28882_G|A, 28883_G|C, 29510_A|C, 29734_29759

## Case 19 (1BP mid): XBP                test: OK

Target: (75%) 23 samples
Number of changes: 88
GT: BA.2.75* + BQ.1*
GT BR: 40–42
GT BR coord: 22192 – 22331
Rank L1 L2: 11 7
BC: BL.1 + BQ.1.1.3
BC BR: 41–42
BC BR coord: 22200 – 22201
Direction L1: >>
Initial region span: 1–41,43–88
Gap history (edge excluded): 41–43
Alt. candidates: [BA.2.75.1], [BQ.1]
Model 1BP/2BP comparison: 1BP vs 2BP: 2.19e–06
Rec. model vs L1: 1.04e–150
Rec. model vs L2: 1.11e–170
Flags: Model_1BP_Best

## BL.1 >>

| | num_seq | t_ch_MAX | max_CL | CL@BC_t_ch_MAX | aic | PV | PV_OK | t_ch_MAX_OK | phyl_OK |
|---|---|---|---|---|---|---|---|---|---|
| **BL.1** | 158 | 41 | 63.259120 | NaN | NaN | NaN | * | * | * |
| **BL.2** | 59 | 41 | 58.531935 | 58.531935 | -25.063870 | 8.826471e-03 | * | * | |
| **BA.2.75.1** | 158 | 41 | 52.697170 | 52.697170 | -13.394341 | 2.580351e-05 | * | * | * |
| **BA.2.75.5** | 215 | 41 | 48.089533 | 48.089533 | -4.179066 | 2.580790e-07 | | * | |
| **CA.3.1** | 148 | 41 | 41.942610 | 41.942610 | 8.114779 | 5.533668e-10 | | * | |
| **BN.1** | 942 | 41 | 41.023642 | 41.023642 | 9.952715 | 2.205272e-10 | | * | |
| **CB.1** | 48 | 23 | 37.068601 | 32.790851 | 26.418299 | 5.848536e-14 | | | |
| **BN.1.1** | 73 | 23 | 36.663670 | 33.352086 | 25.295829 | 1.023887e-13 | | | |
| **BN.1.7** | 232 | 23 | 36.197682 | 22.087846 | 47.824308 | 1.318549e-18 | | | |
| **BA.2.75.2** | 582 | 41 | 31.568604 | 31.568604 | 28.862793 | 1.726664e-14 | | * | |

## BQ.1.1.3 <<

| | num_seq | t_ch_MAX | max_CL | CL@BC_t_ch_MAX | aic | PV | PV_OK | t_ch_MAX_OK | phyl_OK |
|---|---|---|---|---|---|---|---|---|---|
| **BQ.1.1.3** | 1817 | 43 | 57.330542 | NaN | NaN | NaN | * | * | * |
| **BQ.1.8** | 1146 | 42 | 45.639871 | 45.639871 | 4.720258 | 0.000084 | * | * | |
| **BQ.1.23** | 776 | 42 | 45.602318 | 45.602318 | 4.795363 | 0.000081 | * | * | |
| **BQ.1.13** | 914 | 42 | 45.562608 | 45.562608 | 4.874784 | 0.000078 | * | * | |
| **BQ.1.5** | 867 | 42 | 45.562075 | 45.562075 | 4.875851 | 0.000078 | * | * | |
| **BQ.1.15** | 499 | 42 | 45.561166 | 45.561166 | 4.877669 | 0.000078 | * | * | |
| **BQ.1** | 11861 | 42 | 45.552491 | 45.552491 | 4.895018 | 0.000077 | * | * | * |
| **BQ.1.14** | 1516 | 42 | 45.551316 | 45.551316 | 4.897369 | 0.000077 | * | * | |
| **BQ.1.1.37** | 13 | 43 | 44.823212 | 42.511615 | 10.976771 | 0.000004 | | * | * |
| **ET.1** | 40 | 43 | 44.823212 | 42.511615 | 10.976771 | 0.000004 | | * | * |

Cumulative Likelihood per-region

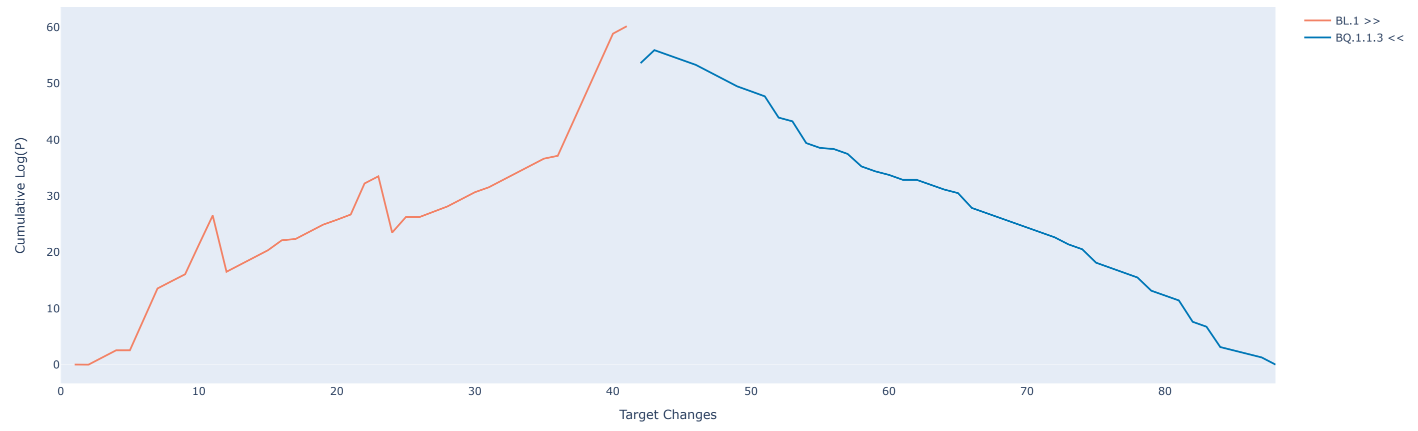


Cumulative Likelihood whole genome

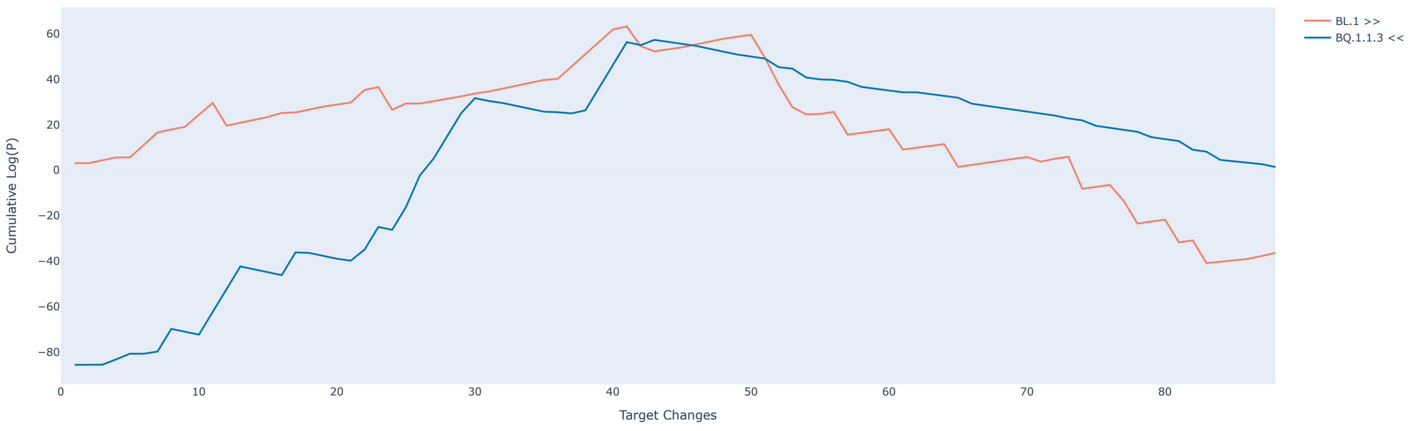


**Target sequence**

, 204_G|A, 241_C|T, 670_T|G, 2790_C|T, 3037_C|T, 3796_C|T, 3927_C|T, 4184_G|A, 4321_C|T, 4586_C|T, 5183_C|T, 7303_C|T, 9344_C|T, 9424_A|G, 9534_C|T, 9866_C|T, 10029_C|T, 10198_C|T, 10447_G|A, 10449_C|A, 11288_11296, 12444_A|G, 12880_C|T, 12952_C|T, 13965_C|T, 14408_C|T, 15277_C|T, 15451_G|A, 15714_C|T, 17410_C|T, 18163_A|G, 19955_C|T, 20055_A|G, 21618_C|T, 21633_21641, 21987_G|A, 22001_A|G, 22016_T|C, 22033_C|A, 22190_A|G, 22200_T|G, 22578_G|A, 22674_C|T, 22679_T|C, 22686_C|T, 22688_A|G, 22775_G|A, 22786_A|C, 22813_G|T, 22882_T|G, 22893_A|C, 22917_T|G, 22942_T|A, 22992_G|A, 22995_C|A, 23013_A|C, 23018_T|G, 23055_A|G, 23063_A|T, 23075_T|C, 23403_A|G, 23525_C|T, 23599_T|G, 23604_C|A, 23773_T|C, 23854_C|A, 23948_G|T, 24424_A|T, 24469_T|A, 25000_C|T, 25584_C|T, 26060_C|T, 26270_C|T, 26529_G|A, 26577_C|G, 26709_G|A, 27807_C|T, 27889_C|T, 28271_A|T, 28311_C|T, 28312_C|T, 28362_28370, 28681_G|T, 28881_G|A, 28882_G|A, 28883_G|C, 29510_A|C, 29734_29759

## Case 20 (1BP mid): XBR    test: OK

Target: (75%) 8 samples    Number of changes: 94
GT: BA.2.75 + BQ.1    GT BR: 41–42    GT BR coord: 22033 – 22190    Rank L1 L2: 11 10
BC: BN.3.1 + BQ.1.25.1    BC BR: 41–42    BC BR coord: 22035 – 22036
Direction L1: >>    Initial region span: 1–41,42–94    Gap history (edge excluded):
Alt. candidates: [], []
Model 1BP/2BP comparison:    1BP vs 2BP: 1.10e–07
Rec. model vs L1: 1.17e–236    Rec. model vs L2: 4.73e–205
Flags: Model_1BP_Best

### BN.3.1 >>

| | num_seq | t_ch_MAX | max_CL | CL@BC_t_ch_MAX | aic | PV | PV_OK | t_ch_MAX_OK | phyl_OK |
|---|---|---|---|---|---|---|---|---|---|
| **BN.3.1** | 79 | 41 | 125.794281 | NaN | NaN | NaN | * | * | * |
| **BN.1** | 942 | 41 | 18.506284 | 18.506284 | 94.987431 | 2.538312e-47 | | * | |
| **BA.2.75.5** | 215 | 41 | 16.473121 | 16.473121 | 99.053758 | 3.333706e-48 | | * | * |
| **BN.1.4** | 255 | 13 | 9.664408 | -8.550542 | 149.101085 | 4.515521e-59 | | | |
| **BN.1.2.3** | 81 | 3 | 5.538094 | -7.119325 | 146.238651 | 1.886900e-58 | | | |
| **BA.5.3.4** | 49 | 3 | 5.347576 | -151.607816 | 435.215631 | 3.346103e-121 | | | |
| **BQ.1.16** | 67 | 3 | 5.034704 | -172.310912 | 476.621823 | 3.424865e-130 | | | |
| **BA.5.2.13** | 710 | 3 | 4.867126 | -134.915535 | 401.831069 | 5.957796e-114 | | | |
| **BA.2.56** | 585 | 3 | 4.657833 | -93.381570 | 318.763141 | 6.508890e-96 | | | |
| **EF.1.1** | 323 | 3 | 4.554155 | -172.662667 | 477.325334 | 2.401424e-130 | | | |

### BQ.1.25.1 <<

| | num_seq | t_ch_MAX | max_CL | CL@BC_t_ch_MAX | aic | PV | PV_OK | t_ch_MAX_OK | phyl_OK |
|---|---|---|---|---|---|---|---|---|---|
| **BQ.1.25.1** | 556 | 42 | 80.575294 | NaN | NaN | NaN | * | * | * |
| **BQ.1.25** | 133 | 42 | 64.507571 | 64.507571 | -21.015141 | 1.054531e-07 | | * | * |
| **BQ.1.30** | 18 | 45 | 59.193826 | 51.386111 | 5.227779 | 2.103508e-13 | | | |
| **BQ.1.1** | 26162 | 42 | 47.292362 | 47.292362 | 13.415275 | 3.503549e-15 | | * | |
| **BQ.1.1.18** | 1461 | 42 | 45.875446 | 45.875446 | 16.249108 | 8.511017e-16 | | * | |
| **BQ.1.1.32** | 1089 | 42 | 42.427825 | 42.427825 | 23.144350 | 2.715420e-17 | | * | |
| **BQ.1.8** | 1146 | 45 | 39.150061 | 31.341473 | 45.317054 | 4.144873e-22 | | | |
| **BQ.1.1.52** | 404 | 42 | 36.333721 | 36.333721 | 35.332558 | 6.120855e-20 | | * | |
| **BQ.1.1.67** | 167 | 42 | 34.440705 | 27.989070 | 52.021860 | 1.454202e-23 | | * | |
| **BQ.1** | 11861 | 45 | 32.339514 | 24.530786 | 58.938428 | 4.570522e-25 | | | * |

Cumulative Likelihood per-region

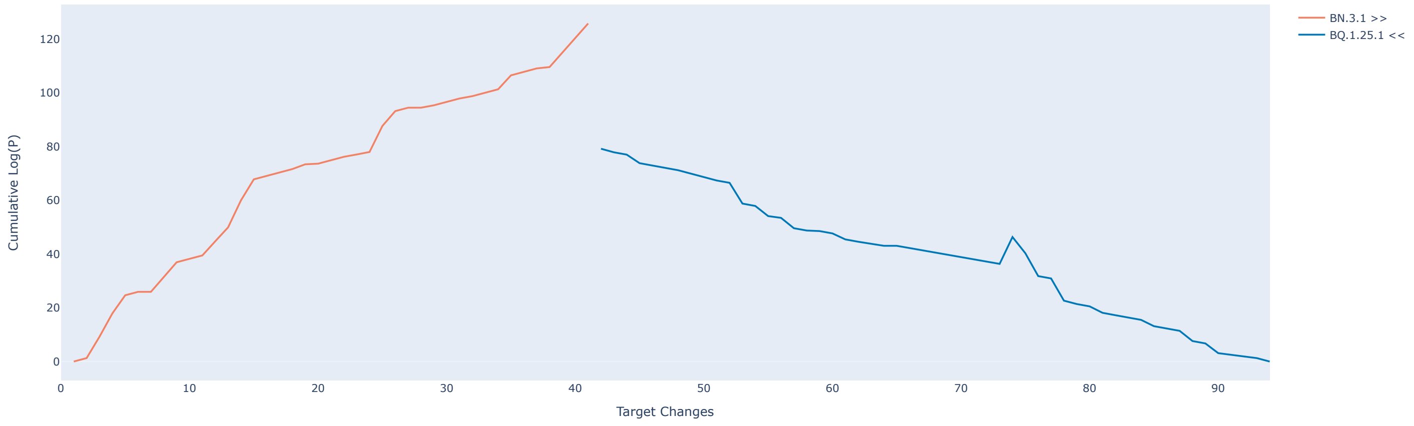


Cumulative Likelihood whole genome

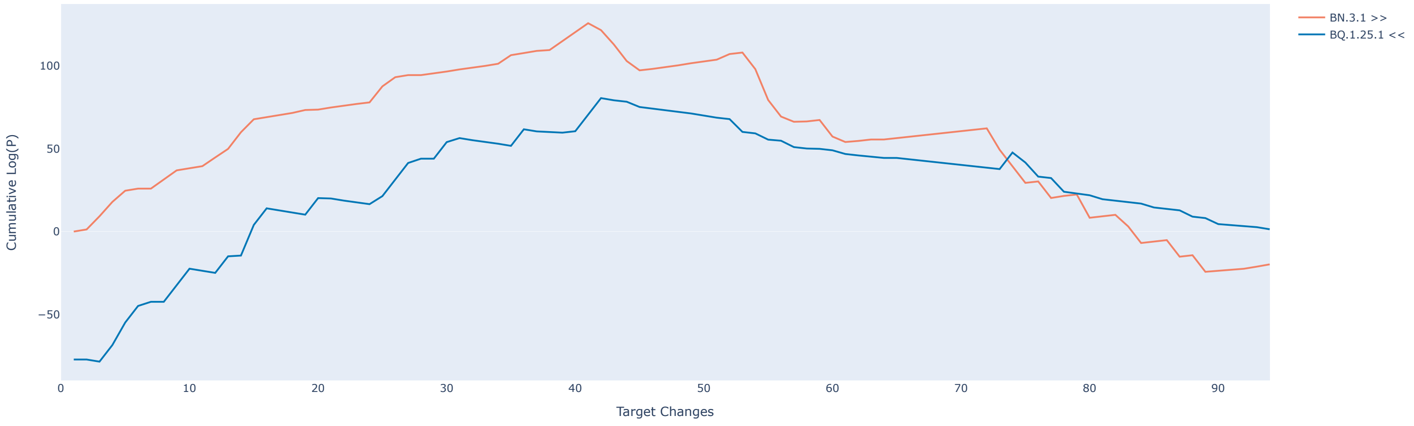


**Target sequence**

, 241_C|T, 670_T|G, 1093_C|T, 1440_G|A, 1964_A|G, 2790_C|T, 3037_C|T, 3796_C|T, 3927_C|T, 4184_G|A, 4321_C|T, 4586_C|T, 5183_C|T, 5844_A|G, 8597_C|T, 9344_C|T, 9424_A|G, 9534_C|T, 9866_C|T, 10029_C|T, 10198_C|T, 10447_G|A, 10449_C|A, 11288_11296, 12440_T|C, 12444_A|G, 12880_C|T, 14408_C|T, 15451_G|A, 15714_C|T, 17410_C|T, 18163_A|G, 19955_C|T, 20055_A|G, 21077_C|T, 21618_C|T, 21633_21641, 21987_G|A, 22001_A|G, 22016_T|C, 22033_C|A, 22200_T|G, 22578_G|A, 22599_G|C, 22674_C|T, 22679_T|C, 22686_C|T, 22688_A|G, 22775_G|A, 22786_A|C, 22813_G|T, 22855_C|T, 22882_T|G, 22893_A|C, 22917_T|G, 22942_T|A, 22992_G|A, 22995_C|A, 23013_A|C, 23018_T|G, 23055_A|G, 23063_A|T, 23075_T|C, 23403_A|G, 23525_C|T, 23599_T|G, 23604_C|A, 23854_C|A, 23948_G|T, 24424_A|T, 24469_T|A, 25000_C|T, 25471_G|C, 25553_C|T, 25569_T|C, 25584_C|T, 26031_G|A, 26060_C|T, 26270_C|T, 26529_G|A, 26577_C|G, 26709_G|A, 27807_C|T, 27889_C|T, 28271_A|T, 28311_C|T, 28312_C|T, 28362_28370, 28681_G|T, 28881_G|A, 28882_G|A, 28883_G|C, 29510_A|C, 29734_29759

## Case 21 (1BP mid): XBW test: OK

Target: (75%) 5 samples
GT: XBB.1.5 + BQ.1.14
BC: XBB.1.5 + BQ.1.14
Direction L1: >>
Alt. candidates: [], [BQ.1.1, BQ.1]
Model 1BP/2BP comparison:
Rec. model vs L1: 9.08e-72
Flags: Model_1BP_Best

Number of changes: 95
GT BR: 76-80
BC BR: 79-80
Initial region span: 1-79,80-95
1BP vs 2BP: 3.11e-07
Rec. model vs L2: 8.87e-266

GT BR coord: 25416 - 26275
BC BR coord: 26274 - 26275
Gap history (edge excluded):

Rank_L1_L2: 1 1

### XBB.1.5 >>

| | num_seq | t_ch_MAX | max_CL | CL@BC_t_ch_MAX | aic | PV | PV_OK | t_ch_MAX_OK | phyl_OK |
|---|---|---|---|---|---|---|---|---|---|
| **XBB.1.5** | 35885 | 79 | 144.019078 | NaN | NaN | NaN | * | * | * |
| **XBB.1.5.35** | 273 | 74 | 127.049366 | 125.566116 | -73.132231 | 9.662630e-09 | | | * |
| **XBB.1.5.18** | 338 | 79 | 126.402203 | 126.402203 | -74.804406 | 2.227056e-08 | | * | * |
| **XBB.1.5.31** | 410 | 71 | 126.265268 | 116.371946 | -54.743892 | 9.812001e-13 | | | * |
| **XBB.1.5.4** | 250 | 71 | 126.237802 | 117.247920 | -56.495841 | 2.365575e-12 | | | * |
| **XBB.1.5.3** | 82 | 79 | 126.119075 | 126.119075 | -74.238151 | 1.683173e-08 | | * | * |
| **XBB.1.5.1** | 556 | 79 | 125.936076 | 125.936076 | -73.872153 | 1.398892e-08 | | * | * |
| **XBB.1.5.13** | 1073 | 79 | 124.337003 | 124.337003 | -70.674005 | 2.824315e-09 | | * | * |
| **XBB.1.5.8** | 100 | 71 | 120.889240 | 112.860002 | -47.720003 | 2.933485e-14 | | | * |
| **XBB.1.5.6** | 62 | 71 | 120.874181 | 109.980719 | -41.961437 | 1.646705e-15 | | | * |

### BQ.1.14 <<

| | num_seq | t_ch_MAX | max_CL | CL@BC_t_ch_MAX | aic | PV | PV_OK | t_ch_MAX_OK | phyl_OK |
|---|---|---|---|---|---|---|---|---|---|
| **BQ.1.14** | 1516 | 80 | 28.068746 | NaN | NaN | NaN | * | * | * |
| **DT.2** | 418 | 80 | 24.750862 | 24.750862 | -11.501724 | 3.615283e-02 | * | * | |
| **BQ.1.1** | 26162 | 80 | 23.142908 | 23.142908 | -8.285816 | 7.262726e-03 | * | * | * |
| **BQ.1.6** | 405 | 80 | 22.657429 | 15.787765 | 6.424471 | 4.643696e-06 | | * | |
| **BQ.1.22** | 216 | 82 | 22.038366 | 20.813692 | -3.627384 | 7.066324e-04 | * | | |
| **BQ.1.23** | 776 | 80 | 22.028114 | 22.028114 | -6.056228 | 2.381559e-03 | * | * | |
| **BQ.1** | 11861 | 80 | 20.877291 | 20.877291 | -3.754582 | 7.503281e-04 | * | * | * |
| **BQ.1.10.1** | 281 | 83 | 18.892391 | 16.762348 | 4.475304 | 1.224981e-05 | * | | |
| **DM.1** | 15 | 80 | 11.980647 | 11.980647 | 14.038706 | 1.028494e-07 | | * | |
| **DN.1.1** | 169 | 80 | 11.980647 | 11.980647 | 14.038706 | 1.028494e-07 | | * | |

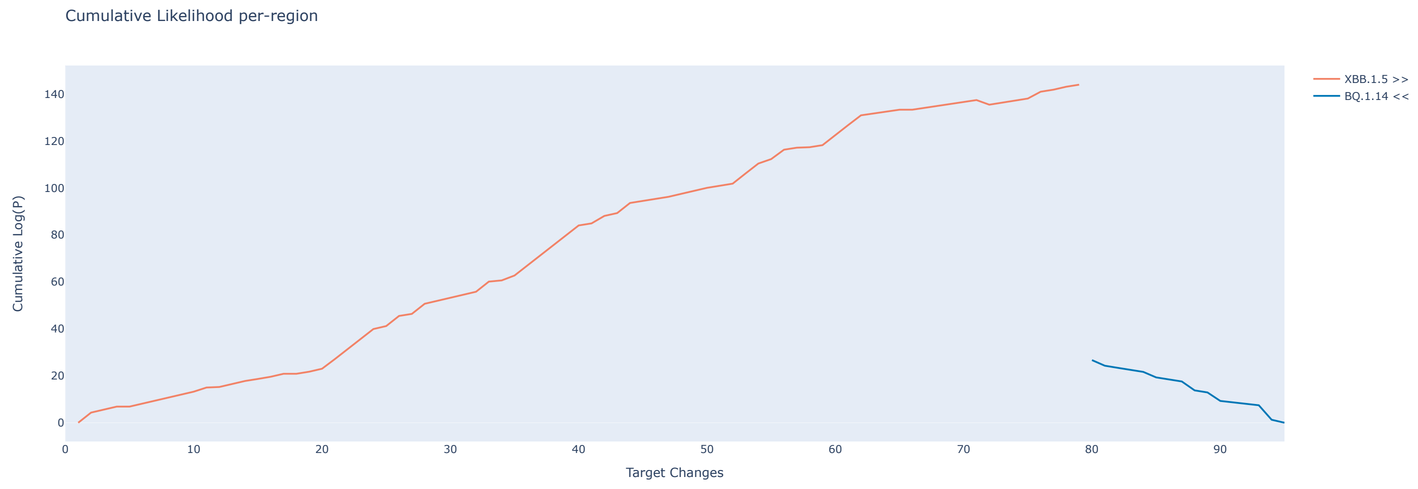


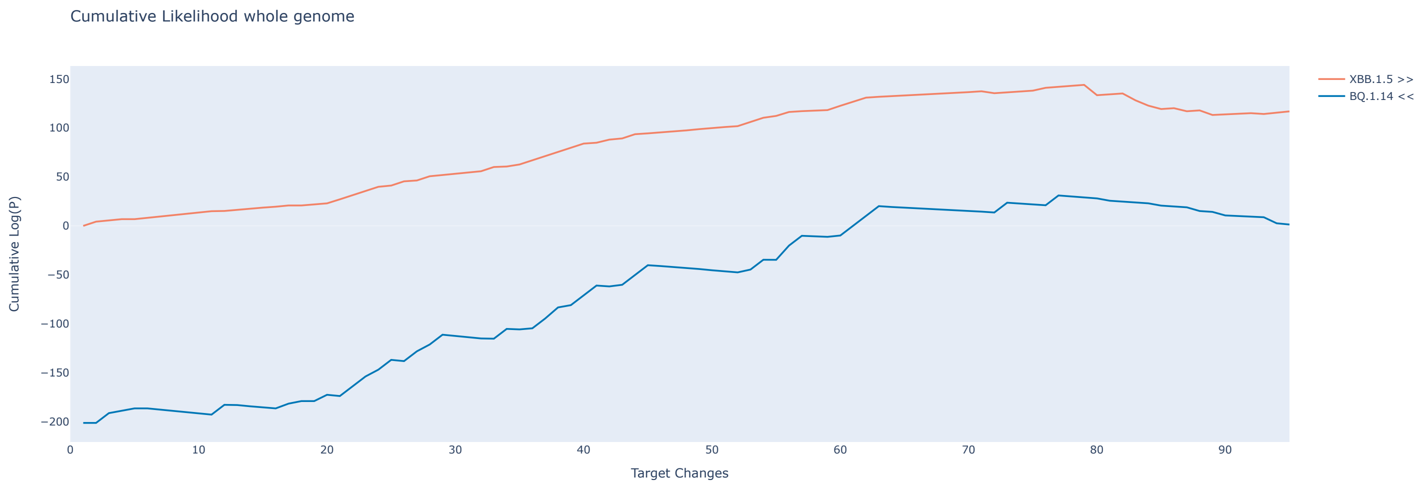


**Target sequence**

, 241_C|T, 405_A|G, 670_T|G, 2790_C|T, 3037_C|T, 4184_G|A, 4321_C|T, 9344_C|T, 9424_A|G, 9534_C|T, 9866_C|T, 10029_C|T, 10198_C|T, 10447_G|A, 10449_C|A, 11288_11296, 12880_C|T, 14408_C|T, 15451_G|A, 15714_C|T, 15738_C|T, 15939_T|C, 16342_T|C, 17124_T|C, 17410_C|T, 17859_T|C, 18163_A|G, 19326_A|G, 19955_C|T, 20055_A|G, 21618_C|T, 21633_21641, 21810_T|C, 21987_G|A, 21992_21994, 22000_C|A, 22109_C|G, 22200_T|A, 22317_G|T, 22577_G|C, 22578_G|A, 22599_G|C, 22612_T|C, 22664_C|A, 22674_C|T, 22679_T|C, 22686_C|T, 22688_A|G, 22775_G|A, 22786_A|C, 22813_G|T, 22882_T|G, 22895_G|C, 22896_T|C, 22898_G|A, 22942_T|G, 22992_G|A, 22995_C|A, 23013_A|C, 23018_T|C, 23019_T|C, 23031_T|C, 23055_A|G, 23063_A|T, 23075_T|C, 23403_A|G, 23525_C|T, 23599_T|G, 23604_C|A, 23854_C|A, 23948_G|T, 24130_C|T, 24424_A|T, 24469_T|A, 25000_C|T, 25416_C|T, 25584_C|T, 26060_C|T, 26270_C|T, 26529_G|A, 26577_C|G, 26709_G|A, 27807_C|T, 27889_C|T, 28271_A|T, 28311_C|T, 28312_C|T, 28362_28370, 28681_G|T, 28881_G|A, 28882_G|A, 28883_G|C, 29260_G|A, 29510_A|C, 29734_29759

## Case 22 (1BP mid): XJ test: OK

Target: (75%) 24 samples
GT: BA.1* + BA.2*
BC: BA.1.24 + BA.2.1
Direction L1: <<
Alt. candidates: [BA.1], [BA.2, BA.2.11]
Model 1BP/2BP comparison:
Rec. model vs L1: 9.39e−259
Flags: Model_2BP_Bad_L1_opp

Number of changes: 65
GT BR: 11−14
BC BR: 11−12
Initial region span: 1−11,12−65
−
Rec. model vs L2: 1.22e−182

GT BR coord: 13199 − 17401
BC BR coord: 13215 − 13216
Gap history (edge excluded):

Rank L1 L2: 8 5

## BA.1.24 >>

| | num_seq | t_ch_MAX | max_CL | CL@BC_t_ch_MAX | aic | PV | PV_OK | t_ch_MAX_OK | phyl_OK |
|---|---|---|---|---|---|---|---|---|---|
| **BA.1.24** | 96 | 11 | 14.920857 | NaN | NaN | NaN | * | * | * |
| **BA.1.6** | 60 | 11 | 14.920857 | 14.920857 | -7.841714 | 1.000000 | * | * | |
| **BA.1.12** | 635 | 11 | 14.919281 | 14.919281 | -7.838562 | 1.000000 | * | * | |
| **BA.1.9** | 186 | 11 | 14.915466 | 14.915466 | -7.830933 | 0.995012 | * | * | |
| **BA.1.10** | 459 | 11 | 14.914309 | 14.914309 | -7.828619 | 0.995012 | * | * | |
| **BA.1.7** | 141 | 11 | 14.913740 | 14.913740 | -7.827480 | 0.995012 | * | * | |
| **BA.1.1.4** | 767 | 11 | 14.907784 | 14.907784 | -7.815567 | 0.990050 | * | * | |
| **BA.1** | 100089 | 11 | 14.901478 | 14.901478 | -7.802956 | 0.980199 | * | * | * |
| **BA.1.21.1** | 51 | 11 | 14.901055 | 14.901055 | -7.802109 | 0.980199 | * | * | |
| **BA.1.1.1** | 8304 | 11 | 14.892600 | 14.892600 | -7.785200 | 0.975310 | * | * | |

## BA.2.1 <<

| | num_seq | t_ch_MAX | max_CL | CL@BC_t_ch_MAX | aic | PV | PV_OK | t_ch_MAX_OK | phyl_OK |
|---|---|---|---|---|---|---|---|---|---|
| **BA.2.1** | 7140 | 12 | 52.374239 | NaN | NaN | NaN | * | * | * |
| **BA.2.23** | 8100 | 12 | 52.349110 | 52.349110 | 7.301780 | 0.975310 | * | * | |
| **BA.2.26** | 580 | 12 | 52.230030 | 52.230030 | 7.539940 | 0.865022 | * | * | |
| **BA.2.31** | 453 | 12 | 52.146648 | 52.146648 | 7.706705 | 0.794534 | * | * | |
| **BA.2** | 359165 | 12 | 51.865792 | 51.865792 | 8.268415 | 0.600496 | * | * | * |
| **BA.2.29** | 102 | 12 | 51.847872 | 51.847872 | 8.304257 | 0.591555 | * | * | |
| **BA.2.27** | 19 | 12 | 51.807281 | 51.807281 | 8.385438 | 0.565525 | * | * | |
| **BA.2.5** | 473 | 12 | 51.592229 | 51.592229 | 8.815542 | 0.456120 | * | * | |
| **BA.2.10** | 12936 | 14 | 50.942678 | 48.133114 | 15.733772 | 0.014408 | * | | * |
| **BA.2.11** | 22 | 12 | 50.704783 | 50.704783 | 10.590435 | 0.188247 | * | * | * |

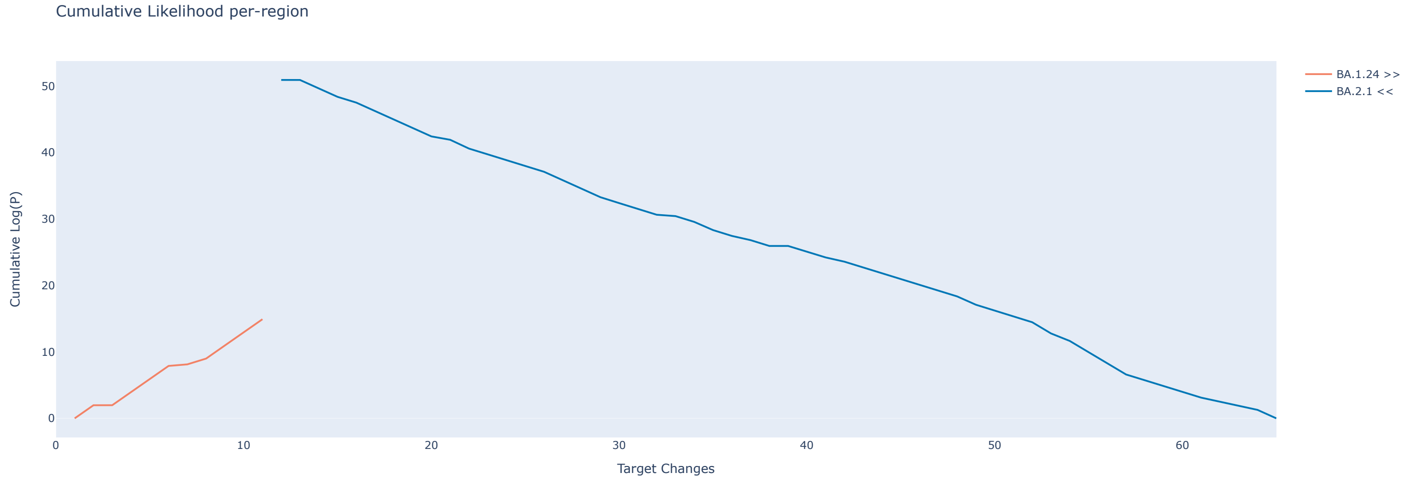


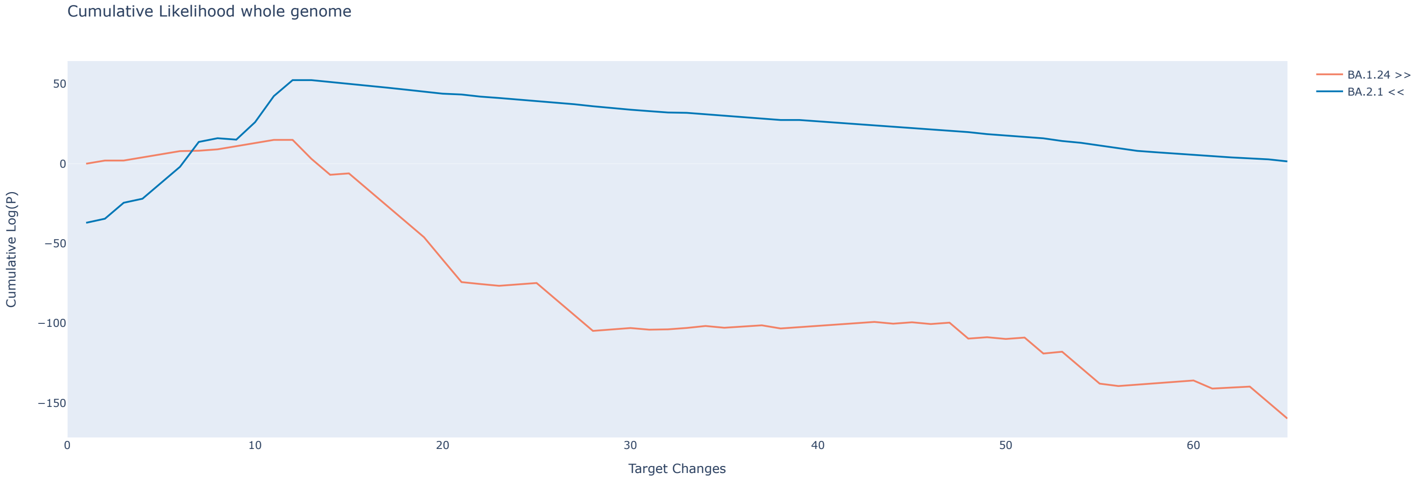


**Target sequence**

, 241_C|T, 2832_A|G, 3037_C|T, 5386_T|G, 6513_6515, 8393_G|A, 10029_C|T, 10449_C|A, 11285_11293, 11537_A|G, 13195_T|C, 14408_C|T, 15714_C|T, 17410_C|T, 18163_A|G, 19955_C|T, 20055_A|G, 21618_C|T, 21633_21641, 21987_G|A, 22200_T|G, 22578_G|A, 22674_C|T, 22679_T|C, 22686_C|T, 22688_A|G, 22775_G|A, 22786_A|C, 22813_G|T, 22882_T|G, 22992_G|A, 22995_C|A, 23013_A|C, 23040_A|G, 23055_A|G, 23063_A|T, 23075_T|C, 23403_A|G, 23525_C|T, 23599_T|G, 23604_C|A, 23854_C|A, 23948_G|T, 24424_A|T, 24469_T|A, 25000_C|T, 25584_C|T, 26060_C|T, 26270_C|T, 26577_C|G, 26709_G|A, 26858_C|T, 27259_A|C, 27382_G|C, 27383_A|T, 27384_T|C, 27807_C|T, 28271_A|T, 28311_C|T, 28362_28370, 28881_G|A, 28882_G|A, 28883_G|C, 29510_A|C, 29734_29759

## Case 23 (1BP mid): XM          test: KO

Target: (75%) 145 samples
Number of changes: 62
GT: BA.1.1* + BA.2*
GT BR: 13–16
GT BR coord: 17409 – 19996
Rank L1 L2: 11 2
BC: BA.1.24 + BA.2.27
BC BR: 13–14
BC BR coord: 17426 – 17427
Direction L1: <<
Initial region span: 1–13,14–62
Gap history (edge excluded):
Alt. candidates: [BA.1], [BA.2]
Model 1BP/2BP comparison: –
Rec. model vs L1: 1.96e–227
Rec. model vs L2: 4.56e–200
Flags: Model_2BP_Bad_L1_opp

## BA.1.24 >>

| | num_seq | t_ch_MAX | max_CL | CL@BC_t_ch_MAX | aic | PV | PV_OK | t_ch_MAX_OK | phyl_OK |
|---|---|---|---|---|---|---|---|---|---|
| **BA.1.24** | 96 | 13 | 16.788197 | NaN | NaN | NaN | * | * | * |
| **BA.1.6** | 60 | 13 | 16.788197 | 16.788197 | 0.423606 | 1.000000 | * | * | |
| **BA.1.12** | 635 | 13 | 16.786621 | 16.786621 | 0.426758 | 0.995012 | * | * | |
| **BA.1.9** | 186 | 13 | 16.782806 | 16.782806 | 0.434388 | 0.995012 | * | * | |
| **BA.1.10** | 459 | 13 | 16.781649 | 8.085065 | 17.829871 | 0.000166 | * | * | |
| **BA.1.1.4** | 767 | 13 | 16.775123 | 10.510386 | 12.979229 | 0.001873 | * | * | |
| **BA.1.21.1** | 51 | 13 | 16.768394 | 5.521764 | 22.956472 | 0.000013 | * | * | |
| **BA.1** | 100089 | 13 | 16.768108 | 16.768108 | 0.463784 | 0.980199 | * | * | * |
| **BA.1.1.1** | 8304 | 13 | 16.758976 | 10.655245 | 12.689509 | 0.002166 | * | * | |
| **BA.1.19** | 691 | 13 | 16.753279 | 16.753279 | 0.493442 | 0.965605 | * | * | |

## BA.2.27 <<

| | num_seq | t_ch_MAX | max_CL | CL@BC_t_ch_MAX | aic | PV | PV_OK | t_ch_MAX_OK | phyl_OK |
|---|---|---|---|---|---|---|---|---|---|
| **BA.2.27** | 19 | 14 | 45.792740 | NaN | NaN | NaN | * | * | * |
| **BA.2** | 359165 | 14 | 45.638829 | 45.638829 | 14.722341 | 0.856415 | * | * | * |
| **BA.2.11** | 22 | 14 | 44.836845 | 44.836845 | 16.326310 | 0.382893 | * | * | |
| **BA.2.40** | 27 | 15 | 44.574726 | 38.734725 | 28.530551 | 0.000859 | * | * | |
| **BA.2.23** | 8100 | 14 | 44.470849 | 44.470849 | 17.058301 | 0.265803 | * | * | |
| **BA.2.1** | 7140 | 14 | 44.452858 | 44.452858 | 17.094284 | 0.261846 | * | * | |
| **BA.2.10** | 12936 | 14 | 44.393090 | 44.393090 | 17.213820 | 0.246597 | * | * | |
| **BA.2.26** | 580 | 14 | 44.322870 | 44.322870 | 17.354261 | 0.229925 | * | * | |
| **BA.2.31** | 453 | 14 | 44.291531 | 44.291531 | 17.416937 | 0.222017 | * | * | |
| **BA.2.5** | 473 | 14 | 44.052634 | 44.052634 | 17.894732 | 0.175520 | * | * | |

Cumulative Likelihood per-region

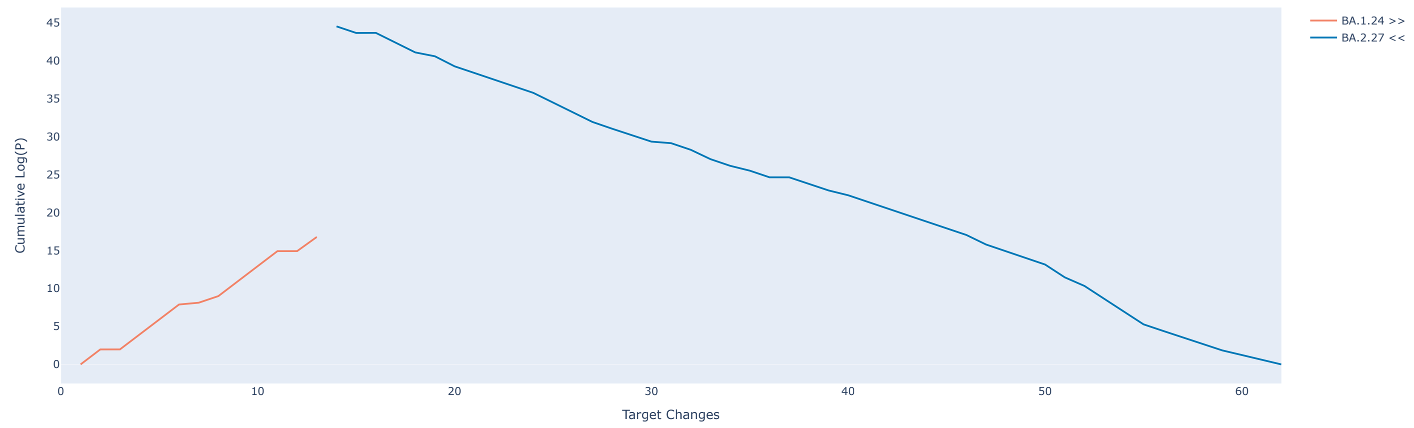


Cumulative Likelihood whole genome

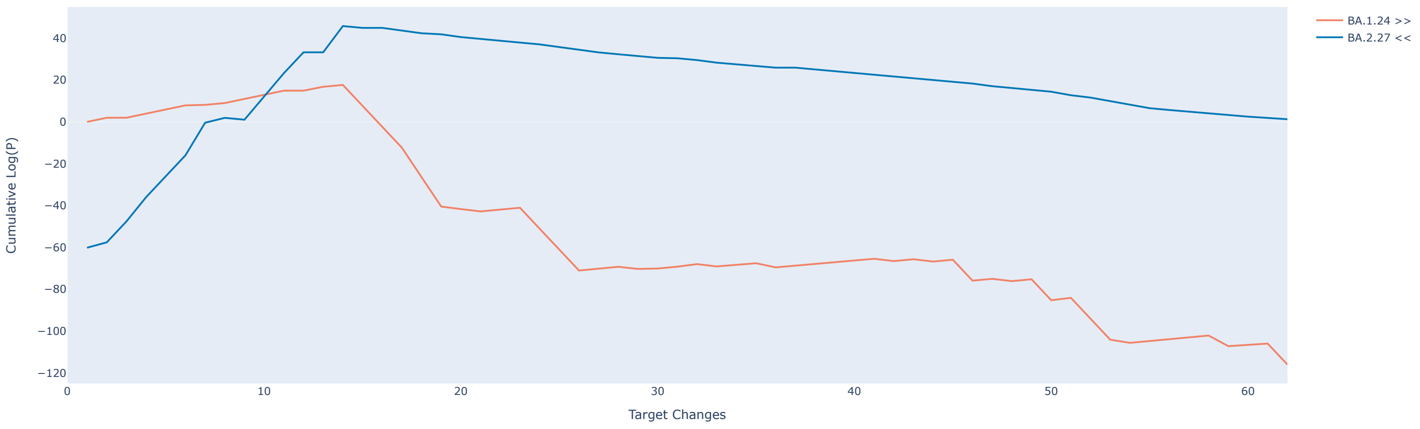


**Target sequence**

, 241_C|T, 2832_A|G, 3037_C|T, 5386_T|G, 6513_6515, 8393_G|A, 10029_C|T, 10449_C|A, 11285_11293, 11537_A|G, 13195_T|C, 14408_C|T, 15240_C|T, 18163_A|G, 19955_C|T, 21618_C|T, 21633_21641, 21987_G|A, 22200_T|G, 22578_G|A, 22674_C|T, 22679_T|C, 22686_C|T, 22688_A|G, 22775_G|A, 22786_A|C, 22813_G|T, 22882_T|G, 22992_G|A, 22995_C|A, 23013_A|C, 23040_A|G, 23055_A|G, 23063_A|T, 23075_T|C, 23403_A|G, 23525_C|T, 23599_T|G, 23604_C|A, 23854_C|A, 23948_G|T, 24424_A|T, 24469_T|A, 25000_C|T, 25584_C|T, 26060_C|T, 26270_C|T, 26577_C|G, 26709_G|A, 26858_C|T, 27259_A|C, 27382_G|C, 27383_A|T, 27384_T|C, 27807_C|T, 28271_A|T, 28311_C|T, 28362_28370, 28881_G|A, 28882_G|A, 28883_G|C, 29510_A|C

## Case 24 (1BP mid): XV    test: OK

Target: (75%) 25 samples    Number of changes: 66
GT: BA.1* + BA.2*    GT BR: 12–14    GT BR coord: 13195 – 15714    Rank L1 L2: 3 3
BC: BA.1.14 + BA.2.52    BC BR: 12–13    BC BR coord: 13215 – 13216
Direction L1: <<    Initial region span: 1–11,13–66    Gap history (edge excluded): 11–13
Alt. candidates: [BA.1, BA.1.1], [BA.2]
Model 1BP/2BP comparison:    –
Rec. model vs L1: 1.08e–241    Rec. model vs L2: 5.14e–165
Flags: Model_2BP_Bad_L1_opp

## BA.1.14 >>

| | num_seq | t_ch_MAX | max_CL | CL@BC_t_ch_MAX | aic | PV | PV_OK | t_ch_MAX_OK | phyl_OK |
|---|---|---|---|---|---|---|---|---|---|
| **BA.1.14** | 1016 | 11 | 13.974118 | NaN | NaN | NaN | * | * | * |
| **BA.1.1.1** | 8304 | 12 | 13.836143 | 13.836143 | 10.327715 | 135.639414 | * | * | |
| **BA.1** | 100089 | 12 | 13.658283 | 13.658283 | 10.683433 | 113.863459 | * | * | * |
| **BA.1.1** | 262925 | 12 | 12.520043 | 12.520043 | 12.959913 | 36.415700 | * | * | * |
| **BA.1.1.15** | 5038 | 12 | 11.804349 | 11.804349 | 14.391302 | 17.814273 | * | * | |
| **BA.1.17** | 6954 | 12 | 10.343131 | 10.343131 | 17.313737 | 4.137120 | * | * | |
| **BA.1.15** | 46393 | 12 | 9.828735 | 9.828735 | 18.342531 | 2.471932 | * | * | |
| **BA.1.18** | 10104 | 12 | 8.778298 | 8.778298 | 20.443403 | 0.865022 | * | * | |
| **BA.1.1.18** | 22283 | 12 | 8.256680 | 8.256680 | 21.486640 | 0.511709 | * | * | |
| **BA.1.1.2** | 2970 | 9 | 7.099268 | 5.818897 | 26.362206 | 0.044825 | * | | |

## BA.2.52 <<

| | num_seq | t_ch_MAX | max_CL | CL@BC_t_ch_MAX | aic | PV | PV_OK | t_ch_MAX_OK | phyl_OK |
|---|---|---|---|---|---|---|---|---|---|
| **BA.2.52** | 263 | 13 | 53.623632 | NaN | NaN | NaN | * | * | * |
| **BA.2.25** | 97 | 13 | 53.279797 | 53.279797 | 11.440407 | 0.708220 | * | * | |
| **BA.2** | 359165 | 13 | 52.537695 | 52.537695 | 12.924609 | 0.337902 | * | * | * |
| **BA.2.7** | 1628 | 13 | 52.054213 | 52.054213 | 13.891574 | 0.208045 | * | * | |
| **BA.2.22** | 2689 | 16 | 49.863956 | 46.786329 | 24.427342 | 0.001070 | * | | |
| **BA.2.9.5** | 109 | 13 | 49.739985 | 49.739985 | 18.520030 | 0.020548 | * | * | |
| **BA.2.9** | 61803 | 13 | 49.382715 | 49.382715 | 19.234571 | 0.014408 | * | * | |
| **BA.2.9.2** | 355 | 13 | 47.780714 | 47.780714 | 22.438572 | 0.002894 | * | * | |
| **BA.2.36** | 1320 | 13 | 46.550714 | 46.550714 | 24.898571 | 0.000846 | * | * | |
| **BA.2.9.6** | 98 | 13 | 46.536065 | 46.536065 | 24.927869 | 0.000833 | * | * | |

Cumulative Likelihood per-region

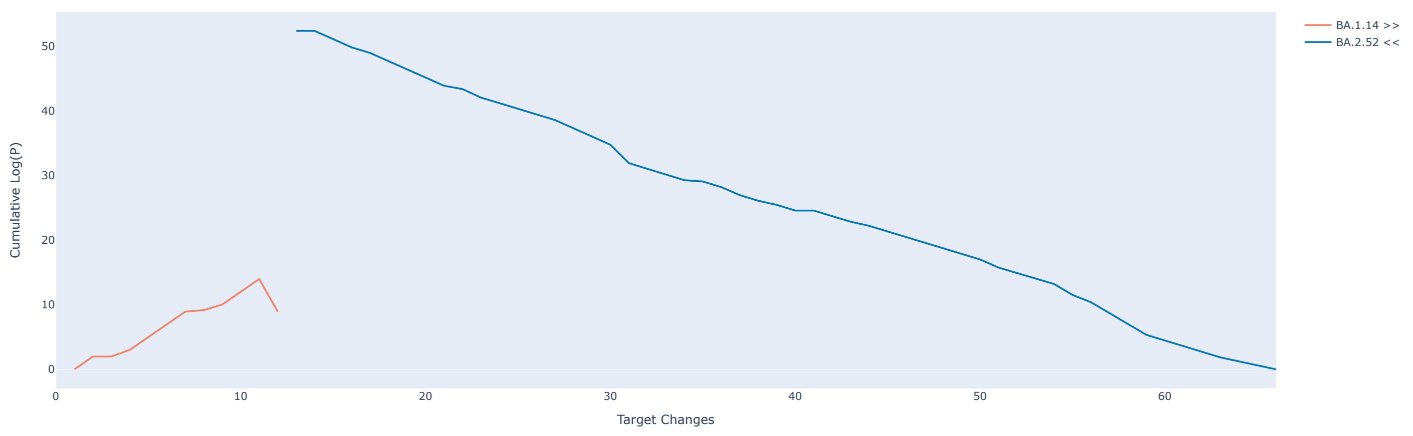


Cumulative Likelihood whole genome

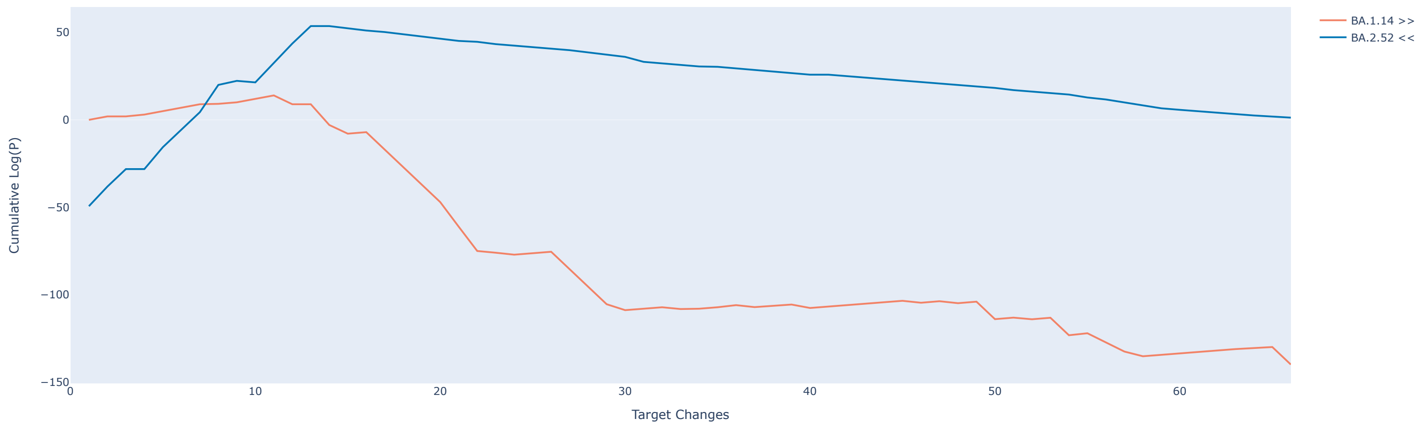


**Target sequence**

, 241_C|T, 2832_A|G, 3037_C|T, 3583_C|T, 5386_T|G, 6513_6515, 8393_G|A, 10029_C|T, 10449_C|A, 11285_11293, 11537_A|G, 13195_T|C, 14408_C|T, 15714_C|T, 17410_C|T, 18163_A|G, 19955_C|T, 20055_A|G, 21618_C|T, 21633_21641, 21987_G|A, 22200_T|G, 22578_G|A, 22674_C|T, 22679_T|C, 22686_C|T, 22688_A|G, 22775_G|A, 22786_A|C, 22792_C|T, 22813_G|T, 22882_T|G, 22992_G|A, 22995_C|A, 23013_A|C, 23040_A|G, 23055_A|G, 23063_A|T, 23075_T|C, 23403_A|G, 23525_C|T, 23599_T|G, 23604_C|A, 23854_C|A, 23948_G|T, 24424_A|T, 24469_T|A, 25000_C|T, 25584_C|T, 26060_C|T, 26270_C|T, 26577_C|G, 26709_G|A, 26858_C|T, 27259_A|C, 27382_G|C, 27383_A|T, 27384_T|C, 27807_C|T, 28271_A|T, 28311_C|T, 28362_28370, 28881_G|A, 28882_G|A, 28883_G|C, 29510_A|C

**Case 25 (1BP mid): XY** **test: OK**

Target: (75%) 62 samples    Number of changes: 74
GT: BA.1* + BA.2*    GT BR: 14-16    GT BR coord: 11539 - 12880    Rank L1 L2: 11 1
BC: BA.1.1 + BA.2    BC BR: 14-15    BC BR coord: 11561 - 11562
Direction L1: <<    Initial region span: 1-14,16-74    Gap history (edge excluded): 14-16
Alt. candidates: [], [BA.2.9]
Model 1BP/2BP comparison: -
Rec. model vs L1: 4.16e-126    Rec. model vs L2: 8.66e-77
Flags: Model_2BP_Bad_L1_opp

## BA.1.1 >>

| | num_seq | t_ch_MAX | max_CL | CL@BC_t_ch_MAX | aic | PV | PV_OK | t_ch_MAX_OK | phyl_OK |
|---|---|---|---|---|---|---|---|---|---|
| **BA.1.1** | 262925 | 14 | 17.233406 | NaN | NaN | NaN | * | * | * |
| **BA.1.1.9** | 172 | 8 | 0.444016 | -14.501106 | 59.002212 | 1.650687e-14 | | | * |
| **BA.1.1.13** | 3393 | 8 | 0.441953 | -4.545530 | 39.091059 | 3.475891e-10 | | | * |
| **BA.1.1.14** | 5810 | 8 | 0.437642 | -14.518005 | 59.036010 | 1.618001e-14 | | | * |
| **BA.1.1.15** | 5038 | 8 | 0.437454 | -14.509059 | 59.018117 | 1.634262e-14 | | | * |
| **BA.1.1.16** | 594 | 8 | 0.432205 | -14.517975 | 59.035951 | 1.618001e-14 | | | * |
| **BA.1.1.2** | 2970 | 8 | 0.430179 | -21.719198 | 73.438396 | 1.207976e-17 | | | * |
| **BA.1.1.10** | 592 | 8 | 0.425356 | -0.894927 | 31.789854 | 1.337338e-08 | | | * |

## BA.2 <<

| | num_seq | t_ch_MAX | max_CL | CL@BC_t_ch_MAX | aic | PV | PV_OK | t_ch_MAX_OK | phyl_OK |
|---|---|---|---|---|---|---|---|---|---|
| **BA.2** | 359165 | 16 | 53.195199 | NaN | NaN | NaN | * | * | * |
| **BA.2.9** | 61803 | 15 | 48.446735 | 48.446735 | 43.106531 | 1.477232e-02 | * | * | * |
| **BA.2.7** | 1628 | 29 | 44.201251 | 25.828262 | 88.343477 | 2.227815e-12 | | | * |
| **BA.2.48** | 852 | 29 | 39.660966 | -2.171271 | 144.342542 | 1.540400e-24 | | | * |
| **BA.2.24** | 82 | 37 | 36.580489 | 6.329656 | 127.340688 | 7.570711e-21 | | | * |
| **BA.2.22** | 2689 | 29 | 34.599331 | 3.497857 | 133.004286 | 4.467692e-22 | | | * |
| **BA.2.52** | 263 | 29 | 34.378391 | 15.965792 | 108.068416 | 1.157615e-16 | | | * |
| **BA.2.36** | 1320 | 29 | 34.216589 | 19.076257 | 101.847485 | 2.595493e-15 | | | * |
| **BA.2.1** | 7140 | 37 | 34.210004 | 19.067869 | 101.864261 | 2.582548e-15 | | | * |
| **BA.2.71** | 139 | 29 | 34.153913 | 19.120906 | 101.758189 | 2.714958e-15 | | | * |

Cumulative Likelihood per-region

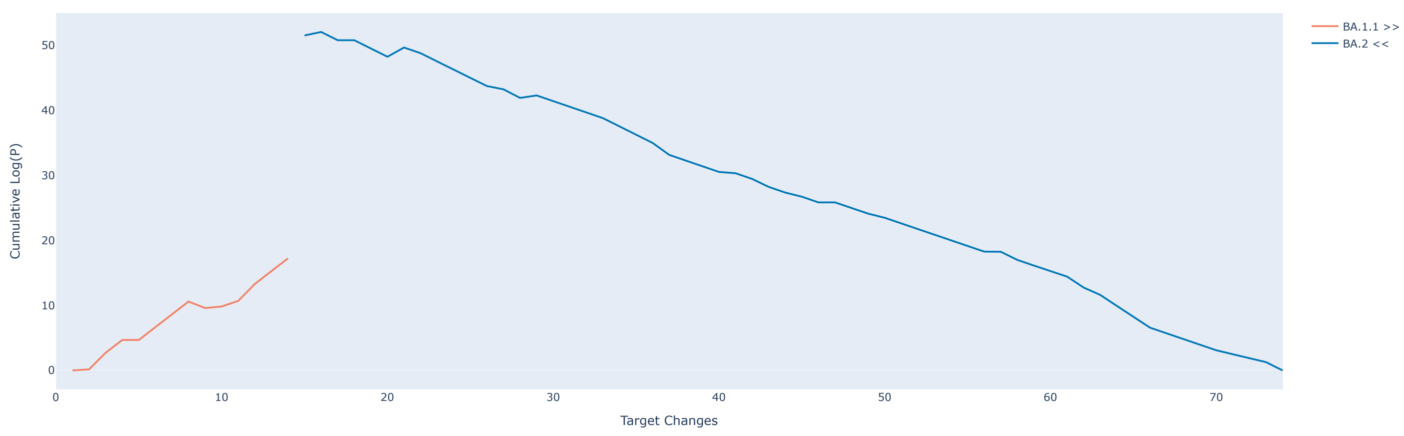


Cumulative Likelihood whole genome

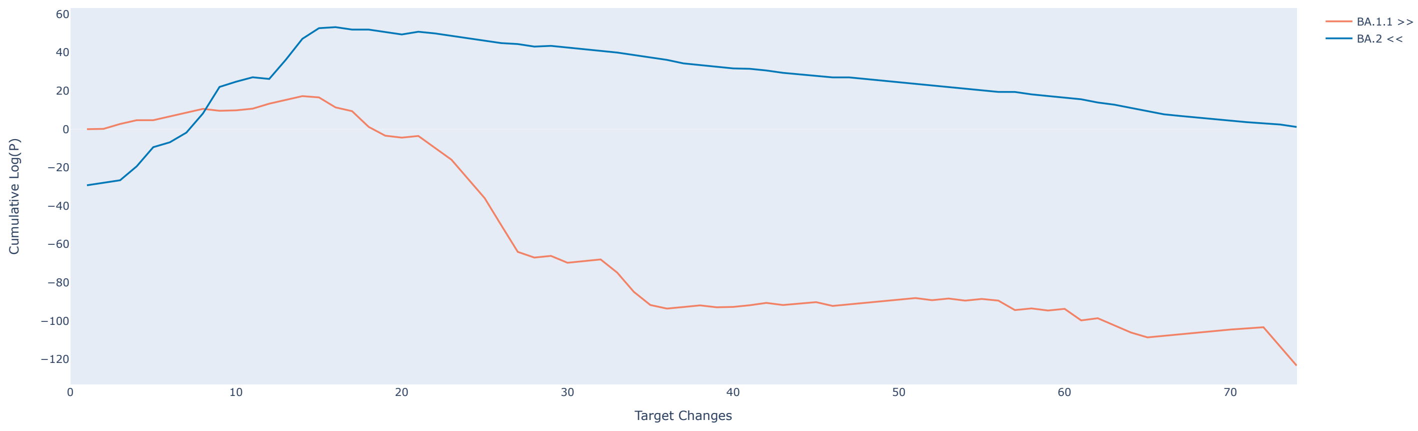


**Target sequence**

, 241_C|T, 1585_A|G, 2470_C|T, 2832_A|G, 3037_C|T, 5386_T|G, 6513_6515, 8393_G|A, 9611_C|T, 10029_C|T, 10449_C|A, 11049_T|C, 11285_11293, 11537_A|G, 12085_C|T, 12880_C|T, 14408_C|T, 15714_C|T, 17410_C|T, 17443_G|T, 18163_A|G, 19955_C|T, 20055_A|G, 21618_C|T, 21633_21641, 21987_G|A, 22200_T|G, 22351_A|G, 22578_G|A, 22674_C|T, 22679_T|C, 22686_C|T, 22688_A|G, 22775_G|A, 22786_A|C, 22792_C|T, 22813_G|T, 22882_T|G, 22992_G|A, 22995_C|A, 23013_A|C, 23040_A|G, 23055_A|G, 23063_A|T, 23075_T|C, 23403_A|G, 23525_C|T, 23599_T|G, 23604_C|A, 23854_C|A, 23948_G|T, 24424_A|T, 24469_T|A, 25000_C|T, 25584_C|T, 26058_C|T, 26060_C|T, 26270_C|T, 26577_C|G, 26709_G|A, 26858_C|T, 27259_A|C, 27382_G|C, 27383_A|T, 27384_T|C, 27807_C|T, 28271_A|T, 28311_C|T, 28362_28370, 28881_G|A, 28882_G|A, 28883_G|C, 29510_A|C, 29734_29759

## Case 26 (1BP mid): XZ test: OK

Target: (75%) 140 samples Number of changes: 66
GT: BA.2* + BA.1* GT BR: 54–55 GT BR coord: 26061 – 26251 Rank_L1_L2: 2 11
BC: BA.2.34 + BA.1.23 BC BR: 55–56 BC BR coord: 26274 – 26275
Direction L1: >> Initial region span: 1–55,56–66 Gap history (edge excluded):
Alt. candidates: [BA.2], []
Model 1BP/2BP comparison: –
Rec. model vs L1: 8.68e–70 Rec. model vs L2: 0.00e+00
Flags: Model_2BP_Bad_L1_opp

## BA.2.34 >>

| | num_seq | t_ch_MAX | max_CL | CL@BC_t_ch_MAX | aic | PV | PV_OK | t_ch_MAX_OK | phyl_OK |
|---|---|---|---|---|---|---|---|---|---|
| **BA.2.34** | 44 | 55 | 54.092869 | NaN | NaN | NaN | * | * | * |
| **BA.2** | 359165 | 55 | 52.934821 | 52.934821 | 20.130358 | 0.313486 | * | * | * |
| **BA.2.9** | 61803 | 53 | 51.713635 | 49.855732 | 26.288536 | 0.014408 | * | | |
| **BA.2.6** | 327 | 52 | 50.842282 | 45.930833 | 34.138334 | 0.000284 | * | | |
| **BA.2.10.2** | 42 | 55 | 48.085272 | 48.085272 | 29.829457 | 0.002454 | * | * | |
| **BA.2.7** | 1628 | 55 | 47.675167 | 47.675167 | 30.649665 | 0.001629 | * | * | |
| **BA.2.49** | 212 | 55 | 47.524977 | 47.524977 | 30.950046 | 0.001402 | * | * | |
| **BA.2.36** | 1320 | 55 | 46.968571 | 46.968571 | 32.062857 | 0.000805 | * | * | |
| **BA.2.51** | 234 | 47 | 46.524675 | 46.507550 | 32.984900 | 0.000508 | * | | |
| **BA.2.14** | 912 | 55 | 45.761882 | 45.761882 | 34.476236 | 0.000240 | * | * | |

## BA.1.23 <<

| | num_seq | t_ch_MAX | max_CL | CL@BC_t_ch_MAX | aic | PV | PV_OK | t_ch_MAX_OK | phyl_OK |
|---|---|---|---|---|---|---|---|---|---|
| **BA.1.23** | 12 | 56 | 10.223988 | NaN | NaN | NaN | * | * | * |
| **BA.1.5** | 274 | 56 | 10.223988 | 10.223988 | 1.552024 | 1.000000 | * | * | |
| **BA.1.1.12** | 1943 | 56 | 10.219352 | 10.219352 | 1.561295 | 0.995012 | * | * | |
| **BC.1** | 209 | 56 | 10.219192 | 10.219192 | 1.561616 | 0.995012 | * | * | |
| **BA.1.9** | 186 | 56 | 10.213207 | 10.213207 | 1.573587 | 0.990050 | * | * | |
| **BA.1.1.13** | 3393 | 56 | 10.212778 | 10.212778 | 1.574444 | 0.990050 | * | * | |
| **BA.1.1.17** | 191 | 56 | 10.208157 | 10.208157 | 1.583687 | 0.985112 | * | * | |
| **BA.1.10** | 459 | 56 | 10.202140 | 10.202140 | 1.595720 | 0.975310 | * | * | |
| **BD.1** | 2101 | 56 | 10.197169 | 10.197169 | 1.605662 | 0.970446 | * | * | |
| **BA.1.1.9** | 172 | 56 | 10.194627 | 10.194627 | 1.610746 | 0.970446 | * | * | |

Cumulative Likelihood per-region

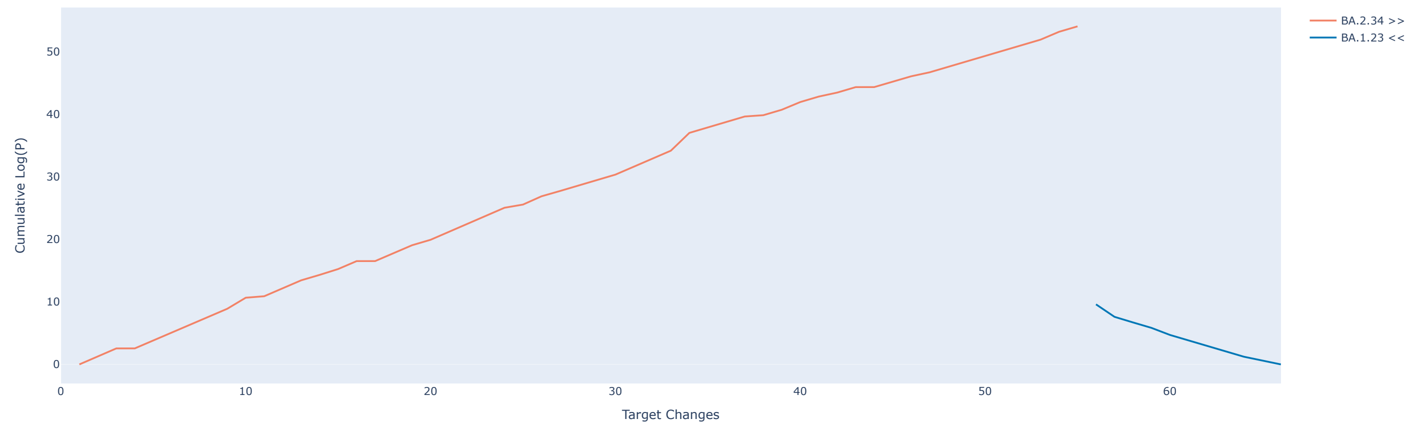


Cumulative Likelihood whole genome

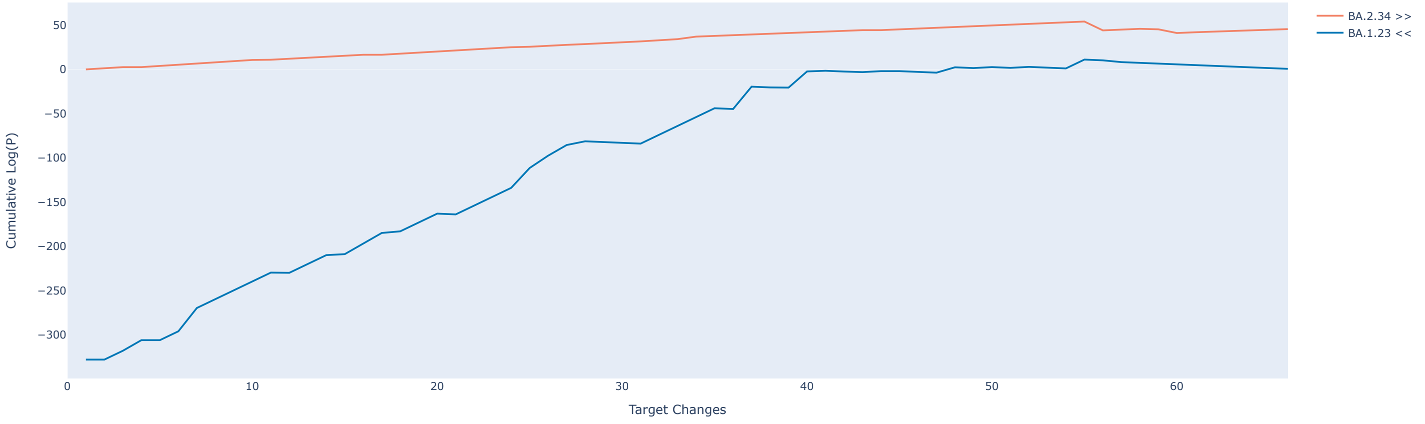


**Target sequence**

, 241_C|T, 670_T|G, 2790_C|T, 3037_C|T, 4184_G|A, 4321_C|T, 9344_C|T, 9424_A|G, 9534_C|T, 9866_C|T, 10029_C|T, 10198_C|T, 10447_G|A, 10449_C|A, 11288_11296, 12880_C|T, 14408_C|T, 15714_C|T, 17410_C|T, 18163_A|G, 19955_C|T, 20055_A|G, 21618_C|T, 21633_21641, 21987_G|A, 22200_T|G, 22578_G|A, 22674_C|T, 22679_T|C, 22686_C|T, 22688_A|G, 22775_G|A, 22786_A|C, 22792_C|T, 22813_G|T, 22882_T|G, 22992_G|A, 22995_C|A, 23013_A|C, 23040_A|G, 23055_A|G, 23063_A|T, 23075_T|C, 23403_A|G, 23525_C|T, 23599_T|G, 23604_C|A, 23854_C|A, 23948_G|T, 24424_A|T, 24469_T|A, 25000_C|T, 25584_C|T, 26060_C|T, 26270_C|T, 26530_A|G, 26577_C|G, 26709_G|A, 27259_A|C, 27807_C|T, 28271_A|T, 28311_C|T, 28362_28370, 28881_G|A, 28882_G|A, 28883_G|C

## Case 27 (1BP 5'): XAA test: OK

Target: (75%) 52 samples Number of changes: 73
GT: BA.1* + BA.2* GT BR: 7–8 GT BR coord: 8938 – 9344 Rank L1 L2: 3 1
BC: BA.1.20 + BA.2 BC BR: 7–8 BC BR coord: 8250 – 8251
Direction L1: << Initial region span: 1–6,8–73 Gap history (edge excluded): 6–8
Alt. candidates: [BA.1], []
Model 1BP/2BP comparison: –
Rec. model vs L1: 2.65e−216 Rec. model vs L2: 9.03e−37
Flags: Model_2BP_Bad_L1_opp

## BA.1.20 >>

| | num_seq | t_ch_MAX | max_CL | CL@BC_t_ch_MAX | aic | PV | PV_OK | t_ch_MAX_OK | phyl_OK |
|---|---|---|---|---|---|---|---|---|---|
| **BA.1.20** | 10512 | 6 | 5.954697 | NaN | NaN | NaN | * | * | * |
| **BA.1.15** | 46393 | 6 | 4.467433 | 4.341834 | 9.316332 | 0.204948 | * | * | |
| **BA.1** | 100089 | 6 | 4.399568 | 3.659208 | 10.681584 | 0.103830 | * | * | * |
| **BA.1.1.18** | 22283 | 6 | 3.348994 | -0.577687 | 19.155375 | 0.001496 | * | * | |
| **BA.1.1** | 262925 | 6 | 1.287444 | 0.274423 | 17.451154 | 0.003518 | * | * | |

## BA.2 <<

| | num_seq | t_ch_MAX | max_CL | CL@BC_t_ch_MAX | aic | PV | PV_OK | t_ch_MAX_OK | phyl_OK |
|---|---|---|---|---|---|---|---|---|---|
| **BA.2** | 359165 | 8 | 61.821121 | NaN | NaN | NaN | * | * | * |
| **BA.2.1** | 7140 | 24 | 48.961671 | 38.740017 | 66.519967 | 9.472909e-11 | | | * |
| **BA.2.23** | 8100 | 24 | 48.936525 | 43.795038 | 56.409924 | 1.485395e-08 | | | * |
| **BA.2.26** | 580 | 24 | 48.819048 | 36.734543 | 70.530913 | 1.275625e-11 | | | * |
| **BA.2.10** | 12936 | 24 | 48.803253 | 39.570584 | 64.858831 | 2.172440e-10 | | | * |
| **BA.2.31** | 453 | 24 | 48.733940 | 36.608171 | 70.783659 | 1.125735e-11 | | | * |
| **BA.2.63** | 46 | 24 | 48.725758 | 34.700366 | 74.599268 | 1.666993e-12 | | | * |
| **BA.2.29** | 102 | 24 | 48.435164 | 43.298123 | 57.403754 | 9.054536e-09 | | | * |
| **BA.2.27** | 19 | 24 | 48.394573 | 43.257532 | 57.484936 | 8.699502e-09 | | | * |
| **BA.2.19** | 114 | 24 | 48.266070 | 25.510582 | 92.978836 | 1.701247e-16 | | | * |

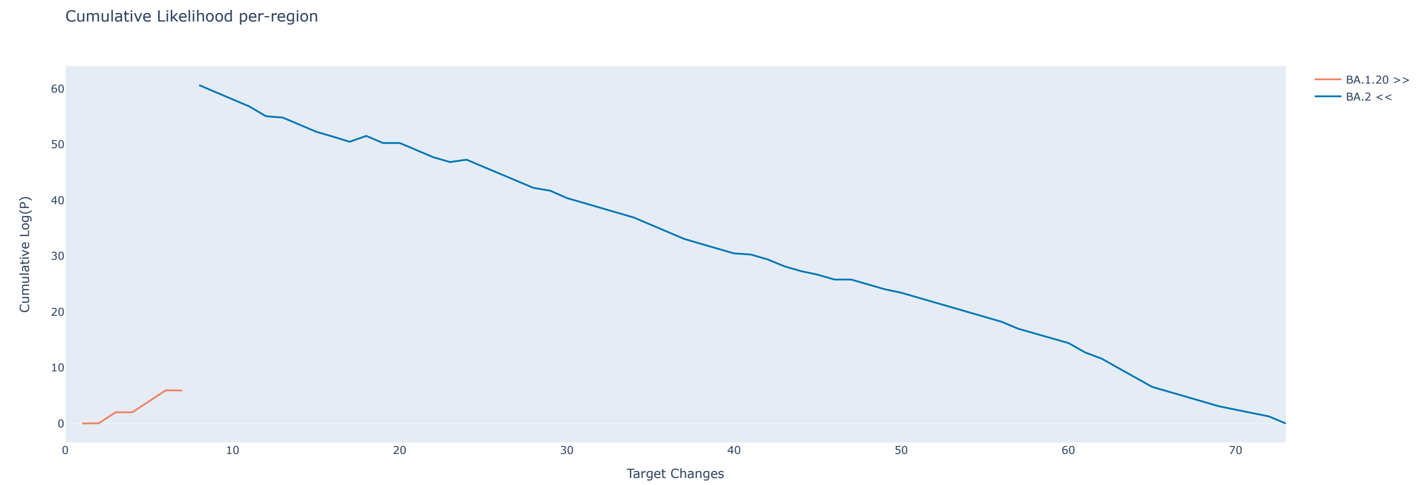


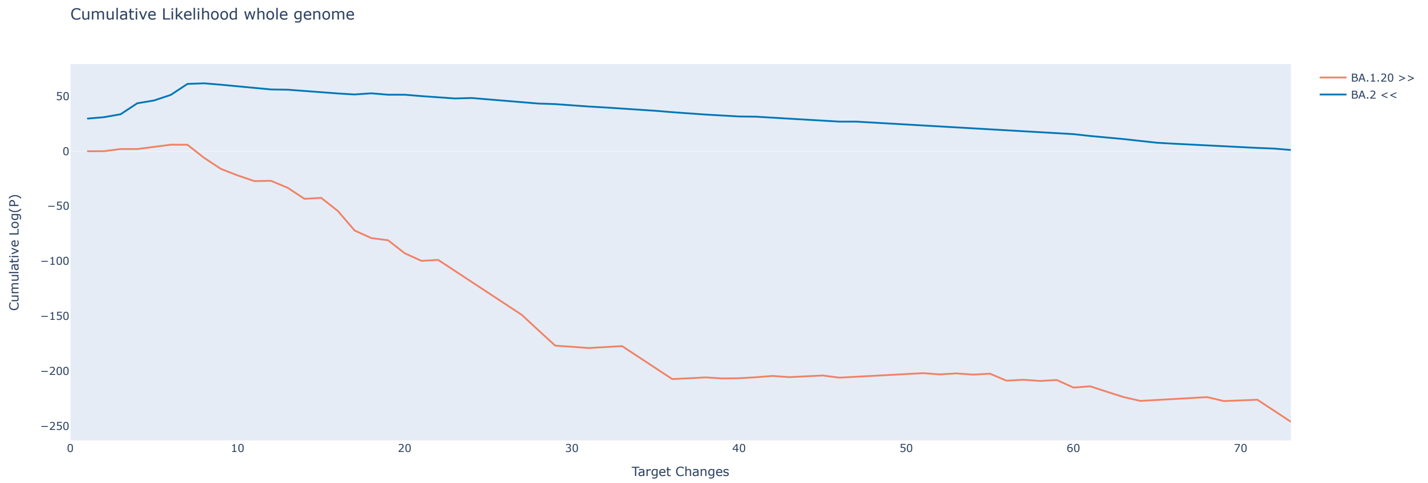


**Target sequence**

, 241_C|T, 1610_A|G, 2832_A|G, 3037_C|T, 5386_T|G, 6513_6515, 8247_C|T, 9344_C|T, 9424_A|G, 9534_C|T, 9866_C|T, 10029_C|T, 10198_C|T, 10447_G|A, 10449_C|A, 11288_11296, 12445_C|T, 12880_C|T, 14408_C|T, 15714_C|T, 17410_C|T, 18163_A|G, 19356_A|G, 19955_C|T, 20055_A|G, 21618_C|T, 21633_21641, 21987_G|A, 22200_T|G, 22578_G|A, 22674_C|T, 22679_T|C, 22686_C|T, 22688_A|G, 22775_G|A, 22786_A|C, 22813_G|T, 22882_T|G, 22992_G|A, 22995_C|A, 23013_A|C, 23040_A|G, 23055_A|G, 23063_A|T, 23075_T|C, 23403_A|G, 23525_C|T, 23599_T|G, 23604_C|A, 23854_C|A, 23948_G|T, 24424_A|T, 24469_T|A, 25000_C|T, 25584_C|T, 26060_C|T, 26270_C|T, 26577_C|G, 26709_G|A, 26858_C|T, 27259_A|C, 27382_G|C, 27383_A|T, 27384_T|C, 27807_C|T, 28271_A|T, 28311_C|T, 28362_28370, 28881_G|A, 28882_G|A, 28883_G|C, 29510_A|C, 29734_29759

## Case 28 (1BP 5'): XAB test: OK

Target: (75%) 88 samples    Number of changes: 67
GT: BA.1* + BA.2*    GT BR: 4-5    GT BR coord: 6515 - 8393    Rank L1 L2: 11 2
BC: BA.1.6 + BA.2.27    BC BR: 4-5    BC BR coord: 5387 - 5388
Direction L1: <<    Initial region span: 1-4,5-67    Gap history (edge excluded):
Alt. candidates: [], [BA.2]
Model 1BP/2BP comparison: -
Rec. model vs L1: 0.00e+00    Rec. model vs L2: 4.30e-61
Flags: Model_2BP_Bad_L1_opp

## BA.1.6 >>

| | num_seq | t_ch_MAX | max_CL | CL@BC_t_ch_MAX | aic | PV | PV_OK | t_ch_MAX_OK | phyl_OK |
|---|---|---|---|---|---|---|---|---|---|
| **BA.1.6** | 60 | 4 | 3.950634 | NaN | NaN | NaN | * | * | * |
| **BA.1.4** | 12 | 4 | 3.950634 | 3.950634 | 0.098731 | 1.000000 | * | * | |
| **BA.1.24** | 96 | 4 | 3.950634 | 3.950634 | 0.098731 | 1.000000 | * | * | |
| **BA.1.23** | 12 | 4 | 3.950634 | 3.950634 | 0.098731 | 1.000000 | * | * | |
| **BA.1.10** | 459 | 4 | 3.950634 | 3.950634 | 0.098731 | 1.000000 | * | * | |
| **BC.1** | 209 | 4 | 3.950634 | 3.950634 | 0.098731 | 1.000000 | * | * | |
| **BA.1.12** | 635 | 4 | 3.949058 | 3.949058 | 0.101884 | 1.000000 | * | * | |
| **BA.1.1.1** | 8304 | 4 | 3.946175 | 3.946175 | 0.107650 | 0.995012 | * | * | |
| **BA.1.8** | 208 | 4 | 3.945815 | 3.945815 | 0.108370 | 0.995012 | * | * | |
| **BA.1.9** | 186 | 4 | 3.945243 | 3.945243 | 0.109513 | 0.995012 | * | * | |

## BA.2.27 <<

| | num_seq | t_ch_MAX | max_CL | CL@BC_t_ch_MAX | aic | PV | PV_OK | t_ch_MAX_OK | phyl_OK |
|---|---|---|---|---|---|---|---|---|---|
| **BA.2.27** | 19 | 5 | 62.350237 | NaN | NaN | NaN | * | * | * |
| **BA.2** | 359165 | 5 | 62.120412 | 62.120412 | 15.759176 | 0.794534 | * | * | * |
| **BA.2.11** | 22 | 5 | 61.136513 | 51.668738 | 36.662524 | 0.000023 | * | * | |
| **BA.2.23** | 8100 | 5 | 60.964123 | 60.964123 | 18.071755 | 0.250324 | * | * | |
| **BA.2.26** | 580 | 5 | 60.857825 | 53.929451 | 32.141097 | 0.000220 | * | * | |
| **BA.2.5** | 473 | 5 | 60.550445 | 60.550445 | 18.899111 | 0.165299 | * | * | |
| **BA.2.29** | 102 | 5 | 60.450150 | 60.450150 | 19.099699 | 0.149569 | * | * | |
| **BA.2.52** | 263 | 5 | 59.378200 | 59.378200 | 21.243599 | 0.051303 | * | * | |
| **BA.2.25** | 97 | 5 | 59.058900 | 59.058900 | 21.882199 | 0.037254 | * | * | |
| **BA.2.57** | 14 | 5 | 58.018463 | 48.768624 | 42.462752 | 0.000001 | | * | |

Cumulative Likelihood per-region

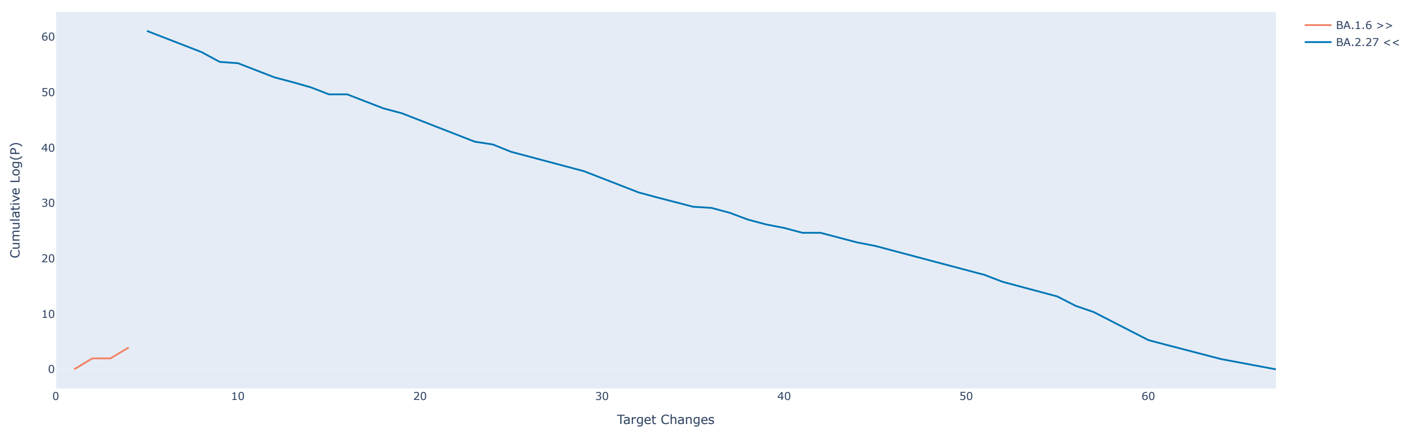


Cumulative Likelihood whole genome

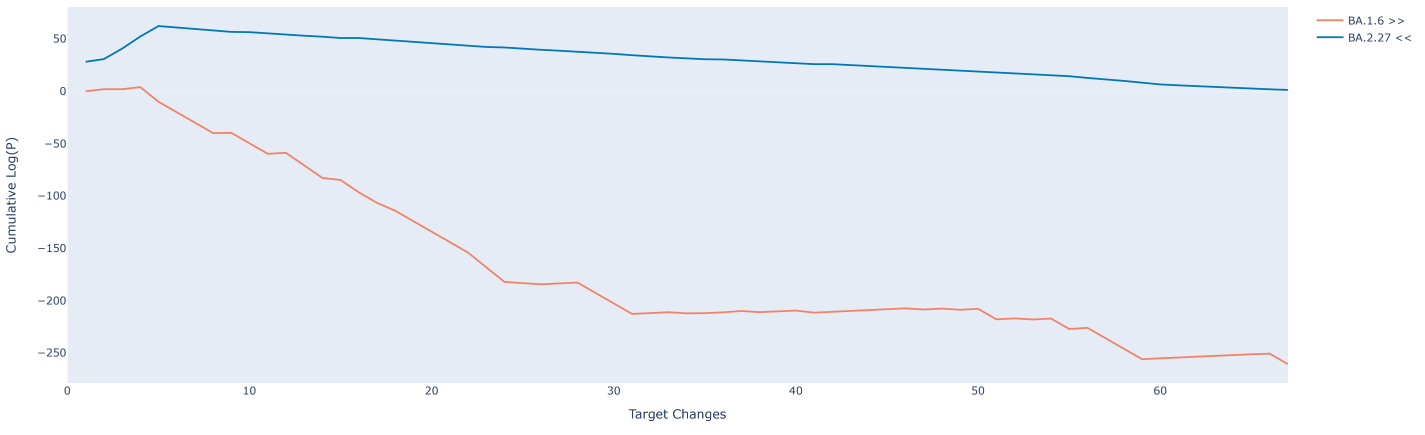


**Target sequence**

, 241_C|T, 2832_A|G, 3037_C|T, 5386_T|G, 9344_C|T, 9424_A|G, 9534_C|T, 9866_C|T, 10029_C|T, 10198_C|T, 10447_G|A, 10449_C|A, 11288_11296, 12880_C|T, 14408_C|T, 15714_C|T, 17410_C|T, 18163_A|G, 19955_C|T, 20055_A|G, 21618_C|T, 21633_21641, 21987_G|A, 22200_T|G, 22578_G|A, 22674_C|T, 22679_T|C, 22686_C|T, 22688_A|G, 22775_G|A, 22786_A|C, 22813_G|T, 22882_T|G, 22992_G|A, 22995_C|A, 23013_A|C, 23040_A|G, 23055_A|G, 23063_A|T, 23075_T|C, 23403_A|G, 23525_C|T, 23599_T|G, 23604_C|A, 23854_C|A, 23948_G|T, 24424_A|T, 24469_T|A, 25000_C|T, 25584_C|T, 26060_C|T, 26270_C|T, 26577_C|G, 26709_G|A, 26858_C|T, 27259_A|C, 27382_G|C, 27383_A|T, 27384_T|C, 27807_C|T, 28271_A|T, 28311_C|T, 28362_28370, 28881_G|A, 28882_G|A, 28883_G|C, 29510_A|C

## Case 29 (1BP 5'): XAF          test: OK

Target: (75%) 51 samples          Number of changes: 66
GT: BA.1* + BA.2*          GT BR: 8–10          GT BR coord: 10447 – 11288          Rank L1 L2: 11 4
BC: BA.1.1.9 + BA.2.7          BC BR: 8–9          BC BR coord: 10203 – 10204
Direction L1: <<          Initial region span: 1–8,9–66          Gap history (edge excluded):
Alt. candidates: [BA.1.1], [BA.2]
Model 1BP/2BP comparison:          –
Rec. model vs L1: 8.39e−293          Rec. model vs L2: 1.41e−130
Flags: Model_2BP_Bad_L1_opp

## BA.1.1.9 >>

| | num_seq | t_ch_MAX | max_CL | CL@BC_t_ch_MAX | aic | PV | PV_OK | t_ch_MAX_OK | phyl_OK |
|---|---|---|---|---|---|---|---|---|---|
| **BA.1.1.9** | 172 | 8 | 10.679710 | NaN | NaN | NaN | * | * | * |
| **BA.1.1.13** | 3393 | 8 | 10.677646 | 10.677646 | -3.355293 | 1.000000 | * | * | |
| **BA.1.1.14** | 5810 | 8 | 10.673336 | 10.673336 | -3.346672 | 0.995012 | * | * | |
| **BA.1.1.15** | 5038 | 8 | 10.672949 | 10.672949 | -3.345899 | 0.995012 | * | * | |
| **BA.1.1.16** | 594 | 8 | 10.667899 | 10.667899 | -3.335797 | 0.990050 | * | * | |
| **BA.1.1** | 262925 | 8 | 10.665914 | 10.665914 | -3.331829 | 0.985112 | * | * | * |
| **BA.1.1.2** | 2970 | 8 | 10.665536 | 10.665536 | -3.331072 | 0.985112 | * | * | |
| **BA.1.1.10** | 592 | 8 | 10.661050 | 10.661050 | -3.322100 | 0.980199 | * | * | |
| **BA.1.1.18** | 22283 | 6 | 8.457156 | 7.226730 | 3.546541 | 0.031587 | * | | |
| **BA.1.21** | 556 | 8 | 6.632564 | 6.632564 | 4.734873 | 0.017510 | * | * | |

## BA.2.7 <<

| | num_seq | t_ch_MAX | max_CL | CL@BC_t_ch_MAX | aic | PV | PV_OK | t_ch_MAX_OK | phyl_OK |
|---|---|---|---|---|---|---|---|---|---|
| **BA.2.7** | 1628 | 9 | 57.922105 | NaN | NaN | NaN | * | * | * |
| **BA.2.52** | 263 | 9 | 57.766363 | 56.492179 | 13.015642 | 0.852144 | * | * | |
| **BA.2.25** | 97 | 9 | 57.201115 | 55.926931 | 14.146137 | 0.484325 | * | * | |
| **BA.2** | 359165 | 9 | 56.764604 | 55.490665 | 15.018671 | 0.313486 | * | * | * |
| **BA.2.9.2** | 355 | 9 | 53.665235 | 52.393872 | 21.212257 | 0.014193 | * | * | |
| **BA.2.9.5** | 109 | 9 | 53.237937 | 51.963753 | 22.072494 | 0.009233 | * | * | |
| **BA.2.9** | 61803 | 9 | 53.123697 | 51.849708 | 22.300585 | 0.008230 | * | * | |
| **BA.2.22** | 2689 | 15 | 52.356527 | 44.024328 | 37.951344 | 0.000003 | | | |
| **BA.2.26** | 580 | 9 | 51.784837 | 50.510653 | 24.978694 | 0.002155 | * | * | |
| **BA.2.36** | 1320 | 9 | 50.563605 | 49.289421 | 27.421157 | 0.000636 | * | * | |

Cumulative Likelihood per-region

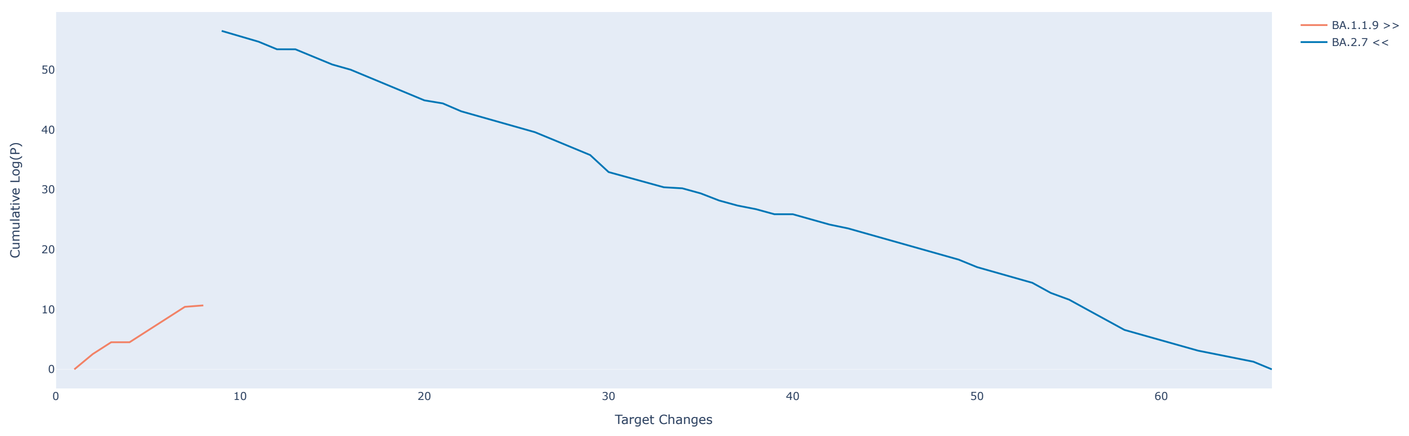


Cumulative Likelihood whole genome

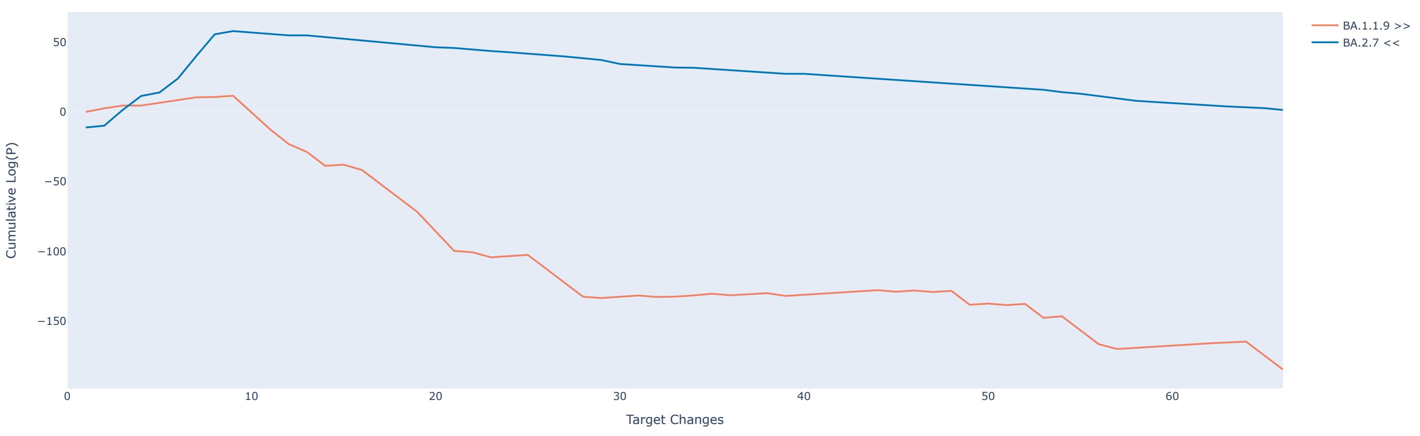


**Target sequence**

, 241_C|T, 2470_C|T, 2832_A|G, 3037_C|T, 5386_T|G, 6513_6515, 8393_G|A, 10029_C|T, 10449_C|A, 11288_11296, 12880_C|T, 14408_C|T, 15714_C|T, 17410_C|T, 18163_A|G, 19955_C|T, 20055_A|G, 21618_C|T, 21633_21641, 21987_G|A, 22200_T|G, 22578_G|A, 22674_C|T, 22679_T|C, 22686_C|T, 22688_A|G, 22775_G|A, 22786_A|C, 22792_C|T, 22813_G|T, 22882_T|G, 22992_G|A, 22995_C|A, 23013_A|C, 23040_A|G, 23055_A|G, 23063_A|T, 23075_T|C, 23403_A|G, 23525_C|T, 23599_T|G, 23604_C|A, 23854_C|A, 23948_G|T, 24424_A|T, 24469_T|A, 25000_C|T, 25584_C|T, 26060_C|T, 26270_C|T, 26577_C|G, 26709_G|A, 26858_C|T, 27259_A|C, 27382_G|C, 27383_A|T, 27384_T|C, 27807_C|T, 28271_A|T, 28311_C|T, 28362_28370, 28881_G|A, 28882_G|A, 28883_G|C, 29510_A|C, 29734_29759

## Case 30 (1BP 5'): XAG    test: OK

Target: (75%) 15 samples
Number of changes: 74
GT: BA.1* + BA.2*
GT BR: 8–9
GT BR coord: 6515 – 8393
Rank L1 L2: 11 2
BC: BA.1.1.14 + BA.2.9
BC BR: 8–9
BC BR coord: 6524 – 6525
Direction L1: <<
Initial region span: 1–6,9–74
Gap history (edge excluded): 6–9
Alt. candidates: [BA.1.1], [BA.2, BA.2.9.5]
Model 1BP/2BP comparison: –
Rec. model vs L1: 2.35e–219
Rec. model vs L2: 5.25e–38
Flags: Model_2BP_Bad_L1_opp

## BA.1.1.14 >>

| | num_seq | t_ch_MAX | max_CL | CL@BC_t_ch_MAX | aic | PV | PV_OK | t_ch_MAX_OK | phyl_OK |
|---|---|---|---|---|---|---|---|---|---|
| **BA.1.1.14** | 5810 | 6 | 8.824282 | NaN | NaN | NaN | * | * | * |
| **BA.1.1.13** | 3393 | 6 | 6.274233 | -1.755002 | 23.510004 | 7.808167e-02 | * | * | |
| **BA.1.1** | 262925 | 6 | 6.245058 | 5.921783 | 8.156434 | 1.681741e+02 | * | * | * |
| **BA.1.1.9** | 172 | 3 | 4.528654 | -11.526286 | 43.052572 | 4.461614e-06 | | | |
| **BA.1.1.6** | 22 | 3 | 4.528654 | -17.403084 | 54.806168 | 1.246925e-08 | | | |
| **BA.1.1.12** | 1943 | 3 | 4.528654 | -19.035361 | 58.070722 | 2.443095e-09 | | | |
| **BA.1.1.15** | 5038 | 3 | 4.528058 | -11.532848 | 43.065697 | 4.417220e-06 | | | |
| **BA.1.1.2** | 2970 | 3 | 4.521573 | -11.540123 | 43.080247 | 4.395189e-06 | | | |
| **BA.1.1.16** | 594 | 3 | 4.518527 | -11.538098 | 43.076195 | 4.395189e-06 | | | |
| **BA.1.1.18** | 22283 | 3 | 4.515195 | -3.651606 | 27.303211 | 1.173711e-02 | * | | |

## BA.2.9 <<

| | num_seq | t_ch_MAX | max_CL | CL@BC_t_ch_MAX | aic | PV | PV_OK | t_ch_MAX_OK | phyl_OK |
|---|---|---|---|---|---|---|---|---|---|
| **BA.2.9** | 61803 | 9 | 60.696081 | NaN | NaN | NaN | * | * | * |
| **BA.2** | 359165 | 9 | 55.356958 | 55.356958 | 39.286083 | 4.795871e-03 | * | * | * |
| **BA.2.9.5** | 109 | 9 | 53.997978 | 53.997978 | 42.004045 | 1.237082e-03 | * | * | * |
| **BA.2.7** | 1628 | 9 | 53.883809 | 47.769577 | 54.460846 | 2.436373e-06 | | * | |
| **BA.2.81** | 34 | 20 | 51.517315 | 42.968189 | 64.063622 | 2.005074e-08 | | | |
| **BA.2.52** | 263 | 24 | 51.081445 | 45.070074 | 59.859853 | 1.637377e-07 | | | |
| **BA.2.25** | 97 | 24 | 50.737609 | 44.730048 | 60.539904 | 1.165436e-07 | | | |
| **BA.2.22** | 2689 | 24 | 49.863956 | 31.177589 | 87.644821 | 1.519842e-13 | | | |
| **BA.2.45** | 108 | 19 | 46.532353 | 41.264455 | 67.471090 | 3.644670e-09 | | | |
| **BA.2.9.2** | 355 | 24 | 45.238527 | 39.216837 | 71.566326 | 4.691962e-10 | | | * |

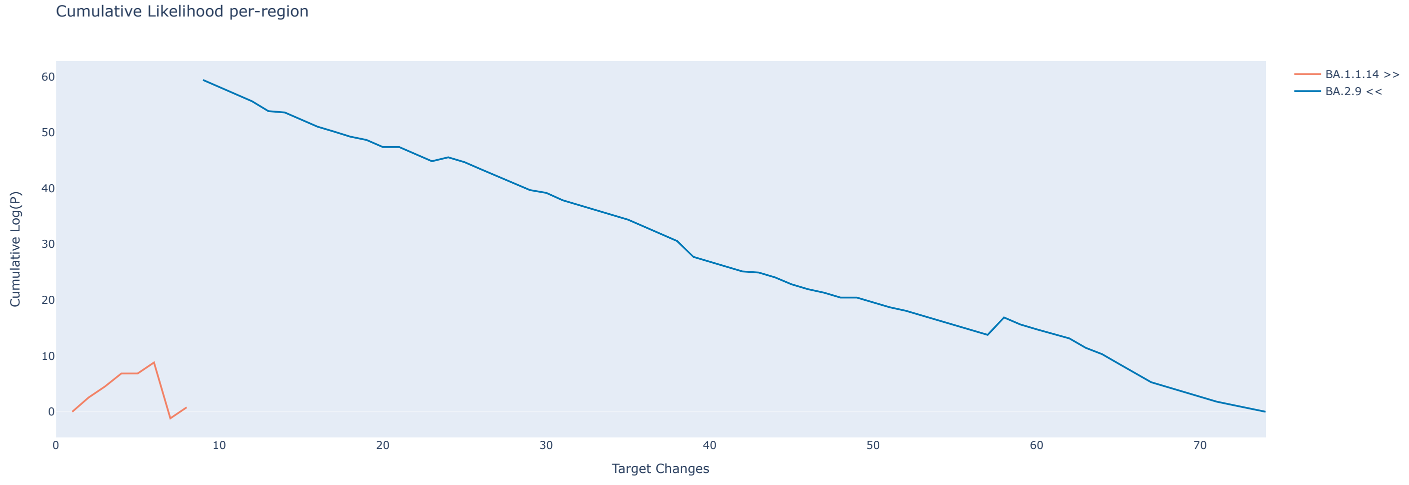


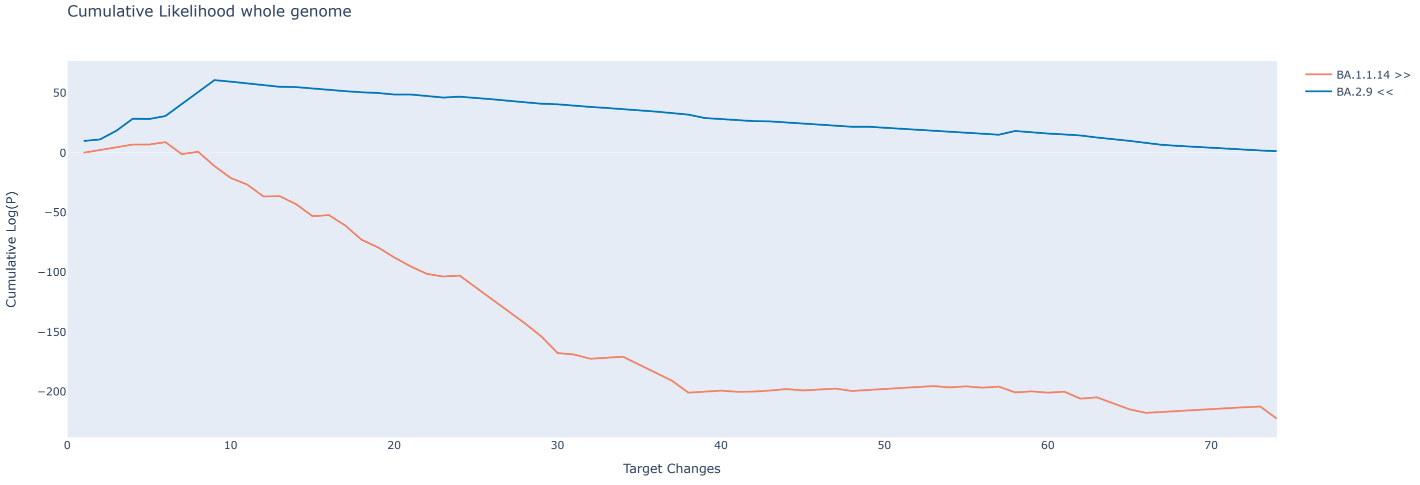


**Target sequence**

, 241_C|T, 2470_C|T, 2832_A|G, 2857_C|T, 3037_C|T, 5386_T|G, 5585_C|A, 6513_6515, 9344_C|T, 9424_A|G, 9534_C|T, 9866_C|T, 10029_C|T, 10198_C|T, 10447_G|A, 10449_C|A, 11288_11296, 12334_A|G, 12880_C|T, 14408_C|T, 15714_C|T, 17410_C|T, 17502_C|T, 18163_A|G, 19955_C|T, 20055_A|G, 21618_C|T, 21633_21641, 21987_G|A, 22200_T|G, 22578_G|A, 22674_C|T, 22679_T|C, 22686_C|T, 22688_A|G, 22775_G|A, 22786_A|C, 22792_C|T, 22813_G|T, 22882_T|G, 22992_G|A, 22995_C|A, 23013_A|C, 23040_A|G, 23055_A|G, 23063_A|T, 23075_T|C, 23403_A|G, 23525_C|T, 23599_T|G, 23604_C|A, 23854_C|A, 23948_G|T, 24424_A|T, 24469_T|A, 25000_C|T, 25584_C|T, 26060_C|T, 26270_C|T, 26577_C|G, 26709_G|A, 26858_C|T, 27259_A|C, 27382_G|C, 27383_A|T, 27384_T|C, 27807_C|T, 28271_A|T, 28311_C|T, 28362_28370, 28881_G|A, 28882_G|A, 28883_G|C, 29510_A|C

## Case 31 (1BP 5'): XAM test: OK

Target: (75%) 67 samples
Number of changes: 74
GT: BA.1.1 + BA.2.9
GT BR: 6–7
GT BR coord: 8087 – 9192
Rank L1 L2: 6 1
BC: BA.1.1.9 + BA.2.9
BC BR: 6–7
BC BR coord: 6524 – 6525
Direction L1: <<
Initial region span: 1–6,7–74
Gap history (edge excluded):
Alt. candidates: [BA.1.1], [BA.2.9.3]
Model 1BP/2BP comparison: –
Rec. model vs L1: 2.08e-321
Rec. model vs L2: 7.24e-52
Flags: Model_2BP_Bad_L1_opp

## BA.1.1.9 >>

| | num_seq | t_ch_MAX | max_CL | CL@BC_t_ch_MAX | aic | PV | PV_OK | t_ch_MAX_OK | phyl_OK |
|---|---|---|---|---|---|---|---|---|---|
| **BA.1.1.9** | 172 | 6 | 8.473714 | NaN | NaN | NaN | * | * | * |
| **BA.1.1.13** | 3393 | 6 | 8.471650 | 8.471650 | -2.943300 | 0.995012 | * | * | |
| **BA.1.1.14** | 5810 | 6 | 8.467340 | 8.467340 | -2.934680 | 0.990050 | * | * | |
| **BA.1.1.15** | 5038 | 6 | 8.467152 | 8.467152 | -2.934303 | 0.990050 | * | * | |
| **BA.1.1.16** | 594 | 6 | 8.461902 | 8.461902 | -2.923805 | 0.985112 | * | * | |
| **BA.1.1** | 262925 | 6 | 8.460241 | 8.460241 | -2.920483 | 0.985112 | * | * | * |
| **BA.1.1.2** | 2970 | 6 | 8.459877 | 8.459877 | -2.919753 | 0.985112 | * | * | |
| **BA.1.1.18** | 22283 | 6 | 8.457156 | 8.457156 | -2.914312 | 0.980199 | * | * | |
| **BA.1.1.10** | 592 | 6 | 8.455054 | 8.455054 | -2.910107 | 0.980199 | * | * | |
| **BA.1.1.6** | 22 | 4 | 4.532562 | 2.596916 | 8.806168 | 0.002795 | * | | |

## BA.2.9 <<

| | num_seq | t_ch_MAX | max_CL | CL@BC_t_ch_MAX | aic | PV | PV_OK | t_ch_MAX_OK | phyl_OK |
|---|---|---|---|---|---|---|---|---|---|
| **BA.2.9** | 61803 | 7 | 71.361311 | NaN | NaN | NaN | * | * | * |
| **BA.2.9.3** | 351 | 7 | 65.379932 | 65.379932 | 25.240136 | 2.528826e-03 | * | * | * |
| **BA.2** | 359165 | 7 | 57.183810 | 57.183810 | 41.632380 | 6.980326e-07 | | * | * |
| **BA.2.9.2** | 355 | 7 | 49.998639 | 49.998639 | 56.002722 | 5.290173e-10 | | * | * |
| **BA.2.9.5** | 109 | 7 | 49.582649 | 49.582649 | 56.834703 | 3.493314e-10 | | * | * |
| **BA.2.7** | 1628 | 7 | 44.419240 | 38.305009 | 79.389982 | 4.409567e-15 | | * | |
| **BA.2.9.6** | 98 | 7 | 41.654632 | 41.654632 | 72.690737 | 1.256847e-13 | | * | * |
| **BA.2.9.1** | 420 | 19 | 40.448741 | 34.803053 | 86.393893 | 1.331574e-16 | | | * |
| **BA.2.26** | 580 | 7 | 38.208551 | 31.280177 | 93.439645 | 3.921725e-18 | | * | |
| **BA.2.3** | 22517 | 7 | 36.545629 | 31.789061 | 92.421879 | 6.530815e-18 | | * | |

Cumulative Likelihood per-region

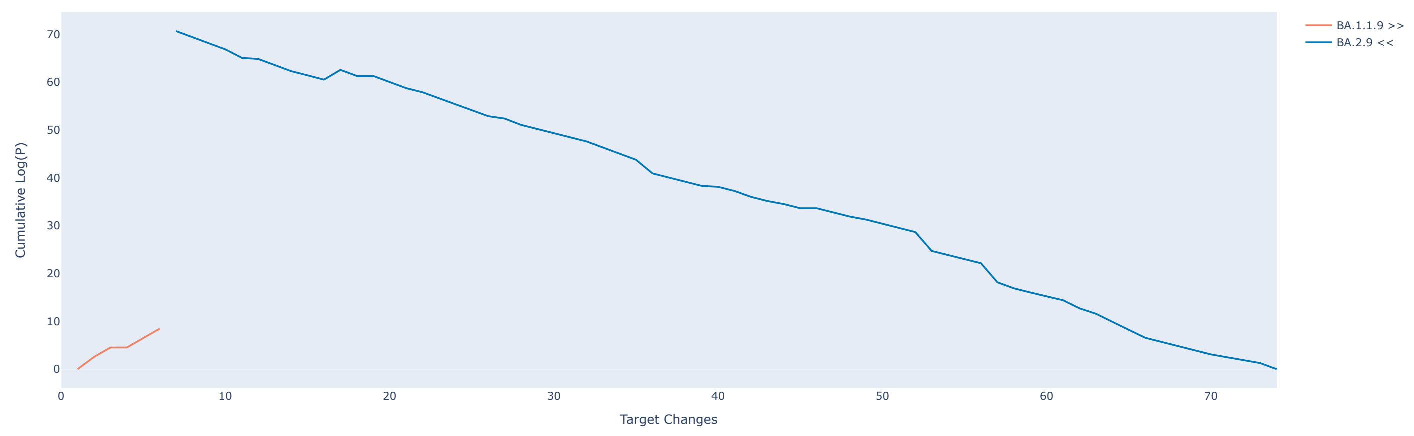


Cumulative Likelihood whole genome

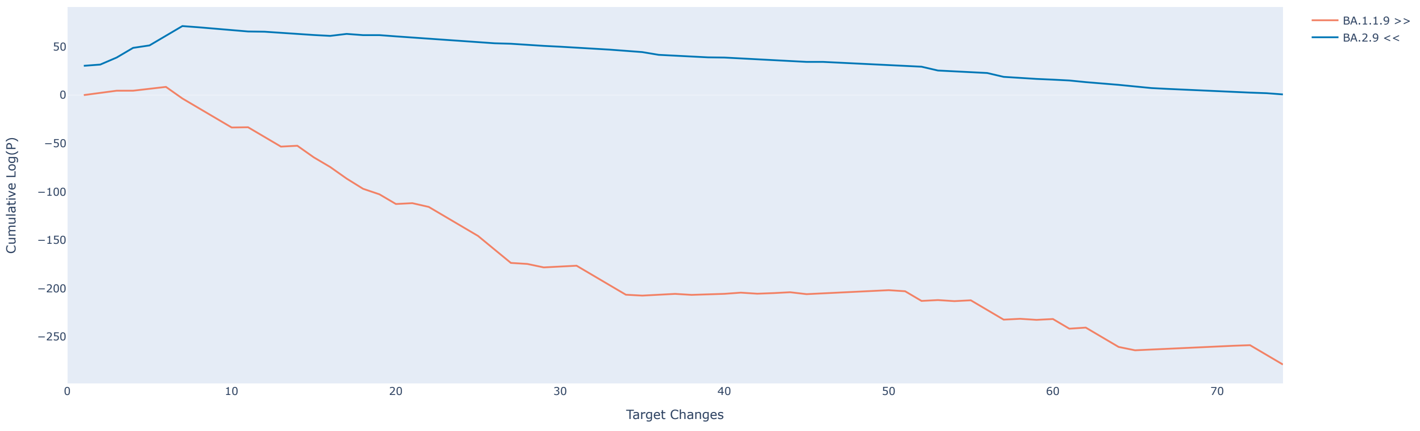


**Target sequence**

, 241_C|T, 2470_C|T, 2832_A|G, 3037_C|T, 5386_T|G, 6513_6515, 9344_C|T, 9424_A|G, 9534_C|T, 9866_C|T, 10029_C|T, 10198_C|T, 10447_G|A, 10449_C|A, 11288_11296, 11514_C|T, 12880_C|T, 14408_C|T, 15714_C|T, 17410_C|T, 18163_A|G, 19955_C|T, 20055_A|G, 21618_C|T, 21633_21641, 21987_G|A, 22200_T|G, 22578_G|A, 22674_C|T, 22679_T|C, 22686_C|T, 22688_A|G, 22775_G|A, 22786_A|C, 22792_C|T, 22813_G|T, 22882_T|G, 22992_G|A, 22995_C|A, 23013_A|C, 23040_A|G, 23055_A|G, 23063_A|T, 23075_T|C, 23403_A|G, 23525_C|T, 23599_T|G, 23604_C|A, 23854_C|A, 23948_G|T, 24424_A|T, 24433_A|G, 24469_T|A, 25000_C|T, 25584_C|T, 25624_C|T, 26060_C|T, 26270_C|T, 26577_C|G, 26709_G|A, 26858_C|T, 27259_A|C, 27382_G|C, 27383_A|T, 27384_T|C, 27807_C|T, 28271_A|T, 28311_C|T, 28362_28370, 28881_G|A, 28882_G|A, 28883_G|C, 29510_A|C, 29734_29759

## Case 32 (1BP 5'): XAR    test: KO

Target: (75%) 65 samples
Number of changes: 69
GT: BA.1* + BA.2*
GT BR: 2–4
GT BR coord: 2833 – 4184
Rank L1 L2: 11 –
BC: BA.2.23
BC BR:
BC BR coord:
Direction L1: <<
Initial region span: 3–69
Gap history (edge excluded):
Alt. candidates: [BA.2]
Model 1BP/2BP comparison: –
Rec. model vs L1: –
Rec. model vs L2: –
Flags: NotEnoughSpaceAfterL1, SingleCandidateGenome

## BA.2.23 <<

| | num_seq | t_ch_MAX | max_CL | CL@BC_t_ch_MAX | aic | PV | PV_OK | t_ch_MAX_OK | phyl_OK |
|---|---|---|---|---|---|---|---|---|---|
| **BA.2.23** | 8100 | 3 | 66.335333 | NaN | NaN | NaN | * | * | * |
| **BA.2.29** | 102 | 3 | 65.838789 | 43.661241 | 78.677518 | 3.263909e-05 | * | * | |
| **BA.2** | 359165 | 3 | 65.821588 | 53.266182 | 59.467635 | 4.843246e-01 | * | * | * |
| **BA.2.27** | 19 | 4 | 65.794290 | 44.173752 | 77.652497 | 5.462603e-05 | * | * | |
| **BA.2.5** | 473 | 4 | 65.562289 | 45.154473 | 75.691054 | 1.455487e-04 | * | * | |
| **BA.2.26** | 580 | 6 | 63.662917 | 39.876934 | 86.246132 | 7.411965e-07 | | | |
| **BA.2.52** | 263 | 3 | 62.989699 | 41.782043 | 82.435913 | 4.980402e-06 | | * | |
| **BA.2.25** | 97 | 3 | 62.448986 | 40.507971 | 84.984058 | 1.398654e-06 | | * | |
| **BA.2.10** | 12936 | 3 | 62.109936 | 49.553229 | 66.893542 | 1.185507e-02 | * | * | |
| **BA.2.11** | 22 | 6 | 61.897205 | 42.414474 | 81.171053 | 9.398128e-06 | | | |

Cumulative Likelihood per-region

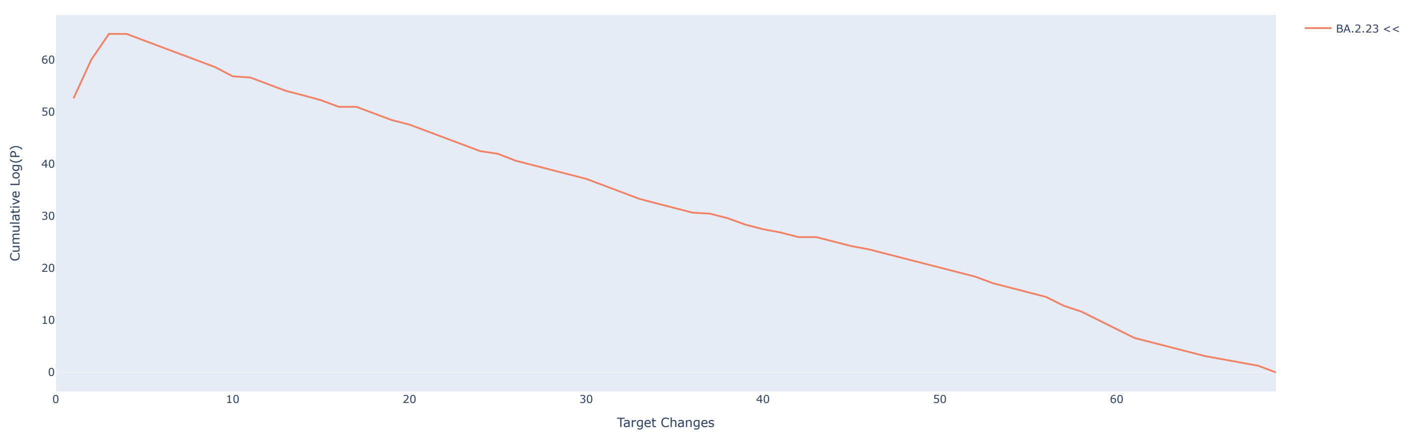


Cumulative Likelihood whole genome

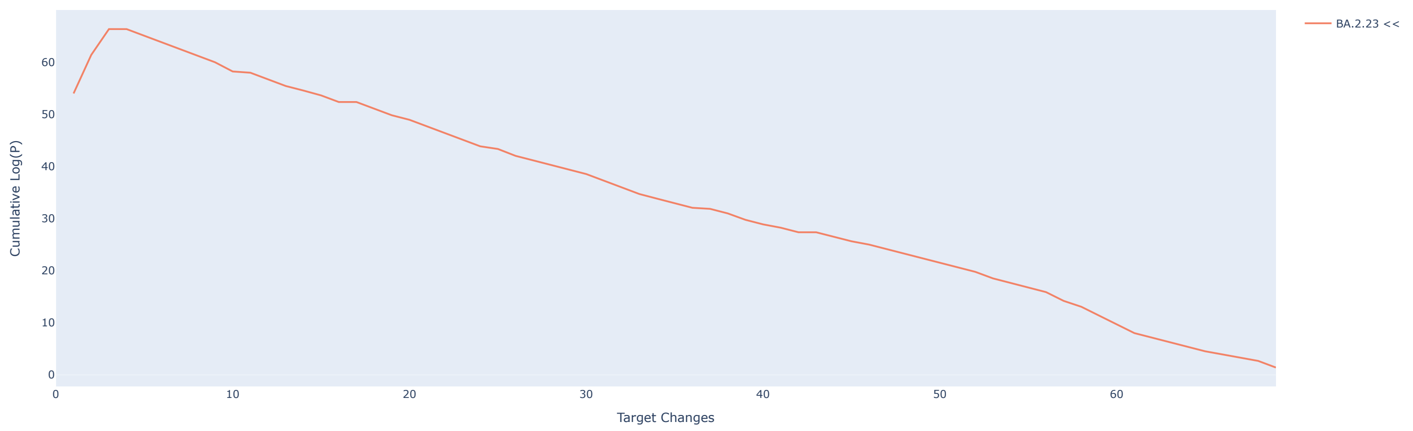


**Target sequence**

, 241_C|T, 2832_A|G, 3037_C|T, 4184_G|A, 4321_C|T, 9344_C|T, 9424_A|G, 9534_C|T, 9866_C|T, 10029_C|T, 10198_C|T, 10447_G|A, 10449_C|A, 11288_11296, 12880_C|T, 14408_C|T, 15714_C|T, 17410_C|T, 18163_A|G, 19955_C|T, 20055_A|G, 21618_C|T, 21633_21641, 21987_G|A, 22200_T|G, 22578_G|A, 22674_C|T, 22679_T|C, 22686_C|T, 22688_A|G, 22775_G|A, 22786_A|C, 22813_G|T, 22882_T|G, 22992_G|A, 22995_C|A, 23013_A|C, 23040_A|G, 23055_A|G, 23063_A|T, 23075_T|C, 23403_A|G, 23525_C|T, 23599_T|G, 23604_C|A, 23854_C|A, 23948_G|T, 24424_A|T, 24469_T|A, 25000_C|T, 25584_C|T, 26060_C|T, 26270_C|T, 26577_C|G, 26709_G|A, 26858_C|T, 27259_A|C, 27382_G|C, 27383_A|T, 27384_T|C, 27807_C|T, 28271_A|T, 28311_C|T, 28362_28370, 28881_G|A, 28882_G|A, 28883_G|C, 29510_A|C, 29734_29759

## Case 33 (1BP 5'): XAU          test: OK

Target: (75%) 17 samples
Number of changes: 71
GT: BA.1.1* + BA.2.9*
GT BR: 3–5
GT BR coord: 2834 – 4184
Rank L1 L2: 8 1
BC: BA.1.1.9 + BA.2.9
BC BR: 4–5
BC BR coord: 3037 – 3038
Direction L1: <<
Initial region span: 1–4,5–71
Gap history (edge excluded):
Alt. candidates: [BA.1.1], [BA.2, BA.2.9.3]
Model 1BP/2BP comparison: –
Rec. model vs L1: 0.00e+00
Rec. model vs L2: 1.08e–25
Flags: Model_2BP_Bad_L1_opp

## BA.1.1.9 >>

| | num_seq | t_ch_MAX | max_CL | CL@BC_t_ch_MAX | aic | PV | PV_OK | t_ch_MAX_OK | phyl_OK |
|---|---|---|---|---|---|---|---|---|---|
| **BA.1.1.9** | 172 | 4 | 4.532562 | NaN | NaN | NaN | * | * | * |
| **BA.1.1.6** | 22 | 4 | 4.532562 | 4.532562 | 0.934876 | 1.000000 | * | * | |
| **BA.1.1.15** | 5038 | 4 | 4.531768 | 4.531768 | 0.936464 | 0.995012 | * | * | |
| **BA.1.1.13** | 3393 | 4 | 4.531678 | 4.531678 | 0.936645 | 0.995012 | * | * | |
| **BA.1.1.12** | 1943 | 3 | 4.528654 | -2.974968 | 15.949936 | 0.000548 | * | * | |
| **BA.1.1.14** | 5810 | 4 | 4.528600 | 4.528600 | 0.942800 | 0.995012 | * | * | |
| **BA.1.1.2** | 2970 | 4 | 4.525482 | 4.525482 | 0.949037 | 0.990050 | * | * | |
| **BA.1.1** | 262925 | 4 | 4.522543 | 4.522543 | 0.954914 | 0.990050 | * | * | * |
| **BA.1.1.16** | 594 | 4 | 4.522435 | 4.522435 | 0.955129 | 0.985112 | * | * | |
| **BA.1.1.18** | 22283 | 4 | 4.519059 | 4.519059 | 0.961882 | 0.985112 | * | * | |

## BA.2.9 <<

| | num_seq | t_ch_MAX | max_CL | CL@BC_t_ch_MAX | aic | PV | PV_OK | t_ch_MAX_OK | phyl_OK |
|---|---|---|---|---|---|---|---|---|---|
| **BA.2.9** | 61803 | 5 | 71.300851 | NaN | NaN | NaN | * | * | * |
| **BA.2.9.5** | 109 | 7 | 69.156596 | 61.446773 | 29.106454 | 5.248412e-05 | * | | * |
| **BA.2.9.2** | 355 | 7 | 67.183196 | 63.616784 | 24.766432 | 4.596708e-04 | * | | * |
| **BA.2** | 359165 | 4 | 64.587876 | 64.586552 | 22.826896 | 1.212586e-03 | * | * | * |
| **BA.2.9.3** | 351 | 4 | 61.828350 | 61.824441 | 28.351117 | 7.674671e-05 | * | * | * |
| **BA.2.9.6** | 98 | 7 | 61.527663 | 58.885414 | 34.229172 | 4.057271e-06 | | | * |
| **BA.2.52** | 263 | 4 | 57.606930 | 57.606831 | 36.786338 | 1.128073e-06 | | * | |
| **BA.2.25** | 97 | 4 | 57.270714 | 57.266805 | 37.466390 | 8.029286e-07 | | * | |
| **BA.2.7** | 1628 | 7 | 53.492145 | 49.914671 | 52.170658 | 5.159558e-10 | | | |
| **BA.2.9.7** | 852 | 4 | 52.529224 | 52.525315 | 46.949369 | 7.016509e-09 | | * | * |

Cumulative Likelihood per-region

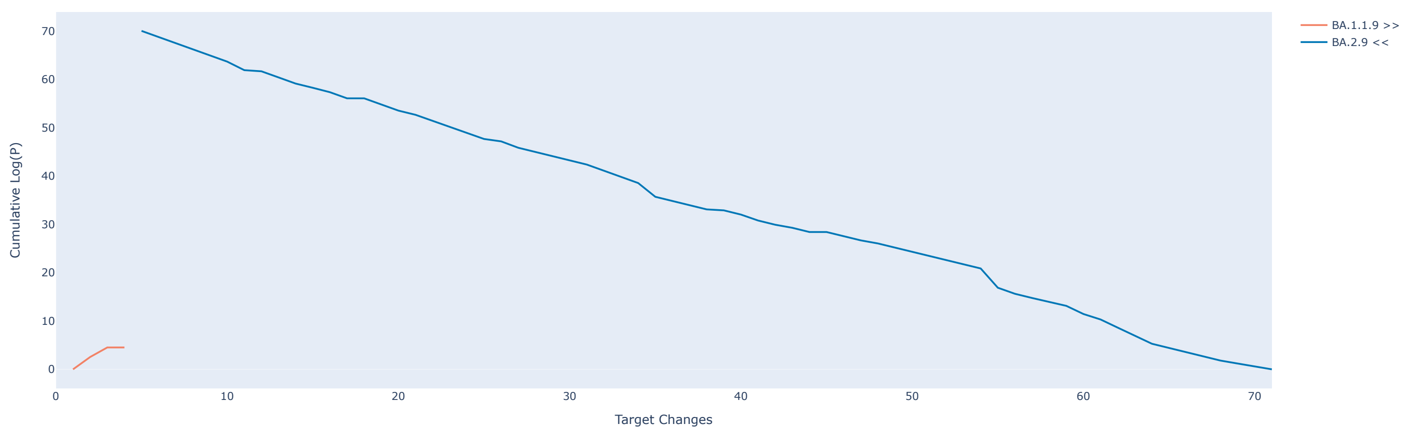


Cumulative Likelihood whole genome

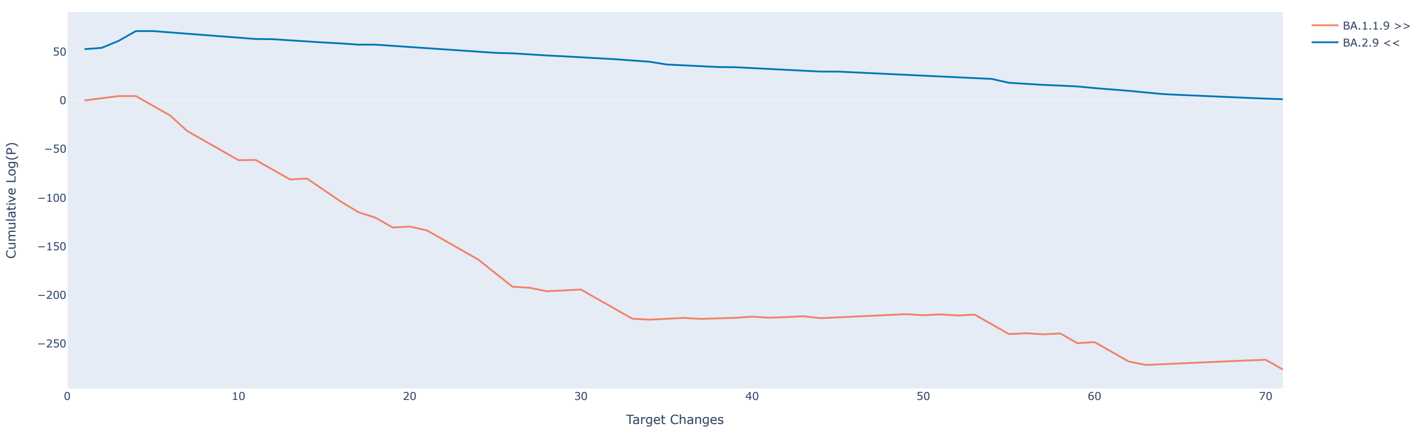


**Target sequence**

, 241_C|T, 2470_C|T, 2832_A|G, 3037_C|T, 4184_G|A, 4321_C|T, 9344_C|T, 9424_A|G, 9534_C|T, 9866_C|T, 10029_C|T, 10198_C|T, 10447_G|A, 10449_C|A, 11288_11296, 12880_C|T, 14408_C|T, 15714_C|T, 17410_C|T, 18163_A|G, 19955_C|T, 20055_A|G, 21618_C|T, 21633_21641, 21987_G|A, 22200_T|G, 22578_G|A, 22674_C|T, 22679_T|C, 22686_C|T, 22688_A|G, 22775_G|A, 22786_A|C, 22792_C|T, 22813_G|T, 22882_T|G, 22992_G|A, 22995_C|A, 23013_A|C, 23040_A|G, 23055_A|G, 23063_A|T, 23075_T|C, 23403_A|G, 23525_C|T, 23599_T|G, 23604_C|A, 23854_C|A, 23948_G|T, 24424_A|T, 24469_T|A, 25000_C|T, 25584_C|T, 25624_C|T, 26060_C|T, 26270_C|T, 26577_C|G, 26709_G|A, 26858_C|T, 27259_A|C, 27382_G|C, 27383_A|T, 27384_T|C, 27807_C|T, 28271_A|T, 28311_C|T, 28362_28370, 28881_G|A, 28882_G|A, 28883_G|C, 29510_A|C

## Case 34 (1BP 5'): XE    test: OK

Target: (75%) 1342 samples
Number of changes: 67
GT: BA.1* + BA.2*
GT BR: 9–11
GT BR coord: 10447 – 11288
Rank L1 L2: 4 2
BC: BD.1 + BA.2.31
BC BR: 10–11
BC BR coord: 10449 – 10450
Direction L1: <<
Initial region span: 1–10,12–67
Gap history (edge excluded): 10–12
Alt. candidates: [BA.1.17.2, BA.1.17], []
Model 1BP/2BP comparison: –
Rec. model vs L1: 3.92e–298
Rec. model vs L2: 6.83e–134
Flags: Model_2BP_Bad_L1_opp

## BD.1 >>

| | num_seq | t_ch_MAX | max_CL | CL@BC_t_ch_MAX | aic | PV | PV_OK | t_ch_MAX_OK | phyl_OK |
|---|---|---|---|---|---|---|---|---|---|
| **BD.1** | 2101 | 10 | 18.007111 | NaN | NaN | NaN | * | * | * |
| **BA.1.17.2** | 39682 | 10 | 17.790985 | 17.790985 | -15.581969 | 8.065414e-01 | * | * | * |
| **BA.1.17** | 6954 | 10 | 14.469936 | 14.469936 | -8.939872 | 2.915876e-02 | * | * | * |
| **BA.1** | 100089 | 10 | 6.376175 | 6.376175 | 7.247651 | 8.895189e-06 | | * | * |
| **BA.1.1.1** | 8304 | 10 | 3.041938 | 3.041938 | 13.916125 | 3.167985e-07 | | * | |
| **BA.1.21** | 556 | 5 | 2.975626 | -2.011945 | 24.023890 | 2.030467e-09 | | | |
| **BA.1.1.4** | 767 | 5 | 2.653763 | -2.301454 | 24.602908 | 1.519325e-09 | | | |
| **BA.1.6** | 60 | 3 | 1.981133 | -11.000667 | 42.001335 | 2.530979e-13 | | | |
| **BA.1.4** | 12 | 3 | 1.981133 | -11.087679 | 42.175357 | 2.313141e-13 | | | |
| **BA.1.24** | 96 | 3 | 1.981133 | -11.000667 | 42.001335 | 2.530979e-13 | | | |

## BA.2.31 <<

| | num_seq | t_ch_MAX | max_CL | CL@BC_t_ch_MAX | aic | PV | PV_OK | t_ch_MAX_OK | phyl_OK |
|---|---|---|---|---|---|---|---|---|---|
| **BA.2.31** | 453 | 12 | 53.453162 | NaN | NaN | NaN | * | * | * |
| **BA.2** | 359165 | 10 | 53.319035 | 52.448122 | 17.103756 | 152.170256 | * | | * |
| **BA.2.1** | 7140 | 15 | 52.371636 | 46.485751 | 29.028498 | 0.390628 | * | | |
| **BA.2.23** | 8100 | 15 | 52.346506 | 50.317448 | 21.365104 | 17.993310 | * | | |
| **BA.2.26** | 580 | 15 | 52.229152 | 44.430877 | 33.138247 | 0.050037 | * | | |
| **BA.2.29** | 102 | 15 | 51.845268 | 44.048718 | 33.902563 | 0.034218 | * | | |
| **BA.2.27** | 19 | 15 | 51.804677 | 44.008128 | 33.983745 | 0.032876 | * | | |
| **BA.2.5** | 473 | 15 | 51.589626 | 43.790959 | 34.418081 | 0.026384 | * | | |
| **BA.2.10** | 12936 | 16 | 50.942678 | 48.031984 | 25.936031 | 1.831252 | * | | |
| **BA.2.37** | 2937 | 10 | 50.877084 | 50.006032 | 21.987936 | 13.197138 | * | | |

Cumulative Likelihood per-region

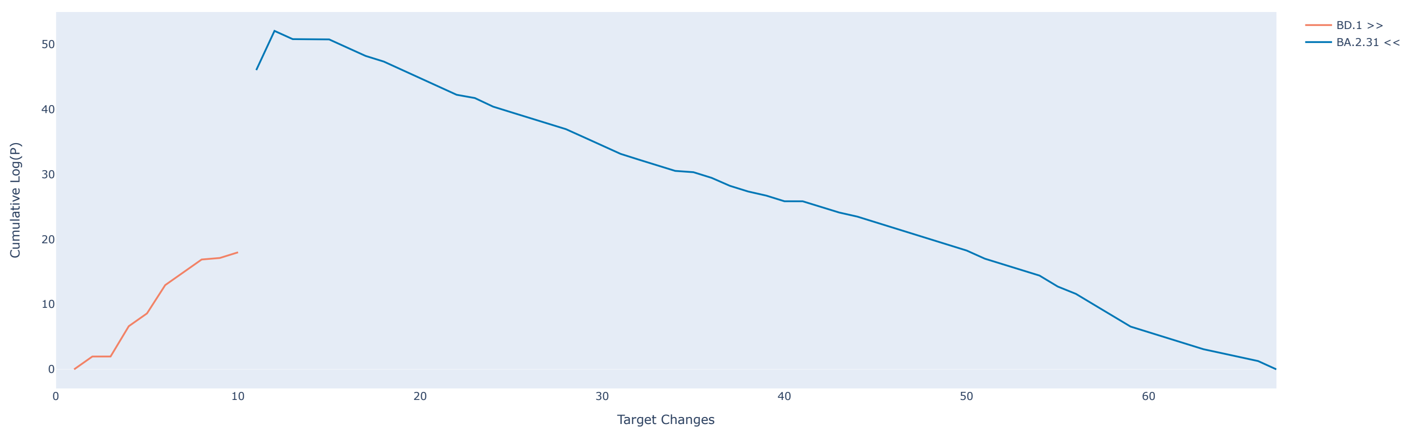


Cumulative Likelihood whole genome

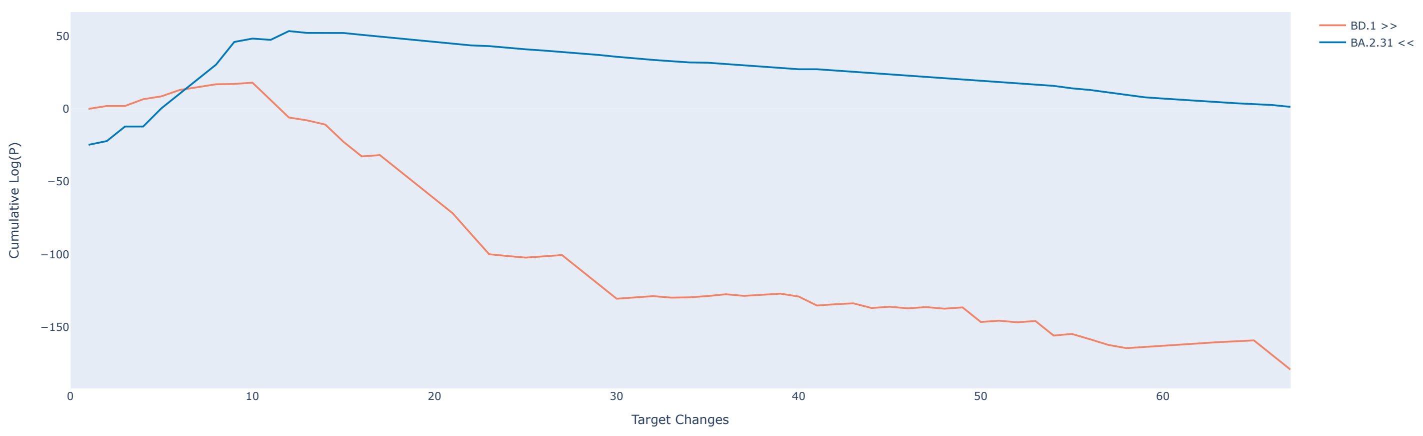


**Target sequence**

, 241_C|T, 2832_A|G, 3037_C|T, 3241_C|T, 5386_T|G, 5924_G|A, 6513_6515, 8393_G|A, 10029_C|T, 10449_C|A, 11288_11296, 12880_C|T, 14408_C|T, 14599_C|T, 15714_C|T, 17410_C|T, 18163_A|G, 19955_C|T, 20055_A|G, 21618_C|T, 21633_21641, 21987_G|A, 22200_T|G, 22578_G|A, 22674_C|T, 22679_T|C, 22686_C|T, 22688_A|G, 22775_G|A, 22786_A|C, 22813_G|T, 22882_T|G, 22992_G|A, 22995_C|A, 23013_A|C, 23040_A|G, 23055_A|G, 23063_A|T, 23075_T|C, 23403_A|G, 23525_C|T, 23599_T|G, 23604_C|A, 23854_C|A, 23948_G|T, 24424_A|T, 24469_T|A, 25000_C|T, 25584_C|T, 26060_C|T, 26270_C|T, 26577_C|G, 26709_G|A, 26858_C|T, 27259_A|C, 27382_G|C, 27383_A|T, 27384_T|C, 27807_C|T, 28271_A|T, 28311_C|T, 28362_28370, 28881_G|A, 28882_G|A, 28883_G|C, 29510_A|C, 29734_29759

## Case 35 (1BP 5'): XF          test: OK

Target: (75%) 19 samples
Number of changes: 64
GT: B.1.617.2* + BA.1*
GT BR: 6–7
GT BR coord: 5385 – 6512
Rank L1 L2: 7 2
BC: AY.4.2 + BA.1.16
BC BR: 6–7
BC BR coord: 5387 – 5388
Direction L1: <<
Initial region span: 1–3,7–64
Gap history (edge excluded): 3–7
Alt. candidates: [AY.4.2.1], [BA.1, BA.1.1]
Model 1BP/2BP comparison: –
Rec. model vs L1: 0.00e+00
Rec. model vs L2: 9.20e–46
Flags: Model_2BP_Bad_L1_opp

## AY.4.2 >>

| | num_seq | t_ch_MAX | max_CL | CL@BC_t_ch_MAX | aic | PV | PV_OK | t_ch_MAX_OK | phyl_OK |
|---|---|---|---|---|---|---|---|---|---|
| **AY.4.2** | 47333 | 3 | 4.077975 | NaN | NaN | NaN | * | * | * |
| **AY.37** | 1178 | 3 | 3.763495 | -33.425898 | 86.851795 | 4.152067e-13 | | * | |
| **AY.5** | 24784 | 6 | 3.106542 | 3.106542 | 13.786916 | 3.041177e+03 | * | | |
| **AY.3** | 79233 | 3 | 2.950925 | -6.051023 | 32.102046 | 3.214221e-01 | * | * | |
| **AY.117** | 7025 | 3 | 1.279257 | -13.971529 | 47.943057 | 1.168055e-04 | * | * | |
| **AY.4.2.1** | 8281 | 3 | 1.121055 | -7.873524 | 35.747048 | 5.181892e-02 | * | * | * |
| **B.1.617.2** | 41469 | 6 | 1.048206 | 1.048206 | 17.903588 | 3.895530e+02 | * | | * |

## BA.1.16 <<

| | num_seq | t_ch_MAX | max_CL | CL@BC_t_ch_MAX | aic | PV | PV_OK | t_ch_MAX_OK | phyl_OK |
|---|---|---|---|---|---|---|---|---|---|
| **BA.1.16** | 3129 | 7 | 69.768099 | NaN | NaN | NaN | * | * | * |
| **BA.1** | 100089 | 7 | 68.077816 | 68.077816 | -8.155632 | 0.184520 | * | * | * |
| **BA.1.9** | 186 | 7 | 64.662332 | 64.662332 | -1.324665 | 0.006036 | * | * | |
| **BA.1.18** | 10104 | 7 | 63.419597 | 57.873603 | 12.252795 | 0.000007 | | * | |
| **BA.1.17** | 6954 | 7 | 63.376797 | 59.022066 | 9.955869 | 0.000021 | * | * | |
| **BA.1.13** | 306 | 7 | 62.685242 | 62.685242 | 2.629516 | 0.000838 | * | * | |
| **BA.1.1.17** | 191 | 7 | 61.934982 | 61.934982 | 4.130037 | 0.000396 | * | * | |
| **BA.1.24** | 96 | 7 | 61.555780 | 61.555780 | 4.888440 | 0.000271 | * | * | |
| **BA.1.1** | 262925 | 7 | 60.104986 | 60.104986 | 7.790027 | 0.000063 | * | * | * |
| **BA.1.5** | 274 | 7 | 58.982813 | 58.982813 | 10.034374 | 0.000021 | * | * | |

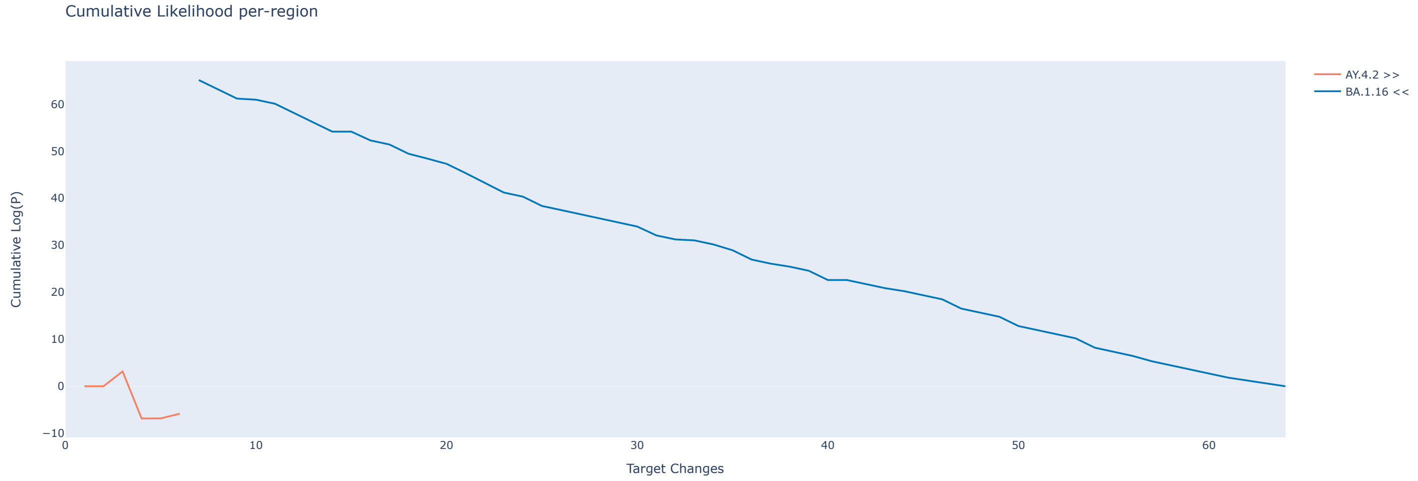


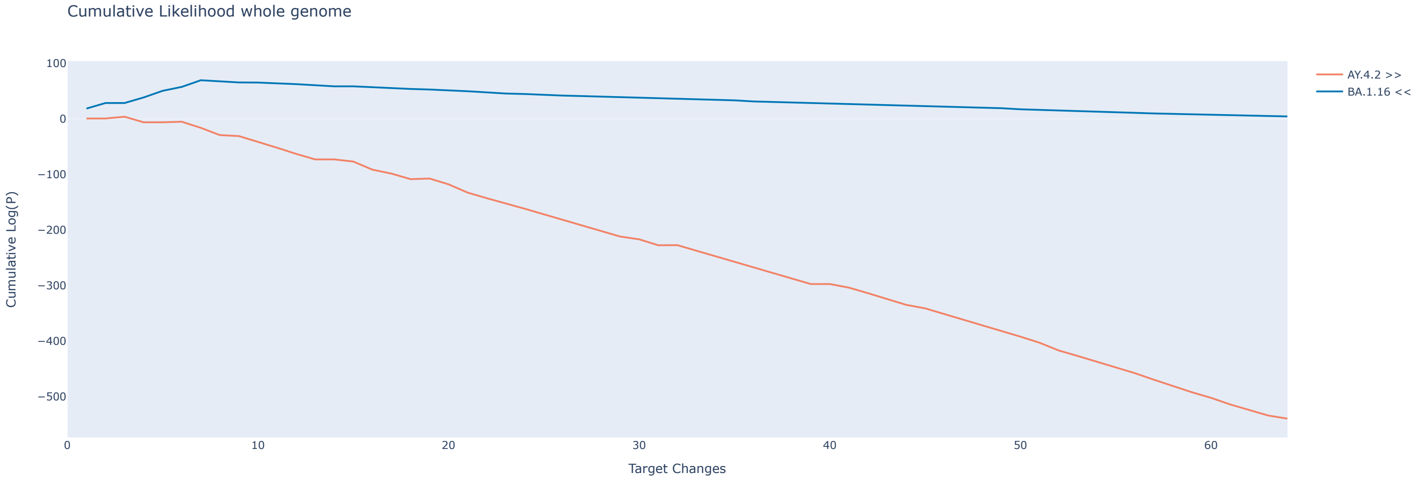


**Target sequence**

, 210_G|T, 241_C|T, 1390_T|C, 2255_A|G, 3037_C|T, 4181_G|T, 6513_6515, 8393_G|A, 10029_C|T, 10449_C|A, 11285_11293, 11537_A|G, 13195_T|C, 14408_C|T, 15240_C|T, 18163_A|G, 21762_C|T, 21765_21770, 21846_C|T, 21987_21995, 22194_22196, 22204_.|GAGCCAGAA, 22578_G|A, 22673_T|C, 22674_C|T, 22679_T|C, 22686_C|T, 22813_G|T, 22882_T|G, 22898_G|A, 22992_G|A, 22995_C|A, 23013_A|C, 23040_A|G, 23048_G|A, 23055_A|G, 23063_A|T, 23075_T|C, 23202_C|A, 23403_A|G, 23525_C|T, 23599_T|G, 23604_C|A, 23854_C|A, 23948_G|T, 24130_C|A, 24424_A|T, 24469_T|A, 24503_C|T, 25000_C|T, 25584_C|T, 26270_C|T, 26530_A|G, 26577_C|G, 26709_G|A, 27259_A|C, 27807_C|T, 28271_A|T, 28311_C|T, 28362_28370, 28881_G|A, 28882_G|A, 28883_G|C, 29632_C|T

## Case 36 (1BP 5'): XG test: OK

Target: (75%) 305 samples
GT: BA.1* + BA.2*
BC: BA.1.17 + BA.2
Direction L1: <<
Alt. candidates: [], [BA.2.7, BA.2.9, BA.2.52, BA.2.25]
Model 1BP/2BP comparison: -
Rec. model vs L1: 1.05e-197
Flags: Model_2BP_Bad_L1_opp

Number of changes: 70
GT BR: 6-7
BC BR: 6-7
Initial region span: 1-6,7-70
Rec. model vs L2: 4.42e-49

GT BR coord: 5926 - 6512
BC BR coord: 5943 - 5944
Gap history (edge excluded):

Rank L1 L2: 11 1

## BA.1.17 >>

| | num_seq | t_ch_MAX | max_CL | CL@BC_t_ch_MAX | aic | PV | PV_OK | t_ch_MAX_OK | phyl_OK |
|---|---|---|---|---|---|---|---|---|---|
| **BA.1.17** | 6954 | 6 | 13.886461 | NaN | NaN | NaN | * | * | * |
| **BA.1.9** | 186 | 5 | 6.270221 | -3.729779 | 21.459557 | 2.238220e-08 | | * | |
| **BA.1.12** | 635 | 5 | 4.640693 | -5.359307 | 24.718615 | 4.385334e-09 | | * | |
| **BA.1.20** | 10512 | 5 | 4.460463 | -5.539537 | 25.079075 | 3.662939e-09 | | * | |
| **BA.1.10** | 459 | 5 | 4.273696 | -5.726304 | 25.452607 | 3.044284e-09 | | * | |
| **BA.1.6** | 60 | 4 | 3.950634 | -16.049366 | 46.098731 | 9.986072e-14 | | | |
| **BA.1.4** | 12 | 4 | 3.950634 | -16.049366 | 46.098731 | 9.986072e-14 | | | |
| **BA.1.24** | 96 | 4 | 3.950634 | -16.049366 | 46.098731 | 9.986072e-14 | | | |
| **BA.1.23** | 12 | 4 | 3.950634 | -16.049366 | 46.098731 | 9.986072e-14 | | | |
| **BC.1** | 209 | 4 | 3.950634 | -24.026135 | 62.052270 | 3.434758e-17 | | | |

## BA.2 <<

| | num_seq | t_ch_MAX | max_CL | CL@BC_t_ch_MAX | aic | PV | PV_OK | t_ch_MAX_OK | phyl_OK |
|---|---|---|---|---|---|---|---|---|---|
| **BA.2** | 359165 | 7 | 64.391320 | NaN | NaN | NaN | * | * | * |
| **BA.2.7** | 1628 | 7 | 63.897425 | 57.783193 | 32.433613 | 1.353583e-03 | * | * | * |
| **BA.2.9** | 61803 | 7 | 56.018223 | 56.018223 | 35.963555 | 2.317156e-04 | * | * | * |
| **BA.2.45** | 108 | 9 | 54.148954 | 51.246247 | 45.507506 | 1.955235e-06 | | | * |
| **BA.2.52** | 263 | 7 | 53.477722 | 53.477722 | 41.044556 | 1.827457e-05 | * | * | * |
| **BA.2.25** | 97 | 7 | 53.202049 | 53.202049 | 41.595902 | 1.381162e-05 | * | * | * |
| **BA.2.9.5** | 109 | 7 | 49.530868 | 49.530868 | 48.938264 | 3.518714e-07 | | * | * |
| **BA.2.9.2** | 355 | 7 | 47.436018 | 41.332849 | 65.334303 | 9.712717e-11 | | * | * |
| **BA.2.9.6** | 98 | 7 | 47.398559 | 47.398559 | 53.202883 | 4.181545e-08 | | * | * |
| **BA.2.22** | 2689 | 22 | 47.298860 | 39.483014 | 69.033972 | 1.527200e-11 | | | * |

Cumulative Likelihood per-region

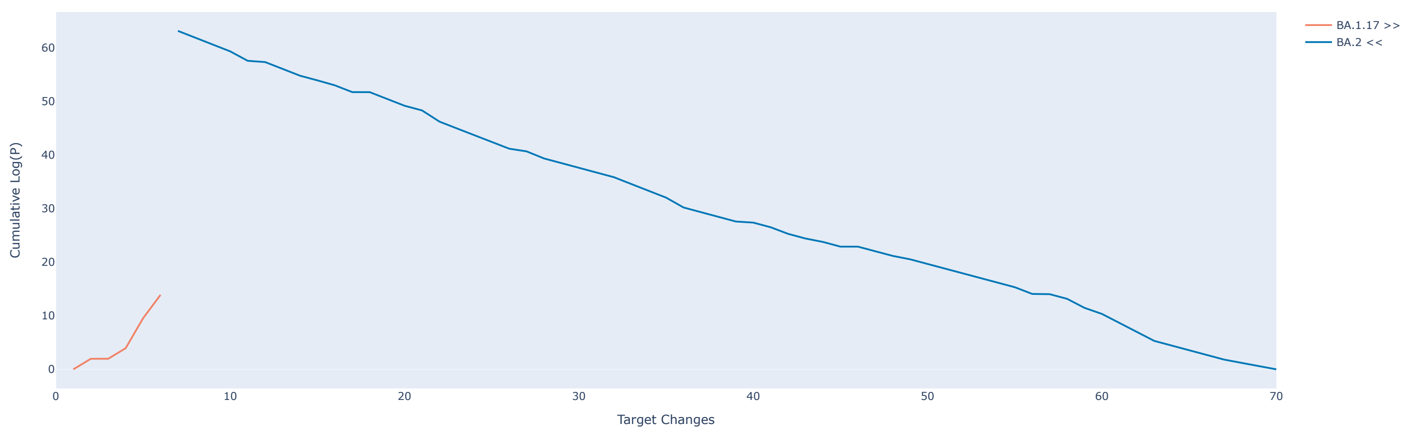


Cumulative Likelihood whole genome

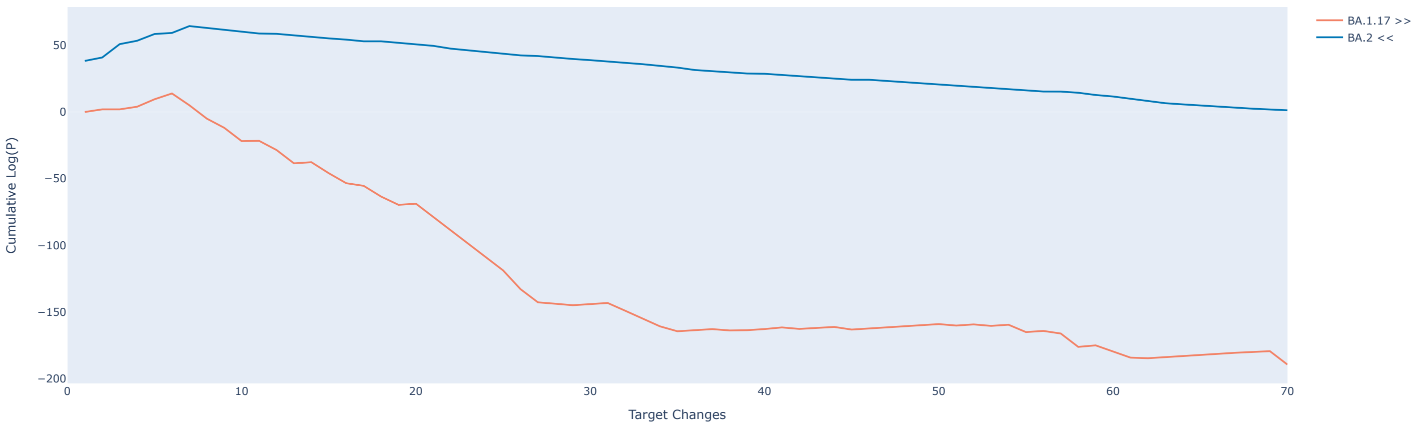


**Target sequence**

, 241_C|T, 2832_A|G, 3037_C|T, 5386_T|G, 5672_C|T, 5924_G|A, 9344_C|T, 9424_A|G, 9534_C|T, 9866_C|T, 10029_C|T, 10198_C|T, 10447_G|A, 10449_C|A, 11288_11296, 12880_C|T, 14408_C|T, 15714_C|T, 17410_C|T, 18163_A|G, 19855_A|G, 19955_C|T, 20055_A|G, 21618_C|T, 21633_21641, 21987_G|A, 22200_T|G, 22578_G|A, 22674_C|T, 22679_T|C, 22686_C|T, 22688_A|G, 22775_G|A, 22786_A|C, 22792_C|T, 22813_G|T, 22882_T|G, 22992_G|A, 22995_C|A, 23013_A|C, 23040_A|G, 23055_A|G, 23063_A|T, 23075_T|C, 23403_A|G, 23525_C|T, 23599_T|G, 23604_C|A, 23854_C|A, 23948_G|T, 24424_A|T, 24469_T|A, 25000_C|T, 25584_C|T, 26060_C|T, 26270_C|T, 26709_G|A, 26858_C|T, 27259_A|C, 27382_G|C, 27383_A|T, 27384_T|C, 27807_C|T, 28271_A|T, 28311_C|T, 28362_28370, 28881_G|A, 28882_G|A, 28883_G|C, 29510_A|C

## Case 37 (1BP 5'): XH          test: OK

Target: (75%) 102 samples
Number of changes: 68
GT: BA.1* + BA.2*
GT BR: 10–12
GT BR coord: 10447 – 11288
Rank L1 L2: 1 4
BC: BA.1 + BA.2.9
BC BR: 10–11
BC BR coord: 10203 – 10204
Direction L1: <<
Initial region span: 1–10,11–68
Gap history (edge excluded):
Alt. candidates: [], [BA.2.9.5, BA.2.9.7, BA.2]
Model 1BP/2BP comparison: –
Rec. model vs L1: 9.32e–148
Rec. model vs L2: 6.81e–61
Flags: Model_2BP_Bad_L1_opp

## BA.1 >>

| | num_seq | t_ch_MAX | max_CL | CL@BC_t_ch_MAX | aic | PV | PV_OK | t_ch_MAX_OK | phyl_OK |
|---|---|---|---|---|---|---|---|---|---|
| **BA.1** | 100089 | 10 | 7.365379 | NaN | NaN | NaN | * | * | * |
| **BA.1.17** | 6954 | 3 | 2.101600 | -4.145319 | 30.290637 | 0.00001 | * | | * |

## BA.2.9 <<

| | num_seq | t_ch_MAX | max_CL | CL@BC_t_ch_MAX | aic | PV | PV_OK | t_ch_MAX_OK | phyl_OK |
|---|---|---|---|---|---|---|---|---|---|
| **BA.2.9** | 61803 | 11 | 62.413528 | NaN | NaN | NaN | * | * | * |
| **BA.2.9.5** | 109 | 11 | 61.334105 | 60.059921 | 17.880158 | 3.395955e-01 | * | * | * |
| **BA.2.9.7** | 852 | 11 | 51.528794 | 50.254610 | 37.490779 | 1.873719e-05 | * | * | * |
| **BA.2** | 359165 | 11 | 51.085523 | 49.811584 | 38.376832 | 1.200725e-05 | * | * | * |
| **BA.2.9.2** | 355 | 11 | 47.035332 | 45.763969 | 46.472063 | 2.102434e-07 | | * | * |
| **BA.2.9.6** | 98 | 11 | 42.561553 | 41.287369 | 55.425262 | 2.382775e-09 | | * | * |
| **BA.2.7** | 1628 | 11 | 41.780955 | 40.507386 | 56.985228 | 1.092278e-09 | | * | |
| **BA.2.9.3** | 351 | 11 | 39.277298 | 38.003114 | 61.993771 | 8.965968e-11 | | * | * |
| **BA.2.3** | 22517 | 11 | 36.840888 | 35.566971 | 66.866059 | 7.814814e-12 | | * | |
| **B.1.1.529** | 248 | 13 | 36.813243 | 35.143825 | 67.712350 | 5.134698e-12 | | | * |

Cumulative Likelihood per-region

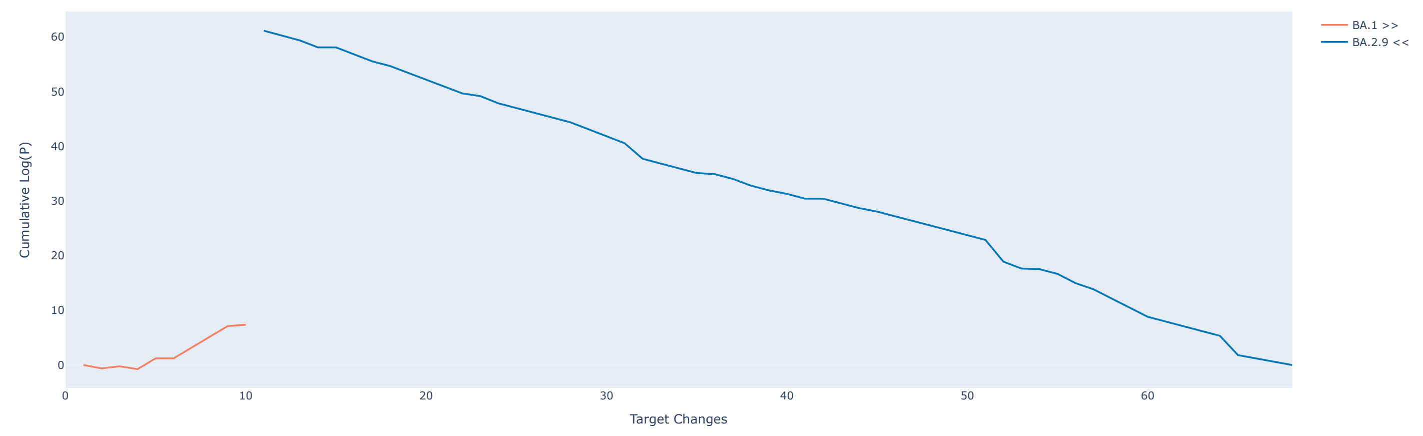


Cumulative Likelihood whole genome

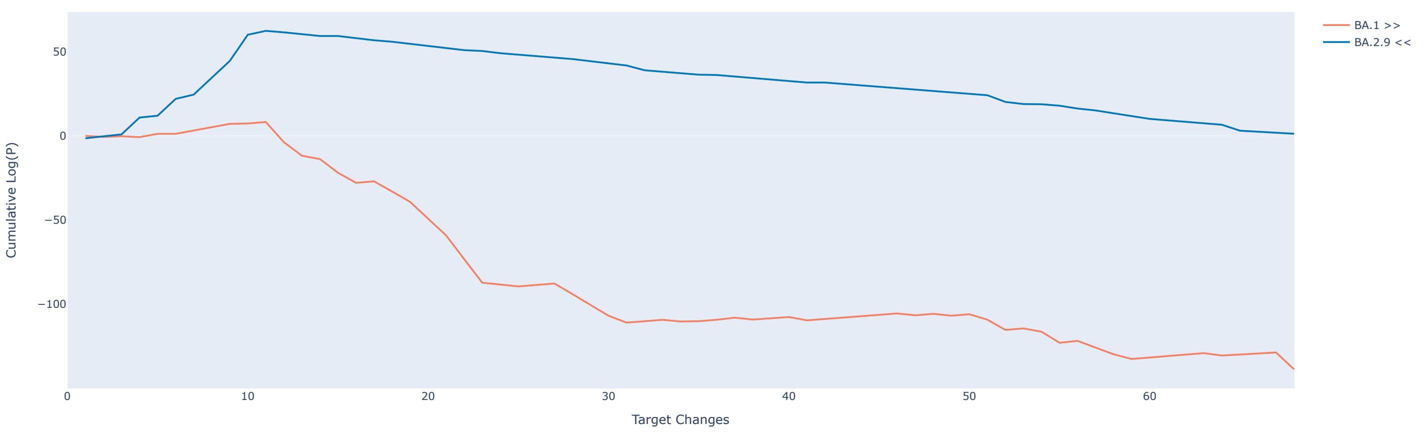


**Target sequence**

, 241_C|T, 902_T|C, 904_C|A, 1244_G|A, 2832_A|G, 3037_C|T, 5386_T|G, 6513_6515, 8393_G|A, 10029_C|T, 10449_C|A, 11288_11296, 12880_C|T, 14408_C|T, 15714_C|T, 17410_C|T, 18163_A|G, 19955_C|T, 20055_A|G, 21618_C|T, 21633_21641, 21987_G|A, 22200_T|G, 22578_G|A, 22674_C|T, 22679_T|C, 22686_C|T, 22688_A|G, 22775_G|A, 22786_A|C, 22792_C|T, 22813_G|T, 22882_T|G, 22992_G|A, 22995_C|A, 23013_A|C, 23040_A|G, 23055_A|G, 23063_A|T, 23075_T|C, 23403_A|G, 23525_C|T, 23599_T|G, 23604_C|A, 23854_C|A, 23948_G|T, 24424_A|T, 24469_T|A, 25000_C|T, 25584_C|T, 25624_C|T, 26060_C|T, 26270_C|T, 26709_G|A, 26858_C|T, 27259_A|C, 27382_G|C, 27383_A|T, 27384_T|C, 27807_C|T, 28271_A|T, 28311_C|T, 28362_28370, 28435_C|T, 28881_G|A, 28882_G|A, 28883_G|C, 29510_A|C

## Case 38 (1BP 5'): XL    test: OK

Target: (75%) 70 samples    Number of changes: 74
GT: BA.1* + BA.2*    GT BR: 8-9    GT BR coord: 6517 - 8393    Rank L1 L2: 7 1
BC: BA.1.17.2 + BA.2    BC BR: 8-9    BC BR coord: 6524 - 6525
Direction L1: <<    Initial region span: 1-8,10-74    Gap history (edge excluded): 8-10
Alt. candidates: [BA.1.17, BD.1, BA.1], [BA.2.9]
Model 1BP/2BP comparison: -
Rec. model vs L1: 1.91e-176    Rec. model vs L2: 1.83e-39
Flags: Model_2BP_Bad_L1_opp

## BA.1.17.2 >>

| | num_seq | t_ch_MAX | max_CL | CL@BC_t_ch_MAX | aic | PV | PV_OK | t_ch_MAX_OK | phyl_OK |
|---|---|---|---|---|---|---|---|---|---|
| **BA.1.17.2** | 39682 | 8 | 13.015662 | NaN | NaN | NaN | * | * | * |
| **BA.1.17** | 6954 | 8 | 11.155373 | 11.155373 | -4.310746 | 1.556726e-01 | * | * | * |
| **BD.1** | 2101 | 8 | 4.930538 | 4.930538 | 8.138923 | 3.081265e-04 | * | * | * |
| **BA.1.20** | 10512 | 4 | 3.707056 | -6.953867 | 31.907733 | 2.123925e-09 | | | |
| **BA.1.1.4** | 767 | 6 | 3.513847 | -4.515807 | 27.031614 | 2.436788e-08 | | | |
| **BA.1.15.1** | 9272 | 4 | 3.114080 | -1.102761 | 20.205522 | 7.374998e-07 | | | |
| **BA.1** | 100089 | 8 | 2.724089 | 2.724089 | 12.551821 | 3.397111e-05 | * | * | * |
| **BA.1.16** | 3129 | 4 | 1.432357 | -21.685066 | 61.370133 | 8.511017e-16 | | | |
| **BA.1.15** | 46393 | 4 | 0.805629 | -2.805747 | 23.611494 | 1.347291e-07 | | | |
| **BA.1.1.1** | 8304 | 6 | 0.459283 | -1.551479 | 21.102957 | 4.726078e-07 | | | |

## BA.2 <<

| | num_seq | t_ch_MAX | max_CL | CL@BC_t_ch_MAX | aic | PV | PV_OK | t_ch_MAX_OK | phyl_OK |
|---|---|---|---|---|---|---|---|---|---|
| **BA.2** | 359165 | 10 | 61.963517 | NaN | NaN | NaN | * | * | * |
| **BA.2.3** | 22517 | 10 | 59.024551 | 44.267983 | 57.464035 | 3.305807e-08 | | * | * |
| **BA.2.9** | 61803 | 10 | 56.237147 | 54.889886 | 36.220229 | 1.353583e-03 | * | * | * |
| **BA.2.23** | 8100 | 10 | 53.795038 | 43.795038 | 58.409924 | 2.055832e-08 | | * | * |
| **BA.2.26** | 580 | 10 | 53.662917 | 36.734543 | 72.530913 | 1.765504e-11 | | * | * |
| **BA.2.29** | 102 | 10 | 53.298123 | 43.298123 | 59.403754 | 1.253176e-08 | | * | * |
| **BA.2.27** | 19 | 10 | 53.257532 | 43.257532 | 59.484936 | 1.204038e-08 | | * | * |
| **BA.2.5** | 473 | 10 | 53.025531 | 43.025531 | 59.948938 | 9.518772e-09 | | * | * |
| **BA.2.1** | 7140 | 21 | 52.374239 | 38.740017 | 68.519967 | 1.311080e-10 | | | * |
| **BA.2.31** | 453 | 21 | 52.146648 | 36.608171 | 72.783659 | 1.558052e-11 | | | * |

Cumulative Likelihood per-region

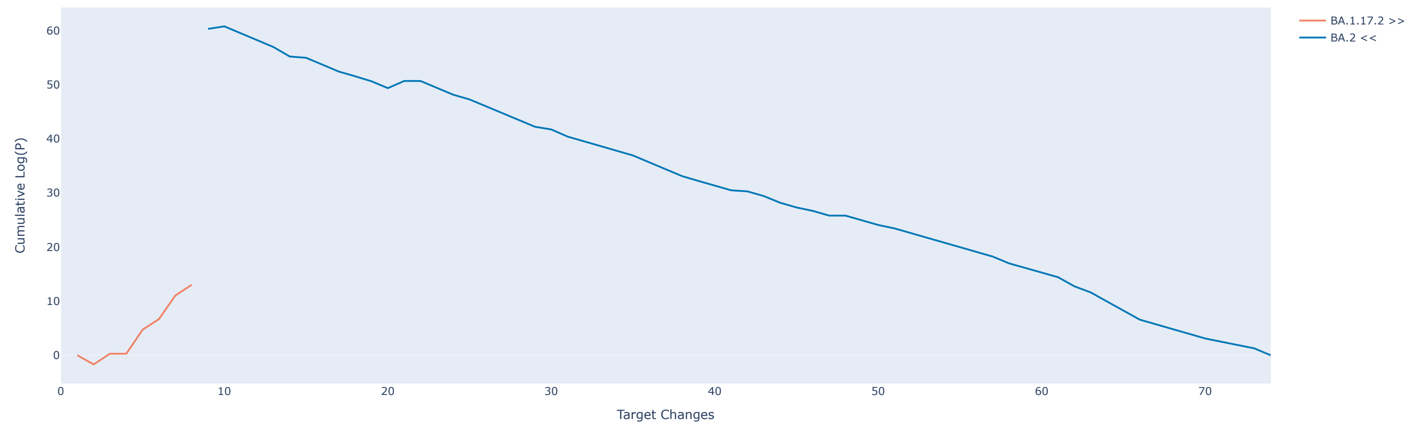


Cumulative Likelihood whole genome

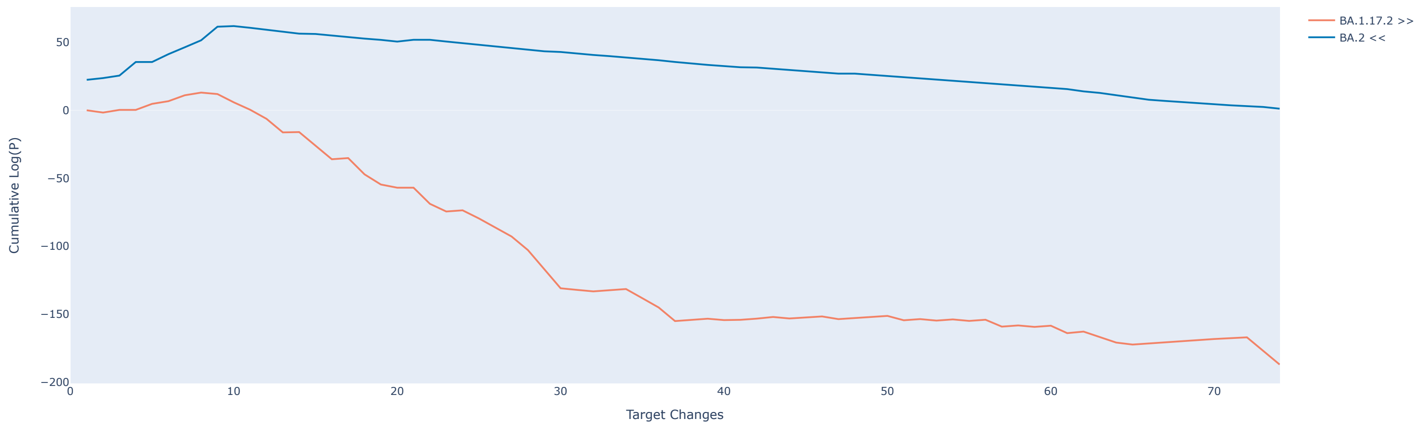


**Target sequence**

, 241_C|T, 875_C|T, 2832_A|G, 3037_C|T, 3241_C|T, 5386_T|G, 5924_G|A, 6513_6515, 9208_T|C, 9344_C|T, 9424_A|G, 9534_C|T, 9866_C|T, 10029_C|T, 10198_C|T, 10447_G|A, 10449_C|A, 11288_11296, 12880_C|T, 14229_G|A, 14408_C|T, 15714_C|T, 17410_C|T, 18163_A|G, 19955_C|T, 20055_A|G, 21618_C|T, 21633_21641, 21987_G|A, 22200_T|G, 22578_G|A, 22674_C|T, 22679_T|C, 22686_C|T, 22688_A|G, 22775_G|A, 22786_A|C, 22813_G|T, 22882_T|G, 22992_G|A, 22995_C|A, 23013_A|C, 23040_A|G, 23055_A|G, 23063_A|T, 23075_T|C, 23403_A|G, 23525_C|T, 23599_T|G, 23604_C|A, 23854_C|A, 23948_G|T, 24424_A|T, 24469_T|A, 25000_C|T, 25584_C|T, 26060_C|T, 26270_C|T, 26577_C|G, 26709_G|A, 26858_C|T, 27259_A|C, 27382_G|C, 27383_A|T, 27384_T|C, 27807_C|T, 28271_A|T, 28311_C|T, 28362_28370, 28881_G|A, 28882_G|A, 28883_G|C, 29510_A|C, 29734_29759

## Case 39 (1BP 5'): XN    test: KO

Target: (75%) 167 samples    Number of changes: 71
GT: BA.1* + BA.2*    GT BR: 2-4    GT BR coord: 2833 - 4184    Rank L1 L2: 11 -
BC: BA.2    BC BR:    BC BR coord:
Direction L1: <<    Initial region span: 3-71    Gap history (edge excluded):
Alt. candidates: [BA.2.9]
Model 1BP/2BP comparison:    -
Rec. model vs L1: -    Rec. model vs L2: -
Flags: NotEnoughSpaceAfterL1, SingleCandidateGenome

# BA.2 <<

| | num_seq | t_ch_MAX | max_CL | CL@BC_t_ch_MAX | aic | PV | PV_OK | t_ch_MAX_OK | phyl_OK |
|---|---|---|---|---|---|---|---|---|---|
| **BA.2** | 359165 | 3 | 69.094942 | NaN | NaN | NaN | * | * | * |
| **BA.2.9** | 61803 | 4 | 62.177283 | 49.620309 | 66.759381 | 9.878299e-04 | * | * | * |
| **BA.2.52** | 263 | 3 | 58.681572 | 37.473916 | 91.052167 | 5.250198e-09 | | * | * |
| **BA.2.25** | 97 | 3 | 58.120134 | 36.179118 | 93.641763 | 1.438018e-09 | | * | * |
| **BA.2.7** | 1628 | 15 | 57.051667 | 40.156482 | 85.687036 | 7.657463e-08 | | | * |
| **BA.2.9.2** | 355 | 15 | 52.797004 | 35.902823 | 94.194355 | 1.092278e-09 | | | * |
| **BA.2.9.5** | 109 | 15 | 52.366885 | 31.289277 | 103.421446 | 1.081594e-11 | | | * |
| **BA.2.22** | 2689 | 20 | 52.356527 | 33.652970 | 98.694060 | 1.151253e-10 | | | * |
| **BA.2.56** | 585 | 3 | 52.003593 | 39.446260 | 87.107481 | 3.764747e-08 | | * | * |
| **BA.2.36** | 1320 | 3 | 51.477318 | 38.915616 | 88.168768 | 2.215949e-08 | | * | * |

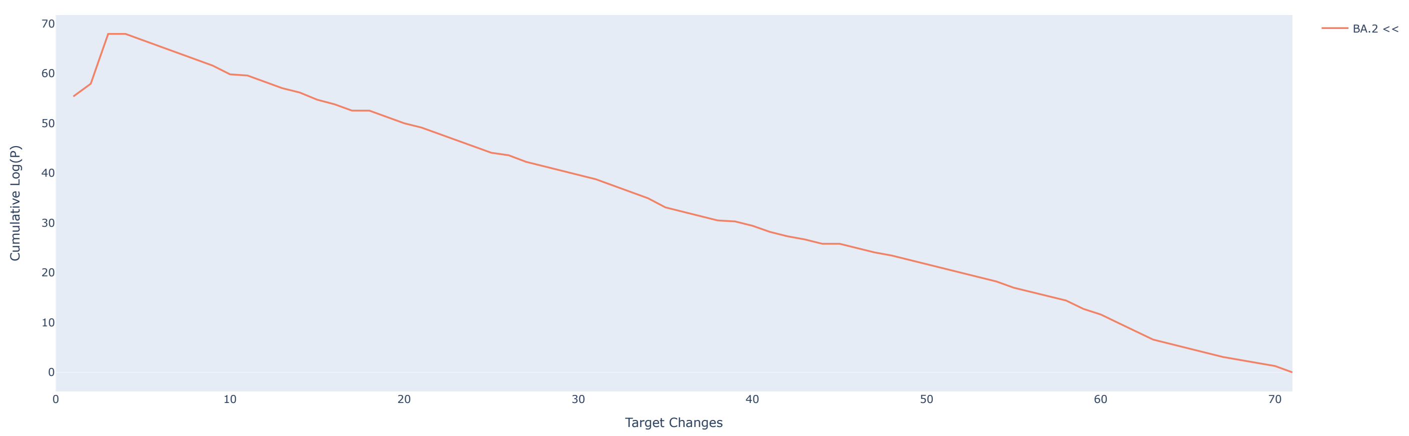


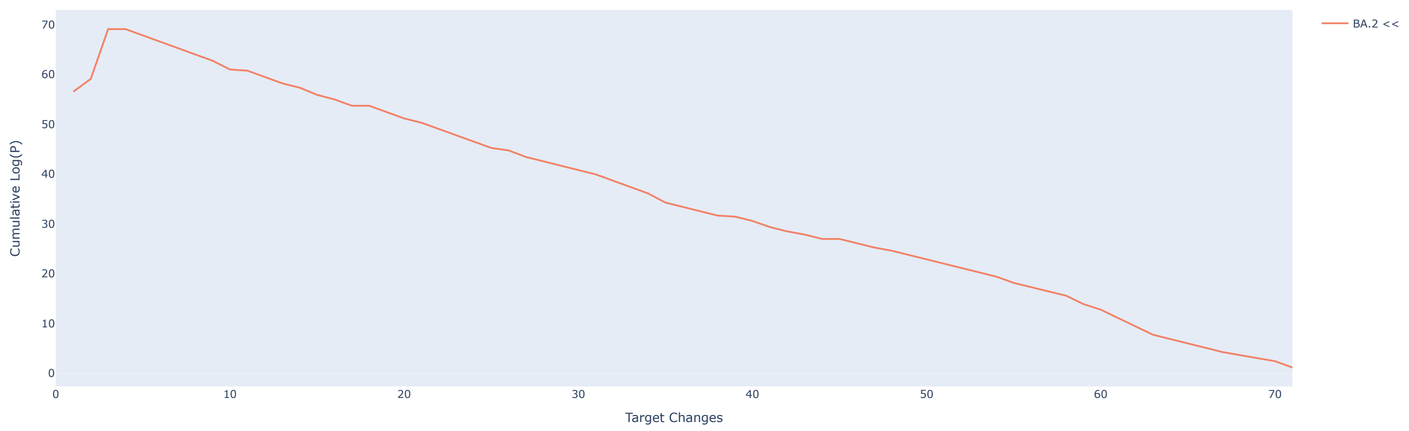


**Target sequence**

, 241_C|T, 2832_A|G, 3037_C|T, 4184_G|A, 4321_C|T, 9344_C|T, 9424_A|G, 9534_C|T, 9866_C|T, 10029_C|T, 10198_C|T, 10447_G|A, 10449_C|A, 10986_G|A, 11288_11296, 12880_C|T, 14408_C|T, 15714_C|T, 17410_C|T, 18163_A|G, 19955_C|T, 20055_A|G, 21618_C|T, 21633_21641, 21987_G|A, 22200_T|G, 22578_G|A, 22674_C|T, 22679_T|C, 22686_C|T, 22688_A|G, 22775_G|A, 22786_A|C, 22792_C|T, 22813_G|T, 22882_T|G, 22992_G|A, 22995_C|A, 23013_A|C, 23040_A|G, 23055_A|G, 23063_A|T, 23075_T|C, 23403_A|G, 23525_C|T, 23599_T|G, 23604_C|A, 23854_C|A, 23948_G|T, 24424_A|T, 24469_T|A, 25000_C|T, 25584_C|T, 26060_C|T, 26270_C|T, 26577_C|G, 26709_G|A, 26858_C|T, 27259_A|C, 27382_G|C, 27383_A|T, 27384_T|C, 27807_C|T, 28271_A|T, 28311_C|T, 28362_28370, 28881_G|A, 28882_G|A, 28883_G|C, 29510_A|C, 29734_29759

## Case 40 (1BP 5'): XQ test: OK

Target: (75%) 90 samples
Number of changes: 68
GT: BA.1.1* + BA.2*
GT BR: 4–5
GT BR coord: 4321 – 5386
Rank L1 L2: 8 4
BC: BA.1.1.9 + BA.2.23
BC BR: 4–5
BC BR coord: 4345 – 4346
Direction L1: <<
Initial region span: 1–4,5–68
Gap history (edge excluded):
Alt. candidates: [BA.1.1], [BA.2]
Model 1BP/2BP comparison: –
Rec. model vs L1: 0.00e+00
Rec. model vs L2: 1.32e–46
Flags: Model_2BP_Bad_L1_opp

## BA.1.1.9 >>

| | num_seq | t_ch_MAX | max_CL | CL@BC_t_ch_MAX | aic | PV | PV_OK | t_ch_MAX_OK | phyl_OK |
|---|---|---|---|---|---|---|---|---|---|
| **BA.1.1.9** | 172 | 4 | 4.532562 | NaN | NaN | NaN | * | * | * |
| **BA.1.1.6** | 22 | 4 | 4.532562 | 4.532562 | 0.934876 | 1.000000 | * | * | |
| **BA.1.1.15** | 5038 | 4 | 4.531768 | 4.531768 | 0.936464 | 0.995012 | * | * | |
| **BA.1.1.13** | 3393 | 4 | 4.531678 | 4.531678 | 0.936645 | 0.995012 | * | * | |
| **BA.1.1.12** | 1943 | 3 | 4.528654 | -2.974968 | 15.949936 | 0.000548 | * | * | |
| **BA.1.1.14** | 5810 | 4 | 4.528600 | 4.528600 | 0.942800 | 0.995012 | * | * | |
| **BA.1.1.2** | 2970 | 4 | 4.525482 | 4.525482 | 0.949037 | 0.990050 | * | * | |
| **BA.1.1** | 262925 | 4 | 4.522543 | 4.522543 | 0.954914 | 0.990050 | * | * | * |
| **BA.1.1.16** | 594 | 4 | 4.522435 | 4.522435 | 0.955129 | 0.985112 | * | * | |
| **BA.1.1.18** | 22283 | 4 | 4.519059 | 4.519059 | 0.961882 | 0.985112 | * | * | |

## BA.2.23 <<

| | num_seq | t_ch_MAX | max_CL | CL@BC_t_ch_MAX | aic | PV | PV_OK | t_ch_MAX_OK | phyl_OK |
|---|---|---|---|---|---|---|---|---|---|
| **BA.2.23** | 8100 | 5 | 63.795038 | NaN | NaN | NaN | * | * | * |
| **BA.2.26** | 580 | 5 | 63.662917 | 56.734543 | 30.530913 | 0.000859 | * | * | |
| **BA.2.29** | 102 | 5 | 63.298123 | 63.298123 | 17.403754 | 0.609571 | * | * | |
| **BA.2** | 359165 | 5 | 63.284593 | 63.284593 | 17.430814 | 0.600496 | * | * | * |
| **BA.2.27** | 19 | 5 | 63.257532 | 63.257532 | 17.484936 | 0.585669 | * | * | |
| **BA.2.5** | 473 | 5 | 63.025531 | 63.025531 | 17.948938 | 0.463013 | * | * | |
| **BA.2.11** | 22 | 5 | 61.897205 | 52.429430 | 39.141140 | 0.000012 | * | * | |
| **BA.2.37** | 2937 | 5 | 60.859482 | 53.676935 | 36.646130 | 0.000040 | * | * | |
| **BA.2.57** | 14 | 5 | 60.617841 | 51.368001 | 41.263997 | 0.000004 | | * | |
| **BA.2.7** | 1628 | 5 | 60.605494 | 54.491262 | 35.017476 | 0.000091 | * | * | |

Cumulative Likelihood per-region

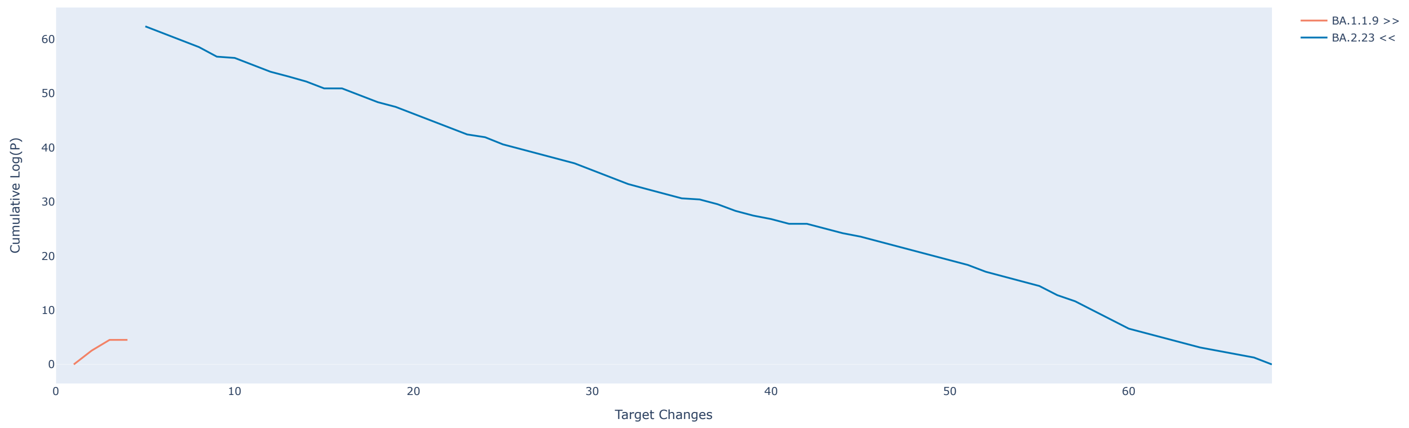


Cumulative Likelihood whole genome

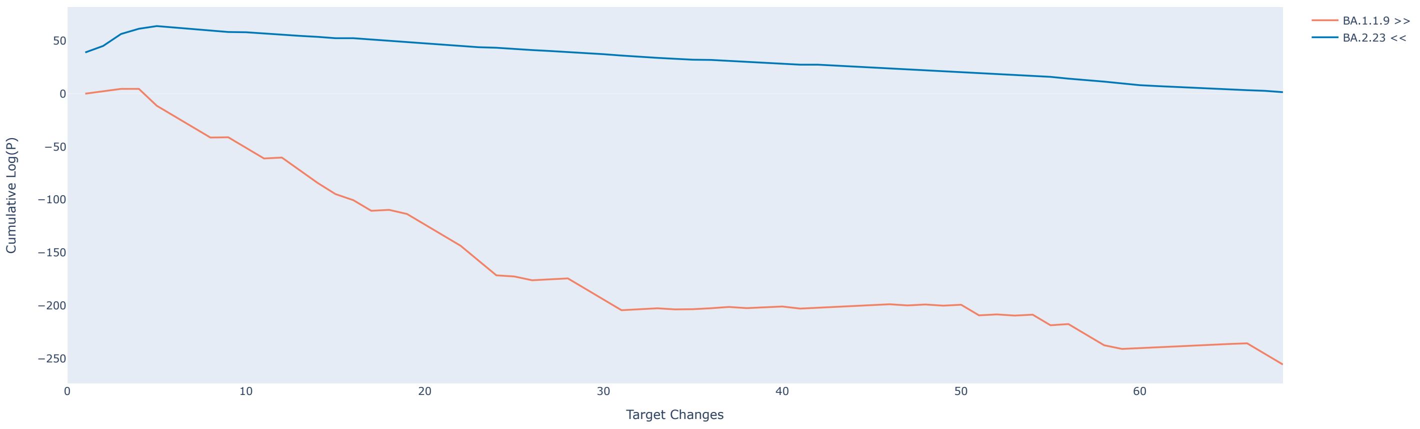


**Target sequence**

, 241_C|T, 2470_C|T, 2832_A|G, 3037_C|T, 9344_C|T, 9424_A|G, 9534_C|T, 9866_C|T, 10029_C|T, 10198_C|T, 10447_G|A, 10449_C|A, 11288_11296, 12880_C|T, 14408_C|T, 15714_C|T, 17410_C|T, 18163_A|G, 19955_C|T, 20055_A|G, 21618_C|T, 21633_21641, 21987_G|A, 22200_T|G, 22578_G|A, 22674_C|T, 22679_T|C, 22686_C|T, 22688_A|G, 22775_G|A, 22786_A|C, 22813_G|T, 22882_T|G, 22992_G|A, 22995_C|A, 23013_A|C, 23040_A|G, 23055_A|G, 23063_A|T, 23075_T|C, 23403_A|G, 23525_C|T, 23599_T|G, 23604_C|A, 23854_C|A, 23948_G|T, 24424_A|T, 24469_T|A, 25000_C|T, 25584_C|T, 26060_C|T, 26270_C|T, 26577_C|G, 26709_G|A, 26858_C|T, 27259_A|C, 27382_G|C, 27383_A|T, 27384_T|C, 27807_C|T, 28271_A|T, 28311_C|T, 28362_28370, 28881_G|A, 28882_G|A, 28883_G|C, 29510_A|C, 29734_29759

## Case 41 (1BP 5'): XR    test: OK

Target: (75%) 24 samples    Number of changes: 72
GT: BA.1.1* + BA.2*    GT BR: 4–5    GT BR coord: 4321 – 4892    Rank L1 L2: 8 2
BC: BA.1.1.15 + BA.2.9    BC BR: 5–6    BC BR coord: 4900 – 4901
Direction L1: <<    Initial region span: 1–5,6–72    Gap history (edge excluded):
Alt. candidates: [BA.1.1], [BA.2]
Model 1BP/2BP comparison:    –
Rec. model vs L1: 7.45e–280    Rec. model vs L2: 2.23e–32
Flags: Model_2BP_Bad_L1_opp

## BA.1.1.15 >>

| | num_seq | t_ch_MAX | max_CL | CL@BC_t_ch_MAX | aic | PV | PV_OK | t_ch_MAX_OK | phyl_OK |
|---|---|---|---|---|---|---|---|---|---|
| **BA.1.1.15** | 5038 | 5 | 4.623776 | NaN | NaN | NaN | * | * | * |
| **BA.1.1.9** | 172 | 4 | 4.532562 | -5.467438 | 22.934876 | 4.149241e-05 | * | * | |
| **BA.1.1.6** | 22 | 4 | 4.532562 | -5.467438 | 22.934876 | 4.149241e-05 | * | * | |
| **BA.1.1.13** | 3393 | 4 | 4.531678 | -5.468322 | 22.936645 | 4.128547e-05 | * | * | |
| **BA.1.1.12** | 1943 | 3 | 4.528654 | -12.974968 | 37.949936 | 2.272046e-08 | | | |
| **BA.1.1.14** | 5810 | 4 | 4.528600 | -5.471400 | 22.942800 | 4.128547e-05 | * | * | |
| **BA.1.1.2** | 2970 | 4 | 4.525482 | 2.948713 | 6.102575 | 1.873082e-01 | * | * | |
| **BA.1.1** | 262925 | 4 | 4.522543 | 3.207399 | 5.585202 | 2.417140e-01 | * | * | * |
| **BA.1.1.16** | 594 | 4 | 4.522435 | -5.477565 | 22.955129 | 4.087467e-05 | * | * | |
| **BA.1.1.18** | 22283 | 4 | 4.519059 | 2.536466 | 6.927069 | 1.236871e-01 | * | * | |

## BA.2.9 <<

| | num_seq | t_ch_MAX | max_CL | CL@BC_t_ch_MAX | aic | PV | PV_OK | t_ch_MAX_OK | phyl_OK |
|---|---|---|---|---|---|---|---|---|---|
| **BA.2.9** | 61803 | 6 | 54.092199 | NaN | NaN | NaN | * | * | * |
| **BA.2** | 359165 | 5 | 53.892490 | 52.039914 | 61.920173 | 1.287349e-01 | * | * | * |
| **BA.2.3** | 22517 | 6 | 47.886191 | 43.129622 | 79.740755 | 1.738331e-05 | * | * | |
| **BA.2.18** | 3723 | 6 | 46.068781 | 46.068781 | 73.862438 | 3.288200e-04 | * | * | |
| **BA.2.23** | 8100 | 6 | 44.689988 | 44.689988 | 76.620024 | 8.272407e-05 | * | * | |
| **BA.2.10** | 12936 | 19 | 38.809900 | 37.437807 | 91.124386 | 5.874851e-08 | | | |
| **BA.2.12.1** | 94944 | 18 | 36.760315 | 30.933497 | 104.133006 | 8.788429e-11 | | | |
| **BA.2.38** | 706 | 6 | 36.088956 | 36.088956 | 93.822089 | 1.522998e-08 | | * | |
| **BA.2.26** | 580 | 6 | 33.662917 | 26.734543 | 112.530913 | 1.317876e-12 | | * | |
| **BA.2.32** | 346 | 6 | 33.577806 | 33.577806 | 98.844389 | 1.237714e-09 | | * | |

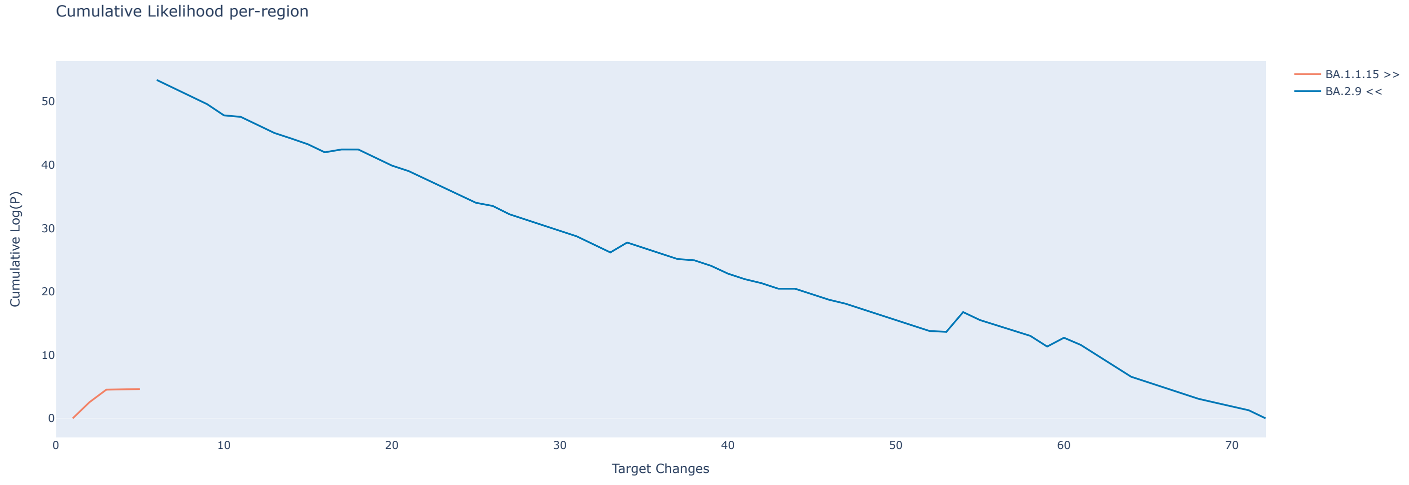


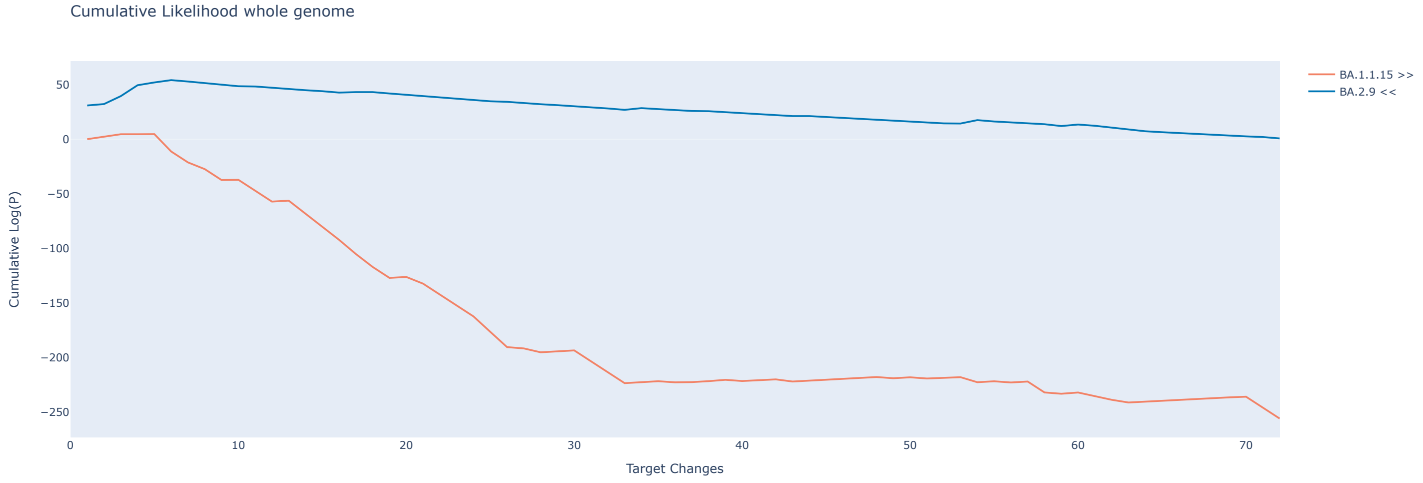


**Target sequence**

, 241_C|T, 2470_C|T, 2832_A|G, 3037_C|T, 4893_C|T, 9344_C|T, 9424_A|G, 9534_C|T, 9866_C|T, 10029_C|T, 10198_C|T, 10447_G|A, 10449_C|A, 11288_11296, 12880_C|T, 13295_G|A, 14408_C|T, 15714_C|T, 17410_C|T, 18163_A|G, 19955_C|T, 20055_A|G, 21618_C|T, 21633_21641, 21987_G|A, 22200_T|G, 22578_G|A, 22674_C|T, 22679_T|C, 22686_C|T, 22688_A|G, 22775_G|A, 22786_A|C, 22813_G|T, 22882_T|G, 22992_G|A, 22995_C|A, 23013_A|C, 23040_A|G, 23055_A|G, 23063_A|T, 23075_T|C, 23403_A|G, 23525_C|T, 23599_T|G, 23604_C|A, 23854_C|A, 23948_G|T, 24424_A|T, 24469_T|A, 25000_C|T, 25524_A|G, 25584_C|T, 26060_C|T, 26270_C|T, 26577_C|G, 26709_G|A, 26858_C|T, 27131_C|T, 27259_A|C, 27382_G|C, 27383_A|T, 27384_T|C, 27807_C|T, 28271_A|T, 28311_C|T, 28362_28370, 28881_G|A, 28882_G|A, 28883_G|C, 29510_A|C, 29734_29759

## Case 42 (1BP 5'): XS test: OK

Target: (75%) 24 samples Number of changes: 68
GT: B.1.617.2* + BA.1.1* GT BR: 10–12 GT BR coord: 9054 – 10448 Rank L1 L2: 11 1
BC: AY.126 + BA.1.1 BC BR: 10–11 BC BR coord: 9071 – 9072
Direction L1: << Initial region span: 1–10,11–68 Gap history (edge excluded):
Alt. candidates: [], [BA.1.1.1]
Model 1BP/2BP comparison: –
Rec. model vs L1: 0.00e+00 Rec. model vs L2: 3.01e–68
Flags: Model_2BP_Bad_L1_opp

## AY.126 >>

| | num_seq | t_ch_MAX | max_CL | CL@BC_t_ch_MAX | aic | PV | PV_OK | t_ch_MAX_OK | phyl_OK |
|---|---|---|---|---|---|---|---|---|---|
| **AY.126** | 9654 | 10 | 7.685116 | NaN | NaN | NaN | * | * | * |
| **AY.3** | 79233 | 10 | 7.577773 | 7.577773 | 4.844454 | 0.900325 | * | * | |
| **AY.107** | 1693 | 10 | 6.971639 | 6.971639 | 6.056723 | 0.489192 | * | * | |
| **AY.43** | 72949 | 10 | 5.381005 | 5.381005 | 9.237990 | 0.099759 | * | * | |
| **AY.25** | 71546 | 10 | 5.374838 | 5.374838 | 9.250324 | 0.099261 | * | * | |
| **AY.39.1** | 4652 | 10 | 5.302162 | 5.302162 | 9.395676 | 0.092089 | * | * | |
| **AY.36** | 5902 | 10 | 4.949264 | 4.949264 | 10.101473 | 0.064894 | * | * | |
| **AY.113** | 4809 | 10 | 4.833379 | 4.833379 | 10.333242 | 0.057844 | * | * | |
| **AY.129** | 5456 | 10 | 4.680102 | 4.680102 | 10.639795 | 0.049539 | * | * | |
| **AY.103** | 149086 | 10 | 4.333242 | 4.333242 | 11.333516 | 0.035084 | * | * | |

## BA.1.1 <<

| | num_seq | t_ch_MAX | max_CL | CL@BC_t_ch_MAX | aic | PV | PV_OK | t_ch_MAX_OK | phyl_OK |
|---|---|---|---|---|---|---|---|---|---|
| **BA.1.1** | 262925 | 11 | 63.635116 | NaN | NaN | NaN | * | * | * |
| **BA.1.1.10** | 592 | 20 | 55.200924 | 49.183088 | 27.633825 | 5.302061e-07 | | | * |
| **BA.1.1.7** | 168 | 20 | 54.748507 | 54.490540 | 17.018920 | 1.067522e-04 | * | | * |
| **BA.1.1.9** | 172 | 20 | 54.427624 | 45.618091 | 34.763818 | 1.500323e-08 | | | * |
| **BA.1.1.12** | 1943 | 21 | 53.987114 | 51.143816 | 23.712367 | 3.764107e-06 | | | * |
| **BA.1.1.17** | 191 | 21 | 53.798191 | 51.655614 | 22.688771 | 6.268334e-06 | | | * |
| **BA.1.1.1** | 8304 | 11 | 53.701609 | 53.701609 | 18.596782 | 4.844894e-05 | * | * | * |
| **BA.1.1.14** | 5810 | 21 | 53.646647 | 43.662025 | 38.675951 | 2.113332e-09 | | | * |
| **BA.1.1.2** | 2970 | 21 | 53.565289 | 42.588438 | 40.823125 | 7.248909e-10 | | | * |
| **BA.1.1.18** | 22283 | 21 | 53.262052 | 51.234422 | 23.531157 | 4.118589e-06 | | | * |

Cumulative Likelihood per-region

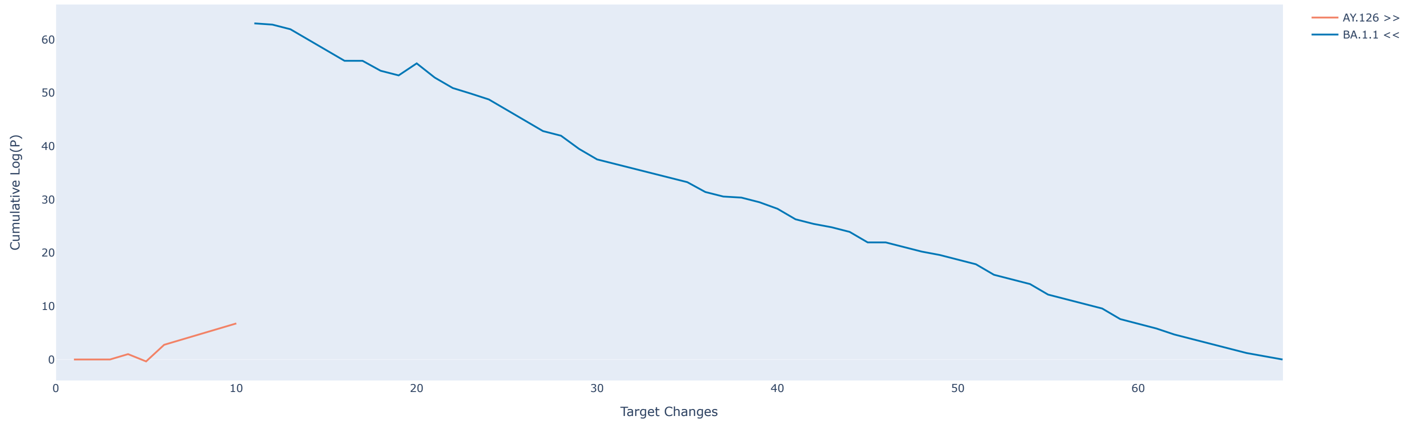


Cumulative Likelihood whole genome

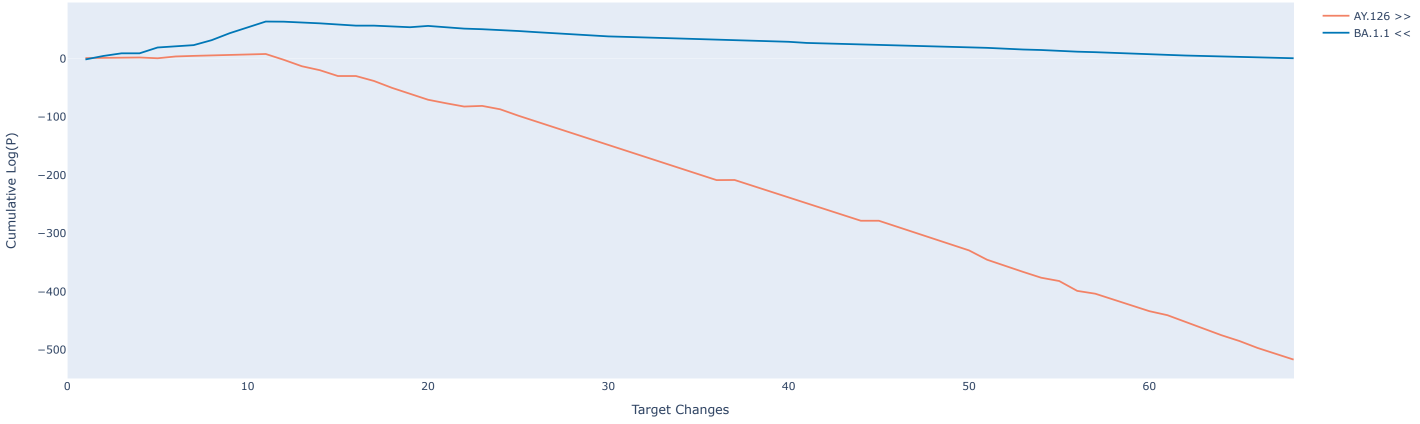


**Target sequence**

, 210_G|T, 241_C|T, 3037_C|T, 4181_G|T, 5365_C|T, 6196_C|T, 6402_C|T, 7124_C|T, 8986_C|T, 9053_G|T, 10029_C|T, 10449_C|A, 11285_11293, 11537_A|G, 13195_T|C, 14408_C|T, 15240_C|T, 18163_A|G, 18986_T|C, 21595_C|T, 21762_C|T, 21765_21770, 21846_C|T, 21987_21995, 22194_22196, 22204_.|GAGCCAGAA, 22578_G|A, 22599_G|A, 22673_T|C, 22674_C|T, 22679_T|C, 22686_C|T, 22813_G|T, 22882_T|G, 22898_G|A, 22992_G|A, 22995_C|A, 23013_A|C, 23040_A|G, 23048_G|A, 23055_A|G, 23063_A|T, 23075_T|C, 23202_C|A, 23403_A|G, 23525_C|T, 23599_T|G, 23604_C|A, 23854_C|A, 23948_G|T, 24130_C|A, 24424_A|T, 24469_T|A, 24503_C|T, 25000_C|T, 25584_C|T, 26270_C|T, 26530_A|G, 26577_C|G, 26709_G|A, 27259_A|C, 27807_C|T, 28271_A|T, 28311_C|T, 28362_28370, 28881_G|A, 28882_G|A, 28883_G|C

## Case 43 (1BP 5'): XU     test: OK

Target: (75%) 6 samples | Number of changes: 71
GT: BA.1* + BA.2* | GT BR: 6–7 | GT BR coord: 6517 – 9344 | Rank L1 L2: 4 1
BC: BA.1.10 + BA.2 | BC BR: 6–7 | BC BR coord: 6524 – 6525
Direction L1: << | Initial region span: 1–6,7–71 | Gap history (edge excluded):
Alt. candidates: [BA.1], [BA.2.9]
Model 1BP/2BP comparison: –
Rec. model vs L1: 2.80e–279 | Rec. model vs L2: 4.38e–47
Flags: Model_2BP_Bad_L1_opp

## BA.1.10 >>

| | num_seq | t_ch_MAX | max_CL | CL@BC_t_ch_MAX | aic | PV | PV_OK | t_ch_MAX_OK | phyl_OK |
|---|---|---|---|---|---|---|---|---|---|
| **BA.1.10** | 459 | 6 | 7.365188 | NaN | NaN | NaN | * | * | * |
| **BA.1.1.1** | 8304 | 6 | 6.702380 | 6.702380 | -1.404759 | 0.514274 | * | * | |
| **BA.1.14.1** | 181 | 6 | 6.677373 | 6.677373 | -1.354745 | 0.501576 | * | * | |
| **BA.1** | 100089 | 6 | 6.554483 | 6.554483 | -1.108967 | 0.444858 | * | * | * |
| **BA.1.15.2** | 840 | 6 | 6.239066 | 6.239066 | -0.478131 | 0.324652 | * | * | |
| **BA.1.21** | 556 | 6 | 6.232197 | 6.232197 | -0.464393 | 0.321422 | * | * | |
| **BA.1.12** | 635 | 6 | 6.124927 | 6.124927 | -0.249854 | 0.289384 | * | * | |
| **BA.1.14** | 1016 | 6 | 6.051119 | 6.051119 | -0.102238 | 0.268474 | * | * | |
| **BA.1.15.1** | 9272 | 6 | 6.037151 | 6.037151 | -0.074303 | 0.264477 | * | * | |
| **BA.1.19** | 691 | 6 | 6.023117 | 6.023117 | -0.046234 | 0.261846 | * | * | |

## BA.2 <<

| | num_seq | t_ch_MAX | max_CL | CL@BC_t_ch_MAX | aic | PV | PV_OK | t_ch_MAX_OK | phyl_OK |
|---|---|---|---|---|---|---|---|---|---|
| **BA.2** | 359165 | 7 | 60.467765 | NaN | NaN | NaN | * | * | * |
| **BA.2.1** | 7140 | 19 | 49.818690 | 36.184468 | 71.631064 | 2.838955e-11 | | | * |
| **BA.2.23** | 8100 | 19 | 49.789826 | 41.235754 | 61.528492 | 4.429408e-09 | | | * |
| **BA.2.26** | 580 | 19 | 49.681869 | 34.186382 | 75.627235 | 3.842108e-12 | | | * |
| **BA.2.31** | 453 | 19 | 49.606844 | 34.068367 | 75.863267 | 3.424725e-12 | | | * |
| **BA.2.9** | 61803 | 7 | 49.583049 | 49.583049 | 44.833903 | 1.873719e-05 | * | * | * |
| **BA.2.29** | 102 | 19 | 49.285870 | 40.736121 | 62.527758 | 2.686571e-09 | | | * |
| **BA.2.27** | 19 | 19 | 49.245279 | 40.695530 | 62.608939 | 2.581230e-09 | | | * |
| **BA.2.5** | 473 | 19 | 49.090318 | 40.523620 | 62.952761 | 2.177693e-09 | | | * |
| **BA.2.10** | 12936 | 21 | 48.395885 | 37.023792 | 69.952416 | 6.576062e-11 | | | * |

Cumulative Likelihood per-region

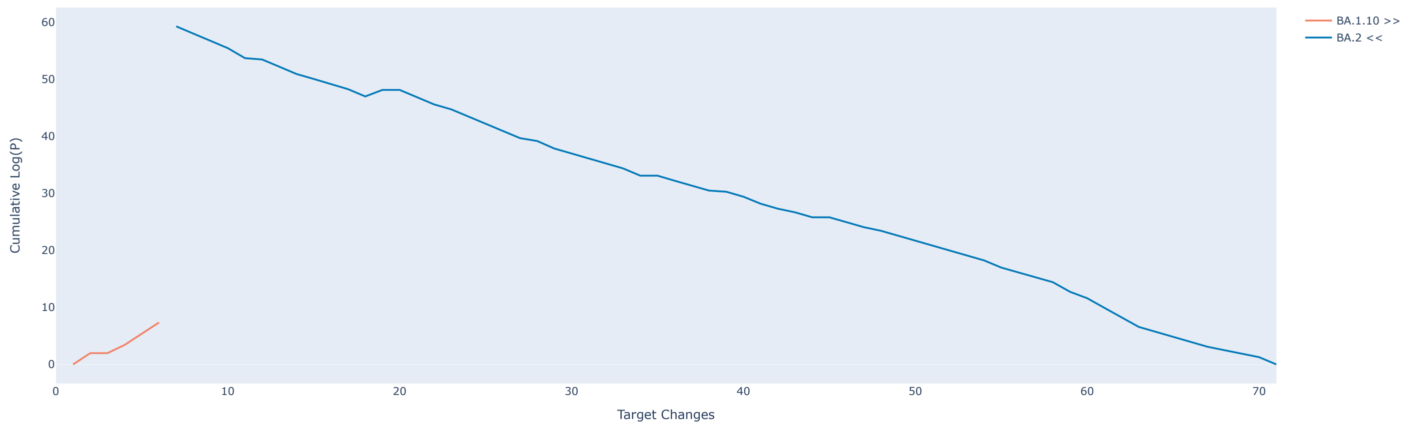


Cumulative Likelihood whole genome

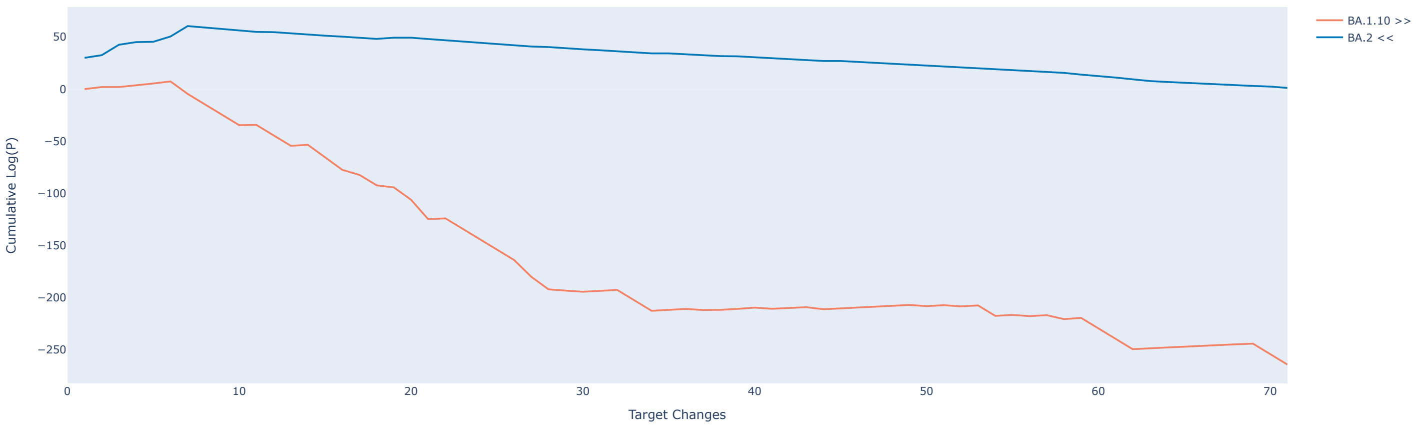


**Target sequence**

, 241_C|T, 2832_A|G, 3037_C|T, 4579_T|A, 5386_T|G, 6513_6515, 9344_C|T, 9424_A|G, 9534_C|T, 9866_C|T, 10029_C|T, 10198_C|T, 10447_G|A, 10449_C|A, 11288_11296, 12832_G|T, 12880_C|T, 13026_C|T, 14408_C|T, 15714_C|T, 17410_C|T, 18163_A|G, 19955_C|T, 20055_A|G, 21618_C|T, 21633_21641, 21987_G|A, 22200_T|G, 22578_G|A, 22674_C|T, 22679_T|C, 22686_C|T, 22688_A|G, 22775_G|A, 22813_G|T, 22882_T|G, 22992_G|A, 22995_C|A, 23013_A|C, 23040_A|G, 23055_A|G, 23063_A|T, 23075_T|C, 23403_A|G, 23525_C|T, 23599_T|G, 23604_C|A, 23854_C|A, 23948_G|T, 24424_A|T, 24469_T|A, 25000_C|T, 25584_C|T, 26060_C|T, 26270_C|T, 26577_C|G, 26709_G|A, 26858_C|T, 27259_A|C, 27382_G|C, 27383_A|T, 27384_T|C, 27807_C|T, 28271_A|T, 28311_C|T, 28362_28370, 28881_G|A, 28882_G|A, 28883_G|C, 29510_A|C, 29734_29759

## Case 44 (1BP 5'): XW     test: OK

Target: (75%) 47 samples
GT: BA.1* + BA.2*
BC: BA.1.1.9 + BA.2
Direction L1: <<
Alt. candidates: [BA.1.1], [BA.2.23]
Model 1BP/2BP comparison: -
Rec. model vs L1: 2.13e-306
Flags: Model_2BP_Bad_L1_opp

Number of changes: 72
GT BR: 3-5
BC BR: 3-4
Initial region span: 1-3,4-72
Rec. model vs L2: 1.85e-25

GT BR coord: 2833 - 4184
BC BR coord: 2835 - 2836
Gap history (edge excluded):

Rank L1 L2: 11 1

## BA.1.1.9 >>

| | num_seq | t_ch_MAX | max_CL | CL@BC_t_ch_MAX | aic | PV | PV_OK | t_ch_MAX_OK | phyl_OK |
|---|---|---|---|---|---|---|---|---|---|
| **BA.1.1.9** | 172 | 3 | 4.528654 | NaN | NaN | NaN | * | * | * |
| **BA.1.1.6** | 22 | 3 | 4.528654 | 4.528654 | -3.057307 | 1.000000 | * | * | |
| **BA.1.1.12** | 1943 | 3 | 4.528654 | 4.528654 | -3.057307 | 1.000000 | * | * | |
| **BA.1.1.15** | 5038 | 3 | 4.528058 | 4.528058 | -3.056116 | 1.000000 | * | * | |
| **BA.1.1.13** | 3393 | 3 | 4.527769 | 4.527769 | -3.055539 | 1.000000 | * | * | |
| **BA.1.1.14** | 5810 | 3 | 4.524864 | 4.524864 | -3.049727 | 0.995012 | * | * | |
| **BA.1.1.2** | 2970 | 3 | 4.521573 | 4.521573 | -3.043146 | 0.990050 | * | * | |
| **BA.1.1** | 262925 | 3 | 4.518882 | 4.518882 | -3.037763 | 0.990050 | * | * | * |
| **BA.1.1.16** | 594 | 3 | 4.518527 | 4.518527 | -3.037054 | 0.990050 | * | * | |
| **BA.1.1.18** | 22283 | 3 | 4.515195 | 4.515195 | -3.030391 | 0.985112 | * | * | |

## BA.2 <<

| | num_seq | t_ch_MAX | max_CL | CL@BC_t_ch_MAX | aic | PV | PV_OK | t_ch_MAX_OK | phyl_OK |
|---|---|---|---|---|---|---|---|---|---|
| **BA.2** | 359165 | 4 | 61.817721 | NaN | NaN | NaN | * | * | * |
| **BA.2.23** | 8100 | 4 | 55.921911 | 55.921911 | 42.156178 | 2.739445e-03 | * | * | * |
| **BA.2.1** | 7140 | 21 | 51.101110 | 36.724457 | 80.551086 | 1.262932e-11 | | | * |
| **BA.2.26** | 580 | 21 | 50.958627 | 25.736644 | 102.526713 | 2.130510e-16 | | | * |
| **BA.2.10** | 12936 | 21 | 50.942678 | 44.934309 | 64.131381 | 4.644486e-08 | | | * |
| **BA.2.31** | 453 | 21 | 50.873519 | 25.605846 | 102.788308 | 1.870791e-16 | | | * |
| **BA.2.29** | 102 | 21 | 50.574743 | 32.300223 | 89.399554 | 1.512261e-13 | | | * |
| **BA.2.27** | 19 | 21 | 50.534152 | 23.190808 | 107.618383 | 1.671876e-17 | | | * |
| **BA.2.19** | 114 | 21 | 50.405649 | 28.033548 | 97.932903 | 2.125010e-15 | | | * |
| **BA.2.5** | 473 | 21 | 50.319100 | 33.793459 | 86.413082 | 6.743683e-13 | | | * |

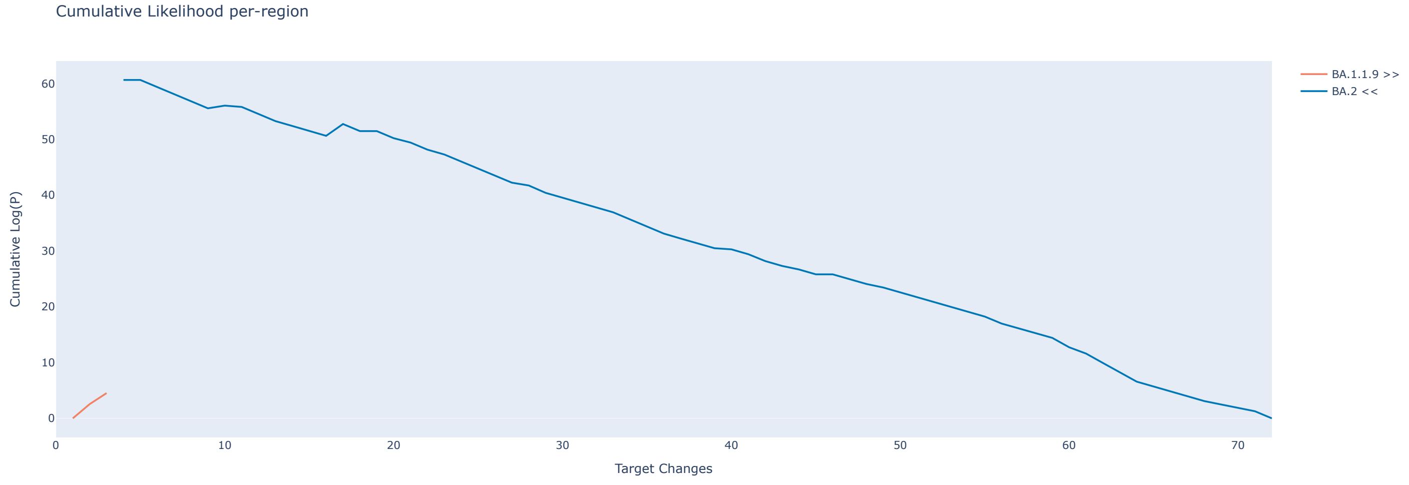


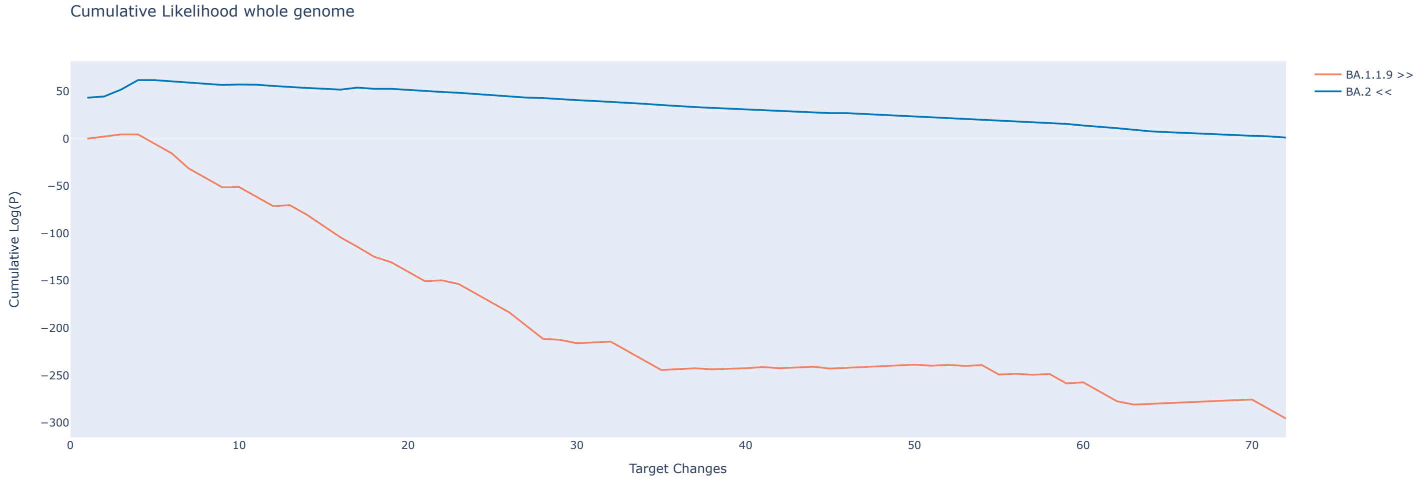


**Target sequence**

, 241_C|T, 2470_C|T, 2832_A|G, 3037_C|T, 4184_G|A, 4321_C|T, 9344_C|T, 9424_A|G, 9534_C|T, 10029_C|T, 10198_C|T, 10447_G|A, 10449_C|A, 10507_C|T, 11288_11296, 12756_C|T, 12880_C|T, 14408_C|T, 15714_C|T, 16020_G|T, 17410_C|T, 18163_A|G, 19955_C|T, 20055_A|G, 21618_C|T, 21633_21641, 21987_G|A, 22200_T|G, 22578_G|A, 22674_C|T, 22679_T|C, 22686_C|T, 22688_A|G, 22775_G|A, 22786_A|C, 22813_G|T, 22882_T|G, 22992_G|A, 22995_C|A, 23013_A|C, 23040_A|G, 23055_A|G, 23063_A|T, 23075_T|C, 23403_A|G, 23525_C|T, 23599_T|G, 23604_C|A, 23854_C|A, 23948_G|T, 24424_A|T, 24469_T|A, 25000_C|T, 25584_C|T, 26060_C|T, 26270_C|T, 26577_C|G, 26709_G|A, 26858_C|T, 27259_A|C, 27382_G|C, 27383_A|T, 27384_T|C, 27807_C|T, 28271_A|T, 28311_C|T, 28362_28370, 28881_G|A, 28882_G|A, 28883_G|C, 29510_A|C, 29734_29759

## Case 45 (1BP 3'): XAH    test: OK

Target: (75%) 1 samples
Number of changes: 71
GT: BA.2* + BA.1*
GT BR: 63–65
GT BR coord: 26858 – 27382
Rank L1 L2: 1 11
BC: BA.2 + BA.1.1.18
BC BR: 58–59
BC BR coord: 26061 – 26062
Direction L1: >>
Initial region span: 1–58,59–71
Gap history (edge excluded):
Alt. candidates: [BA.2.3], [BA.1.1]
Model 1BP/2BP comparison: 1BP vs 2BP: 8.09e–14
Rec. model vs L1: 1.33e–22
Rec. model vs L2: 1.04e–219
Flags: Model_1BP_Best

## BA.2 >>

| | num_seq | t_ch_MAX | max_CL | CL@BC_t_ch_MAX | aic | PV | PV_OK | t_ch_MAX_OK | phyl_OK |
|---|---|---|---|---|---|---|---|---|---|
| **BA.2** | 359165 | 58 | 47.575434 | NaN | NaN | NaN | * | * | * |
| **BA.2.3** | 22517 | 57 | 42.117949 | 38.515569 | 82.968863 | 1.162230e-04 | * | * | * |
| **BA.2.9** | 61803 | 57 | 35.560597 | 32.832940 | 94.334120 | 3.967339e-07 | | * | * |
| **BA.2.65** | 815 | 58 | 34.708631 | 34.708631 | 90.582738 | 2.587030e-06 | | * | * |
| **BA.2.56** | 585 | 58 | 31.728373 | 31.728373 | 96.543254 | 1.314026e-07 | | * | * |
| **BA.2.10** | 12936 | 60 | 28.915311 | 27.796535 | 104.406929 | 2.568356e-09 | | | * |
| **BA.2.12.1** | 94944 | 56 | 22.285852 | 21.497937 | 117.004125 | 4.739924e-12 | | | * |
| **BA.5.2.1** | 58578 | 43 | 22.128422 | 21.131560 | 117.736880 | 3.274028e-12 | | | |
| **BA.2.13.1** | 448 | 20 | 22.063780 | -6.554496 | 173.108992 | 3.101985e-24 | | | * |
| **BA.2.44** | 80 | 20 | 21.108478 | -13.735567 | 187.471134 | 2.362682e-27 | | | * |

## BA.1.1.18 <<

| | num_seq | t_ch_MAX | max_CL | CL@BC_t_ch_MAX | aic | PV | PV_OK | t_ch_MAX_OK | phyl_OK |
|---|---|---|---|---|---|---|---|---|---|
| **BA.1.1.18** | 22283 | 59 | 12.128775 | NaN | NaN | NaN | * | * | * |
| **BA.1.1** | 262925 | 59 | 11.320089 | 11.320089 | 5.359822 | 4.448581e-01 | * | * | * |
| **BA.1.5** | 274 | 60 | 11.094536 | 1.094536 | 25.810928 | 1.612725e-05 | * | * | |
| **BA.1.23** | 12 | 60 | 11.094536 | 1.094536 | 25.810928 | 1.612725e-05 | * | * | |
| **BA.1.1.12** | 1943 | 60 | 11.089900 | 1.089900 | 25.820200 | 1.604682e-05 | * | * | |
| **BC.1** | 209 | 60 | 11.089740 | 1.089740 | 25.820521 | 1.604682e-05 | * | * | |
| **BA.1.9** | 186 | 60 | 11.083754 | 1.083754 | 25.832492 | 1.596678e-05 | * | * | |
| **BA.1.1.13** | 3393 | 60 | 11.083326 | -5.106080 | 38.212159 | 3.272914e-08 | | * | |
| **BA.1.1.17** | 191 | 60 | 11.073455 | 1.073455 | 25.853090 | 1.580791e-05 | * | * | |
| **BA.1.10** | 459 | 60 | 11.072687 | 1.072687 | 25.854625 | 1.580791e-05 | * | * | |

Cumulative Likelihood per-region

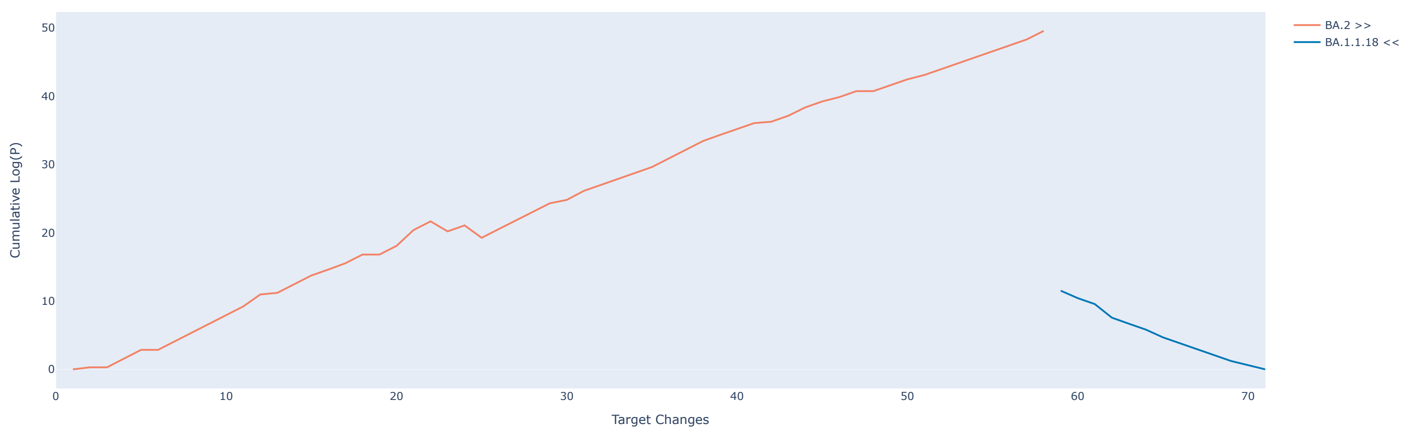


Cumulative Likelihood whole genome

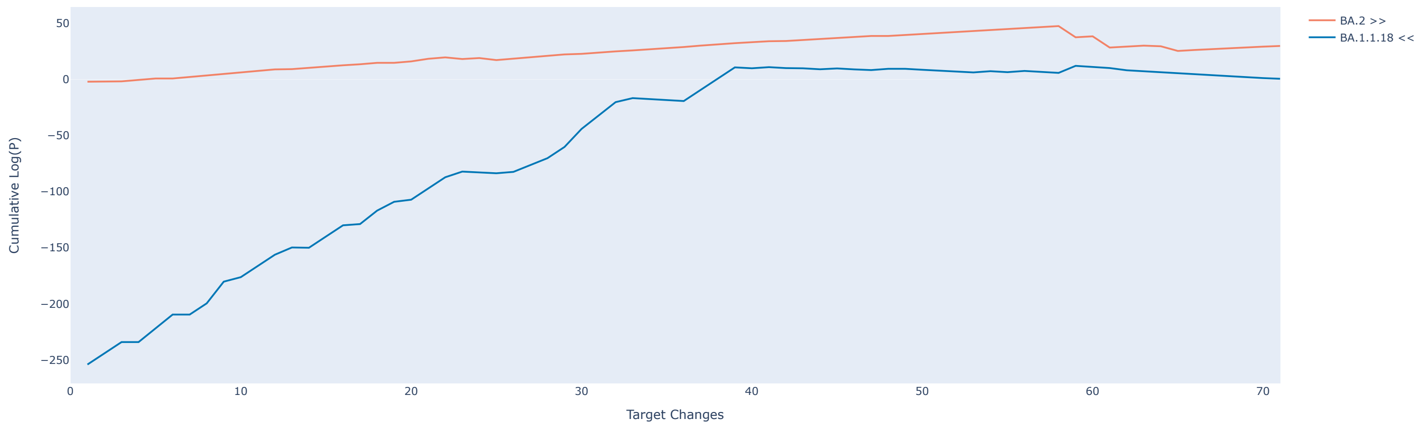


**Target sequence**

, 14_.|C, 44_C|T, 241_C|T, 670_T|G, 2790_C|T, 3037_C|T, 4184_G|A, 4321_C|T, 9344_C|T, 9424_A|G, 9534_C|T, 9866_C|T, 10029_C|T, 10198_C|T, 10447_G|A, 10449_C|A, 11288_11296, 12880_C|T, 14408_C|T, 15714_C|T, 17242_G|T, 17410_C|T, 17533_G|A, 18163_A|G, 18246_C|T, 19955_C|T, 20055_A|G, 21618_C|T, 21633_21641, 21987_G|A, 22200_T|G, 22578_G|A, 22674_C|T, 22679_T|C, 22686_C|T, 22688_A|G, 22775_G|A, 22786_A|C, 22813_G|T, 22882_T|G, 22992_G|A, 22995_C|A, 23013_A|C, 23040_A|G, 23055_A|G, 23063_A|T, 23075_T|C, 23403_A|G, 23525_C|T, 23599_T|G, 23604_C|A, 23854_C|A, 23948_G|T, 24424_A|T, 24469_T|A, 25000_C|T, 25584_C|T, 26060_C|T, 26235_G|T, 26270_C|T, 26530_A|G, 26577_C|G, 26709_G|A, 27259_A|C, 27807_C|T, 28271_A|T, 28311_C|T, 28362_28370, 28881_G|A, 28882_G|A, 28883_G|C

## Case 46 (1BP 3'): XP        test: KO

```
Target: (75%) 56 samples   Number of changes: 68
GT: BA.1.1* + BA.2*        GT BR: 58–67                GT BR coord: 27384 – 29510        Rank L1 L2: 1 11
BC: BA.1.1 + BA.5.6.3      BC BR: 59–60                BC BR coord: 27889 – 27890
Direction L1: >>           Initial region span: 1–56,60–68 Gap history (edge excluded): 56–60
Alt. candidates: [], [BA.5]
Model 1BP/2BP comparison:  –
Rec. model vs L1: 3.88e–12 Rec. model vs L2: 0.00e+00
Flags: Model_2BP_Bad_L1_opp
```

## BA.1.1 >>

| | num_seq | t_ch_MAX | max_CL | CL@BC_t_ch_MAX | aic | PV | PV_OK | t_ch_MAX_OK | phyl_OK |
|---|---|---|---|---|---|---|---|---|---|
| **BA.1.1** | 262925 | 56 | 65.322736 | NaN | NaN | NaN | * | * | * |
| **BA.1.1.13** | 3393 | 46 | 57.633917 | 43.530289 | 50.939421 | 1.020722e-06 | | | * |
| **BA.1.1.16** | 594 | 56 | 54.022748 | 37.301041 | 63.397917 | 2.010264e-09 | | * | * |
| **BA.1.1.18** | 22283 | 56 | 53.585571 | 45.585788 | 46.828425 | 7.968614e-06 | | * | * |
| **BA.1.1.7** | 168 | 56 | 52.612297 | 44.613052 | 48.773897 | 3.020766e-06 | | * | * |
| **BA.1.1.10** | 592 | 56 | 51.614835 | 43.615590 | 50.768820 | 1.111278e-06 | | * | * |
| **BA.1.1.14** | 5810 | 56 | 50.908746 | 42.908640 | 52.182720 | 5.490919e-07 | | * | * |
| **BA.1.1.6** | 22 | 56 | 50.828469 | 42.829223 | 52.341553 | 5.068757e-07 | | * | * |
| **BA.1.1.12** | 1943 | 56 | 50.221569 | 42.222323 | 53.555353 | 2.754114e-07 | | * | * |
| **BA.1.1.2** | 2970 | 56 | 50.110230 | 42.110985 | 53.778031 | 2.467229e-07 | | * | * |

## BA.5.6.3 <<

| | num_seq | t_ch_MAX | max_CL | CL@BC_t_ch_MAX | aic | PV | PV_OK | t_ch_MAX_OK | phyl_OK |
|---|---|---|---|---|---|---|---|---|---|
| **BA.5.6.3** | 46 | 60 | 11.488502 | NaN | NaN | NaN | * | * | * |
| **BA.5** | 3423 | 59 | 10.939393 | 10.069776 | 1.860448 | 0.241714 | * | * | * |
| **CH.1.1.11** | 170 | 59 | 10.919959 | 10.050342 | 1.899316 | 0.236928 | * | * | |
| **BG.2** | 5326 | 60 | 10.802126 | 5.051424 | 11.897152 | 0.001596 | * | * | |
| **CH.1.1.15** | 14 | 59 | 10.373279 | 9.503662 | 2.992676 | 0.137381 | * | * | |
| **BN.1.3.3** | 27 | 60 | 10.195436 | 3.257279 | 15.485442 | 0.000265 | * | * | |
| **BA.2.75.6** | 17 | 59 | 10.179123 | 9.309506 | 3.380988 | 0.113042 | * | * | |
| **BA.2.75.4** | 24 | 59 | 9.749163 | 8.879546 | 4.240908 | 0.073535 | * | * | |
| **BM.1.1** | 104 | 59 | 9.427337 | 8.557720 | 4.884560 | 0.053397 | * | * | |
| **BA.2.9.2** | 355 | 59 | 9.167892 | 8.298276 | 5.403449 | 0.041172 | * | * | |

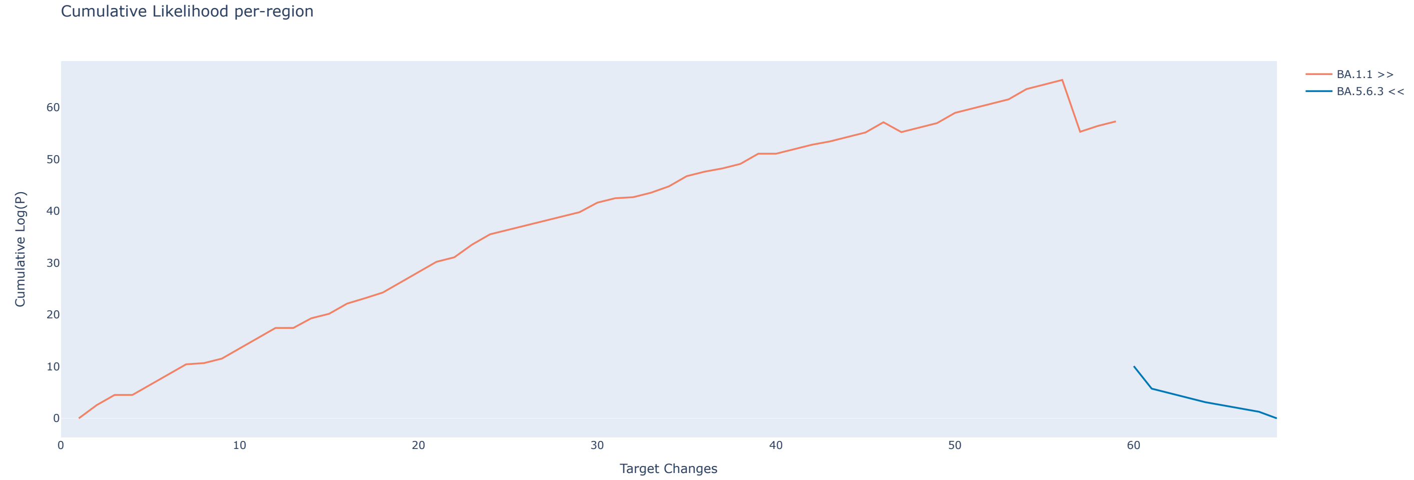


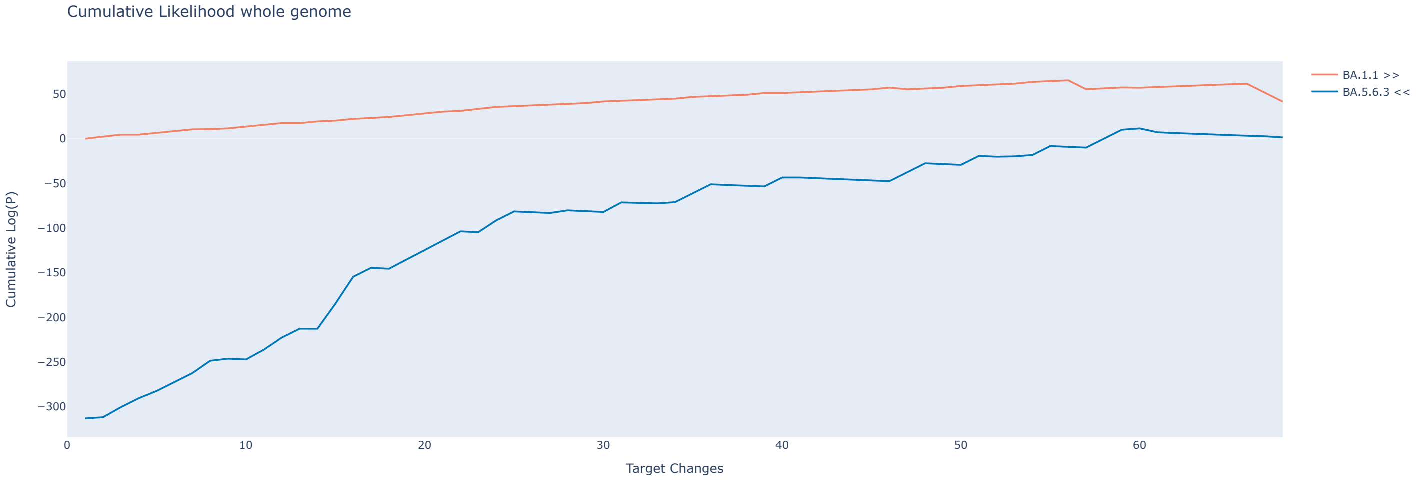


**Target sequence**

, 241_C|T, 2470_C|T, 2832_A|G, 3037_C|T, 5386_T|G, 6513_6515, 8393_G|A, 10029_C|T, 10449_C|A, 11285_11293, 11537_A|G, 13195_T|C, 14408_C|T, 15240_C|T, 18163_A|G, 21762_C|T, 21765_21770, 21846_C|T, 21987_21995, 22194_22196, 22204_.|GAGCCAGAA, 22578_G|A, 22599_G|A, 22673_T|C, 22674_C|T, 22679_T|C, 22686_C|T, 22813_G|T, 22882_T|G, 22898_G|A, 22992_G|A, 22995_C|A, 23013_A|C, 23040_A|G, 23048_G|A, 23055_A|G, 23063_A|T, 23075_T|C, 23202_C|A, 23403_A|G, 23525_C|T, 23599_T|G, 23604_C|A, 23854_C|A, 23948_G|T, 24130_C|A, 24190_A|C, 24424_A|T, 24469_T|A, 24503_C|T, 25000_C|T, 25584_C|T, 26270_C|T, 26530_A|G, 26577_C|G, 26709_G|A, 26880_C|A, 27259_A|C, 27807_C|T, 28254_28254, 28271_A|T, 28311_C|T, 28362_28370, 28881_G|A, 28882_G|A, 28883_G|C, 29510_A|C, 29734_29759

## Case 47 (2BP): XAC          test: OK

Target: (75%) 34 samples
Number of changes: 71
GT: BA.2* + BA.1* + BA.2*
GT BR: 56-58, 62-70
GT BR coord: 25812 - 26060, 27384 - 29510
Rank L1 L2: 3 11 11
BC: BA.2.3 + BA.1.1.9 + BA.2.3
BC BR: 57-58, 62-63
BC BR coord: 26030 - 26031, 27806 - 27807
Direction L1: >>
Initial region span: 1-56,58-62,63-71
Gap history (edge excluded): 56-63 -> 56-58
Alt. candidates: [], []
Model 1BP/2BP comparison: 2BP vs 1BP: 2.21e-09
Rec. model vs L1: 5.84e-46
Rec. model vs L2: 0.00e+00
Flags: Model_2BP_Best

## BA.2.3 >>

| | num_seq | t_ch_MAX | max_CL | CL@BC_t_ch_MAX | aic | PV | PV_OK | t_ch_MAX_OK | phyl_OK |
|---|---|---|---|---|---|---|---|---|---|
| **BA.2.3** | 22517 | 56 | 53.275674 | NaN | NaN | NaN | * | * | * |
| **BA.2.3.2** | 339 | 54 | 52.368959 | 45.097806 | 51.804388 | 2.323549e-07 | | | * |
| **BA.2** | 359165 | 54 | 46.882998 | 53.346048 | 35.307904 | 8.849318e-04 | * | | * |
| **BA.2.3.1** | 111 | 46 | 46.846054 | 36.084811 | 69.830378 | 2.824796e-11 | | | * |
| **BA.2.3.9** | 127 | 54 | 43.760731 | 36.301630 | 69.396739 | 3.502356e-11 | | | * |
| **BA.2.3.10** | 121 | 54 | 43.662876 | 28.308346 | 85.383307 | 1.186718e-14 | | | * |
| **BA.2.3.16** | 36 | 54 | 43.374085 | 36.054034 | 69.891931 | 2.741310e-11 | | | * |
| **BA.2.9** | 61803 | 54 | 40.613593 | 39.200486 | 63.599028 | 6.365234e-10 | | | |
| **BA.2.65** | 815 | 54 | 37.816683 | 15.766004 | 110.467992 | 4.227885e-20 | | | |
| **BA.2.3.5** | 139 | 54 | 37.676374 | 30.556232 | 80.887535 | 1.120311e-13 | | | * |

## BA.1.1.9 <<

| | num_seq | t_ch_MAX | max_CL | CL@BC_t_ch_MAX | aic | PV | PV_OK | t_ch_MAX_OK | phyl_OK |
|---|---|---|---|---|---|---|---|---|---|
| **BA.1.1.9** | 172 | 58 | 5.763703 | NaN | NaN | NaN | * | * | * |
| **BC.1** | 209 | 58 | 5.763703 | 5.763703 | 0.472593 | 1.000000 | * | * | |
| **BA.1.5** | 274 | 58 | 5.763703 | 5.763703 | 0.472593 | 1.000000 | * | * | |
| **BA.1.23** | 12 | 58 | 5.763703 | 5.763703 | 0.472593 | 1.000000 | * | * | |
| **BA.1.16.1** | 68 | 58 | 5.763703 | 5.763703 | 0.472593 | 1.000000 | * | * | |
| **BA.1.1.6** | 22 | 58 | 5.763703 | 5.763703 | 0.472593 | 1.000000 | * | * | |
| **BA.1.1.12** | 1943 | 58 | 5.760612 | 5.760612 | 0.478776 | 0.995012 | * | * | |
| **BA.1.1.17** | 191 | 58 | 5.758454 | 5.758454 | 0.483092 | 0.995012 | * | * | |
| **BA.1.1.13** | 3393 | 58 | 5.758392 | -0.431014 | 12.862027 | 0.002040 | * | * | |
| **BA.1.1.4** | 767 | 58 | 5.757163 | 5.757163 | 0.485674 | 0.990050 | * | * | |

## BA.2.3 <<

| | num_seq | t_ch_MAX | max_CL | CL@BC_t_ch_MAX | aic | PV | PV_OK | t_ch_MAX_OK | phyl_OK |
|---|---|---|---|---|---|---|---|---|---|
| **BA.2.3** | 22517 | 63 | 7.990148 | None | None | None | * | * | * |

Cumulative Likelihood per-region

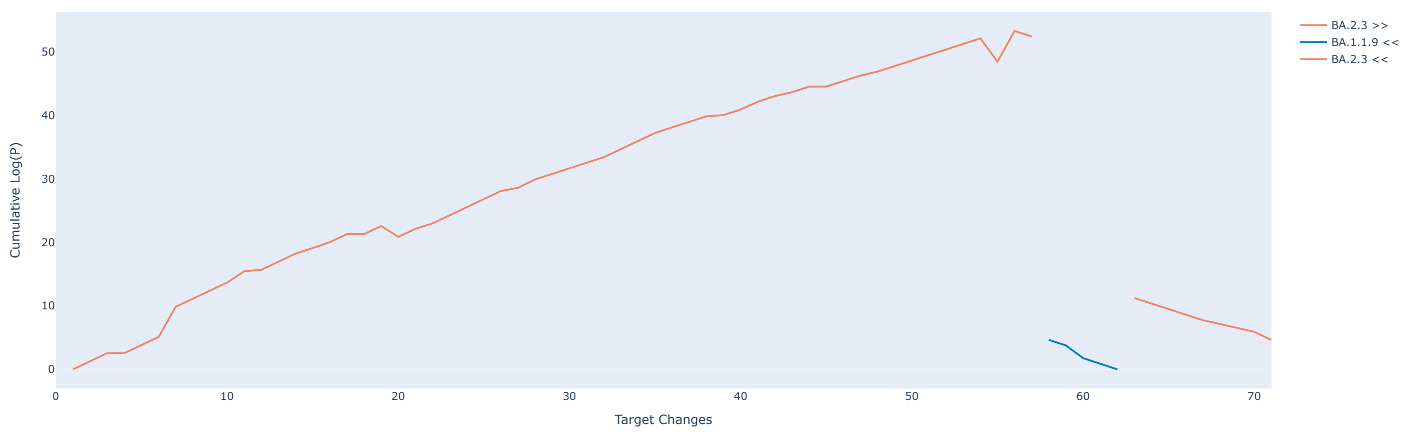


Cumulative Likelihood whole genome

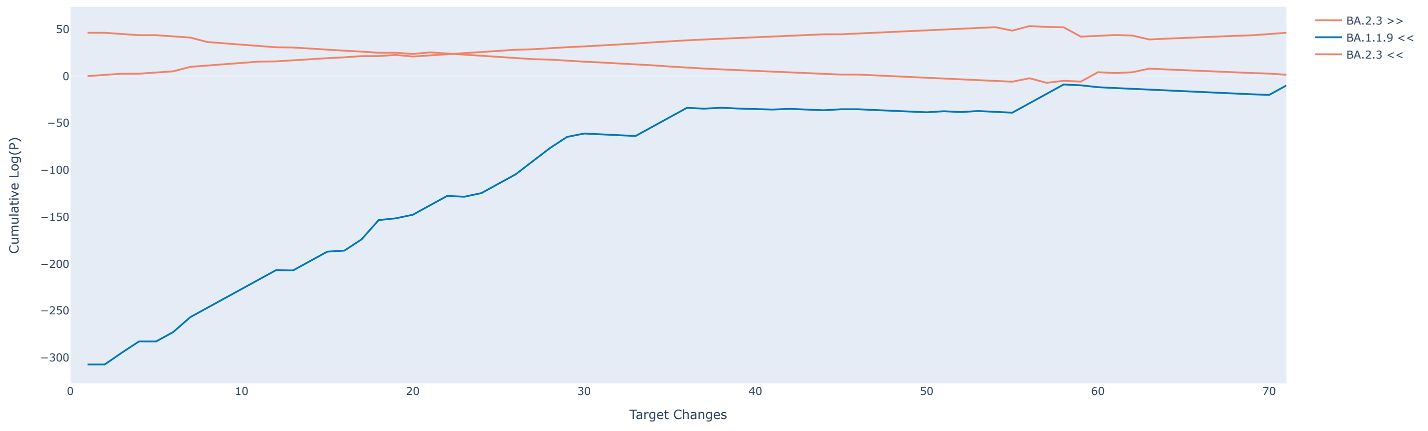


**Target sequence**

, 241_C|T, 670_T|G, 2790_C|T, 3037_C|T, 4184_G|A, 4321_C|T, 8991_C|T, 9344_C|T, 9424_A|G, 9534_C|T, 9866_C|T, 10029_C|T, 10198_C|T, 10447_G|A, 10449_C|A, 11288_11296, 12880_C|T, 14408_C|T, 15714_C|T, 15720_C|T, 17410_C|T, 18163_A|G, 19955_C|T, 20055_A|G, 21618_C|T, 21633_21641, 21987_G|A, 22200_T|G, 22578_G|A, 22674_C|T, 22679_T|C, 22686_C|T, 22688_A|G, 22775_G|A, 22786_A|C, 22813_G|T, 22882_T|G, 22992_G|A, 22995_C|A, 23013_A|C, 23040_A|G, 23055_A|G, 23063_A|T, 23075_T|C, 23403_A|G, 23525_C|T, 23599_T|G, 23604_C|A, 23854_C|A, 23948_G|T, 24424_A|T, 24469_T|A, 25000_C|T, 25584_C|T, 25731_C|T, 25810_C|T, 26028_C|T, 26270_C|T, 26530_A|G, 26577_C|G, 26709_G|A, 27259_A|C, 27807_C|T, 28271_A|T, 28311_C|T, 28362_28370, 28881_G|A, 28882_G|A, 28883_G|C, 29510_A|C, 29734_29759

## Case 48 (2BP): XAK    test: KO

Target: (75%) 5 samples
Number of changes: 73
GT: BA.2* + BA.1* + BA.2*
GT BR: 20–23, 23–24
GT BR coord: 13194 – 15241, 21617 – 21763
Rank L1 L2: 1 11 –
BC: BA.2 + BA.2.10
BC BR: 26–27
BC BR coord: 22001 – 22002
Direction L1: <<
Initial region span: 1–18,39–73
Gap history (edge excluded): 18–39
Alt. candidates: [], [BA.2]
Model 1BP/2BP comparison:
1BP vs 2BP: 2.38e–10
Rec. model vs L1: 1.96e+03
Rec. model vs L2: 6.79e–12
Flags: Model_1BP_L1eqL2, Model_1BP_Best

## BA.2 >>

| | num_seq | t_ch_MAX | max_CL | CL@BC_t_ch_MAX | aic | PV | PV_OK | t_ch_MAX_OK | phyl_OK |
|---|---|---|---|---|---|---|---|---|---|
| **BA.2** | 359165 | 18 | 8.791758 | NaN | NaN | NaN | * | * | * |
| **BA.2.36** | 1320 | 18 | 7.729049 | -39.558170 | 191.116341 | 3.083889e-14 | | * | * |
| **BF.20** | 26 | 7 | 7.574204 | -65.465163 | 242.930326 | 1.732607e-25 | | | |
| **BA.2.31.1** | 54 | 7 | 7.272938 | -70.966287 | 253.932574 | 7.080770e-28 | | | * |
| **BA.2.72** | 268 | 7 | 6.360344 | -48.458882 | 208.917764 | 4.206082e-18 | | | * |
| **BA.5.2.14** | 108 | 7 | 5.936329 | -83.533876 | 279.067752 | 2.460361e-33 | | | |
| **BA.5.1.10** | 2819 | 7 | 5.386913 | -36.802774 | 185.605548 | 4.848194e-13 | | | |
| **XBB.1.5.4** | 250 | 7 | 5.381937 | -86.586952 | 285.173904 | 1.165200e-34 | | | |
| **BA.2.20** | 357 | 7 | 5.378569 | -41.872366 | 195.744731 | 3.061106e-15 | | | * |
| **BA.2.10.1** | 1150 | 7 | 5.310407 | -44.759845 | 201.519690 | 1.701247e-16 | | | * |

## BA.2.10 <<

| | num_seq | t_ch_MAX | max_CL | CL@BC_t_ch_MAX | aic | PV | PV_OK | t_ch_MAX_OK | phyl_OK |
|---|---|---|---|---|---|---|---|---|---|
| **BA.2.10** | 12936 | 39 | 26.936405 | NaN | NaN | NaN | * | * | * |
| **BA.2** | 359165 | 39 | 26.286362 | 21.433721 | 111.132557 | 4.274149e-01 | * | * | * |
| **BA.2.75.5** | 215 | 30 | 23.447112 | -16.639178 | 187.278356 | 1.244765e-17 | | | |
| **BA.2.9** | 61803 | 39 | 20.664009 | 13.239560 | 127.520880 | 1.179795e-04 | * | * | |
| **BA.2.12.1** | 94944 | 39 | 19.909427 | 10.756585 | 132.486830 | 9.830704e-06 | | * | |
| **BN.1.7** | 232 | 30 | 18.453253 | -35.609014 | 225.218029 | 7.186559e-26 | | | |
| **BA.2.1** | 7140 | 39 | 17.973804 | 8.395319 | 137.209361 | 9.282173e-07 | | * | * |
| **BN.1** | 942 | 30 | 17.824779 | -25.446833 | 204.893665 | 1.866914e-21 | | | |
| **BA.2.76** | 356 | 39 | 16.204563 | -5.488905 | 164.977811 | 8.659060e-13 | | * | |
| **XBB.1.5** | 35885 | 42 | 15.196244 | -37.448706 | 228.897412 | 1.141351e-26 | | | |

Cumulative Likelihood per-region

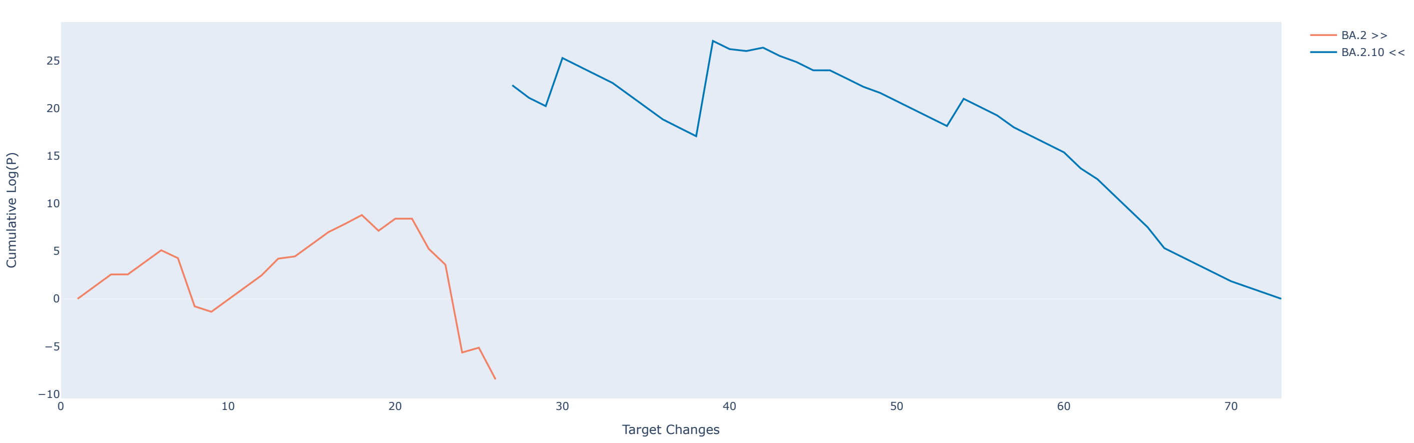


Cumulative Likelihood whole genome

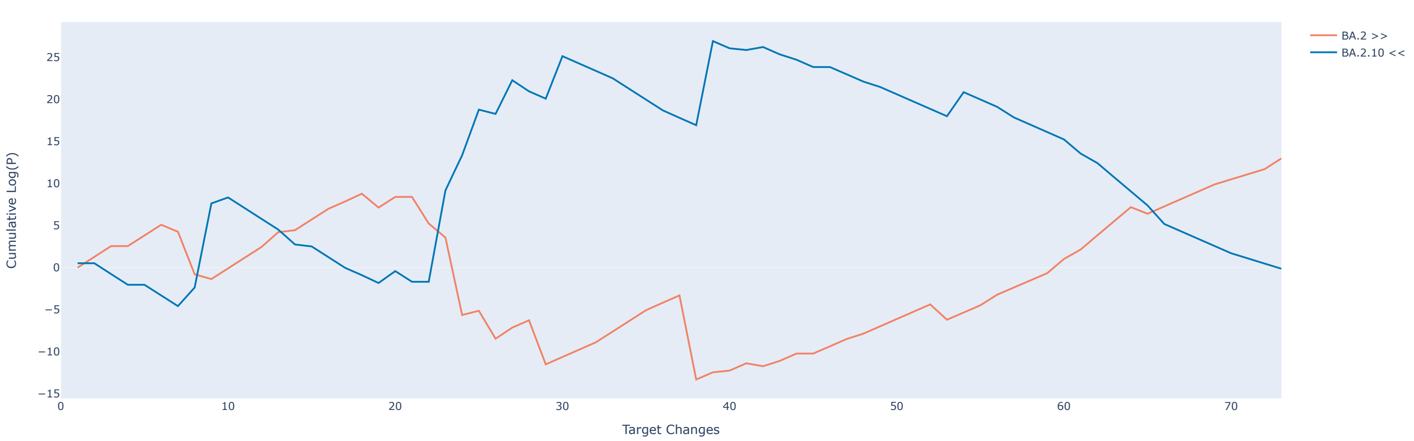


**Target sequence**

, 241_C|T, 670_T|G, 2790_C|T, 3037_C|T, 4184_G|A, 4321_C|T, 4927_C|T, 5386_T|G, 7834_C|T, 9344_C|T, 9424_A|G, 9534_C|T, 9866_C|T, 10029_C|T, 10198_C|T, 10447_G|A, 10449_C|A, 11288_11296, 12049_C|T, 12880_C|T, 14408_C|T, 15240_C|T, 18163_A|G, 21765_21770, 21987_G|A, 22001_A|G, 22200_T|G, 22578_G|A, 22599_G|A, 22674_C|T, 22679_T|C, 22686_C|T, 22688_A|G, 22775_G|A, 22786_A|C, 22813_G|T, 22882_T|G, 22942_T|G, 22992_G|A, 22995_C|A, 23013_A|C, 23055_A|G, 23063_A|T, 23075_T|C, 23403_A|G, 23525_C|T, 23599_T|G, 23604_C|A, 23854_C|A, 23948_G|T, 24424_A|T, 24469_T|A, 24863_C|T, 25000_C|T, 25584_C|T, 26060_C|T, 26270_C|T, 26577_C|G, 26709_G|A, 26858_C|T, 27259_A|C, 27382_G|C, 27383_A|T, 27384_T|C, 27507_A|G, 27807_C|T, 28271_A|T, 28311_C|T, 28362_28370, 28881_G|A, 28882_G|A, 28883_G|C, 29510_A|C

## Case 49 (2BP): XAZ    test: KO

Target: (75%) 289 samples
GT: BA.2.5 + BA.5 + BA.2.5
BC: BA.5.1.27 + BA.5
Direction L1: <<
Alt. candidates: [], [BA.5.3, BA.5.3.1, BA.5.5, BA.5.11, BA.5.3.2, BA.5.2.1, B.1.1.529]
Model 1BP/2BP comparison:
Rec. model vs L1: 8.71e-21
Flags: Model_1BP_L1eqL2, Model_2BP_Bad_L1_opp

Number of changes: 71
GT BR: 8-14, 63-64    GT BR coord: 3358 - 9866, 27384 - 27387    Rank L1 L2: 11 1 -
BC BR: 7-8    BC BR coord: 3323 - 3324
Initial region span: 1-7,9-71    Gap history (edge excluded): 7-9
-
Rec. model vs L2: 2.37e-13

## BA.5.1.27 >>

| | num_seq | t_ch_MAX | max_CL | CL@BC_t_ch_MAX | aic | PV | PV_OK | t_ch_MAX_OK | phyl_OK |
|---|---|---|---|---|---|---|---|---|---|
| **BA.5.1.27** | 1123 | 7 | 6.130829 | NaN | NaN | NaN | * | * | * |
| **XBB.1.5.34** | 211 | 7 | 4.696558 | 4.696558 | 10.606884 | 2.381154e-01 | * | * | |
| **BR.1** | 117 | 3 | 4.370824 | -14.350224 | 48.700449 | 1.275408e-09 | | | |
| **BA.5.1.30** | 3831 | 6 | 3.861987 | -6.138013 | 32.276026 | 4.690366e-06 | | * | |
| **BA.2.67** | 174 | 3 | 3.658309 | -15.062739 | 50.125479 | 6.239194e-10 | | | |
| **BA.5.6.3** | 46 | 3 | 3.608192 | -15.112856 | 50.225712 | 5.934904e-10 | | | |
| **BA.5.1.22** | 3125 | 6 | 3.548784 | -6.451216 | 32.902432 | 3.440134e-06 | | * | |
| **BQ.1.1.28** | 348 | 3 | 3.269144 | -22.847512 | 65.695024 | 2.595051e-13 | | | |
| **CH.1.1.3** | 35 | 3 | 3.188338 | -15.532710 | 51.065420 | 3.899510e-10 | | | |
| **BA.2.65** | 815 | 7 | 3.178485 | 3.178485 | 13.643030 | 5.233971e-02 | * | * | |

## BA.5 <<

| | num_seq | t_ch_MAX | max_CL | CL@BC_t_ch_MAX | aic | PV | PV_OK | t_ch_MAX_OK | phyl_OK |
|---|---|---|---|---|---|---|---|---|---|
| **BA.5** | 3423 | 9 | 61.132904 | NaN | NaN | NaN | * | * | * |
| **BA.5.3** | 440 | 9 | 60.075724 | 50.075724 | 57.848553 | 3.464558e-01 | * | * | * |
| **BA.5.3.1** | 370 | 9 | 55.139889 | 45.139889 | 67.720222 | 2.491177e-03 | * | * | * |
| **BA.5.5** | 22209 | 9 | 53.808275 | 43.808275 | 70.383451 | 6.588597e-04 | * | * | * |
| **BA.5.11** | 250 | 9 | 53.587849 | 43.587849 | 70.824302 | 5.287473e-04 | * | * | * |
| **BA.5.3.2** | 212 | 9 | 53.254709 | 43.254709 | 71.490583 | 3.782331e-04 | * | * | * |
| **BE.1** | 5712 | 5 | 52.284871 | 51.804555 | 54.390889 | 1.954237e+00 | * | | * |
| **BA.5.2.1** | 58578 | 9 | 52.034436 | 50.029637 | 57.940725 | 3.312109e-01 | * | * | * |
| **B.1.1.529** | 248 | 9 | 52.006781 | 42.006781 | 73.986437 | 1.083656e-04 | * | * | * |
| **BF.17** | 10 | 11 | 50.876263 | 33.177991 | 91.644019 | 1.593098e-08 | | | * |

Cumulative Likelihood per-region

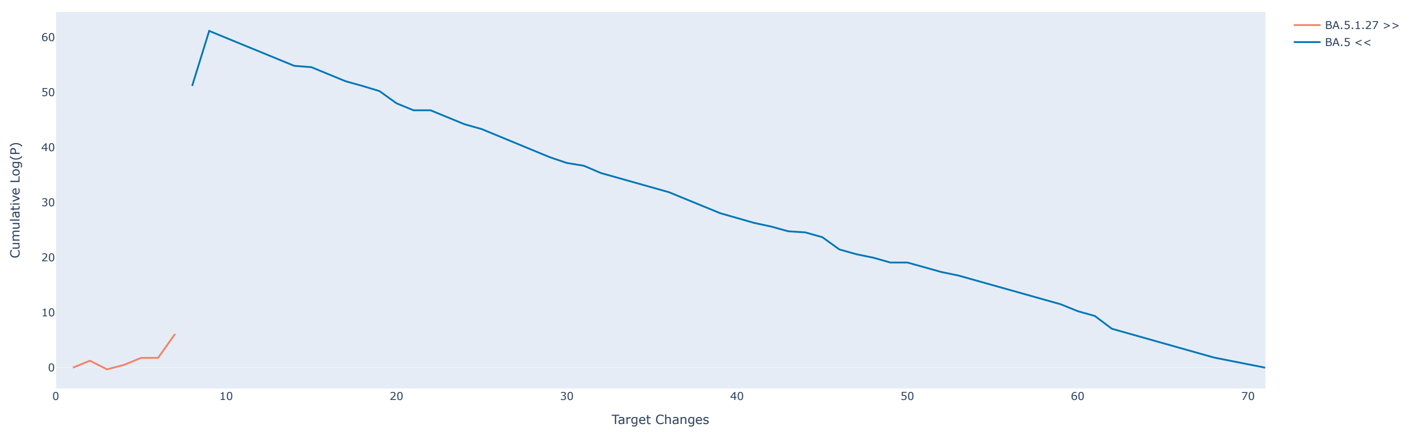


Cumulative Likelihood whole genome

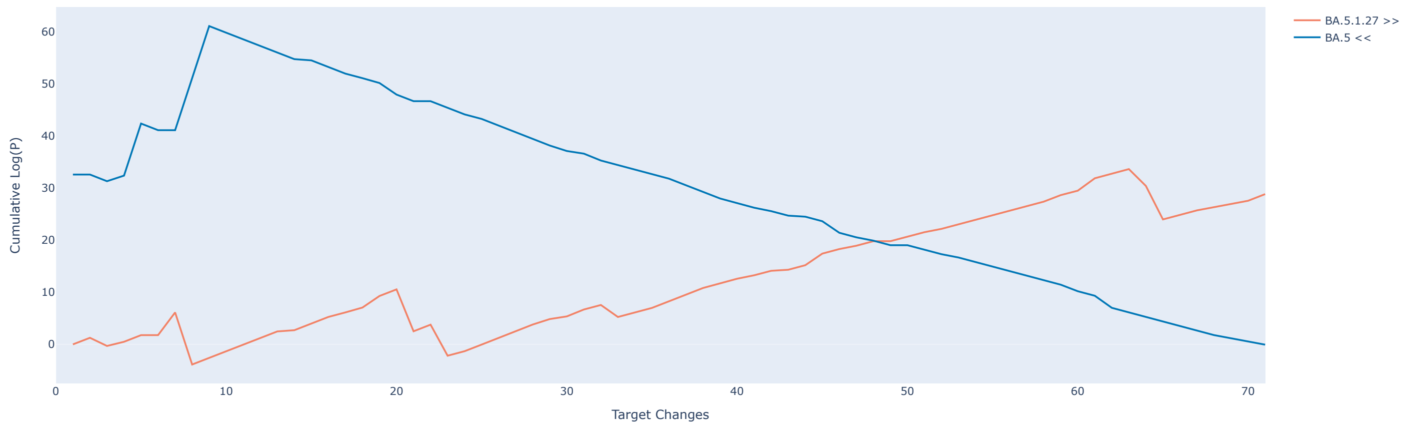


**Target sequence**

, 241_C|T, 670_T|G, 1912_C|T, 2232_C|T, 2790_C|T, 3037_C|T, 3317_C|T, 3358_T|C, 4184_G|A, 4321_C|T, 9344_C|T, 9424_A|G, 9534_C|T, 10029_C|T, 10198_C|T, 10447_G|A, 10449_C|A, 11288_11296, 12160_G|A, 12880_C|T, 14408_C|T, 15714_C|T, 17410_C|T, 18163_A|G, 19955_C|T, 20055_A|G, 21618_C|T, 21633_21641, 21765_21770, 21987_G|A, 22200_T|G, 22578_G|A, 22674_C|T, 22679_T|C, 22686_C|T, 22688_A|G, 22775_G|A, 22786_A|C, 22813_G|T, 22882_T|G, 22917_T|G, 22992_G|A, 22995_C|A, 23013_A|C, 23018_T|G, 23055_A|G, 23063_A|T, 23075_T|C, 23403_A|G, 23525_C|T, 23599_T|G, 23604_C|A, 23854_C|A, 23948_G|T, 24424_A|T, 24469_T|A, 25000_C|T, 25584_C|T, 26060_C|T, 26270_C|T, 26529_G|A, 26577_C|G, 26709_G|A, 27807_C|T, 28271_A|T, 28311_C|T, 28362_28370, 28881_G|A, 28882_G|A, 28883_G|C, 29510_A|C

## Case 50 (2BP): XBL      test: OK

Target: (75%) 5 samples      Number of changes: 101
GT: XBB.1 + BA.2.75 + XBB.1      GT BR: 2–7, 12–22      GT BR coord: 405 – 3796, 5183 – 12444      Rank L1 L2: 11 11 4
BC: XBB.1.5.24 + BN.1.3 + XBB.1.5.24      BC BR: 3–4, 12–13      BC BR coord: 672 – 673, 9343 – 9344
Direction L1: <<      Initial region span: 1–3,4–12,13–101      Gap history (edge excluded): 3–13 ->
Alt. candidates: [], [XBB.1.5]
Model 1BP/2BP comparison:      2BP vs 1BP: 2.54e–06
Rec. model vs L1: 1.54e–44      Rec. model vs L2: 3.99e–251
Flags: Model_2BP_Best

## XBB.1.5.24 >>

| | num_seq | t_ch_MAX | max_CL | CL@BC_t_ch_MAX | aic | PV | PV_OK | t_ch_MAX_OK | phyl_OK |
|---|---|---|---|---|---|---|---|---|---|
| **XBB.1.5.24** | 77 | 3 | 5.609355 | None | None | None | * | * | * |

## BN.1.3 <<

| | num_seq | t_ch_MAX | max_CL | CL@BC_t_ch_MAX | aic | PV | PV_OK | t_ch_MAX_OK | phyl_OK |
|---|---|---|---|---|---|---|---|---|---|
| **BN.1.3** | 919 | 4 | 30.148346 | NaN | NaN | NaN | * | * | * |
| **BA.2.75.6** | 17 | 7 | 23.994176 | 15.273128 | -6.546256 | 3.466327e-07 | | | |
| **BN.1.4.3** | 52 | 7 | 23.994176 | -3.590363 | 31.180726 | 2.222819e-15 | | | |
| **BA.2.75.4** | 24 | 7 | 23.994176 | 15.273128 | -6.546256 | 3.466327e-07 | | | |
| **BA.2.75.3** | 47 | 7 | 23.994176 | 15.273128 | -6.546256 | 3.466327e-07 | | | |
| **BA.2.75.10** | 10 | 7 | 23.994176 | 15.273128 | -6.546256 | 3.466327e-07 | | | |
| **BN.1.3.2** | 15 | 7 | 23.994176 | 15.273128 | -6.546256 | 3.466327e-07 | | | * |
| **BN.1.2.1** | 135 | 7 | 23.994176 | 7.253036 | 9.493928 | 1.139798e-10 | | | |
| **BN.1.2** | 405 | 7 | 23.994176 | 15.273128 | -6.546256 | 3.466327e-07 | | | |
| **BN.1.10** | 43 | 7 | 23.994176 | 15.273128 | -6.546256 | 3.466327e-07 | | | |

## XBB.1.5.24 <<

| | num_seq | t_ch_MAX | max_CL | CL@BC_t_ch_MAX | aic | PV | PV_OK | t_ch_MAX_OK | phyl_OK |
|---|---|---|---|---|---|---|---|---|---|
| **XBB.1.5.24** | 77 | 13 | 162.345223 | NaN | NaN | NaN | * | * | * |
| **XBB.1.5** | 35885 | 13 | 151.129402 | 156.726577 | -117.453154 | 1.327006e-05 | * | * | * |
| **XBB.1.5.33** | 550 | 13 | 141.091800 | 146.693855 | -97.387710 | 5.846545e-10 | | * | |
| **XBB.1** | 1674 | 13 | 137.819386 | 143.409556 | -90.819112 | 2.188981e-11 | | * | * |
| **XBB.1.5.34** | 211 | 16 | 137.044963 | 138.089488 | -80.178976 | 1.071014e-13 | | | |
| **XBB.1.18.1** | 15 | 13 | 136.969465 | 142.578820 | -89.157639 | 9.545035e-12 | | * | |
| **XBB.1.5.21** | 311 | 13 | 133.552353 | 139.155256 | -82.310512 | 3.106840e-13 | | * | |
| **XBB.1.5.17** | 668 | 13 | 133.233208 | 138.835059 | -81.670117 | 2.256029e-13 | | * | |
| **XBB.1.5.35** | 273 | 13 | 133.188864 | 138.794549 | -81.589098 | 2.167569e-13 | | * | |
| **XBB.1.5.26** | 47 | 13 | 132.562180 | 138.171534 | -80.343069 | 1.160216e-13 | | * | |

Cumulative Likelihood per-region

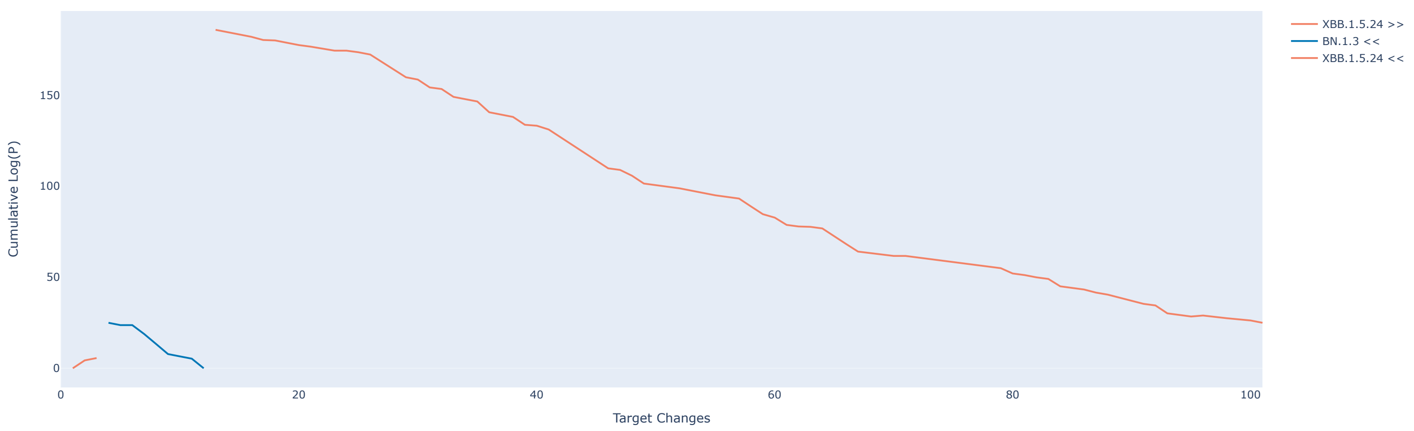


Cumulative Likelihood whole genome

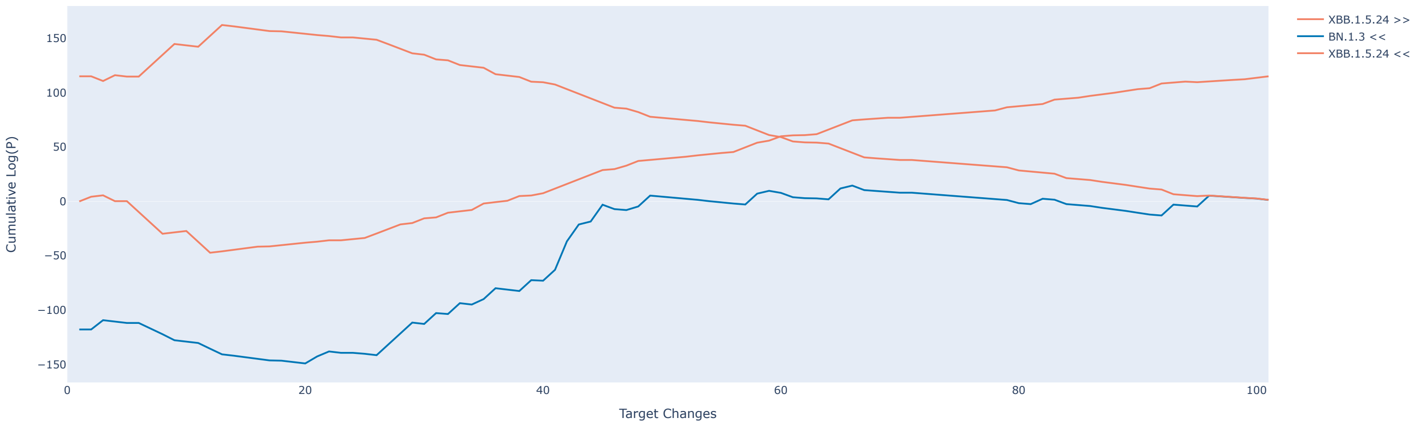


**Target sequence**

, 241_C|T, 405_A|G, 670_T|G, 2790_C|T, 3037_C|T, 3446_G|A, 3796_C|T, 3927_C|T, 4184_G|A, 4321_C|T, 4586_C|T, 5183_C|T, 9344_C|T, 9424_A|G, 9534_C|T, 9866_C|T, 10029_C|T, 10198_C|T, 10447_G|A, 10449_C|A, 11288_11296, 12880_C|T, 14408_C|T, 15451_G|A, 15714_C|T, 15738_C|T, 15939_T|C, 16342_T|C, 17410_C|T, 17859_T|C, 18163_A|G, 19326_A|G, 19955_C|T, 20055_A|G, 20379_A|G, 21618_C|T, 21633_21641, 21810_T|C, 21987_G|A, 21992_21994, 22000_C|A, 22109_C|G, 22200_T|A, 22317_G|T, 22577_G|C, 22578_G|A, 22599_G|C, 22664_C|A, 22674_C|T, 22679_T|C, 22686_C|T, 22688_A|G, 22775_G|A, 22786_A|C, 22813_G|T, 22882_T|G, 22895_G|C, 22896_T|C, 22898_G|A, 22942_T|G, 22992_G|A, 22995_C|A, 23013_A|C, 23018_T|C, 23019_T|C, 23031_T|C, 23055_A|G, 23063_A|T, 23075_T|C, 23403_A|G, 23525_C|T, 23599_T|G, 23604_C|A, 23854_C|A, 23948_G|T, 24424_A|T, 24469_T|A, 25000_C|T, 25416_C|T, 25584_C|T, 26060_C|T, 26270_C|T, 26275_A|G, 26577_C|G, 26709_G|A, 26858_C|T, 27259_A|C, 27382_G|C, 27383_A|T, 27384_T|C, 27807_C|T, 27915_G|T, 28271_A|T, 28311_C|T, 28312_C|T, 28362_28370, 28881_G|A, 28882_G|A, 28883_G|C, 29510_A|C, 29734_29759

**Case 51 (2BP): XBT** test: OK

Target: (75%) 8 samples
GT: BA.5.2.34 + BA.2.75 + BA.5.2.34
BC: BA.5.2.34 + BL.1.5 + BA.5.2.34
Direction L1: <<
Alt. candidates: [BA.2.75.1, BL.1, BA.2.75], []
Model 1BP/2BP comparison:
Rec. model vs L1: 1.21e-110
Flags: Model_1BP_L1eqL2, Model_2BP_Best

Number of changes: 86
GT BR: 10-14, 38-50
BC BR: 13-14, 39-40
Initial region span: 1-13,14-39,40-86
2BP vs 1BP: 2.64e-57
Rec. model vs L2: 4.69e-215

GT BR coord: 5182 - 9767, 22576 - 22899 Rank L1 L2: 1 9 1
BC BR coord: 9610 - 9611, 22577 - 22578
Gap history (edge excluded): 13-40 ->

## BA.5.2.34 >>

| | num_seq | t_ch_MAX | max_CL | CL@BC_t_ch_MAX | aic | PV | PV_OK | t_ch_MAX_OK | phyl_OK |
|---|---|---|---|---|---|---|---|---|---|
| **BA.5.2.34** | 1147 | 13 | 22.752499 | None | None | None | * | * | * |

## BL.1.5 <<

| | num_seq | t_ch_MAX | max_CL | CL@BC_t_ch_MAX | aic | PV | PV_OK | t_ch_MAX_OK | phyl_OK |
|---|---|---|---|---|---|---|---|---|---|
| **BL.1.5** | 13 | 14 | 45.657575 | NaN | NaN | NaN | * | * | * |
| **BL.2** | 59 | 14 | 45.657575 | 45.657575 | -39.315150 | 1.000000 | * | * | |
| **BA.2.75.6** | 17 | 14 | 45.657575 | 45.657575 | -39.315150 | 1.000000 | * | * | |
| **BN.5** | 33 | 14 | 45.657575 | 45.657575 | -39.315150 | 1.000000 | * | * | |
| **BY.1** | 153 | 14 | 45.644460 | 45.644460 | -39.288921 | 0.985112 | * | * | |
| **BA.2.75.1** | 158 | 14 | 45.638527 | 45.638527 | -39.277055 | 0.980199 | * | * | * |
| **CA.1** | 134 | 14 | 45.635103 | 45.635103 | -39.270206 | 0.975310 | * | * | |
| **BL.1** | 158 | 14 | 45.625583 | 45.625583 | -39.251167 | 0.965605 | * | * | * |
| **BA.2.75** | 122 | 14 | 45.599687 | 45.599687 | -39.199375 | 0.941765 | * | * | * |
| **BA.2.75.2** | 582 | 14 | 45.598694 | 45.598694 | -39.197387 | 0.941765 | * | * | |

## BA.5.2.34 <<

| | num_seq | t_ch_MAX | max_CL | CL@BC_t_ch_MAX | aic | PV | PV_OK | t_ch_MAX_OK | phyl_OK |
|---|---|---|---|---|---|---|---|---|---|
| **BA.5.2.34** | 1147 | 40 | 62.945666 | NaN | NaN | NaN | * | * | * |
| **BA.5.2.6** | 2139 | 40 | 56.918184 | 61.578673 | -1.157345 | 3.348244e-11 | | * | |
| **BA.5.2.21** | 2744 | 42 | 51.049595 | 34.221703 | 53.556593 | 4.390562e-23 | | | |
| **BA.5.2** | 32711 | 42 | 49.013822 | 44.672928 | 32.654145 | 1.524294e-18 | | | * |
| **CN.2** | 183 | 40 | 45.480300 | 27.535135 | 66.929729 | 5.486071e-26 | | * | |
| **BA.5.2.13** | 710 | 40 | 45.440575 | 27.496500 | 67.007000 | 5.270960e-26 | | * | |
| **BA.5.2.28** | 617 | 40 | 42.471216 | 24.524021 | 72.951959 | 2.704177e-27 | | * | |
| **BA.5.2.39** | 70 | 40 | 41.952150 | 23.960179 | 74.079642 | 1.536946e-27 | | * | |
| **CF.1** | 42 | 42 | 41.484112 | 14.420032 | 93.159937 | 1.105323e-31 | | | |
| **BA.5.2.31** | 603 | 42 | 41.413764 | 14.334737 | 93.330526 | 1.015253e-31 | | | |

Cumulative Likelihood per-region

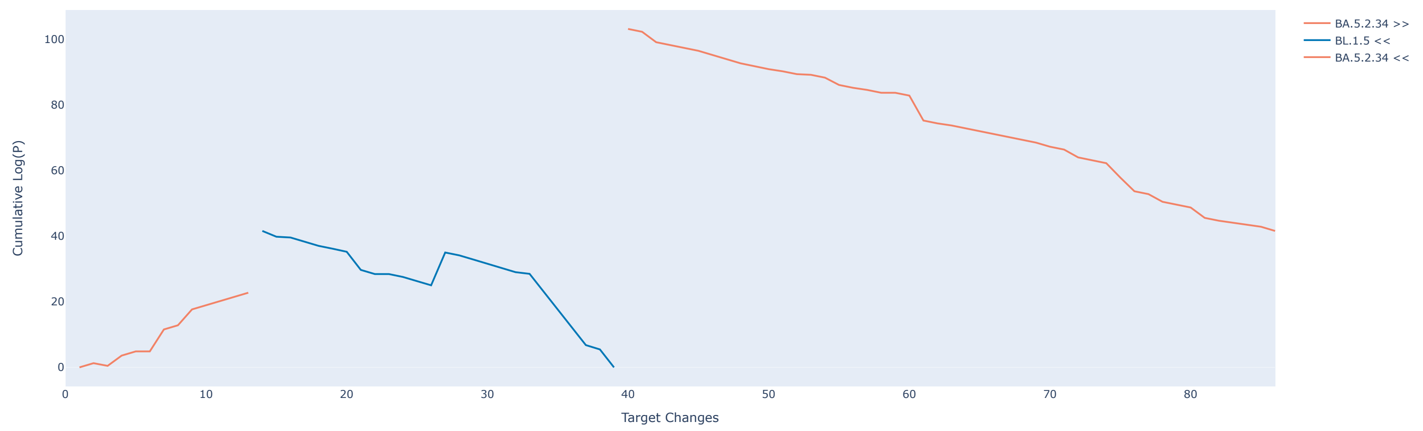


Cumulative Likelihood whole genome

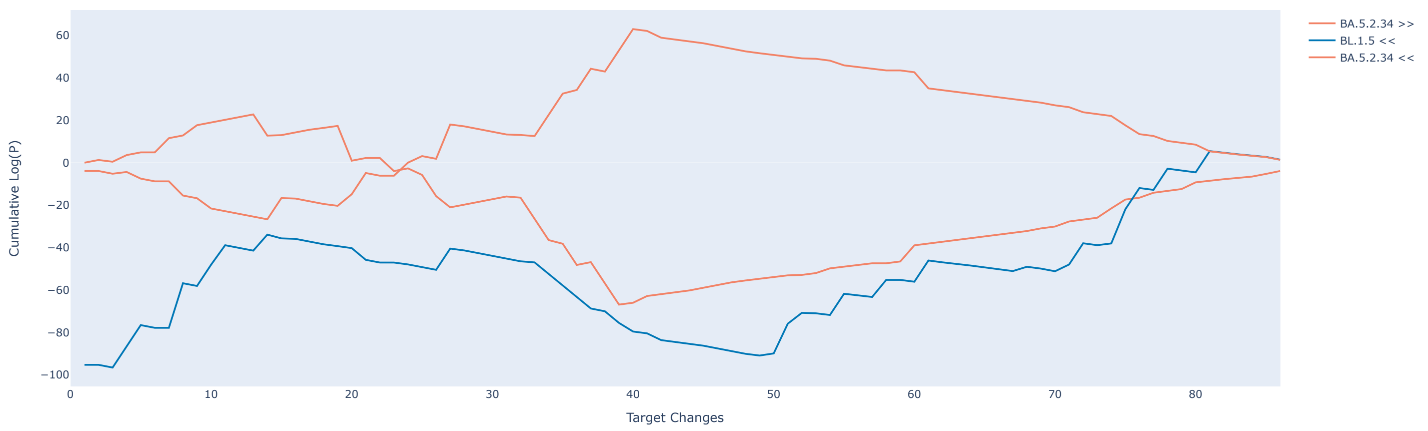


**Target sequence**

, 241_C|T, 670_T|G, 829_C|T, 1627_C|T, 2790_C|T, 3037_C|T, 3207_A|G, 4184_G|A, 4288_G|A, 4321_C|T, 9344_C|T, 9424_A|G, 9534_C|T, 9866_C|T, 10029_C|T, 10198_C|T, 10447_G|A, 10449_C|A, 11288_11296, 12444_A|G, 12880_C|T, 14408_C|T, 15451_G|A, 15714_C|T, 17410_C|T, 17880_A|G, 18163_A|G, 19955_C|T, 20055_A|G, 21618_C|T, 21633_21641, 21987_G|A, 22001_A|G, 22016_T|C, 22033_C|A, 22190_A|G, 22200_T|G, 22331_G|A, 22577_G|C, 22578_G|A, 22599_G|C, 22674_C|T, 22679_T|C, 22686_C|T, 22688_A|G, 22775_G|A, 22786_A|C, 22813_G|T, 22882_T|G, 22917_T|G, 22992_G|A, 22995_C|A,

23013_A|C, 23018_T|G, 23055_A|G, 23063_A|T, 23075_T|C, 23403_A|G, 23525_C|T, 23587_G|A, 23599_T|G, 23604_C|A, 23854_C|A, 23948_G|T, 24424_A|T, 24469_T|A, 25000_C|T, 25584_C|T, 26060_C|T, 26270_C|T, 26529_G|A, 26577_C|G, 26709_G|A, 27012_C|T, 27513_C|T, 27807_C|T, 27889_C|T, 28271_A|T, 28311_C|T, 28330_A|G, 28362_28370, 28881_G|A, 28882_G|A, 28883_G|C, 29510_A|C, 29734_29759

## Case 52 (2BP): XBU    test: OK

Target: (75%) 5 samples
GT: BA.2.75* + BQ.1* + BA.2.75*
BC: BA.2.75.2 + BQ.1.1.19 + BA.2.75.2
Direction L1: >>
Alt. candidates: [], []
Model 1BP/2BP comparison:
Rec. model vs L1: 7.62e-72
Flags: Model_2BP_Best

Number of changes: 89
GT BR: 40-53, 70-75
BC BR: 51-52, 70-71
Initial region span: 1-51,52-70,71-89

2BP vs 1BP: 2.20e-16
Rec. model vs L2: 1.16e-300

GT BR coord: 22576 - 22894, 25415 - 26276
BC BR coord: 22892 - 22893, 25417 - 25418
Gap history (edge excluded): 51-71 ->

Rank L1 L2: 11 11 11

### BA.2.75.2 >>

| | num_seq | t_ch_MAX | max_CL | CL@BC_t_ch_MAX | aic | PV | PV_OK | t_ch_MAX_OK | phyl_OK |
|---|---|---|---|---|---|---|---|---|---|
| **BA.2.75.2** | 582 | 51 | 110.265324 | NaN | NaN | NaN | * | * | * |
| **CH.1.1** | 2870 | 89 | 86.172846 | 102.213915 | -48.427829 | 1.065673e-13 | | | |
| **CA.3** | 34 | 51 | 71.963992 | 93.779881 | -31.559763 | 2.313928e-17 | | * | * |
| **BM.1.1** | 104 | 51 | 71.734146 | 93.619109 | -31.238217 | 1.971800e-17 | | * | |
| **BA.2.75.3** | 47 | 89 | 67.111883 | 80.494137 | -4.988275 | 3.933215e-23 | | | |
| **CH.1.1.7** | 26 | 89 | 66.847520 | 82.889378 | -9.778756 | 4.314028e-22 | | | |
| **BM.4.1.1** | 124 | 51 | 65.242867 | 87.080512 | -18.161024 | 2.848242e-20 | | * | |
| **CA.3.1** | 148 | 51 | 62.298046 | 84.171901 | -12.343803 | 1.551600e-21 | | * | * |
| **BM.1.1.1** | 15 | 51 | 61.257585 | 83.103327 | -10.206654 | 5.348799e-22 | | * | |
| **BA.2.75.5** | 215 | 89 | 57.920611 | 78.981871 | -1.963742 | 8.645528e-24 | | | |

### BQ.1.1.19 >>

| | num_seq | t_ch_MAX | max_CL | CL@BC_t_ch_MAX | aic | PV | PV_OK | t_ch_MAX_OK | phyl_OK |
|---|---|---|---|---|---|---|---|---|---|
| **BQ.1.1.19** | 144 | 70 | 21.608165 | NaN | NaN | NaN | * | * | * |
| **BQ.1.1.20** | 14 | 70 | 21.608165 | 21.608165 | -1.216330 | 1.000000 | * | * | |
| **BQ.1.1.21** | 38 | 70 | 21.608165 | 21.608165 | -1.216330 | 1.000000 | * | * | |
| **BQ.1.1.27** | 125 | 70 | 21.608165 | 21.608165 | -1.216330 | 1.000000 | * | * | |
| **BQ.1.1.37** | 13 | 70 | 21.608165 | 21.608165 | -1.216330 | 1.000000 | * | * | |
| **BQ.1.1.44** | 16 | 70 | 21.608165 | 21.608165 | -1.216330 | 1.000000 | * | * | |
| **BQ.1.1.45** | 99 | 70 | 21.608165 | 21.608165 | -1.216330 | 1.000000 | * | * | |
| **BQ.1.1.47** | 27 | 70 | 21.608165 | 21.608165 | -1.216330 | 1.000000 | * | * | |
| **BQ.1.1.48** | 78 | 70 | 21.608165 | 21.608165 | -1.216330 | 1.000000 | * | * | |
| **BQ.1.1.53** | 15 | 70 | 21.608165 | 21.608165 | -1.216330 | 1.000000 | * | * | |
| **BQ.1.1.55** | 11 | 70 | 21.608165 | 12.326493 | 17.347014 | 0.000093 | * | * | |
| **BQ.1.1.72** | 50 | 70 | 21.608165 | 21.608165 | -1.216330 | 1.000000 | * | * | |
| **BQ.1.26** | 57 | 70 | 21.608165 | 21.608165 | -1.216330 | 1.000000 | * | * | |
| **BQ.1.26.1** | 15 | 70 | 21.608165 | 21.608165 | -1.216330 | 1.000000 | * | * | |
| **BQ.1.30** | 18 | 70 | 21.608165 | 21.608165 | -1.216330 | 1.000000 | * | * | |
| **BQ.1.31** | 10 | 70 | 21.608165 | 21.608165 | -1.216330 | 1.000000 | * | * | |
| **BQ.1.7** | 69 | 70 | 21.608165 | 21.608165 | -1.216330 | 1.000000 | * | * | |
| **DK.1** | 67 | 70 | 21.608165 | 21.608165 | -1.216330 | 1.000000 | * | * | |
| **DM.1** | 15 | 70 | 21.608165 | 10.047877 | 21.904246 | 0.000010 | | * | |
| **DN.1.1** | 169 | 70 | 21.608165 | 21.608165 | -1.216330 | 1.000000 | * | * | |
| **DP.1** | 10 | 70 | 21.608165 | 21.608165 | -1.216330 | 1.000000 | * | * | |
| **DT.3** | 38 | 70 | 21.608165 | 21.608165 | -1.216330 | 1.000000 | * | * | |
| **EA.1** | 22 | 70 | 21.608165 | 21.608165 | -1.216330 | 1.000000 | * | * | |
| **ED.1** | 21 | 70 | 21.608165 | 21.608165 | -1.216330 | 1.000000 | * | * | |
| **ED.2** | 191 | 70 | 21.608165 | 21.608165 | -1.216330 | 1.000000 | * | * | |
| **ER.1** | 47 | 70 | 21.608165 | 21.608165 | -1.216330 | 1.000000 | * | * | |
| **ER.1.1** | 34 | 70 | 21.608165 | 21.608165 | -1.216330 | 1.000000 | * | * | |
| **ET.1** | 40 | 70 | 21.608165 | 21.608165 | -1.216330 | 1.000000 | * | * | |

### BA.2.75.2 <<

| | num_seq | t_ch_MAX | max_CL | CL@BC_t_ch_MAX | aic | PV | PV_OK | t_ch_MAX_OK | phyl_OK |
|---|---|---|---|---|---|---|---|---|---|
| **BA.2.75.2** | 582 | 71 | 21.819831 | None | None | None | * | * | * |

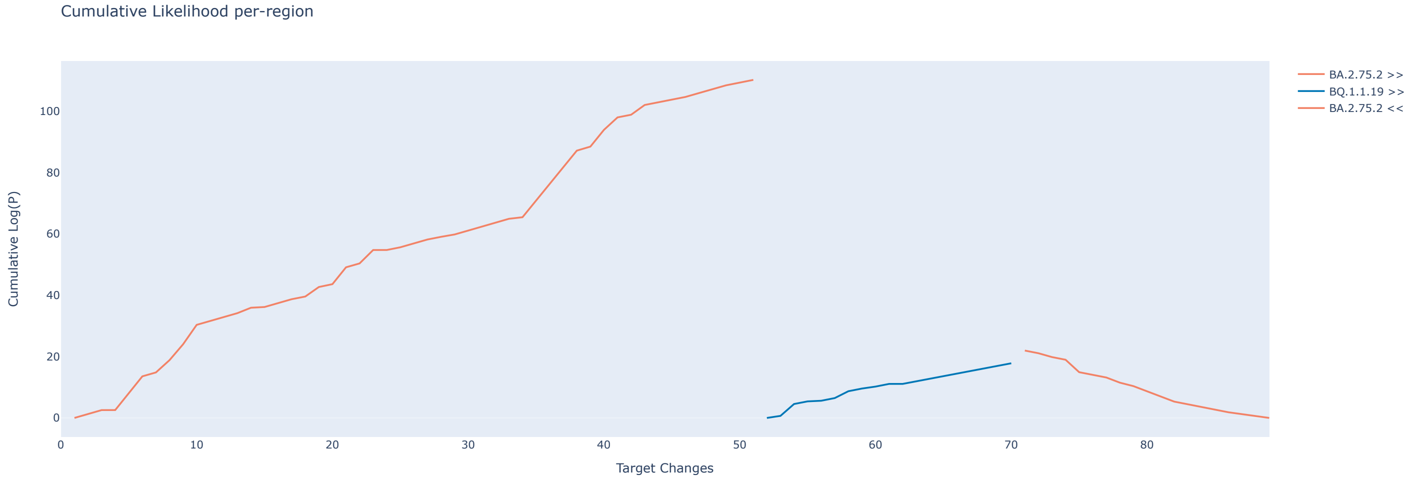


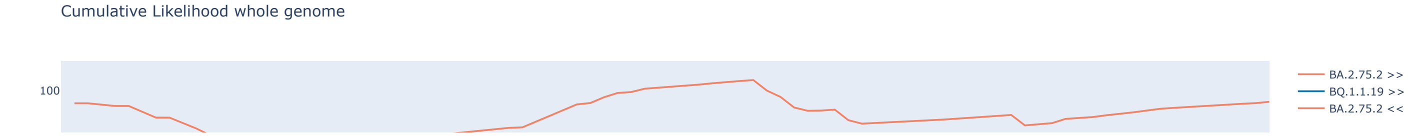

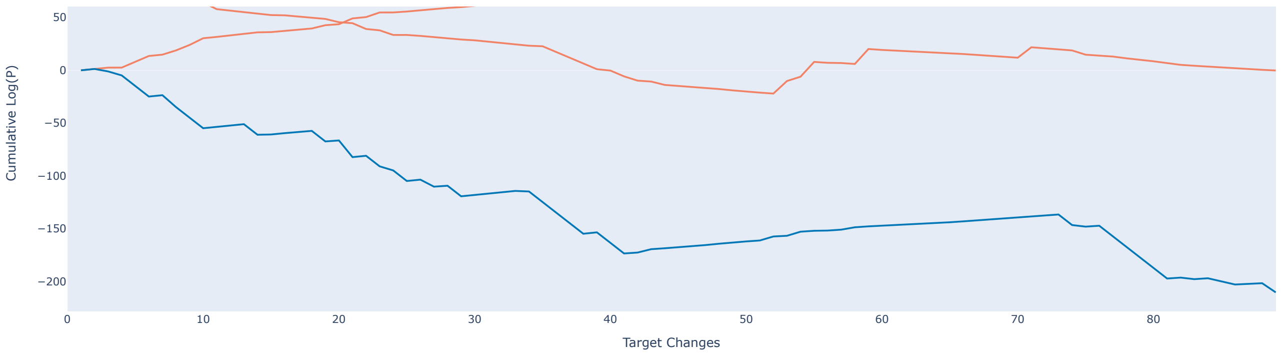


**Target sequence**

, 241_C|T, 670_T|G, 2790_C|T, 3037_C|T, 3796_C|T, 3927_C|T, 4184_G|A, 4586_C|T, 5183_C|T, 5192_C|T, 9344_C|T, 9424_A|G, 9534_C|T, 9866_C|T, 10029_C|T, 10198_C|T, 10447_G|A, 10449_C|A, 11124_C|T, 11288_11296, 12444_A|G, 12880_C|T, 13324_T|C, 14408_C|T, 15451_G|A, 15714_C|T, 17410_C|T, 18163_A|G, 18583_G|A, 19955_C|T, 20055_A|G, 21618_C|T, 21633_21641, 21987_G|A, 22001_A|G, 22016_T|C, 22033_C|A, 22190_A|G, 22200_T|G, 22331_G|A, 22577_G|C, 22578_G|A, 22599_G|C, 22674_C|T, 22679_T|C, 22686_C|T, 22688_A|G, 22775_G|A, 22786_A|C, 22813_G|T, 22882_T|G, 22893_A|C, 22917_T|G, 22942_T|A, 22992_G|A, 22995_C|A, 23013_A|C, 23018_T|G, 23055_A|G, 23063_A|T, 23075_T|C, 23403_A|G, 23525_C|T, 23599_T|G, 23604_C|A, 23854_C|A, 23948_G|T, 24424_A|T, 24469_T|A, 25000_C|T, 25584_C|T, 26060_C|T, 26270_C|T, 26275_A|G, 26577_C|G, 26709_G|A, 26858_C|T, 27259_A|C, 27382_G|C, 27383_A|T, 27384_T|C, 27807_C|T, 28271_A|T, 28311_C|T, 28362_28370, 28881_G|A, 28882_G|A, 28883_G|C, 29510_A|C

## Case 53 (2BP): XD        test: OK

Target: (75%) 10 samples    Number of changes: 69
GT: B.1.617.2* + BA.1* + B.1.617.2*    GT BR: 25–27, 53–55    GT BR coord: 22075 – 22236, 24999 – 25481    Rank L1 L2: 2 11 11
BC: AY.4 + BA.1.15.3 + AY.4    BC BR: 25–26, 54–55    BC BR coord: 22029 – 22030, 25470 – 25471
Direction L1: >>    Initial region span: 1–25,26–54,55–69    Gap history (edge excluded): 25–55 –>
Alt. candidates: [], []
Model 1BP/2BP comparison:    2BP vs 1BP: 1.26e–117
Rec. model vs L1: 1.93e–166    Rec. model vs L2: 0.00e+00
Flags: Model_2BP_Best

### AY.4 >>

| | num_seq | t_ch_MAX | max_CL | CL@BC_t_ch_MAX | aic | PV | PV_OK | t_ch_MAX_OK | phyl_OK |
|---|---|---|---|---|---|---|---|---|---|
| **AY.4** | 410971 | 25 | 21.436845 | NaN | NaN | NaN | * | * | * |
| **B.1.617.2** | 41469 | 25 | 4.136564 | 9.359597 | 61.280807 | 2.118802e-10 | | * | * |
| **AY.5** | 24784 | 7 | 1.831471 | -25.703646 | 131.407292 | 1.251856e-25 | | | |

### BA.1.15.3 >>

| | num_seq | t_ch_MAX | max_CL | CL@BC_t_ch_MAX | aic | PV | PV_OK | t_ch_MAX_OK | phyl_OK |
|---|---|---|---|---|---|---|---|---|---|
| **BA.1.15.3** | 836 | 54 | 30.764532 | NaN | NaN | NaN | * | * | * |
| **BA.1.22** | 1571 | 54 | 30.558068 | 30.558068 | 0.883863 | 0.814647 | * | * | |
| **BA.1.19** | 691 | 54 | 30.431643 | 30.431643 | 1.136714 | 0.715338 | * | * | |
| **BA.1.21** | 556 | 54 | 30.398704 | 30.398704 | 1.202592 | 0.694197 | * | * | |
| **BA.1.24** | 96 | 54 | 29.308507 | 29.308507 | 3.382985 | 0.233400 | * | * | |
| **BA.1.10** | 459 | 54 | 29.274272 | 29.274272 | 3.451456 | 0.225373 | * | * | |
| **BA.1.7** | 141 | 54 | 29.262391 | 29.262391 | 3.475218 | 0.222017 | * | * | |
| **BA.1.16** | 3129 | 54 | 29.232004 | 29.232004 | 3.535992 | 0.215456 | * | * | |
| **BA.1.16.1** | 68 | 54 | 29.209035 | 19.908314 | 22.183371 | 0.000019 | * | * | |
| **BA.1.14.2** | 52 | 54 | 29.062986 | 29.062986 | 3.874029 | 0.182684 | * | * | |

### AY.4 <<

| | num_seq | t_ch_MAX | max_CL | CL@BC_t_ch_MAX | aic | PV | PV_OK | t_ch_MAX_OK | phyl_OK |
|---|---|---|---|---|---|---|---|---|---|
| **AY.4** | 410971 | 55 | 10.199825 | None | None | None | * | * | * |

Cumulative Likelihood per-region

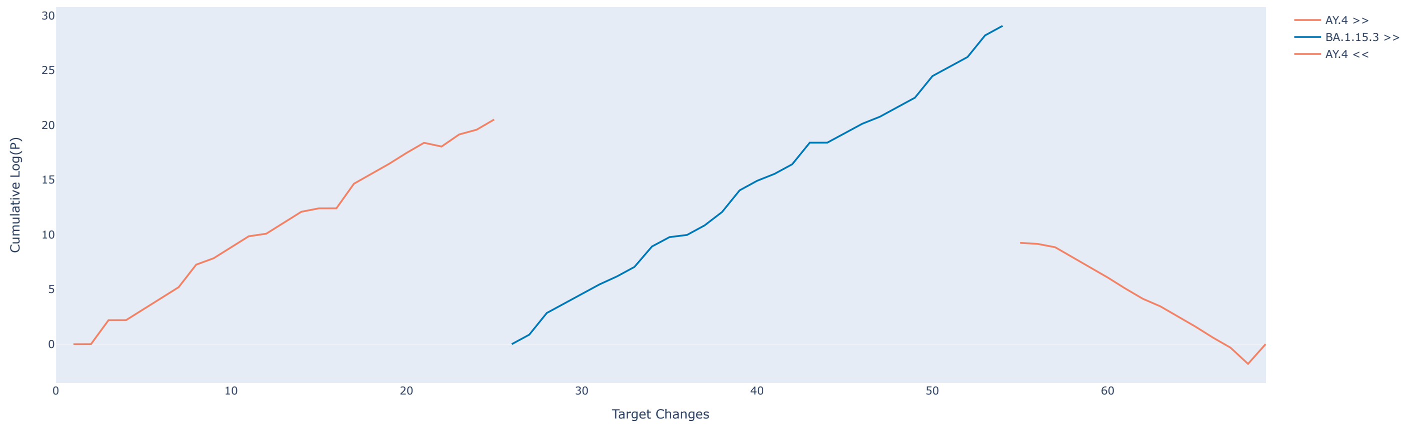


Cumulative Likelihood whole genome

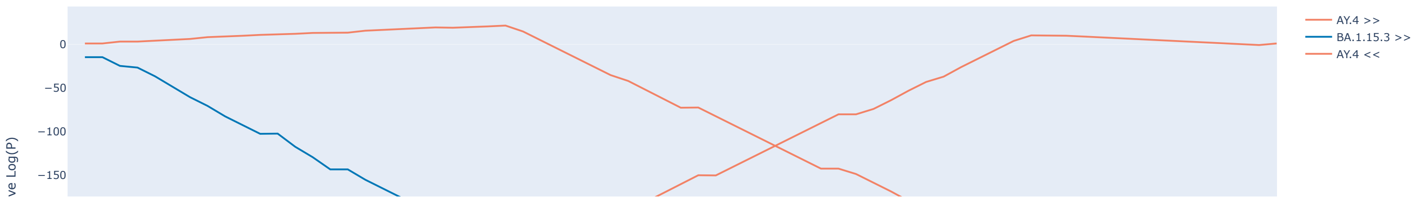

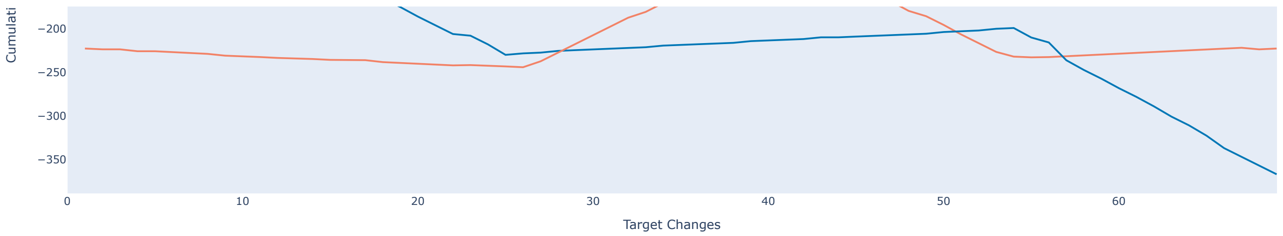


**Target sequence**

, 210_G|T, 241_C|T, 1321_A|C, 3037_C|T, 4181_G|T, 6402_C|T, 7124_C|T, 7851_C|T, 8723_A|G, 8986_C|T, 9053_G|T, 10029_C|T, 11201_A|G, 11332_A|G, 14407_C|T, 14408_C|T, 15264_T|C, 15451_G|A, 16466_C|T, 19220_C|T, 21618_C|G, 21641_G|T, 21846_C|T, 21987_G|A, 22029_22034, 22194_22196, 22578_G|A, 22673_T|C, 22674_C|T, 22679_T|C, 22686_C|T, 22813_G|T, 22882_T|G, 22898_G|A, 22992_G|A, 22995_C|A, 23013_A|C, 23040_A|G, 23048_G|A, 23055_A|G, 23063_A|T, 23075_T|C, 23202_C|A, 23403_A|G, 23525_C|T, 23599_T|G, 23604_C|A, 23854_C|A, 23948_G|T, 24130_C|A, 24424_A|T, 24469_T|A, 24503_C|T, 25000_C|T, 25667_C|T, 25855_G|T, 26767_T|C, 27638_T|C, 27752_C|T, 27874_C|T, 28248_28253, 28271_28271, 28461_A|G, 28881_G|T, 28916_G|T, 29402_G|T, 29540_G|A, 29645_G|T, 29742_G|T

## Case 54 (undefined): XAJ    test: KO

Target: (75%) 25 samples
Number of changes: 77
GT: BA.2.12.1* + BA.4*
GT BR: –
GT BR coord: –
Rank L1 L2: 4 –
BC: B.1.1.529
BC BR:
BC BR coord:
Direction L1: >>
Initial region span: 1–77
Gap history (edge excluded):
Alt. candidates: []
Model 1BP/2BP comparison: –
Rec. model vs L1: –
Rec. model vs L2: –
Flags: NotEnoughSpaceAfterL1, SingleCandidateGenome

## B.1.1.529 >>

| | num_seq | t_ch_MAX | max_CL | CL@BC_t_ch_MAX | aic | PV | PV_OK | t_ch_MAX_OK | phyl_OK |
|---|---|---|---|---|---|---|---|---|---|
| **B.1.1.529** | 248 | 77 | 65.841898 | NaN | NaN | NaN | * | * | * |
| **BA.4** | 6396 | 68 | 38.536525 | 37.712162 | 108.575677 | 6.071503e-13 | | | * |
| **BA.2.12** | 21 | 77 | 36.928900 | 36.928900 | 110.142199 | 2.783214e-13 | | * | * |
| **BA.2.12.1** | 94944 | 77 | 36.804841 | 36.804841 | 110.390318 | 2.456177e-13 | | * | * |
| **BA.5** | 3423 | 51 | 31.359892 | 11.097671 | 161.804659 | 1.685466e-24 | | | * |
| **BA.5.1** | 33374 | 48 | 27.746619 | -9.649272 | 203.298544 | 1.641004e-33 | | | * |
| **BA.5.5** | 22209 | 48 | 27.711915 | -10.435744 | 204.871488 | 7.484941e-34 | | | * |
| **BA.2** | 359165 | 77 | 25.421331 | 25.421331 | 133.157337 | 2.791368e-18 | | * | * |
| **BA.4.4** | 2242 | 68 | 24.999956 | 24.286812 | 135.426375 | 8.972073e-19 | | | * |
| **BG.5** | 563 | 26 | 23.943271 | 17.502570 | 148.994859 | 1.019474e-21 | | | * |

Cumulative Likelihood per-region

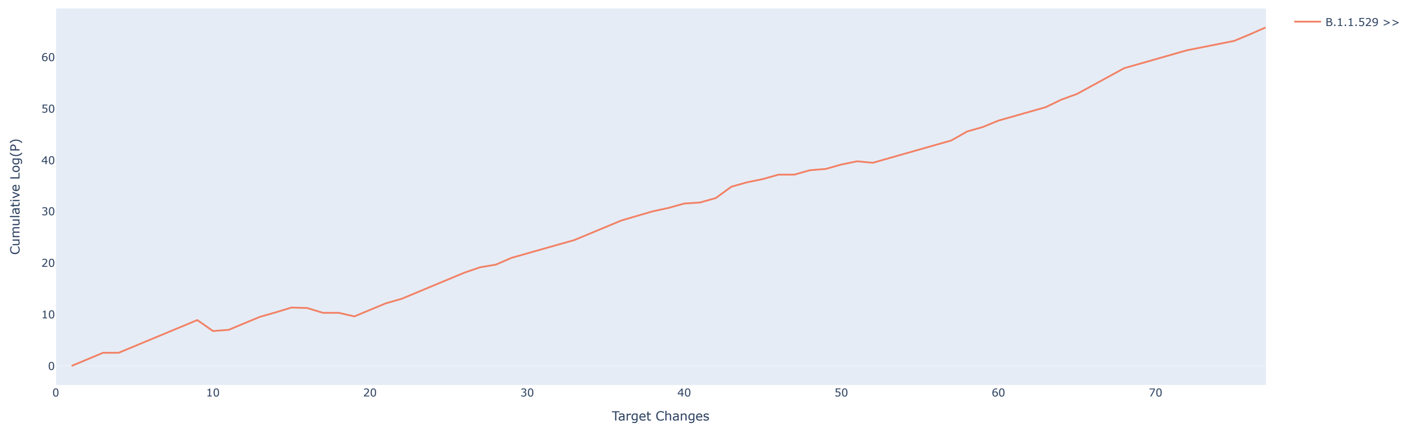


Cumulative Likelihood whole genome

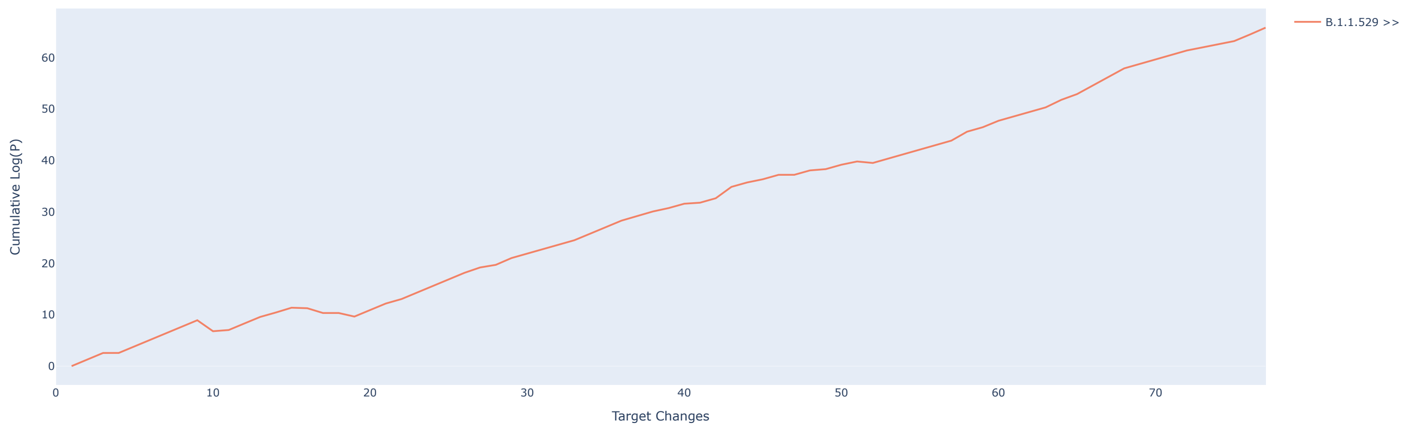

**Target sequence**

, 241_C|T, 670_T|G, 2790_C|T, 3037_C|T, 4184_G|A, 4321_C|T, 9344_C|T, 9424_A|G, 9534_C|T, 9866_C|T, 10029_C|T, 10198_C|T, 10447_G|A, 10449_C|A, 11288_11296, 11674_C|T, 12880_C|T, 14408_C|T, 15009_T|C, 15714_C|T, 17410_C|T, 18163_A|G, 19955_C|T, 20055_A|G, 21618_C|T, 21633_21641, 21765_21770, 21987_G|A, 22200_T|G, 22578_G|A, 22674_C|T, 22679_T|C, 22686_C|T, 22688_A|G, 22775_G|A, 22786_A|C, 22813_G|T, 22882_T|G, 22917_T|G, 22992_G|A, 22995_C|A, 23013_A|C, 23018_T|G, 23055_A|G, 23063_A|T, 23075_T|C, 23403_A|G, 23525_C|T, 23535_A|G, 23599_T|G, 23604_C|A, 23673_C|T, 23854_C|A, 23948_G|T, 24424_A|T, 24469_T|A, 25000_C|T, 25416_C|T, 25584_C|T, 26060_C|T, 26270_C|T, 26577_C|G, 26709_G|A, 26858_C|T, 27259_A|C, 27382_G|C, 27383_A|T, 27384_T|C, 27807_C|T, 28271_A|T, 28311_C|T, 28362_28370, 28881_G|A, 28882_G|A, 28883_G|C, 29510_A|C, 29734_29759

## Case 55 (undefined): XAY    test: KO

Target: (75%) 1 samples
Number of changes: 82
GT: BA.2* + AY.45 + BA.2* + AY.45 + BA.2*
GT BR: –
GT BR coord: –
Rank L1 L2: 11 11 – – –
BC: BA.5.1.27 + BA.2.38.1
BC BR: 72–73
BC BR coord: 27387 – 27388
Direction L1: <<
Initial region span: 1–7,74–82
Gap history (edge excluded): 7–74
Alt. candidates: [BA.5], []
Model 1BP/2BP comparison:
1BP vs 2BP: 2.38e–37
Rec. model vs L1: 2.75e–37
Rec. model vs L2: 2.83e–91
Flags: Model_1BP_Best

### BA.5.1.27 >>

| | num_seq | t_ch_MAX | max_CL | CL@BC_t_ch_MAX | aic | PV | PV_OK | t_ch_MAX_OK | phyl_OK |
|---|---|---|---|---|---|---|---|---|---|
| **BA.5.1.27** | 1123 | 7 | 6.601941 | NaN | NaN | NaN | * | * | * |
| **BA.2.3.1** | 111 | 7 | 6.099049 | -346.898187 | 931.796374 | 4.106425e-30 | | * | |
| **CL.1** | 246 | 7 | 5.968566 | -333.473130 | 904.946261 | 2.778864e-24 | | * | |
| **BA.5.5** | 22209 | 7 | 5.727064 | -170.534525 | 579.069050 | 1.613653e+47 | * | * | |
| **BA.5.1.6** | 980 | 7 | 5.715707 | -298.794478 | 835.588956 | 3.200368e-09 | | * | |
| **BA.2.76** | 356 | 7 | 5.624578 | -327.666340 | 893.332679 | 9.270822e-22 | | * | |
| **BA.5** | 3423 | 7 | 5.439354 | -216.836794 | 671.673588 | 1.258855e+27 | * | * | * |
| **BA.5.5.1** | 886 | 7 | 5.397958 | -246.011105 | 730.022210 | 2.688030e+14 | * | * | |
| **BA.2.13** | 1138 | 7 | 5.383878 | -276.362798 | 790.725597 | 1.763702e+01 | * | * | |
| **B.1.1.529** | 248 | 9 | 5.338225 | -283.425533 | 804.851067 | 1.514628e-02 | * | | * |

### BA.2.38.1 <<

| | num_seq | t_ch_MAX | max_CL | CL@BC_t_ch_MAX | aic | PV | PV_OK | t_ch_MAX_OK | phyl_OK |
|---|---|---|---|---|---|---|---|---|---|
| **BA.2.38.1** | 34 | 74 | 11.214450 | NaN | NaN | NaN | * | * | * |
| **CH.1.1.3** | 35 | 74 | 8.777132 | -1.222868 | 30.445735 | 0.087161 | * | * | |
| **CH.1.1.23** | 10 | 74 | 8.643601 | -1.356399 | 30.712798 | 0.076536 | * | * | |
| **BN.1.3.6** | 217 | 74 | 8.563558 | -1.436442 | 30.872883 | 0.070651 | * | * | |
| **BF.17** | 10 | 77 | 8.182319 | -4.722935 | 37.445871 | 0.002632 | * | | |
| **BA.2.12** | 21 | 74 | 7.901664 | -2.098336 | 32.196673 | 0.036334 | * | * | |
| **BA.5.2.58** | 79 | 77 | 7.831357 | -9.238852 | 46.477704 | 0.000029 | * | | |
| **BA.2.36** | 1320 | 74 | 7.594308 | -2.405692 | 32.811385 | 0.026783 | * | * | |
| **BR.1.2** | 98 | 74 | 7.512199 | -2.487801 | 32.975602 | 0.024600 | * | * | |
| **BN.5** | 33 | 74 | 7.480450 | -2.519550 | 33.039099 | 0.023873 | * | * | |

Cumulative Likelihood per-region

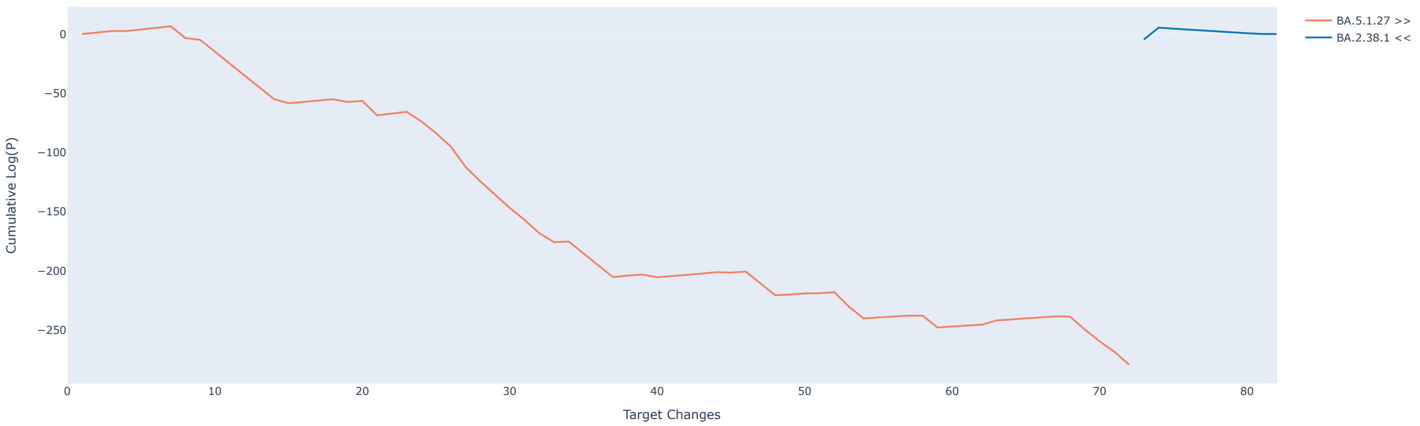


Cumulative Likelihood whole genome

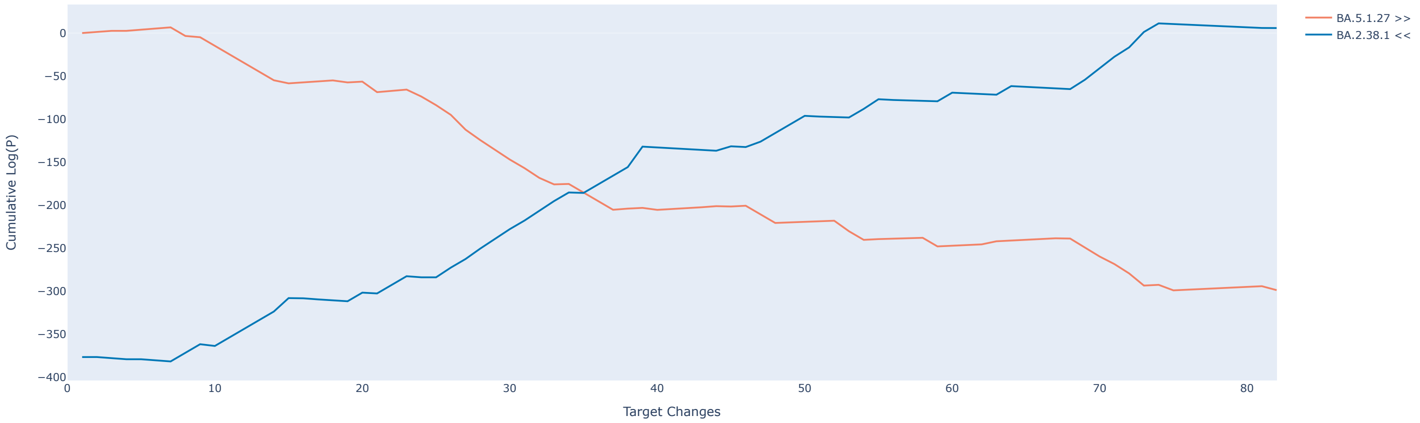

**Target sequence**

, 241_C|T, 670_T|G, 2790_C|T, 3037_C|T, 4184_G|A, 4321_C|T, 4456_C|T, 4543_C|T, 5869_C|T, 6428_C|T, 7124_C|T, 8595_C|T, 8986_C|T, 9053_G|T, 10029_C|T, 10198_C|T, 10447_G|A, 10449_C|A, 11083_G|T, 11288_11296, 12163_G|A, 12747_C|T, 12880_C|T, 14408_C|T, 15451_G|A, 16466_C|T, 17288_C|T, 19220_C|T, 19972_C|T, 21614_C|T, 21618_C|G, 21623_A|G, 21846_C|T, 21987_G|A, 22017_G|T, 22029_22034, 22118_T|C, 22200_T|G, 22578_G|A, 22674_C|T, 22679_T|C, 22686_C|T, 22688_A|G, 22775_G|A, 22813_G|T, 22882_T|G, 22899_G|A, 22916_C|A, 22917_T|G, 22992_G|A, 22995_C|A, 23013_A|C, 23018_T|C, 23019_T|C, 23055_A|G, 23063_A|T, 23075_T|C, 23403_A|G, 23423_C|T, 23525_C|T, 23599_T|G, 23604_C|A, 23679_C|T, 23854_C|A, 23948_G|T, 24424_A|T, 24469_T|A, 24912_C|T, 25413_C|T, 25469_C|T, 26681_C|T, 26767_T|C, 27575_C|G, 27807_C|T, 28271_A|T, 28311_C|T, 28362_28370, 28881_G|A, 28882_G|A, 28883_G|C, 29510_A|C, 29779_G|T

## Case 56 (undefined): XBK test: OK

Target: (75%) 58 samples
Number of changes: 92
GT: BA.5.2 + CJ.1
GT BR: –
GT BR coord: –
Rank L1 L2: 11 11
BC: BA.5.2.10 + CJ.1.1
BC BR: 3–4
BC BR coord: 1638 – 1639
Direction L1: <<
Initial region span: 1–3,4–92
Gap history (edge excluded):
Alt. candidates: [], [BM.1.1.1, BM.1.1]
Model 1BP/2BP comparison: –
Rec. model vs L1: 1.26e–321
Rec. model vs L2: 1.15e–17
Flags: Model_2BP_Bad_L1_opp

### BA.5.2.10 >>

| | num_seq | t_ch_MAX | max_CL | CL@BC_t_ch_MAX | aic | PV | PV_OK | t_ch_MAX_OK | phyl_OK |
|---|---|---|---|---|---|---|---|---|---|
| **BA.5.2.10** | 10 | 3 | 4.426274 | NaN | NaN | NaN | * | * | * |
| **BA.5.2.12** | 65 | 3 | 4.426274 | 4.426274 | -2.852549 | 1.0 | * | * | |
| **BA.5.2.30** | 19 | 3 | 4.426274 | 4.426274 | -2.852549 | 1.0 | * | * | |
| **BA.5.2.32** | 30 | 3 | 4.426274 | 4.426274 | -2.852549 | 1.0 | * | * | |
| **BA.5.2.38** | 13 | 3 | 4.426274 | 4.426274 | -2.852549 | 1.0 | * | * | |
| **BA.5.2.4** | 72 | 3 | 4.426274 | 4.426274 | -2.852549 | 1.0 | * | * | |
| **BA.5.2.41** | 11 | 3 | 4.426274 | 4.426274 | -2.852549 | 1.0 | * | * | |
| **BA.5.2.44** | 77 | 3 | 4.426274 | 4.426274 | -2.852549 | 1.0 | * | * | |
| **BA.5.2.52** | 33 | 3 | 4.426274 | 4.426274 | -2.852549 | 1.0 | * | * | |
| **BA.5.2.54** | 63 | 3 | 4.426274 | 4.426274 | -2.852549 | 1.0 | * | * | |
| **BA.5.2.58** | 79 | 3 | 4.426274 | 4.426274 | -2.852549 | 1.0 | * | * | |
| **BA.5.2.60** | 12 | 3 | 4.426274 | 4.426274 | -2.852549 | 1.0 | * | * | |
| **BA.5.2.61** | 11 | 3 | 4.426274 | 4.426274 | -2.852549 | 1.0 | * | * | |
| **BA.5.2.62** | 81 | 3 | 4.426274 | 4.426274 | -2.852549 | 1.0 | * | * | |
| **BA.5.2.63** | 11 | 3 | 4.426274 | 4.426274 | -2.852549 | 1.0 | * | * | |
| **BF.10.1** | 35 | 3 | 4.426274 | 4.426274 | -2.852549 | 1.0 | * | * | |
| **BF.12** | 81 | 3 | 4.426274 | 4.426274 | -2.852549 | 1.0 | * | * | |
| **BF.18** | 24 | 3 | 4.426274 | 4.426274 | -2.852549 | 1.0 | * | * | |
| **BF.19** | 14 | 3 | 4.426274 | 4.426274 | -2.852549 | 1.0 | * | * | |
| **BF.20** | 26 | 3 | 4.426274 | 4.426274 | -2.852549 | 1.0 | * | * | |
| **BF.22** | 131 | 3 | 4.426274 | 4.426274 | -2.852549 | 1.0 | * | * | |
| **BF.23** | 32 | 3 | 4.426274 | 4.426274 | -2.852549 | 1.0 | * | * | |
| **BF.24** | 52 | 3 | 4.426274 | 4.426274 | -2.852549 | 1.0 | * | * | |
| **BF.34** | 16 | 3 | 4.426274 | 4.426274 | -2.852549 | 1.0 | * | * | |
| **BF.38** | 26 | 3 | 4.426274 | 4.426274 | -2.852549 | 1.0 | * | * | |
| **BF.40** | 51 | 3 | 4.426274 | 4.426274 | -2.852549 | 1.0 | * | * | |
| **BF.41** | 108 | 3 | 4.426274 | 4.426274 | -2.852549 | 1.0 | * | * | |
| **BF.5.2** | 11 | 3 | 4.426274 | 4.426274 | -2.852549 | 1.0 | * | * | |
| **BF.5.4** | 11 | 3 | 4.426274 | 4.426274 | -2.852549 | 1.0 | * | * | |
| **BF.5.5** | 43 | 3 | 4.426274 | 4.426274 | -2.852549 | 1.0 | * | * | |
| **BF.7.1** | 18 | 3 | 4.426274 | 4.426274 | -2.852549 | 1.0 | * | * | |
| **BF.7.10** | 20 | 3 | 4.426274 | 4.426274 | -2.852549 | 1.0 | * | * | |
| **BF.7.12** | 51 | 3 | 4.426274 | 4.426274 | -2.852549 | 1.0 | * | * | |
| **BF.7.15** | 47 | 3 | 4.426274 | 4.426274 | -2.852549 | 1.0 | * | * | |
| **BF.7.16** | 15 | 3 | 4.426274 | 4.426274 | -2.852549 | 1.0 | * | * | |
| **BF.7.19.1** | 13 | 3 | 4.426274 | 4.426274 | -2.852549 | 1.0 | * | * | |
| **BF.7.20** | 98 | 3 | 4.426274 | 4.426274 | -2.852549 | 1.0 | * | * | |
| **BF.7.3** | 12 | 3 | 4.426274 | 4.426274 | -2.852549 | 1.0 | * | * | |
| **BF.7.6** | 260 | 3 | 4.426274 | 4.426274 | -2.852549 | 1.0 | * | * | |
| **BF.7.8** | 23 | 3 | 4.426274 | 4.426274 | -2.852549 | 1.0 | * | * | |
| **BV.1** | 39 | 3 | 4.426274 | 4.426274 | -2.852549 | 1.0 | * | * | |
| **BV.2** | 30 | 3 | 4.426274 | 4.426274 | -2.852549 | 1.0 | * | * | |
| **CD.1** | 31 | 3 | 4.426274 | 4.426274 | -2.852549 | 1.0 | * | * | |
| **CE.1** | 66 | 3 | 4.426274 | 4.426274 | -2.852549 | 1.0 | * | * | |
| **CF.1** | 42 | 3 | 4.426274 | 4.426274 | -2.852549 | 1.0 | * | * | |
| **CG.1** | 42 | 3 | 4.426274 | 4.426274 | -2.852549 | 1.0 | * | * | |
| **CK.1.2** | 31 | 3 | 4.426274 | 4.426274 | -2.852549 | 1.0 | * | * | |
| **CK.2.1** | 153 | 3 | 4.426274 | 4.426274 | -2.852549 | 1.0 | * | * | |
| **CK.3** | 78 | 3 | 4.426274 | 4.426274 | -2.852549 | 1.0 | * | * | |
| **CN.1** | 149 | 3 | 4.426274 | 4.426274 | -2.852549 | 1.0 | * | * | |
| **CP.2** | 34 | 3 | 4.426274 | 4.426274 | -2.852549 | 1.0 | * | * | |
| **CP.6** | 12 | 3 | 4.426274 | 4.426274 | -2.852549 | 1.0 | * | * | |
| **CR.1** | 77 | 3 | 4.426274 | 4.426274 | -2.852549 | 1.0 | * | * | |
| **CR.2** | 14 | 3 | 4.426274 | 4.426274 | -2.852549 | 1.0 | * | * | |
| **DG.1** | 39 | 3 | 4.426274 | 4.426274 | -2.852549 | 1.0 | * | * | |
| **DQ.1** | 44 | 3 | 4.426274 | 4.426274 | -2.852549 | 1.0 | * | * | |
| **DY.2** | 30 | 3 | 4.426274 | 4.426274 | -2.852549 | 1.0 | * | * | |
| **XBF.7** | 10 | 3 | 4.426274 | 4.426274 | -2.852549 | 1.0 | * | * | |
| **XBF.7.1** | 10 | 3 | 4.426274 | 4.426274 | -2.852549 | 1.0 | * | * | |

### CJ.1.1 <<

| | num_seq | t_ch_MAX | max_CL | CL@BC_t_ch_MAX | aic | PV | PV_OK | t_ch_MAX_OK | phyl_OK |
|---|---|---|---|---|---|---|---|---|---|
| **CJ.1.1** | 15 | 4 | 140.528222 | NaN | NaN | NaN | * | * | * |
| **BM.1.1.1** | 15 | 4 | 135.554759 | 135.554759 | -65.109517 | 6.908519e-03 | * | * | * |
| **CJ.1.2** | 43 | 4 | 133.498860 | 133.498860 | -60.997721 | 8.849318e-04 | * | * | |
| **BM.1.1** | 104 | 4 | 131.434903 | 131.434903 | -56.869806 | 1.122255e-04 | * | * | * |
| **XBF.3** | 37 | 12 | 125.094866 | 56.217779 | 93.564442 | 2.424556e-37 | | | |
| **XBF.5** | 14 | 12 | 125.075556 | 58.812062 | 88.375876 | 3.231878e-36 | | | |
| **XBF.6** | 10 | 23 | 119.336323 | 55.422074 | 95.155852 | 1.089423e-37 | | | |
| **XBF.7** | 10 | 12 | 118.628307 | 59.022168 | 87.955663 | 3.987097e-36 | | | |
| **XBF.4** | 38 | 12 | 117.266735 | 48.731818 | 108.536363 | 1.354461e-40 | | | |
| **BM.1.1.3** | 259 | 6 | 116.637014 | 109.585899 | -13.171798 | 3.637117e-14 | | | |

Cumulative Likelihood per-region

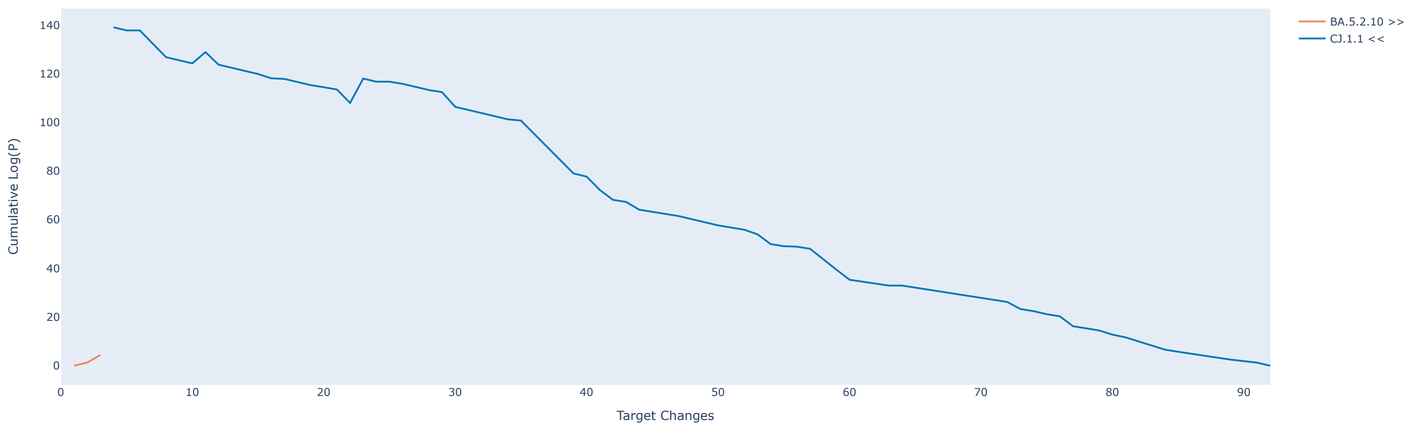


Cumulative Likelihood whole genome

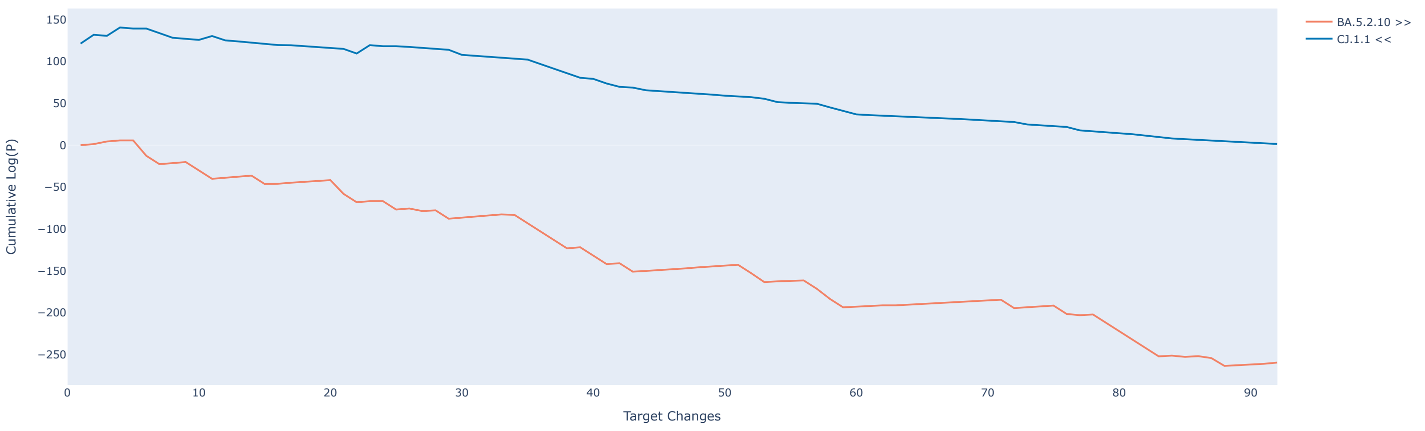


**Target sequence**

, 241_C|T, 670_T|G, 1627_C|T, 2790_C|T, 3037_C|T, 3796_C|T, 3927_C|T, 4184_G|A, 4321_C|T, 4586_C|T, 5183_C|T, 9344_C|T, 9424_A|G, 9534_C|T, 9866_C|T, 10029_C|T, 10198_C|T, 10447_G|A, 10449_C|A, 11288_11296, 12444_A|G, 12574_T|C, 12880_C|T, 14408_C|T, 15451_G|A, 15714_C|T, 17410_C|T, 18163_A|G, 18583_G|A, 19955_C|T, 20055_A|G, 21618_C|T, 21633_21641, 21987_G|A, 22001_A|G, 22016_T|C, 22033_C|A, 22190_A|G, 22200_T|G, 22331_G|A, 22577_G|C, 22578_G|A, 22599_G|C, 22674_C|T, 22679_T|C, 22686_C|T, 22688_A|G, 22775_G|A, 22786_A|C, 22813_G|T, 22882_T|G, 22898_G|A, 22942_T|G, 22992_G|A, 22995_C|A, 23013_A|C, 23018_T|C, 23019_T|C, 23031_T|C, 23055_A|G, 23063_A|T, 23075_T|C, 23403_A|G, 23525_C|T, 23599_T|G, 23604_C|A, 23854_C|A, 23948_G|T, 24424_A|T, 24469_T|A, 25000_C|T, 25416_C|T, 25584_C|T, 26060_C|T, 26270_C|T, 26275_A|G, 26577_C|G, 26709_G|A, 26858_C|T, 27259_A|C, 27382_G|C, 27383_A|T, 27384_T|C, 27807_C|T, 28271_A|T, 28311_C|T, 28362_28370, 28881_G|A, 28882_G|A, 28883_G|C, 29510_A|C, 29734_29759

## Case 57 (undefined): XBN test: OK

Target: (75%) 19 samples
Number of changes: 98
GT: BA.2.75 + XBB.3
GT BR: –
GT BR coord: –
Rank L1 L2: 2 1
BC: BA.2.75.5 + XBB.3
BC BR: 11–12
BC BR coord: 5183 – 5184
Direction L1: <<
Initial region span: 1–11,12–98
Gap history (edge excluded):
Alt. candidates: [BA.2.75], [XBB.3.1]
Model 1BP/2BP comparison: –
Rec. model vs L1: 1.95e–197
Rec. model vs L2: 6.67e–53
Flags: Model_2BP_Bad_L1_opp

### BA.2.75.5 >>

| | num_seq | t_ch_MAX | max_CL | CL@BC_t_ch_MAX | aic | PV | PV_OK | t_ch_MAX_OK | phyl_OK |
|---|---|---|---|---|---|---|---|---|---|
| **BA.2.75.5** | 215 | 11 | 32.577642 | NaN | NaN | NaN | * | * | * |
| **BA.2.75** | 122 | 11 | 32.033848 | 32.033848 | -40.067696 | 5.798418e-01 | * | * | * |
| **CH.1.1** | 2870 | 11 | 22.434778 | 22.434778 | -20.869557 | 3.927195e-05 | * | * | |
| **BN.1.10** | 43 | 11 | 16.561731 | 16.561731 | -9.123462 | 1.103068e-07 | | * | * |
| **BM.1.1.5** | 11 | 11 | 16.561731 | 16.561731 | -9.123462 | 1.103068e-07 | | * | |
| **BN.1.4.1** | 60 | 11 | 16.561731 | 16.561731 | -9.123462 | 1.103068e-07 | | * | * |
| **BN.1.7** | 232 | 11 | 16.561731 | 16.561731 | -9.123462 | 1.103068e-07 | | * | * |
| **BN.2.1** | 19 | 11 | 16.561731 | 16.561731 | -9.123462 | 1.103068e-07 | | * | * |
| **BN.6** | 49 | 11 | 16.561731 | 16.561731 | -9.123462 | 1.103068e-07 | | * | * |
| **BR.4** | 11 | 11 | 16.561731 | 16.561731 | -9.123462 | 1.103068e-07 | | * | |

### XBB.3 <<

| | num_seq | t_ch_MAX | max_CL | CL@BC_t_ch_MAX | aic | PV | PV_OK | t_ch_MAX_OK | phyl_OK |
|---|---|---|---|---|---|---|---|---|---|
| **XBB.3** | 115 | 12 | 131.541445 | NaN | NaN | NaN | * | * | * |
| **XBB.1.5** | 35885 | 12 | 125.312425 | 125.312425 | -50.624851 | 1.969452e-03 | * | * | |
| **XBB.3.1** | 11 | 12 | 123.349761 | 123.349761 | -46.699521 | 2.774139e-04 | * | * | * |
| **XBB.2** | 470 | 12 | 120.338905 | 120.338905 | -40.677809 | 1.367420e-05 | * | * | |
| **XBB.3.3** | 22 | 12 | 116.967423 | 116.967423 | -33.934845 | 4.679053e-07 | | * | * |
| **XBB.3.2** | 51 | 12 | 116.797494 | 108.560613 | -17.121225 | 1.046918e-10 | | * | * |
| **XBB.1** | 1674 | 12 | 115.099358 | 115.099358 | -30.198716 | 7.247675e-08 | | * | |
| **XBB.1.5.18** | 338 | 12 | 110.033244 | 102.944594 | -5.889189 | 3.813724e-13 | | * | |
| **XBB.1.5.33** | 550 | 12 | 108.899073 | 108.899073 | -17.798145 | 1.470865e-10 | | * | |
| **XBB.2.6** | 246 | 12 | 108.300543 | 98.633864 | 2.732272 | 5.123185e-15 | | * | |

Cumulative Likelihood per-region

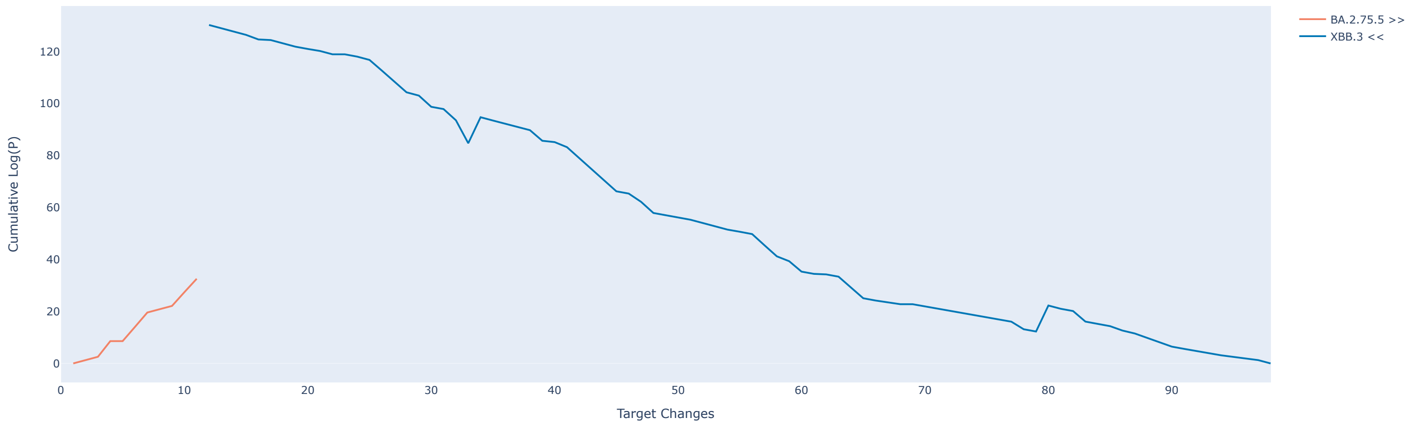


Cumulative Likelihood whole genome

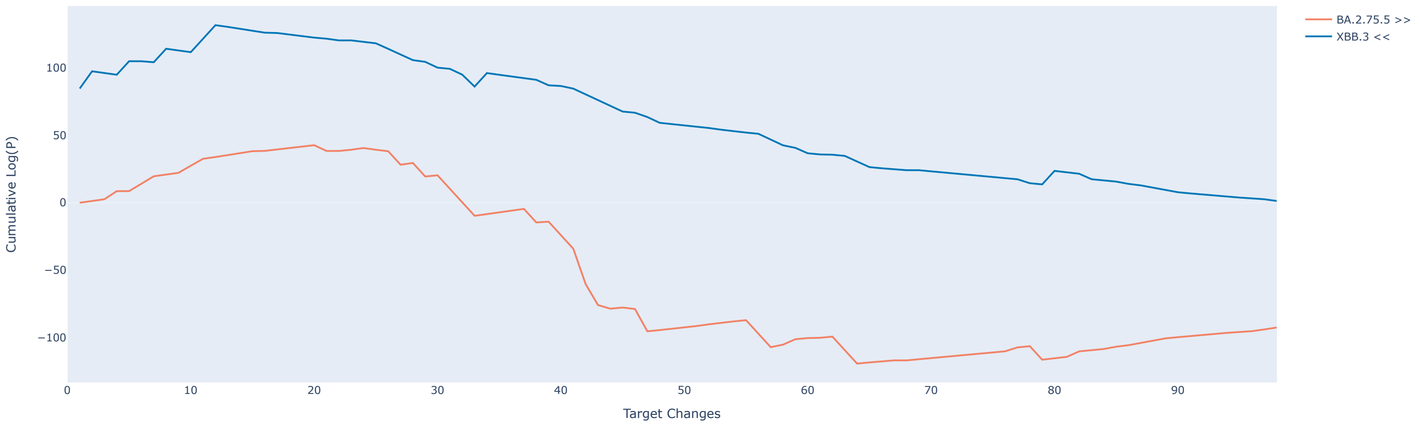


**Target sequence**

, 241_C|T, 670_T|G, 2790_C|T, 2863_A|G, 3037_C|T, 3796_C|T, 3927_C|T, 4184_G|A, 4321_C|T, 4586_C|T, 5183_C|T, 9344_C|T, 9424_A|G, 9534_C|T, 9866_C|T, 10029_C|T, 10198_C|T, 10447_G|A, 10449_C|A, 11288_11296, 12880_C|T, 14408_C|T, 15451_G|A, 15714_C|T, 15738_C|T, 15939_T|C, 16342_T|C, 17410_C|T, 17859_T|C, 18163_A|G, 19326_A|G, 19459_A|G, 19554_C|T, 19955_C|T, 20055_A|G, 21618_C|T, 21633_21641, 21810_T|C, 21987_G|A, 21992_21994, 22000_C|A, 22109_C|G, 22200_T|A, 22577_G|C, 22578_G|A, 22599_G|C, 22664_C|A, 22674_C|T, 22679_T|C, 22686_C|T, 22688_A|G, 22775_G|A, 22786_A|C, 22813_G|T, 22882_T|G, 22895_G|C, 22896_T|C, 22898_G|A, 22942_T|G, 22992_G|A, 22995_C|A, 23013_A|C, 23019_T|C, 23031_T|C, 23055_A|G, 23063_A|T, 23075_T|C, 23403_A|G, 23525_C|T, 23599_T|G, 23604_C|A, 23854_C|A, 23948_G|T, 24424_A|T, 24469_T|A, 25000_C|T, 25416_C|T, 25584_C|T, 25638_C|T, 26060_C|T, 26270_C|T, 26275_A|G, 26577_C|G, 26709_G|A, 26858_C|T, 27259_A|C, 27382_G|C, 27383_A|T, 27384_T|C, 27807_C|T, 28271_A|T, 28311_C|T, 28362_28370, 28881_G|A, 28882_G|A, 28883_G|C, 29510_A|C, 29734_29759

## Case 58 (undefined): XBQ    test: OK

Target: (75%) 20 samples
Number of changes: 92
GT: BA.5.2 + CJ.1
GT BR: –
GT BR coord: –
Rank L1 L2: 11 11
BC: BA.5.2.10 + CJ.1.1
BC BR: 3–4
BC BR coord: 1638 – 1639
Direction L1: <<
Initial region span: 1–3,4–92
Gap history (edge excluded):
Alt. candidates: [], [BM.1.1.1, BM.1.1]
Model 1BP/2BP comparison: –
Rec. model vs L1: 1.26e−321
Rec. model vs L2: 1.15e−17
Flags: Model_2BP_Bad_L1_opp

## BA.5.2.10 >>

| | num_seq | t_ch_MAX | max_CL | CL@BC_t_ch_MAX | aic | PV | PV_OK | t_ch_MAX_OK | phyl_OK |
|---|---|---|---|---|---|---|---|---|---|
| **BA.5.2.10** | 10 | 3 | 4.426274 | NaN | NaN | NaN | * | * | * |
| **BA.5.2.12** | 65 | 3 | 4.426274 | 4.426274 | -2.852549 | 1.0 | * | * | |
| **BA.5.2.30** | 19 | 3 | 4.426274 | 4.426274 | -2.852549 | 1.0 | * | * | |
| **BA.5.2.32** | 30 | 3 | 4.426274 | 4.426274 | -2.852549 | 1.0 | * | * | |
| **BA.5.2.38** | 13 | 3 | 4.426274 | 4.426274 | -2.852549 | 1.0 | * | * | |
| **BA.5.2.4** | 72 | 3 | 4.426274 | 4.426274 | -2.852549 | 1.0 | * | * | |
| **BA.5.2.41** | 11 | 3 | 4.426274 | 4.426274 | -2.852549 | 1.0 | * | * | |
| **BA.5.2.44** | 77 | 3 | 4.426274 | 4.426274 | -2.852549 | 1.0 | * | * | |
| **BA.5.2.52** | 33 | 3 | 4.426274 | 4.426274 | -2.852549 | 1.0 | * | * | |
| **BA.5.2.54** | 63 | 3 | 4.426274 | 4.426274 | -2.852549 | 1.0 | * | * | |
| **BA.5.2.58** | 79 | 3 | 4.426274 | 4.426274 | -2.852549 | 1.0 | * | * | |
| **BA.5.2.60** | 12 | 3 | 4.426274 | 4.426274 | -2.852549 | 1.0 | * | * | |
| **BA.5.2.61** | 11 | 3 | 4.426274 | 4.426274 | -2.852549 | 1.0 | * | * | |
| **BA.5.2.62** | 81 | 3 | 4.426274 | 4.426274 | -2.852549 | 1.0 | * | * | |
| **BA.5.2.63** | 11 | 3 | 4.426274 | 4.426274 | -2.852549 | 1.0 | * | * | |
| **BF.10.1** | 35 | 3 | 4.426274 | 4.426274 | -2.852549 | 1.0 | * | * | |
| **BF.12** | 81 | 3 | 4.426274 | 4.426274 | -2.852549 | 1.0 | * | * | |
| **BF.18** | 24 | 3 | 4.426274 | 4.426274 | -2.852549 | 1.0 | * | * | |
| **BF.19** | 14 | 3 | 4.426274 | 4.426274 | -2.852549 | 1.0 | * | * | |
| **BF.20** | 26 | 3 | 4.426274 | 4.426274 | -2.852549 | 1.0 | * | * | |
| **BF.22** | 131 | 3 | 4.426274 | 4.426274 | -2.852549 | 1.0 | * | * | |
| **BF.23** | 32 | 3 | 4.426274 | 4.426274 | -2.852549 | 1.0 | * | * | |
| **BF.24** | 52 | 3 | 4.426274 | 4.426274 | -2.852549 | 1.0 | * | * | |
| **BF.34** | 16 | 3 | 4.426274 | 4.426274 | -2.852549 | 1.0 | * | * | |
| **BF.38** | 26 | 3 | 4.426274 | 4.426274 | -2.852549 | 1.0 | * | * | |
| **BF.40** | 51 | 3 | 4.426274 | 4.426274 | -2.852549 | 1.0 | * | * | |
| **BF.41** | 108 | 3 | 4.426274 | 4.426274 | -2.852549 | 1.0 | * | * | |
| **BF.5.2** | 11 | 3 | 4.426274 | 4.426274 | -2.852549 | 1.0 | * | * | |
| **BF.5.4** | 11 | 3 | 4.426274 | 4.426274 | -2.852549 | 1.0 | * | * | |
| **BF.5.5** | 43 | 3 | 4.426274 | 4.426274 | -2.852549 | 1.0 | * | * | |
| **BF.7.1** | 18 | 3 | 4.426274 | 4.426274 | -2.852549 | 1.0 | * | * | |
| **BF.7.10** | 20 | 3 | 4.426274 | 4.426274 | -2.852549 | 1.0 | * | * | |
| **BF.7.12** | 51 | 3 | 4.426274 | 4.426274 | -2.852549 | 1.0 | * | * | |
| **BF.7.15** | 47 | 3 | 4.426274 | 4.426274 | -2.852549 | 1.0 | * | * | |
| **BF.7.16** | 15 | 3 | 4.426274 | 4.426274 | -2.852549 | 1.0 | * | * | |
| **BF.7.19.1** | 13 | 3 | 4.426274 | 4.426274 | -2.852549 | 1.0 | * | * | |
| **BF.7.20** | 98 | 3 | 4.426274 | 4.426274 | -2.852549 | 1.0 | * | * | |
| **BF.7.3** | 12 | 3 | 4.426274 | 4.426274 | -2.852549 | 1.0 | * | * | |
| **BF.7.6** | 260 | 3 | 4.426274 | 4.426274 | -2.852549 | 1.0 | * | * | |
| **BF.7.8** | 23 | 3 | 4.426274 | 4.426274 | -2.852549 | 1.0 | * | * | |
| **BV.1** | 39 | 3 | 4.426274 | 4.426274 | -2.852549 | 1.0 | * | * | |
| **BV.2** | 30 | 3 | 4.426274 | 4.426274 | -2.852549 | 1.0 | * | * | |
| **CD.1** | 31 | 3 | 4.426274 | 4.426274 | -2.852549 | 1.0 | * | * | |
| **CE.1** | 66 | 3 | 4.426274 | 4.426274 | -2.852549 | 1.0 | * | * | |
| **CF.1** | 42 | 3 | 4.426274 | 4.426274 | -2.852549 | 1.0 | * | * | |
| **CG.1** | 42 | 3 | 4.426274 | 4.426274 | -2.852549 | 1.0 | * | * | |
| **CK.1.2** | 31 | 3 | 4.426274 | 4.426274 | -2.852549 | 1.0 | * | * | |
| **CK.2.1** | 153 | 3 | 4.426274 | 4.426274 | -2.852549 | 1.0 | * | * | |
| **CK.3** | 78 | 3 | 4.426274 | 4.426274 | -2.852549 | 1.0 | * | * | |
| **CN.1** | 149 | 3 | 4.426274 | 4.426274 | -2.852549 | 1.0 | * | * | |
| **CP.2** | 34 | 3 | 4.426274 | 4.426274 | -2.852549 | 1.0 | * | * | |
| **CP.6** | 12 | 3 | 4.426274 | 4.426274 | -2.852549 | 1.0 | * | * | |
| **CR.1** | 77 | 3 | 4.426274 | 4.426274 | -2.852549 | 1.0 | * | * | |
| **CR.2** | 14 | 3 | 4.426274 | 4.426274 | -2.852549 | 1.0 | * | * | |
| **DG.1** | 39 | 3 | 4.426274 | 4.426274 | -2.852549 | 1.0 | * | * | |
| **DQ.1** | 44 | 3 | 4.426274 | 4.426274 | -2.852549 | 1.0 | * | * | |
| **DY.2** | 30 | 3 | 4.426274 | 4.426274 | -2.852549 | 1.0 | * | * | |
| **XBF.7** | 10 | 3 | 4.426274 | 4.426274 | -2.852549 | 1.0 | * | * | |
| **XBF.7.1** | 10 | 3 | 4.426274 | 4.426274 | -2.852549 | 1.0 | * | * | |

## CJ.1.1 <<

| | num_seq | t_ch_MAX | max_CL | CL@BC_t_ch_MAX | aic | PV | PV_OK | t_ch_MAX_OK | phyl_OK |
|---|---|---|---|---|---|---|---|---|---|
| **CJ.1.1** | 15 | 4 | 140.528222 | NaN | NaN | NaN | * | * | * |
| **BM.1.1.1** | 15 | 4 | 135.554759 | 135.554759 | -65.109517 | 6.908519e-03 | * | * | * |
| **CJ.1.2** | 43 | 4 | 133.498860 | 133.498860 | -60.997721 | 8.849318e-04 | * | * | |
| **BM.1.1** | 104 | 4 | 131.434903 | 131.434903 | -56.869806 | 1.122255e-04 | * | * | * |
| **XBF.3** | 37 | 12 | 125.094866 | 56.217779 | 93.564442 | 2.424556e-37 | | | |
| **XBF.5** | 14 | 12 | 125.075556 | 58.812062 | 88.375876 | 3.231878e-36 | | | |
| **XBF.7** | 10 | 12 | 118.628307 | 59.022168 | 87.955663 | 3.987097e-36 | | | |
| **XBF.6** | 10 | 25 | 118.066201 | 55.422074 | 95.155852 | 1.089423e-37 | | | |
| **XBF.4** | 38 | 12 | 117.266735 | 48.731818 | 108.536363 | 1.354461e-40 | | | |
| **BM.1.1.3** | 259 | 6 | 116.637014 | 109.585899 | -13.171798 | 3.637117e-14 | | | |

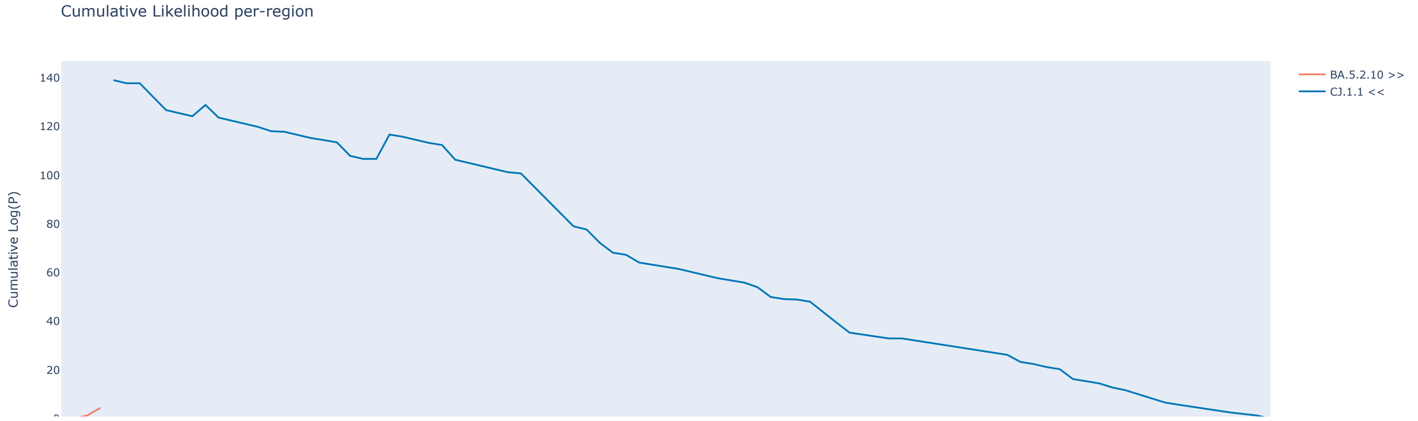

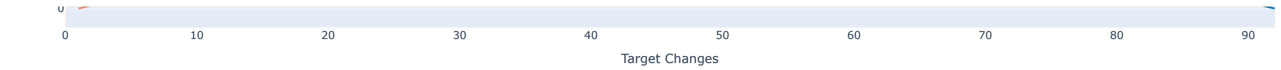


Cumulative Likelihood whole genome

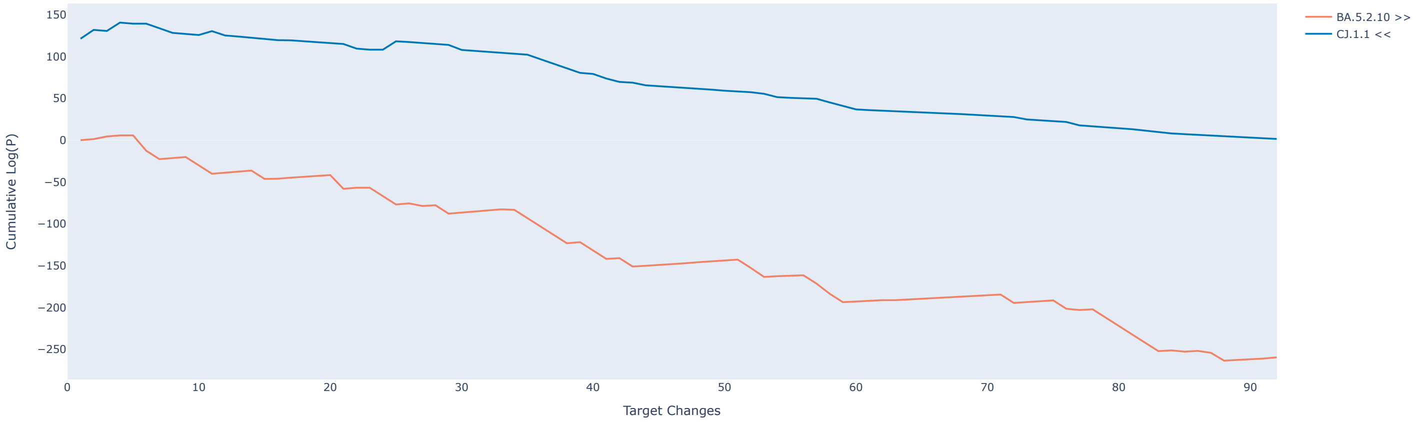


**Target sequence**

, 241_C|T, 670_T|G, 1627_C|T, 2790_C|T, 3037_C|T, 3796_C|T, 3927_C|T, 4184_G|A, 4321_C|T, 4586_C|T, 5183_C|T, 9344_C|T, 9424_A|G, 9534_C|T, 9866_C|T, 10029_C|T, 10198_C|T, 10447_G|A, 10449_C|A, 11288_11296, 12444_A|G, 12880_C|T, 14408_C|T, 14829_G|T, 15451_G|A, 15714_C|T, 17410_C|T, 18163_A|G, 18583_G|A, 19955_C|T, 20055_A|G, 21618_C|T, 21633_21641, 21987_G|A, 22001_A|G, 22016_T|C, 22033_C|A, 22190_A|G, 22200_T|G, 22331_G|A, 22577_G|C, 22578_G|A, 22599_G|C, 22674_C|T, 22679_T|C, 22686_C|T, 22688_A|G, 22775_G|A, 22786_A|C, 22813_G|T, 22882_T|G, 22898_G|A, 22942_T|G, 22992_G|A, 22995_C|A, 23013_A|C, 23018_T|C, 23019_T|C, 23031_T|C, 23055_A|G, 23063_A|T, 23075_T|C, 23403_A|G, 23525_C|T, 23599_T|G, 23604_C|A, 23854_C|A, 23948_G|T, 24424_A|T, 24469_T|A, 25000_C|T, 25416_C|T, 25584_C|T, 26060_C|T, 26270_C|T, 26275_A|G, 26577_C|G, 26709_G|A, 26858_C|T, 27259_A|C, 27382_G|C, 27383_A|T, 27384_T|C, 27807_C|T, 28271_A|T, 28311_C|T, 28362_28370, 28881_G|A, 28882_G|A, 28883_G|C, 29510_A|C, 29734_29759

## Case 59 (undefined): XBS test: KO

Target: (75%) 15 samples
Number of changes: 88
GT: BA.2.75 + BQ.1
GT BR: –
GT BR coord: –
Rank_L1_L2: 11 11 –
BC: BQ.1.1 + BN.1 + BQ.1.1
BC BR: 5–6, 29–30
BC BR coord: 2844 – 2845, 17409 – 17410
Direction L1: <<
Initial region span: 1–3,6–29,30–88
Gap history (edge excluded): 3–30 -> 3–6
Alt. candidates: [BN.1.3], []
Model 1BP/2BP comparison: 2BP vs 1BP: 2.17e–03
Rec. model vs L1: 3.05e–68
Rec. model vs L2: 2.17e–109
Flags: Model_2BP_Best

## BQ.1.1 >>

| | num_seq | t_ch_MAX | max_CL | CL@BC_t_ch_MAX | aic | PV | PV_OK | t_ch_MAX_OK | phyl_OK |
|---|---|---|---|---|---|---|---|---|---|
| **BQ.1.1** | 26162 | 3 | 1.568491 | None | None | None | * | * | * |

## BN.1 <<

| | num_seq | t_ch_MAX | max_CL | CL@BC_t_ch_MAX | aic | PV | PV_OK | t_ch_MAX_OK | phyl_OK |
|---|---|---|---|---|---|---|---|---|---|
| **BN.1** | 942 | 6 | 27.714376 | NaN | NaN | NaN | * | * | * |
| **BN.1.3** | 919 | 6 | 18.987036 | 18.987036 | 14.025928 | 1.616625e-04 | * | * | * |
| **BM.1.1.1** | 15 | 9 | 17.649785 | 13.131943 | 25.736114 | 4.632495e-07 | | | |
| **CH.1.1.23** | 10 | 9 | 17.649785 | 6.686334 | 38.627332 | 7.358462e-10 | | | |
| **CH.1.1.17** | 13 | 9 | 17.649785 | 6.686334 | 38.627332 | 7.358462e-10 | | | |
| **BN.1.1** | 73 | 9 | 17.649785 | 13.131943 | 25.736114 | 4.632495e-07 | | | * |
| **BM.1.1** | 104 | 9 | 17.649785 | 13.131943 | 25.736114 | 4.632495e-07 | | | |
| **BN.1.10** | 43 | 9 | 17.649785 | 13.131943 | 25.736114 | 4.632495e-07 | | | * |
| **CH.1.1.3** | 35 | 9 | 17.649785 | 6.686334 | 38.627332 | 7.358462e-10 | | | |
| **BN.2.1** | 19 | 9 | 17.649785 | 13.131943 | 25.736114 | 4.632495e-07 | | | |

## BQ.1.1 <<

| | num_seq | t_ch_MAX | max_CL | CL@BC_t_ch_MAX | aic | PV | PV_OK | t_ch_MAX_OK | phyl_OK |
|---|---|---|---|---|---|---|---|---|---|
| **BQ.1.1** | 26162 | 30 | 61.189472 | NaN | NaN | NaN | * | * | * |
| **BQ.1.1.18** | 1461 | 35 | 60.667713 | 54.805865 | 30.388271 | 5.247518e-03 | * | | * |
| **BQ.1.1.37** | 13 | 41 | 60.197218 | 5.870436 | 128.259128 | 2.935983e-24 | | | * |
| **ET.1** | 40 | 41 | 60.197218 | 10.854043 | 118.291913 | 4.292512e-22 | | | * |
| **BQ.1.1.24** | 271 | 41 | 60.174954 | 3.822101 | 132.355798 | 3.779635e-25 | | | * |
| **DT.3** | 38 | 41 | 60.170550 | -10.164409 | 160.328817 | 3.190373e-31 | | | * |
| **BQ.1.1.6** | 459 | 41 | 60.159905 | 24.915087 | 90.169826 | 5.481405e-16 | | | * |
| **BQ.1.1.72** | 50 | 41 | 60.156396 | 3.760952 | 132.478096 | 3.559526e-25 | | | * |
| **BQ.1.1.22** | 267 | 41 | 60.155628 | 3.716749 | 132.566501 | 3.402898e-25 | | | * |
| **BQ.1.1.52** | 404 | 41 | 60.134670 | 16.160610 | 107.678780 | 8.642586e-20 | | | * |

Cumulative Likelihood per-region

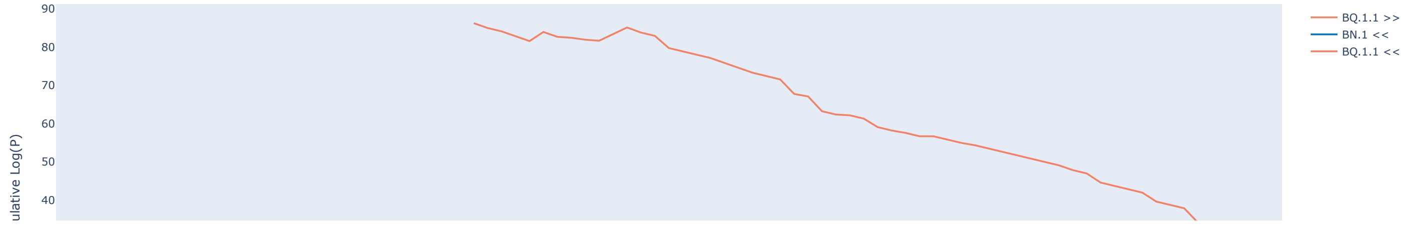

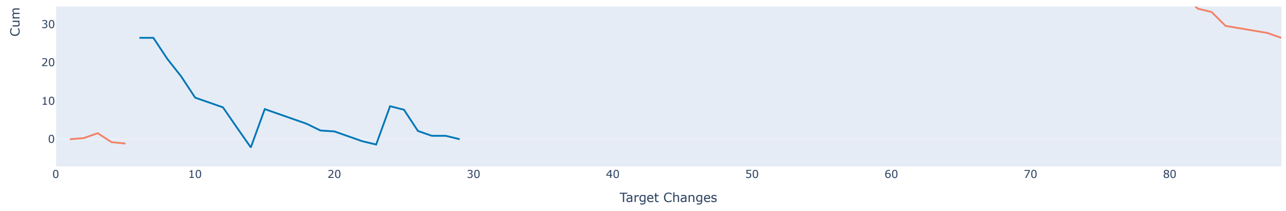


Cumulative Likelihood whole genome

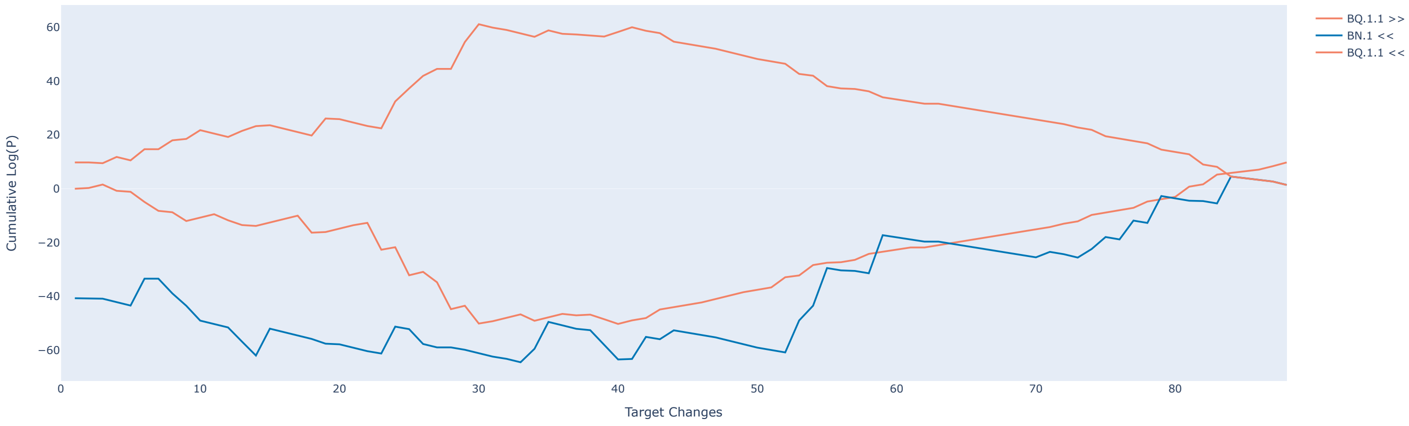


**Target sequence**

, 241_C|T, 510_518, 670_T|G, 2790_C|T, 2842_C|T, 3037_C|T, 3796_C|T, 3809_C|T, 3927_C|T, 4184_G|A, 4321_C|T, 4586_C|T, 5183_C|T, 6982_C|T, 9344_C|T, 9424_A|G, 9534_C|T, 9866_C|T, 10029_C|T, 10198_C|T, 10447_G|A, 10449_C|A, 10744_T|C, 11288_11296, 12444_A|G, 12880_C|T, 14408_C|T, 15451_G|A, 15714_C|T, 17410_C|T, 18163_A|G, 19955_C|T, 20055_A|G, 20748_T|C, 21618_C|T, 21633_21641, 21987_G|A, 22001_A|G, 22016_T|C, 22033_C|A, 22200_T|G, 22578_G|A, 22599_G|C, 22674_C|T, 22679_T|C, 22686_C|T, 22688_A|G, 22775_G|A, 22786_A|C, 22813_G|T, 22882_T|G, 22893_A|C, 22917_T|G, 22942_T|A, 22992_G|A, 22995_C|A, 23013_A|C, 23018_T|G, 23055_A|G, 23063_A|T, 23075_T|C, 23403_A|G, 23525_C|T, 23599_T|G, 23604_C|A, 23854_C|A, 23948_G|T, 24424_A|T, 24469_T|A, 25000_C|T, 25584_C|T, 26060_C|T, 26270_C|T, 26529_G|A, 26577_C|G, 26709_G|A, 27807_C|T, 27889_C|T, 28271_A|T, 28311_C|T, 28312_C|T, 28362_28370, 28681_G|T, 28881_G|A, 28882_G|A, 28883_G|C, 29510_A|C, 29734_29759

## Case 60 (undefined): XBV test: OK

Target: (75%) 5 samples
Number of changes: 94
GT: CR.1 + XBB.1
GT BR: –
GT BR coord: –
Rank_L1_L2: 1 2
BC: CR.1 + XBB.1.5
BC BR: 25–26
BC BR coord: 19336 – 19337
Direction L1: <<
Initial region span: 1–25,26–94
Gap history (edge excluded):
Alt. candidates: [], []
Model 1BP/2BP comparison: 1BP vs 2BP: 3.48e–09
Rec. model vs L1: 1.24e–224
Rec. model vs L2: 2.17e–100
Flags: Model_1BP_Best

## CR.1 >>

| | num_seq | t_ch_MAX | max_CL | CL@BC_t_ch_MAX | aic | PV | PV_OK | t_ch_MAX_OK | phyl_OK |
|---|---|---|---|---|---|---|---|---|---|
| **CR.1** | 77 | 25 | 45.434917 | NaN | NaN | NaN | * | * | * |
| **BA.5.2** | 32711 | 25 | 31.688804 | 31.688804 | 0.622392 | 1.073056e-06 | | * | * |
| **BA.5.2.24** | 84 | 21 | 30.460688 | 26.941050 | 10.117899 | 9.283753e-09 | | | |
| **CR.1.1** | 341 | 23 | 24.593325 | 19.941515 | 24.116971 | 8.465687e-12 | | | * |
| **BA.5.2.27** | 545 | 21 | 22.190162 | 18.678828 | 26.642345 | 2.401326e-12 | | | |
| **BA.5.2.6** | 2139 | 25 | 22.004948 | 22.004948 | 19.990105 | 6.675446e-11 | | * | |
| **BA.5.2.21** | 2744 | 25 | 21.847656 | 21.847656 | 20.304687 | 5.716953e-11 | | * | |
| **BA.5.2.9** | 5582 | 20 | 20.521305 | -2.246854 | 68.493709 | 1.962633e-21 | | | |
| **BF.3.1** | 271 | 17 | 18.235518 | -7.087953 | 78.175906 | 1.544125e-23 | | | |
| **BA.5.2.1** | 58578 | 17 | 17.956795 | 10.683543 | 42.632913 | 8.095930e-16 | | | * |

## XBB.1.5 <<

| | num_seq | t_ch_MAX | max_CL | CL@BC_t_ch_MAX | aic | PV | PV_OK | t_ch_MAX_OK | phyl_OK |
|---|---|---|---|---|---|---|---|---|---|
| **XBB.1.5** | 35885 | 26 | 113.313657 | NaN | NaN | NaN | * | * | * |
| **XBB.1** | 1674 | 26 | 100.265828 | 100.265828 | -52.531656 | 2.150092e-06 | | * | * |
| **XBB.1.15** | 956 | 26 | 97.892409 | 97.892409 | -47.784819 | 1.999897e-07 | | * | |
| **FD.2** | 577 | 26 | 86.746735 | 86.746735 | -25.493469 | 2.889320e-12 | | * | * |
| **XBB.1.32** | 18 | 26 | 85.859561 | 85.859561 | -23.719121 | 1.192463e-12 | | * | |
| **XBB.1.1** | 69 | 26 | 85.650063 | 85.650063 | -23.300126 | 9.665919e-13 | | * | |
| **XBB.1.9.2** | 134 | 26 | 84.794240 | 84.794240 | -21.588479 | 4.110753e-13 | | * | |
| **XBB.1.30** | 25 | 28 | 82.675803 | 80.934393 | -13.868787 | 8.660534e-15 | | | |
| **XBB.1.5.39** | 78 | 26 | 81.453082 | 81.453082 | -14.906164 | 1.456726e-14 | | * | * |
| **XBB.1.5.23** | 38 | 26 | 81.411918 | 81.411918 | -14.823837 | 1.392626e-14 | | * | * |

Cumulative Likelihood per-region

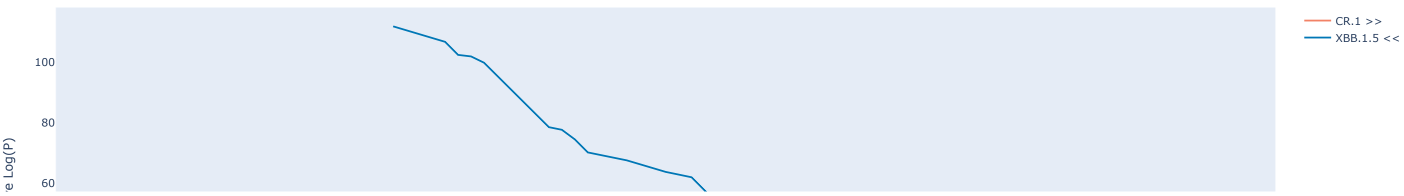

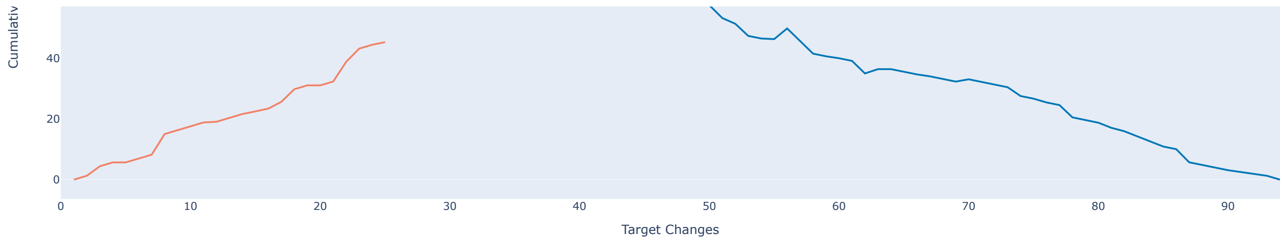


Cumulative Likelihood whole genome

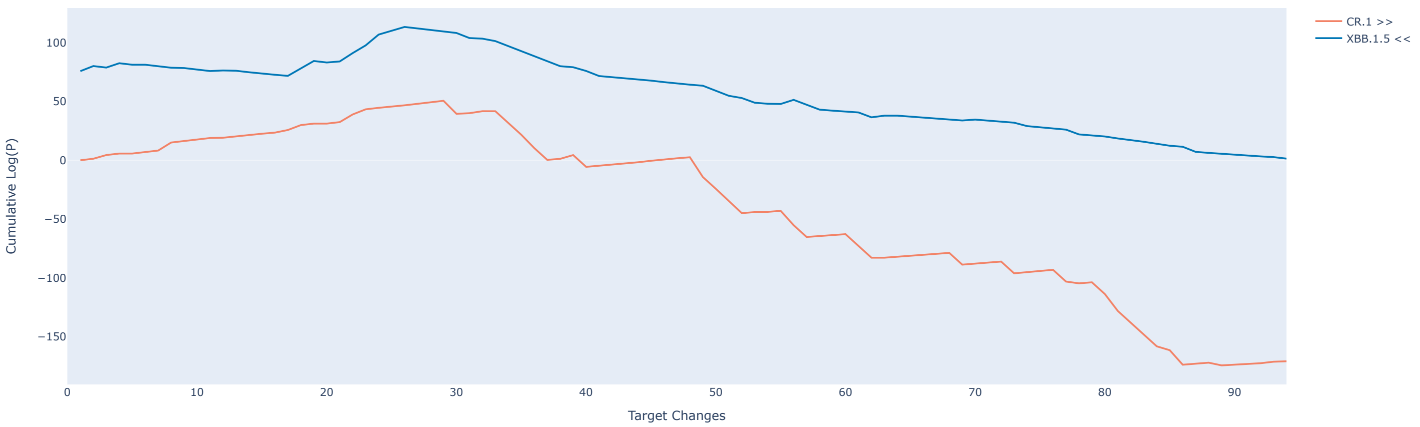


**Target sequence**

, 241_C|T, 670_T|G, 1627_C|T, 2790_C|T, 3037_C|T, 4184_G|A, 4321_C|T, 8040_A|G, 9344_C|T, 9424_A|G, 9534_C|T, 10029_C|T, 10198_C|T, 10447_G|A, 10449_C|A, 11288_11296, 12160_G|A, 12310_G|A, 12880_C|T, 14408_C|T, 15714_C|T, 15957_G|T, 16616_C|A, 17410_C|T, 18163_A|G, 19955_C|T, 20055_A|G, 21618_C|T, 21633_21641, 21810_T|C, 21987_G|A, 21992_21994, 22000_C|A, 22109_C|G, 22200_T|A, 22317_G|T, 22577_G|C, 22578_G|A, 22599_G|C, 22664_C|A, 22674_C|T, 22679_T|C, 22686_C|T, 22688_A|G, 22775_G|A, 22786_A|C, 22813_G|T, 22882_T|G, 22895_G|C, 22896_T|C, 22898_G|A, 22942_T|G, 22992_G|A, 22995_C|A, 23013_A|C, 23019_T|C, 23031_T|C, 23055_A|G, 23063_A|T, 23075_T|C, 23123_C|A, 23223_A|G, 23403_A|G, 23525_C|T, 23599_T|G, 23604_C|A, 23854_C|A, 23948_G|T, 24170_A|G, 24424_A|T, 24469_T|A, 25000_C|T, 25416_C|T, 25584_C|T, 26060_C|T, 26270_C|T, 26275_A|G, 26577_C|G, 26709_G|A, 26858_C|T, 27259_A|C, 27382_G|C, 27383_A|T, 27384_T|C, 27807_C|T, 27915_G|T, 28271_A|T, 28311_C|T, 28362_28370, 28881_G|A, 28882_G|A, 28883_G|C, 29510_A|C, 29734_29759

## Case 61 (undefined): XCA        test: OK

Target: (75%) 11 samples
Number of changes: 97
GT: BA.2.75* + BQ.1*
GT BR: –
GT BR coord: –
Rank L1 L2: 11 1
BC: BA.2.75.2 + BQ.1
BC BR: 58–59
BC BR coord: 22892 – 22893
Direction L1: >>
Initial region span: 1–58,59–97
Gap history (edge excluded):
Alt. candidates: [], [BQ.1.1.18, BQ.1.1, BQ.1.1.1]
Model 1BP/2BP comparison: –
Rec. model vs L1: 2.33e–132
Rec. model vs L2: 2.12e–126
Flags: Model_2BP_Bad_L1_opp

### BA.2.75.2 >>

| | num_seq | t_ch_MAX | max_CL | CL@BC_t_ch_MAX | aic | PV | PV_OK | t_ch_MAX_OK | phyl_OK |
|---|---|---|---|---|---|---|---|---|---|
| **BA.2.75.2** | 582 | 58 | 75.853189 | NaN | NaN | NaN | * | * | * |
| **CH.1.1** | 2870 | 53 | 59.059058 | 53.387475 | 31.225050 | 1.743424e-10 | | | |
| **BN.1.5** | 730 | 12 | 29.599589 | -5.792831 | 149.585662 | 3.466215e-36 | | | |
| **BY.1** | 153 | 11 | 28.876496 | 10.583537 | 116.832926 | 4.504008e-29 | | | |
| **BN.1** | 942 | 11 | 26.052067 | 5.226866 | 127.546267 | 2.117292e-31 | | | |
| **CA.3** | 34 | 12 | 22.894152 | 14.488579 | 109.022842 | 2.236244e-27 | | | * |
| **CA.3.1** | 148 | 12 | 22.866894 | 4.822633 | 128.354735 | 1.419263e-31 | | | * |
| **CH.1.1.14** | 55 | 11 | 22.454570 | -0.978597 | 139.957194 | 4.275466e-34 | | | |
| **BN.1.3** | 919 | 11 | 20.011188 | -9.172193 | 156.344386 | 1.186074e-37 | | | |
| **BM.1.1** | 104 | 53 | 19.956603 | 14.258733 | 109.482535 | 1.776771e-27 | | | |

### BQ.1 <<

| | num_seq | t_ch_MAX | max_CL | CL@BC_t_ch_MAX | aic | PV | PV_OK | t_ch_MAX_OK | phyl_OK |
|---|---|---|---|---|---|---|---|---|---|
| **BQ.1** | 11861 | 59 | 47.835439 | NaN | NaN | NaN | * | * | * |
| **BQ.1.1.18** | 1461 | 59 | 46.698576 | 46.698576 | -11.397153 | 3.214221e-01 | * | * | * |
| **BQ.1.1** | 26162 | 59 | 43.937431 | 43.937431 | -5.874861 | 2.024191e-02 | * | * | * |
| **BQ.1.1.1** | 723 | 59 | 37.199353 | 37.199353 | 7.601295 | 2.405903e-05 | * | * | * |
| **BQ.1.13** | 914 | 59 | 35.134866 | 35.134866 | 11.730267 | 3.051126e-06 | | * | * |
| **BQ.1.23** | 776 | 59 | 34.770519 | 34.770519 | 12.458962 | 2.118081e-06 | | * | * |
| **BE.1** | 5712 | 62 | 28.062647 | 8.727184 | 64.545633 | 1.034529e-17 | | | * |
| **BQ.1.1.31** | 163 | 59 | 25.720224 | 25.720224 | 30.559551 | 2.486437e-10 | | * | * |
| **BQ.1.1.63** | 200 | 59 | 25.564766 | 25.564766 | 30.870467 | 2.129423e-10 | | * | * |
| **BQ.1.2** | 2858 | 59 | 24.950014 | 24.950014 | 32.099972 | 1.151253e-10 | | * | * |

Cumulative Likelihood per-region

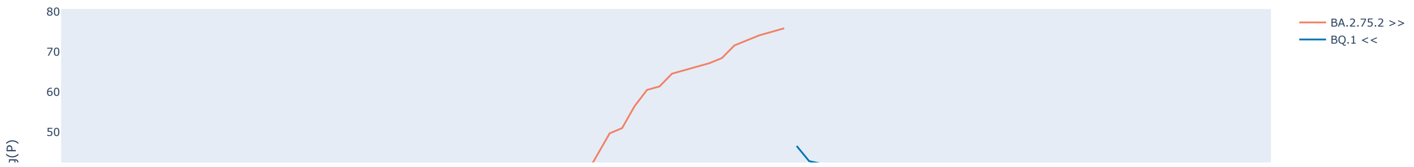

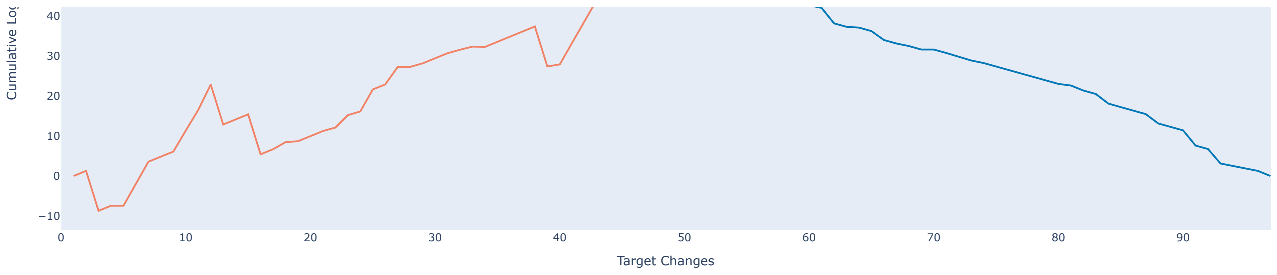


Cumulative Likelihood whole genome

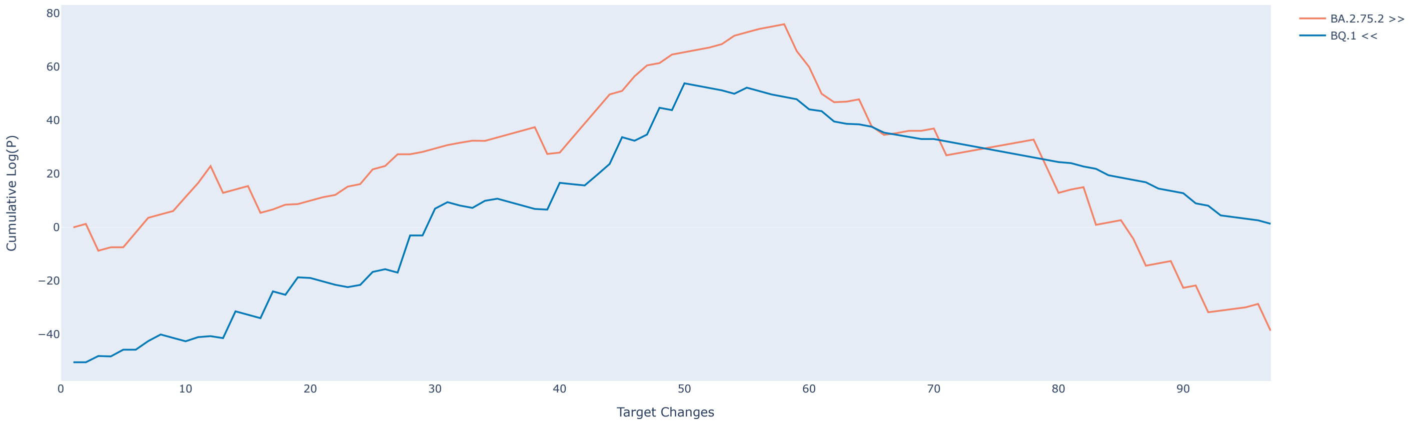


**Target sequence**

, 241_C|T, 670_T|G, 2062_C|T, 2790_C|T, 3037_C|T, 3796_C|T, 3927_C|T, 4184_G|A, 4321_C|T, 4586_C|T, 5183_C|T, 5192_C|T, 5748_C|A, 9344_C|T, 9424_A|G, 9454_T|C, 9534_C|T, 9866_C|T, 10029_C|T, 10198_C|T, 10447_G|A, 10449_C|A, 11124_C|T, 11288_11296, 12444_A|G, 12880_C|T, 13324_T|C, 14408_C|T, 15451_G|A, 15714_C|T, 17410_C|T, 18163_A|G, 18583_G|A, 19017_C|T, 19955_C|T, 20055_A|G, 21618_C|T, 21633_21641, 21893_G|A, 21987_G|A, 22001_A|G, 22016_T|C, 22033_C|A, 22190_A|G, 22200_T|G, 22331_G|A, 22577_G|C, 22578_G|A, 22599_G|C, 22674_C|T, 22679_T|C, 22686_C|T, 22688_A|G, 22747_C|T, 22775_G|A, 22786_A|C, 22813_G|T, 22882_T|G, 22893_A|C, 22917_T|G, 22942_T|A, 22992_G|A, 22995_C|A, 23013_A|C, 23018_T|G, 23055_A|G, 23063_A|T, 23075_T|C, 23403_A|G, 23525_C|T, 23595_C|T, 23599_T|G, 23604_C|A, 23854_C|A, 23948_G|T, 24424_A|T, 24469_T|A, 25000_C|T, 25584_C|T, 25975_T|C, 26060_C|T, 26270_C|T, 26529_G|A, 26577_C|G, 26709_G|A, 27807_C|T, 27889_C|T, 28271_A|T, 28311_C|T, 28312_C|T, 28362_28370, 28681_G|T, 28881_G|A, 28882_G|A, 28883_G|C, 29510_A|C, 29728_29776

**Supplementary Notes 4**

RecombinHunt output when run on the Nextstrain mpox genome sequences. One result is provided for each single genome sequence (as listed in Table 5c).

# Case 1: ON838178.1

Target: 138 samples      Number of changes: 61
GT: B.1.3
BC: B.1.3 + B.1.2 + B.1.3  BC BR: 32-33, 56-57  BC BR coord: 85217 - 85218, 174729 - 174730
Direction L1: >>  Initial region span: [[0, 32], [32, 49], [56, 61]]  Gap history (edge excluded): 32-57 -> 49-57
Alt. candidates: [], []
Model 1BP/2BP comparison: 2BP vs 1BP: 8.76e-02
Rec. model vs L1: 9.91e-17  Rec. model vs L2: 1.84e-45
Flags: Model_2BP_Best

## B.1.3 >>

| | num_seq | t_ch_MAX | max_CL | CL@BC_t_ch_MAX | aic | PV | PV_OK | t_ch_MAX_OK | phyl_OK |
|---|---|---|---|---|---|---|---|---|---|
| **B.1.3** | 138 | 32 | 7.024316 | NaN | NaN | NaN | * | * | * |
| **B.1.6** | 14 | 19 | 0.828990 | -28.465654 | 160.931307 | 1.104007e-17 | | | * |
| **B.1.13** | 34 | 19 | 0.828990 | -28.574020 | 161.148039 | 9.890074e-18 | | | * |
| **B.1** | 1309 | 19 | 0.815200 | -6.609864 | 117.219728 | 3.423559e-08 | | | * |
| **B.1.2** | 189 | 16 | 0.680611 | -30.986247 | 165.972494 | 8.882800e-19 | | | * |
| **B.1.12** | 61 | 7 | 0.273744 | -32.297142 | 168.594284 | 2.396757e-19 | | | * |
| **B.1.8** | 74 | 6 | 0.232510 | -42.180161 | 188.360322 | 1.220740e-23 | | | * |
| **B.1.5** | 59 | 6 | 0.232510 | -39.344732 | 182.689463 | 2.078969e-22 | | | * |
| **B.1.1** | 183 | 6 | 0.232510 | -33.790153 | 171.580307 | 5.374695e-20 | | | * |
| **B.1.11** | 94 | 5 | 0.186725 | -35.840287 | 175.680575 | 6.919109e-21 | | | * |

## B.1.2 >>

| | num_seq | t_ch_MAX | max_CL | CL@BC_t_ch_MAX | aic | PV | PV_OK | t_ch_MAX_OK | phyl_OK |
|---|---|---|---|---|---|---|---|---|---|
| **B.1.2** | 189 | 49 | 1.092474 | NaN | NaN | NaN | * | * | * |
| **B.1** | 1309 | 56 | 0.777612 | 0.584322 | 116.831355 | 7.767901e+00 | * | | * |
| **B.1.14** | 22 | 43 | 0.448867 | -35.853363 | 189.706726 | 1.160413e-15 | | | * |
| **B.1.1** | 183 | 43 | 0.448867 | -2.027831 | 122.055662 | 5.683601e-01 | * | | * |
| **B.1.8** | 74 | 38 | 0.231112 | -8.096307 | 134.192614 | 1.320163e-03 | * | | * |
| **B.1.7** | 134 | 38 | 0.231112 | -5.475856 | 128.951712 | 1.813340e-02 | * | | * |
| **B.1.17** | 16 | 37 | 0.190290 | -28.808872 | 175.617743 | 1.331121e-12 | | | * |
| **B.1.5** | 59 | 37 | 0.190290 | -18.511097 | 155.022194 | 3.957770e-08 | | | * |
| **B.1.6** | 14 | 37 | 0.190290 | -6.093518 | 130.187037 | 9.754759e-03 | * | | * |
| **B.1.12** | 61 | 36 | 0.169076 | -17.069705 | 152.139410 | 1.670454e-07 | | | * |

## B.1.3 <<

| | num_seq | t_ch_MAX | max_CL | CL@BC_t_ch_MAX | aic | PV | PV_OK | t_ch_MAX_OK | phyl_OK |
|---|---|---|---|---|---|---|---|---|---|
| **B.1.3** | 138 | 57 | 3.559497 | None | None | None | * | * | * |

Cumulative Likelihood per-region

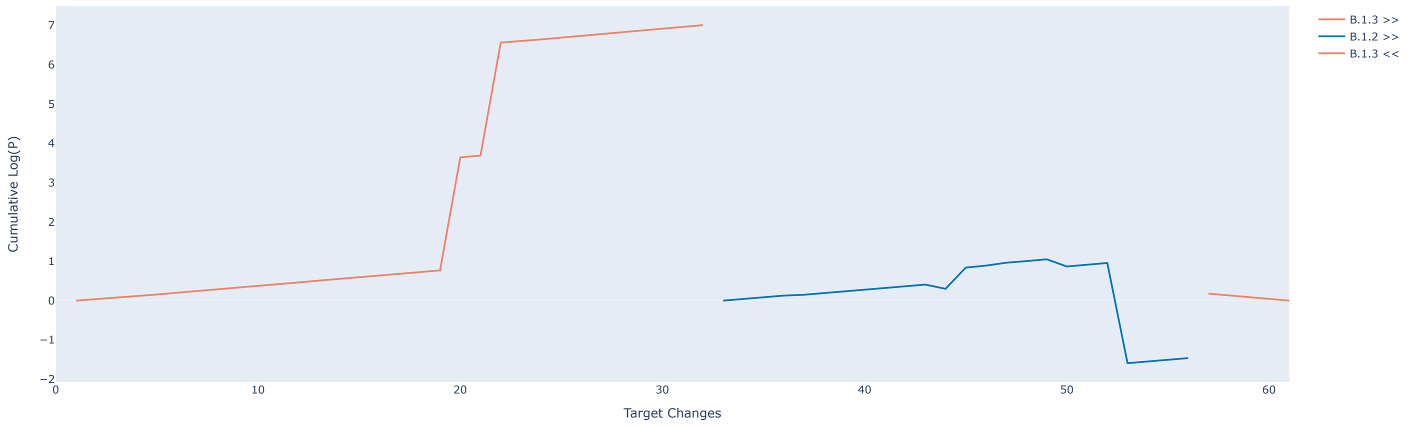


Cumulative Likelihood whole genome

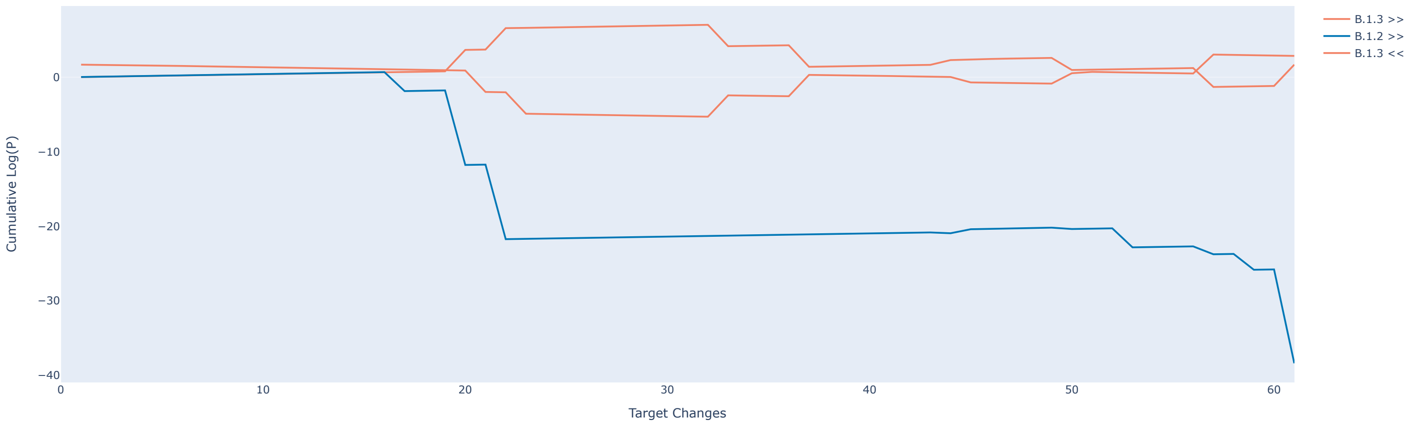


**Target sequence**

, 7927_A|G, 7934_C|T, 9945_A|G, 14163_G|T, 15591_G|A, 21886_G|A, 23727_C|T, 25824_G|A, 30530_G|A, 31216_G|A, 34622_G|A, 37365_G|A, 38523_G|A, 38825_C|T, 39282_C|T, 39302_C|T, 53048_G|A, 54280_G|A, 54798_G|A, 55296_G|A, 64460_G|A, 64589_C|T, 72525_C|T, 73229_C|T, 73402_G|A, 74368_G|A, 77546_G|A, 81438_G|A, 82536_C|T, 82614_G|A, 83489_C|T, 84750_C|T, 87393_G|A, 87460_G|A, 91891_G|A, 95197_G|A, 109899_A|G, 119459_C|T, 121483_C|T, 124293_G|A, 124837_G|A, 125412_G|A, 128861_C|T, 133266_TTTN|...., 133328_T|C, 133331_CAATCTTTCT|..........,

147062_....................|TATATTTTATTTTATATTT, 148563_G|A, 150620_C|T, 150736_GATATGATGGATATGAT|................., 151612_A|C, 155946_G|A, 162394_G|A, 162482_C|T, 164983_C|T, 168271_C|T, 182170_G|A, 183709_C|T, 186768_G|A, 187618_C|T, 190850_G|A

# Case 2: ON609725.2

Target: 138 samples  Number of changes: 62
GT: B.1.3
BC: B.1.3 + B.1.2 + B.1.3  BC BR: 32–33, 50–51  BC BR coord: 85217 – 85218, 150981 – 150982
Direction L1: >>  Initial region span: [[0, 32], [32, 49], [57, 62]]  Gap history (edge excluded): 32–58 -> 49–58
Alt. candidates: [], []
Model 1BP/2BP comparison: 2BP vs 1BP: 1.39e–01
Rec. model vs L1: 1.45e–16  Rec. model vs L2: 3.82e–52
Flags: Model_2BP_Best

## B.1.3 >>

| | num_seq | t_ch_MAX | max_CL | CL@BC_t_ch_MAX | aic | PV | PV_OK | t_ch_MAX_OK | phyl_OK |
|---|---|---|---|---|---|---|---|---|---|
| **B.1.3** | 138 | 32 | 7.024316 | NaN | NaN | NaN | * | * | * |
| **B.1.6** | 14 | 19 | 0.828990 | -38.271904 | 200.543807 | 8.558047e-22 | | | * |
| **B.1.13** | 34 | 19 | 0.828990 | -38.380269 | 200.760539 | 7.666591e-22 | | | * |
| **B.1** | 1309 | 19 | 0.815200 | -16.453608 | 156.907215 | 2.549816e-12 | | | * |
| **B.1.2** | 189 | 16 | 0.680611 | -40.792497 | 205.584994 | 6.885771e-23 | | | * |
| **B.1.12** | 61 | 7 | 0.273744 | -42.119921 | 208.239843 | 1.821130e-23 | | | * |
| **B.1.8** | 74 | 6 | 0.232510 | -52.340722 | 228.681445 | 6.635159e-28 | | | * |
| **B.1.5** | 59 | 6 | 0.232510 | -49.588355 | 223.176711 | 1.037913e-26 | | | * |
| **B.1.1** | 183 | 6 | 0.232510 | -44.363159 | 212.726319 | 1.929078e-24 | | | * |
| **B.1.11** | 94 | 5 | 0.186725 | -45.646537 | 215.293075 | 5.363557e-25 | | | * |

## B.1.2 >>

| | num_seq | t_ch_MAX | max_CL | CL@BC_t_ch_MAX | aic | PV | PV_OK | t_ch_MAX_OK | phyl_OK |
|---|---|---|---|---|---|---|---|---|---|
| **B.1.2** | 189 | 49 | 1.092474 | NaN | NaN | NaN | * | * | * |
| **B.1** | 1309 | 55 | 0.735965 | 0.512287 | 72.975425 | 6.703200e-01 | * | | * |
| **B.1.14** | 22 | 43 | 0.448867 | -15.373172 | 104.746343 | 8.462806e-08 | | | * |
| **B.1.1** | 183 | 43 | 0.448867 | -1.454825 | 76.909650 | 9.394930e-02 | * | | * |
| **B.1.8** | 74 | 38 | 0.231112 | -4.560554 | 83.121107 | 4.211232e-03 | * | | * |
| **B.1.7** | 134 | 38 | 0.231112 | -4.862880 | 83.725760 | 3.104198e-03 | * | | * |
| **B.1.17** | 16 | 37 | 0.190290 | -14.928170 | 103.856340 | 1.320613e-07 | | | * |
| **B.1.5** | 59 | 37 | 0.190290 | -11.336694 | 96.673388 | 4.809103e-06 | | | * |
| **B.1.6** | 14 | 37 | 0.190290 | -6.287269 | 86.574537 | 7.503281e-04 | * | | * |
| **B.1.12** | 61 | 36 | 0.169076 | -13.523803 | 101.047607 | 5.382192e-07 | | | * |

## B.1.3 <<

| | num_seq | t_ch_MAX | max_CL | CL@BC_t_ch_MAX | aic | PV | PV_OK | t_ch_MAX_OK | phyl_OK |
|---|---|---|---|---|---|---|---|---|---|
| **B.1.3** | 138 | 58 | 3.559497 | None | None | None | * | * | * |

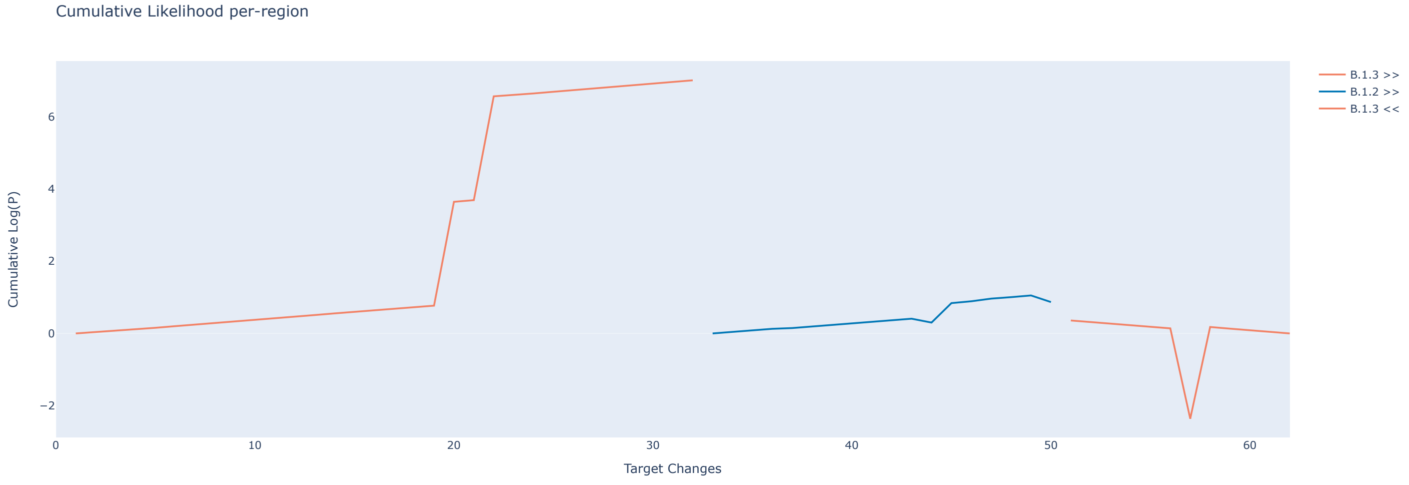


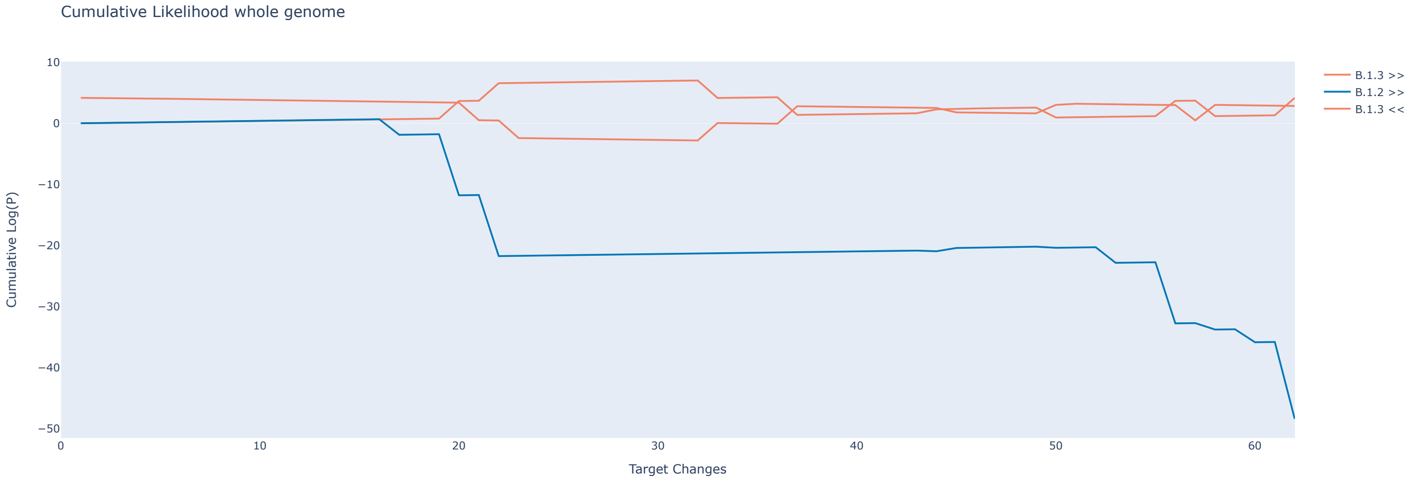


**Target sequence**

, 7927_A|G, 7934_C|T, 9945_A|G, 14163_G|T, 15591_G|A, 21886_G|A, 23727_C|T, 25824_G|A, 30530_G|A, 31216_G|A, 34622_G|A, 37365_G|A, 38523_G|A, 38825_C|T, 39282_C|T, 39302_C|T, 53048_G|A, 54280_G|A, 54798_G|A, 55296_G|A, 64460_G|A, 64589_C|T, 72525_C|T, 73229_C|T, 73402_G|A, 74368_G|A, 77546_G|A, 81438_G|A, 82536_C|T, 82614_G|A, 83489_C|T, 84750_C|T, 87393_G|A, 87460_G|A,

91891_G|A, 95197_G|A, 109899_A|G, 119459_C|T, 121483_C|T, 124293_G|A, 124837_G|A, 125412_G|A, 128861_C|T, 133266_TTTN|...., 133328_T|C, 133331_CAATCTTTCT|.........., 147062_...................|TATATTTTATTTTATATTT, 148563_G|A, 150620_C|T, 150736_GATATGATGGATATGAT|................., 151612_A|C, 155946_G|A, 162394_G|A, 162482_C|T, 164983_C|T, 167668_G|A, 168271_C|T, 182170_G|A, 183709_C|T, 186768_G|A, 187618_C|T, 190850_G|A

## Case 3: ON754985.1

```
Target: 138 samples       Number of changes: 61
GT: B.1.3
BC: B.1.3 + B.1.2 + B.1.3  BC BR: 32-33, 56-57                         BC BR coord: 85217 - 85218, 174729 - 174730
Direction L1: >>          Initial region span: [[0, 32], [32, 49], [56, 61]] Gap history (edge excluded): 32-57 -> 49-57
Alt. candidates: [], []
Model 1BP/2BP comparison:  2BP vs 1BP: 8.76e-02
Rec. model vs L1: 9.91e-17 Rec. model vs L2: 1.84e-45
Flags: Model_2BP_Best
```

### B.1.3 >>

| | num_seq | t_ch_MAX | max_CL | CL@BC_t_ch_MAX | aic | PV | PV_OK | t_ch_MAX_OK | phyl_OK |
|---|---|---|---|---|---|---|---|---|---|
| **B.1.3** | 138 | 32 | 7.024316 | NaN | NaN | NaN | * | * | * |
| **B.1.6** | 14 | 19 | 0.828990 | -28.465654 | 160.931307 | 1.104007e-17 | | | * |
| **B.1.13** | 34 | 19 | 0.828990 | -28.574020 | 161.148039 | 9.890074e-18 | | | * |
| **B.1** | 1309 | 19 | 0.815200 | -6.609864 | 117.219728 | 3.423559e-08 | | | * |
| **B.1.2** | 189 | 16 | 0.680611 | -30.986247 | 165.972494 | 8.882800e-19 | | | * |
| **B.1.12** | 61 | 7 | 0.273744 | -32.297142 | 168.594284 | 2.396757e-19 | | | * |
| **B.1.8** | 74 | 6 | 0.232510 | -42.180161 | 188.360322 | 1.220740e-23 | | | * |
| **B.1.5** | 59 | 6 | 0.232510 | -39.344732 | 182.689463 | 2.078969e-22 | | | * |
| **B.1.1** | 183 | 6 | 0.232510 | -33.790153 | 171.580307 | 5.374695e-20 | | | * |
| **B.1.11** | 94 | 5 | 0.186725 | -35.840287 | 175.680575 | 6.919109e-21 | | | * |

### B.1.2 >>

| | num_seq | t_ch_MAX | max_CL | CL@BC_t_ch_MAX | aic | PV | PV_OK | t_ch_MAX_OK | phyl_OK |
|---|---|---|---|---|---|---|---|---|---|
| **B.1.2** | 189 | 49 | 1.092474 | NaN | NaN | NaN | * | * | * |
| **B.1** | 1309 | 56 | 0.777612 | 0.584322 | 116.831355 | 7.767901e+00 | * | | * |
| **B.1.14** | 22 | 43 | 0.448867 | -35.853363 | 189.706726 | 1.160413e-15 | | | * |
| **B.1.1** | 183 | 43 | 0.448867 | -2.027831 | 122.055662 | 5.683601e-01 | * | | * |
| **B.1.8** | 74 | 38 | 0.231112 | -8.096307 | 134.192614 | 1.320163e-03 | * | | * |
| **B.1.7** | 134 | 38 | 0.231112 | -5.475856 | 128.951712 | 1.813340e-02 | * | | * |
| **B.1.17** | 16 | 37 | 0.190290 | -28.808872 | 175.617743 | 1.331121e-12 | | | * |
| **B.1.5** | 59 | 37 | 0.190290 | -18.511097 | 155.022194 | 3.957770e-08 | | | * |
| **B.1.6** | 14 | 37 | 0.190290 | -6.093518 | 130.187037 | 9.754759e-03 | * | | * |
| **B.1.12** | 61 | 36 | 0.169076 | -17.069705 | 152.139410 | 1.670454e-07 | | | * |

### B.1.3 <<

| | num_seq | t_ch_MAX | max_CL | CL@BC_t_ch_MAX | aic | PV | PV_OK | t_ch_MAX_OK | phyl_OK |
|---|---|---|---|---|---|---|---|---|---|
| **B.1.3** | 138 | 57 | 3.559497 | None | None | None | * | * | * |

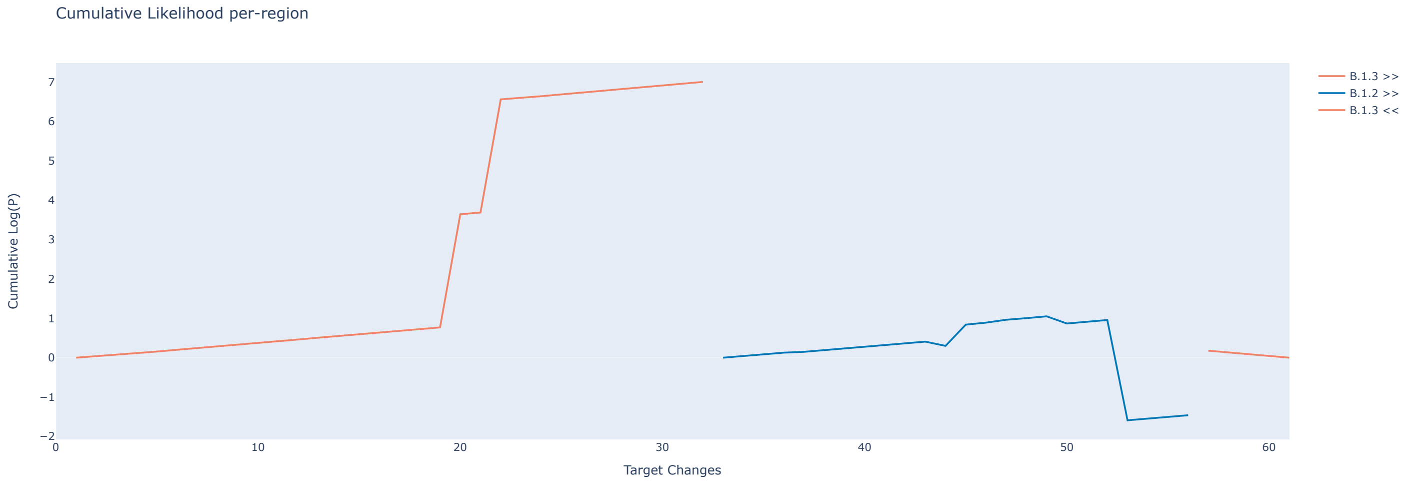


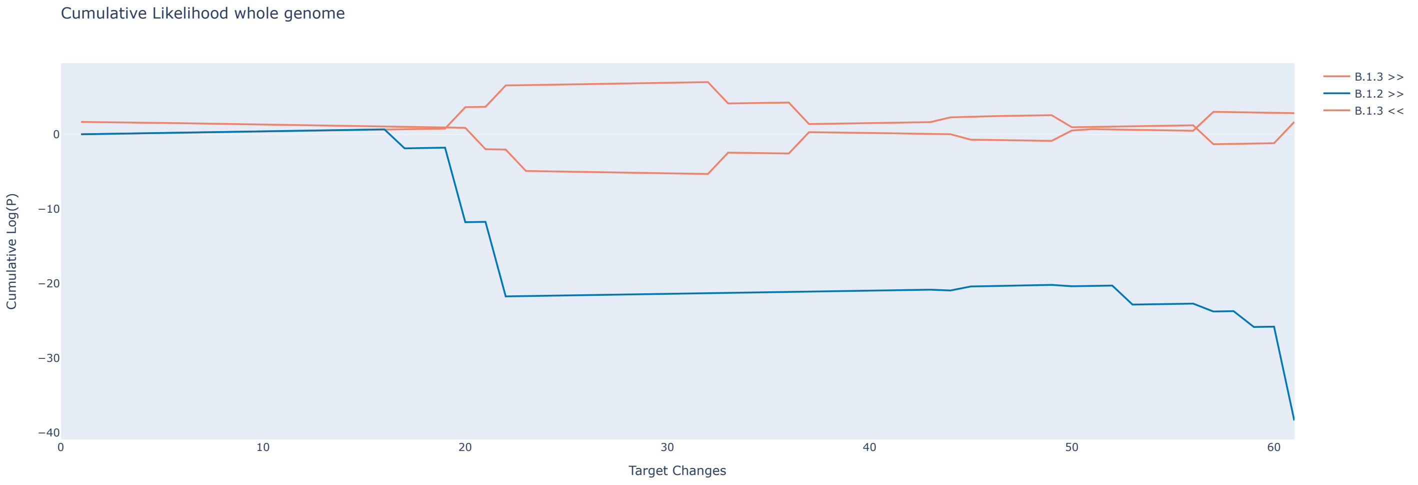


**Target sequence**

, 7927_A|G, 7934_C|T, 9945_A|G, 14163_G|T, 15591_G|A, 21886_G|A, 23727_C|T, 25824_G|A, 30530_G|A, 31216_G|A, 34622_G|A, 37365_G|A, 38523_G|A, 38825_C|T, 39282_C|T, 39302_C|T, 53048_G|A,

54280_G|A, 54798_G|A, 55296_G|A, 64460_G|A, 64589_C|T, 72525_C|T, 73229_C|T, 73402_G|A, 74368_G|A, 77546_G|A, 81438_G|A, 82536_C|T, 82614_G|A, 83489_C|T, 84750_C|T, 87393_G|A, 87460_G|A, 91891_G|A, 95197_G|A, 109899_A|G, 119459_C|T, 121483_C|T, 124293_G|A, 124837_G|A, 125412_G|A, 128861_C|T, 133266_TTTN|...., 133328_T|C, 133331_CAATCTTTCT|.........., 147062_...................|TATATTTTATTTTATATTT, 148563_G|A, 150620_C|T, 150736_GATATGATGGATATGAT|................., 151612_A|C, 155946_G|A, 162394_G|A, 162482_C|T, 164983_C|T, 168271_C|T, 182170_G|A, 183709_C|T, 186768_G|A, 187618_C|T, 190850_G|A

## Case 4: ON754986.1

Target: 138 samples    Number of changes: 61
GT: B.1.3
BC: B.1.3 + B.1.2 + B.1.3    BC BR: 32–33, 56–57    BC BR coord: 85217 – 85218, 174729 – 174730
Direction L1: >>    Initial region span: [[0, 32], [32, 49], [56, 61]]    Gap history (edge excluded): 32-57 -> 49-57
Alt. candidates: [], []
Model 1BP/2BP comparison: 2BP vs 1BP: 8.76e−02
Rec. model vs L1: 9.91e−17    Rec. model vs L2: 1.84e−45
Flags: Model_2BP_Best

### B.1.3 >>

| | num_seq | t_ch_MAX | max_CL | CL@BC_t_ch_MAX | aic | PV | PV_OK | t_ch_MAX_OK | phyl_OK |
|---|---|---|---|---|---|---|---|---|---|
| **B.1.3** | 138 | 32 | 7.024316 | NaN | NaN | NaN | * | * | * |
| **B.1.6** | 14 | 19 | 0.828990 | -28.465654 | 160.931307 | 1.104007e-17 | | | * |
| **B.1.13** | 34 | 19 | 0.828990 | -28.574020 | 161.148039 | 9.890074e-18 | | | * |
| **B.1** | 1309 | 19 | 0.815200 | -6.609864 | 117.219728 | 3.423559e-08 | | | * |
| **B.1.2** | 189 | 16 | 0.680611 | -30.986247 | 165.972494 | 8.882800e-19 | | | * |
| **B.1.12** | 61 | 7 | 0.273744 | -32.297142 | 168.594284 | 2.396757e-19 | | | * |
| **B.1.8** | 74 | 6 | 0.232510 | -42.180161 | 188.360322 | 1.220740e-23 | | | * |
| **B.1.5** | 59 | 6 | 0.232510 | -39.344732 | 182.689463 | 2.078969e-22 | | | * |
| **B.1.1** | 183 | 6 | 0.232510 | -33.790153 | 171.580307 | 5.374695e-20 | | | * |
| **B.1.11** | 94 | 5 | 0.186725 | -35.840287 | 175.680575 | 6.919109e-21 | | | * |

### B.1.2 >>

| | num_seq | t_ch_MAX | max_CL | CL@BC_t_ch_MAX | aic | PV | PV_OK | t_ch_MAX_OK | phyl_OK |
|---|---|---|---|---|---|---|---|---|---|
| **B.1.2** | 189 | 49 | 1.092474 | NaN | NaN | NaN | * | * | * |
| **B.1** | 1309 | 56 | 0.777612 | 0.584322 | 116.831355 | 7.767901e+00 | * | | * |
| **B.1.14** | 22 | 43 | 0.448867 | -35.853363 | 189.706726 | 1.160413e-15 | | | * |
| **B.1.1** | 183 | 43 | 0.448867 | -2.027831 | 122.055662 | 5.683601e-01 | * | | * |
| **B.1.8** | 74 | 38 | 0.231112 | -8.096307 | 134.192614 | 1.320163e-03 | * | | * |
| **B.1.7** | 134 | 38 | 0.231112 | -5.475856 | 128.951712 | 1.813340e-02 | * | | * |
| **B.1.17** | 16 | 37 | 0.190290 | -28.808872 | 175.617743 | 1.331121e-12 | | | * |
| **B.1.5** | 59 | 37 | 0.190290 | -18.511097 | 155.022194 | 3.957770e-08 | | | * |
| **B.1.6** | 14 | 37 | 0.190290 | -6.093518 | 130.187037 | 9.754759e-03 | * | | * |
| **B.1.12** | 61 | 36 | 0.169076 | -17.069705 | 152.139410 | 1.670454e-07 | | | * |

### B.1.3 <<

| | num_seq | t_ch_MAX | max_CL | CL@BC_t_ch_MAX | aic | PV | PV_OK | t_ch_MAX_OK | phyl_OK |
|---|---|---|---|---|---|---|---|---|---|
| **B.1.3** | 138 | 57 | 3.559497 | None | None | None | * | * | * |

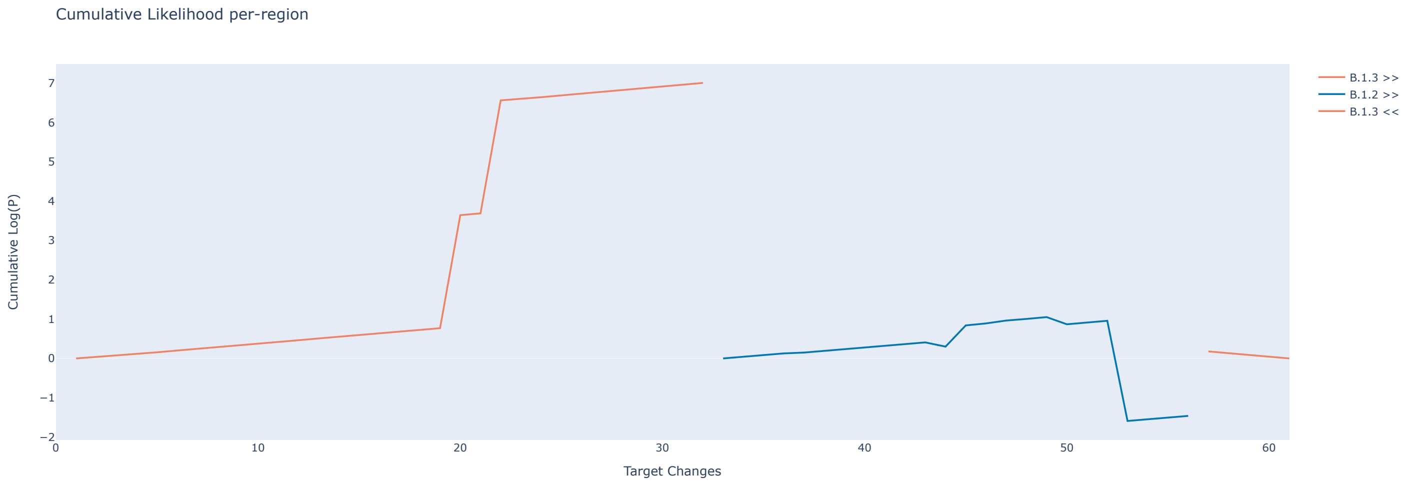


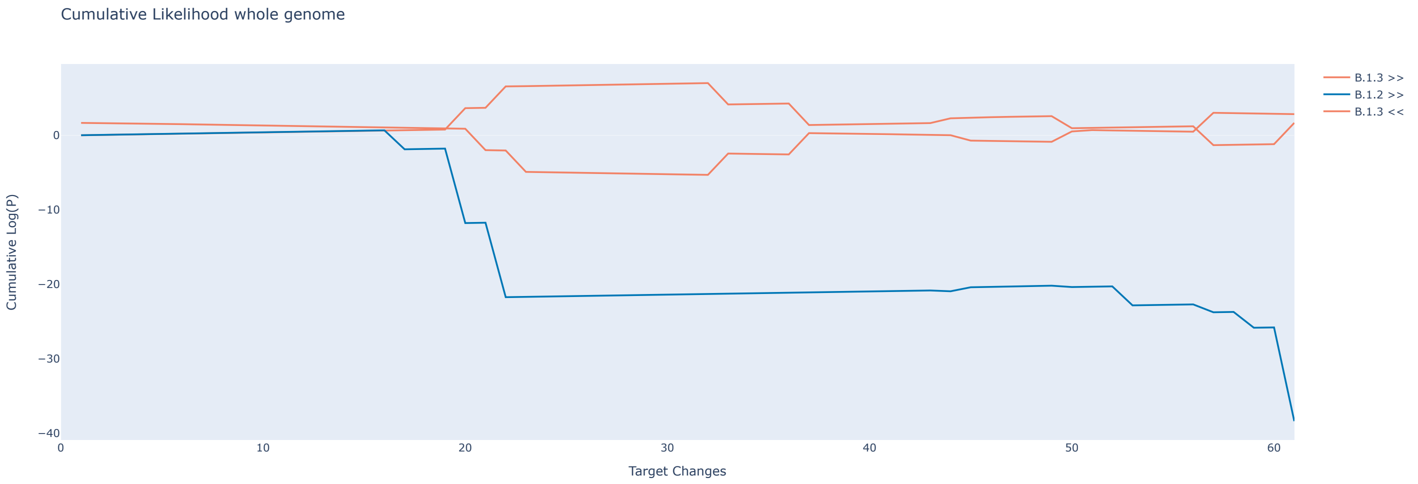


**Target sequence**

, 7927_A|G, 7934_C|T, 9945_A|G, 14163_G|T, 15591_G|A, 21886_G|A, 23727_C|T, 25824_G|A, 30530_G|A, 31216_G|A, 34622_G|A, 37365_G|A, 38523_G|A, 38825_C|T, 39282_C|T, 39302_C|T, 53048_G|A, 54280_G|A, 54798_G|A, 55296_G|A, 64460_G|A, 64589_C|T, 72525_C|T, 73229_C|T, 73402_G|A, 74368_G|A, 77546_G|A, 81438_G|A, 82536_C|T, 82614_G|A, 83489_C|T, 84750_C|T, 87393_G|A, 87460_G|A, 91891_G|A, 95197_G|A, 109899_A|G, 119459_C|T, 121483_C|T, 124293_G|A, 124837_G|A, 125412_G|A, 128861_C|T, 133266_TTTN|...., 133328_T|C, 133331_CAATCTTTCT|.........., 147062_...................|TATATTTTATTTTATATTT, 148563_G|A, 150620_C|T, 150736_GATATGATGGATATGAT|................., 151612_A|C, 155946_G|A, 162394_G|A, 162482_C|T, 164983_C|T, 168271_C|T, 182170_G|A, 183709_C|T, 186768_G|A, 187618_C|T, 190850_G|A

## Case 5: ON754987.1

Target: 138 samples    Number of changes: 62
GT: B.1.3
BC: B.1.3 + B.1.2 + B.1.3    BC BR: 32-33, 50-51    BC BR coord: 85217 - 85218, 150981 - 150982
Direction L1: >>    Initial region span: [[0, 32], [32, 49], [57, 62]]    Gap history (edge excluded): 32-58 -> 49-58
Alt. candidates: [], []
Model 1BP/2BP comparison: 2BP vs 1BP: 1.39e-01
Rec. model vs L1: 1.45e-16    Rec. model vs L2: 3.82e-52
Flags: Model_2BP_Best

### B.1.3 >>

| | num_seq | t_ch_MAX | max_CL | CL@BC_t_ch_MAX | aic | PV | PV_OK | t_ch_MAX_OK | phyl_OK |
|---|---|---|---|---|---|---|---|---|---|
| **B.1.3** | 138 | 32 | 7.024316 | NaN | NaN | NaN | * | * | * |
| **B.1.6** | 14 | 19 | 0.828990 | -38.271904 | 200.543807 | 8.558047e-22 | | | * |
| **B.1.13** | 34 | 19 | 0.828990 | -38.380269 | 200.760539 | 7.666591e-22 | | | * |
| **B.1** | 1309 | 19 | 0.815200 | -16.453608 | 156.907215 | 2.549816e-12 | | | * |
| **B.1.2** | 189 | 16 | 0.680611 | -40.792497 | 205.584994 | 6.885771e-23 | | | * |
| **B.1.12** | 61 | 7 | 0.273744 | -42.119921 | 208.239843 | 1.821130e-23 | | | * |
| **B.1.8** | 74 | 6 | 0.232510 | -52.340722 | 228.681445 | 6.635159e-28 | | | * |
| **B.1.5** | 59 | 6 | 0.232510 | -49.588355 | 223.176711 | 1.037913e-26 | | | * |
| **B.1.1** | 183 | 6 | 0.232510 | -44.363159 | 212.726319 | 1.929078e-24 | | | * |
| **B.1.11** | 94 | 5 | 0.186725 | -45.646537 | 215.293075 | 5.363557e-25 | | | * |

### B.1.2 >>

| | num_seq | t_ch_MAX | max_CL | CL@BC_t_ch_MAX | aic | PV | PV_OK | t_ch_MAX_OK | phyl_OK |
|---|---|---|---|---|---|---|---|---|---|
| **B.1.2** | 189 | 49 | 1.092474 | NaN | NaN | NaN | * | * | * |
| **B.1** | 1309 | 55 | 0.735965 | 0.512287 | 72.975425 | 6.703200e-01 | * | | * |
| **B.1.14** | 22 | 43 | 0.448867 | -15.373172 | 104.746343 | 8.462806e-08 | | | * |
| **B.1.1** | 183 | 43 | 0.448867 | -1.454825 | 76.909650 | 9.394930e-02 | * | | * |
| **B.1.8** | 74 | 38 | 0.231112 | -4.560554 | 83.121107 | 4.211232e-03 | * | | * |
| **B.1.7** | 134 | 38 | 0.231112 | -4.862880 | 83.725760 | 3.104198e-03 | * | | * |
| **B.1.17** | 16 | 37 | 0.190290 | -14.928170 | 103.856340 | 1.320613e-07 | | | * |
| **B.1.5** | 59 | 37 | 0.190290 | -11.336694 | 96.673388 | 4.809103e-06 | | | * |
| **B.1.6** | 14 | 37 | 0.190290 | -6.287269 | 86.574537 | 7.503281e-04 | * | | * |
| **B.1.12** | 61 | 36 | 0.169076 | -13.523803 | 101.047607 | 5.382192e-07 | | | * |

### B.1.3 <<

| | num_seq | t_ch_MAX | max_CL | CL@BC_t_ch_MAX | aic | PV | PV_OK | t_ch_MAX_OK | phyl_OK |
|---|---|---|---|---|---|---|---|---|---|
| **B.1.3** | 138 | 58 | 3.559497 | None | None | None | * | * | * |

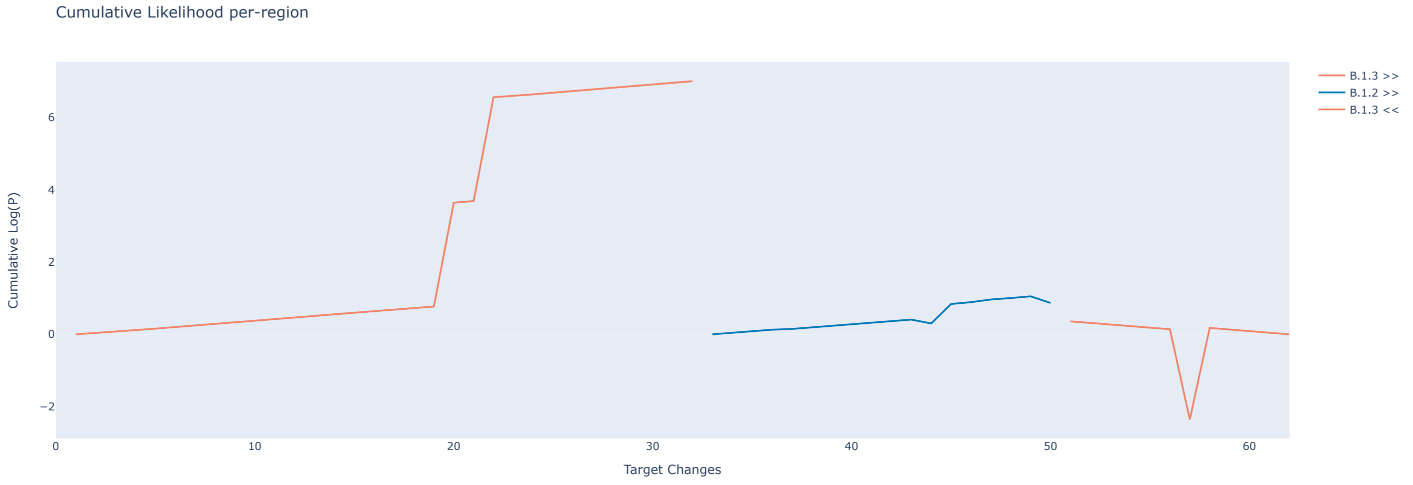


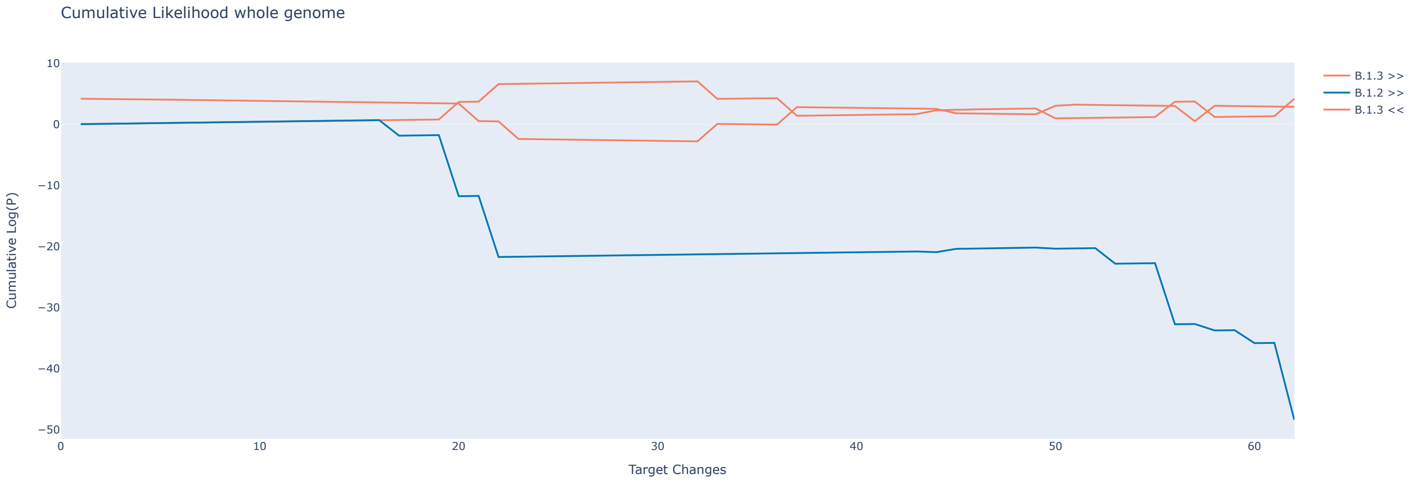


**Target sequence**

, 7927_A|G, 7934_C|T, 9945_A|G, 14163_G|T, 15591_G|A, 21886_G|A, 23727_C|T, 25824_G|A, 30530_G|A, 31216_G|A, 34622_G|A, 37365_G|A, 38523_G|A, 38825_C|T, 39282_C|T, 39302_C|T, 53048_G|A, 54280_G|A, 54798_G|A, 55296_G|A, 64460_G|A, 64589_C|T, 72525_C|T, 73229_C|T, 73402_G|A, 74368_G|A, 77546_G|A, 81438_G|A, 82536_C|T, 82614_G|A, 83489_C|T, 84750_C|T, 87393_G|A, 87460_G|A, 91891_G|A, 95197_G|A, 109899_A|G, 119459_C|T, 121483_C|T, 124293_G|A, 124837_G|A, 125412_G|A, 128861_C|T, 133266_TTTN|...., 133328_T|C, 133331_CAATCTTTCT|.........., 147062_...................|TATATTTTATTTTATATTT, 148563_G|A, 150620_C|T, 150736_GATATGATGGATATGAT|................., 151612_A|C, 155946_G|A, 162394_G|A, 162482_C|T, 164983_C|T, 167668_G|A, 168271_C|T, 182170_G|A, 183709_C|T, 186768_G|A, 187618_C|T, 190850_G|A

## Case 6: ON631241.1

Target: 1309 samples
GT: B.1
BC: B.1
Direction L1: >>
Alt. candidates: []
Model 1BP/2BP comparison:
Rec. model vs L1: –
Flags: Model_2BP_NotEnoughSpace_ForL2, Model_2BP_Bad_L2, SingleCandidateGenome

Number of changes: 58
BC BR:
BC BR coord:
Initial region span: [[0, 54]]
Gap history (edge excluded):
–
Rec. model vs L2: –

### B.1 >>

| | num_seq | t_ch_MAX | max_CL | CL@BC_t_ch_MAX | aic | PV | PV_OK | t_ch_MAX_OK | phyl_OK |
|---|---|---|---|---|---|---|---|---|---|
| **B.1** | 1309 | 54 | 2.087127 | NaN | NaN | NaN | * | * | * |
| **B.1.6** | 14 | 35 | 1.515887 | -4.559172 | 231.118344 | 1.495941e-03 | * | | * |
| **B.1.13** | 34 | 30 | 1.325598 | -12.250057 | 246.500115 | 6.842106e-07 | | | * |
| **B.1.3** | 138 | 19 | 0.785247 | -15.533215 | 253.066430 | 2.561725e-08 | | | * |
| **B.1.2** | 189 | 16 | 0.680611 | -8.378233 | 238.756466 | 3.280269e-05 | * | | * |
| **B.1.12** | 61 | 7 | 0.273744 | -23.073709 | 268.147418 | 1.361294e-11 | | | * |
| **B.1.8** | 74 | 6 | 0.232510 | -20.276468 | 262.552936 | 2.238601e-10 | | | * |
| **B.1.5** | 59 | 6 | 0.232510 | -27.855829 | 277.711657 | 1.142943e-13 | | | * |
| **B.1.1** | 183 | 6 | 0.232510 | -5.817984 | 233.635968 | 4.243296e-04 | * | | * |
| **B.1.11** | 94 | 5 | 0.186725 | -14.892083 | 251.784166 | 4.882614e-08 | | | * |

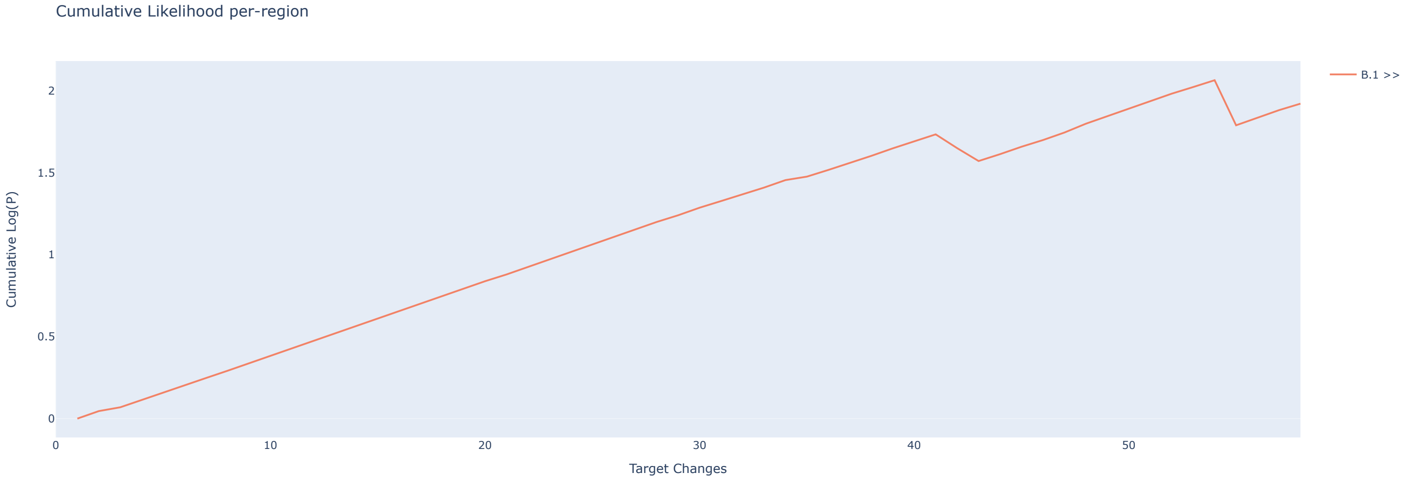


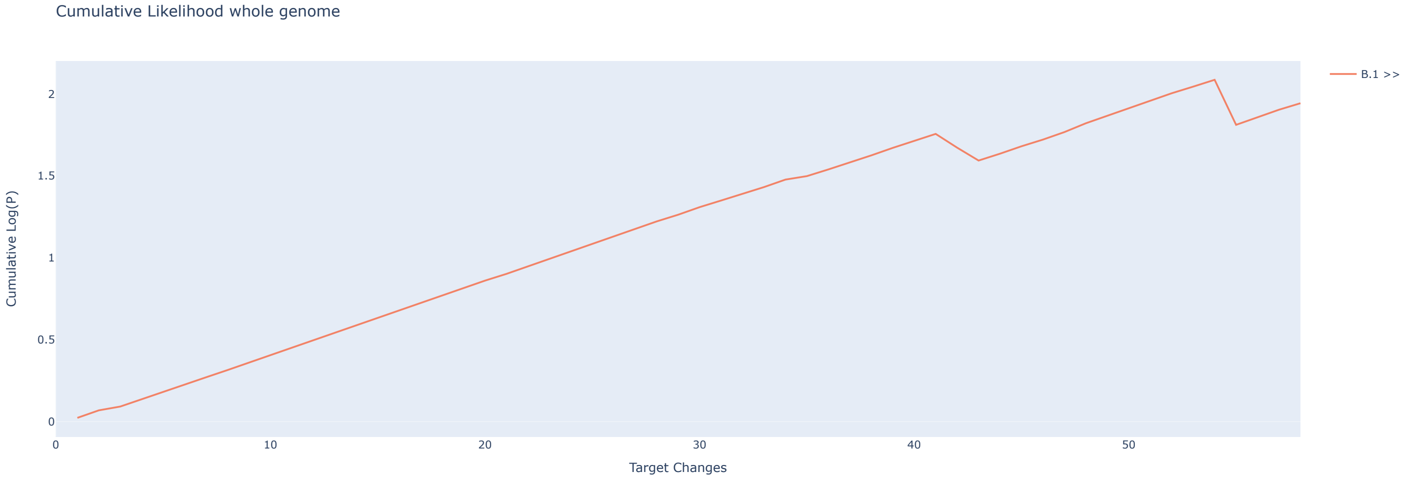


**Target sequence**

, 7927_A|G, 7934_C|T, 9945_A|G, 14163_G|T, 15591_G|A, 21886_G|A, 23727_C|T, 25824_G|A, 30530_G|A, 31216_G|A, 34622_G|A, 37365_G|A, 38523_G|A, 38825_C|T, 39282_C|T, 39302_C|T, 53048_G|A, 54280_G|A, 54798_G|A, 64460_G|A, 72525_C|T, 73229_C|T, 73402_G|A, 74368_G|A, 77546_G|A, 81438_G|A, 82536_C|T, 82614_G|A, 83489_C|T, 84750_C|T, 87393_G|A, 87460_G|A, 91891_G|A, 95197_G|A, 109899_A|G, 119459_C|T, 121483_C|T, 124293_G|A, 124837_G|A, 125412_G|A, 128861_C|T, 133266_TTTN|...., 133328_T|C, 133331_CAATCTTTCT|.........., 147062_...................|TATATTTTATTTTATATTT, 148563_G|A, 150620_C|T, 150736_GATATGATGGATATGAT|................., 151612_A|C, 155946_G|A, 162394_G|A, 162482_C|T, 164983_C|T, 168271_C|T, 182170_G|A, 183709_C|T, 186768_G|A, 187618_C|T

## Case 7: ON755039.1

Target: 1309 samples
GT: B.1
BC: B.1
Direction L1: >>
Alt. candidates: []
Model 1BP/2BP comparison:

Number of changes: 62
BC BR:
BC BR coord:
Initial region span: [[0, 62]]
Gap history (edge excluded):
–

Rec. model vs L1: – Rec. model vs L2: –
Flags: NotEnoughSpaceAfterL1, SingleCandidateGenome

## B.1 >>

| | num_seq | t_ch_MAX | max_CL | CL@BC_t_ch_MAX | aic | PV | PV_OK | t_ch_MAX_OK | phyl_OK |
|---|---|---|---|---|---|---|---|---|---|
| **B.1** | 1309 | 62 | 3.324001 | NaN | NaN | NaN | * | * | * |
| **B.1.6** | 14 | 35 | 1.515887 | -36.196496 | 300.392992 | 6.865657e-18 | | | * |
| **B.1.13** | 34 | 30 | 1.325598 | -43.499069 | 314.998139 | 4.614885e-21 | | | * |
| **B.1.3** | 138 | 19 | 0.785247 | -46.815230 | 321.630459 | 1.676775e-22 | | | * |
| **B.1.2** | 189 | 16 | 0.680611 | -37.883511 | 303.767021 | 1.266848e-18 | | | * |
| **B.1.12** | 61 | 7 | 0.273744 | -30.685562 | 289.371123 | 1.696855e-15 | | | * |
| **B.1.8** | 74 | 6 | 0.232510 | -50.033025 | 328.066050 | 6.699563e-24 | | | * |
| **B.1.5** | 59 | 6 | 0.232510 | -57.357515 | 342.715030 | 4.414072e-27 | | | * |
| **B.1.1** | 183 | 6 | 0.232510 | -34.740358 | 297.480716 | 2.941579e-17 | | | * |
| **B.1.11** | 94 | 5 | 0.186725 | -45.108777 | 318.217554 | 9.224584e-22 | | | * |

Cumulative Likelihood per-region

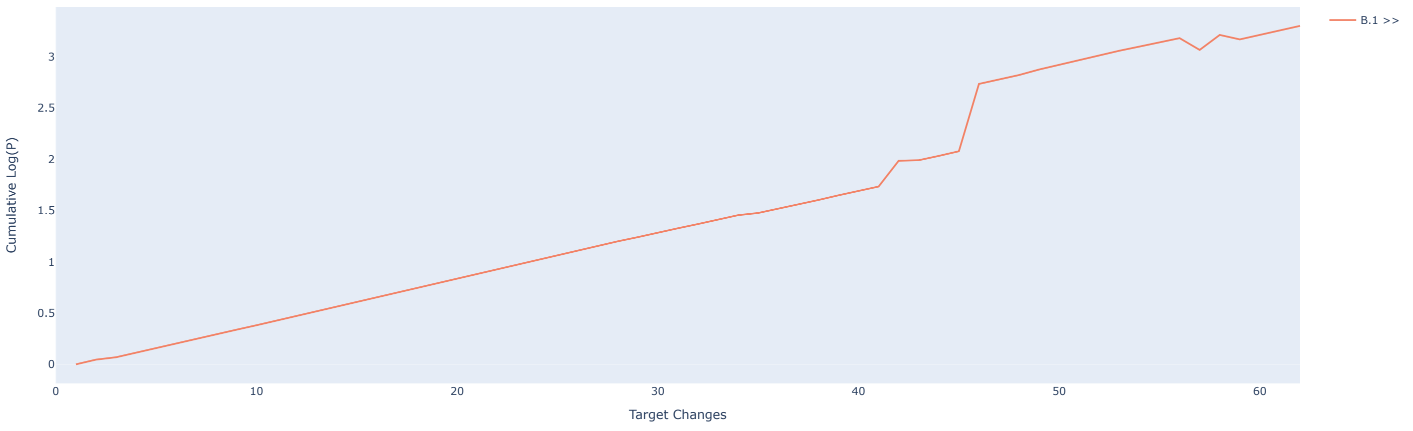


Cumulative Likelihood whole genome

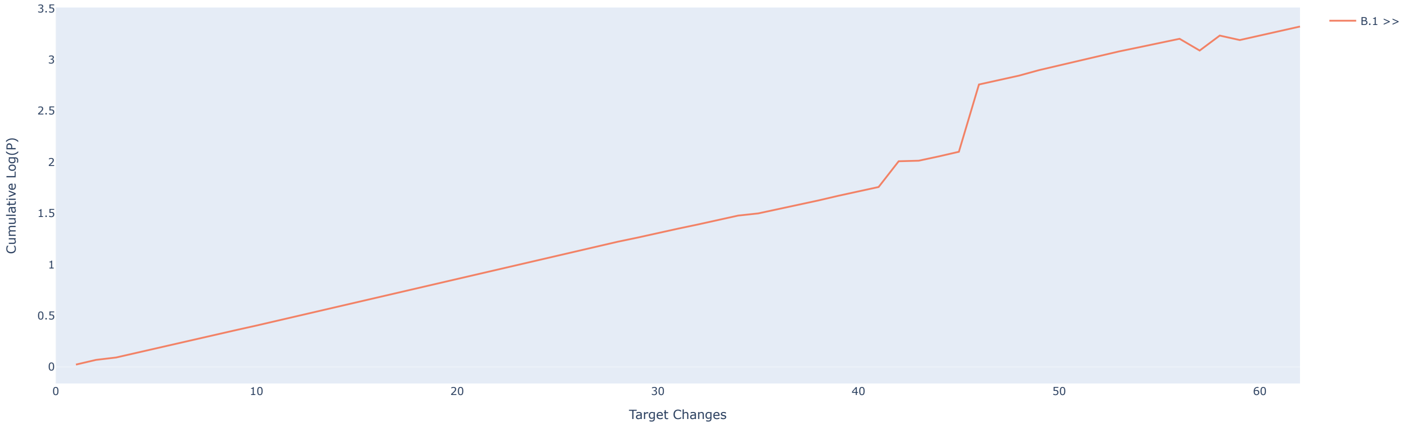


**Target sequence**

, 7927_A|G, 7934_C|T, 9945_A|G, 14163_G|T, 15591_G|A, 21886_G|A, 23727_C|T, 25824_G|A, 30530_G|A, 31216_G|A, 34622_G|A, 37365_G|A, 38523_G|A, 38825_C|T, 39282_C|T, 39302_C|T, 53048_G|A, 54280_G|A, 54798_G|A, 64460_G|A, 72525_C|T, 73229_C|T, 73402_G|A, 74368_G|A, 77546_G|A, 81438_G|A, 82536_C|T, 82614_G|A, 83489_C|T, 84750_C|T, 87393_G|A, 87460_G|A, 91891_G|A, 95197_G|A, 109899_A|G, 119459_C|T, 121483_C|T, 124293_G|A, 124837_G|A, 125412_G|A, 128861_C|T, 133260_TTTTTTTTTN|.........., 133328_T|C, 133331_CAATCTTTCT|.........., 147062_..................|TATATTTTATTTTATATTT, 148419_C|T, 148563_G|A, 150620_C|T, 150736_GATATGATGGATATGAT|................., 151612_A|C, 155946_G|A, 162394_G|A, 162482_C|T, 164983_C|T, 168271_C|T, 170413_G|A, 178320_G|A, 179346_TTATATACATTATATACATTATATACATTATA|..............................., 182170_G|A, 183709_C|T, 186768_G|A, 187618_C|T

## Case 8: ON631963.1

Target: 1309 samples
GT: B.1
BC: B.1
Direction L1: >>
Alt. candidates: []
Model 1BP/2BP comparison: –
Rec. model vs L1: –
Flags: Model_2BP_NotEnoughSpace_ForL2, Model_2BP_Bad_L2, SingleCandidateGenome

Number of changes: 60
BC BR: BC BR coord:
Initial region span: [[0, 56]] Gap history (edge excluded):
Rec. model vs L2: –

# B.1 >>

| | num_seq | t_ch_MAX | max_CL | CL@BC_t_ch_MAX | aic | PV | PV_OK | t_ch_MAX_OK | phyl_OK |
|---|---|---|---|---|---|---|---|---|---|
| **B.1** | 1309 | 56 | 3.938544 | NaN | NaN | NaN | * | * | * |
| **B.1.8** | 74 | 5 | 0.186725 | -50.169293 | 326.338585 | 3.658460e-24 | | | * |
| **B.1.6** | 14 | 5 | 0.186725 | -36.498304 | 298.996609 | 3.163034e-18 | | | * |
| **B.1.5** | 59 | 5 | 0.186725 | -57.590945 | 341.181891 | 2.191963e-27 | | | * |
| **B.1.2** | 189 | 5 | 0.186725 | -38.164099 | 302.328197 | 5.984165e-19 | | | * |
| **B.1.13** | 34 | 5 | 0.186725 | -43.800878 | 313.601755 | 2.136752e-21 | | | * |
| **B.1.12** | 61 | 5 | 0.186725 | -55.032418 | 336.064835 | 2.835483e-26 | | | * |
| **B.1.11** | 94 | 5 | 0.186725 | -45.410585 | 316.821171 | 4.271103e-22 | | | * |
| **B.1.10** | 25 | 5 | 0.186725 | -51.801635 | 329.603271 | 7.168005e-25 | | | * |
| **B.1.1** | 183 | 5 | 0.186725 | -34.942561 | 295.885121 | 1.497724e-17 | | | * |

Cumulative Likelihood per-region

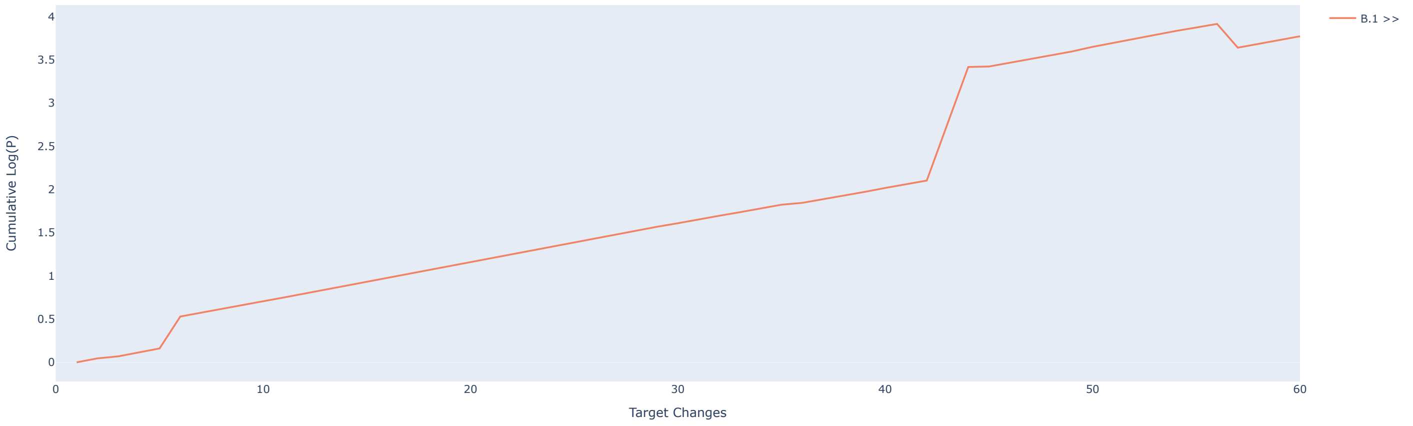


Cumulative Likelihood whole genome

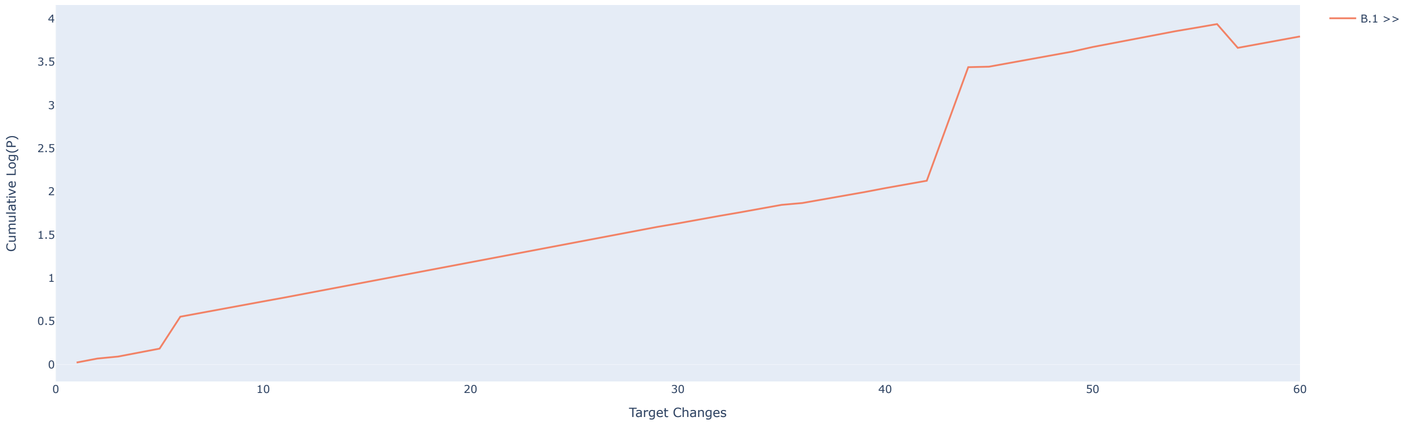


**Target sequence**

, 7927_A|G, 7934_C|T, 9945_A|G, 14163_G|T, 15591_G|A, 21291_C|T, 21886_G|A, 23727_C|T, 25824_G|A, 30530_G|A, 31216_G|A, 34622_G|A, 37365_G|A, 38523_G|A, 38825_C|T, 39282_C|T, 39302_C|T, 53048_G|A, 54280_G|A, 54798_G|A, 64460_G|A, 72525_C|T, 73229_C|T, 73402_G|A, 74368_G|A, 77546_G|A, 81438_G|A, 82536_C|T, 82614_G|A, 83489_C|T, 84750_C|T, 87393_G|A, 87460_G|A, 91891_G|A, 95197_G|A, 109899_A|G, 119459_C|T, 121483_C|T, 124293_G|A, 124837_G|A, 125412_G|A, 128861_C|T, 133238_...|TTT, 133240_C|T, 133328_T|C, 133331_CAATCTTTCT|.........., 147062_..................|TATATTTTATTTTATATTT, 148563_G|A, 150620_C|T, 150736_GATATGATGGATATGAT|................., 151612_A|C, 155946_G|A, 162394_G|A, 162482_C|T, 164983_C|T, 168271_C|T, 182170_G|A, 183709_C|T, 186768_G|A, 187618_C|T

**Supplementary Notes 5**

Detailed report on GISAID cases on which RH disagrees with Pango lineage GT. This document extends the discussion presented in the 'Lineage analysis using consensus-genomes' section.

# Detailed report on GISAID cases
# on which RH disagrees with Pango lineage GT

# Summary of contents

- 57 cases found (with any number of "good" sequences available up to April 1st):
  - 1BP: 45
  - 2BP: 6
  - 3B or more: 2 (XAY, XBC)
  - 1/2BP with ill-defined ground truth: 4 (XB, XAS, XAJ, XBK), i.e. cases with uncertain donor/acceptor lineages or breakpoint position
- We discuss the comparison of 1BP and 2BP results only for cases where the ground truth is sound: 51 cases.
- We reduced input noise using the consensus sequence
- Results:
  - 40 cases mathcing the ground truth
    - 37 cases single breakpoint
    - 3 cases double breakpoint
  - 11 cases needing discussion divided in three groups:

    G1: 6 cases that we judge being not-recombinant (recombination is based on 1/2 mutations: XAV, XAR, XBF, XN, XAK, XAZ)
    G2: 2 cases where our method suggests a more probable model (XBM, XBH)
    G3: 3 cases highlights the limits of our approach (XAT, XAW, XP)

# Discussion on G1 cases

Non recombinant cases

# XAR, XN, XBF

- 1 breakpoint at 5'
  - XAR: only 2 mutations left at 5' to find L2  (detailed explanation follows in next slides)
- 1 breakpoint at 3'
  - XN: only 2 mutations left at 3' to find L2
  - XBF: only 2 mutations left at 3' to find L2 (detailed explanation follows in next slides)

# XAR

**Case 29 (1BP 5'): XAR** **test: KO**

**Target**: (75%) 12 samples
**GT**: BA.1* + BA.2*
**BC**: BA.2
**Direction L1**: <<
**Alt. candidates**: [BA.2.10]
**Model 1BP/2BP comparison**: -

**Number of changes**: 64
**GT BR**: 1-3
**GT BR coord**: 2833 - 4184
**Rank L1 L2**: 11 -
**BC BR**:
**BC BR coord**:
**Initial region span**: 2-64
**Gap history (edge excluded)**:

| | BA.1 | BA.2 | seq_change | t_pos | merg_pos |
|---|---|---|---|---|---|
| 241_C\|T | True | True | False | 0 | 241 |
| 670_T\|G | False | True | False | 0 | 670 |
| 2790_C\|T | False | True | False | 0 | 2790 |
| 2832_A\|G | True | False | True | 1 | 2832 |
| 3037_C\|T | True | True | True | 2 | 3037 |
| 4184_G\|A | False | True | True | 3 | 4184 |
| 4321_C\|T | False | True | True | 4 | 4321 |
| 5386_T\|G | True | False | False | 4 | 5386 |
| 6513_GTT\|... | True | False | False | 4 | 6513 |
| 8393_G\|A | True | False | False | 4 | 8393 |
| 9344_C\|T | False | True | True | 5 | 9344 |

} breakpoint. ↑ BA.1, ↓ BA.2 according to GT

only 2 mutations of target to detect recombination

**L1 BA.2 <<**

| | num_seq | t_ch_MAX | max_CL | CL@BC_t_ch_MAX | aic | PV | PV_OK | t_ch_MAX_OK | phyl_OK |
|---|---|---|---|---|---|---|---|---|---|
| **BA.2** | 378689 | 2 | 50.565165 | NaN | NaN | NaN | * | * | * |
| **BA.2.37** | 2257 | 5 | 46.914125 | 29.282294 | 103.435411 | 1.078435e-06 | | | * |
| **BA.2.10** | 28641 | 2 | 45.750688 | 36.898300 | 88.203400 | 2.198456e-03 | * | * | * |
| **BA.2.3** | 36275 | 5 | 43.763269 | 33.970321 | 94.059359 | 1.173910e-04 | * | | * |
| **BA.2.9** | 108830 | 2 | 42.440214 | 30.395057 | 101.209886 | 3.288760e-06 | | * | * |
| **BA.2.23** | 3557 | 2 | 41.440215 | 23.311004 | 115.377993 | 2.754582e-09 | | * | * |
| **BA.2.29** | 1418 | 2 | 41.083370 | 20.819526 | 120.360948 | 2.283823e-10 | | * | * |
| [illegible] | [illegible] | [illegible] | 40.620877 | 21.495835 | 119.008330 | 4.485504e-10 | | * | * |
| [illegible] | [illegible] | [illegible] | 40.001516 | 18.702153 | 124.595694 | 2.741310e-11 | | * | * |
| [illegible] | [illegible] | [illegible] | 39.996156 | 17.327281 | 127.345438 | 6.931118e-12 | | | * |

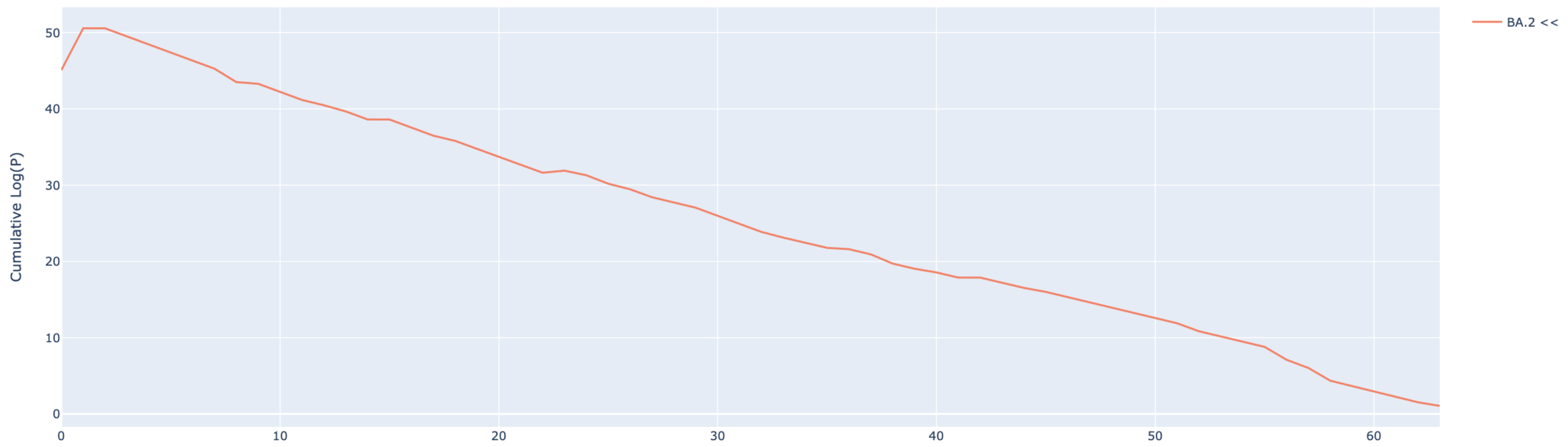

# XBF

**Case 12 (1BP mid): XBF** **test: KO**

Target: (75%) 4944 samples
GT: BA.5.2 + CJ.1
BC: BM.1.1.1
Direction L1: <<

Number of changes: 84
GT BR: 14-16
BC BR:
Initial region span: 2-84

GT BR coord: 9864 - 9867
BC BR coord:
Gap history (edge excluded):

Rank L1 L2: 11 -

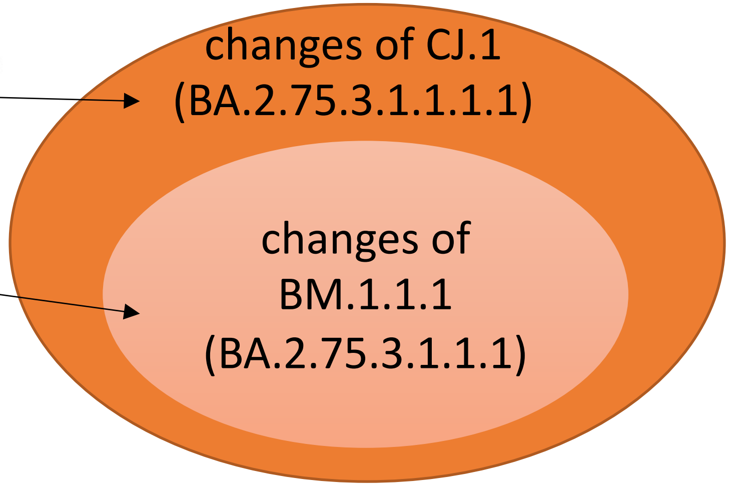


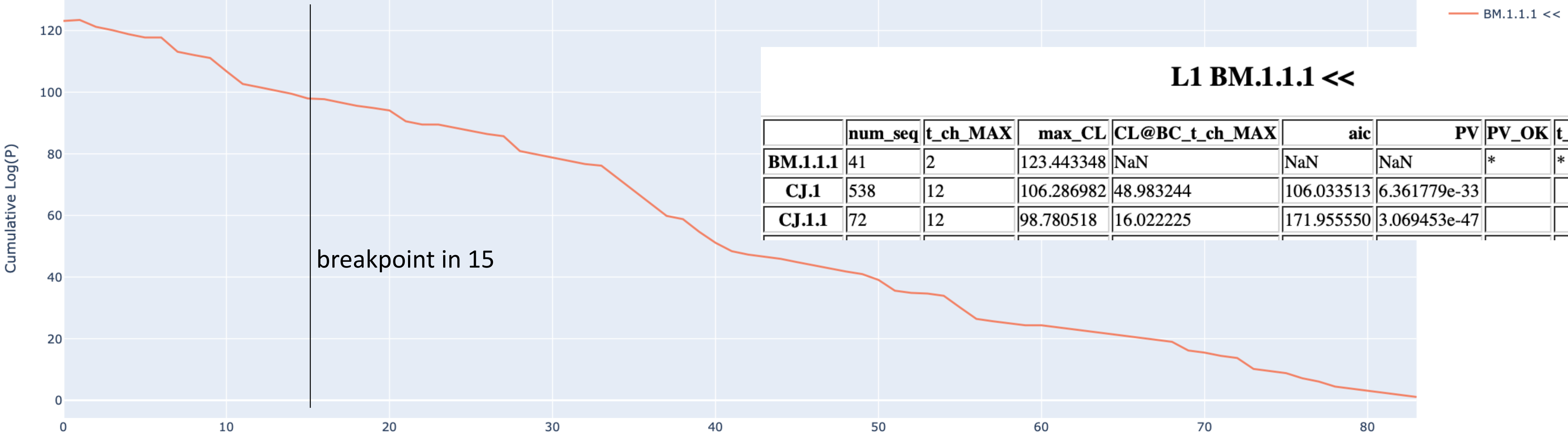


**L1 BM.1.1.1 <<**

| | num_seq | t_ch_MAX | max_CL | CL@BC_t_ch_MAX | aic | PV | PV_OK | t_ch_MAX_OK | phyl_OK |
|---|---|---|---|---|---|---|---|---|---|
| **BM.1.1.1** | 41 | 2 | 123.443348 | NaN | NaN | NaN | * | * | * |
| **CJ.1** | 538 | 12 | 106.286982 | 48.983244 | 106.033513 | 6.361779e-33 | | | * |
| **CJ.1.1** | 72 | 12 | 98.780518 | 16.022225 | 171.955550 | 3.069453e-47 | | | * |

# XBF

**L1 BM.1.1.1 <<**

| | num_seq | t_ch_MAX | max_CL | CL@BC_t_ch_MAX | aic | PV | PV_OK | t_ch_MAX_OK | phyl_OK |
|---|---|---|---|---|---|---|---|---|---|
| **BM.1.1.1** | 41 | 2 | 123.443348 | NaN | NaN | NaN | * | * | * |
| **CJ.1** | 538 | 12 | 106.286982 | 48.983244 | 106.033513 | 6.361779e-33 | | | * |
| **CJ.1.1** | 72 | 12 | 98.780518 | 16.022225 | 171.955550 | 3.069453e-47 | | | * |

GT candidate from 5' to BP ↓ (BA.5.2)

| | CJ.1 | BA.5.2 | BM.1.1.1 | seq_change | t_pos |
|---|---|---|---|---|---|
| 241_C\|T | True | True | False | True | 1 |
| 625_G\|T | False | False | False | True | 2 |
| 670_T\|G | True | True | True | True | 3 |
| 1627_C\|T | False | True | False | True | 4 |
| 2790_C\|T | True | True | True | True | 5 |
| 3037_C\|T | True | True | True | True | 6 |
| 3339_T\|C | False | False | False | True | 7 |
| 3796_C\|T | True | False | False | False | 7 |
| 3927_C\|T | True | False | False | False | 7 |
| 4184_G\|A | True | True | True | True | 8 |
| 4321_C\|T | True | True | True | True | 9 |
| 4586_C\|T | True | False | False | False | 9 |
| 5183_C\|T | True | False | False | False | 9 |
| 6070_C\|T | False | False | False | True | 10 |
| 8692_C\|T | False | False | False | True | 11 |
| 9344_C\|T | True | True | True | True | 12 |
| 9424_A\|G | True | True | True | True | 13 |
| 9534_C\|T | True | True | True | True | 14 |

events supporting BA.5.2 in spite of BM.1.1.1 (241_C|T)

events supporting BA.5.2 in spite of BM.1.1.1 (1627_C|T)

events supporting BM.1.1.1 in spite of BA.5.2 (3796_C|T, 3927_C|T)

events supporting BM.1.1.1 in spite of BA.5.2 (4586_C|T, 5183_C|T)

breakpoint. ↑ BA.5.2, ↓ CJ.1 according to GT

GT candidate from 3' to BP ↑ (CJ.1)

our best candidate from 3' to BP ↑ (BM.1.1.1)

<u>Is BM.1.1.1 is favoured with respect to CJ.1 only because of characterisation derived from 41 sequences</u>?

Or should we say that this target is BM.1.1.1 ?

# XAV

- 1 breakpoint in the middle
  - XAV: L1 and L2 from GT differ by 2 mutations before breakpoint

# XAV

**Case 7 (1BP mid): XAV** **test: KO**

Target: (75%) 42 samples
GT: BA.2* + BA.5*
BC: BA.5.1.24
Direction L1: >>
Alt. candidates: [BA.5]
Model 1BP/2BP comparison: -
Rec. model vs L1: -
Flags: NotEnoughSpaceAfterL1, SingleCandidateGenome

Number of changes: 70
GT BR: 19-22
BC BR:
Initial region span: 1-69
Rec. model vs L2: -

GT BR coord: 15959 - 17279 Rank L1 L2: 11 -
BC BR coord:
Gap history (edge excluded):

## BA.5.1.24 >>

| | num_seq | t_ch_MAX | max_CL | CL@BC_t_ch_MAX | aic | PV | PV_OK | t_ch_MAX_OK | phyl_OK |
|---|---|---|---|---|---|---|---|---|---|
| **BA.5.1.24** | 3729 | 69 | 55.366186 | NaN | NaN | NaN | * | * | * |
| **BA.5** | 16505 | 70 | 46.645542 | 46.645542 | 78.708916 | 1.773146e-03 | * | * | * |
| **BA.5.3.1** | 4372 | 62 | 37.468326 | 27.227311 | 117.545378 | 6.527481e-12 | | | |
| **BA.5.2.1** | 93917 | 62 | 35.771453 | 28.713219 | 114.573561 | 2.896306e-11 | | | |
| **BA.5.1** | 112751 | 69 | 34.754075 | 32.302796 | 107.394408 | 1.049450e-09 | | * | * |
| **BA.5.5** | 17354 | 62 | 33.231756 | 32.063089 | 107.873821 | 8.255263e-10 | | | |
| **BA.5.1.3** | 5405 | 69 | 32.957703 | 30.515174 | 110.969651 | 1.752163e-10 | | * | |
| **BA.5.1.10** | 6066 | 67 | 31.894366 | 24.687676 | 122.624647 | 5.173794e-13 | | | |
| **BE.1** | 14022 | 62 | 30.047253 | 25.732811 | 120.534379 | 1.471116e-12 | | | |
| **BA.5.1.23** | 5847 | 63 | 29.248469 | 22.599424 | 126.801151 | 6.399317e-14 | | | |

| | BA.5.1.24 | BA.2 | BA.5 | seq_change | t_pos |
|---|---|---|---|---|---|
| 241_C\|T | True | True | True | True | 1 |
| 670_T\|G | True | True | True | True | 2 |
| 2790_C\|T | True | True | True | True | 3 |
| 3037_C\|T | True | True | True | True | 4 |
| 4184_G\|A | True | True | True | True | 5 |
| 4321_C\|T | True | True | True | True | 6 |
| 6606_C\|T | False | False | False | True | 7 |
| 9344_C\|T | True | True | True | True | 8 |
| 9424_A\|G | True | True | True | True | 9 |
| 9534_C\|T | True | True | True | True | 10 |
| 9866_C\|T | False | True | False | True | 11 |
| 10029_C\|T | True | True | True | True | 12 |
| 10198_C\|T | True | True | True | True | 13 |
| 10447_G\|A | True | True | True | True | 14 |
| 10449_C\|A | True | True | True | True | 15 |
| 11288_TC… | True | True | True | True | 16 |
| 12160_G\|A | True | False | True | False | 16 |
| 12880_C\|T | True | True | True | True | 17 |
| 14408_C\|T | True | True | True | True | 18 |
| 15714_C\|T | True | True | True | True | 19 |

breakpoint. ↑ BA.2, ↓ BA.5 according to GT

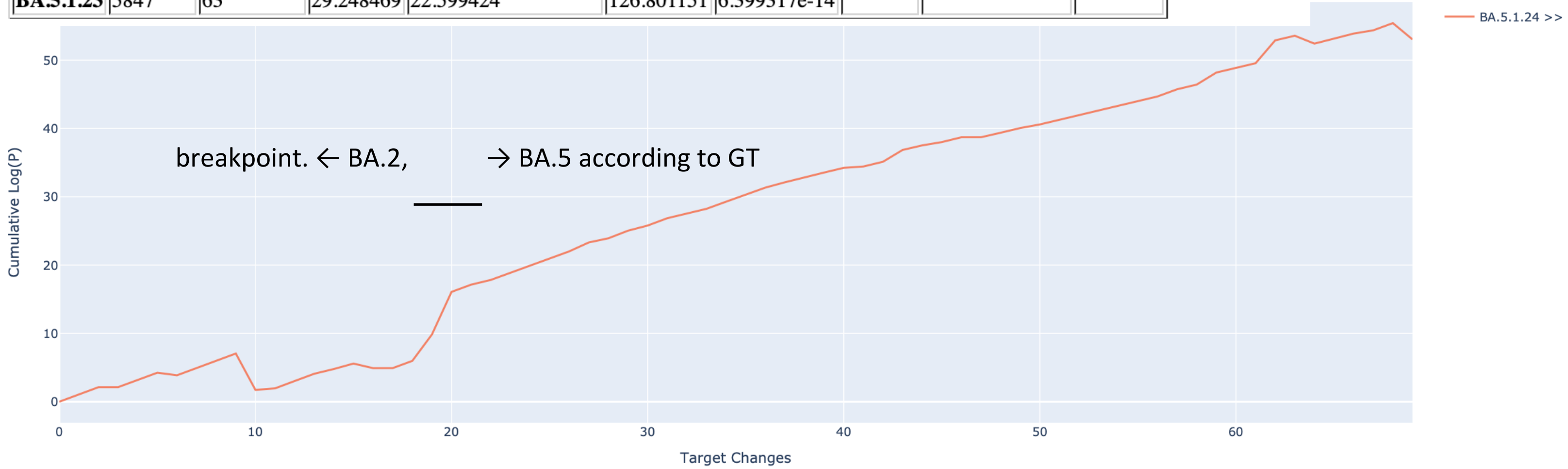


**BA.2 and BA.5 differ by 2 changes only before the breakpoint**

# XAV

| | BA.5.1.24 | BA.2 | BA.5 | seq_change | t_pos | merg_pos |
|---|---|---|---|---|---|---|
| 241_C\|T | True | True | True | True | 1 | 241 |
| 670_T\|G | True | True | True | True | 2 | 670 |
| 2790_C\|T | True | True | True | True | 3 | 2790 |
| 3037_C\|T | True | True | True | True | 4 | 3037 |
| 4184_G\|A | True | True | True | True | 5 | 4184 |
| 4321_C\|T | True | True | True | True | 6 | 4321 |
| 6606_C\|T | False | False | False | True | 7 | 6606 |
| 9344_C\|T | True | True | True | True | 8 | 9344 |
| 9424_A\|G | True | True | True | True | 9 | 9424 |
| 9534_C\|T | True | True | True | True | 10 | 9534 |
| 9866_C\|T | False | True | False | True | 11 | 9866 |
| 10029_C\|T | True | True | True | True | 12 | 10029 |
| 10198_C\|T | True | True | True | True | 13 | 10198 |
| 10447_G\|A | True | True | True | True | 14 | 10447 |
| 10449_C\|A | True | True | True | True | 15 | 10449 |
| 11288_TC… | True | True | True | True | 16 | 11288 |
| 12160_G\|A | True | False | True | False | 16 | 12160 |
| 12880_C\|T | True | True | True | True | 17 | 12880 |
| 14408_C\|T | True | True | True | True | 18 | 14408 |
| 15714_C\|T | True | True | True | True | 19 | 15714 |

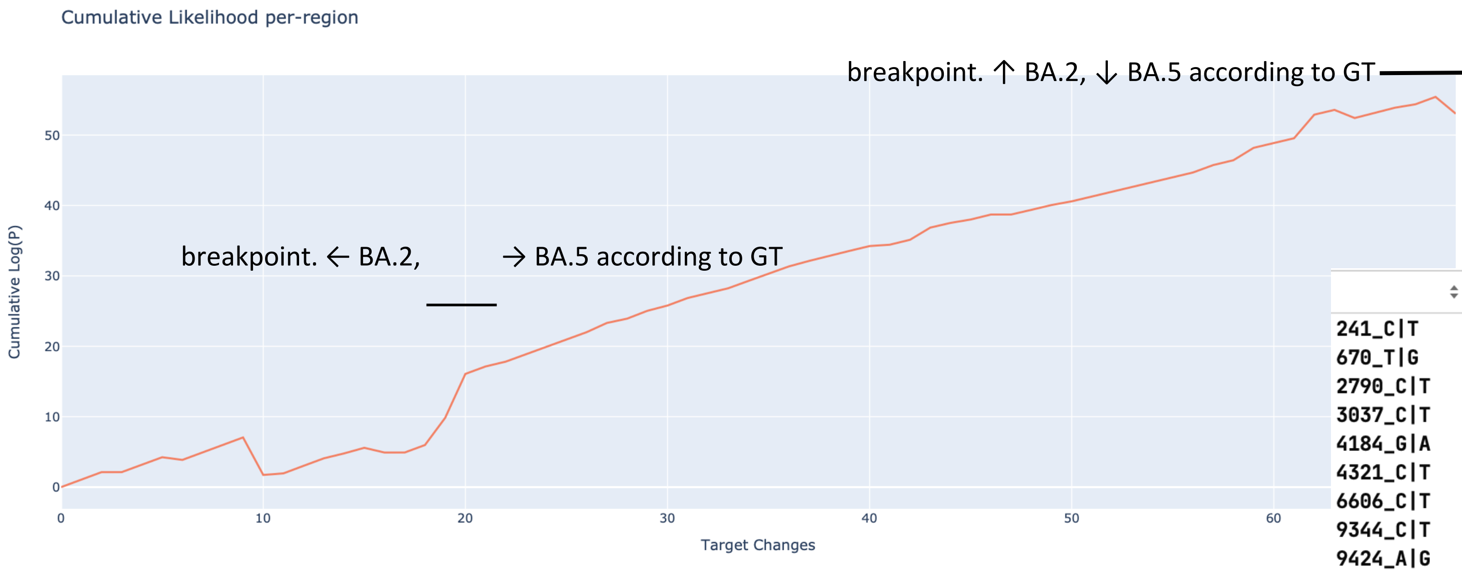


**BA.2 and BA.5 differ by 2 changes only before the breakpoint**

| | BA.5.1.24 | BA.2 | BA.5 | seq_change | t_pos | merg_pos |
|---|---|---|---|---|---|---|
| 241_C\|T | 0.887101 | 0.878372 | 0.833141 | True | 1 | 241 |
| 670_T\|G | 0.998927 | 0.996007 | 0.995214 | True | 2 | 670 |
| 2790_C\|T | 0.995441 | 0.995001 | 0.992729 | True | 3 | 2790 |
| 3037_C\|T | 0.998391 | 0.998637 | 0.996971 | True | 4 | 3037 |
| 4184_G\|A | 0.999195 | 0.997380 | 0.997879 | True | 5 | 4184 |
| 4321_C\|T | 0.985787 | 0.985550 | 0.978188 | True | 6 | 4321 |
| 6606_C\|T | 0.000536 | 0.000499 | 0.000364 | True | 7 | 6606 |
| 9344_C\|T | 0.995441 | 0.993992 | 0.989337 | True | 8 | 9344 |
| 9424_A\|G | 0.984446 | 0.984959 | 0.974735 | True | 9 | 9424 |
| 9534_C\|T | 0.990078 | 0.981040 | 0.987034 | True | 10 | 9534 |
| 9866_C\|T | 0.000805 | 0.959909 | 0.003151 | True | 11 | 9866 |
| 10029_C\|T | 0.998927 | 0.998357 | 0.998788 | True | 12 | 10029 |
| 10198_C\|T | 0.986860 | 0.967488 | 0.989215 | True | 13 | 10198 |
| 10447_G\|A | 0.998927 | 0.996047 | 0.996849 | True | 14 | 10447 |
| 10449_C\|A | 0.998927 | 0.997681 | 0.996244 | True | 15 | 10449 |
| 11288_TCTG… | 0.963261 | 0.973789 | 0.967161 | True | 16 | 11288 |
| 12160_G\|A | 0.997318 | 0.000539 | 0.982672 | False | 16 | 12160 |
| 12880_C\|T | 0.998659 | 0.998078 | 0.977704 | True | 17 | 12880 |
| 14408_C\|T | 0.997318 | 0.998783 | 0.990851 | True | 18 | 14408 |

**The evidence in favor of BA.2 is made of:**

- **1 change of BA.2**
- **1 change of BA.5 that is missing in the target**

# XAZ

- 2 breakpoints
    - XAZ:

      Target should be BA.2.5 + BA.5 + BA.2.5 according to the ground truth.

      Actually, the 2° breakpoint is non-existent and between 5' and 1° breakpoint, the target is closer to BA.2.5 more than BA.5 only for one change.

# XAZ

**BQ.1.9 and BA.5 are on the same branch (BA.5)**
**hence, we propose the non-recombinant model**

Target: (75%) 1390 samples
GT: BA.2.5 + BA.5 + BA.2.5
BC: BQ.1.9 + BA.5
Direction L1: <<
Alt. candidates: [], [BA.5.5, BA.5.2.1, BE.1, BA.5.1]
Model 1BP/2BP comparison: -
Rec. model vs L1: 1.63e-48

Number of changes: 69
GT BR: 8-14, 63-64
BC BR: 3-4
Initial region span: 1-3,9-69
Rec. model vs L2: 3.94e-02

GT BR coord: 3358 - 9866, 27384 - 27387
BC BR coord: 1912 - 1913
Gap history (edge excluded): 3-9

Rank L1 L2: 11 1 -

From 3' up to 2° breakpoint, Target == BA.2 == BA.5

| | BA.2.5 | BA.5 | seq_change | t_pos | merg_pos |
|---|---|---|---|---|---|
| 26858_C\|T | True | False | False | 63 | 26858 |
| 27259_A\|C | True | False | False | 63 | 27259 |
| 27382_GAT\|CTC | True | False | False | 63 | 27382 |
| 27807_C\|T | True | True | True | 64 | 27807 |
| 28271_A\|T | True | True | True | 65 | 28271 |
| 28311_C\|T | True | True | True | 66 | 28311 |
| 28362_GAGAACGCA\|......... | True | True | True | 67 | 28362 |
| 28881_GGG\|AAC | True | True | True | 68 | 28881 |
| 29510_A\|C | True | True | True | 69 | 29510 |

From 2° breakpoint to 1° breakpoint, Target == BA.5 (and ≠ BA.2.5). So... <u>we can say there's no 2° breakpoint!</u>

From 1° breakpoint to 3':

| | BA.2.5 | BA.5 | seq_change | t_pos | merg_pos |
|---|---|---|---|---|---|
| 241_C\|T | True | True | True | 1 | 241 |
| 670_T\|G | True | True | True | 2 | 670 |
| 1912_C\|T | False | False | True | 3 | 1912 |
| 2232_C\|T | False | False | True | 4 | 2232 |
| 2790_C\|T | True | True | True | 5 | 2790 |
| 3037_C\|T | True | True | True | 6 | 3037 |
| 3317_C\|T | True | False | True | 7 | 3317 |
| 3358_T\|C | False | False | True | 8 | 3358 |
| 4184_G\|A | True | True | True | 9 | 4184 |
| 4321_C\|T | True | True | True | 10 | 4321 |
| 9344_C\|T | True | True | True | 11 | 9344 |
| 9424_A\|G | True | True | True | 12 | 9424 |
| 9534_C\|T | True | True | True | 13 | 9534 |
| 9866_C\|T | True | False | False | 13 | 9866 |

<u>single poor evidence of BA.2.5</u>

1° breakpoint region

# XAZ

## We found instead the recombination BQ.1.9 + BA.5. Why?

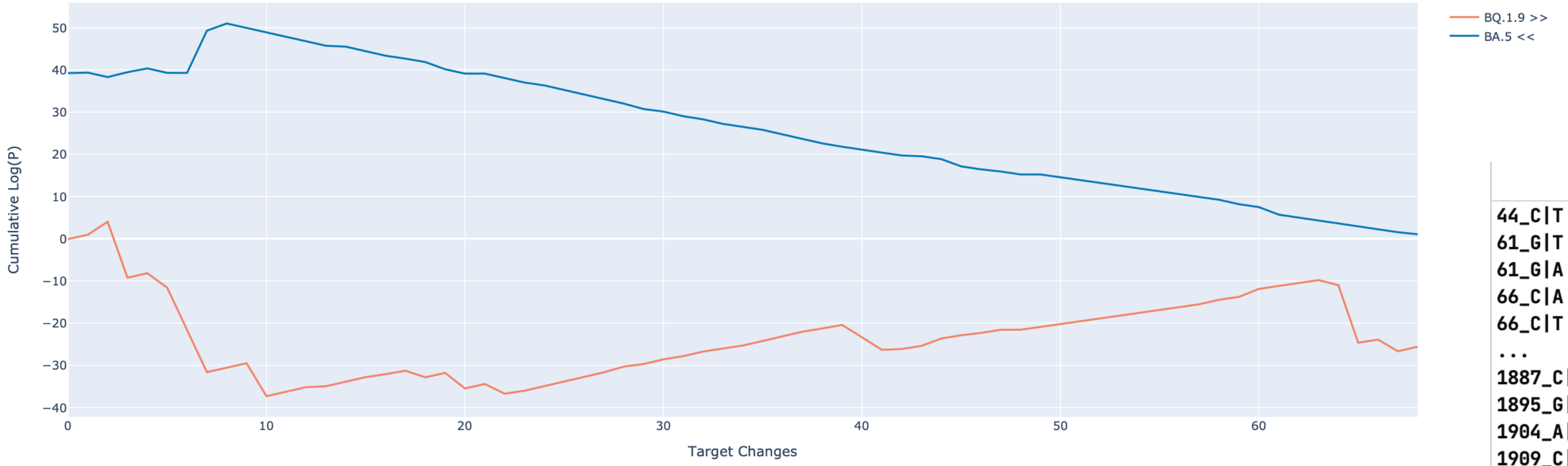


| | 0 |
|---|---|
| 44_C\|T | 0.000000 |
| 61_G\|T | 0.000000 |
| 61_G\|A | 0.000000 |
| 66_C\|A | 0.000000 |
| 66_C\|T | 0.000000 |
| ... | ... |
| 1887_C\|T | 0.958898 |
| 1895_G\|A | 0.958898 |
| 1904_A\|G | 0.958898 |
| 1909_C\|T | 0.958898 |
| 1912_C\|T | 4.016068 |

Because BQ.1 had a subtle spike in likelihood at the 3rd mutation of T (1912_C|T) induced by the higher probability of this change in BQ.1.9 than others

| | BQ.1.9 | BA.2.5 | BA.5 | seq_change | t_pos |
|---|---|---|---|---|---|
| 241_C\|T | 0.862069 | 0.844340 | 0.833141 | True | 1 |
| 670_T\|G | 0.980843 | 0.999476 | 0.995214 | True | 2 |
| 1912_C\|T | 0.111111 | 0.001572 | 0.001636 | True | 3 |
| 1931_C\|A | 1.000000 | 0.000000 | 0.003514 | False | 3 |
| 2232_C\|T | 0.000000 | 0.000000 | 0.000545 | True | 4 |
| 2790_C\|T | 1.000000 | 0.999476 | 0.992729 | True | 5 |
| 2954_T\|C | 1.000000 | 0.000000 | 0.001757 | False | 5 |
| 3037_C\|T | 1.000000 | 0.998428 | 0.996971 | True | 6 |
| 3317_C\|T | 0.000000 | 1.000000 | 0.000000 | True | 7 |
| 3358_T\|C | 0.000000 | 0.004717 | 0.000061 | True | 8 |
| 4184_G\|A | 0.988506 | 0.995807 | 0.997879 | True | 9 |

Moreover, BQ.1.9 is BA.5.3.1.1.1.1.... which again supports non-recombination hypothesis.

# XAZ

**Case 51 (2BP): XAZ** **test: KO**

Target: (75%) 1390 samples
GT: BA.2.5 + BA.5 + BA.2.5
BC: BQ.1.9 + BA.5
Direction L1: <<
Alt. candidates: [], [BA.5.5, BA.5.2.1, BE.1, BA.5.1]
Model 1BP/2BP comparison: -
Rec. model vs L1: 1.63e-48
Flags: Model_1BP_L1eqL2, Model_2BP_Bad_L1_opp

Number of changes: 69
GT BR: 8-14, 63-64
BC BR: 3-4
Initial region span: 1-3,9-69
Rec. model vs L2: 3.94e-02

GT BR coord: 3358 - 9866, 27384 - 27387 Rank L1 L2: 11 1 -
BC BR coord: 1912 - 1913
Gap history (edge excluded): 3-9

**BQ.1.9 >>**

| | num_seq | t_ch_MAX | max_CL | CL@BC_t_ch_MAX | aic | PV | PV_OK | t_ch_MAX_OK | phyl_OK |
|---|---|---|---|---|---|---|---|---|---|
| **BQ.1.9** | 261 | 3 | 4.016068 | NaN | NaN | NaN | * | * | * |
| **BA.2.10.3** | 101 | 3 | 3.292852 | 3.292852 | -0.585703 | 0.486752 | * | * | |
| **BA.5.1.26** | 554 | 7 | 3.157240 | 1.008949 | 3.982101 | 0.049539 | * | | |
| **BA.2.3.14** | 529 | 5 | 3.085477 | 1.724770 | 2.550459 | 0.101266 | * | | |
| **BA.2.32** | 598 | 6 | 3.076592 | 1.782277 | 2.435446 | 0.106992 | * | | |
| **BA.2.65** | 1625 | 6 | 3.025377 | 1.637668 | 2.724663 | 0.093014 | * | | |
| **BA.2.63** | 120 | 3 | 2.892846 | 2.892846 | 0.214307 | 0.326280 | * | * | |
| **BA.2.67** | 258 | 3 | 2.822638 | 2.822638 | 0.354724 | 0.304221 | * | * | |
| **BL.6** | 138 | 3 | 2.780377 | 2.780377 | 0.439247 | 0.290835 | * | * | |
| **BA.5.1.12** | 1348 | 6 | 2.741270 | -0.910132 | 7.820265 | 0.007263 | * | | |

**BA.5 <<**

| | num_seq | t_ch_MAX | max_CL | CL@BC_t_ch_MAX | aic | PV | PV_OK | t_ch_MAX_OK | phyl_OK |
|---|---|---|---|---|---|---|---|---|---|
| **BA.5** | 16505 | 9 | 50.999342 | NaN | NaN | NaN | * | * | * |
| **BA.5.3.3** | 1282 | 11 | 47.514322 | 20.304322 | 117.391355 | 4.822372e-09 | | | * |
| **BA.5.5** | 17354 | 9 | 44.210813 | 31.771002 | 94.457995 | 4.596708e-04 | * | * | * |
| **BA.5.2.1** | 93917 | 9 | 43.237413 | 40.457053 | 77.085894 | 2.718282e+00 | * | * | * |
| **BA.5.3** | 1582 | 9 | 42.804823 | 21.261839 | 115.476321 | 1.253176e-08 | | * | * |
| **BE.1** | 14022 | 9 | 42.490736 | 28.329889 | 101.340221 | 1.473920e-05 | * | * | * |
| **BA.5.1** | 112751 | 9 | 42.465047 | 38.056481 | 81.887037 | 2.465970e-01 | * | * | * |
| **BF.17** | 357 | 11 | 42.264684 | 17.202073 | 123.595854 | 2.161605e-10 | | | * |
| **BA.5.3.2** | 912 | 9 | 41.888687 | 9.715789 | 138.568423 | 1.213618e-13 | | * | * |
| **BF.28** | 4033 | 11 | 41.675675 | 21.192800 | 115.614399 | 1.174310e-08 | | | * |

BA.5.3, BA.5.1 here

and BA.5* there

Despite the candidates of the proposed model are not the same, they are close lineages. **Therefore we judge this case as not recombinant.**

# XAK

**Case 49 (2BP): XAK** **test: KO**

Target: (75%) 110 samples
GT: BA.2* + BA.1* + BA.2*
BC: BA.2
Direction L1: <<
Alt. candidates: []
Model 1BP/2BP comparison: -
Rec. model vs L1: -
Flags: Model_1BP_L1eqL2, Model_2BP_Bad_L2, SingleCandidateGenome

Number of changes: 69
GT BR: 20-23, 23-24
BC BR:
Initial region span: 30-69
Rec. model vs L2: -

GT BR coord: 13194 - 15241, 21617 - 21763
BC BR coord:
Gap history (edge excluded):

Rank L1 L2: 1 - -

GT Breakpoint leaves 1 mutation of target to BA.1 candidate

# Discussion on G2 cases

More probable models with respect to the ground truth

# XBH

- XBH is a 1 breakpoint case according to the GT. RecombinHunt proposes a 2BP model.

**Case 14 (1BP mid): XBH** **test: KO**

Target: (75%) 71 samples
Number of changes: 78
GT: BA.2.3.17 + BA.2.75.2
GT BR: 19-28
GT BR coord: 15450 - 22001
Rank_L1_L2: 11 11 -
BC: BA.2.75.2 + BA.2.3.17 + BA.2.75.2
BC BR: 4-5, 19-20
BC BR coord: 3039 - 3040, 15713 - 15714
Direction L1: <<
Initial region span: 1-4,8-19,20-78
Gap history (edge excluded): 4-20 -> 4-8
Alt. candidates: [], [CA.6, CA.2]
Model 1BP/2BP comparison: 2BP vs 1BP: 3.65e-02
Rec. model vs L1: 4.34e-55
Rec. model vs L2: 3.88e-142
Flags: Model_2BP_Best

Cumulative Likelihood whole genome

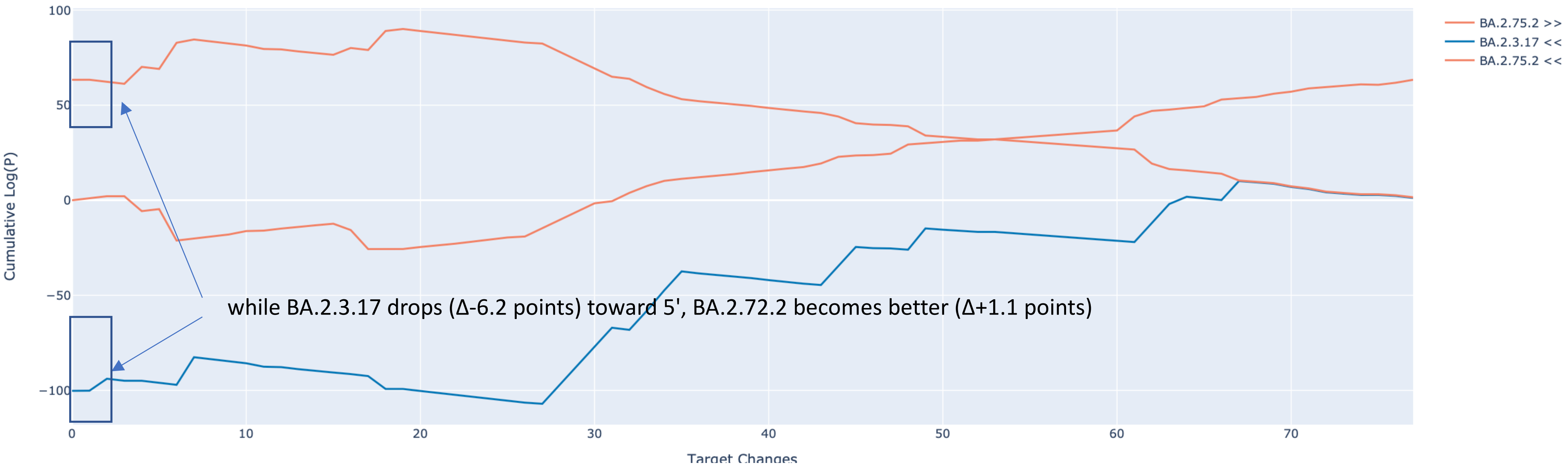

# XBM

- RecombinHunt proposes a 2BP model

**Case 16 (1BP mid): XBM** **test: KO**

Target: (75%) 205 samples Number of changes: 77

GT: BA.2.76 + BF.3 GT BR: 33-42 GT BR coord: 22600 - 22916 Rank L1 L2: 11 11 -

BC: BF.3 + BA.2.76 + BF.3 BC BR: 6-7, 33-34 BC BR coord: 4330 - 4331, 22673 - 22674

Direction L1: << Initial region span: 1-6,25-33,34-77 Gap history (edge excluded): 6-34 -> 6-25

Alt. candidates: [], [BF.3.1]

Model 1BP/2BP comparison: 2BP vs 1BP: 2.47e-11

Rec. model vs L1: 9.42e-38 Rec. model vs L2: 8.83e-96

Flags: Model_2BP_Best

The likelihood plot highlights the high similarity of the proposed model with the target.

Moreover, the P-value comparing the 2BP model against the 1BP model favours the proposed solution (2e-11)

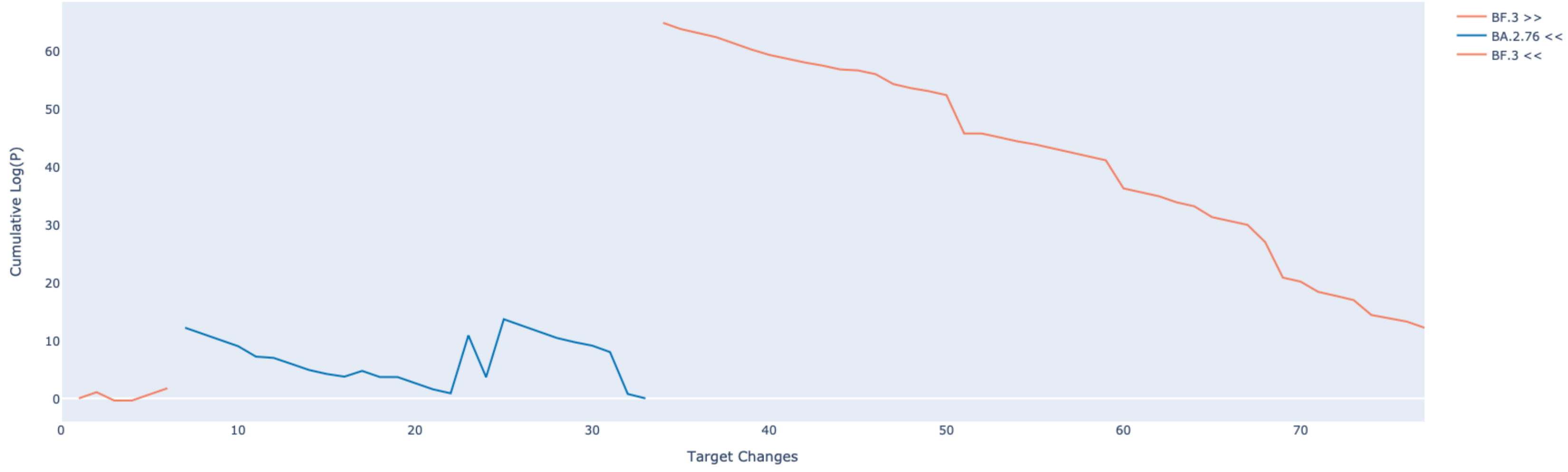

# Discussion on G3 cases

Controversial cases

# XAT

**Case 44 (1BP 3'): XAT** **test: KO**

Target: (75%) 28 samples
GT: BA.2.3.13 + BA.1*
BC: BA.2.3.13
Direction L1: >>
Alt. candidates: []
Model 1BP/2BP comparison: -
Rec. model vs L1: -
Flags: NotEnoughSpaceAfterL1, SingleCandidateGenome

Number of changes: 66
GT BR: 57-59 GT BR coord: 26061 - 26529 Rank L1 L2: 1 -
BC BR: BC BR coord:
Initial region span: 1-66 Gap history (edge excluded):
Rec. model vs L2: -

- Breakpoint is not detected because candidate L1 BA.2.3.13 continues to have common mutations with the target (positions from 59 to 65) even after the breakpoint region.

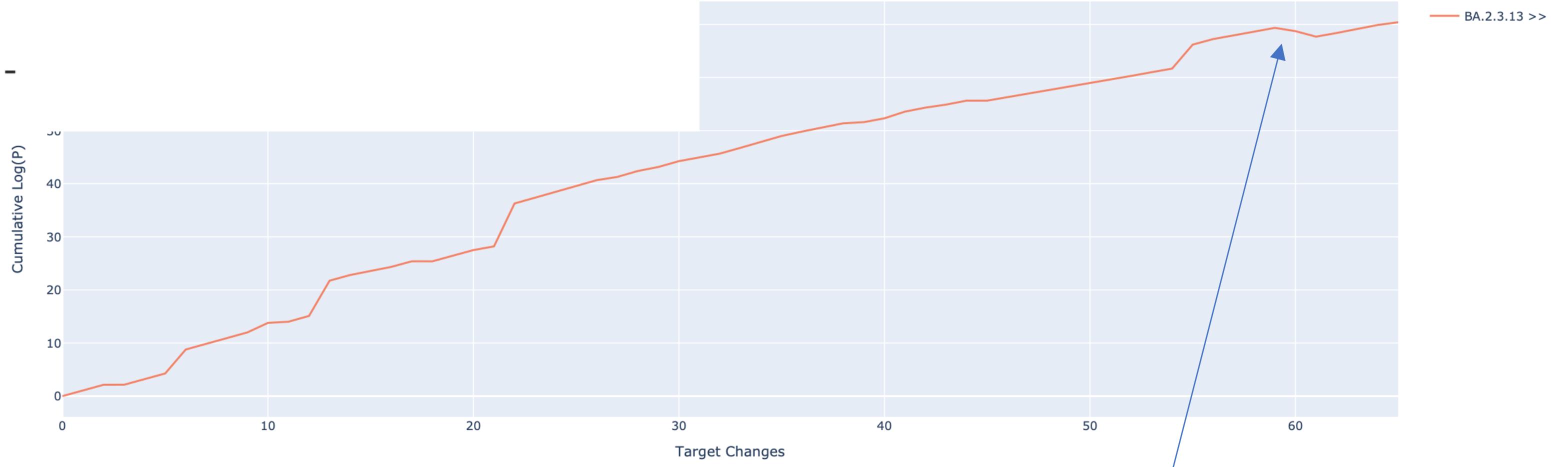


likelihood drop caused by the two mutations at 27K is "only" Δ-2.3

breakpoint

| | BA.2.3.13 | BA.1 | seq_change | t_pos |
|---|---|---|---|---|
| 26270_C\|T | True | True | True | 58 |
| 26530_A\|G | False | True | False | 58 |
| 26577_C\|G | True | True | True | 59 |
| 26709_G\|A | True | True | True | 60 |
| 26858_C\|T | True | False | False | 60 |
| 27259_A\|C | True | True | True | 61 |
| 27382_GAT\|CTC | True | False | False | 61 |
| 27807_C\|T | True | True | True | 62 |
| 28271_A\|T | True | True | True | 63 |
| 28311_C\|T | True | True | True | 64 |
| 28362_GAGAACGCA\|......... | True | True | True | 65 |
| 28881_GGG\|AAC | True | True | True | 66 |
| 29510_A\|C | True | False | False | 66 |

candidate L1 is same as GT L1.
And has 3 mutations more than BA.1 (L2 from the ground truth) after the breakpoint.

# XAW

- XAW: RecombinHunt struggles to find any good candidate for the enclosed region (BA.2 according to the GT)

**Case 50 (2BP): XAW** **test: KO**

Target: (75%) 28 samples Number of changes: 103
GT: AY.122 + BA.2* + AY.122 GT BR: 48-53, 91-93 GT BR coord: 22035 - 22588, 28270 - 28312 Rank_L1_L2: 1 11 1
BC: AY.122 + BQ.1.12 + AY.122 BC BR: 51-52, 97-98 BC BR coord: 22291 - 22292, 28880 - 28881
Direction L1: >> Initial region span: 1-48,96-97,98-103 Gap history (edge excluded): 48-98 -> 48-96
Alt. candidates: [], []
Model 1BP/2BP comparison: 2BP vs 1BP: 9.82e-83
Rec. model vs L1: 8.83e-102 Rec. model vs L2: 0.00e+00
Flags: Model_2BP_Best

**AY.122 >>**

| | num_seq | t_ch_MAX | max_CL | CL@BC_t_ch_MAX | aic | PV | PV_OK | t_ch_MAX_OK | phyl_OK |
|---|---|---|---|---|---|---|---|---|---|
| **AY.122** | 80528 | 48 | 19.968974 | NaN | NaN | NaN | * | * | * |
| **AY.28** | 280 | 3 | 6.864636 | -378.477802 | 942.955603 | 7.921939e-172 | | | |
| **AY.117** | 7391 | 3 | 4.769573 | -243.151582 | 672.303163 | 4.697900e-113 | | | |
| **AY.124.1** | 562 | 3 | 3.836279 | -309.733224 | 805.466448 | 5.680750e-142 | | | |
| **AY.99.2** | 14345 | 5 | 2.768780 | -148.744584 | 483.489167 | 4.693257e-72 | | | |
| **AY.43.1** | 697 | 3 | 2.732541 | -303.742955 | 793.485910 | 2.268974e-139 | | | |
| **AY.42** | 16868 | 3 | 2.717974 | -182.997481 | 551.994963 | 6.264527e-87 | | | |
| **AY.34.1** | 3230 | 3 | 2.699284 | -211.808617 | 609.617234 | 1.917312e-99 | | | |
| **AY.33** | 10326 | 3 | 2.686574 | -136.373318 | 458.746637 | 1.105852e-66 | | | |
| **AY.25** | 47119 | 37 | 2.585180 | -31.645088 | 249.290176 | 3.367892e-21 | | | |

**BQ.1.12 <<**

| | num_seq | t_ch_MAX | max_CL | CL@BC_t_ch_MAX | aic | PV | PV_OK | t_ch_MAX_OK | phyl_OK |
|---|---|---|---|---|---|---|---|---|---|
| **BQ.1.12** | 2239 | 96 | 7.499143 | NaN | NaN | NaN | * | * | * |
| **C.37** | 5858 | 96 | 4.944372 | -360.244348 | 920.488695 | 1.405005e-118 | | * | |
| **B.1.1.525** | 39 | 97 | 4.432309 | -407.727328 | 1015.454657 | 3.368030e-139 | | * | |
| **AY.103** | 145033 | 91 | 3.843814 | -292.321496 | 784.642991 | 4.437421e-89 | | | |
| **AZ.2** | 794 | 97 | 3.199799 | -433.501875 | 1067.003749 | 2.154942e-150 | | * | |
| **BF.11.5** | 292 | 97 | 3.093161 | -164.094019 | 528.188038 | 2.160432e-33 | | * | |
| **AY.90** | 568 | 96 | 3.034895 | -420.848098 | 1041.696197 | 6.718322e-145 | | * | |
| **AY.43** | 140119 | 97 | 2.909475 | -329.896904 | 859.793809 | 2.130666e-105 | | * | |
| **BE.1.1.1** | 1136 | 96 | 2.882548 | -121.648006 | 443.296012 | 5.863674e-15 | | * | * |
| **BN.1.7** | 411 | 97 | 2.754229 | -149.234017 | 498.468033 | 6.139833e-27 | | * | |

**AY.122 <<**

| | num_seq | t_ch_MAX | max_CL | CL@BC_t_ch_MAX | aic | PV | PV_OK | t_ch_MAX_OK | phyl_OK |
|---|---|---|---|---|---|---|---|---|---|
| **AY.122** | 80528 | 98 | 4.982981 | None | None | None | * | * | * |

not AY.122, neither BQ.1.12 are well suited for the central region, despite this being the best model for the target sequence (BQ.1.12 has the highest likelihood in this region)

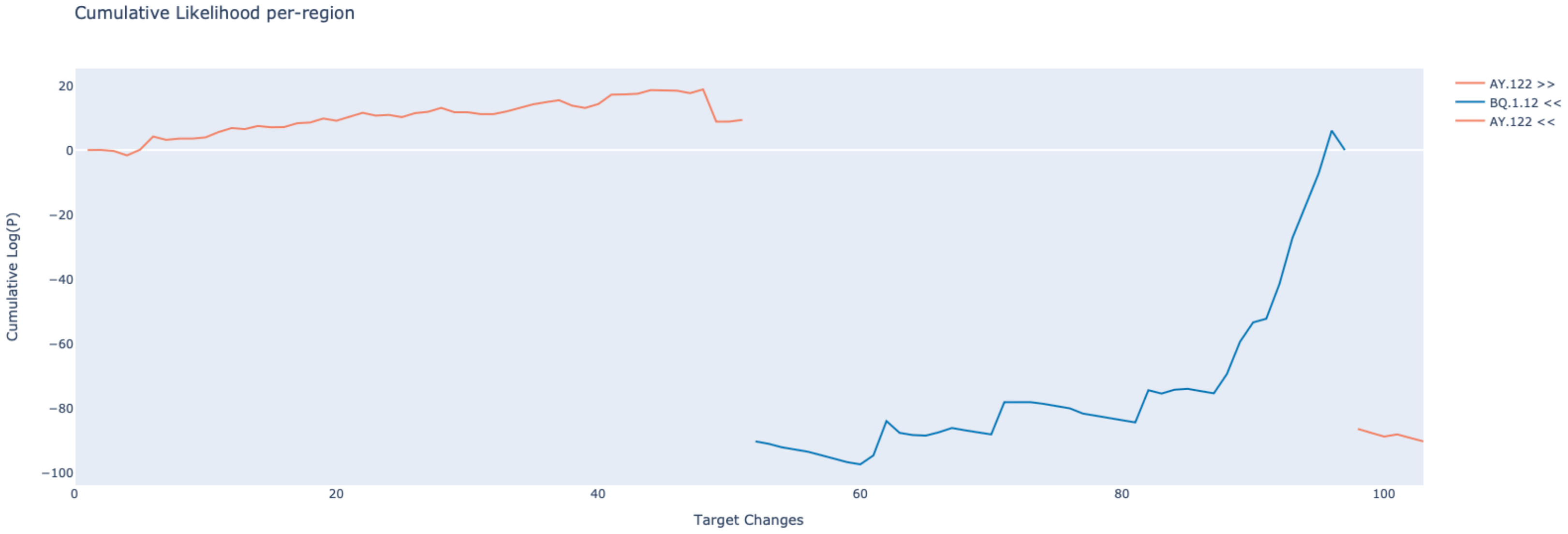

# XP

Target: (75%) 12 samples
GT: BA.1.1* + BA.2*
BC: BA.1.1
Direction L1: >>
Alt. candidates: []
Model 1BP/2BP comparison: -
Rec. model vs L1: -
Flags: NotEnoughSpaceAfterL1, SingleCandidateGenome

Number of changes: 66
GT BR: 58-65
BC BR:
Initial region span: 1-64
Rec. model vs L2: -

GT BR coord: 27384 - 29510
BC BR coord:
Gap history (edge excluded):

Rank L1 L2: 1 -

candidate L1 initially ranges from mutation 1 to 64, leaving only two mutations for L2. Hence the non-recombinant model is proposed.

| | seq_change | BA.1.1 | t_pos | P(BA.1.1) | P(change) |
|---|---|---|---|---|---|
| 26709_G\|A | True | True | 56 | 0.995051 | 0.001563 |
| 26880_C\|A | True | False | 57 | 0.000006 | 0.002689 |
| 27259_A\|C | True | True | 58 | 0.996133 | 0.00012 |
| 27807_C\|T | True | True | 59 | 0.995185 | 0.000125 |
| 28254_A\|. | True | False | 60 | 0.007113 | 0.000108 |
| 28271_A\|T | True | True | 61 | 0.997616 | 0.000135 |
| 28311_C\|T | True | True | 62 | 0.994304 | 0.025659 |
| 28362_GAGAACGCA\|......... | True | True | 63 | 0.951261 | 0.458477 |
| 28881_GGG\|AAC | True | True | 64 | 0.989128 | 0.590661 |
| 29510_A\|C | True | False | 65 | 0.000043 | 0.341634 |
| 29734_GAGGCCACGCGGAGTA( | True | False | 66 | 0.000069 | 0.002785 |

the recognition of the breakpoint is made difficult by the high frequency of the terminal changes in BA.1.1,

and the unsufficient likelihood drop generated by the last two mutations

**Supplementary Notes 6**

Detailed report on Nextstrain cases on which RH disagrees with Pango lineage GT. This document extends the discussion presented in the ‘Comparison with the RIPPLES method’ section.

# Detailed report on Nextstrain cases on which RH disagrees with Pango lineage GT

# Summary of contents

- 61 cases found (with any number of "good" sequences available up to March 30st):
  - 1BP: 44
  - 2BP: 7
  - 3BP or more: 1 (XAY)
  - 1/2BP with ill-defined ground truth: 9 (XB, XAS, XAJ, XBK, XBN, XBQ, XBS, XBV, XCA), i,e. cases with uncertain donor/acceptor lineages or breakpoint position
- We discuss the comparison of 1BP and 2BP results only for cases where the ground truth is sound: 51 cases.
- We reduced input noise using the consensus sequence
- Results
  - 43 cases mathcing the candidatess from the ground truth
    - 38 cases single breakpoint (including 2 cases in a close ancestor lineage to that reported in the ground truth is selected)
    - 5 cases double breakpoint
  - 8 cases needing discussion divided in three groups:

    G1: 5 cases that we judge being not recombinant (recombination is based on 1/2 mutations: XAV, XAR, XN, XAK, XAZ)

    G2: 1 case where our method suggests a more probable model (XBH)

    G3: 2 cases highlights the limits of our approach (XAT and XP)

# Discussion on cases with close ancestor lineage

2 cases

# Superlineage candidate - case XBB

GT:  BJ.1 + BM.1.1.1

BC: BA.2.9 + BM.1.1.1

Why?

- Nextstrain is missing BJ.1 characterization.

- BJ.1 = BA.2.10.1.1.

- It's closest ancestor available in Nextstrain is BA.2.10.1. However, observation of GISAID data reveals large differences between the two lineages: 10 mutations less than BJ.1

- Comparison of BA.2.9 and BA.2.10.1 on target changes before breakpoint and Nextstrain data, show only 1 change less than BA.2.10 at position 24. So the two lineages are quite close.

GISAID data

1-10 32 rows × 2 columns

| | BJ.1 | BA.2.10.1 |
|---|---|---|
| 241_C\|T | True | False |
| 405_A\|G | True | False |
| 15451_G\|A | True | False |
| 15738_C\|T | True | False |
| 15939_T\|C | True | False |
| 17859_T\|C | True | False |
| 21810_T\|C | True | False |
| 21987_G\|A | True | False |
| 21991_TTA\|... | True | False |
| 22000_C\|A | True | False |

# Superlineage candidate - case XM

GT: BA.1.1* + BA.2*

BC: BA.1.24 + BA.2.27

Comparison of BA.1.24 and BA.1.1 before breakpoint on target changes show no difference.

BA.1.24 is chosen because of greater similarity on change 2470_C|T

13 rows ⌄ 13 rows × 4 columns

<table>
<tr><th></th><th>BA.1.1</th><th>BA.1.24</th><th>seq_change</th><th>t_pos</th></tr>
<tr><td>241_C|T</td><td>True</td><td>True</td><td>True</td><td>1</td></tr>
<tr><td>2470_C|T</td><td>True</td><td>False</td><td>False</td><td>1</td></tr>
<tr><td>2832_A|G</td><td>True</td><td>True</td><td>True</td><td>2</td></tr>
<tr><td>3037_C|T</td><td>True</td><td>True</td><td>True</td><td>3</td></tr>
<tr><td>5386_T|G</td><td>True</td><td>True</td><td>True</td><td>4</td></tr>
<tr><td>6513_6515</td><td>True</td><td>True</td><td>True</td><td>5</td></tr>
<tr><td>8393_G|A</td><td>True</td><td>True</td><td>True</td><td>6</td></tr>
<tr><td>10029_C|T</td><td>True</td><td>True</td><td>True</td><td>7</td></tr>
<tr><td>10449_C|A</td><td>True</td><td>True</td><td>True</td><td>8</td></tr>
<tr><td>11285_11293</td><td>True</td><td>True</td><td>True</td><td>9</td></tr>
<tr><td>11537_A|G</td><td>True</td><td>True</td><td>True</td><td>10</td></tr>
<tr><td>13195_T|C</td><td>True</td><td>True</td><td>True</td><td>11</td></tr>
<tr><td>14408_C|T</td><td>True</td><td>True</td><td>True</td><td>12</td></tr>
</table>

# Discussion on G1 cases

Non-recombinant cases

# XAR

**Case 32 (1BP 5'): XAR** **test: KO**

Target: (75%) 65 samples
Number of changes: 69
GT: BA.1* + BA.2*
GT BR: 2-4
GT BR coord: 2833 - 4184
Rank L1 L2: 11 -
BC: BA.2.23
BC BR:
BC BR coord:
Direction L1: <<
Initial region span: 3-69
Gap history (edge excluded):
Alt. candidates: [BA.2]
Model 1BP/2BP comparison: -
Rec. model vs L1: -
Rec. model vs L2: -
Flags: NotEnoughSpaceAfterL1, SingleCandidateGenome

# XN

**Case 39 (1BP 5'): XN** **test: KO**

Target: (75%) 167 samples
Number of changes: 71
GT: BA.1* + BA.2*
GT BR: 2-4
GT BR coord: 2833 - 4184
Rank L1 L2: 11 -
BC: BA.2
BC BR:
BC BR coord:
Direction L1: <<
Initial region span: 3-71
Gap history (edge excluded):
Alt. candidates: [BA.2.9]
Model 1BP/2BP comparison: -
Rec. model vs L1: -
Rec. model vs L2: -
Flags: NotEnoughSpaceAfterL1, SingleCandidateGenome

These two cases have a recombination in positions 2-4 according to the ground truth. indeed RecombinHunt finds the maximum of the candidates from 3' at target position 3. As only 2 target changes are left, RecombinHunt rejects the recombinant hypothesis.

# XAK

**Case 48 (2BP): XAK** **test: KO**

Target: 5 samples
Number of changes: 73
GT: BA.2* + BA.1* + BA.2*
GT BR: 20-23, 23-24
GT BR coord: 13194 - 15241, 21617 - 21763
Rank L1 L2: 1 11 -
BC: BA.2 + BA.2.10
BC BR: 26-27
BC BR coord: 22001 - 22002
Direction L1: <<
Initial region span: 1-18,39-73
Gap history (edge excluded): 18-39
Alt. candidates: [], [BA.2]
Model 1BP/2BP comparison: 1BP vs 2BP: 2.38e-10
Rec. model vs L1: 1.96e+03
Rec. model vs L2: 6.79e-12
Flags: Model_1BP_L1eqL2, Model_1BP_Best

**BA.2 >>**

| | num_seq | t_ch_MAX | max_CL | CL@BC_t_ch_MAX | aic | PV | PV_OK | t_ch_MAX_OK | phyl_OK |
|---|---|---|---|---|---|---|---|---|---|
| **BA.2** | 359165 | 18 | 8.791758 | NaN | NaN | NaN | * | * | * |
| **BA.2.36** | 1320 | 18 | 7.729049 | -39.558170 | 191.116341 | 3.083889e-14 | | * | * |
| **BF.20** | 26 | 7 | 7.574204 | -65.465163 | 242.930326 | 1.732607e-25 | | | |
| **BA.2.31.1** | 54 | 7 | 7.272938 | -70.966287 | 253.932574 | 7.080770e-28 | | | * |
| **BA.2.72** | 268 | 7 | 6.360344 | -48.458882 | 208.917764 | 4.206082e-18 | | | * |
| **BA.5.2.14** | 108 | 7 | 5.936329 | -83.533876 | 279.067752 | 2.460361e-33 | | | |
| **BA.5.1.10** | 2819 | 7 | 5.386913 | -36.802774 | 185.605548 | 4.848194e-13 | | | |
| **XBB.1.5.4** | 250 | 7 | 5.381937 | -86.586952 | 285.173904 | 1.165200e-34 | | | |
| **BA.2.20** | 357 | 7 | 5.378569 | -41.872366 | 195.744731 | 3.061106e-15 | | | * |
| **BA.2.10.1** | 1150 | 7 | 5.310407 | -44.759845 | 201.519690 | 1.701247e-16 | | | * |

**BA.2.10 <<**

| | num_seq | t_ch_MAX | max_CL | CL@BC_t_ch_MAX | aic | PV | PV_OK | t_ch_MAX_OK | phyl_OK |
|---|---|---|---|---|---|---|---|---|---|
| **BA.2.10** | 12936 | 39 | 26.936405 | NaN | NaN | NaN | * | * | * |
| **BA.2** | 359165 | 39 | 26.286362 | 21.433721 | 111.132557 | 4.274149e-01 | * | * | * |
| **BA.2.75.5** | 215 | 30 | 23.447112 | -16.639178 | 187.278356 | 1.244765e-17 | | | |
| **BA.2.9** | 61803 | 39 | 20.664009 | 13.239560 | 127.520880 | 1.179795e-04 | * | * | |
| **BA.2.12.1** | 94944 | 39 | 19.909427 | 10.756585 | 132.486830 | 9.830704e-06 | | * | |
| **BN.1.7** | 232 | 30 | 18.453253 | -35.609014 | 225.218029 | 7.186559e-26 | | | |
| **BA.2.1** | 7140 | 39 | 17.973804 | 8.395319 | 137.209361 | 9.282173e-07 | | * | * |
| **BN.1** | 942 | 30 | 17.824779 | -25.446833 | 204.893665 | 1.866914e-21 | | | |
| **BA.2.76** | 356 | 39 | 16.204563 | -5.488905 | 164.977811 | 8.659060e-13 | | * | |
| **XBB.1.5** | 35885 | 42 | 15.196244 | -37.448706 | 228.897412 | 1.141351e-26 | | | |

Here the length of region assigned to L2 candidate BA.1 is too short as the breakpoints are 20-23 and 23-24 according to the ground truth.
Therefore the candidate L2 cannot be found.

Furthermore, as the two candidates of the proposed 1BP model (BA.2 and BA.2.10) are close linegaes, we classify this case as not recombinant.

# XAZ

**Case 49 (2BP): XAZ** **test: KO**

Target: (75%) 289 samples
GT: BA.2.5 + BA.5 + BA.2.5
BC: BA.5.1.27 + BA.5
Direction L1: <<
Alt. candidates: [], [BA.5.3, BA.5.3.1, BA.5.5, BA.5.11, BA.5.3.2, BA.5.2.1, B.1.1.529]
Model 1BP/2BP comparison: -
Rec. model vs L1: 8.71e-21
Flags: Model_1BP_L1eqL2, Model_2BP_Bad_L1_opp

Number of changes: 71
GT BR: 8-14, 63-64
BC BR: 7-8
Initial region span: 1-7,9-71
Rec. model vs L2: 2.37e-13

GT BR coord: 3358 - 9866, 27384 - 27387
BC BR coord: 3323 - 3324
Gap history (edge excluded): 7-9

Rank L1 L2: 11 1 -

Cumulative Likelihood per-region

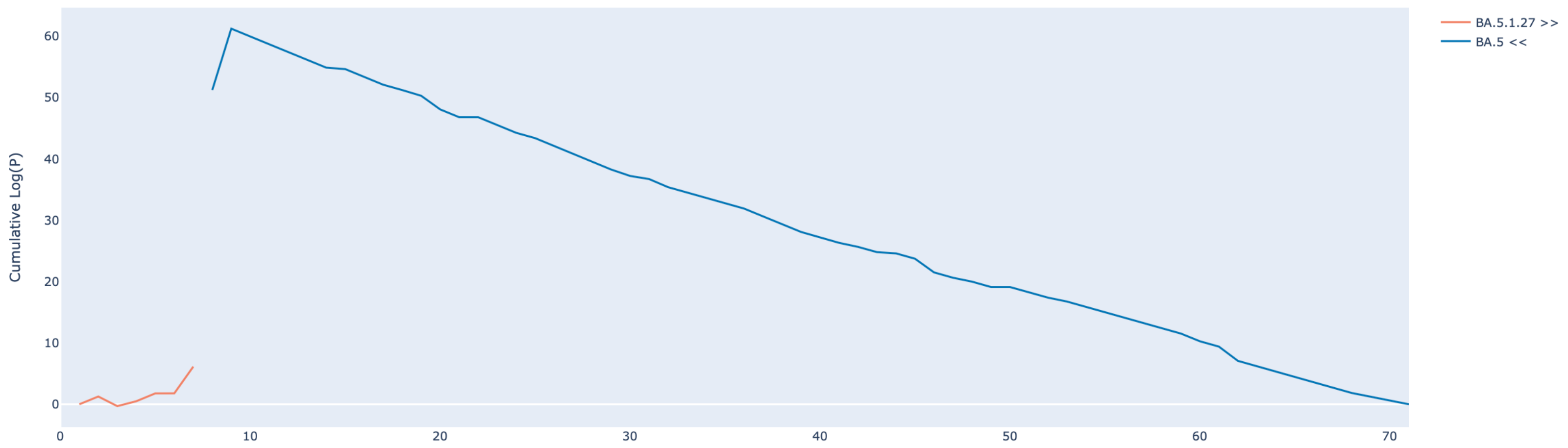


Apaprently the second breakpoint (63-64) is not detected. This is due to the similarity of seq_change with BA.5 in the region between the breakpoints (9-62). After the comparison of the target changes with the characterisation of BA.5 we can confirm the list of changes is identical.
Furthermore, as the two candidates of the proposed 1BP model (BA.5.1.27 and BA.5) are close linegaes, we classify this case as not recombinant.

# XAV

**Target**: (75%) 19 samples
**GT**: BA.2* + BA.5*
**BC**: BA.5.1.24
**Direction L1:** >>
**Alt. candidates**: [BA.5.1]
**Model 1BP/2BP comparison**: -
**Rec. model vs L1**: -
**Flags**: NotEnoughSpaceAfterL1, SingleCandidateGenome

**Number of changes**: 72
**GT BR**: 19-22 **GT BR coord**: 15959 - 17279 **Rank L1 L2**: 11 -
**BC BR**: **BC BR coord**:
**Initial region span**: 1-71 **Gap history (edge excluded)**:
**Rec. model vs L2**: -

Cumulative Likelihood per-region

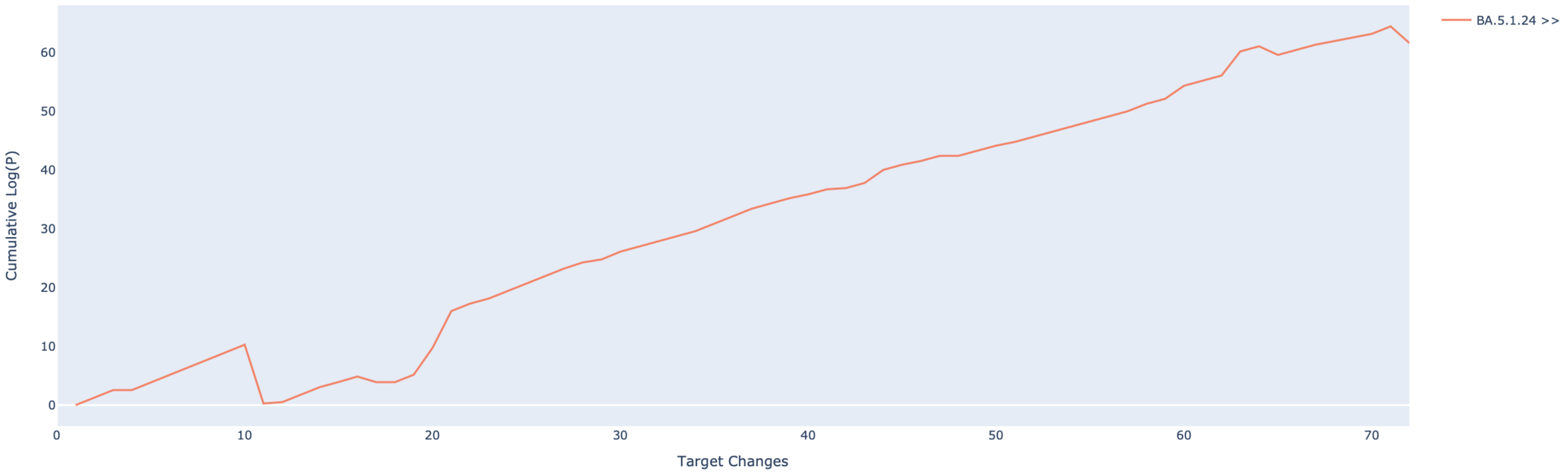


| | seq_change | BA.2 | BA.5 | BA.5.1.24 | t_pos | P(BA.2) | P(BA.5) | P(BA.5.1.24) | P(change) |
|---|---|---|---|---|---|---|---|---|---|
| 241_C\|T | True | True | True | True | 1 | 0.998082 | 0.993579 | 0.998374 | 0.995997 |
| 670_T\|G | True | True | True | True | 2 | 0.998499 | 0.996497 | 1.000000 | 0.276872 |
| 2790_C\|T | True | True | True | True | 3 | 0.999365 | 0.999416 | 1.000000 | 0.279530 |
| 3037_C\|T | True | True | True | True | 4 | 0.997419 | 1.000000 | 1.000000 | 0.996100 |
| 4184_G\|A | True | True | True | True | 5 | 0.999889 | 0.999708 | 1.000000 | 0.279692 |
| 4321_C\|T | True | True | True | True | 6 | 0.999026 | 0.998249 | 1.000000 | 0.283115 |
| 6606_C\|T | True | False | False | False | 7 | 0.000381 | 0.000000 | 0.004065 | 0.001050 |
| 9344_C\|T | True | True | True | True | 8 | 0.999490 | 0.998249 | 1.000000 | 0.280342 |
| 9424_A\|G | True | True | True | True | 9 | 0.999326 | 0.997665 | 0.996748 | 0.279324 |
| 9534_C\|T | True | True | True | True | 10 | 0.997241 | 0.996497 | 1.000000 | 0.279295 |
| 9866_C\|T | True | True | False | False | 11 | 0.987532 | 0.001168 | 0.000000 | 0.170493 |
| 10029_C\|T | True | True | True | True | 12 | 0.999880 | 1.000000 | 1.000000 | 0.790055 |
| 10198_C\|T | True | True | True | True | 13 | 0.994593 | 1.000000 | 1.000000 | 0.279435 |
| 10447_G\|A | True | True | True | True | 14 | 0.999755 | 0.998249 | 1.000000 | 0.279771 |
| 10449_C\|A | True | True | True | True | 15 | 0.999861 | 0.998249 | 1.000000 | 0.418601 |
| 11288_11296 | True | True | True | True | 16 | 0.991177 | 0.997957 | 0.997561 | 0.393335 |
| 12160_G\|A | False | False | True | True | 16 | 0.000267 | 0.997373 | 1.000000 | 0.108238 |
| 12880_C\|T | True | True | True | True | 17 | 0.999827 | 1.000000 | 1.000000 | 0.281641 |
| 14408_C\|T | True | True | True | True | 18 | 0.999808 | 1.000000 | 1.000000 | 0.997400 |
| 15714_C\|T | True | True | True | True | 19 | 0.999866 | 1.000000 | 1.000000 | 0.280795 |
| 15960_C\|T | True | False | False | False | 20 | 0.002342 | 0.004086 | 0.220325 | 0.002297 |
| 17278_G\|T | True | False | False | True | 21 | 0.000198 | 0.004670 | 1.000000 | 0.001910 |

Before the breakpoint (19-22) the BC BA.5.1.24 and the GT candidate BA.2 differ by only few mutations

- The target has 1 characteristic mutation of BA.2 which is not in BA.5.1.26

- The target has one mutation less than BA.5.1.24

- The target has 2 private mutations more than BA.5.1.26 that are not characteristic of BA.2.

# Discussion on G2 cases

More probable models with respect to the ground truth

# XBH

Target: (75%) 10 samples
GT: BA.2.3.17 + BA.2.75.2
BC: BA.2.75.2 + BA.2.3.17 + BA.2.75.2
Direction L1: <<
Alt. candidates: [], [CA.3]
Model 1BP/2BP comparison: 2BP vs 1BP: 3.35e-03
Rec. model vs L1: 2.52e-68
Flags: Model_2BP_Best

Number of changes: 84
GT BR: 19-28
BC BR: 4-5, 19-20
Initial region span: 1-4,8-19,20-84
Rec. model vs L2: 3.16e-139

GT BR coord: 15450 - 22001
BC BR coord: 3037 - 3038, 15713 - 15714
Gap history (edge excluded): 4-20 -> 4-8

Rank L1 L2: 11 11 -

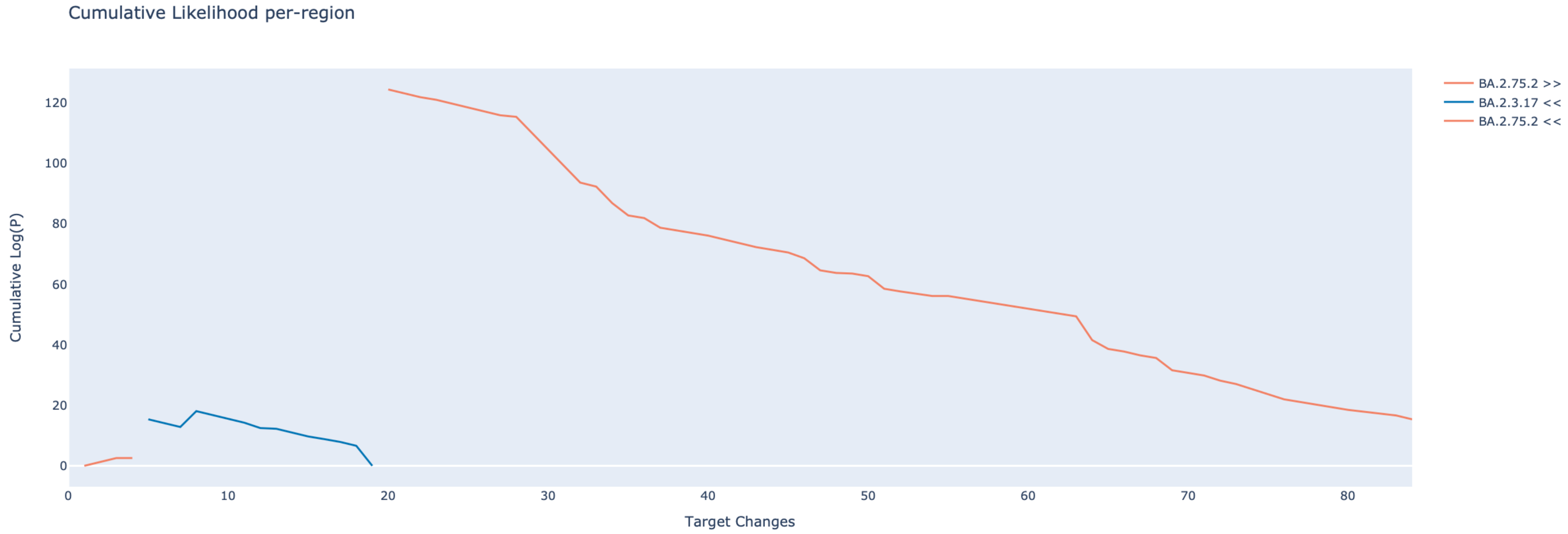


The proposed model is 2BP BA.2.75.2 + BA.2.3.17 + BA.2.75.2.
The candidates are same as in the ground truth.
The 2BP model is proposed by RecombinHunt because it wins the comparison against the 1BP model (3*10e-3)

# Discussion on G3 cases

Controversial cases

# XP

Target: (75%) 56 samples
Number of changes: 68
GT: BA.1.1* + BA.2*
GT BR: 58-67
GT BR coord: 27384 - 29510
Rank_L1_L2: 1 11
BC: BA.1.1 + BA.5.6.3
BC BR: 59-60
BC BR coord: 27889 - 27890
Direction L1: >>
Initial region span: 1-56,60-68
Gap history (edge excluded): 56-60
Alt. candidates: [], [BA.5]
Model 1BP/2BP comparison: -
Rec. model vs L1: 3.88e-12
Rec. model vs L2: 0.00e+00

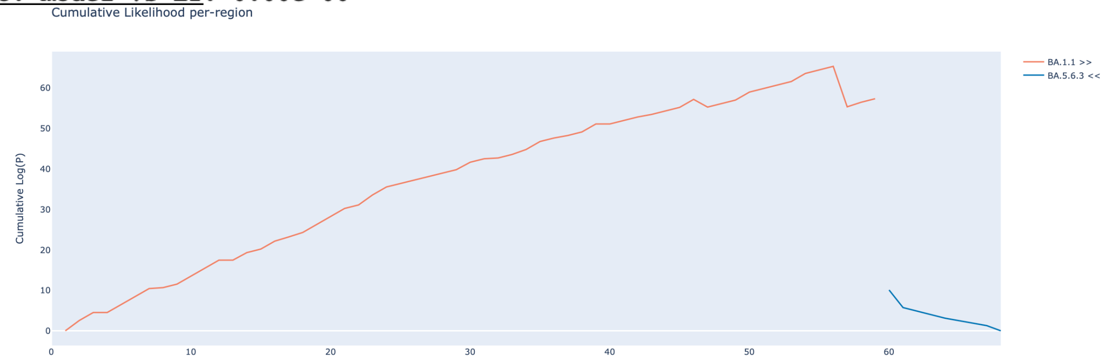


GT: BA.1.1 + BA.2

BC: BA.1.1 + BA.5.6.3

target: 68 changes

GT breakpoint between 58-67

BC breakpoint between 58-59

The breakpoint region (according to the GT) extends to 1 target mutation from the end of the genome, leaving too little information to correctly guide the identification of the candidate.

BA.5.6.3 seems a better candidate overall if we consider the probability of the changes between the breakpoint and the 3' edge (i.e., positions 59-68).

The probability of target changes 61-67 (included) is 100% for BA.5.6.3, and lower for BA.2. The result is justified by the higher probability of BA.5.6.3 changes, even if it may be biased because the characterisation is obtained from 56 samples.

| | seq_change | BA.1.1 | BA.2 | BA.5.6.3 | t_pos | P(BA.1.1) | P(BA.2) | P(BA.5.6.3) | P(change) |
|---|---|---|---|---|---|---|---|---|---|
| 27807_C\|T | True | True | True | True | 59 | 0.999825 | 0.999248 | 1.000000 | 0.419202 |
| 27889_C\|T | False | False | False | True | 59 | 0.000122 | 0.000084 | 1.000000 | 0.095890 |
| 28254_28254 | True | False | False | False | 60 | 0.005347 | 0.001910 | 0.543478 | 0.007057 |
| 28271_A\|T | True | True | True | True | 61 | 0.999544 | 0.999950 | 1.000000 | 0.418979 |
| 28311_C\|T | True | True | True | True | 62 | 0.997239 | 0.999688 | 1.000000 | 0.419669 |
| 28362_28370 | True | True | True | True | 63 | 0.986844 | 0.995058 | 1.000000 | 0.415939 |
| 28881_G\|A | True | True | True | True | 64 | 0.996258 | 0.998803 | 1.000000 | 0.542031 |
| 28882_G\|A | True | True | True | True | 65 | 0.991739 | 0.989002 | 1.000000 | 0.539845 |
| 28883_G\|C | True | True | True | True | 66 | 0.991986 | 0.989314 | 1.000000 | 0.540192 |
| 29510_A\|C | True | False | True | True | 67 | 0.000019 | 0.999251 | 1.000000 | 0.279864 |
| 29734_29759 | True | False | False | True | 68 | 0.000008 | 0.748515 | 0.956522 | 0.233687 |

# XAT

**Target**: 3 samples
**GT**: BA.2.3.13 + BA.1*
**BC**: BA.2.3
**Direction L1:** >>
**Alt. candidates**: [BA.2.3.9, BA.2.3.16, BA.2.3.5, BA.2.3.10, BA.2]
**Model 1BP/2BP comparison**:
**Rec. model vs L1**: -
**Flags**: Model_2BP_NotEnoughSpace_ForL2, Model_2BP_Bad_L2, SingleCandidateGenome

**Number of changes**: 66
**GT BR**: 55-57 **GT BR coord**: 26061 - 26529 **Rank L1 L2**: 11 -
**BC BR**: **BC BR coord**:
**Initial region span**: 1-58 **Gap history (edge excluded)**:
-
**Rec. model vs L2**: -

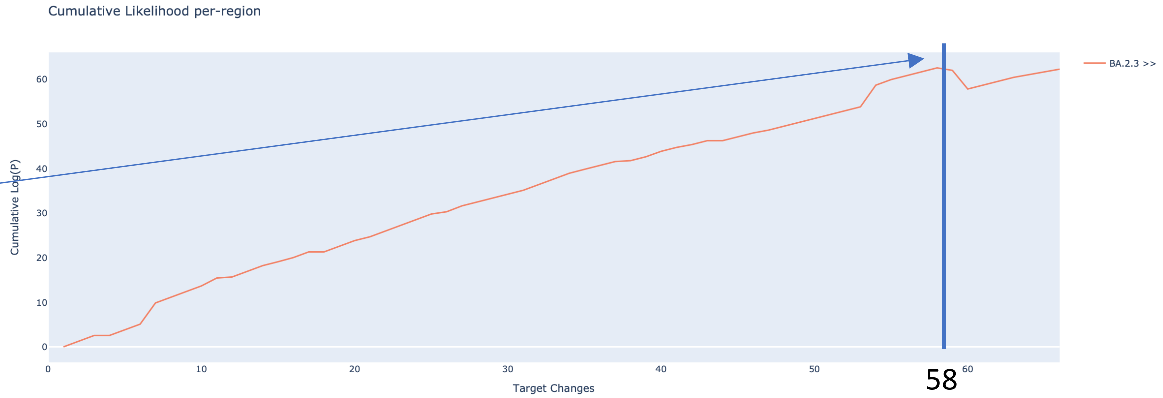


Here, candidate L1 BA.2.3 is compatible with the target both at 5' (from 1 to 58)

and at 3' (from 60 to 66) ( we know it because of the maximum of the cumulative likelihood CL_BA.2.3).

| | seq_change | BA.2.3.13 | BA.1 | BA.2.3 | t_pos | CL_BA.2.3 |
|---|---|---|---|---|---|---|
| 26709_G\|A | True | True | True | True | 58 | -2.091981 |
| 26858_C\|T | False | True | False | True | 58 | -2.961069 |
| 27259_A\|C | True | True | True | True | 59 | -1.274722 |
| 27382_G\|C | False | True | False | True | 59 | -2.405563 |
| 27383_A\|T | False | True | False | True | 59 | -0.719054 |
| 27384_T\|C | False | False | False | True | 59 | 0.972860 |
| 27807_C\|T | True | True | True | True | 60 | 2.639408 |
| 28271_A\|T | True | True | True | True | 61 | 1.770183 |
| 28311_C\|T | True | True | True | True | 62 | 0.900472 |
| 28362_28370 | True | True | True | True | 63 | 0.033516 |
| 28881_G\|A | True | True | True | True | 64 | -0.839384 |
| 28882_G\|A | True | True | True | True | 65 | -1.450039 |
| 28883_G\|C | True | True | True | True | 66 | -2.062596 |
| 29510_A\|C | False | True | False | True | 66 | -2.674510 |
| 29734_29759 | False | True | False | True | 66 | -1.401770 |

Therefore, the 2BP model is a valid option for this sequence. However, since only 1 change (59th) is left to search the second candidate L2, the 2BP model is refused and a non-recombinant model BA.2.3 is proposed instead.
