# Supplementary material for: Data-driven recombination detection in viral genomes
Source: Nat Commun. 2024 Apr 17;15:3313. doi: 10.1038/s41467-024-47464-5 (PMC11024102; doi:10.1038/s41467-024-47464-5)
Supplement: Supplementary file 3 — Description of Additional Supplementary Files [file 41467_2024_47464_MOESM3_ESM.pdf]

Description of additional supplementary

Title: SupplementaryData1_ sensitivity_specificity_analysis.tsv
Description: Dataset of 10,500 simulated sequences to perform the sensitivity and specificity analysis reported in the 'Sensitivity, Specificity, Minimum Requirements' section.

Title: SupplementaryData2_nextstrain_mpox_all_recombinants_accession_ids.txt
Description: This dataset complements the information presented in the 'RecombinHunt on monkeypox' section.
