# Supplementary material for: Data-driven recombination detection in viral genomes
Source: Nat Commun. 2024 Apr 17;15:3313. doi: 10.1038/s41467-024-47464-5 (PMC11024102; doi:10.1038/s41467-024-47464-5)
Supplement: Supplementary file 6 — Reporting Summary [file 41467_2024_47464_MOESM6_ESM.pdf]

nature portfolio

Corresponding author(s): Anna Bernasconi

Last updated by author(s): Mar 14, 2024

Reporting Summary

Nature Portfolio wishes to improve the reproducibility of the work that we publish. This form provides structure for consistency and transparency in reporting. For further information on Nature Portfolio policies, see our Editorial Policies and the Editorial Policy Checklist.

Statistics

For all statistical analyses, confirm that the following items are present in the figure legend, table legend, main text, or Methods section.

n/a	Confirmed	
[ ]	[x]	The exact sample size (*n*) for each experimental group/condition, given as a discrete number and unit of measurement
[x]	[ ]	A statement on whether measurements were taken from distinct samples or whether the same sample was measured repeatedly
[ ]	[x]	The statistical test(s) used AND whether they are one- or two-sided<br>*Only common tests should be described solely by name; describe more complex techniques in the Methods section.*
[x]	[ ]	A description of all covariates tested
[x]	[ ]	A description of any assumptions or corrections, such as tests of normality and adjustment for multiple comparisons
[ ]	[x]	A full description of the statistical parameters including central tendency (e.g. means) or other basic estimates (e.g. regression coefficient) AND variation (e.g. standard deviation) or associated estimates of uncertainty (e.g. confidence intervals)
[ ]	[x]	For null hypothesis testing, the test statistic (e.g. *F*, *t*, *r*) with confidence intervals, effect sizes, degrees of freedom and *P* value noted<br>*Give P values as exact values whenever suitable.*
[x]	[ ]	For Bayesian analysis, information on the choice of priors and Markov chain Monte Carlo settings
[x]	[ ]	For hierarchical and complex designs, identification of the appropriate level for tests and full reporting of outcomes
[x]	[ ]	Estimates of effect sizes (e.g. Cohen's *d*, Pearson's *r*), indicating how they were calculated

Our web collection on statistics for biologists contains articles on many of the points above.

Software and code

Policy information about availability of computer code

Data collection	Employed data was downloaded from public databases. No particular software was used for collection.
Data analysis	The implementation was performed with Python v3.10.12.<br>Data filtering and numerical analyses use the libraries numpy (v1.26.0), pandas (v2.1.1), and tqdm (v4.66.1).<br>Outputs are prepared using, additionally, plotly (v5.17.0), inflect (v6.0.2), tabulate (v0.9.0), and kaleido (0.2.1).<br>The code is documented on a Zenodo repository https://zenodo.org/doi/10.5281/zenodo.8123832, available in Jupyter notebooks (prepared using jupyter v1.0.0).

For manuscripts utilizing custom algorithms or software that are central to the research but not yet described in published literature, software must be made available to editors and reviewers. We strongly encourage code deposition in a community repository (e.g. GitHub). See the Nature Portfolio guidelines for submitting code & software for further information.

Data

Policy information about availability of data

All manuscripts must include a data availability statement. This statement should provide the following information, where applicable:

- Accession codes, unique identifiers, or web links for publicly available datasets
- A description of any restrictions on data availability
- For clinical datasets or third party data, please ensure that the statement adheres to our policy

The simulated dataset for sensitivity/specificity analysis generated in this study is provided in Supplementary Data 1.
The recombinant genomes identified by RecombinHunt for monkeypox are provided in Supplementary Data 2.
Original sequences and metadata of SARS-CoV-2 used in this work are accessible through the GISAID (https://www.gisaid.org/) and Nextstrain (https://nextstrain.org/ncov/open/global) databases.
Original sequences and metadata of mpox used in this work are accessible on the Nextstrain database (https://nextstrain.org/monkeypox/hmpxv1).
The datasets were downloaded on April 1st, 2023.
The lists of considered accession ids are provided on the Zenodo database under accession code https://zenodo.org/doi/10.5281/zenodo.8123832.

Research involving human participants, their data, or biological material

Policy information about studies with human participants or human data. See also policy information about sex, gender (identity/presentation), and sexual orientation and race, ethnicity and racism.

Reporting on sex and gender	not applicable
Reporting on race, ethnicity, or other socially relevant groupings	not applicable
Population characteristics	not applicable
Recruitment	not applicable
Ethics oversight	not applicable

Note that full information on the approval of the study protocol must also be provided in the manuscript.

Field-specific reporting

Please select the one below that is the best fit for your research. If you are not sure, read the appropriate sections before making your selection.

☐ Life sciences ☐ Behavioural & social sciences ☒ Ecological, evolutionary & environmental sciences

For a reference copy of the document with all sections, see nature.com/documents/nr-reporting-summary-flat.pdf

Ecological, evolutionary & environmental sciences study design

All studies must disclose on these points even when the disclosure is negative.

Study description	The study does not involve quantitative data.
Research sample	Our study is based on two existing datasets of SARS-CoV-2 sequences - respectively shared via GISAID and Nextstrain - and on one existing dataset of monkeypox sequences - shared via Nextstrain. All the available data were retrieved; low quality sequences were subsequently discarded.
Sampling strategy	After discarding low quality sequences (see Research sample), all sequences were considered suitable for the study; no sampling strategy was applied.
Data collection	We downloaded all the genome sequences from the public databases; genomes had already been sequenced and analyzed.
Timing and spatial scale	The considered SARS-CoV-2 genome sequences from GISAID are within collection dates [01.01.2020-21.03-2023]; from Nextstrain are within collection dates [23.12.2019-22.03.2023]. The considered mpox genome sequences from Nextstrain are within collection dates [09.10.2006-13.02-2023]. Sample periodicity is daily, when available from public databases; sequences are collected from all around the world.
Data exclusions	Original GISAID genome sequences were processed by the HaploCoV pipeline to derive a large table with the list of mutations and matched metadata for every genome sequence. Viral genomes identical to the reference sequence or having conflicting lineage assignments according to HaploCoV and GISAID were discarded. Records with incomplete metadata, labeled as low-coverage and/or associated with low-quality sequences (percentage of Ns < 2) were removed by our quality filters. The resulting datasets respectively contain 1) 5,255,228 records (GISAID SARS-CoV-2), 2) 3,984,308 records (Nextstrain SARS-CoV-2), and 3) 2,526 (Nextstrain mpox).

	More details in the 'Data collection and genome quality filtering' section in the Methods.
Reproducibility	The study is in silico; no wet-lab experiments were performed.
Randomization	Three strategies are applied according to the different analyses:<br>1) In the sensitivity/specificity analysis, simulated datasets of random SARS-CoV-2 sequences have been generated by adding 0, 3, 5, 10, 15, 20, or 30 mutations from a specific set and choosing a random breakpoint position.<br>2) For lineage-level analysis: randomization was not applied, as only one group was used.<br>3) For the genome-level analysis: we extracted 100 random samples of high quality sequences for every lineage, without resampling.<br>Allocation into experimental groups (corresponding to designation of recombinants) was made according to the Pango-lineage nomenclature.
Blinding	Blinding was not applied, as experiment bias cannot affect the results in this analysis.

Did the study involve field work? ☐ Yes ☒ No

Reporting for specific materials, systems and methods

We require information from authors about some types of materials, experimental systems and methods used in many studies. Here, indicate whether each material, system or method listed is relevant to your study. If you are not sure if a list item applies to your research, read the appropriate section before selecting a response.

Materials & experimental systems

n/a	Involved in the study	
☒	☐	Antibodies
☒	☐	Eukaryotic cell lines
☒	☐	Palaeontology and archaeology
☒	☐	Animals and other organisms
☒	☐	Clinical data
☒	☐	Dual use research of concern
☒	☐	Plants

Methods

n/a	Involved in the study	
☒	☐	ChIP-seq
☒	☐	Flow cytometry
☒	☐	MRI-based neuroimaging
